# Organocatalytic C─O Bond Cleavage and Asymmetric Transformations via [1,3]‐Sigmatropic Rearrangement

**DOI:** 10.1002/advs.202504718

**Published:** 2025-05-19

**Authors:** Lei Peng, Yu Chang, Liangchen Yin, Jinbang Zhang, Xuli Feng, Pengfei Wang, Wenling Qin, Hailong Yan

**Affiliations:** ^1^ Chongqing University FuLing Hospital No.2 Gaosuntang Road, Fuling District Chongqing 408000 P. R. China; ^2^ Chongqing Key Laboratory of Natural Product Synthesis and Drug Research School of Pharmaceutical Sciences Chongqing University Chongqing 401331 P. R. China

**Keywords:** [1,3]‐sigmatropic rearrangement, anti‐cancer activity, asymmetric organocatalysis, chiral benzofuran

## Abstract

Here, an organocatalytic asymmetric [1,3]‐sigmatropic rearrangement process for aryl ether insertion through C─O bond cleavage and downstream transformation is reported, enabling the practical and atom‐economic synthesis of diverse valuable chiral benzofuran derivatives bearing a quaternary carbon stereocenter. The reaction shows a wide substrate scope, yielding moderate to good products with excellent enantioselectivity and diastereoselectivity (up to >99% ee and >99:1 d.r.). Initial biological activity tests suggest that the resulting enantioenriched benzofuran products hold potential as anticancer agents.

## Graphical abstract

A direct asymmetric strategy for fulfilling the functional group transfer by aryl ether cleavage and migration is developed, representing the first Brønsted base‐catalyzed asymmetric [1,3]‐sigmatropic rearrangement reaction. The resulting chiral benzofuran products generated through this strategy exhibit significant 3D‐dimensionality and demonstrate promising antiproliferative properties in biological assays, suggesting potential for medicinal chemistry research.

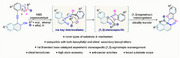

## 1 Introduction

**Figure 1:**
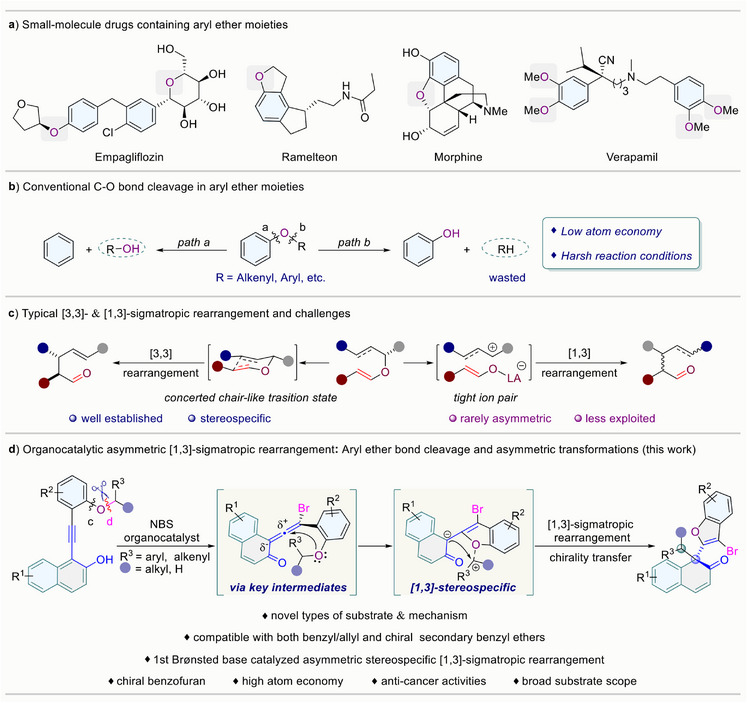
Research background and our new strategy.

The development of atom‐economical procedures for stereoselectively constructing chiral building blocks represents one of the key issues in the field of organic synthesis.^[^ ^1^ ^]^ Great efforts have been made in the past decades and tremendous advances have been achieved to reach this goal. While there are still some crucial fundamental issues that need to be addressed, to overcome the obstacles involving harsh reaction conditions, costly materials, and lengthy synthetic processes. Among them, the selective cleavage of C─O bond by directly editing an aryl ether moiety, is one of the keys to developing important synthetic strategies for the convenient introduction of new functional groups into existing molecular scaffolds.^[^ ^2^ ^]^ Not only because the aryl ether moieties are commonly found in a wide range of biologically important molecules (**Figure** **1**a), but also because the C─O bond often acts as synthetically valuable linkers in organic transformations.^[^ ^3^ ^]^ It is commonly known that the C─O bond can be cleaved under harsh and strongly acidic conditions, or by using strong Lewis acids like BBr_3_ and trimethylsilyl iodide (Figure 1b).^[^ ^4^ ^]^ However, these protocols are not tolerant to many other useful functional groups in the molecules, and more critically, the fragment that comes out from the cleavage cannot be used for downstream transformations,^[^ ^5^ ^]^ and thus could not be considered atom‐economical processes from the viewpoint of sustainable chemistry. Despite some elegant protocols that have been developed through the insertion of low‐covalent transition metal, boron, and carbene into C─O bonds,^[^ ^6^ ^]^ the selective approach for catalytic C─O bond cleavage and asymmetric transformation of the cleaved part into target molecules is still in its infancy.

O‐to‐C rearrangement reactions^[^ ^7^ ^]^ were considered one of the most atom‐ and step‐economical strategies to cleavage the C─O bond and utilize the alkyl groups through intramolecular migration. One notable example is the well‐investigated [3,3]‐sigmatropic rearrangement,^[^ ^8^ ^]^ which has been shown to facilitate the transfer of stereochemistry from a cleaved C─O bond to a resulting C─C bond via a concerted chair‐like transition state and is therefore stereospecific.^[^ ^9^ ^]^ In contrast, the [1,3] O‐to‐C rearrangement has been less extensively investigated, and achieving stereospecific [1,3]‐sigmatropic rearrangement is highly challenging due to the formation of presumed zwitterion pairs (Figure 1c).^[^ ^10^ ^]^ Although several pioneering works on thermal and Lewis acid‐mediated [1,3]‐sigmatropic rearrangements that transmit stereochemical information have been documented,^[^ ^11^ ^]^ the transformation in these specific instances lacks universality and results in a significant decrease in enantiomeric excess.^[^ ^12^ ^]^ Moreover, most examples of highly enantioselective [1,3]‐sigmatropic rearrangement reactions have been achieved through transition metal catalysis,^[^ ^11a–d^ ^]^ with only a very few being catalyzed by organocatalysts.^[^ ^11e^ ^]^ Given the significance of such reactions, the development of a novel organocatalytic asymmetric [1,3]‐sigmatropic rearrangement would be attractive and highly desirable. Nevertheless, the non‐transition metal catalytic enantioselective [1,3]‐sigmatropic rearrangement is highly challenging due to the following reasons. First, the energy barrier for cleaving the C─O bond is generally high and mostly relies on a metal‐involved process. Second, it is difficult to achieve reaction site matching and stereocontrol for organic‐catalyzed alkyl migration. Third, the more favorable [3,3]‐sigmatropic rearrangement is the predominant pathway. Therefore, the identification of a suitable intermediate that bears remarkable reactivity and well‐defined conformational behaviors is the key to this strategy. Based on our previous experiences on vinylidene *ortho*‐quinone methide intermediates (**VQMs**) chemistry,^[^ ^13^ ^]^ and more recently, its high reactivity and stereoselectivity have been demonstrated by enantioselective dearomatization of non‐activated arenes.^[^ ^13i^ ^]^ Thus, we anticipated that **VQMs** intermediates might be used as the key intermediate to selectively cleave the C─O bond (Figure 1d). The stereo‐defined multiple reactive sites of **VQMs** might offer the opportunity to capture the leaving part of bond cleavage in a stereoselective manner at the meta‐position of an oxygen atom and realize an asymmetric [1,3]‐sigmatropic rearrangement reaction.

Herein, we describe an organocatalytic asymmetric [1,3]‐sigmatropic rearrangement reaction involving C─O bond cleavage and enantioselective benzyl/allyl migration process to access various valuable chiral benzofuran derivatives bearing a quaternary carbon stereocenter. To our knowledge, this protocol represents the first example of a Brønsted base‐catalyzed asymmetric [1,3]‐sigmatropic rearrangement reaction. In particular, when a chiral center was previously installed into the aryl ether group, the corresponding alkyl group can migrate into the *meta*‐position of oxygen and the corresponding configuration could remain. In this way, two continuous stereo carbon centers are created in one step. Moreover, the preliminary biological activity investigations indicated that these benzofuran molecules exhibited potential as anticancer agents.^[^ ^14^ ^]^

## 2 Results and Discussion

### 2.1 Reaction Conditions Screening

**Figure 2:**
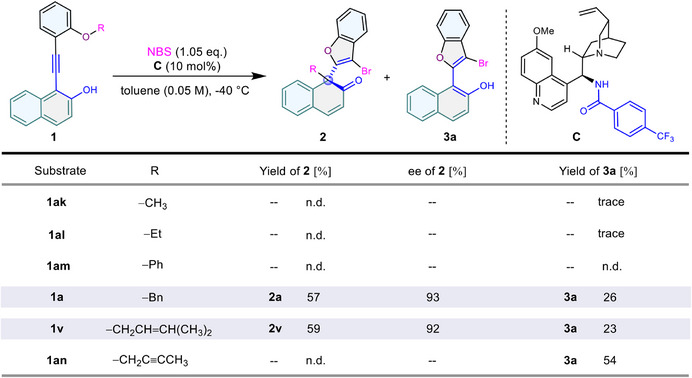
Substituents screening. Reaction conditions: substrate **1** (0.05 mmol) and catalyst **C** (10 mol%) in toluene (1.0 mL) at −40 °C for 15 min, then NBS (1.05 eq.) at −40 °C, 12 h. Isolated yield. Enantiomeric excess (ee) determined by HPLC.

Initially, we investigated the substrates to screen out suitable aryl ethers for the functional group migration process (**Figure** **2**). Naphthoquinones linked with different *ortho*‐ether substituted phenyls (**1a**, **1v**, **1ak**‐**1an**) were prepared and subjected to an empirical reaction condition of NBS and cinchona alkaloid catalyst **C** ^[^ ^15^ ^]^ in toluene at −40 °C for 12 h. Distinct outcomes were acquired depending on the phenyl ether types. For phenyl ethers with methyl or an ethyl group (**1ak** and **1al**), only a trace amount of benzofuran formation (**3a**) without alkane transferring via direct 5‐endo‐dig cyclization were monitored, and no target products were detected for the phenyl‐substituted substrate (**1am**). To our delight, substrates with a benzyl or methylbutene group not only gave rise to the minor product benzofuran (**3a**), but also produced the major asymmetric transformation products (**2a** and **2v**) with excellent enantioselectivity (92–93% ee). Further attempts with the more unsaturated butynyl substituent (**1an**) only outputted the background benzofuran (**3a**) in moderate yield (54%).

**Figure 3:**
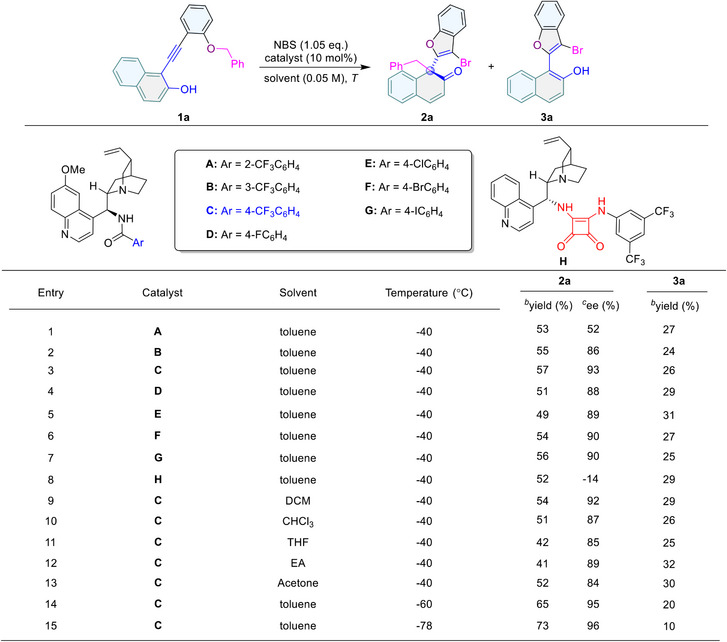
Reaction optimization. *^a^* Reaction conditions: **1a** (0.05 mmol, 1.0 eq.), catalyst (0.005 mmol, 10 mol%) in solvent (1.0 mL) at corresponding temperature for 15 min, then NBS (1.05 eq.) was added at corresponding temperature for 12 h. *^b^* Isolated yield. *^c^* Enantiomeric excess (ee) determined by HPLC.

We then focused on the cinchona alkaloid organocatalysts for condition optimization with **1a** as the template substrate (**Figure** **3**). Albeit similar yields (49–57%) were achieved with all cinchona alkaloids, they led to different enantioselective performances. Catalyst **A** with an *ortho*‐substituted chiral benzoic amide moiety only gave product **2a** with moderate ee (52%).

In contrast, *meta*‐ or *para*‐substituted chiral benzoic amides (**B**‐**G**) catalyzed the reaction with good to excellent enantioselectivity (86–93% ee). Moreover, the squaramide **H** exhibited poor enantioselective control on the reaction (−14% ee). Hence, benzoic amide **C** was chosen as the optimal catalyst. Further, common solvents including dichloromethane (DCM）, chloroform, tetrahydrofuran (THF), ethyl acetate (EA), and acetone were also investigated but failed to improve the yield and enantioselectivity. Reducing the reaction temperature to −60 °C allowed for enhancement in both yield (65%) and ee (95%) of product **2a**, and even lower temperature (−78 °C) further improved the yield of **2a** to 73%, with only a small amount of byproduct **3a** observed (10% yield).

### 2.2 Substrate Scope and Control Experiments

**Figure 4:**
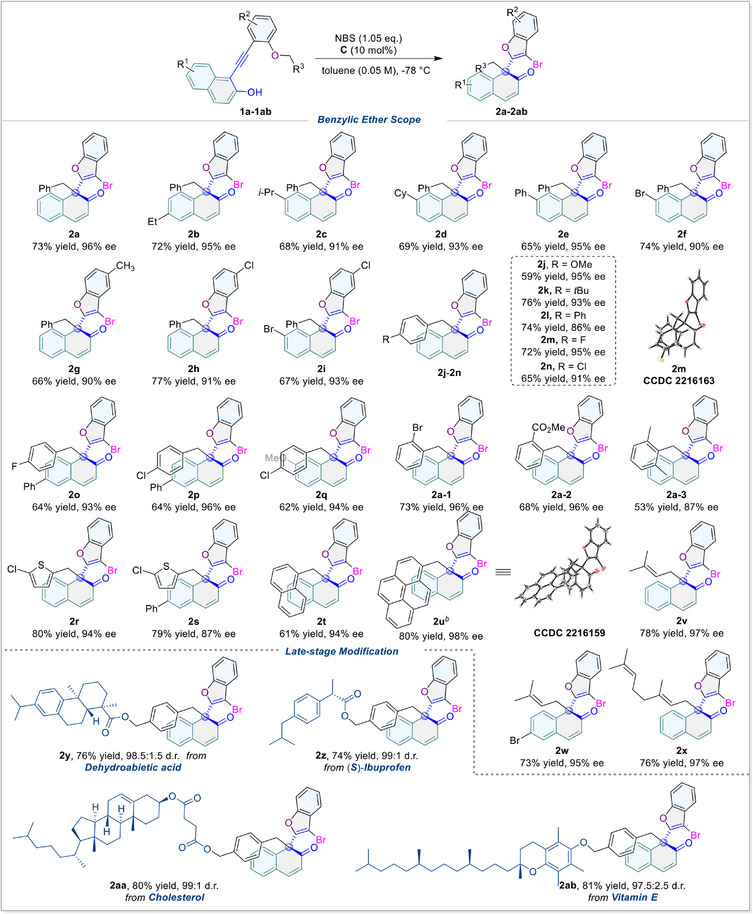
Substrate scope of benzylic ethers: Substrates **1a**‐**1ab** (0.05 mmol) and catalyst **C** (10 mol%) in toluene (1.0 mL) at −78 °C for 15 min, then NBS (1.05 eq.) at −78 °C, 12 h. *^b^* 4.0 mL toluene was used. Isolated yield. Enantiomeric excess (ee) and diastereomeric ratio (d.r.) determined by HPLC.

With the optimal reaction conditions in hand, we then explored the substrate scope of this reaction to illuminate its universality (**Figure** **4**). As the substrates were composed of three sub‐structural components, we first focused on the 2‐naphthol part. It was found that the electron‐donating (Et) alkyl group at the 6‐position of naphthol (**2b**) was well‐tolerated. With regard to the substituents at the 7‐position of 2‐naphthol (**2c**‐**2f**), a slight decrease in yield (65–69%) and ee (91–95%) was observed for electron‐donating (*i*Pr and Cy) and aryl (Ph) groups, whereas the electron‐withdrawing group (Br) maintained the yield (74%) but slightly reduced the enantioselectivity (90% ee). For phenyl groups, both electron‐donating (Me) and electron‐withdrawing group (Cl) at the 4‐position (**2g**‐**2h**) did not cause an obvious loss in yield (66% and 77%) or ee (90% and 91%). Besides, the combination of substituents at the 7‐position of 2‐naphthol and the 4‐position of phenyl (**2i**) retained excellent enantioselectivity (93% ee). We further investigated the ether branch and found that *para*‐substituted benzyls with either electron‐donating (OMe and *t*Bu), aryl (Ph), or electron‐withdrawing groups (F, Cl) (**2j**‐**2n**) were well‐tolerated and achieved satisfying yield (59–76%) as well as good to excellent enantioselectivity (86–95% ee). Combinations of substituents at the 4‐position of benzyl and the 6‐ or 7‐position of 2‐naphthol (**2o**‐**2q**) also retained excellent enantioselectivity (93–96% ee). Additionally, substrates with bromo and ester substituents at the *ortho*‐position of the benzyl group exhibited remarkable reactivity (**2a‐1**, 73% yield, 96% ee; **2a‐2**, 68% yield, 96% ee). However, a different scenario emerged when examining substrate **1a‐3**, which has methyl groups at the 2,6‐positions of the benzyl moiety (**2a‐3**, 53% yield, 87% ee). In addition, other forms of the ether branches, such as methyl heterocycle (**2r**‐**2s**), bulky methyl aromatics (**2t**‐**2u**), and olefins (**2v**‐**2x**), could be smoothly transferred during the reaction (61–80% yield, 87–98% ee). To further elucidate the synthetic value of our approach, we have loaded several bioactive molecules including dehydroabietic acid (**2y**), (*S*)‐ibuprofen (**2z**), cholesterol (**2aa**), and vitamin E (**2ab**) onto the benzyl moiety and fulfilled the transformations of corresponding ethers with nearly perfect stereocontrol. Hence, our strategy may be used to deliver pharmaceuticals or to explore extraordinary pharmacological characteristics. Finally, the absolute configurations of **2m** and **2u** were detected by single‐crystal X‐ray crystallographic analysis and others were assigned to analog.

**Figure 5:**
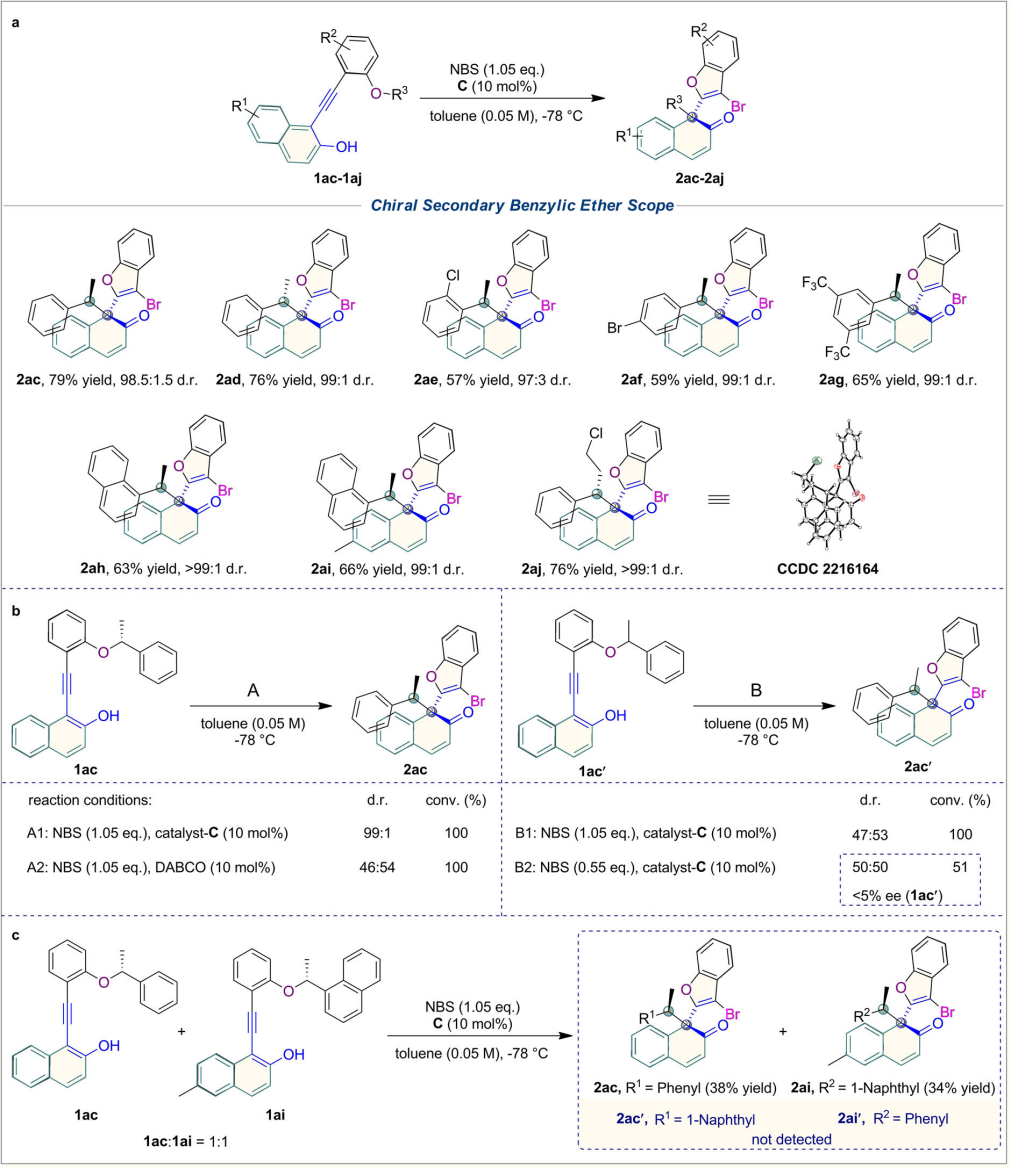
Substrate scope of chiral secondary benzylic ethers and control experiments: a) Substrates **1ac**‐**1aj** (0.05 mmol) and catalyst **C** (10 mol%) in toluene (1.0 mL) at −78 °C for 15 min, then NBS (1.05 eq.) at −78 °C, 12 h. Isolated yield. The diastereomeric (d.r.) ratio is determined by HPLC. b) Substrate **1ac** or **1ac′** (0.05 mmol) and catalyst **C** or DABCO (10 mol%) in toluene (1.0 mL) at −78 °C for 15 min, then NBS (1.05 or 0.55 eq.) at −78 °C, 12 h. Enantiomeric excess (ee) and diastereomeric ratio (d.r.) determined by HPLC. c) Crossover experiments.

In addition, we extended the application scope of this strategy with chiral secondary benzylic ether substrates (**Figure** **5**a). The results turned out that various ether blocks were compatible with this reaction (**2ac**‐**2aj**). Especially, the original stereochirality of the tertiary carbon center was well transferred into the product (up to >99:1 d.r.) in each case. To be specific, both (*R*)‐ and (*S*)‐1‐ phenylethyl moieties could be stereoselectively installed into thenaphthalen‐2(1*H*)‐one scaffold with high yields and excellent diastereoselectivities (**2ac** and **2ad**). Halogens in the *ortho*‐ (**2ae**) or *para*‐position (**2af**) of 1‐phenylethyl somehow decreased the yield (57% and 59%), yet obtained excellent diastereoselectivities (97:3 d.r. and 99:1 d.r.). Furthermore, trifluoromethyl groups at both *meta*‐positions of 1‐phenylethyl (**2ag**) elevated the diastereoselectivity to a perfect level (99:1 d.r.). Replacing 1‐phenylethyl with 1‐naphthalenylethyl, with or without modification on the 2‐naphthol, also gave products with perfect diastereoselectivities (**2ah** and **2ai**). Moreover, a stereocenter with a linear alkyl group also contributed to the perfect stereoselectivity (**2aj**, >99:1 d.r.).

To demonstrate the manipulation of stereoselectivity in asymmetric catalysis reactions, several control experiments were conducted (Figure 5b). Under the standard conditions (1.05 eq. NBS, 10 mol% catalyst **C,** and 0.05 m toluene), chiral organocatalyst **C** carried out the reaction with nearly perfect diastereoselectivity (99:1 d.r.). In contrast, achiral DABCO exhibited very poor diastereoselectivity in completing the conversion of **1ac** to **2ac** (46:54 d.r.). A racemic substrate **1ac′** was also synthesized, and when subjected to the same conditions as catalyst **C**, the product was obtained with similarly low diastereoselectivity (47:53 d.r.). Besides, reducing the amount of NBS gave the product with a conversion of 51% and poor stereoselectivity. Overall, the results of the control experiment suggested that the cinchona alkaloid catalyst effectively controlled the formation of the chiral **VQM** intermediate and its chirality transfer. Furthermore, through theoretical calculations, we have identified the ion pair intermediate involved in the rearrangement reaction (Figure S12, Supporting Information). To gain insight into the reaction mechanism and validate the theoretical calculations, a crossover reaction was carried out using substrates **1ac** and **1ai**. As shown in Figure 5c, products **2ac** and **2ai** were obtained cleanly while no crossover products were detected (**2ac**, 38% yield; **2ai**, 34% yield). This indicates that the rearrangement is an intramolecular reaction, which should involve a tight ion pair intermediate.^[^ ^11a^ ^]^ This experimental result also validates the theoretical calculations. Finally, to have a clearer understanding of the rearrangement mechanism, a plausible reaction mechanism has been proposed based on the above studies and investigated **VQM**s‐based reactions (Figure S11, Supporting Information).

### 2.3 Biological Activities Tests

**Figure 6:**
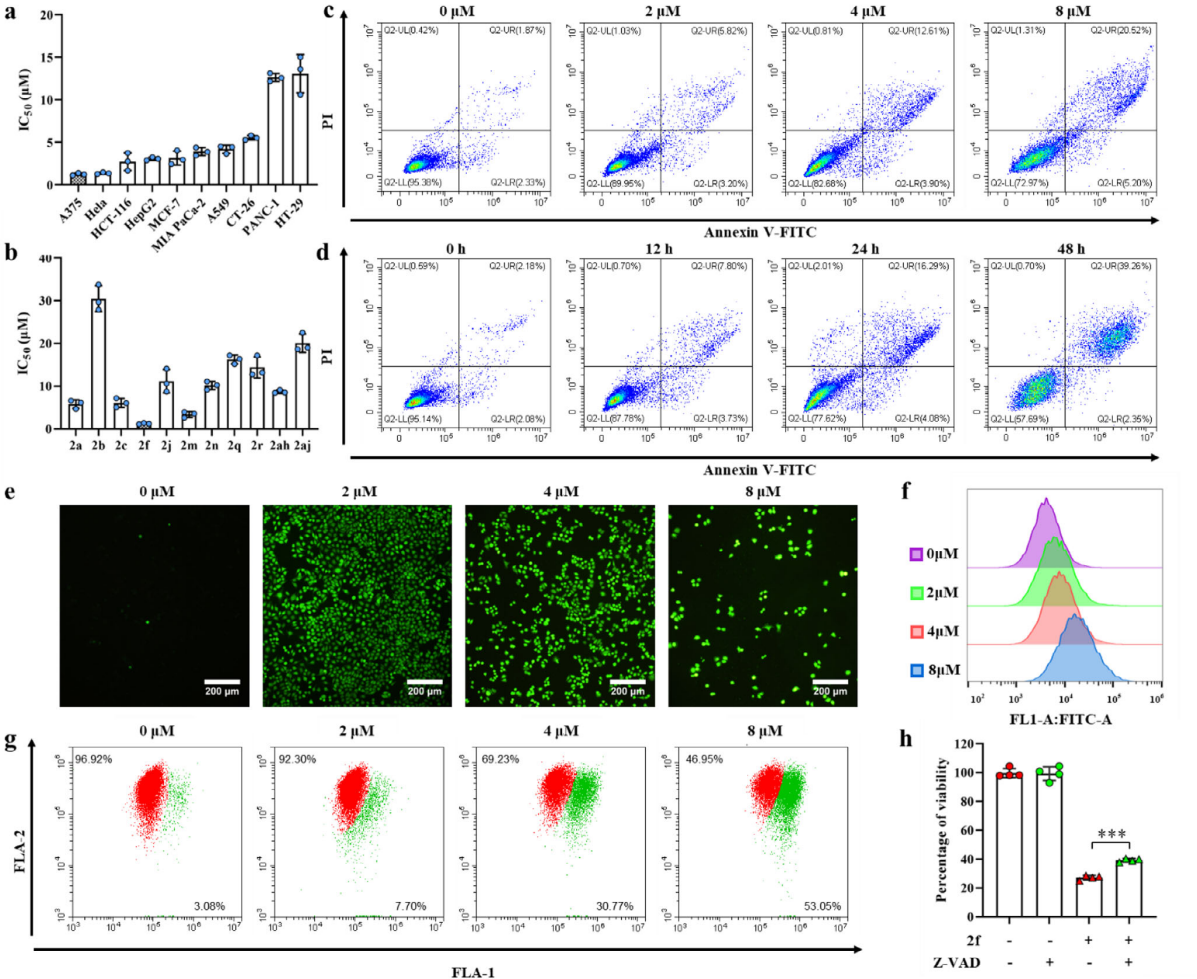
Pharmacological profile of **2f**. a) The IC_50_ values of **2f** toward a panel of cancer cell lines. b) The IC_50_ by **2f** (0, 2, 4, and 8 µM) for 24 h. c) A375 cells apoptosis induced by **2f** (0, 2, 4, and 8 µmM) for 24 h. d) A375 cells apoptosis induced by **2f** (0, 12, 24, and 48 h) at 4 µM. e) Fluorescent images of the intracellular ROS generation in A375 cells after incubation with **2f** (0, 2, 4, and 8 µM) for 24 h. f) Quantitative flow cytometry analysis of ROS level in A375 cells after incubation with **2f** (0, 2, 4, and 8 µM) for 24 h. g) MMP loss in A375 cells after incubation with **2f** (0, 2, 4, and 8 µM) for 24 h. h) The percentage of cell viability after the incubation with **2f** (2 µM) and cell apoptosis inhibitor Z‐VAD‐FMK (10 µM) for 72 h. Analysis results represented mean ± SD, ^***^ *p*<0.001.

We further screened and explored the potential biological activities of the obtained chiral benzofuran derivatives. Cell viability assays showed that some chiral benzofuran compounds with certain substituents had antiproliferation activity toward a variety of cancer cells at the concentration of 20 µM (Figures S4–S8, Supporting Information). Among them, compound **2f** with a quaternary carbon stereocenter showed the better inhibitory effects (**Figure** **6**a), and exhibited the best anti‐cancer activity against A375 cells, with an IC_50_ of ≈1 µM (Figure 6b). In addition, FACS assays with Annexin V and PI staining demonstrated that the apoptosis percentages of A375 cells treated with **2f** were in a dose‐ and time‐dependent manner, and higher concentration and longer incubation time led to more apoptosis (Figure 6c,d). To further explore the apoptosis mechanism of **2f**, intracellular ROS (reactive oxygen species) level and mitochondrial membrane potential (MMP) loss, hallmarks of mitochondrial dysfunction, were evaluated. As shown in Figure 6e–g, ROS and MMP depolarization levels of A375 cells increased significantly with the increasing concentration of **2f**, indicating that **2f** can cause mitochondrial‐dependent apoptosis. Moreover, the induction of apoptosis in A375 cells by **2f** was inhibited in the presence of apoptosis inhibitor Z‐VAD‐FMK (Figure 6h), while necrosis inhibitor necrostatin‐1 had no effect (Figure S9, Supporting Information). These results collectively demonstrated that **2f** exerted anti‐cancer activities mainly through apoptosis, and its unique chemical structure may provide more possibilities for developing new anticancer drugs.

## 3 Conclusion

In conclusion, we have developed a direct asymmetric strategy to fulfill the functional group transfer by aryl ether cleavage and migration, which represents the first Brønsted base‐catalyzed asymmetric [1,3]‐sigmatropic rearrangement reaction. A wide range of aryl ether substrate scope has been evaluated and proved to be quite compatible with this reaction process. Of interest, the original stereochirality of the ether branch was well maintained and transferred into the new site. As these products possessed high 3D‐dimensionality and scaffold complexity, we performed a set of biological assays on these new chemicals. Several molecules exhibited interesting antiproliferative properties and this asymmetric catalytic approach may be employed in future medicinal chemistry studies.

## 4 Experimental Section

### General Procedure for the Synthesis of Chiral Benzofuran **2a**‐**2aj**

Substrates **1a**‐**1aj** (0.05 mmol) and catalyst **C** (10 mol%) in toluene (1.0 mL) at −78 °C for 15 min, then NBS (1.05 eq.) was added at −78 °C. The resulting reaction mixture was stirred for 12 h under these conditions. When the reaction was completed (monitored by TLC), the mixture was purified by flash column chromatography on silica gel (petroleum ether/ethyl acetate = 40:1 to 25:1) to afford the pure products **2a**‐**2aj** in good to high yields.

[CCDC 2216163 (for **2m**), 2216159 (for **2u**) and 2216164 (for **2aj**) contains the supplementary crystallographic data for this paper. These data can be obtained free of charge from The Cambridge Crystallographic Data Centre via www.ccdc.cam.ac.uk/data_request/cif.

### Materials

Unless otherwise specified, the reagents and materials were obtained from commercial sources and used as received. Organic chemicals, including toluene, dichloromethane (DCM), chloroform (CHCl_3_), tetrahydrofuran (THF), ethyl acetate (EA), acetone, methanol (MeOH), acetonitrile (ACN), *N*,*N*‐dimethylformamide (DMF), petroleum ether (PE), anhydrous ethanol (EtOH), and other chemicals, e.g., potassium carbonate (K_2_CO_3_), and triphenylphosphine (PPh_3_), were purchased from commercial suppliers (Adamas, J&K, Sigma–Aldrich, TCI). All the reagents were analytical and chromatographic grade.

### Characterizations

^1^H and ^13^C NMR spectra were recorded on Agilent 400MR DD2 (400 MHz) and 600MR DD2 (600 MHz) spectrometer. Chemical shifts were reported in parts per million (ppm), and tetramethylsilane or the residual solvent peak was used as an internal reference: CDCl_3_ (^1^H NMR tetramethylsilane δ 0.00, ^1^H NMR δ 7.25, ^13^C NMR δ 77.00), data are reported as follows: chemical shift, multiplicity (s = singlet, d = doublet, t = triplet, q = quartet, m = multiplet, br = broad), coupling constants (Hz) and integration. Enantiomeric excesses (ee) were determined by HPLC analysis on Hitachi Chromaster using DAICEL CHIRALCEL IA‐H, 4.6 mm*Ф*×250 mm, DAICEL CHIRALCEL IB‐H, 4.6 mm*Ф*×250 mm, DAICEL CHIRALCEL IC‐H, 4.6 mm*Ф*×250 mm. High‐resolution mass spectra (HRMS) were performed on Bruker Solarix 7.0 T. X‐ray crystallography analysis of a single crystal was performed on an Agilent SuperNova‐CCD X‐Ray diffractometer. Optical rotations were measured on a Rudolph Autopol I polarimeter and are reported as follows: ${\left[\alpha \right]}_{D}^{25}$ (*c* in g per 100 mL solvent).

## Conflict of Interest

The authors declare no conflict of interest.

## Supporting information



Supporting Information

Supporting Information

## Data Availability

The data that support the findings of this study are available in the supplementary material of this article.
