# Supplementary material for: Organocatalytic C─O Bond Cleavage and Asymmetric Transformations via [1,3]‐Sigmatropic Rearrangement
Source: Adv Sci (Weinh). 2025 May 19;12(29):70006. doi: 10.1002/advs.202504718 (PMC12362732; doi:10.1002/advs.202504718)
Supplement: Supplementary file 1 — Supporting Information [file ADVS-12-70006-s002.pdf]

ADVANCED SCIENCE
Open Access

Supporting Information

for *Adv. Sci.*, DOI 10.1002/advs.202504718

Organocatalytic C—O Bond Cleavage and Asymmetric Transformations via [1,3]-Sigmatropic Rearrangement

Lei Peng, Yu Chang, Liangchen Yin, Jinbang Zhang, Xuli Feng, Pengfei Wang*, Wenling Qin* and Hailong Yan**

Supporting Information

Organocatalytic C−O Bond Cleavage and Asymmetric Transformations *via* [1,3]-Sigmatropic Rearrangement

Lei Peng,*[ab] Yu Chang,[b] Liangchen Yin,[b] Jinbang Zhang,[b] Xuli Feng,[b] Pengfei Wang,*[b] Wenling Qin,*[b] and Hailong Yan*[ab]

[a]Chongqing University FuLing Hospital, No.2 Gaosuntang Road, Fuling District, Chongqing 408000, P. R. China.

[b]Chongqing Key Laboratory of Natural Product Synthesis and Drug Research, School of Pharmaceutical Sciences, Chongqing University, Chongqing 401331, P. R. China.

*Corresponding author. Emails: pl19940415@163.com; wangpf@cqu.edu.cn; wenling.qin@cqu.edu.cn; yhl198151@cqu.edu.cn

Table of Contents

Materials and Methods 2
General procedure for the synthesis of substrates 2
Optimization of the reaction condition 7
General procedure for the preparation of racemic **2a**-**2ab** 8
General procedure for the preparation of racemic **2ac**-**2aj** 8
General procedure for the asymmetric reaction of **1a**-**1ab** 8
General procedure for the asymmetric reaction of **1ac**-**1aj** 8
^{1}H, ^{13}C NMR and HRMS data of compounds (**1a**-**1an**) 9
^{1}H, ^{13}C NMR and HRMS data of compounds (**2a**-**2aj**, **3a**) 30
Control experiments 61
Crossover experiments 63
Electrophilic reagent screening 63
^{1}H and ^{13}C NMR spectra 64
X-ray crystallographic information 148
Biological evaluation 154
Plausible catalytic cycle 159
Theoretical calculations 160
References 163

Materials and Methods

^{1}H and ^{13}C NMR spectra were recorded on Agilent 400MR DD2 (400 MHz) spectrometer. Chemical shifts were reported in parts per million (ppm), and tetramethylsilane or the residual solvent peak was used as an internal reference: $CDCl_3$ (^{1}H NMR tetramethylsilane δ 0.00, ^{1}H NMR δ 7.25, ^{13}C NMR δ 77.00), data are reported as follows: chemical shift, multiplicity (s = singlet, d = doublet, t = triplet, q = quartet, m = multiplet, br = broad), coupling constants (Hz) and integration. Enantiomeric excesses (ee) were determined by HPLC analysis on Hitachi Chromaster using DAICEL CHIRALCEL IA-H, 4.6mm*Φ*×250mm, DAICEL CHIRALCEL IB-H, 4.6mm*Φ*×250mm, DAICEL CHIRALCEL IC-H, 4.6mm*Φ*×250mm. High resolution mass spectra (HRMS) were performed on Bruker Solarix 7.0 T. X-ray crystallography analysis of single crystal was performed on an Agilent SuperNova-CCD X-Ray diffractometer. Optical rotations were measured on a Rudolph Autopol I polarimeter and are reported as follows: $[\alpha]_D^{25}$ (*c* in g per 100 mL solvent). Unless otherwise stated, all reagents were purchased from commercial suppliers (Adamas, J&K, Sigma-Aldrich, TCI) and used without further purification.

General procedure for the synthesis of substrates

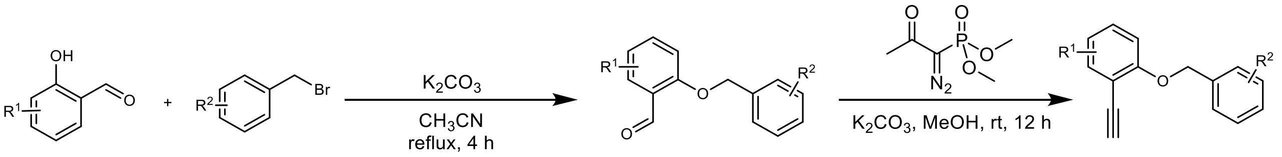

S1 **S2**

General procedure for the synthesis of **S1**:

This step was carried out according to a literature method[1] with some modifications. A suspension of K_2CO_3 (60.0 mmol, 3 equiv.) in acetonitrile (100 mL) was added the substituted salicylaldehyde (20.0 mmol, 1.0 equiv.) and substituted benzyl bromide (24.0 mmol, 1.2 equiv.). The reaction was stirred at 80°C under nitrogen atmosphere for 4 hours. After completion, the reaction mixture was poured into brine (80 mL) and extracted with EA (3 × 60 mL). The organic layer was dried over Na_2SO_4 and concentrated under reduced pressure. The crude product was purified by column chromatography on silica gel (PE/EA = 30:1) to afford **S1** (56-89% yield).

General procedure for the synthesis of **S2:**

This step was carried out according to a literature method[2] with some modifications. A mixture of **S1** (12.0 mmol, 1.0 equiv.), K_2CO_3 (24.0 mmol, 2.0 equiv.) in MeOH (30 mL) was added dimethyl (1-diazo-2-oxopropyl)phosphonate (14.4 mmol, 1.2 equiv.) at room temperature. The reaction mixture was stirred at room temperature for 12 hours. After completion, the reaction mixture was poured into brine (30 mL) and extracted with EA (3 × 30 mL). The organic layer was dried over Na_2SO_4 and concentrated under reduced pressure. The crude product was purified by column chromatography on silica gel (PE/EA = 40:1) to afford **S2** (65-87% yield).

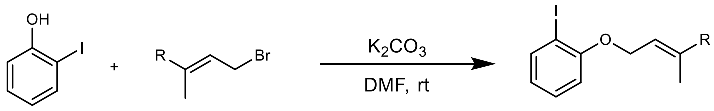

S3 **S4**

This step was carried out according to a literature method[3] with some modifications. A mixture of 2-Iodophenol (12.0 mmol, 1.0 equiv.), K_2CO_3 (24.0 mmol, 2.0 equiv.) in DMF (40 mL) was added **S3** (14.4 mmol, 1.2 equiv.) at room temperature. The reaction mixture was stirred at room temperature for 8 hours. After completion, the reaction mixture was poured into brine (30 mL) and extracted with EA (4 × 30 mL). The organic layer was dried over Na_2SO_4 and concentrated under reduced pressure. The crude product was purified by column chromatography on silica gel (PE/EA = 30:1) to afford **S4** as a yellow oil (62-77% yield).

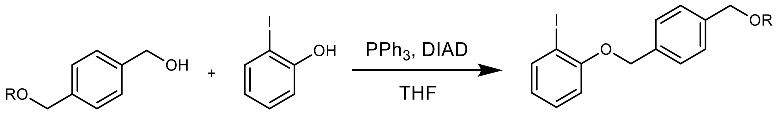

S5 **S6**

General procedure for the synthesis of **S6**:

This step was carried out according to a literature method[4] with some modifications. To a solution of **S5** (24.0 mmol, 1.0 equiv.), 2-Iodophenol (24.0 mmol, 1.0 equiv.), Ph_3P (24.0 mmol, 1.0 equiv.) in THF (40 mL) was added DIAD (24.0 mmol, 1.0 equiv.) slowly at 0°C, and the mixture was stirred for 8 hours at room temperature. After completion, the mixture was concentrated under reduced pressure to leave a residue. The crude product was purified by column chromatography on silica gel (PE/EA = 15:1-30:1) to afford **S6** (67-84% yield).

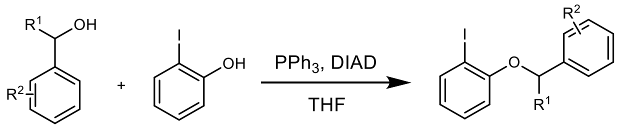

S7 **S8**

General procedure for the synthesis of **S8**:

This step was carried out according to a literature method[4] with some modifications. To a solution of chiral **S7** (36.0 mmol, 1.0 equiv.), 2-Iodophenol (36.0 mmol, 1.0 equiv.), Ph_3P (36.0 mmol, 1.0 equiv.) in THF (60 mL) was added DIAD (36.0 mmol, 1.0 equiv.) slowly at 0°C, and the mixture was stirred for 8 hours at room temperature. After completion, the mixture was concentrated under reduced pressure to leave a residue. The crude product was purified by column chromatography on silica gel (PE/EA = 30:1) to afford **S8** (54-72% yield).

Method A: (**1a-1u**)

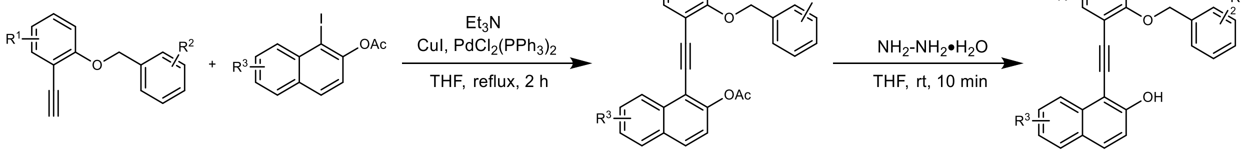

S2 **S9** **S10** **S11**

General procedure for the synthesis of **S10**:

$PdCl_2(PPh_3)_2$ (0.16 mmol, 0.08 equiv.), CuI (0.32 mmol, 0.16 equiv.) and **S9** (2.0 mmol, 1.0 equiv.) were weighed and added into an oven dried flask, evacuated and backfilled with nitrogen (3 times). THF (10.0 mL) and Et_3N (10 mmol, 5.0 equiv.) were injected into the flask. Then, the mixture was stirred for 30 min at 60 °C. After that, the **S2** (2.4 mmol, 1.2 equiv.) dissolved in THF (5.0 mL) was added slowly. The resulting mixture kept stirring for 2 hours at 60 °C. Then, the mixture was filtered through a pad of celite. Removal of the solvent under reduced pressure afforded a residue which is purified by chromatography on silica gel (PE/EA = 20:1) to afford **S10** (49-76% yield).

General procedure for the synthesis of **S11**:

Hydrazine monohydrate (5.0 mmol, 5.0 equiv.) was dropwise added to a solution of **S10** (1.0 mmol, 1.0 equiv.) in THF (10.0 mL). After the resulted mixture was stirred at room temperature for 10 minutes, the mixture was treated with sat. aq. NH_4Cl and extracted with ethyl acetate three times, and dried over Na_2SO_4. Removal of the solvent under reduced pressure afforded a residue which is purified by chromatography on silica gel (PE/EA = 20:1) to afford **S11** (78-91% yield).

Method B: (**1v-1x**)

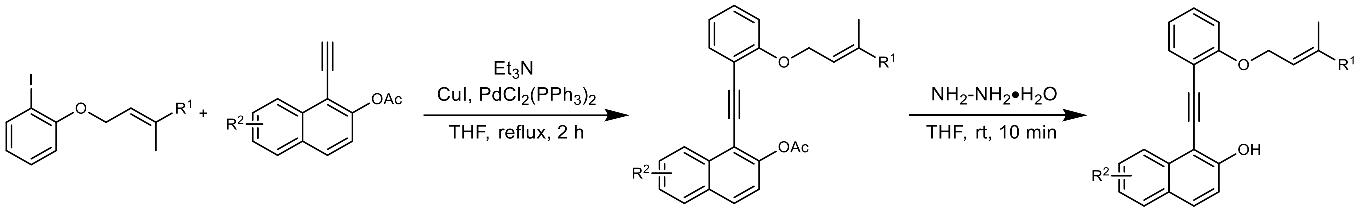

General procedure for the synthesis of **S13**:

$PdCl_2(PPh_3)_2$ (0.16 mmol, 0.08 equiv.), CuI (0.32 mmol, 0.16 equiv.) and **S4** (2.0 mmol, 1.0 equiv.) were weighed and added into an oven dried flask, evacuated and backfilled with nitrogen (3 times). THF (10.0 mL) and Et_3N (10 mmol, 5.0 equiv.) were injected into the flask. Then, the mixture was stirred for 30 min at 60 °C. After that, the **S12** (2.4 mmol, 1.2 equiv.) dissolved in THF (5.0 mL) was added slowly. The resulting mixture kept stirring for 2 hours at 60 °C. Then, the mixture was filtered through a pad of celite. Removal of the solvent under reduced pressure afforded a residue which is purified by chromatography on silica gel (PE/EA = 25:1) to afford **S13** (63-79% yield).

General procedure for the synthesis of **S14**:

Hydrazine monohydrate (5.0 mmol, 5.0 equiv.) was dropwise added to a solution of **S13** (1.0 mmol, 1.0 equiv.) in THF (10.0 mL). After the resulted mixture was stirred at room temperature for 10 minutes, the mixture was treated with sat. aq. NH_4Cl and extracted with ethyl acetate three times, and dried over Na_2SO_4. Removal of the solvent under reduced pressure afforded a residue which is purified by chromatography on silica gel (PE/EA = 30:1) to afford **S14** (83-94% yield).

Method C: (**1y-1ab**)

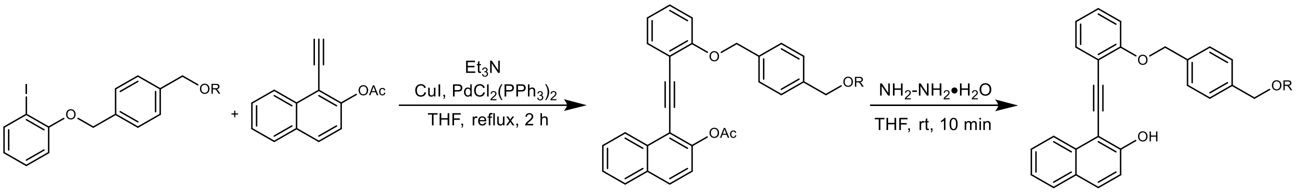

S6 **S12** **S15** **S16**

General procedure for the synthesis of **S15**:

$PdCl_2(PPh_3)_2$ (0.16 mmol, 0.08 equiv.), CuI (0.32 mmol, 0.16 equiv.) and **S6** (2.0 mmol, 1.0 equiv.) were weighed and added into an oven dried flask, evacuated and backfilled with nitrogen (3 times). THF (15.0 mL) and Et_3N (10 mmol, 5.0 equiv.) were injected into the flask. Then, the mixture was stirred for 30 min at 60 ^oC. After that, the **S12** (2.4 mmol, 1.2 equiv.) dissolved in THF (5.0 mL) was added slowly. The resulting mixture kept stirring for 2 hours at 60 ^oC. Then the mixture was filtered through a pad of celite. Removal of the solvent under reduced pressure afforded a residue which is purified by chromatography on silica gel (PE/EA = 15:1) to afford **S15** (58-71% yield).

General procedure for the synthesis of **S16**:

Hydrazine monohydrate (5.0 mmol, 5.0 equiv.) was dropwise added to a solution of **S15** (1.0 mmol, 1.0 equiv.) in THF (10.0 mL). After the resulted mixture was stirred at room temperature for 10 minutes, the mixture was treated with sat. aq. NH_4Cl and extracted with ethyl acetate three times, and dried over Na_2SO_4. Removal of the solvent under reduced pressure afforded a residue which is purified by chromatography on silica gel (PE/EA = 10:1) to afford the compound **S16** as a solid (79-92% yield).

Method D: (**1ac-1aj**)

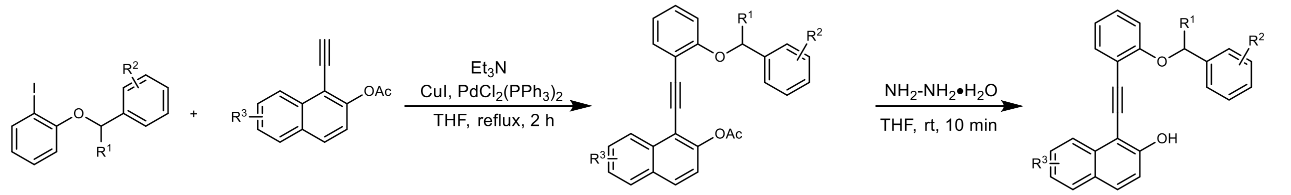

S8 **S12** **S17** **S18**

General procedure for the synthesis of **S17**:

$PdCl_2(PPh_3)_2$ (0.16 mmol, 0.08 equiv.), CuI (0.32 mmol, 0.16 equiv.) and chiral **S8** (2.0 mmol, 1.0 equiv.) were weighed and added into an oven dried flask, evacuated and backfilled with nitrogen (3 times). THF (15.0 mL) and Et_3N (10 mmol, 5.0 equiv.) were injected into the flask. Then, the mixture was stirred for 30 min at 60 ^oC. After that, the **S12** (2.4 mmol, 1.2 equiv.) dissolved in THF (5.0 mL) was added slowly. The resulting mixture kept stirring for 2 hours at 60 ^oC. Then the mixture was filtered through a pad of celite. Removal of the solvent under reduced pressure afforded a residue which is purified by chromatography on silica gel (PE/EA = 15:1) to afford **S17** as a solid (69-83% yield).

General procedure for the synthesis of **S18**:

Hydrazine monohydrate (5.0 mmol, 5.0 equiv.) was dropwise added to a solution of **S17** (1.0 mmol, 1.0 equiv.) in THF (10.0 mL). After the resulted mixture was stirred at room temperature for 10

minutes, the mixture was treated with sat. aq. NH_4Cl and extracted with ethyl acetate three times, and dried over Na_2SO_4. Removal of the solvent under reduced pressure afforded a residue which is purified by chromatography on silica gel (PE/EA = 20:1) to afford the compound **S18** as a solid (76-89% yield).

Method E: (**1ak-1an**)

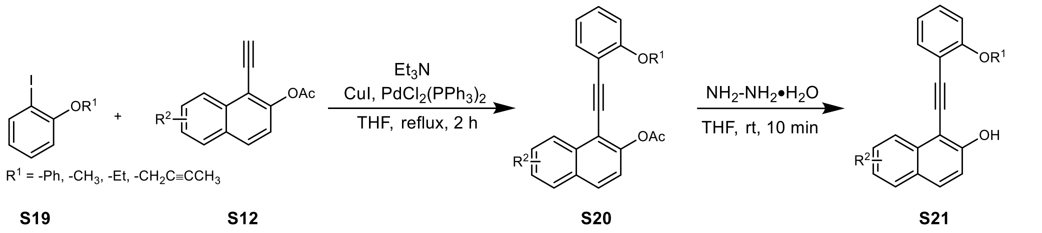

<u>General procedure for the synthesis of **S20**:</u>

$PdCl_2(PPh_3)_2$ (0.16 mmol, 0.08 equiv.), CuI (0.32 mmol, 0.16 equiv.) and **S19** (2.0 mmol, 1.0 equiv.) were weighed and added into an oven dried flask, evacuated and backfilled with nitrogen (3 times). THF (15.0 mL) and Et_3N (10 mmol, 5.0 equiv.) were injected into the flask. Then, the mixture was stirred for 30 min at 60 ^oC. After that, the **S12** (2.4 mmol, 1.2 equiv.) dissolved in THF (5.0 mL) was added slowly. The resulting mixture kept stirring for 2 hours at 60 ^oC. Then the mixture was filtered through a pad of celite. Removal of the solvent under reduced pressure afforded a residue which is purified by chromatography on silica gel (PE/EA = 15:1) to afford **S20** as a solid (64-81% yield).

<u>General procedure for the synthesis of **S21**:</u>

Hydrazine monohydrate (5.0 mmol, 5.0 equiv.) was dropwise added to a solution of **S20** (1.0 mmol, 1.0 equiv.) in THF (10.0 mL). After the resulted mixture was stirred at room temperature for 10 minutes, the mixture was treated with sat. aq. NH_4Cl and extracted with ethyl acetate three times, and dried over Na_2SO_4. Removal of the solvent under reduced pressure afforded a residue which is purified by chromatography on silica gel (PE/EA = 20:1) to afford the compound **S21** as a solid (72-88% yield).

Optimization of the reaction condition

Table S1. Reaction condition optimization[a]

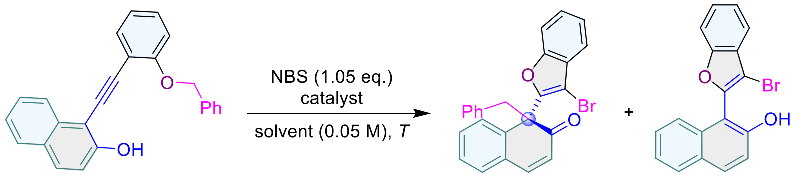

1a 2a 3a

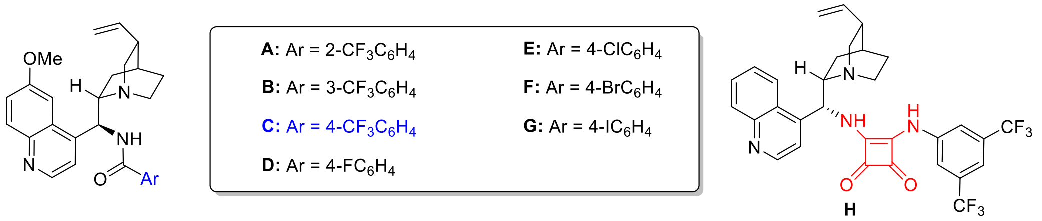

Entry	Catalyst	Loading (mol%)	Solvent	Temperature (°C)	2a		3a
					[b]yield (%)	[c]ee (%)	[b]yield (%)
1	**A**	10	toluene	-40	53	52	27
2	**B**	10	toluene	-40	55	86	24
3	**C**	10	toluene	-40	57	93	26
4	**D**	10	toluene	-40	51	88	29
5	**E**	10	toluene	-40	49	89	31
6	**F**	10	toluene	-40	54	90	27
7	**G**	10	toluene	-40	56	90	25
8	**H**	10	toluene	-40	52	-14	29
9	**C**	10	DCM	-40	54	92	29
10	**C**	10	$CHCl_3$	-40	51	87	26
11	**C**	10	THF	-40	42	85	25
12	**C**	10	EA	-40	41	89	32
13	**C**	10	Acetone	-40	52	84	30
14	**C**	10	toluene	-60	65	95	20
15	**C**	10	toluene	-78	73	96	10

[a]Reaction conditions: **1a** (0.05 mmol, 1.0 equiv.), catalyst (0.005 mmol, 10 mol%) in solvent (1.0 mL) at corresponding temperature for 15 min. then NBS (0.053 mmol, 1.05 equiv.) was added at corresponding temperature for 12 h. [b]Isolated yield. [c]Enantiomeric excess (ee) determined by HPLC.

General procedure for the preparation of racemic 2a-2ab

A solution of **1a-1ab** (0.05 mmol, 1.0 equiv.) in toluene (1.0 mL) was stirred at -40 °C for 15 min, then NBS (1.05 equiv.) was added. The reaction mixture was stirred at -40 °C and monitored by TLC. After completion of the reaction, the mixture was purified by preparative TLC on silica gel to yield the target molecular racemic compounds.

General procedure for the preparation of racemic 2ac-2aj

A solution of *rac*-**1ac-1aj** (0.05 mmol, 1.0 equiv.) in toluene (1.0 mL) was stirred at -40 °C for 15 min, then NBS (1.05 equiv.) was added. The reaction mixture was stirred at -40 °C and monitored by TLC. After completion of the reaction, the mixture was purified by preparative TLC on silica gel to yield the target molecular racemic compounds.

General procedure for the asymmetric reaction of 1a-1ab

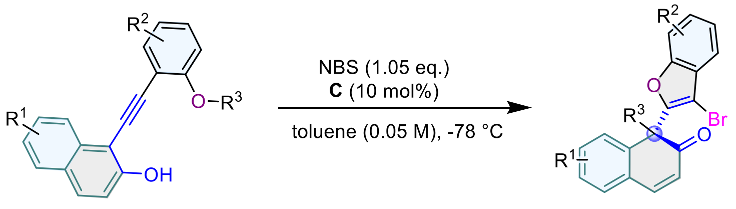

1a-1ab **2a-2ab**

Condition [**A**]: A solution of **1a-1ab** (0.05 mmol, 1.0 equiv.) and catalyst **C** (10 mol%) in toluene (1.0 mL) was stirred at -78 °C for 15 min, then NBS (1.05 equiv.) was added. After stirring at -78 °C for 12 h, the mixture was subjected to silica gel flash column chromatography using PE/EA eluent (40:1 to 25:1) to afford the product **2a-2ab**.

General procedure for the asymmetric reaction of 1ac-1aj

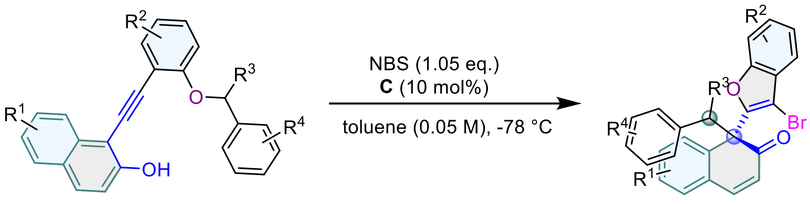

1ac-1aj **2ac-2aj**

Condition [**B**]: A solution of chiral-**1ac-1aj** (0.05 mmol, 1.0 equiv.) and catalyst **C** (10 mol%) in toluene (1.0 mL) was stirred at -78 °C for 15 min, then NBS (1.05 equiv.) was added. After stirring at -78 °C for 12 h, the reaction mixture was subjected to silica gel flash column chromatography using PE/EA eluent (40:1 to 25:1) to afford the product **2ac-2aj**.

^{1}H, ^{13}C NMR and HRMS data of compounds (1a-1an)

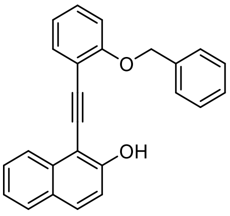

1a

1-((2-(benzyloxy)phenyl)ethynyl)naphthalen-2-ol (1a)

Compound **1a** is an unknown compound, and was synthesized in 62% yield (2 steps, 4 mmol, 869 mg) following the general procedure (**Method A**).

Brown solid. (R_f = 0.5, PE/EA= 10:1)

^{1}H NMR (400 MHz, $CDCl_3$) δ 8.18 (d, *J* = 8.3 Hz, 1H), 7.74 (t, *J* = 9.4 Hz, 2H), 7.59 (d, *J* = 7.5 Hz, 1H), 7.49 (d, *J* = 7.4 Hz, 2H), 7.45 (d, *J* = 8.0 Hz, 1H), 7.42 – 7.32 (m, 4H), 7.29 (t, 1H), 7.18 (d, *J* = 8.9 Hz, 1H), 7.00 (t, *J* = 8.0 Hz, 2H), 6.80 (s, 1H), 5.27 (s, 2H).

^{13}C NMR (100 MHz, $CDCl_3$) δ 158.49, 156.50, 136.22, 132.98, 131.97, 130.35, 129.76, 128.72, 128.25, 128.18, 128.13, 127.49, 127.20, 125.03, 123.85, 120.96, 116.41, 112.67, 112.02, 103.23, 97.84, 87.24, 70.60.

HRMS (ESI) *m/z* Calcd for $C_{25}H_{18}NaO_2^+$ $[M + Na]^+$: 373.1199, Found: 373.1197.

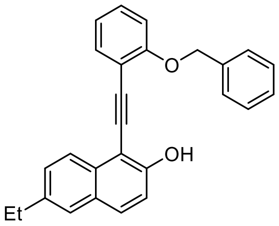

1b

1-((2-(benzyloxy)phenyl)ethynyl)-6-ethylnaphthalen-2-ol (1b)

Compound **1b** is an unknown compound, and was synthesized in 57% yield (2 steps, 4 mmol, 863 mg) following the general procedure (**Method A**).

Yellow solid. (R_f = 0.5, PE/EA= 10:1)

^{1}H NMR (400 MHz, $CDCl_3$) δ 8.10 (d, *J* = 8.5 Hz, 1H), 7.66 (d, *J* = 8.9 Hz, 1H), 7.58 (d, *J* = 7.5 Hz, 1H), 7.53 (s, 1H), 7.48 (d, *J* = 7.3 Hz, 2H), 7.38 (t, *J* = 7.2 Hz, 2H), 7.35 – 7.24 (m, 3H), 7.14 (d, *J* = 8.9 Hz, 1H), 7.03 – 6.94 (m, 2H), 6.72 (s, 1H), 5.25 (s, 2H), 2.77 (q, *J* = 7.5 Hz, 2H), 1.30 (t, *J* = 7.6 Hz, 3H).

^{13}C NMR (100 MHz, $CDCl_3$) δ 158.48, 155.94, 139.69, 136.25, 131.96, 131.34, 129.88, 129.67, 128.71, 128.46, 128.35, 128.13, 127.48, 125.91, 125.00, 120.93, 116.29, 112.76, 112.02, 103.06, 97.62, 87.45, 70.57, 28.70, 15.56.

HRMS (ESI) *m/z* Calcd for $C_{27}H_{22}NaO_2^+$ $[M + Na]^+$: 401.1512, Found: 401.1511.

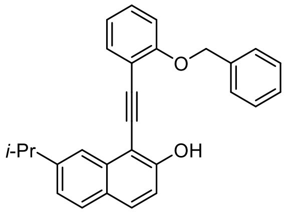

1c

1-((2-(benzyloxy)phenyl)ethynyl)-7-isopropylnaphthalen-2-ol (1c)

Compound **1c** is an unknown compound, and was synthesized in 58% yield (2 steps, 2 mmol, 455 mg) following the general procedure (**Method A**).

White solid. (R_f = 0.5, PE/EA= 10:1)

^{1}H NMR (400 MHz, $CDCl_3$) δ 8.00 (s, 1H), 7.69 (dd, *J* = 8.6, 4.1 Hz, 2H), 7.61 (d, *J* = 7.5 Hz, 1H), 7.47 (d, *J* = 7.3 Hz, 2H), 7.40 – 7.31 (m, 3H), 7.28 (t, *J* = 7.2 Hz, 2H), 7.12 (d, *J* = 8.9 Hz, 1H), 7.04 – 6.95 (m, 2H), 6.85 (s, 1H), 5.29 (s, 2H), 3.08 (hept, *J* = 6.8 Hz, 1H), 1.35 (d, *J* = 6.9 Hz, 6H).

^{13}C NMR (100 MHz, $CDCl_3$) ^{13}C NMR (101 MHz, $cdcl_3$) δ 158.39, 156.76, 148.01, 136.27, 133.11, 131.97, 130.09, 129.66, 128.71, 128.18, 128.12, 127.28, 126.86, 123.39, 121.63, 120.96, 115.53, 112.84, 112.15, 102.91, 97.79, 87.57, 70.55, 34.49, 23.95.

HRMS (ESI) *m/z* Calcd for $C_{28}H_{24}NaO_2^+$ $[M + Na]^+$: 415.1669, Found: 415.1665.

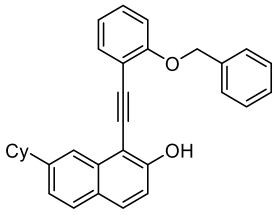

1d

1-((2-(benzyloxy)phenyl)ethynyl)-7-cyclohexylnaphthalen-2-ol (1d)

Compound **1d** is an unknown compound, and was synthesized in 64% yield (2 steps, 2 mmol, 554 mg) following the general procedure (**Method A**).

Yellow solid. (R_f = 0.5, PE/EA= 10:1)

^{1}H NMR (400 MHz, $CDCl_3$) δ 7.99 (s, 1H), 7.68 (d, *J* = 8.6 Hz, 2H), 7.63 (d, *J* = 7.4 Hz, 1H), 7.47 (d, *J* = 7.3 Hz, 2H), 7.36 (t, *J* = 7.2 Hz, 2H), 7.32 (d, *J* = 7.1 Hz, 1H), 7.28 – 7.22 (m, 2H), 7.11 (d, *J* = 8.9 Hz, 1H), 7.00 (t, *J* = 7.6 Hz, 1H), 6.97 (d, *J* = 8.4 Hz, 1H), 6.86 (s, 1H), 5.30 (s, 2H), 2.75 – 2.63 (m, 1H), 2.00 – 1.92 (m, 2H), 1.91 – 1.81 (m, 2H), 1.81 – 1.73 (m, 1H), 1.60 – 1.49 (m, 2H), 1.49 – 1.36 (m, 2H), 1.35 – 1.22 (m, 1H).

^{13}C NMR (100 MHz, $CDCl_3$) δ 158.39, 156.75, 147.24, 136.28, 133.13, 131.96, 130.08, 129.64, 128.70, 128.09, 128.08, 127.19, 126.89, 123.87, 122.03, 120.95, 115.49, 112.86, 112.17, 102.90, 97.76, 87.65, 70.52, 44.96, 34.33, 26.89, 26.16.

HRMS (ESI) *m/z* Calcd for $C_{31}H_{28}NaO_2^+$ [M + Na]$^+$: 455.1982, Found: 455.1980.

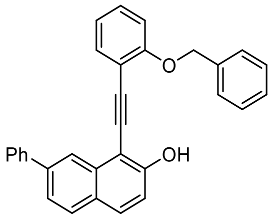

1e

1-((2-(benzyloxy)phenyl)ethynyl)-7-phenylnaphthalen-2-ol (1e)

Compound **1e** is an unknown compound, and was synthesized in 58% yield (2 steps, 4 mmol, 990 mg) following the general procedure (**Method A**).

White solid. (R_f = 0.5, PE/EA= 10:1)

^{1}H NMR (400 MHz, $CDCl_3$) δ 8.41 (s, 1H), 7.81 (d, *J* = 8.4 Hz, 1H), 7.74 (d, *J* = 7.9 Hz, 3H), 7.60 (t, *J* = 6.6 Hz, 2H), 7.45 (t, *J* = 7.1 Hz, 4H), 7.37 (d, *J* = 7.4 Hz, 1H), 7.36 – 7.28 (m, 3H), 7.26 (t, 1H), 7.17 (d, *J* = 8.9 Hz, 1H), 7.01 – 6.92 (m, 2H), 6.90 (s, 1H), 5.26 (s, 2H).

^{13}C NMR (100 MHz, $CDCl_3$) δ 158.44, 157.00, 141.22, 139.96, 136.23, 133.22, 131.95, 130.06, 129.75, 128.79, 128.69, 128.10, 127.53, 127.44, 127.40, 127.20, 123.62, 123.06, 120.95, 116.44, 112.66, 112.13, 103.52, 98.17, 87.36, 70.54.

HRMS (ESI) *m/z* Calcd for $C_{31}H_{22}NaO_2^+$ [M + Na]$^+$: 449.1512, Found: 449.1507.

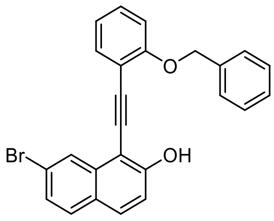

1f

1-((2-(benzyloxy)phenyl)ethynyl)-7-bromonaphthalen-2-ol (1f)

Compound **1f** is an unknown compound, and was synthesized in 50% yield (2 steps, 4 mmol, 859 mg) following the general procedure (**Method A**).

Yellow solid. (R_f = 0.5, PE/EA= 10:1)

^{1}H NMR (400 MHz, $CDCl_3$) δ 8.37 (s, 1H), 7.67 (d, *J* = 8.9 Hz, 1H), 7.61 (d, *J* = 8.3 Hz, 2H), 7.47 (d, *J* = 7.2 Hz, 2H), 7.43 (dd, *J* = 8.7, 1.7 Hz, 1H), 7.38 (t, *J* = 7.2 Hz, 2H), 7.35 – 7.26 (m, 2H), 7.17 (d, *J* = 8.9 Hz, 1H), 7.05 – 6.96 (m, 2H), 6.89 (s, 1H), 5.30 (s, 2H).

^{13}C NMR (100 MHz, $CDCl_3$) δ 158.50, 157.18, 136.17, 134.16, 131.97, 130.14, 129.96, 129.74, 128.75, 128.19, 127.36, 127.27, 126.69, 121.84, 121.01, 116.90, 112.36, 112.09, 102.69, 98.32, 86.63, 70.60.

HRMS (ESI) *m/z* Calcd for $C_{25}H_{17}BrNaO_2^+$ [M + Na]$^+$: 451.0304, Found: 451.0302.

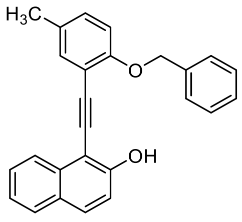

1g

1-((2-(benzyloxy)-5-methylphenyl)ethynyl)naphthalen-2-ol (1g)

Compound **1g** is an unknown compound, and was synthesized in 66% yield (2 steps, 3 mmol, 721 mg) following the general procedure (**Method A**).

White solid. (R_f = 0.5, PE/EA= 10:1)

^{1}H NMR (400 MHz, $CDCl_3$) δ 8.18 (d, *J* = 8.3 Hz, 1H), 7.74 (dd, *J* = 11.7, 8.7 Hz, 2H), 7.51 – 7.42 (m, 3H), 7.42 – 7.31 (m, 5H), 7.18 (d, *J* = 8.9 Hz, 1H), 7.08 (d, *J* = 7.4 Hz, 1H), 6.87 (d, *J* = 8.5 Hz, 1H), 6.83 (s, 1H), 5.23 (s, 2H), 2.31 (s, 3H).

^{13}C NMR (100 MHz, $CDCl_3$) δ 156.49, 136.44, 132.99, 132.30, 130.30, 130.26, 128.68, 128.26, 128.12, 128.09, 127.45, 127.16, 125.05, 123.83, 116.42, 112.37, 112.01, 103.32, 98.08, 86.94, 70.67, 20.34.

HRMS (ESI) *m/z* Calcd for $C_{26}H_{20}NaO_2^+$ [M + Na]$^+$: 387.1356, Found: 387.1355.

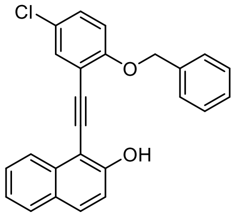

1h

1-((2-(benzyloxy)-5-chlorophenyl)ethynyl)naphthalen-2-ol (1h)

Compound **1h** is an unknown compound, and was synthesized in 67% yield (2 steps, 2 mmol, 516 mg) following the general procedure (**Method A**).

Yellow solid. (R_f = 0.5, PE/EA= 10:1)

^{1}H NMR (400 MHz, $CDCl_3$) δ 8.13 (d, *J* = 8.3 Hz, 1H), 7.74 (t, 2H), 7.55 (d, *J* = 2.5 Hz, 1H), 7.49 – 7.42 (m, 3H), 7.42 – 7.32 (m, 4H), 7.22 (dd, 1H), 7.16 (d, *J* = 8.9 Hz, 1H), 6.88 (d, *J* = 8.9 Hz, 1H), 6.69 (s, 1H), 5.22 (s, 2H).

^{13}C NMR (100 MHz, $CDCl_3$) δ 157.04, 156.77, 135.75, 132.97, 131.31, 130.82, 129.36, 128.79, 128.36, 128.25, 128.18, 127.51, 127.38, 125.79, 124.90, 123.98, 116.44, 114.30, 113.18, 102.72, 96.47, 88.45, 71.00.

HRMS (ESI) *m/z* Calcd for $C_{25}H_{17}ClNaO_2^+$ [M + Na]$^+$: 407.0809, Found: 407.0809.

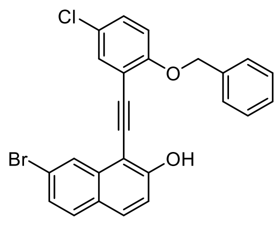

1i

1-((2-(benzyloxy)-5-chlorophenyl)ethynyl)-7-bromonaphthalen-2-ol (1i)

Compound **1i** is an unknown compound, and was synthesized in 59% yield (2 steps, 4 mmol, 1.09 g) following the general procedure (**Method A**).

Yellow Solid. (R_f = 0.4, PE/EA= 10:1)

^{1}H NMR (400 MHz, $CDCl_3$) δ 8.32 (s, 1H), 7.68 (d, *J* = 8.9 Hz, 1H), 7.60 (d, *J* = 8.6 Hz, 1H), 7.55 (d, *J* = 2.3 Hz, 1H), 7.47 – 7.30 (m, 6H), 7.22 (dd, *J* = 8.8, 2.3 Hz, 1H), 7.15 (d, *J* = 8.9 Hz, 1H), 6.87 (d, *J* = 8.9 Hz, 1H), 6.78 (s, 1H), 5.26 (s, 2H).

^{13}C NMR (100 MHz, $CDCl_3$) δ 157.42, 157.05, 135.69, 134.13, 131.26, 130.59, 129.77, 129.57, 128.82, 128.37, 127.41, 127.37, 127.15, 126.68, 125.86, 122.04, 116.91, 113.96, 113.25, 102.17, 96.91, 87.81, 71.00.

HRMS (ESI) *m/z* Calcd for $C_{25}H_{16}BrClNaO_2^+$ $[M + Na]^+$: 484.9914, Found: 484.9910.

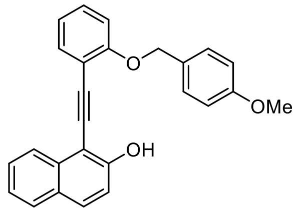

1j

1-((2-((4-methoxybenzyl)oxy)phenyl)ethynyl)naphthalen-2-ol (1j)

Compound **1j** is an unknown compound, and was synthesized in 57% yield (2 steps, 4 mmol, 867 mg) following the general procedure (**Method A**).

White solid. (R_f = 0.5, PE/EA= 10:1)

^{1}H NMR (400 MHz, $CDCl_3$) δ 8.17 (d, *J* = 8.3 Hz, 1H), 7.76 (d, 1H), 7.72 (d, *J* = 9.2 Hz, 1H), 7.58 (d, *J* = 7.7 Hz, 1H), 7.45 (t, *J* = 7.6 Hz, 1H), 7.41 (d, *J* = 8.5 Hz, 2H), 7.34 (t, *J* = 7.2 Hz, 1H), 7.29 (t, *J* = 7.9 Hz, 1H), 7.17 (d, *J* = 8.9 Hz, 1H), 7.00 (d, *J* = 7.9 Hz, 2H), 6.91 (d, *J* = 8.6 Hz, 2H), 6.81 (s, 1H), 5.18 (s, 2H), 3.80 (s, 3H).

^{13}C NMR (100 MHz, $CDCl_3$) δ 159.54, 158.56, 156.53, 132.97, 131.88, 130.30, 129.71, 129.27, 128.24, 128.11, 127.17, 125.03, 123.82, 120.86, 116.43, 114.10, 112.71, 112.08, 103.25, 97.90, 87.24, 70.42, 55.26.

HRMS (ESI) *m/z* Calcd for $C_{26}H_{20}NaO_3^+$ $[M + Na]^+$: 403.1305, Found: 403.1303.

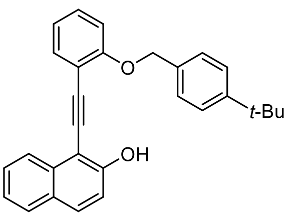

1k

1-((2-((4-(tert-butyl)benzyl)oxy)phenyl)ethynyl)naphthalen-2-ol (1k)

Compound **1k** is an unknown compound, and was synthesized in 44% yield (2 steps, 2 mmol, 358 mg) following the general procedure (**Method A**).

Yellow solid. (R_f = 0.5, PE/EA= 10:1)

^{1}H NMR (400 MHz, $CDCl_3$) δ 8.17 (d, *J* = 8.2 Hz, 1H), 7.73 (t, *J* = 9.7 Hz, 2H), 7.58 (d, *J* = 7.4 Hz, 1H), 7.46 – 7.37 (m, 5H), 7.35 – 7.27 (m, 2H), 7.15 (d, *J* = 8.7 Hz, 1H), 6.99 (t, *J* = 8.0 Hz, 2H), 6.77 (s, 1H), 5.23 (s, 2H), 1.33 (s, 9H).

^{13}C NMR (100 MHz, $CDCl_3$) δ 158.68, 156.51, 151.16, 133.17, 133.01, 131.86, 130.29, 129.74, 128.24, 128.09, 127.47, 127.18, 125.65, 125.09, 123.78, 120.84, 116.40, 112.68, 111.98, 103.27, 97.90, 87.27, 70.49, 34.60, 31.32.

HRMS (ESI) *m/z* Calcd for $C_{29}H_{26}NaO_2^+$ $[M + Na]^+$: 429.1825, Found: 429.1822.

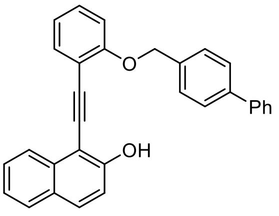

1l

1-((2-([1,1'-biphenyl]-4-ylmethoxy)phenyl)ethynyl)naphthalen-2-ol (1l)

Compound **1l** is an unknown compound, and was synthesized in 57% yield (2 steps, 3 mmol, 729 mg) following the general procedure (**Method A**).

White solid. (R_f = 0.5, PE/EA= 10:1)

^{1}H NMR (400 MHz, $CDCl_3$) δ 8.19 (d, *J* = 8.2 Hz, 1H), 7.73 (t, *J* = 9.1 Hz, 2H), 7.65 – 7.51 (m, 7H), 7.43 (t, *J* = 7.4 Hz, 3H), 7.38 – 7.27 (m, 3H), 7.17 (d, *J* = 8.9 Hz, 1H), 7.02 (d, *J* = 7.6 Hz, 2H), 6.82 (s, 1H), 5.30 (s, 2H).

^{13}C NMR (100 MHz, $CDCl_3$) δ 158.53, 156.52, 141.08, 140.71, 135.23, 133.00, 131.99, 130.37, 129.78, 128.76, 128.27, 128.13, 127.99, 127.46, 127.36, 127.20, 127.10, 125.05, 123.86, 121.01, 116.42, 112.74, 112.08, 103.25, 97.87, 87.31, 70.40.

HRMS (ESI) *m/z* Calcd for $C_{31}H_{22}NaO_2^+$ $[M + Na]^+$: 449.1512, Found: 449.1511.

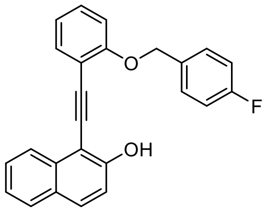

1m

1-((2-((4-fluorobenzyl)oxy)phenyl)ethynyl)naphthalen-2-ol (1m)

Compound **1m** is an unknown compound, and was synthesized in 70% yield (2 steps, 4 mmol, 1.03 g) following the general procedure (**Method A**).

Yellow solid. (R_f = 0.5, PE/EA= 10:1)

^{1}H NMR (400 MHz, $CDCl_3$) δ 8.16 (d, *J* = 8.3 Hz, 1H), 7.74 (t, *J* = 9.4 Hz, 2H), 7.59 (dd, 1H), 7.51 – 7.42 (m, 3H), 7.35 (t, *J* = 7.6 Hz, 1H), 7.30 (t, *J* = 7.3 Hz, 1H), 7.17 (d, *J* = 8.9 Hz, 1H), 7.07 (t, *J* = 8.6 Hz, 2H), 7.02 (t, *J* = 7.5 Hz, 1H), 6.97 (d, *J* = 8.4 Hz, 1H), 6.73 (s, 1H), 5.20 (s, 2H).

^{13}C NMR (100 MHz, $CDCl_3$) δ 162.63 (d, *J* = 245.0 Hz), 158.35, 156.50, 132.96, 132.04, 131.94 (d, *J* = 3.0 Hz), 130.42, 129.76, 129.52 (d, *J* = 8.0 Hz), 128.27, 128.16, 127.19, 124.96, 123.90, 121.12, 116.39, 115.66 (d, *J* = 22.0 Hz), 112.74, 111.96, 103.15, 97.73, 87.29, 69.99.

HRMS (ESI) *m/z* Calcd for $C_{25}H_{17}FNaO_2^+$ $[M + Na]^+$: 391.1105, Found: 391.1102.

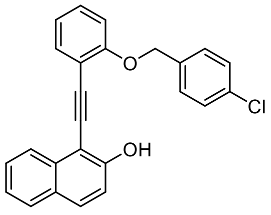

1n

1-((2-((4-chlorobenzyl)oxy)phenyl)ethynyl)naphthalen-2-ol (1n)

Compound **1n** is an unknown compound, and was synthesized in 67% yield (2 steps, 3 mmol, 774 mg) following the general procedure (**Method A**).

Yellow solid. (R_f = 0.5, PE/EA= 10:1)

^{1}H NMR (400 MHz, $CDCl_3$) δ 8.16 (d, *J* = 8.3 Hz, 1H), 7.75 (t, *J* = 8.8 Hz, 2H), 7.59 (d, *J* = 7.5 Hz, 1H), 7.46 (t, 1H), 7.42 (d, *J* = 8.3 Hz, 2H), 7.39 – 7.33 (m, 3H), 7.30 (t, *J* = 7.9 Hz, 1H), 7.18 (d, *J* = 8.9 Hz, 1H), 7.01 (t, *J* = 7.5 Hz, 1H), 6.95 (d, *J* = 8.4 Hz, 1H), 6.74 (s, 1H), 5.21 (s, 2H).

^{13}C NMR (100 MHz, $CDCl_3$) δ 158.23, 156.48, 134.68, 134.02, 132.94, 132.07, 130.46, 129.78, 128.91, 128.90, 128.27, 128.17, 127.22, 124.95, 123.93, 121.19, 116.38, 112.71, 111.95, 103.14, 97.69, 87.31, 69.84.

HRMS (ESI) *m/z* Calcd for $C_{25}H_{17}ClNaO_2^+$ $[M + Na]^+$: 407.0809, Found: 407.0804.

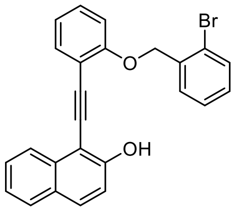

1a-1

1-((2-((2-bromobenzyl)oxy)phenyl)ethynyl)naphthalen-2-ol (1a-1)

Compound **1a-1** is an unknown compound, and was synthesized in 65% yield (2 steps, 4 mmol, 1.12 g) following the general procedure (**Method A**).

White solid. (R_f = 0.5, PE/EA= 5:1)

^{1}H NMR (400 MHz, $CDCl_3$) δ 8.21 (d, *J* = 8.2 Hz, 1H), 7.74 (t, *J* = 9.3 Hz, 2H), 7.60 (t, *J* = 6.8 Hz, 3H), 7.48 (t, *J* = 7.4 Hz, 1H), 7.35 (t, 1H), 7.32 – 7.25 (m, 2H), 7.22 – 7.14 (m, 2H), 7.02 (t, *J* = 7.5 Hz, 1H), 6.95 (d, *J* = 8.3 Hz, 1H), 6.76 (s, 1H), 5.32 (s, 2H).

^{13}C NMR (100 MHz, $CDCl_3$) δ 158.18, 156.46, 135.50, 133.02, 132.68, 132.17, 130.43, 129.91, 129.46, 128.90, 128.28, 128.16, 127.77, 127.23, 125.01, 123.89, 122.22, 121.21, 116.37, 112.59, 112.00, 103.19, 97.72, 87.18, 69.93.

HRMS (ESI) *m/z* Calcd for $C_{25}H_{17}BrNaO_2^+$ $[M + Na]^+$: 451.0304, Found: 451.0302.

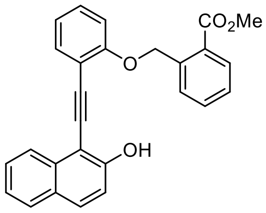

1a-2

methyl 2-((2-((2-hydroxynaphthalen-1-yl)ethynyl)phenoxy)methyl)benzoate (1a-2)

Compound **1a-2** is an unknown compound, and was synthesized in 56% yield (2 steps, 3 mmol, 686 mg) following the general procedure (**Method A**).

Yellow solid. (R_f = 0.5, PE/EA= 5:1)

^{1}H NMR (400 MHz, $CDCl_3$) δ 8.23 (d, *J* = 8.2 Hz, 1H), 8.04 (d, *J* = 7.7 Hz, 1H), 7.81 (d, *J* = 7.7 Hz, 1H), 7.76 (t, *J* = 9.3 Hz, 2H), 7.62 (d, *J* = 7.4 Hz, 1H), 7.49 (t, *J* = 7.1 Hz, 2H), 7.40 – 7.33 (m, 2H), 7.30 (t, *J* = 7.8 Hz, 1H), 7.20 (d, *J* = 8.8 Hz, 1H), 7.05 – 6.95 (m, 2H), 6.85 (s, 1H), 5.71 (s, 2H), 3.89 (s, 3H).

^{13}C NMR (100 MHz, $CDCl_3$) δ 167.33, 158.51, 156.40, 138.85, 133.10, 132.83, 132.23, 130.80, 130.38, 129.94, 128.28, 128.15, 127.59, 127.45, 127.37, 127.20, 125.01, 123.86, 120.94, 116.45, 112.42, 112.11, 103.25, 97.82, 86.99, 68.73, 52.13.

HRMS (ESI) *m/z* Calcd for $C_{27}H_{20}NaO_4^+$ $[M + Na]^+$: 431.1254, Found: 431.1251.

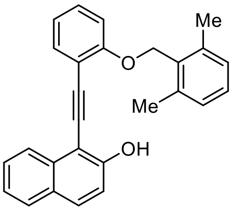

1a-3

1-((2-((2,6-dimethylbenzyl)oxy)phenyl)ethynyl)naphthalen-2-ol (1a-3)

Compound **1a-3** is an unknown compound, and was synthesized in 47% yield (2 steps, 5 mmol, 889 mg) following the general procedure (**Method A**).

White solid. (R_f = 0.4, PE/EA= 10:1)

^{1}H NMR (400 MHz, $CDCl_3$) δ 8.05 – 7.99 (m, 1H), 7.73 – 7.68 (m, 1H), 7.66 (d, *J* = 9.0 Hz, 1H), 7.59 (d, *J* = 7.5 Hz, 1H), 7.38 (t, *J* = 7.8 Hz, 1H), 7.29 (dd, *J* = 6.0, 3.1 Hz, 2H), 7.22 (d, *J* = 8.1 Hz, 1H), 7.13 (t, *J* = 9.6 Hz, 3H), 7.08 – 7.00 (m, 2H), 6.26 (s, 1H), 5.20 (s, 2H), 2.44 (s, 6H).

^{13}C NMR (100 MHz, $CDCl_3$) δ 159.13, 156.28, 138.21, 133.04, 132.03, 130.18, 129.78, 128.73, 128.43, 128.13, 128.00, 127.12, 124.93, 123.73, 120.85, 116.43, 112.66, 111.63, 103.04, 97.62, 86.79, 65.54, 19.66.

HRMS (ESI) *m/z* Calcd for $C_{27}H_{22}NaO_2^+$ $[M + Na]^+$: 401.1512, Found: 401.1510.

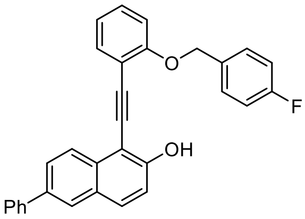

1o

1-((2-((4-fluorobenzyl)oxy)phenyl)ethynyl)-6-phenylnaphthalen-2-ol (1o)

Compound **1o** is an unknown compound, and was synthesized in 59% yield (2 steps, 3 mmol, 787 mg) following the general procedure (**Method A**).

Yellow solid. (R_f = 0.5, PE/EA= 10:1)

^{1}H NMR (400 MHz, $CDCl_3$) δ 8.20 (d, *J* = 8.6 Hz, 1H), 7.95 (s, 1H), 7.78 (d, *J* = 8.9 Hz, 1H), 7.75 – 7.66 (m, 3H), 7.60 (d, *J* = 7.5 Hz, 1H), 7.52 – 7.43 (m, 4H), 7.36 (t, *J* = 7.3 Hz, 1H), 7.31 (t, *J* = 7.9 Hz, 1H), 7.19 (d, *J* = 8.9 Hz, 1H), 7.08 (t, *J* = 8.6 Hz, 2H), 7.03 (t, *J* = 7.5 Hz, 1H), 6.98 (d, *J* = 8.4 Hz, 1H), 6.72 (s, 1H), 5.20 (s, 2H).

^{13}C NMR (100 MHz, $CDCl_3$) δ 162.66 (d, *J* = 245.0 Hz), 158.41, 156.55, 140.88, 136.68, 132.16, 132.05, 131.95 (d, *J* = 3.0 Hz), 130.68, 129.81, 129.63 (d, *J* = 8.0 Hz), 128.84, 128.53, 127.19, 126.80, 126.07, 125.55, 121.13, 116.83, 115.81, 115.59, 112.70, 111.95, 103.12, 97.79, 87.24, 70.02.

HRMS (ESI) *m/z* Calcd for $C_{31}H_{21}FNaO_2^+$ $[M + Na]^+$: 467.1418, Found: 467.1416.

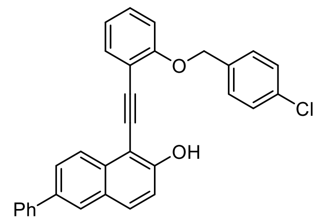

1p

1-((2-((4-chlorobenzyl)oxy)phenyl)ethynyl)-6-phenylnaphthalen-2-ol (1p)

Compound **1p** is an unknown compound, and was synthesized in 60% yield (2 steps, 3 mmol, 830 mg) following the general procedure (**Method A**).

White solid. (R_f = 0.5, PE/EA= 10:1)

^{1}H NMR (400 MHz, $CDCl_3$) δ 8.19 (d, J = 8.6 Hz, 1H), 7.95 (s, 1H), 7.78 (d, J = 8.9 Hz, 1H), 7.73 – 7.66 (m, 3H), 7.60 (d, J = 7.5 Hz, 1H), 7.47 (t, J = 7.6 Hz, 2H), 7.43 (d, J = 8.2 Hz, 2H), 7.39 – 7.33 (m, 3H), 7.30 (t, J = 7.9 Hz, 1H), 7.20 (d, J = 8.9 Hz, 1H), 7.02 (t, J = 7.5 Hz, 1H), 6.95 (d, J = 8.3 Hz, 1H), 6.73 (s, 1H), 5.20 (s, 2H).

^{13}C NMR (100 MHz, $CDCl_3$) δ 158.33, 156.50, 140.84, 136.70, 134.69, 134.06, 132.18, 132.10, 130.72, 129.83, 129.01, 128.94, 128.83, 128.55, 127.21, 127.18, 126.82, 126.06, 125.55, 121.20, 116.82, 112.69, 111.96, 103.12, 97.76, 87.24, 69.90.

HRMS (ESI) *m/z* Calcd for $C_{31}H_{21}ClNaO_2^+$ $[M + Na]^+$: 483.1122, Found: 483.1121.

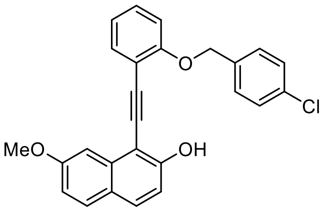

1q

1-((2-((4-chlorobenzyl)oxy)phenyl)ethynyl)-7-methoxynaphthalen-2-ol (1q)

Compound **1q** is an unknown compound, and was synthesized in 67% yield (2 steps, 3 mmol, 834 mg) following the general procedure (**Method A**).

Yellow solid. (R_f = 0.5, PE/EA= 10:1)

^{1}H NMR (400 MHz, $CDCl_3$) δ 7.66 (d, *J* = 8.8 Hz, 2H), 7.58 (d, *J* = 8.6 Hz, 1H), 7.50 (d, *J* = 2.1 Hz, 1H), 7.40 (d, *J* = 8.3 Hz, 2H), 7.34 (s, 1H), 7.33 – 7.26 (m, 2H), 7.04 (d, *J* = 5.9 Hz, 1H), 7.03 – 6.99 (m, 2H), 6.94 (d, *J* = 8.3 Hz, 1H), 6.76 (s, 1H), 5.23 (s, 2H), 3.92 (s, 3H).

^{13}C NMR (100 MHz, $CDCl_3$) δ 159.09, 158.14, 157.13, 134.72, 134.53, 133.99, 132.03, 130.29, 129.79, 129.71, 128.92, 128.67, 123.59, 121.21, 116.06, 113.80, 112.87, 112.14, 104.05, 102.34, 97.75, 87.54, 69.85, 55.24.

HRMS (ESI) *m/z* Calcd for $C_{26}H_{19}ClNaO_3^+$ $[M + Na]^+$: 437.0915, Found: 437.0912.

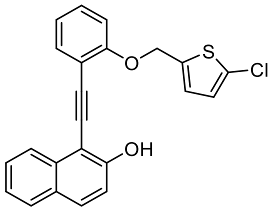

1r

1-((2-((5-chlorothiophen-2-yl)methoxy)phenyl)ethynyl)naphthalen-2-ol (1r)

Compound **1r** is an unknown compound, and was synthesized in 56% yield (2 steps, 3 mmol, 657 mg) following the general procedure (**Method A**).

Yellow solid. (R_f = 0.5, PE/EA= 10:1)

^{1}H NMR (400 MHz, $CDCl_3$) δ 8.18 (d, J = 8.3 Hz, 1H), 7.75 (t, J = 8.1 Hz, 2H), 7.59 (d, J = 7.3 Hz, 1H), 7.49 (t, J = 7.5 Hz, 1H), 7.39 – 7.28 (m, 2H), 7.20 (d, J = 8.9 Hz, 1H), 7.07 – 6.97 (m, 2H), 6.94 (d, J = 3.4 Hz, 1H), 6.82 (d, J = 3.6 Hz, 1H), 6.70 (s, 1H), 5.27 (s, 2H).

^{13}C NMR (100 MHz, $CDCl_3$) δ 157.79, 156.48, 137.04, 133.03, 132.24, 131.11, 130.47, 129.71, 128.29, 128.15, 127.21, 126.88, 125.92, 125.04, 123.92, 121.52, 116.42, 113.04, 112.07, 103.12, 97.48, 87.30, 65.53.

HRMS (ESI) *m/z* Calcd for $C_{23}H_{15}ClNaO_2S^+$ $[M + Na]^+$: 413.0373, Found: 413.0371.

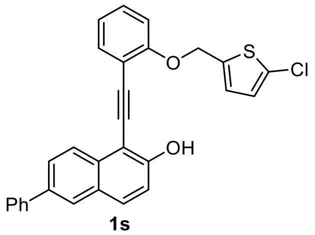

1s

1-((2-((5-chlorothiophen-2-yl)methoxy)phenyl)ethynyl)-6-phenylnaphthalen-2-ol (1s)

Compound **1s** is an unknown compound, and was synthesized in 61% yield (2 steps, 5 mmol, 1.42 g) following the general procedure (**Method A**).

White solid. (R_f = 0.5, PE/EA= 10:1)

^{1}H NMR (400 MHz, $CDCl_3$) δ 8.23 (d, J = 8.6 Hz, 1H), 7.96 (s, 1H), 7.79 (d, J = 8.9 Hz, 1H), 7.75 (dd, 1H), 7.71 (d, J = 7.5 Hz, 2H), 7.60 (d, J = 7.5 Hz, 1H), 7.48 (t, J = 7.6 Hz, 2H), 7.37 (d, J = 7.3 Hz, 1H), 7.35 – 7.30 (m, 1H), 7.22 (d, J = 8.9 Hz, 1H), 7.08 – 6.99 (m, 2H), 6.96 (d, J = 3.6 Hz, 1H), 6.84 (d, J = 3.7 Hz, 1H), 6.70 (s, 1H), 5.28 (s, 2H).

^{13}C NMR (100 MHz, $CDCl_3$) δ 157.86, 156.51, 140.91, 137.04, 136.71, 132.27, 132.25, 131.16, 130.74, 129.77, 128.82, 128.55, 127.22, 127.17, 126.95, 126.81, 126.06, 125.95, 125.64, 121.53, 116.86, 112.98, 112.04, 103.09, 97.53, 87.23, 65.54.

HRMS (ESI) *m/z* Calcd for $C_{29}H_{19}ClNaO_2S^+$ $[M + Na]^+$: 489.0686, Found: 489.0683.

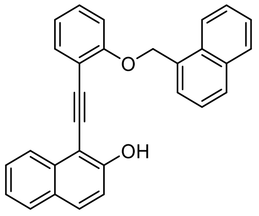

1t

1-((2-(naphthalen-1-ylmethoxy)phenyl)ethynyl)naphthalen-2-ol (1t)

Compound **1t** is an unknown compound, and was synthesized in 67% yield (2 steps, 3 mmol, 805 mg) following the general procedure (**Method A**).

Yellow solid. (R_f = 0.5, PE/EA= 10:1)

^{1}H NMR (400 MHz, $CDCl_3$) δ 8.14 – 8.02 (m, 2H), 7.89 (d, 1H), 7.86 (d, *J* = 8.8 Hz, 1H), 7.70 (d, *J* = 3.4 Hz, 1H), 7.67 (d, *J* = 7.6 Hz, 1H), 7.64 (t, 1H), 7.60 (d, *J* = 7.4 Hz, 1H), 7.50 – 7.41 (m, 3H), 7.35 – 7.25 (m, 3H), 7.08 (d, *J* = 8.5 Hz, 1H), 7.05 (d, *J* = 9.1 Hz, 1H), 7.01 (t, 1H), 6.49 (s, 1H), 5.67 (s, 2H).

^{13}C NMR (100 MHz, $CDCl_3$) δ 158.67, 156.32, 133.77, 133.00, 132.19, 131.54, 131.30, 130.26, 129.82, 129.07, 128.71, 128.17, 128.02, 127.09, 126.54, 126.36, 125.92, 125.31, 124.93, 123.76, 123.30, 121.07, 116.32, 112.79, 112.17, 103.09, 97.68, 87.02, 69.19.

HRMS (ESI) *m/z* Calcd for $C_{29}H_{20}NaO_2^+$ $[M + Na]^+$: 423.1356, Found: 423.1355.

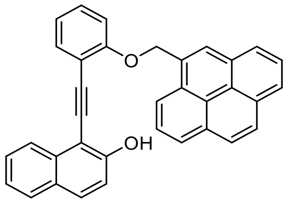

1u

1-((2-(pyren-4-ylmethoxy)phenyl)ethynyl)naphthalen-2-ol (1u)

Compound **1u** is an unknown compound, and was synthesized in 56% yield (2 steps, 3 mmol, 797 mg) following the general procedure (**Method A**).

Brown solid. (R_f = 0.5, PE/EA= 10:1)

^{1}H NMR (400 MHz, $CDCl_3$) δ 8.31 (d, *J* = 9.2 Hz, 1H), 8.16 (d, *J* = 7.5 Hz, 1H), 8.14 – 7.98 (m, 7H), 7.96 (t, *J* = 7.6 Hz, 1H), 7.61 (t, *J* = 8.1 Hz, 2H), 7.56 (d, *J* = 9.0 Hz, 1H), 7.29 (t, *J* = 7.8 Hz, 1H), 7.17 (t, *J* = 7.4 Hz, 1H), 7.14 – 7.05 (m, 2H), 7.00 (t, *J* = 7.4 Hz, 1H), 6.90 (d, *J* = 8.9 Hz, 1H), 6.54 (s, 1H), 5.89 (s, 2H).

^{13}C NMR (100 MHz, $CDCl_3$) δ 158.71, 156.29, 132.92, 132.19, 131.54, 131.15, 130.66, 130.20, 129.81, 129.10, 129.02, 128.17, 128.11, 127.95, 127.64, 127.36, 126.96, 126.54, 125.94, 125.40, 125.37, 124.93, 124.85, 124.71, 124.64, 123.68, 122.61, 121.13, 116.23, 112.90, 112.37, 103.07, 97.73, 87.12, 69.37.

HRMS (ESI) *m/z* Calcd for $C_{35}H_{22}NaO_2^+$ $[M + Na]^+$: 497.1512, Found: 497.1511.

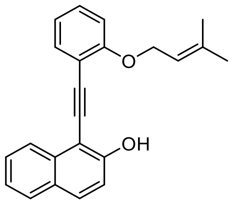

1v

1-((2-((3-methylbut-2-en-1-yl)oxy)phenyl)ethynyl)naphthalen-2-ol (1v)

Compound **1v** is an unknown compound, and was synthesized in 49% yield (2 steps, 2 mmol, 322 mg) following the general procedure (**Method B**).

Yellow oil. (R_f = 0.6, PE/EA= 10:1)

^{1}H NMR (400 MHz, $CDCl_3$) δ 8.22 (d, *J* = 8.3 Hz, 1H), 7.74 (t, *J* = 8.8 Hz, 2H), 7.58 – 7.49 (m, 2H), 7.34 (t, *J* = 7.4 Hz, 1H), 7.29 (t, *J* = 7.9 Hz, 1H), 7.23 (d, *J* = 8.9 Hz, 1H), 7.05 (s, 1H), 6.97 (t, *J* = 7.6 Hz, 1H), 6.93 (d, *J* = 8.4 Hz, 1H), 5.60 (t, *J* = 6.0 Hz, 1H), 4.66 (d, *J* = 6.6 Hz, 2H), 1.84 (s, 3H), 1.76 (s, 3H).

^{13}C NMR (100 MHz, $CDCl_3$) δ 158.73, 156.82, 139.22, 132.85, 131.41, 130.22, 129.63, 128.21, 128.11, 127.09, 125.06, 123.78, 120.52, 118.92, 116.46, 112.47, 111.48, 103.31, 97.96, 87.49, 65.41, 25.80, 18.25.

HRMS (ESI) *m/z* Calcd for $C_{23}H_{20}NaO_2^+$ $[M + Na]^+$: 351.1356, Found: 351.1353.

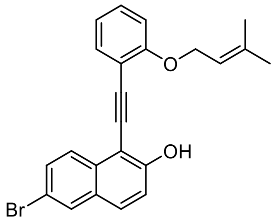

1w

6-bromo-1-((2-((3-methylbut-2-en-1-yl)oxy)phenyl)ethynyl)naphthalen-2-ol (1w)

Compound **1w** is an unknown compound, and was synthesized in 43% yield (2 steps, 2 mmol, 350 mg) following the general procedure (**Method B**).

Yellow solid. (R_f = 0.6, PE/EA= 10:1)

^{1}H NMR (400 MHz, $CDCl_3$) δ 8.08 (d, *J* = 8.8 Hz, 1H), 7.91 (s, 1H), 7.64 (d, *J* = 8.9 Hz, 1H), 7.57 (t, *J* = 9.4 Hz, 2H), 7.34 (t, *J* = 7.7 Hz, 1H), 7.24 (d, *J* = 9.0 Hz, 1H), 7.06 (s, 1H), 7.00 (d, *J* = 7.5 Hz, 1H), 6.97 (d, *J* = 8.0 Hz, 1H), 5.61 (t, *J* = 5.9 Hz, 1H), 4.69 (d, *J* = 6.6 Hz, 2H), 1.86 (s, 3H), 1.79 (s, 3H).

^{13}C NMR (100 MHz, $CDCl_3$) δ 158.83, 156.98, 139.42, 131.46, 131.39, 130.26, 130.05, 129.90, 129.35, 129.13, 126.94, 120.59, 118.84, 117.60, 117.46, 112.19, 111.52, 103.65, 98.40, 86.90, 65.46, 25.85, 18.29.

HRMS (ESI) *m/z* Calcd for $C_{23}H_{19}BrNaO_2^+$ $[M + Na]^+$: 429.0461, Found: 429.0460.

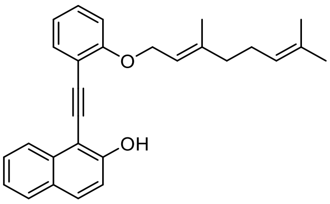

1x

(E)-1-((2-((3,7-dimethylocta-2,6-dien-1-yl)oxy)phenyl)ethynyl)naphthalen-2-ol (1x)

Compound **1x** is an unknown compound, and was synthesized in 58% yield (2 steps, 4 mmol, 920 mg) following the general procedure (**Method B**).

Brown oil. (R_f = 0.6, PE/EA= 10:1)

^{1}H NMR (400 MHz, $CDCl_3$) δ 8.22 (d, *J* = 8.2 Hz, 1H), 7.75 (t, *J* = 9.1 Hz, 2H), 7.55 (dd, 2H), 7.39 – 7.29 (m, 2H), 7.22 (d, *J* = 8.9 Hz, 1H), 7.08 (s, 1H), 7.02 – 6.93 (m, 2H), 5.63 (t, *J* = 5.4 Hz, 1H), 5.12 (t, *J* = 6.3 Hz, 1H), 4.72 (d, *J* = 6.4 Hz, 2H), 2.23 – 2.09 (m, 4H), 1.78 (s, 3H), 1.67 (s, 3H), 1.62 (s, 3H).

^{13}C NMR (100 MHz, $CDCl_3$) δ 158.75, 156.90, 142.31, 132.84, 131.77, 131.42, 130.23, 129.64, 128.22, 128.13, 127.11, 125.07, 123.89, 123.79, 120.56, 118.68, 116.48, 112.50, 111.55, 103.31, 97.97, 87.56, 65.57, 39.56, 26.22, 25.67, 17.72, 16.77.

HRMS (ESI) *m/z* Calcd for $C_{28}H_{28}NaO_2^+$ [M + Na]$^+$: 419.1982, Found: 419.1980.

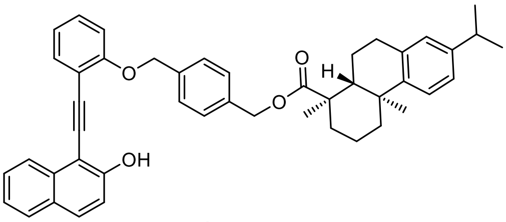

1y

4-((2-((2-hydroxynaphthalen-1-yl)ethynyl)phenoxy)methyl)benzyl (1R,4aS,10aR)-7-isopropyl-1,4a-dimethyl-1,2,3,4,4a,9,10,10a-octahydrophenanthrene-1-carboxylate (1y)

Compound **1y** is an unknown compound, and was synthesized in 45% yield (2 steps, 2 mmol, 597 mg) following the general procedure (**Method C**).

Yellow solid. (R_f = 0.3, PE/EA= 10:1)

^{1}H NMR (400 MHz, $CDCl_3$) δ 8.18 (d, *J* = 8.3 Hz, 1H), 7.73 (dd, *J* = 12.2, 8.5 Hz, 2H), 7.59 (d, *J* = 7.5 Hz, 1H), 7.46 (t, *J* = 8.8 Hz, 3H), 7.34 (d, *J* = 7.7 Hz, 3H), 7.29 (t, *J* = 7.3 Hz, 1H), 7.17 (d, *J* = 9.0 Hz, 1H), 7.13 (s, 1H), 7.03 – 6.95 (m, 3H), 6.82 (d, *J* = 3.3 Hz, 2H), 5.27 (s, 2H), 5.16 (d, *J* = 12.5 Hz, 1H), 5.07 (d, *J* = 12.5 Hz, 1H), 2.82 – 2.71 (m, 3H), 2.26 (t, *J* = 10.8 Hz, 2H), 1.84 – 1.61 (m, 6H), 1.48 (dd, *J* = 17.2, 7.9 Hz, 1H), 1.28 (s, 3H), 1.20 (s, 3H), 1.18 (s, 6H).

^{13}C NMR (100 MHz, $CDCl_3$) δ 178.26, 158.39, 156.54, 146.75, 145.64, 136.30, 136.12, 134.61, 132.97, 131.99, 130.38, 129.74, 128.38, 128.27, 128.14, 127.56, 127.18, 126.90, 125.01, 124.14, 123.86, 121.04, 116.40, 112.73, 112.04, 103.21, 97.79, 87.35, 70.26, 65.98, 47.67, 44.83, 37.88, 36.95, 36.48, 33.40, 29.96, 25.24, 23.95, 23.92, 21.64, 18.56, 16.57.

HRMS (ESI) *m/z* Calcd for $C_{46}H_{46}NaO_4^+$ [M + Na]$^+$: 685.3288, Found: 685.3284.

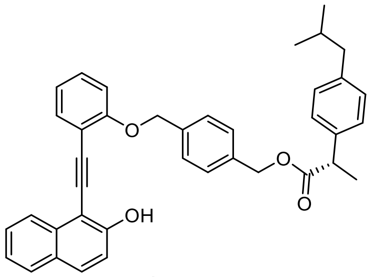

1z

4-((2-((2-hydroxynaphthalen-1-yl)ethynyl)phenoxy)methyl)benzyl (S)-2-(4-isobutylphenyl)propanoate (1z)

Compound **1z** is an unknown compound, and was synthesized in 65% yield (2 steps, 4 mmol, 1.48 g) following the general procedure (**Method C**).

Yellow oil. (R_f = 0.4, PE/EA= 10:1)

^{1}H NMR (400 MHz, $CDCl_3$) δ 8.17 (d, *J* = 8.3 Hz, 1H), 7.74 (t, *J* = 9.4 Hz, 2H), 7.61 – 7.56 (m, 1H), 7.45 (d, *J* = 7.1 Hz, 1H), 7.41 (d, *J* = 8.1 Hz, 2H), 7.34 (t, *J* = 7.5 Hz, 1H), 7.28 (t, *J* = 7.9 Hz, 1H), 7.22 (d, *J* = 7.9 Hz, 2H), 7.20 – 7.15 (m, 3H), 7.05 (d, *J* = 7.9 Hz, 2H), 6.99 (t, *J* = 7.5 Hz, 1H), 6.95 (d, *J* = 8.4 Hz, 1H), 6.81 (s, 1H), 5.23 (s, 2H), 5.10 (s, 2H), 3.74 (q, *J* = 7.1 Hz, 1H), 2.41 (d, *J* = 7.2 Hz, 2H), 1.80 (hept, *J* = 13.5, 6.7 Hz, 1H), 1.50 (d, *J* = 7.2 Hz, 3H), 0.86 (d, *J* = 6.6 Hz, 6H).

^{13}C NMR (100 MHz, $CDCl_3$) δ 174.47, 158.39, 156.51, 140.54, 137.49, 136.04, 132.98, 131.99, 130.38, 129.73, 129.28, 128.27, 128.13, 128.03, 127.49, 127.19, 127.17, 125.00, 123.87, 121.02, 116.39, 112.71, 112.01, 103.21, 97.79, 87.31, 70.24, 65.92, 45.11, 44.96, 30.13, 22.32, 18.37.

HRMS (ESI) *m/z* Calcd for $C_{39}H_{36}NaO_4^+$ [M + Na]$^+$: 591.2506, Found: 591.2505.

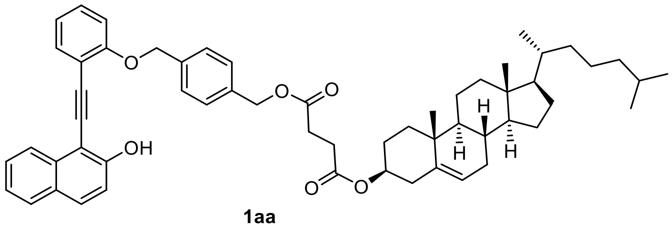

1aa

(3S,8S,9S,10R,13R,14S,17R)-10,13-dimethyl-17-((R)-6-methylheptan-2-yl)-2,3,4,7,8,9,10,11,12,13,14,15,16,17-tetradecahydro-1H-cyclopenta[a]phenanthren-3-yl (4-((2-((2-hydroxynaphthalen-1-yl)ethynyl)phenoxy)methyl)benzyl) succinate (1aa)

Compound **1aa** is an unknown compound, and was synthesized in 67% yield (2 steps, 3 mmol, 1.71 g) following the general procedure (**Method C**).

Yellow solid. (R_f = 0.3, PE/EA= 10:1)

^{1}H NMR (400 MHz, $CDCl_3$) δ 8.17 (d, *J* = 8.3 Hz, 1H), 7.74 (t, *J* = 9.1 Hz, 2H), 7.59 (d, *J* = 7.5 Hz, 1H), 7.49 (d, *J* = 7.8 Hz, 2H), 7.44 (d, *J* = 7.9 Hz, 1H), 7.40 – 7.32 (m, 3H), 7.29 (t, *J* = 7.9 Hz, 1H), 7.18 (d, *J* = 8.9 Hz, 1H), 7.04 – 6.94 (m, 2H), 6.81 (s, 1H), 5.33 (d, 1H), 5.26 (s, 2H), 5.14 (s, 2H), 4.67 – 4.55 (m, 1H), 2.72 – 2.57 (m, 4H), 2.27 (d, *J* = 6.9 Hz, 2H), 2.01 – 1.88 (m, 2H), 1.87 – 1.75

(m, 3H), 1.59 – 1.47 (m, 4H), 1.47 – 1.40 (m, 3H), 1.40 – 1.30 (m, 4H), 1.30 – 1.19 (m, 2H), 1.18 – 0.99 (m, 8H), 0.97 (s, 3H), 0.90 (d, J = 6.3 Hz, 3H), 0.86 (d, J = 6.5 Hz, 6H), 0.65 (s, 3H).

^{13}C NMR (100 MHz, $CDCl_3$) δ 172.09, 171.55, 158.40, 156.50, 139.47, 136.34, 135.73, 133.01, 132.05, 130.37, 129.74, 128.53, 128.27, 128.13, 127.60, 127.19, 125.01, 123.85, 122.65, 121.05, 116.42, 112.75, 112.00, 103.22, 97.74, 87.31, 74.38, 70.22, 66.14, 56.60, 56.08, 49.92, 42.25, 39.66, 39.49, 37.98, 36.88, 36.50, 36.15, 35.76, 31.83, 31.78, 29.46, 29.26, 28.19, 27.99, 27.66, 24.23, 23.81, 22.81, 22.55, 20.97, 19.25, 18.69, 11.81.

HRMS (ESI) *m/z* Calcd for $C_{57}H_{68}NaO_6^+$ $[M + Na]^+$: 871.4908, Found: 871.4905.

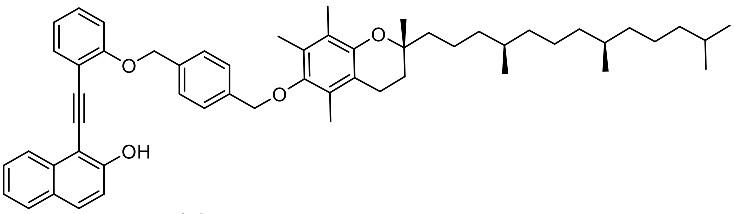

1ab

1-((2-((4-((((R)-2,5,7,8-tetramethyl-2-((4R,8R)-4,8,12-trimethyltridecyl)chroman-6-yl)oxy)methyl)benzyl)oxy)phenyl)ethynyl)naphthalen-2-ol (1ab)

Compound **1ab** is an unknown compound, and was synthesized in 70% yield (2 steps, 4 mmol, 2.22 g) following the general procedure (**Method C**).

Yellow solid. (R_f = 0.4, PE/EA= 10:1)

^{1}H NMR (400 MHz, $CDCl_3$) δ 8.19 (d, J = 8.2 Hz, 1H), 7.75 (t, J = 8.5 Hz, 2H), 7.60 (d, J = 7.1 Hz, 1H), 7.56 – 7.49 (m, 4H), 7.49 – 7.43 (m, 1H), 7.37 – 7.27 (m, 2H), 7.19 (d, J = 8.9 Hz, 1H), 7.05 – 6.97 (m, 2H), 6.85 (s, 1H), 5.31 (s, 2H), 4.71 (s, 2H), 2.58 (t, J = 6.4 Hz, 2H), 2.21 (s, 3H), 2.16 (s, 3H), 2.10 (s, 3H), 1.86 – 1.72 (m, 2H), 1.55 – 1.04 (m, 24H), 0.93 – 0.78 (m, 12H).

^{13}C NMR (100 MHz, $CDCl_3$) δ 158.45, 156.52, 148.03, 147.89, 138.00, 135.75, 132.99, 131.94, 130.34, 129.74, 128.26, 128.11, 128.04, 127.87, 127.58, 127.22, 125.91, 125.04, 123.85, 122.91, 120.97, 117.57, 116.39, 112.71, 112.09, 103.26, 97.87, 87.31, 74.79, 74.35, 70.38, 40.01, 39.35, 37.44, 37.41, 37.27, 32.78, 32.68, 31.28, 27.96, 24.79, 24.43, 23.86, 22.72, 22.62, 21.01, 20.66, 19.75, 19.66, 12.87, 12.00, 11.82.

HRMS (ESI) *m/z* Calcd for $C_{55}H_{68}NaO_4^+$ $[M + Na]^+$: 815.5010, Found: 815.5008.

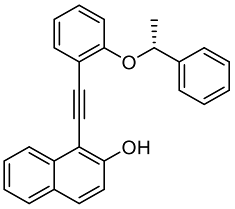

1ac

(*R*)-1-((2-(1-phenylethoxy)phenyl)ethynyl)naphthalen-2-ol (1ac)

Compound **1ac** is an unknown compound, and was synthesized in 56% yield (2 steps, 3 mmol, 612 mg) following the general procedure (**Method D**).

White solid. (R_f = 0.5, PE/EA= 10:1)

^{1}H NMR (400 MHz, $CDCl_3$) δ 8.28 (d, *J* = 8.3 Hz, 1H), 7.78 (t, *J* = 7.7 Hz, 2H), 7.60 – 7.53 (m, 2H), 7.44 – 7.36 (m, 3H), 7.32 (t, *J* = 7.5 Hz, 2H), 7.29 – 7.21 (m, 2H), 7.13 (t, *J* = 7.9 Hz, 1H), 7.07 (s, 1H), 6.91 (t, *J* = 7.5 Hz, 1H), 6.77 (d, *J* = 8.4 Hz, 1H), 5.49 (q, *J* = 6.4 Hz, 1H), 1.81 (d, *J* = 6.4 Hz, 3H).

^{13}C NMR (100 MHz, $CDCl_3$) δ 157.79, 156.64, 142.37, 132.89, 131.70, 130.35, 129.56, 128.78, 128.29, 128.20, 127.67, 127.20, 125.40, 125.07, 123.91, 120.59, 116.32, 113.33, 112.66, 103.42, 98.29, 87.12, 76.81, 24.85.

HRMS (ESI) *m/z* Calcd for $C_{26}H_{20}NaO_2^+$ $[M + Na]^+$: 387.1356, Found: 387.1353.

Optical Rotation: $[\alpha]_D^{25}$= -250.9° (*c* = 0.3, DCM).

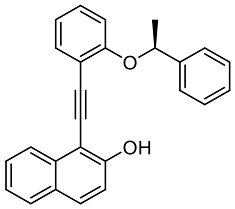

1ad

(*S*)-1-((2-(1-phenylethoxy)phenyl)ethynyl)naphthalen-2-ol (1ad)

Compound **1ad** is an unknown compound, and was synthesized in 67% yield (2 steps, 3 mmol, 733 mg) following the general procedure (**Method D**).

White solid. (R_f = 0.5, PE/EA= 10:1)

^{1}H NMR (400 MHz, $CDCl_3$) δ 8.28 (d, *J* = 8.3 Hz, 1H), 7.77 (t, *J* = 7.8 Hz, 2H), 7.61 – 7.51 (m, 2H), 7.43 – 7.35 (m, 3H), 7.32 (t, *J* = 7.5 Hz, 2H), 7.28 – 7.21 (m, 2H), 7.13 (t, *J* = 7.8 Hz, 1H), 7.05 (s, 1H), 6.90 (t, *J* = 7.5 Hz, 1H), 6.77 (d, *J* = 8.4 Hz, 1H), 5.48 (q, *J* = 6.3 Hz, 1H), 1.81 (d, *J* = 6.4 Hz, 3H).

^{13}C NMR (100 MHz, $CDCl_3$) δ 157.84, 156.65, 142.39, 132.93, 131.74, 130.35, 129.56, 128.78, 128.33, 128.20, 127.67, 127.20, 125.42, 125.09, 123.91, 120.60, 116.33, 113.37, 112.72, 103.44, 98.31, 87.11, 76.85, 24.81.

HRMS (ESI) *m/z* Calcd for $C_{26}H_{20}NaO_2^+$ $[M + Na]^+$: 387.1356, Found: 387.1354.

Optical Rotation: $[\alpha]_D^{25}$= +343.4° (*c* = 0.3, DCM).

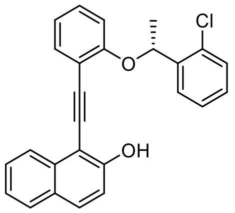

1ae

(*R*)-1-((2-(1-(2-chlorophenyl)ethoxy)phenyl)ethynyl)naphthalen-2-ol (1ae)

Compound **1ae** is an unknown compound, and was synthesized in 70% yield (2 steps, 5 mmol, 1.40 g) following the general procedure (**Method D**).

White solid. (R_f = 0.5, PE/EA= 10:1)

^{1}H NMR (400 MHz, $CDCl_3$) δ 8.29 (d, J = 8.3 Hz, 1H), 7.78 (t, J = 7.4 Hz, 2H), 7.61 – 7.48 (m, 3H), 7.42 – 7.33 (m, 2H), 7.26 (d, J = 8.9 Hz, 1H), 7.21 – 7.11 (m, 3H), 7.06 (s, 1H), 6.92 (t, J = 7.5 Hz, 1H), 6.65 (d, J = 8.4 Hz, 1H), 5.88 (q, J = 6.2 Hz, 1H), 1.81 (d, J = 6.3 Hz, 3H).

^{13}C NMR (100 MHz, $CDCl_3$) δ 157.33, 156.68, 139.75, 132.92, 131.73, 131.44, 130.42, 129.76, 129.45, 128.83, 128.34, 128.23, 127.77, 127.24, 126.60, 125.06, 123.95, 120.80, 116.32, 112.84, 112.54, 103.41, 98.18, 87.25, 73.10, 22.74.

HRMS (ESI) *m/z* Calcd for $C_{26}H_{19}ClNaO_2^+$ $[M + Na]^+$: 421.0966, Found: 421.0965.

Optical Rotation: $[\alpha]_D^{25}$= +281.4° (c = 0.3, DCM).

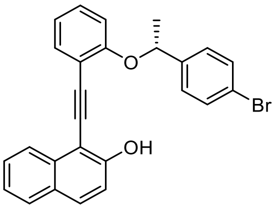

1af

(*R*)-1-((2-(1-(4-bromophenyl)ethoxy)phenyl)ethynyl)naphthalen-2-ol (1af)

Compound **1af** is an unknown compound, and was synthesized in 55% yield (2 steps, 3 mmol, 732 mg) following the general procedure (**Method D**).

Yellow solid. (R_f = 0.5, PE/EA= 10:1)

^{1}H NMR (400 MHz, $CDCl_3$) δ 8.26 (d, J = 8.2 Hz, 1H), 7.78 (t, J = 7.4 Hz, 2H), 7.55 (t, J = 7.8 Hz, 2H), 7.44 (d, J = 8.0 Hz, 2H), 7.38 (t, J = 7.4 Hz, 1H), 7.32 – 7.23 (m, 3H), 7.14 (t, J = 7.8 Hz, 1H), 6.99 (s, 1H), 6.92 (t, J = 7.4 Hz, 1H), 6.71 (d, J = 8.3 Hz, 1H), 5.43 (q, J = 6.1 Hz, 1H), 1.78 (d, J = 6.3 Hz, 3H).

^{13}C NMR (100 MHz, $CDCl_3$) δ 157.49, 156.61, 141.42, 132.90, 131.93, 131.87, 130.45, 129.57, 128.32, 128.23, 127.24, 127.19, 125.02, 123.96, 121.50, 120.89, 116.30, 113.30, 112.81, 103.34, 98.12, 87.22, 76.20, 24.60.

HRMS (ESI) *m/z* Calcd for $C_{26}H_{19}BrNaO_2^+$ $[M + Na]^+$: 465.0461, Found: 465.0460.

Optical Rotation: $[\alpha]_D^{25}$= -171.4° (c = 0.4, DCM).

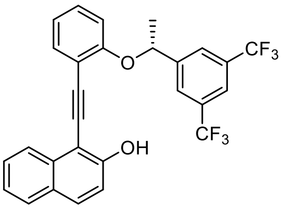

1ag

(*R*)-1-((2-(1-(3,5-bis(trifluoromethyl)phenyl)ethoxy)phenyl)ethynyl)naphthalen-2-ol (1ag)

Compound **1ag** is an unknown compound, and was synthesized in 58% yield (2 steps, 3 mmol, 871 mg) following the general procedure (**Method D**).

White solid. (R_f = 0.4, PE/EA= 10:1)

^{1}H NMR (400 MHz, $CDCl_3$) δ 8.25 (d, J = 8.3 Hz, 1H), 7.88 (s, 2H), 7.84 – 7.75 (m, 3H), 7.65 – 7.59 (m, 1H), 7.55 (t, J = 7.5 Hz, 1H), 7.39 (t, J = 7.4 Hz, 1H), 7.29 – 7.18 (m, 2H), 7.01 (t, J = 7.4 Hz, 1H), 6.84 (s, 1H), 6.72 (d, J = 8.3 Hz, 1H), 5.59 (q, J = 6.4 Hz, 1H), 1.84 (d, J = 6.5 Hz, 3H).

^{13}C NMR (100 MHz, $CDCl_3$) δ 157.10, 156.56, 145.12, 132.96, 132.37, 132.30 (q, J = 33.0 Hz), 130.64, 129.74, 128.35, 128.27, 127.29, 125.74 (d, J = 2.0 Hz), 124.94, 124.00, 123.08 (q, J = 273.0 Hz), 121.98, 121.67, 116.27, 113.43, 113.31, 103.12, 97.62, 87.38, 76.08, 24.32.

HRMS (ESI) *m/z* Calcd for $C_{28}H_{18}F_6NaO_2^+$ [M + Na]$^+$: 523.1103, Found: 523.1102.

Optical Rotation: $[\alpha]_D^{25}$ = -197.7° (c = 0.3, DCM).

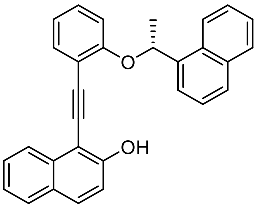

1ah

(*R*)-1-((2-(1-(naphthalen-1-yl)ethoxy)phenyl)ethynyl)naphthalen-2-ol (1ah)

Compound **1ah** is an unknown compound, and was synthesized in 59% yield (2 steps, 3 mmol, 734 mg) following the general procedure (**Method D**).

Yellow solid. (R_f = 0.5, PE/EA= 10:1)

^{1}H NMR (400 MHz, $CDCl_3$) δ 8.32 (d, J = 8.3 Hz, 1H), 8.15 (d, J = 8.3 Hz, 1H), 7.91 (d, J = 8.0 Hz, 1H), 7.78 (q, J = 8.7, 7.8 Hz, 3H), 7.65 (d, J = 7.1 Hz, 1H), 7.63 – 7.49 (m, 4H), 7.44 – 7.35 (m, 2H), 7.28 (d, J = 8.9 Hz, 1H), 7.12 (s, 1H), 7.01 (t, J = 7.9 Hz, 1H), 6.88 (t, J = 7.4 Hz, 1H), 6.59 (d, J = 8.4 Hz, 1H), 6.23 (q, J = 6.3 Hz, 1H), 1.98 (d, J = 6.4 Hz, 3H).

^{13}C NMR (100 MHz, $CDCl_3$) δ 157.69, 156.67, 137.71, 133.82, 132.92, 131.69, 130.39, 129.68, 129.63, 129.25, 128.32, 128.22, 128.03, 127.23, 126.43, 125.95, 125.67, 125.10, 123.94, 122.68, 122.14, 120.58, 116.33, 113.04, 112.47, 103.45, 98.34, 87.17, 73.69, 23.91.

HRMS (ESI) *m/z* Calcd for $C_{30}H_{22}NaO_2^+$ [M + Na]$^+$: 437.1512, Found: 437.1511.

Optical Rotation: $[\alpha]_D^{25}$ = -191.6° (c = 0.4, DCM).

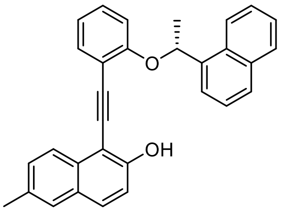

1ai

(*R*)-6-methyl-1-((2-(1-(naphthalen-1-yl)ethoxy)phenyl)ethynyl)naphthalen-2-ol (1ai)

Compound **1ai** is an unknown compound, and was synthesized in 57% yield (2 steps, 3 mmol, 733 mg) following the general procedure (**Method D**).

White solid. (R_f = 0.5, PE/EA= 10:1)

^{1}H NMR (400 MHz, $CDCl_3$) δ 8.21 (d, J = 8.4 Hz, 1H), 8.17 (d, J = 8.3 Hz, 1H), 7.91 (d, J = 8.0 Hz, 1H), 7.77 (d, J = 8.1 Hz, 1H), 7.70 (d, J = 8.9 Hz, 1H), 7.66 (d, J = 7.1 Hz, 1H), 7.62 – 7.56 (m, 3H), 7.53 (t, 1H), 7.45 – 7.35 (m, 2H), 7.25 (s, 1H), 7.02 (t, J = 10.1 Hz, 2H), 6.88 (t, J = 7.3 Hz, 1H), 6.60 (d, J = 8.3 Hz, 1H), 6.24 (q, J = 6.1, 5.6 Hz, 1H), 2.51 (s, 3H), 1.98 (d, J = 6.3 Hz, 3H).

^{13}C NMR (100 MHz, $CDCl_3$) δ 157.71, 156.08, 137.76, 133.84, 133.40, 131.72, 131.09, 129.76, 129.72, 129.56, 129.43, 129.24, 128.53, 128.02, 127.31, 126.42, 125.95, 125.66, 124.95, 122.72, 122.17, 120.56, 116.27, 113.06, 112.59, 103.29, 98.14, 87.34, 73.72, 23.91, 21.39.

HRMS (ESI) *m/z* Calcd for $C_{31}H_{24}NaO_2^+$ $[M + Na]^+$: 451.1669, Found: 451.1664.

Optical Rotation: $[\alpha]_D^{25}$= -39.4° (c = 0.4, DCM).

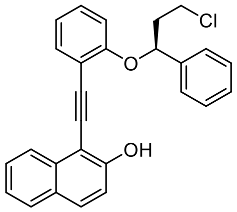

1aj

(*S*)-1-((2-(3-chloro-1-phenylpropoxy)phenyl)ethynyl)naphthalen-2-ol (1aj)

Compound **1aj** is an unknown compound, and was synthesized in 67% yield (2 steps, 3 mmol, 830 mg) following the general procedure (**Method D**).

Yellow solid. (R_f = 0.5, PE/EA= 10:1)

^{1}H NMR (400 MHz, $CDCl_3$) δ 8.26 (d, J = 8.3 Hz, 1H), 7.78 (t, 2H), 7.56 (t, J = 6.8 Hz, 2H), 7.44 (d, J = 7.4 Hz, 2H), 7.39 (d, J = 7.4 Hz, 1H), 7.34 (t, 2H), 7.26 (t, J = 7.8 Hz, 2H), 7.15 (t, J = 7.8 Hz, 1H), 6.99 (s, 1H), 6.92 (t, J = 7.5 Hz, 1H), 6.83 (d, J = 8.4 Hz, 1H), 5.63 (dd, J = 8.4, 4.6 Hz, 1H), 3.95 – 3.85 (m, 1H), 3.69 – 3.58 (m, 1H), 2.76 – 2.64 (m, 1H), 2.38 – 2.25 (m, 1H).

^{13}C NMR (100 MHz, $CDCl_3$) δ 157.70, 156.48, 139.97, 132.90, 131.89, 130.46, 129.75, 128.93, 128.36, 128.26, 128.16, 127.27, 125.87, 124.98, 124.00, 120.96, 116.22, 113.36, 112.63, 103.39, 98.20, 87.06, 77.32, 41.39, 41.13.

HRMS (ESI) *m/z* Calcd for $C_{27}H_{21}ClNaO_2^+$ $[M + Na]^+$: 435.1122, Found: 435.1121.

Optical Rotation: $[\alpha]_D^{25}$= +292.3° (c = 0.4, DCM).

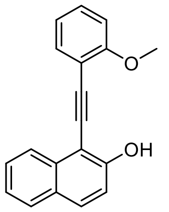

1ak

1-((2-methoxyphenyl)ethynyl)naphthalen-2-ol (1ak)

Compound **1ak** is an unknown compound, and was synthesized in 69% yield (2 steps, 3 mmol, 568 mg) following the general procedure (**Method E**).

White solid. (R_f = 0.6, PE/EA= 10:1)

^{1}H NMR (400 MHz, $CDCl_3$) δ 8.21 (d, J = 8.3 Hz, 1H), 7.81 – 7.74 (m, 2H), 7.60 – 7.51 (m, 2H), 7.41 – 7.32 (m, 2H), 7.30 (s, 1H), 7.25 (d, J = 8.9 Hz, 1H), 7.01 (t, J = 7.5 Hz, 1H), 6.96 (d, J = 8.3 Hz, 1H), 4.00 (s, 3H).

^{13}C NMR (100 MHz, $CDCl_3$) δ 159.23, 156.82, 132.73, 131.26, 130.39, 129.80, 128.23, 128.16, 127.19, 125.04, 123.87, 120.81, 116.36, 112.14, 110.27, 103.27, 97.80, 87.96, 55.85.

HRMS (ESI) *m/z* Calcd for $C_{19}H_{14}NaO_2^+$ [M + Na]$^+$: 297.0886, Found: 297.0884.

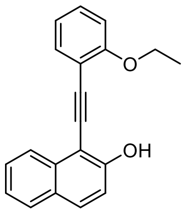

1al

1-((2-ethoxyphenyl)ethynyl)naphthalen-2-ol (1al)

Compound **1al** is an unknown compound, and was synthesized in 72% yield (2 steps, 3 mmol, 623 mg) following the general procedure (**Method E**).

Yellow solid. (R_f = 0.6, PE/EA= 10:1)

^{1}H NMR (400 MHz, $CDCl_3$) δ 8.23 (d, J = 8.3 Hz, 1H), 7.76 (t, J = 8.4 Hz, 2H), 7.61 – 7.50 (m, 2H), 7.36 (t, J = 7.5 Hz, 1H), 7.31 (t, J = 7.9 Hz, 1H), 7.24 (d, J = 8.1 Hz, 1H), 7.07 (s, 1H), 6.98 (t, J = 7.5 Hz, 1H), 6.93 (d, J = 8.3 Hz, 1H), 4.19 (q, J = 6.8 Hz, 2H), 1.58 (t, J = 6.9 Hz, 3H).

^{13}C NMR (100 MHz, $CDCl_3$) δ 158.83, 156.76, 132.83, 131.47, 130.30, 129.76, 128.27, 128.16, 127.14, 125.08, 123.86, 120.56, 116.35, 112.23, 111.15, 103.37, 98.07, 87.43, 64.30, 14.80.

HRMS (ESI) *m/z* Calcd for $C_{20}H_{16}NaO_2^+$ [M + Na]$^+$: 311.1043, Found: 311.1041.

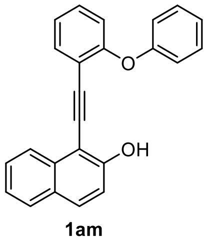

1am

1-((2-phenoxyphenyl)ethynyl)naphthalen-2-ol (1am)

Compound **1am** is an unknown compound, and was synthesized in 68% yield (2 steps, 3 mmol, 686 mg) following the general procedure (**Method E**).

Yellow oil. (R_f = 0.5, PE/EA= 10:1)

^{1}**H NMR** (400 MHz, $CDCl_3$) δ 8.07 (d, *J* = 8.2 Hz, 1H), 7.75 – 7.64 (m, 3H), 7.48 (t, 1H), 7.38 (t, *J* = 7.8 Hz, 2H), 7.36 – 7.28 (m, 2H), 7.20 – 7.12 (m, 3H), 7.10 (d, *J* = 7.9 Hz, 2H), 6.98 (d, *J* = 8.2 Hz, 1H), 6.45 (s, 1H).

^{13}**C NMR** (100 MHz, $CDCl_3$) δ 156.91, 156.58, 156.46, 133.08, 132.47, 130.66, 130.02, 129.91, 128.25, 128.15, 127.28, 124.89, 123.93, 123.76, 123.62, 118.53, 118.40, 116.40, 115.23, 102.82, 97.06, 87.46.

HRMS (ESI) *m/z* Calcd for $C_{24}H_{16}NaO_2^+$ $[M + Na]^+$: 359.1043, Found: 359.1041.

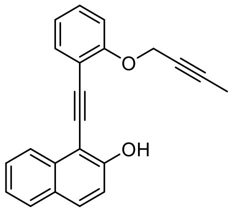

1an

1-((2-(but-2-yn-1-yloxy)phenyl)ethynyl)naphthalen-2-ol (1an)

Compound **1an** is an unknown compound, and was synthesized in 64% yield (2 steps, 3 mmol, 600 mg) following the general procedure (**Method E**).

Yellow solid. (R_f = 0.5, PE/EA= 10:1)

^{1}**H NMR** (400 MHz, $CDCl_3$) δ 8.22 (d, *J* = 8.3 Hz, 1H), 7.76 (t, *J* = 7.6 Hz, 2H), 7.61 – 7.51 (m, 2H), 7.40 – 7.30 (m, 2H), 7.24 (d, 1H), 7.11 (s, 1H), 7.07 (d, *J* = 8.5 Hz, 1H), 7.03 (t, *J* = 7.7 Hz, 1H), 4.82 (s, 2H), 1.89 (s, 3H).

^{13}**C NMR** (100 MHz, $CDCl_3$) δ 157.64, 156.77, 132.86, 131.56, 130.38, 129.60, 128.23, 128.14, 127.17, 125.04, 123.85, 121.23, 116.43, 112.67, 111.88, 103.23, 97.59, 87.67, 84.84, 73.34, 57.16, 3.75.

HRMS (ESI) *m/z* Calcd for $C_{22}H_{16}NaO_2^+$ $[M + Na]^+$: 335.1043, Found: 335.1042.

^{1}H, ^{13}C NMR and HRMS data of compounds (2a-2aj, 3a)

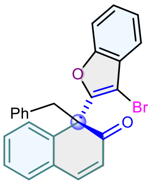

2a

(*R*)-1-benzyl-1-(3-bromobenzofuran-2-yl)naphthalen-2(1H)-one (2a)

Compound **2a** is an unknown compound, and was synthesized in 73% yield (15.7 mg, 0.05 mmol scale) under condition [A].

White solid. (R_f = 0.6, PE/EA = 10:1)

^{1}H NMR (400 MHz, $CDCl_3$) δ 7.57 (d, J = 8.2 Hz, 1H), 7.44 (d, J = 7.7 Hz, 1H), 7.40 – 7.28 (m, 4H), 7.26 (d, J = 8.4 Hz, 1H), 7.16 (d, J = 7.2 Hz, 1H), 7.11 – 7.04 (m, 2H), 6.99 (t, J = 7.5 Hz, 2H), 6.58 (d, J = 7.3 Hz, 2H), 5.99 (d, J = 9.9 Hz, 1H), 4.05 (d, J = 12.5 Hz, 1H), 3.78 (d, J = 12.5 Hz, 1H).

^{13}C NMR (100 MHz, $CDCl_3$) δ 199.04, 155.14, 153.15, 145.03, 142.58, 133.74, 130.54, 130.13, 130.09, 128.75, 128.68, 128.40, 127.65, 127.46, 126.84, 125.93, 125.00, 123.47, 119.55, 111.54, 95.38, 58.08, 47.68.

HRMS (ESI) *m/z* Calcd for $C_{25}H_{18}BrO_2^+$ $[M + H]^+$: 429.0485, Found: 429.0483.

Optical Rotation: $[\alpha]_D^{25}$= -28.0° (c = 0.2, DCM).

HPLC analysis: Chiralcel IB-H (Hexane/i-PrOH = 95:5, flow rate = 1.0 mL/min, wave length = 254 nm), t_R = 7.184 min (major), t_R = 9.286 min (minor), 96% **ee**

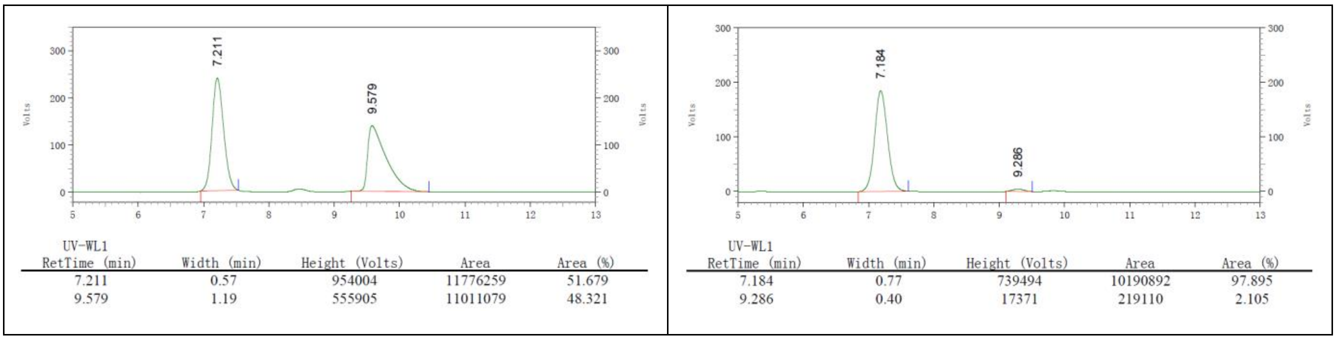

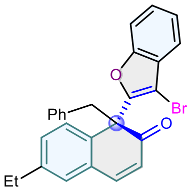

2b

(*R*)-1-benzyl-1-(3-bromobenzofuran-2-yl)-6-ethylnaphthalen-2(1H)-one (2b)

Compound **2b** is an unknown compound, and was synthesized in 72% yield (16.5 mg, 0.05 mmol scale) under condition [A].

White solid. (R_f = 0.6, PE/EA = 10:1)

^{1}H NMR (400 MHz, $CDCl_3$) δ 7.56 (d, J = 8.1 Hz, 1H), 7.44 (d, J = 7.6 Hz, 1H), 7.35 (t, J = 7.6 Hz, 1H), 7.28 (t, J = 7.4 Hz, 1H), 7.18 (q, J = 7.9 Hz, 2H), 7.09 (t, J = 7.2 Hz, 1H), 7.04 (d, J = 9.9 Hz, 1H), 7.01 – 6.95 (m, 3H), 6.58 (d, J = 7.4 Hz, 2H), 5.96 (d, J = 9.9 Hz, 1H), 4.02 (d, J = 12.5 Hz, 1H), 3.76 (d, J = 12.5 Hz, 1H), 2.66 (q, J = 7.5 Hz, 2H), 1.25 (t, J = 7.5 Hz, 3H).

^{13}C NMR (100 MHz, $CDCl_3$) δ 199.23, 155.32, 153.14, 145.30, 143.76, 139.79, 133.95, 130.41, 130.19, 129.79, 128.75, 128.33, 128.10, 127.41, 126.75, 125.81, 124.91, 123.42, 119.53, 111.51, 95.24, 57.87, 47.63, 28.26, 15.36.

HRMS (ESI) *m/z* Calcd for $C_{27}H_{22}BrO_2^+$ $[M + H]^+$: 457.0798, Found: 457.0796.

Optical Rotation: $[\alpha]_D^{25}$= +9.3° (c = 0.3, DCM).

HPLC analysis: Chiralcel IB-H (Hexane/*i*-PrOH = 95:5, flow rate = 1.0 mL/min, wave length = 254 nm), t_R = 6.108 min (major), t_R = 8.293 min (minor), 95% **ee**

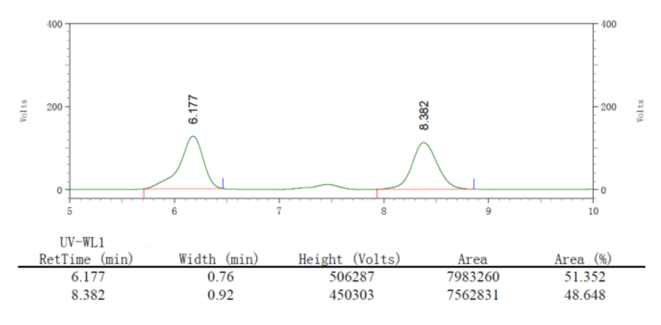

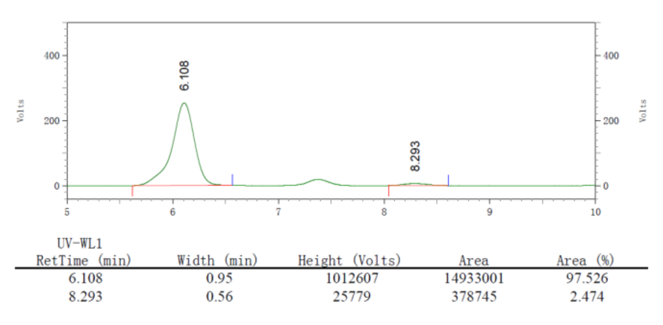

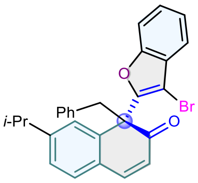

2c

(*R*)-1-benzyl-1-(3-bromobenzofuran-2-yl)-7-isopropylnaphthalen-2(1H)-one (2c)

Compound **2c** is an unknown compound, and was synthesized in 68% yield (16.0 mg, 0.05 mmol scale) under condition [A].

White solid. (R_f = 0.6, PE/EA = 10:1)

^{1}H NMR (400 MHz, $CDCl_3$) δ 7.60 (d, *J* = 8.2 Hz, 1H), 7.46 (d, *J* = 7.6 Hz, 1H), 7.37 (t, *J* = 7.3 Hz, 1H), 7.30 (t, *J* = 7.3 Hz, 1H), 7.17 (d, *J* = 7.8 Hz, 1H), 7.12 – 7.03 (m, 4H), 6.99 (t, *J* = 7.5 Hz, 2H), 6.59 (d, *J* = 7.4 Hz, 2H), 5.96 (d, *J* = 9.9 Hz, 1H), 4.02 (d, *J* = 12.5 Hz, 1H), 3.77 (d, *J* = 12.5 Hz, 1H), 2.85 (hept, *J* = 6.7 Hz, 1H), 1.19 (d, *J* = 6.9 Hz, 6H).

^{13}C NMR (100 MHz, $CDCl_3$) δ 199.12, 155.00, 153.18, 151.53, 145.26, 142.30, 134.00, 130.22, 128.96, 128.80, 128.39, 127.43, 127.11, 126.76, 125.40, 124.92, 124.87, 123.38, 119.61, 111.58, 95.34, 58.25, 47.58, 34.09, 24.05, 23.46.

HRMS (ESI) *m/z* Calcd for $C_{28}H_{24}BrO_2^+$ $[M + H]^+$: 471.0954, Found: 471.0952.

Optical Rotation: $[\alpha]_D^{25}$= -42.7° (*c* = 0.2, DCM).

HPLC analysis: Chiralcel IB-H (Hexane/*i*-PrOH = 95:5, flow rate = 1.0 mL/min, wave length = 254 nm), t_R = 6.125 min (major), t_R = 9.029 min (minor), 91% **ee**

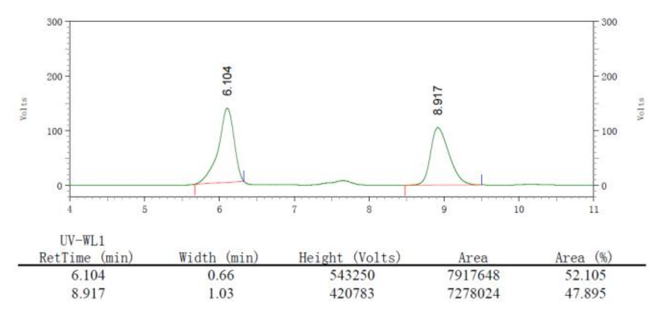

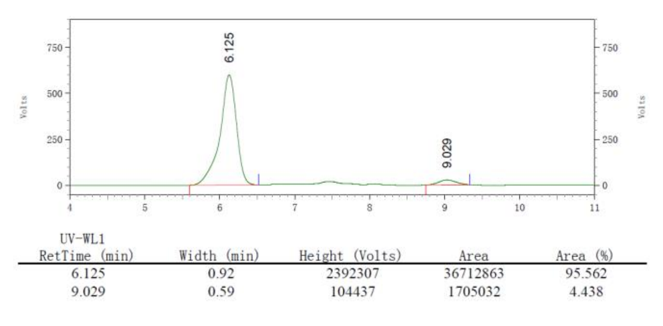

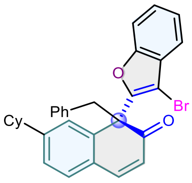

2d

(*R*)-1-benzyl-1-(3-bromobenzofuran-2-yl)-7-cyclohexylnaphthalen-2(1H)-one (2d)

Compound **2d** is an unknown compound, and was synthesized in 69% yield (17.6 mg, 0.05 mmol scale) under condition [A].

White solid. (R_f = 0.6, PE/EA = 10:1)

^{1}H NMR (400 MHz, $CDCl_3$) δ 7.60 (d, *J* = 8.1 Hz, 1H), 7.46 (d, *J* = 7.4 Hz, 1H), 7.38 (t, *J* = 7.4 Hz, 1H), 7.31 (t, 1H), 7.15 (d, *J* = 7.5 Hz, 1H), 7.12 – 7.02 (m, 4H), 6.98 (t, *J* = 7.2 Hz, 2H), 6.58 (d, *J* = 7.2 Hz, 2H), 5.95 (d, *J* = 9.8 Hz, 1H), 4.03 (d, *J* = 12.5 Hz, 1H), 3.75 (d, *J* = 12.5 Hz, 1H), 2.53 – 2.36 (m, 1H), 1.92 – 1.63 (m, 5H), 1.45 – 1.16 (m, 5H).

^{13}C NMR (100 MHz, $CDCl_3$) δ 199.14, 155.06, 153.21, 150.71, 145.31, 142.27, 134.00, 130.22, 128.92, 128.86, 128.42, 127.51, 127.42, 126.74, 125.84, 124.90, 124.86, 123.38, 119.64, 111.61, 95.33, 58.25, 47.65, 44.50, 34.49, 33.79, 26.66, 26.59, 25.91.

HRMS (ESI) *m/z* Calcd for $C_{31}H_{28}BrO_2^+$ $[M + H]^+$: 511.1267, Found: 511.1265.

Optical Rotation: $[\alpha]_D^{25}$= -53.9° (*c* = 0.1, DCM).

HPLC analysis: Chiralcel IB-H (Hexane/*i*-PrOH = 95:5, flow rate = 1.0 mL/min, wave length = 254 nm), t_R = 6.288 min (major), t_R = 8.522 min (minor), 93% **ee**

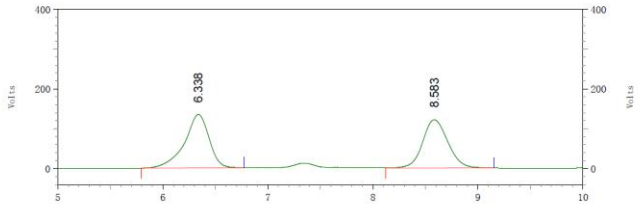

UV-WL1

RetTime (min)	Width (min)	Height (Volts)	Area	Area (%)
6.338	0.99	537592	8750991	51.925
8.583	1.03	485790	8102217	48.075

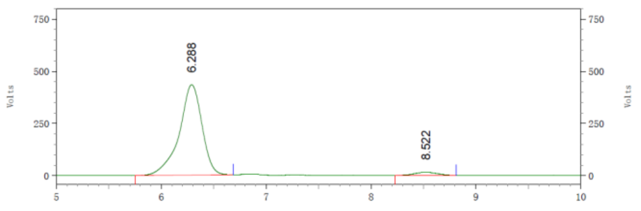

UV-WL1

RetTime (min)	Width (min)	Height (Volts)	Area	Area (%)
6.288	0.93	1733264	26101399	96.620
8.522	0.58	60539	912956	3.380

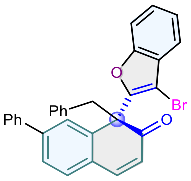

2e

(*R*)-1-benzyl-1-(3-bromobenzofuran-2-yl)-7-phenylnaphthalen-2(1H)-one (2e)

Compound **2e** is an unknown compound, and was synthesized in 65% yield (16.4 mg, 0.05 mmol scale) under condition [A].

White solid. (R_f = 0.6, PE/EA = 10:1)

^{1}H NMR (400 MHz, $CDCl_3$) δ 7.59 (d, J = 8.2 Hz, 1H), 7.54 (d, J = 8.3 Hz, 1H), 7.51 (d, J = 7.4 Hz, 2H), 7.45 (s, 1H), 7.43 (d, J = 4.6 Hz, 1H), 7.40 (d, J = 7.7 Hz, 2H), 7.35 (d, J = 7.6 Hz, 2H), 7.29 (t, 1H), 7.25 (d, J = 5.4 Hz, 1H), 7.14 (d, J = 9.8 Hz, 1H), 7.10 (d, J = 7.3 Hz, 1H), 7.01 (t, J = 7.5 Hz, 2H), 6.65 (d, J = 7.4 Hz, 2H), 6.03 (d, J = 9.9 Hz, 1H), 4.07 (d, J = 12.6 Hz, 1H), 3.85 (d, J = 12.6 Hz, 1H).

^{13}C NMR (100 MHz, $CDCl_3$) δ 198.90, 154.90, 153.22, 144.71, 142.99, 142.84, 139.79, 133.84, 130.29, 129.60, 129.27, 128.88, 128.70, 128.53, 128.03, 127.55, 127.10, 126.89, 126.40, 125.69, 125.03, 123.47, 119.64, 111.60, 95.54, 58.32, 47.69.

HRMS (ESI) *m/z* Calcd for $C_{31}H_{22}BrO_2^+$ $[M + H]^+$: 505.0798, Found: 505.0794.

Optical Rotation: $[\alpha]_D^{25}$= -9.0° (c = 0.3, DCM).

HPLC analysis: Chiralcel IB-H (Hexane/*i*-PrOH = 95:5, flow rate = 1.0 mL/min, wave length = 254 nm), t_R = 7.561 min (major), t_R = 11.013 min (minor), 95% **ee**

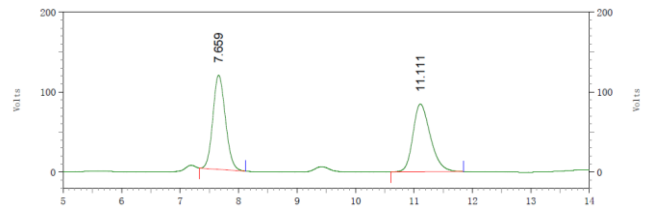

UV-WL1

RetTime (min)	Width (min)	Height (Volts)	Area	Area (%)
7.659	0.78	471548	7027080	49.955
11.111	1.24	339151	7039807	50.045

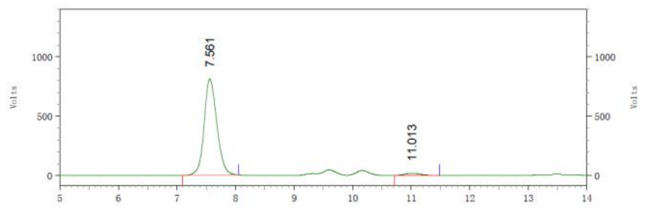

UV-WL1

RetTime (min)	Width (min)	Height (Volts)	Area	Area (%)
7.561	0.96	3234792	48925226	97.460
11.013	0.77	68501	1274879	2.540

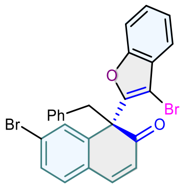

2f

(*R*)-1-benzyl-7-bromo-1-(3-bromobenzofuran-2-yl)naphthalen-2(1H)-one (2f)

Compound **2f** is an unknown compound, and was synthesized in 74% yield (18.8 mg, 0.05 mmol scale) under condition [A].

White solid. (R_f = 0.6, PE/EA = 10:1)

^{1}H NMR (400 MHz, $CDCl_3$) δ 7.59 (d, J = 8.2 Hz, 1H), 7.46 (t, J = 8.2 Hz, 2H), 7.42 – 7.36 (m, 2H), 7.32 (t, J = 7.4 Hz, 1H), 7.12 (t, J = 7.3 Hz, 1H), 7.07 – 6.97 (m, 4H), 6.61 (d, J = 7.3 Hz, 2H), 6.01 (d, J = 9.9 Hz, 1H), 4.04 (d, J = 12.6 Hz, 1H), 3.75 (d, J = 12.6 Hz, 1H).

^{13}C NMR (100 MHz, $CDCl_3$) δ 198.02, 154.27, 153.25, 144.46, 143.79, 133.40, 131.44, 131.04, 130.15, 129.94, 129.52, 128.59, 127.64, 127.06, 126.29, 125.26, 124.75, 123.62, 119.71, 111.67, 95.83, 57.90, 47.67.

HRMS (ESI) *m/z* Calcd for $C_{25}H_{17}Br_2O_2^+$ $[M + H]^+$: 506.9590, Found: 506.9588.

Optical Rotation: $[\alpha]_D^{25}$= +18.1° (c = 0.2, DCM).

HPLC analysis: Chiralcel IB-H (Hexane/i-PrOH = 95:5, flow rate = 1.0 mL/min, wave length = 254 nm), t_R = 7.089 min (major), t_R = 10.835 min (minor), 90% **ee**

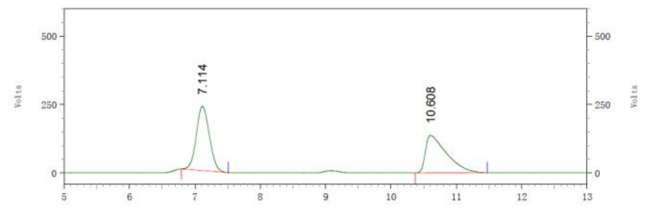

UV-WL1

RetTime (min)	Width (min)	Height (Volts)	Area	Area (%)
7.114	0.72	939079	13036679	51.281
10.608	1.10	549088	12385407	48.719

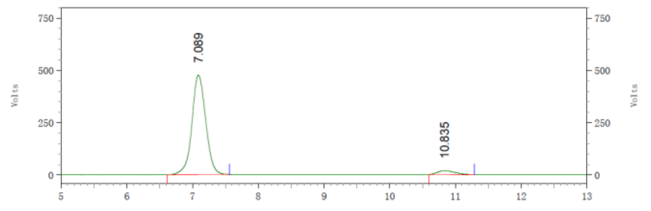

UV-WL1

RetTime (min)	Width (min)	Height (Volts)	Area	Area (%)
7.089	0.95	1906866	27898397	95.144
10.835	0.69	74875	1423888	4.856

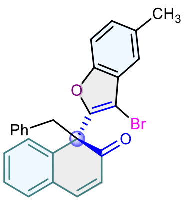

2g

(*R*)-1-benzyl-1-(3-bromo-5-methylbenzofuran-2-yl)naphthalen-2(1H)-one (2g)

Compound **2g** is an unknown compound, and was synthesized in 66% yield (14.6 mg, 0.05 mmol scale) under condition [A].

White solid. (R_f = 0.6, PE/EA = 10:1)

^{1}H NMR (400 MHz, $CDCl_3$) δ 7.44 (d, J = 8.5 Hz, 1H), 7.35 (d, J = 7.3 Hz, 1H), 7.30 (d, J = 6.8 Hz, 1H), 7.26 – 7.20 (m, 2H), 7.19 – 7.13 (m, 2H), 7.07 (t, J = 9.0 Hz, 2H), 6.98 (t, J = 7.5 Hz, 2H), 6.57 (d, J = 7.4 Hz, 2H), 5.98 (d, J = 9.9 Hz, 1H), 4.03 (d, J = 12.5 Hz, 1H), 3.76 (d, J = 12.5 Hz, 1H), 2.45 (s, 3H).

^{13}C NMR (100 MHz, $CDCl_3$) δ 199.05, 155.17, 151.60, 144.96, 142.69, 133.83, 133.15, 130.55, 130.14, 130.06, 128.70, 128.40, 127.60, 127.45, 126.81, 126.26, 125.95, 119.24, 111.08, 95.07, 58.10, 47.67, 21.31.

HRMS (ESI) m/z Calcd for $C_{26}H_{20}BrO_2^+$ $[M + H]^+$: 443.0641, Found: 443.0640.

Optical Rotation: $[\alpha]_D^{25}$= -19.8° (c = 0.3, DCM).

HPLC analysis: Chiralcel IB-H (Hexane/i-PrOH = 95:5, flow rate = 1.0 mL/min, wave length = 254 nm), t_R = 7.046 min (major), t_R = 10.490 min (minor), 90% **ee**

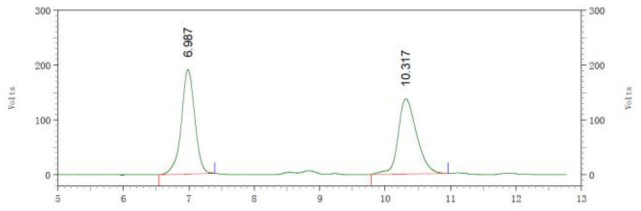

UV-WL1

RetTime (min)	Width (min)	Height (Volts)	Area	Area (%)
6.987	0.86	763413	10863466	50.191
10.317	1.18	548486	10780836	49.809

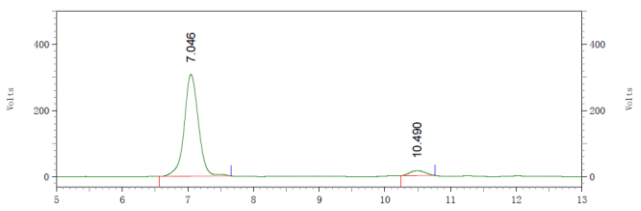

UV-WL1

RetTime (min)	Width (min)	Height (Volts)	Area	Area (%)
7.046	1.09	1229012	18465899	95.045
10.490	0.52	59689	962616	4.955

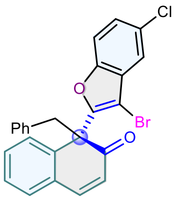

2h

(*R*)-1-benzyl-1-(3-bromo-5-chlorobenzofuran-2-yl)naphthalen-2(1H)-one (2h)

Compound **2h** is an unknown compound, and was synthesized in 77% yield (17.9 mg, 0.05 mmol scale) under condition [A].

White solid. (R_f = 0.6, PE/EA = 10:1)

^{1}H NMR (400 MHz, $CDCl_3$) δ 7.49 (d, J = 8.7 Hz, 1H), 7.44 – 7.40 (m, 1H), 7.38 (d, J = 7.5 Hz, 1H), 7.35 – 7.28 (m, 2H), 7.23 (d, J = 7.6 Hz, 1H), 7.16 (d, J = 7.4 Hz, 1H), 7.13 – 7.03 (m, 2H), 6.99 (t, J = 7.5 Hz, 2H), 6.57 (d, J = 7.5 Hz, 2H), 5.98 (d, J = 9.9 Hz, 1H), 4.02 (d, J = 12.5 Hz, 1H), 3.74 (d, J = 12.5 Hz, 1H).

^{13}C NMR (100 MHz, $CDCl_3$) δ 198.71, 156.79, 151.56, 145.11, 142.22, 133.52, 130.57, 130.17, 130.12, 130.07, 129.33, 128.86, 128.29, 127.81, 127.51, 126.93, 125.89, 125.31, 119.29, 112.64, 94.73, 58.15, 47.62.

HRMS (ESI) *m/z* Calcd for $C_{25}H_{17}BrClO_2^+$ $[M + H]^+$: 463.0095, Found: 463.0092.

Optical Rotation: $[\alpha]_D^{25}$= -25.3° (c = 0.5, DCM).

HPLC analysis: Chiralcel IB-H (Hexane/*i*-PrOH = 95:5, flow rate = 1.0 mL/min, wave length = 254 nm), t_R = 6.716 min (major), t_R = 8.580 min (minor), 91% **ee**

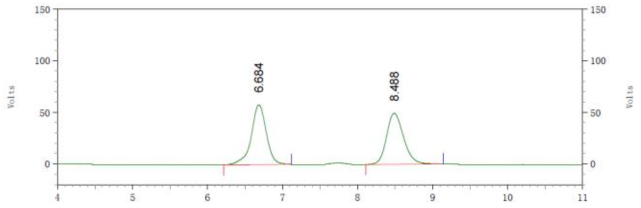

UV-WL1

RetTime (min)	Width (min)	Height (Volts)	Area	Area (%)
6.684	0.91	231690	3222702	50.758
8.488	1.03	197964	3126433	49.242

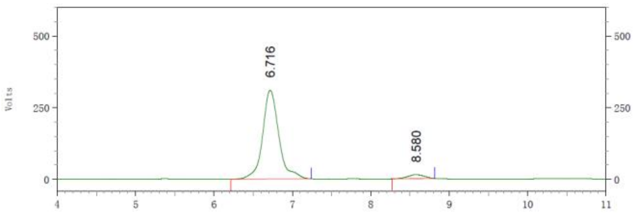

UV-WL1

RetTime (min)	Width (min)	Height (Volts)	Area	Area (%)
6.716	1.02	1239812	17825427	95.631
8.580	0.55	54466	814450	4.369

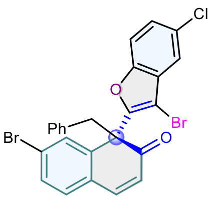

2i

(*R*)-1-benzyl-7-bromo-1-(3-bromo-5-chlorobenzofuran-2-yl)naphthalen-2(1H)-one (2i)

Compound **2i** is an unknown compound, and was synthesized in 67% yield (18.2 mg, 0.05 mmol scale) under condition [A].

White solid. (R_f = 0.6, PE/EA = 10:1)

^{1}H NMR (400 MHz, $CDCl_3$) δ 7.51 (d, *J* = 8.7 Hz, 1H), 7.48 – 7.43 (m, 2H), 7.36 – 7.30 (m, 2H), 7.12 (t, *J* = 7.3 Hz, 1H), 7.04 (d, 2H), 7.01 (d, *J* = 6.7 Hz, 2H), 6.60 (d, *J* = 7.3 Hz, 2H), 6.00 (d, *J* = 9.9 Hz, 1H), 4.01 (d, *J* = 12.6 Hz, 1H), 3.71 (d, *J* = 12.6 Hz, 1H).

^{13}C NMR (100 MHz, $CDCl_3$) δ 197.74, 155.87, 151.61, 144.05, 143.90, 133.12, 131.30, 131.16, 130.09, 130.02, 129.91, 129.48, 127.67, 127.13, 126.20, 125.57, 124.81, 119.41, 112.75, 95.13, 57.91, 47.59.

HRMS (ESI) *m/z* Calcd for $C_{25}H_{16}Br_2ClO_2^+$ $[M + H]^+$: 540.9200, Found: 540.9216.

Optical Rotation: $[\alpha]_D^{25}$= -31.3° (*c* = 0.6, DCM).

HPLC analysis: Chiralcel IB-H (Hexane/*i*-PrOH = 95:5, flow rate = 1.0 mL/min, wave length = 254 nm), t_R = 7.059 min (major), t_R = 9.214 min (minor), 93% **ee**

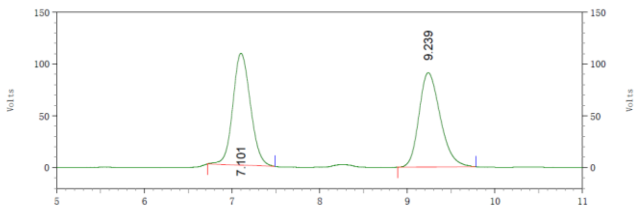

UV-WL1

RetTime (min)	Width (min)	Height (Volts)	Area	Area (%)
7.101	0.77	432427	6030458	49.460
9.239	0.89	364538	6162096	50.540

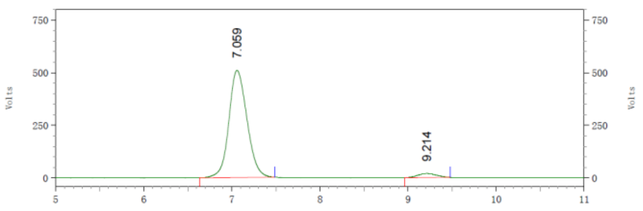

UV-WL1

RetTime (min)	Width (min)	Height (Volts)	Area	Area (%)
7.059	0.86	2038741	29168456	96.251
9.214	0.51	75998	1135992	3.749

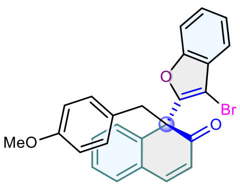

2j

(*R*)-1-(3-bromobenzofuran-2-yl)-1-(4-methoxybenzyl)naphthalen-2(1H)-one (2j)

Compound **2j** is an unknown compound, and was synthesized in 59% yield (13.6 mg, 0.05 mmol scale) under condition [A].

White solid. (R_f = 0.6, PE/EA = 10:1)

^{1}H NMR (400 MHz, $CDCl_3$) δ 7.56 (d, J = 8.1 Hz, 1H), 7.44 (d, J = 7.6 Hz, 1H), 7.40 – 7.33 (m, 2H), 7.29 (q, J = 7.0, 6.6 Hz, 2H), 7.23 (d, J = 8.0 Hz, 1H), 7.17 (d, J = 7.3 Hz, 1H), 7.10 (d, J = 9.9 Hz, 1H), 6.51 (q, J = 8.6 Hz, 4H), 5.99 (d, J = 9.9 Hz, 1H), 4.00 (d, J = 12.7 Hz, 1H), 3.74 (d, 1H), 3.69 (s, 3H).

^{13}C NMR (100 MHz, $CDCl_3$) δ 199.22, 158.44, 155.21, 153.15, 145.11, 142.75, 131.08, 130.57, 130.05, 128.77, 128.68, 128.36, 127.58, 125.88, 125.74, 124.96, 123.44, 119.53, 112.84, 111.51, 95.36, 58.18, 55.01, 46.97.

HRMS (ESI) *m/z* Calcd for $C_{26}H_{20}BrO_3^+$ $[M + H]^+$: 459.0590, Found: 459.0587.

Optical Rotation: $[\alpha]_D^{25}$ = +7.9° (c = 0.2, DCM).

HPLC analysis: Chiralcel IB-H (Hexane/i-PrOH = 95:5, flow rate = 1.0 mL/min, wave length = 254 nm), t_R = 10.377 min (major), t_R = 13.888 min (minor), 95% **ee**

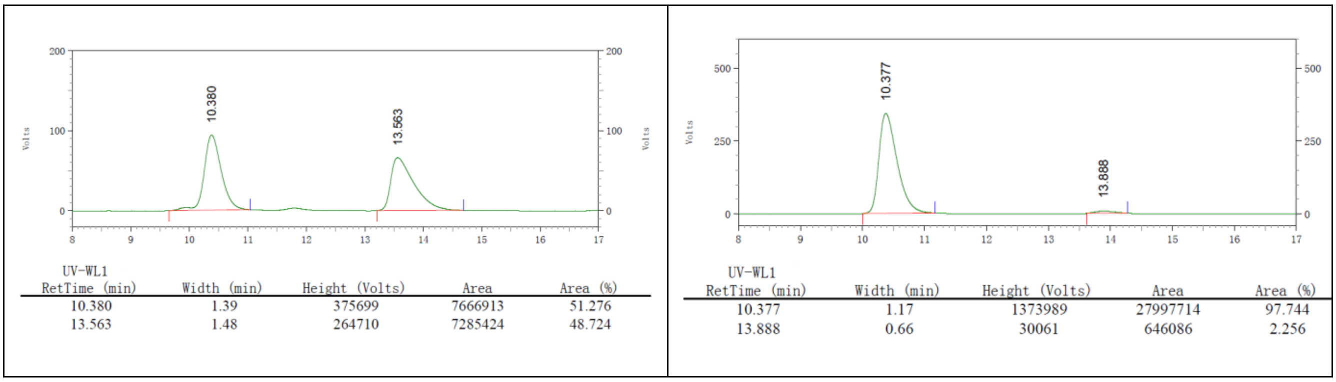

RetTime (min)	Width (min)	Height (Volts)	Area	Area (%)
10.380	1.39	375699	7666913	51.276
13.563	1.48	264710	7285424	48.724

RetTime (min)	Width (min)	Height (Volts)	Area	Area (%)
10.377	1.17	1373989	27997714	97.744
13.888	0.66	30061	646086	2.256

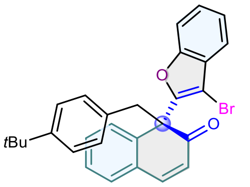

2k

(R)-1-(3-bromobenzofuran-2-yl)-1-(4-(tert-butyl)benzyl)naphthalen-2(1H)-one (2k)

Compound **2k** is an unknown compound, and was synthesized in 76% yield (18.4 mg, 0.05 mmol scale) under condition [A].

White solid. (R_f = 0.6, PE/EA = 10:1)

^{1}H NMR (400 MHz, $CDCl_3$) δ 7.58 (d, J = 8.1 Hz, 1H), 7.44 (d, J = 7.5 Hz, 1H), 7.36 (t, J = 7.4 Hz, 2H), 7.30 (q, J = 6.9 Hz, 2H), 7.23 (d, J = 7.6 Hz, 1H), 7.17 (d, J = 7.3 Hz, 1H), 7.06 (d, J = 9.9 Hz, 1H), 7.00 (d, J = 8.0 Hz, 2H), 6.51 (d, J = 8.0 Hz, 2H), 5.98 (d, J = 9.9 Hz, 1H), 4.00 (d, J = 12.6 Hz, 1H), 3.74 (d, J = 12.6 Hz, 1H), 1.21 (s, 9H).

^{13}C NMR (100 MHz, $CDCl_3$) δ 199.12, 155.24, 153.16, 149.75, 144.87, 142.74, 130.62, 130.61, 129.99, 129.78, 128.71, 128.43, 127.57, 125.90, 124.95, 124.29, 123.44, 119.54, 111.55, 95.38, 58.20, 47.17, 34.28, 31.26.

HRMS (ESI) *m/z* Calcd for $C_{29}H_{26}BrO_2^+$ $[M + H]^+$: 485.1111, Found: 485.1110.

Optical Rotation: $[\alpha]_D^{25}$ = +16.1° (c = 0.3, DCM).

HPLC analysis: Chiralcel IB-H (Hexane/i-PrOH = 95:5, flow rate = 1.0 mL/min, wave length = 254 nm), t_R = 6.052 min (major), t_R = 8.555 min (minor), 93% **ee**

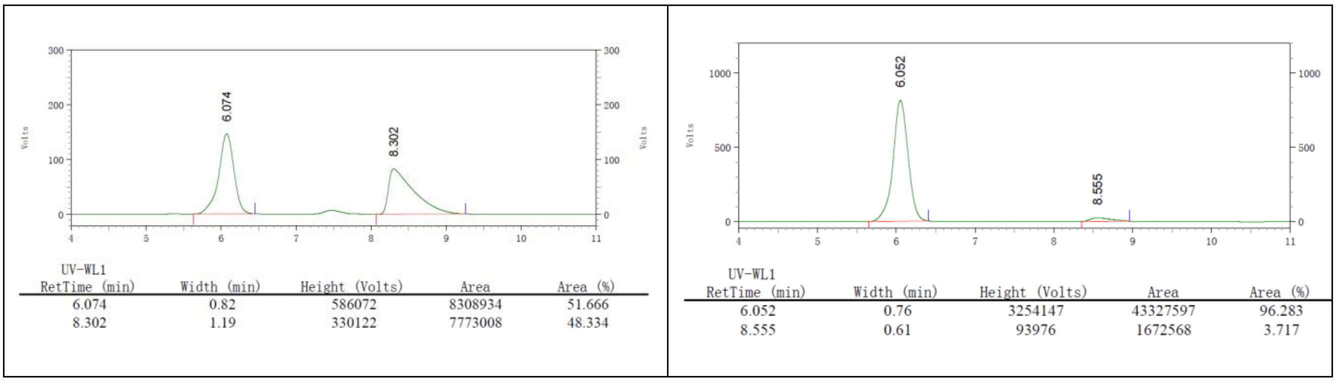

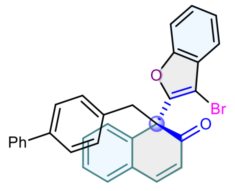

2l

(*R*)-1-([1,1'-biphenyl]-4-ylmethyl)-1-(3-bromobenzofuran-2-yl)naphthalen-2(1H)-one (2l)

Compound **2l** is an unknown compound, and was synthesized in 74% yield (18.7 mg, 0.05 mmol scale) under condition [A].

White solid. (R_f = 0.6, PE/EA = 10:1)

^{1}H NMR (400 MHz, $CDCl_3$) δ 7.58 (d, J = 8.2 Hz, 1H), 7.50 (d, J = 7.4 Hz, 2H), 7.44 (d, J = 7.5 Hz, 1H), 7.37 (q, J = 7.6 Hz, 4H), 7.33 – 7.21 (m, 6H), 7.15 (d, J = 7.3 Hz, 1H), 7.07 (d, J = 9.9 Hz, 1H), 6.65 (d, J = 8.1 Hz, 2H), 6.01 (d, J = 9.9 Hz, 1H), 4.08 (d, J = 12.5 Hz, 1H), 3.81 (d, J = 12.5 Hz, 1H).

^{13}C NMR (100 MHz, $CDCl_3$) δ 198.97, 155.08, 153.15, 145.19, 142.57, 140.48, 139.44, 132.89, 130.53, 130.12, 128.83, 128.66, 128.40, 127.68, 127.16, 126.78, 126.01, 125.94, 125.01, 123.48, 119.56, 111.53, 95.40, 58.09, 47.31.

HRMS (ESI) m/z Calcd for $C_{31}H_{22}BrO_2^+$ $[M + H]^+$: 505.0798, Found: 505.0794.

Optical Rotation: $[\alpha]_D^{25}$ = +54.7° (c = 0.2, DCM).

HPLC analysis: Chiralcel IB-H (Hexane/i-PrOH = 95:5, flow rate = 1.0 mL/min, wave length = 254 nm), t_R = 9.953 min (major), t_R = 12.766 min (minor), 86% **ee**

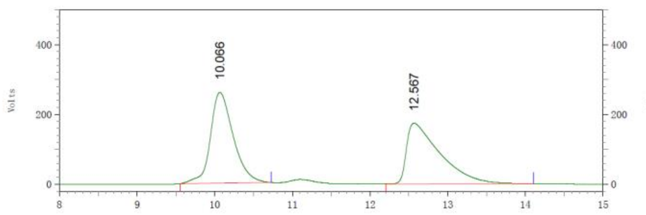

UV-WL1

RetTime (min)	Width (min)	Height (Volts)	Area	Area (%)
10.066	1.17	1042179	21300835	50.459
12.567	1.91	696731	20913446	49.541

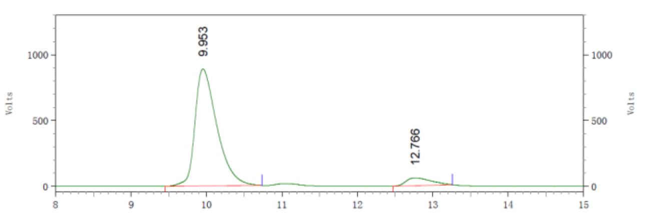

UV-WL1

RetTime (min)	Width (min)	Height (Volts)	Area	Area (%)
9.953	1.29	3555689	71916289	92.993
12.766	0.79	238199	5419286	7.007

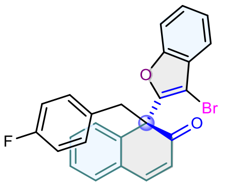

2m

(*R*)-1-(3-bromobenzofuran-2-yl)-1-(4-fluorobenzyl)naphthalen-2(1H)-one (2m)

Compound **2m** is an unknown compound, and was synthesized in 72% yield (16.1 mg, 0.05 mmol scale) under condition [A].

White solid. (R_f = 0.6, PE/EA = 10:1)

^{1}H NMR (400 MHz, $CDCl_3$) δ 7.56 (d, *J* = 2.0 Hz, 1H), 7.44 (d, *J* = 7.5 Hz, 1H), 7.37 (q, *J* = 7.3 Hz, 2H), 7.30 (q, *J* = 7.7 Hz, 2H), 7.25 (d, *J* = 7.4 Hz, 1H), 7.18 (d, *J* = 7.3 Hz, 1H), 7.11 (d, *J* = 9.9 Hz, 1H), 6.68 (t, *J* = 8.5 Hz, 2H), 6.58 – 6.48 (m, 2H), 6.00 (d, *J* = 9.9 Hz, 1H), 4.02 (d, *J* = 12.7 Hz, 1H), 3.74 (d, *J* = 12.7 Hz, 1H).

^{13}C NMR (100 MHz, $CDCl_3$) δ 198.90, 161.92 (d, *J* = 245.3 Hz), 154.91, 153.15, 145.17, 142.42, 131.60 (d, *J* = 8.0 Hz), 130.51, 130.21, 129.48 (d, *J* = 3.0 Hz), 128.87, 128.65, 128.35, 127.78, 125.92, 125.06, 123.51, 119.59, 114.36 (d, *J* = 21.0 Hz), 111.52, 95.45, 57.97, 46.80.

HRMS (ESI) *m/z* Calcd for $C_{25}H_{17}BrFO_2^+$ $[M + H]^+$: 447.0390, Found: 447.0388.

Optical Rotation: $[\alpha]_D^{25}$= -12.0° (*c* = 0.6, DCM).

HPLC analysis: Chiralcel IB-H (Hexane/*i*-PrOH = 95:5, flow rate = 1.0 mL/min, wave length = 254 nm), t_R = 8.073 min (major), t_R = 10.943 min (minor), 95% **ee**

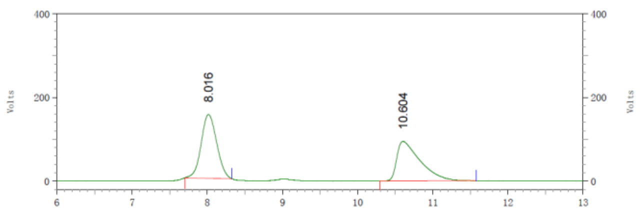

UV-WL1

RetTime (min)	Width (min)	Height (Volts)	Area	Area (%)
8.016	0.63	609555	8762536	51.673
10.604	1.28	378473	8195128	48.327

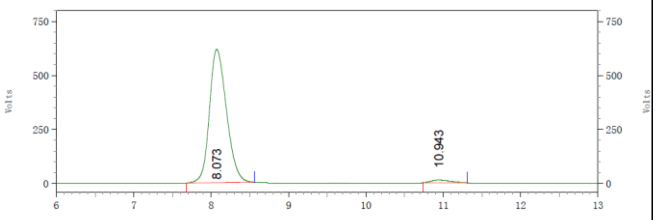

UV-WL1

RetTime (min)	Width (min)	Height (Volts)	Area	Area (%)
8.073	0.88	2473410	37970761	97.717
10.943	0.58	49885	886983	2.283

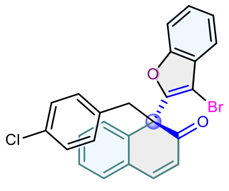

2n

(*R*)-1-(3-bromobenzofuran-2-yl)-1-(4-chlorobenzyl)naphthalen-2(1H)-one (2n)

Compound **2n** is an unknown compound, and was synthesized in 65% yield (15.1 mg, 0.05 mmol scale) under condition [A].

White solid. (R_f = 0.6, PE/EA = 10:1)

^{1}H NMR (400 MHz, $CDCl_3$) δ 7.56 (d, *J* = 8.2 Hz, 1H), 7.44 (d, *J* = 7.6 Hz, 1H), 7.41 – 7.28 (m, 4H), 7.25 (d, *J* = 7.3 Hz, 1H), 7.19 (d, *J* = 7.4 Hz, 1H), 7.12 (d, *J* = 9.9 Hz, 1H), 6.96 (d, *J* = 8.1 Hz, 2H), 6.50 (d, *J* = 8.1 Hz, 2H), 6.01 (d, *J* = 9.9 Hz, 1H), 4.01 (d, *J* = 12.6 Hz, 1H), 3.73 (d, *J* = 12.6 Hz, 1H).

^{13}C NMR (100 MHz, $CDCl_3$) δ 198.71, 154.80, 153.15, 145.29, 142.29, 132.88, 132.32, 131.40, 130.49, 130.24, 128.95, 128.64, 128.35, 127.83, 127.66, 125.94, 125.09, 123.53, 119.60, 111.52, 95.45, 57.85, 46.89.

HRMS (ESI) *m/z* Calcd for $C_{25}H_{17}BrClO_2^+$ [M + H]$^+$: 463.0095, Found: 463.0093.

Optical Rotation: $[\alpha]_D^{25}$ = +68.3° (*c* = 0.3, DCM).

HPLC analysis: Chiralcel IB-H (Hexane/*i*-PrOH = 95:5, flow rate = 1.0 mL/min, wave length = 254 nm), t_R = 10.792 min (major), t_R = 15.847 min (minor), 91% **ee**

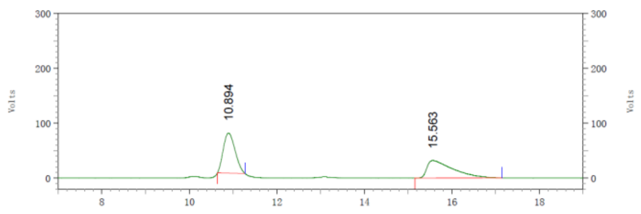

UV-WL1

RetTime (min)	Width (min)	Height (Volts)	Area	Area (%)
10.894	0.63	292512	5458053	50.702
15.563	1.99	128007	5306868	49.298

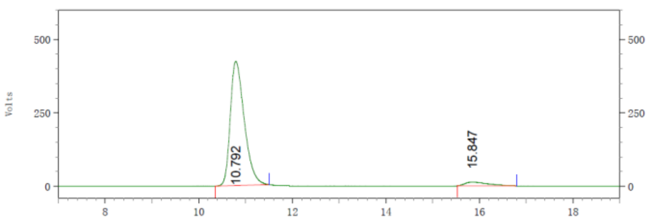

UV-WL1

RetTime (min)	Width (min)	Height (Volts)	Area	Area (%)
10.792	1.15	1694622	35297780	95.381
15.847	1.26	49346	1709447	4.619

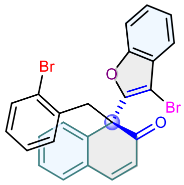

2a-1

(*R*)-1-(3-bromobenzofuran-2-yl)-1-(2-bromobenzyl)naphthalen-2(1H)-one (2a-1)

White solid. (R_f = 0.6, PE/EA= 5:1)

^{1}H NMR (600 MHz, $CDCl_3$) δ 7.60 (d, *J* = 8.2 Hz, 1H), 7.45 (d, *J* = 7.6 Hz, 1H), 7.37 (t, 1H), 7.35 – 7.33 (m, 1H), 7.33 – 7.26 (m, 3H), 7.20 – 7.13 (m, 3H), 6.98 – 6.89 (m, 2H), 6.75 – 6.68 (m, 1H), 6.13 (d, *J* = 9.9 Hz, 1H), 4.26 (d, *J* = 13.4 Hz, 1H), 4.02 (d, *J* = 13.4 Hz, 1H).

^{13}C NMR (100 MHz, $CDCl_3$) δ 198.51, 154.90, 153.12, 145.05, 141.47, 134.18, 132.67, 131.98, 130.44, 130.03, 129.43, 128.81, 128.75, 128.44, 127.86, 126.39, 126.11, 125.89, 125.02, 123.51, 119.57, 111.59, 95.69, 58.15, 44.59.

HRMS (ESI) m/z Calcd for $C_{25}H_{16}Br_2NaO_2^+$ $[M + Na]^+$: 530.9389, Found: 530.9387.

Optical Rotation: $[\alpha]_D^{25}$= -36.0° (*c* = 0.05, DCM).

HPLC analysis: Chiralcel IB-H (Hexane/*i*-PrOH = 95:5, flow rate = 1.0 mL/min, wave length = 254 nm), t_R = 8.738 min (major), t_R = 11.663 min (minor), 96% **ee**

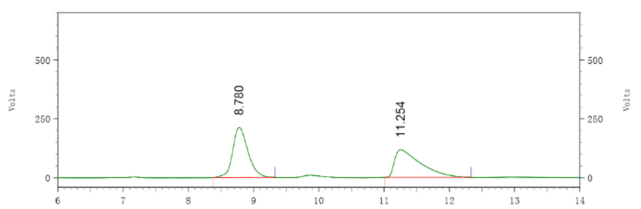

UV-WL1

RetTime (min)	Width (min)	Height (Volts)	Area	Area (%)
8.780	0.95	849320	13889178	51.643
11.254	1.33	466892	13005230	48.357

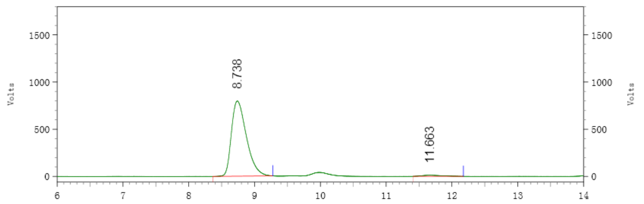

UV-WL1

RetTime (min)	Width (min)	Height (Volts)	Area	Area (%)
8.738	0.91	3187354	50540413	98.108
11.663	0.76	45084	974612	1.892

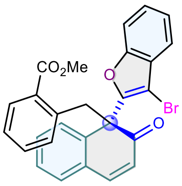

2a-2

methyl (*R*)-2-((1-(3-bromobenzofuran-2-yl)-2-oxo-1,2-dihydronaphthalen-1-yl)methyl)benzoate (2a-2)

White solid. (R_f = 0.6, PE/EA= 5:1)

^{1}H NMR (600 MHz, $CDCl_3$) δ 7.59 (d, *J* = 8.3 Hz, 1H), 7.52 – 7.48 (m, 1H), 7.43 (d, *J* = 7.7 Hz, 1H), 7.37 (t, *J* = 7.8 Hz, 1H), 7.31 – 7.26 (m, 3H), 7.18 – 7.15 (m, 2H), 7.15 – 7.11 (m, 1H), 7.09 (d, *J* = 7.5 Hz, 1H), 7.03 (d, *J* = 9.9 Hz, 1H), 6.91 – 6.85 (m, 1H), 5.96 (d, *J* = 9.9 Hz, 1H), 4.60 (d, *J* = 12.9 Hz, 1H), 4.40 (d, *J* = 12.9 Hz, 1H), 3.67 (s, 3H).

^{13}C NMR (100 MHz, $CDCl_3$) δ 199.42, 168.08, 155.34, 153.12, 144.69, 141.59, 134.24, 132.67, 132.00, 130.52, 130.27, 129.71, 129.67, 129.31, 128.69, 128.65, 127.60, 126.83, 125.91, 124.94, 123.43, 119.47, 111.63, 95.45, 58.44, 51.94, 42.49.

HRMS (ESI) m/z Calcd for $C_{27}H_{19}BrNaO_4^+$ [M + Na]+: 509.0359, Found: 509.0357.

Optical Rotation: $[\alpha]_D^{25}$= -52.0° (*c* = 0.05, DCM).

HPLC analysis: Chiralcel IB-H (Hexane/*i*-PrOH = 95:5, flow rate = 1.0 mL/min, wave length = 254 nm), t_R = 11.903 min (major), t_R = 14.091 min (minor), 96% **ee**

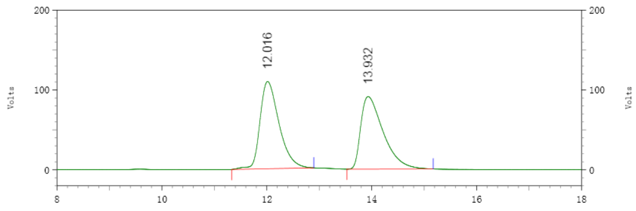

UV-WL1

RetTime (min)	Width (min)	Height (Volts)	Area	Area (%)
12.016	1.57	437200	11060401	50.524
13.932	1.65	363913	10831027	49.476

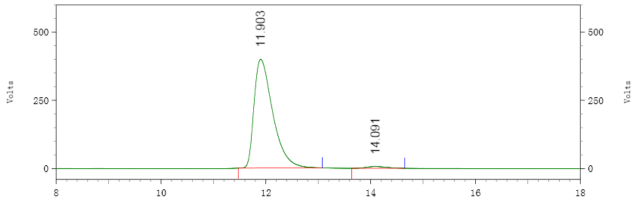

UV-WL1

RetTime (min)	Width (min)	Height (Volts)	Area	Area (%)
11.903	1.60	1594889	39556404	98.247
14.091	1.01	26463	705658	1.753

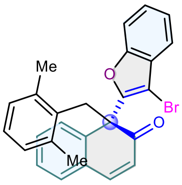

2a-3

(*R*)-1-(3-bromobenzofuran-2-yl)-1-(2,6-dimethylbenzyl)naphthalen-2(1H)-one (2a-3)

White solid. (R_f = 0.6, PE/EA= 10:1)

^{1}H NMR (600 MHz, $CDCl_3$) δ 7.60 (d, *J* = 8.2 Hz, 1H), 7.48 (d, *J* = 7.7 Hz, 1H), 7.37 (t, *J* = 7.7 Hz, 1H), 7.31 (t, *J* = 7.6 Hz, 1H), 7.28 (t, *J* = 7.5 Hz, 1H), 7.23 (d, *J* = 6.0 Hz, 1H), 7.15 (d, *J* = 7.4 Hz, 1H), 7.09 (d, *J* = 7.7 Hz, 1H), 6.97 (d, *J* = 9.9 Hz, 1H), 6.93 (t, *J* = 7.5 Hz, 1H), 6.81 (d, *J* = 7.5 Hz, 2H), 5.85 (d, *J* = 9.9 Hz, 1H), 4.41 (d, *J* = 13.6 Hz, 1H), 3.81 (d, *J* = 13.6 Hz, 1H), 1.77 (s, 6H).

^{13}C NMR (100 MHz, $CDCl_3$) δ 200.05, 156.01, 153.12, 143.87, 142.82, 138.42, 131.75, 130.87, 129.86, 129.44, 128.86, 128.51, 128.28, 127.87, 126.56, 125.50, 124.88, 123.49, 119.55, 111.57, 95.97, 59.31, 39.40, 20.25.

HRMS (ESI) m/z Calcd for $C_{27}H_{21}BrNaO_2^+$ [M + Na]+: 479.0617, Found: 479.0615.

Optical Rotation: $[\alpha]_D^{25}$= -48.8° (*c* = 0.07, DCM).

HPLC analysis: Chiralcel IB-H (Hexane/*i*-PrOH = 98:2, flow rate = 0.5 mL/min, wave length = 254 nm), t_R = 16.167 min (major), t_R = 17.689 min (minor), 87% **ee**

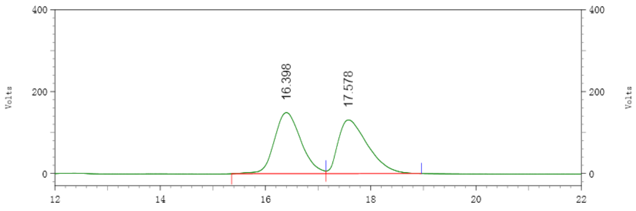

UV-WL1

RetTime (min)	Width (min)	Height (Volts)	Area	Area (%)
16.398	1.79	596272	20973461	50.562
17.578	1.82	524544	20507559	49.438

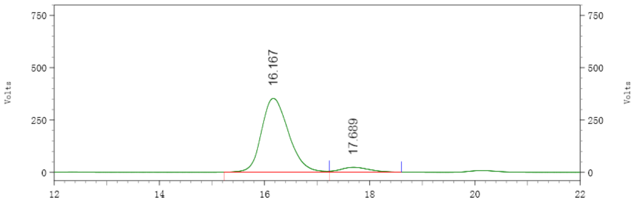

UV-WL1

RetTime (min)	Width (min)	Height (Volts)	Area	Area (%)
16.167	2.00	1409232	50403850	93.573
17.689	1.37	92038	3462109	6.427

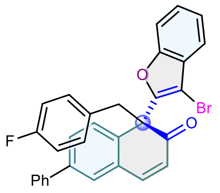

2o

(*R*)-1-(3-bromobenzofuran-2-yl)-1-(4-fluorobenzyl)-6-phenylnaphthalen-2(1H)-one (2o)

Compound **2o** is an unknown compound, and was synthesized in 64% yield (16.7 mg, 0.05 mmol scale) under condition [A].

White solid. (R_f = 0.6, PE/EA = 10:1)

^{1}H NMR (400 MHz, $CDCl_3$) δ 7.64 – 7.59 (m, 3H), 7.58 (d, *J* = 8.3 Hz, 1H), 7.46 (t, *J* = 6.8 Hz, 3H), 7.42 (s, 1H), 7.41 – 7.34 (m, 2H), 7.31 (d, *J* = 8.0 Hz, 2H), 7.17 (d, *J* = 9.9 Hz, 1H), 6.69 (t, *J* = 8.6 Hz, 2H), 6.60 (d, *J* = 5.5 Hz, 1H), 6.58 (d, *J* = 5.9 Hz, 1H), 6.04 (d, *J* = 9.9 Hz, 1H), 4.04 (d, *J* = 12.7 Hz, 1H), 3.78 (d, *J* = 12.7 Hz, 1H).

^{13}C NMR (100 MHz, $CDCl_3$) δ 198.86, 161.94 (d, *J* = 245.6 Hz, 1H), 154.78, 153.18, 145.13, 141.15, 140.60, 139.39, 131.62 (d, *J* = 8.0 Hz, 1H), 130.88, 129.52 (d, *J* = 3.0 Hz, 1H), 128.94, 128.83, 128.66, 127.90, 127.26, 126.89, 126.26, 125.11, 123.55, 119.62, 114.53, 114.32, 111.54, 95.53, 57.82, 46.71.

HRMS (ESI) *m/z* Calcd for $C_{31}H_{21}BrFO_2^+$ $[M + H]^+$: 523.0703, Found: 523.0701.

Optical Rotation: $[\alpha]_D^{25}$ = +56.8° (*c* = 0.4, DCM).

HPLC analysis: Chiralcel IB-H (Hexane/*i*-PrOH = 95:5, flow rate = 1.0 mL/min, wave length = 254 nm), t_R = 9.908 min (major), t_R = 16.725 min (minor), 93% **ee**

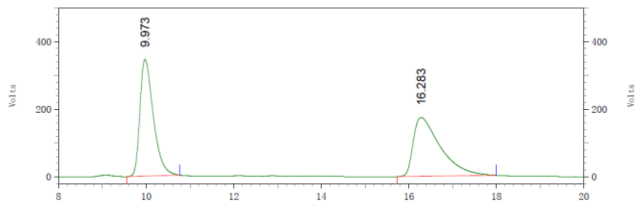

UV-WL1

RetTime (min)	Width (min)	Height (Volts)	Area	Area (%)
9.973	1.21	1384163	29507416	49.564
16.283	2.26	696206	30026073	50.436

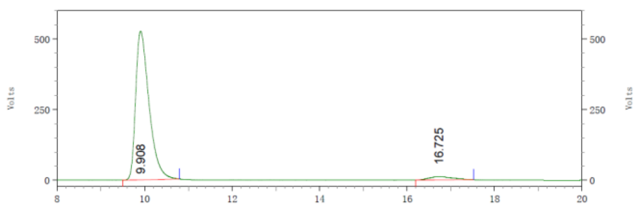

UV-WL1

RetTime (min)	Width (min)	Height (Volts)	Area	Area (%)
9.908	1.30	2105594	44666393	96.313
16.725	1.33	44797	1709673	3.687

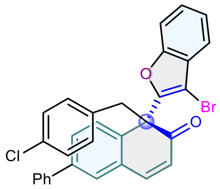

2p

(*R*)-1-(3-bromobenzofuran-2-yl)-1-(4-chlorobenzyl)-6-phenylnaphthalen-2(1H)-one (2p)

Compound **2p** is an unknown compound, and was synthesized in 64% yield (17.3 mg, 0.05 mmol scale) under condition [A].

White solid. (R_f = 0.6, PE/EA = 10:1)

^{1}H NMR (400 MHz, $CDCl_3$) δ 7.63 (d, J = 5.9 Hz, 3H), 7.58 (d, J = 8.2 Hz, 1H), 7.50 – 7.43 (m, 4H), 7.43 – 7.35 (m, 2H), 7.33 (d, J = 8.3 Hz, 2H), 7.21 (d, J = 9.9 Hz, 1H), 6.98 (d, J = 8.3 Hz, 2H), 6.57 (d, J = 8.3 Hz, 2H), 6.06 (d, J = 9.9 Hz, 1H), 4.04 (d, J = 12.6 Hz, 1H), 3.78 (d, J = 12.6 Hz, 1H).

^{13}C NMR (100 MHz, $CDCl_3$) δ 198.66, 154.67, 153.17, 145.24, 140.98, 140.65, 139.35, 132.92, 132.34, 131.44, 130.85, 128.93, 128.81, 128.68, 128.64, 127.91, 127.71, 127.32, 126.88, 126.26, 125.13, 123.56, 119.62, 111.52, 95.52, 57.70, 46.78.

HRMS (ESI) *m/z* Calcd for $C_{31}H_{21}BrClO_2^+$ $[M + H]^+$: 539.0408, Found: 539.0405.

Optical Rotation: $[\alpha]_D^{25}$= +137.0° (c = 0.1, DCM).

HPLC analysis: Chiralcel IB-H (Hexane/i-PrOH = 95:5, flow rate = 1.0 mL/min, wave length = 254 nm), t_R = 10.061 min (major), t_R = 16.959 min (minor), 96% **ee**

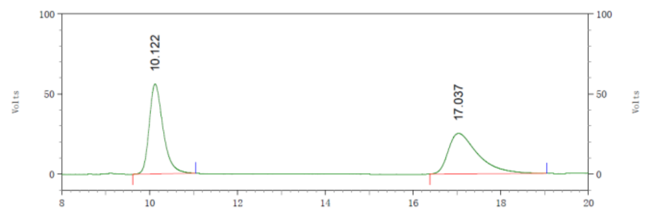

UV-WL1

RetTime (min)	Width (min)	Height (Volts)	Area	Area (%)
10.122	1.43	224628	4929034	50.880
17.037	2.66	101205	4758623	49.120

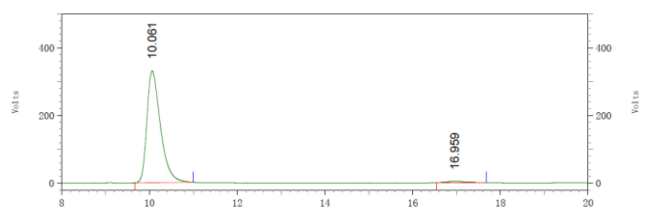

UV-WL1

RetTime (min)	Width (min)	Height (Volts)	Area	Area (%)
10.061	1.33	1322859	28353026	98.192
16.959	1.13	14410	521940	1.808

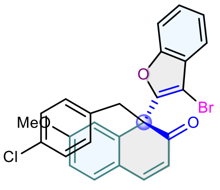

2q

(R)-1-(3-bromobenzofuran-2-yl)-1-(4-chlorobenzyl)-7-methoxynaphthalen-2(1H)-one (2q)

Compound **2q** is an unknown compound, and was synthesized in 62% yield (15.3 mg, 0.05 mmol scale) under condition [A].

White solid. (R_f = 0.6, PE/EA = 10:1)

^{1}H NMR (400 MHz, $CDCl_3$) δ 7.55 (d, J = 8.2 Hz, 1H), 7.45 (d, J = 7.5 Hz, 1H), 7.36 (t, J = 7.2 Hz, 1H), 7.29 (t, J = 7.4 Hz, 1H), 7.13 (d, J = 8.4 Hz, 1H), 7.08 (d, J = 9.9 Hz, 1H), 6.98 (d, J = 8.3 Hz, 2H), 6.84 (dd, J = 8.4, 2.3 Hz, 1H), 6.77 (d, J = 2.0 Hz, 1H), 6.58 (d, J = 8.3 Hz, 2H), 5.90 (d, J = 9.8 Hz, 1H), 3.99 (d, J = 12.6 Hz, 1H), 3.77 (s, 3H), 3.72 (d, J = 12.6 Hz, 1H).

^{13}C NMR (100 MHz, $CDCl_3$) δ 198.50, 161.42, 154.87, 153.14, 145.24, 144.51, 132.85, 132.37, 131.47, 130.54, 128.66, 127.63, 125.06, 123.97, 123.51, 119.62, 115.00, 112.47, 111.51, 95.42, 57.95, 55.45, 47.05.

HRMS (ESI) *m/z* Calcd for $C_{26}H_{19}BrClO_3^+$ $[M + H]^+$: 493.0201, Found: 493.0200.

Optical Rotation: $[\alpha]_D^{25}$= +10.3° (c = 0.2, DCM).

HPLC analysis: Chiralcel IB-H (Hexane/*i*-PrOH = 95:5, flow rate = 1.0 mL/min, wave length = 254 nm), t_R = 8.537 min (major), t_R = 11.303 min (minor), 94% **ee**

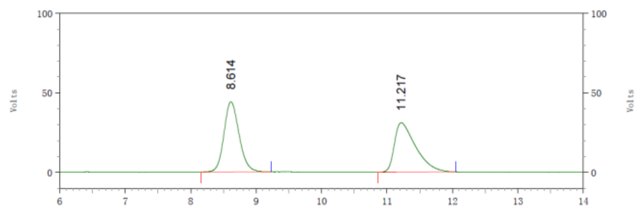

UV-WL1

RetTime (min)	Width (min)	Height (Volts)	Area	Area (%)
8.614	1.07	176775	2858948	50.395
11.217	1.18	124484	2814183	49.605

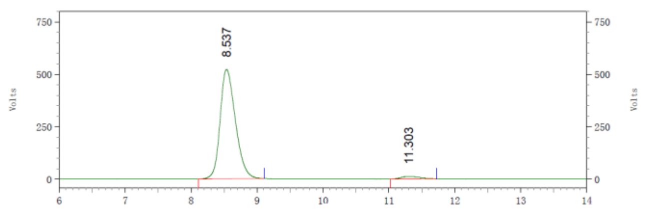

UV-WL1

RetTime (min)	Width (min)	Height (Volts)	Area	Area (%)
8.537	1.00	2091322	33743416	97.154
11.303	0.71	49445	988628	2.846

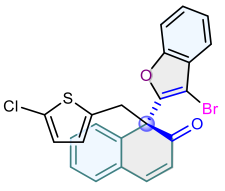

2r

(*R*)-1-(3-bromobenzofuran-2-yl)-1-((5-chlorothiophen-2-yl)methyl)naphthalen-2(1H)-one (2r)

Compound **2r** is an unknown compound, and was synthesized in 80% yield (18.8 mg, 0.05 mmol scale) under condition [A].

White solid. (R_f = 0.6, PE/EA = 10:1)

^{1}H NMR (400 MHz, $CDCl_3$) δ 7.55 (d, *J* = 8.2 Hz, 1H), 7.43 (d, *J* = 7.5 Hz, 1H), 7.40 – 7.26 (m, 6H), 7.24 – 7.19 (m, 1H), 6.53 (d, *J* = 3.7 Hz, 1H), 6.20 (d, *J* = 3.6 Hz, 1H), 6.15 (d, *J* = 9.9 Hz, 1H), 4.20 (d, *J* = 14.1 Hz, 1H), 3.89 (d, *J* = 14.0 Hz, 1H).

^{13}C NMR (100 MHz, $CDCl_3$) δ 198.01, 153.75, 153.06, 145.71, 141.69, 134.52, 130.78, 130.46, 129.32, 128.60, 128.51, 128.32, 128.20, 127.33, 125.76, 125.23, 125.14, 123.57, 119.65, 111.50, 95.51, 57.33, 41.64.

HRMS (ESI) *m/z* Calcd for $C_{23}H_{15}BrClO_2S^+$ $[M + H]^+$: 468.9659, Found: 468.9655.

Optical Rotation: $[\alpha]_D^{25}$= -82.3° (*c* = 0.2, DCM).

HPLC analysis: Chiralcel IB-H (Hexane/*i*-PrOH = 95:5, flow rate = 1.0 mL/min, wave length = 254 nm), t_R = 8.488 min (major), t_R = 11.664 min (minor), 94% **ee**

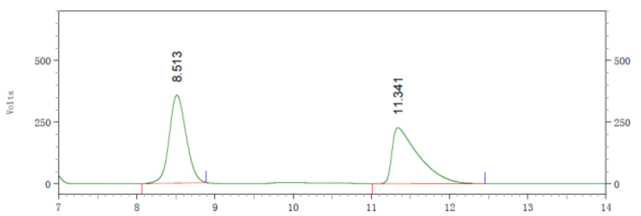

UV-WL1

RetTime (min)	Width (min)	Height (Volts)	Area	Area (%)
8.513	0.83	1425936	21373901	49.760
11.341	1.44	908273	21579951	50.240

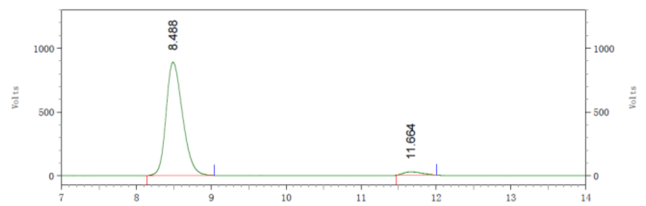

UV-WL1

RetTime (min)	Width (min)	Height (Volts)	Area	Area (%)
8.488	0.90	3555630	54137871	96.929
11.664	0.53	100533	1715520	3.071

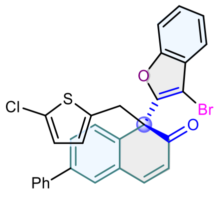

2s

(*R*)-1-(3-bromobenzofuran-2-yl)-1-((5-chlorothiophen-2-yl)methyl)-6-phenylnaphthalen-2(1H)-one (2s)

Compound **2s** is an unknown compound, and was synthesized in 79% yield (21.6 mg, 0.05 mmol scale) under condition [A].

White solid. (R_f = 0.6, PE/EA = 10:1)

^{1}H NMR (400 MHz, $CDCl_3$) δ 7.63 (t, *J* = 8.4 Hz, 3H), 7.56 (d, *J* = 8.8 Hz, 2H), 7.50 – 7.34 (m, 6H), 7.33 – 7.26 (m, 2H), 6.54 (d, *J* = 3.4 Hz, 1H), 6.25 (d, *J* = 3.3 Hz, 1H), 6.19 (d, *J* = 9.9 Hz, 1H), 4.23 (d, *J* = 14.1 Hz, 1H), 3.93 (d, *J* = 14.1 Hz, 1H).

^{13}C NMR (100 MHz, $CDCl_3$) δ 197.98, 153.62, 153.08, 145.68, 141.01, 140.37, 139.39, 134.55, 131.13, 128.94, 128.90, 128.79, 128.61, 128.59, 127.94, 127.74, 127.39, 126.95, 126.10, 125.27, 125.20, 123.60, 119.69, 111.52, 95.58, 57.19, 41.55.

HRMS (ESI) *m/z* Calcd for $C_{29}H_{19}BrClO_2S^+$ $[M + H]^+$: 544.9972, Found: 544.9970.

Optical Rotation: $[\alpha]_D^{25}$= +19.8° (*c* = 0.6, DCM).

HPLC analysis: Chiralcel IB-H (Hexane/*i*-PrOH = 95:5, flow rate = 1.0 mL/min, wave length = 254 nm), t_R = 10.513 min (major), t_R = 15.693 min (minor), 87% **ee**

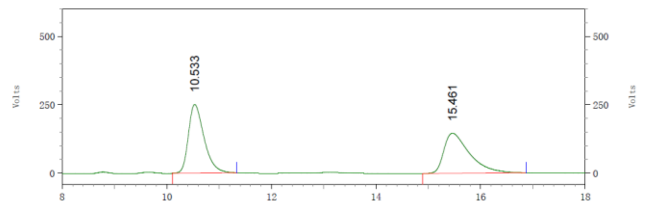

UV-WL1

RetTime (min)	Width (min)	Height (Volts)	Area	Area (%)
10.533	1.23	1005474	20912522	51.104
15.461	1.97	586682	20008622	48.896

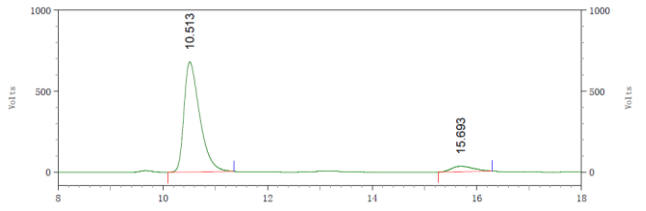

UV-WL1

RetTime (min)	Width (min)	Height (Volts)	Area	Area (%)
10.513	1.26	2716054	56658151	93.431
15.693	1.03	135438	3983486	6.569

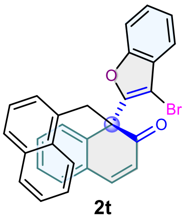

2t

(*R*)-1-(3-bromobenzofuran-2-yl)-1-(naphthalen-1-ylmethyl)naphthalen-2(1H)-one (2t)

Compound **2t** is an unknown compound, and was synthesized in 61% yield (14.6 mg, 0.05 mmol scale) under condition [A].

White solid. (R_f = 0.6, PE/EA = 10:1)

^{1}H NMR (400 MHz, $CDCl_3$) δ 7.72 (dd, 2H), 7.63 (dd, J = 7.9, 2.6 Hz, 2H), 7.49 (d, J = 7.5 Hz, 1H), 7.40 (t, J = 7.4 Hz, 1H), 7.34 (q, J = 6.6 Hz, 3H), 7.29 – 7.22 (m, 3H), 7.06 (t, J = 7.6 Hz, 1H), 7.00 (d, J = 7.3 Hz, 1H), 6.82 (d, J = 9.9 Hz, 1H), 6.60 (d, J = 7.0 Hz, 1H), 5.66 (d, J = 9.9 Hz, 1H), 4.69 (d, J = 13.3 Hz, 1H), 4.13 (d, J = 13.3 Hz, 1H).

^{13}C NMR (100 MHz, $CDCl_3$) δ 199.38, 155.54, 153.25, 144.57, 142.88, 133.26, 132.51, 130.68, 130.32, 130.04, 128.81, 128.64, 128.53, 128.17, 127.73, 127.69, 125.97, 125.59, 125.09, 125.00, 124.41, 123.82, 123.54, 119.61, 111.62, 95.76, 58.75, 42.71.

HRMS (ESI) m/z Calcd for $C_{29}H_{20}BrO_2^+$ $[M + H]^+$: 479.0641, Found: 479.0640.

Optical Rotation: $[\alpha]_D^{25}$= -22.2° (c = 0.2, DCM).

HPLC analysis: Chiralcel IB-H (Hexane/i-PrOH = 95:5, flow rate = 1.0 mL/min, wave length = 254 nm), t_R = 9.797 min (major), t_R = 12.247 min (minor), 94% **ee**

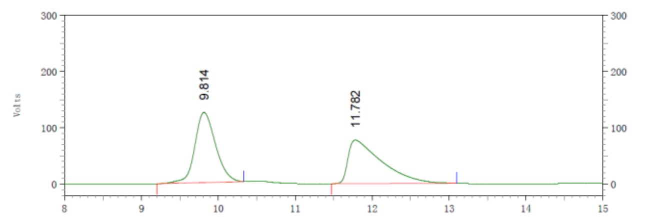

UV-WL1

RetTime (min)	Width (min)	Height (Volts)	Area	Area (%)
9.814	1.14	498941	9385101	49.886
11.782	1.62	309283	9427847	50.114

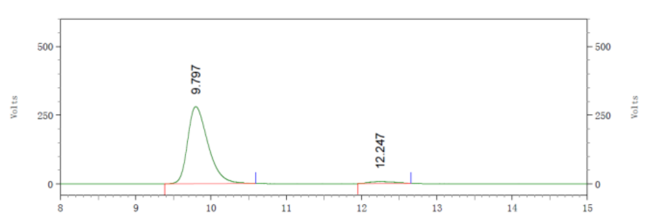

UV-WL1

RetTime (min)	Width (min)	Height (Volts)	Area	Area (%)
9.797	1.20	1121291	21004996	97.101
12.247	0.71	27955	627194	2.899

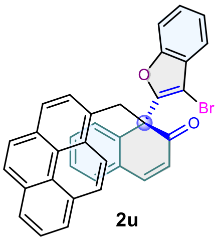

2u

(R)-1-(3-bromobenzofuran-2-yl)-1-(pyren-1-ylmethyl)naphthalen-2(1H)-one (2u)

Compound **2u** is an unknown compound, and was synthesized in 80% yield (22.1 mg, 0.05 mmol scale) under condition [B] (0.0125 M).

Brown solid. (R_f = 0.5, PE/EA = 10:1)

^{1}H NMR (400 MHz, $CDCl_3$) δ 8.11 (dd, J = 11.7, 7.6 Hz, 2H), 7.99 (t, J = 7.6 Hz, 1H), 7.97 – 7.90 (m, 3H), 7.86 (d, J = 9.4 Hz, 1H), 7.76 (d, J = 7.9 Hz, 1H), 7.66 (d, J = 8.2 Hz, 1H), 7.51 (d, J = 7.7 Hz, 1H), 7.45 – 7.31 (m, 4H), 7.29 – 7.22 (m, 1H), 7.11 (d, J = 7.9 Hz, 1H), 6.89 (d, J = 7.5 Hz, 1H), 6.60 (d, J = 9.9 Hz, 1H), 5.61 (d, J = 9.9 Hz, 1H), 4.97 (d, J = 13.2 Hz, 1H), 4.40 (d, J = 13.3 Hz, 1H).

^{13}C NMR (100 MHz, $CDCl_3$) δ 199.45, 155.61, 153.31, 144.67, 142.76, 131.16, 130.63, 130.55, 130.36, 130.13, 130.03, 128.93, 128.84, 128.77, 128.65, 128.26, 127.79, 127.33, 127.18, 126.99, 125.90, 125.75, 125.05, 124.89, 124.77, 124.59, 124.41, 123.70, 123.58, 123.34, 119.64, 111.65, 95.82, 59.01, 43.50.

HRMS (ESI) *m/z* Calcd for $C_{35}H_{22}BrO_2^+$ [M + H]$^+$: 553.0798, Found: 553.0795.

Optical Rotation: $[\alpha]_D^{25}$= +62.7° (*c* = 0.1, DCM).

HPLC analysis: Chiralcel IA-H (Hexane/*i*-PrOH = 90:10, flow rate = 1.0 mL/min, wave length = 254 nm), t_R = 9.898 min (minor), t_R = 14.521 min (major), 98% **ee**

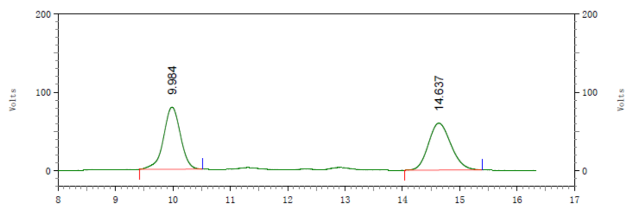

UV-WL1

RetTime (min)	Width (min)	Height (Volts)	Area	Area (%)
9.984	1.10	316848	6486448	49.893
14.637	1.34	238938	6514362	50.107

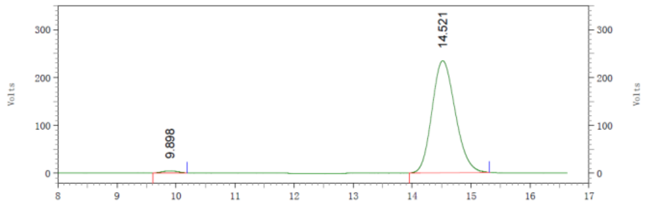

UV-WL1

RetTime (min)	Width (min)	Height (Volts)	Area	Area (%)
9.898	0.58	17015	304299	1.173
14.521	1.37	936205	25627713	98.827

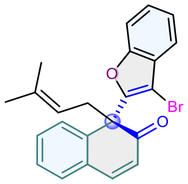

2v

(*R*)-1-(3-bromobenzofuran-2-yl)-1-(3-methylbut-2-en-1-yl)naphthalen-2(1H)-one (2v)

Compound **2v** is an unknown compound, and was synthesized in 78% yield (15.9 mg, 0.05 mmol scale) under condition [B].

Brown solid. (R_f = 0.6, PE/EA = 10:1)

^{1}H NMR (400 MHz, $CDCl_3$) δ 7.53 (dd, 2H), 7.42 (d, *J* = 7.6 Hz, 1H), 7.40 – 7.33 (m, 2H), 7.32 – 7.24 (m, 3H), 7.08 (d, *J* = 7.3 Hz, 1H), 6.30 (d, *J* = 9.9 Hz, 1H), 4.89 (t, *J* = 7.2 Hz, 1H), 3.36 (dd, *J* = 13.4, 8.2 Hz, 1H), 3.15 (dd, *J* = 13.4, 7.2 Hz, 1H), 1.53 (s, 3H), 1.31 (s, 3H).

^{13}C NMR (100 MHz, $CDCl_3$) δ 199.12, 155.09, 153.12, 145.35, 142.94, 136.53, 129.99, 128.87, 128.70, 128.30, 127.34, 125.63, 124.86, 123.36, 119.47, 116.28, 111.51, 95.43, 57.52, 40.10, 25.76, 17.58.

HRMS (ESI) *m/z* Calcd for $C_{23}H_{20}BrO_2^+$ [M + H]$^+$: 407.0641, Found: 407.0640.

Optical Rotation: $[\alpha]_D^{25}$= -34.6° (*c* = 0.1, DCM).

HPLC analysis: Chiralcel IB-H (Hexane/*i*-PrOH = 95:5, flow rate = 1.0 mL/min, wave length = 254 nm), t_R = 6.452 min (major), t_R = 7.498 min (minor), 97% **ee**

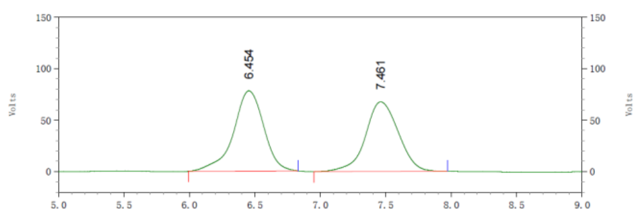

UV-WL1

RetTime (min)	Width (min)	Height (Volts)	Area	Area (%)
6.454	0.84	311886	5041202	51.648
7.461	1.02	270338	4719500	48.352

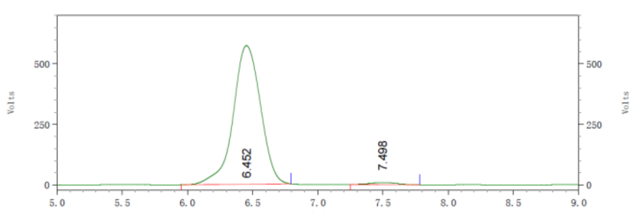

UV-WL1

RetTime (min)	Width (min)	Height (Volts)	Area	Area (%)
6.452	0.84	2288269	33421301	98.528
7.498	0.53	33645	499237	1.472

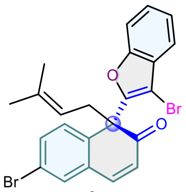

2w

(*R*)-6-bromo-1-(3-bromobenzofuran-2-yl)-1-(3-methylbut-2-en-1-yl)naphthalen-2(1H)-one (2w)

Compound **2w** is an unknown compound, and was synthesized in 73% yield (17.7 mg, 0.05 mmol scale) under condition [B].

Brown solid. (R_f = 0.6, PE/EA = 10:1)

^{1}H NMR (400 MHz, $CDCl_3$) δ 7.53 (d, *J* = 8.3 Hz, 1H), 7.51 (d, 1H), 7.44 (d, *J* = 6.2 Hz, 1H), 7.42 (d, *J* = 3.3 Hz, 1H), 7.40 (dd, *J* = 8.5, 1.8 Hz, 1H), 7.35 (t, *J* = 7.7 Hz, 1H), 7.29 (t, *J* = 7.5 Hz, 1H), 6.96 (d, *J* = 8.3 Hz, 1H), 6.33 (d, *J* = 9.9 Hz, 1H), 4.88 (t, *J* = 7.6 Hz, 1H), 3.35 (dd, *J* = 13.5, 8.2 Hz, 1H), 3.12 (dd, *J* = 13.5, 7.1 Hz, 1H), 1.55 (s, 3H), 1.34 (s, 3H).

^{13}C NMR (100 MHz, $CDCl_3$) δ 198.29, 154.36, 153.16, 143.61, 141.67, 136.99, 132.64, 131.82, 131.32, 129.94, 128.58, 126.75, 125.08, 123.49, 121.06, 119.56, 116.00, 111.55, 95.67, 57.26, 39.87, 25.77, 17.67.

HRMS (ESI) *m/z* Calcd for $C_{23}H_{19}Br_2O_2^+$ $[M + H]^+$: 484.9746, Found: 484.9743.

Optical Rotation: $[\alpha]_D^{25}$= -47.1° (*c* = 0.3, DCM).

HPLC analysis: Chiralcel 2IB-H (Hexane/*i*-PrOH = 95:5, flow rate = 1.0 mL/min, wave length = 254 nm), t_R = 12.122 min (major), t_R = 15.379 min (minor), 95% **ee**

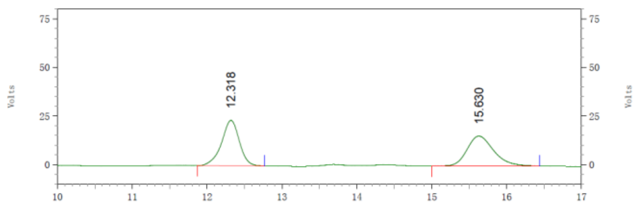

UV-WL1

RetTime (min)	Width (min)	Height (Volts)	Area	Area (%)
12.318	0.90	93598	1555239	50.616
15.630	1.44	61573	1517385	49.384

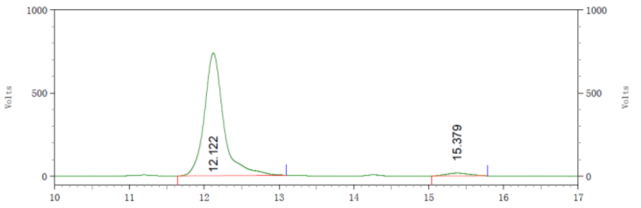

UV-WL1

RetTime (min)	Width (min)	Height (Volts)	Area	Area (%)
12.122	1.46	2947716	52805316	97.404
15.379	0.74	66672	1407513	2.596

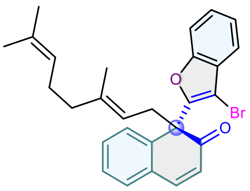

(*R*,*E*)-1-(3-bromobenzofuran-2-yl)-1-(3,7-dimethylocta-2,6-dien-1-yl)naphthalen-2(1H)-one (2x)

Compound **2x** is an unknown compound, and was synthesized in 76% yield (18.1 mg, 0.05 mmol scale) under condition [B].

Brown solid. (R_f = 0.6, PE/EA = 10:1)

^{1}H NMR (400 MHz, $CDCl_3$) δ 7.52 (t, *J* = 8.8 Hz, 2H), 7.42 (d, *J* = 7.6 Hz, 1H), 7.34 (d, *J* = 6.3 Hz, 2H), 7.32 – 7.27 (m, 3H), 7.12 – 7.08 (m, 1H), 6.31 (d, *J* = 9.9 Hz, 1H), 4.96 (t, 1H), 4.89 (t, *J* = 7.5 Hz, 1H), 3.38 (dd, *J* = 13.3, 8.0 Hz, 1H), 3.20 (dd, *J* = 13.3, 7.4 Hz, 1H), 1.90 – 1.78 (m, 4H), 1.64 (s, 3H), 1.54 (s, 3H), 1.31 (s, 3H).

^{13}C NMR (100 MHz, $CDCl_3$) δ 199.09, 155.17, 153.13, 145.42, 142.97, 140.17, 131.35, 130.11, 130.02, 128.83, 128.71, 128.35, 127.35, 125.80, 124.87, 124.08, 123.36, 119.48, 116.03, 111.51, 95.38, 57.46, 40.08, 39.81, 26.58, 25.64, 17.61, 15.98.

HRMS (ESI) *m/z* Calcd for $C_{28}H_{28}BrO_2^+$ $[M + H]^+$: 475.1267, Found: 475.1264.

Optical Rotation: $[\alpha]_D^{25}$= -95.3° (*c* = 0.2, DCM).

HPLC analysis: Chiralcel 2IB-H (Hexane/*i*-PrOH = 95:5, flow rate = 1.0 mL/min, wave length = 254 nm), t_R = 11.878 min (major), t_R = 12.992 min (minor), 97% **ee**

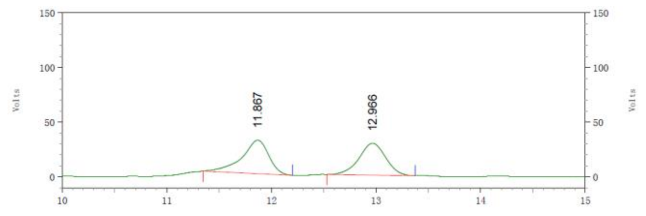

UV-WL1

RetTime (min)	Width (min)	Height (Volts)	Area	Area (%)
11.867	0.85	122533	2188883	51.386
12.966	0.84	116361	2070812	48.614

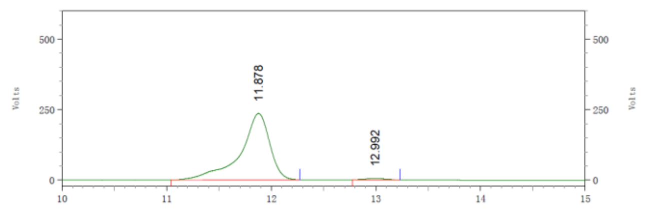

UV-WL1

RetTime (min)	Width (min)	Height (Volts)	Area	Area (%)
11.878	1.23	941531	18384084	98.573
12.992	0.46	18587	266095	1.427

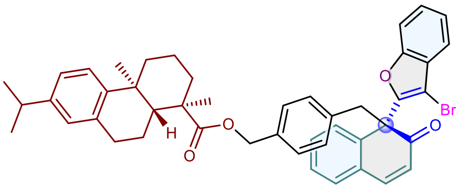

2y

4-(((R)-1-(3-bromobenzofuran-2-yl)-2-oxo-1,2-dihydronaphthalen-1-yl)methyl)benzyl (1R,4aS,10aR)-7-isopropyl-1,4a-dimethyl-1,2,3,4,4a,9,10,10a-octahydrophenanthrene-1-carboxylate (2y)

Compound **2y** is an unknown compound, and was synthesized in 76% yield (28.2 mg, 0.05 mmol scale) under condition [B].

Brown solid. (R_f = 0.5, PE/EA = 10:1)

^{1}H NMR (400 MHz, $CDCl_3$) δ 7.57 (d, J = 8.1 Hz, 1H), 7.44 (d, J = 7.6 Hz, 1H), 7.36 (t, J = 7.2 Hz, 2H), 7.29 (t, J = 7.5 Hz, 2H), 7.27 – 7.24 (m, 1H), 7.15 (t, J = 8.1 Hz, 2H), 7.04 (d, J = 10.0 Hz, 1H), 7.00 (d, J = 8.1 Hz, 1H), 6.95 (d, J = 7.7 Hz, 2H), 6.88 (s, 1H), 6.55 (d, J = 7.7 Hz, 2H), 5.98 (d, J = 9.9 Hz, 1H), 4.98 (q, J = 12.4 Hz, 2H), 4.03 (d, J = 12.5 Hz, 1H), 3.76 (d, J = 12.5 Hz, 1H), 2.88 – 2.71 (m, 3H), 2.29 (d, J = 12.5 Hz, 1H), 2.23 (d, J = 12.2 Hz, 1H), 1.84 – 1.66 (m, 4H), 1.65 – 1.56 (m, 2H), 1.52 – 1.43 (m, 1H), 1.26 (s, 3H), 1.23 (s, 3H), 1.21 (s, 3H), 1.20 (s, 3H).

^{13}C NMR (100 MHz, $CDCl_3$) δ 198.89, 178.18, 155.02, 153.16, 146.77, 145.71, 145.09, 142.48, 134.97, 134.56, 133.66, 130.50, 130.23, 130.14, 128.80, 128.68, 128.39, 127.72, 127.14, 126.87, 125.90, 125.03, 124.17, 123.95, 123.49, 119.57, 111.53, 95.42, 65.95, 58.03, 47.60, 47.33, 44.67, 37.90, 36.93, 36.53, 33.43, 30.03, 25.24, 23.99, 23.95, 21.66, 18.57, 16.54.

HRMS (ESI) *m/z* Calcd for $C_{46}H_{46}BrO_4^+$ $[M + H]^+$: 741.2574, Found: 741.2573.

Optical Rotation: $[\alpha]_D^{25}$ = +25.5° (c = 0.4, DCM).

HPLC analysis: Chiralcel IB-H (Hexane/*i*-PrOH = 95:5, flow rate = 1.0 mL/min, wave length = 254 nm), t_R = 8.946 min (major), t_R = 11.124 min (minor), 98.5:1.5 d. r.

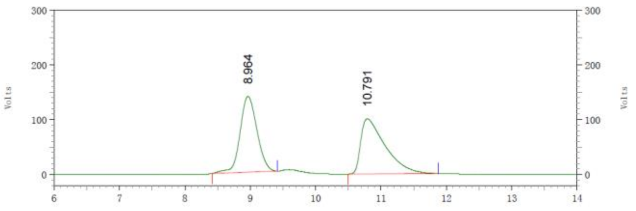

UV-WL1

RetTime (min)	Width (min)	Height (Volts)	Area	Area (%)
8.964	1.00	554547	9985196	48.635
10.791	1.38	403832	10545883	51.365

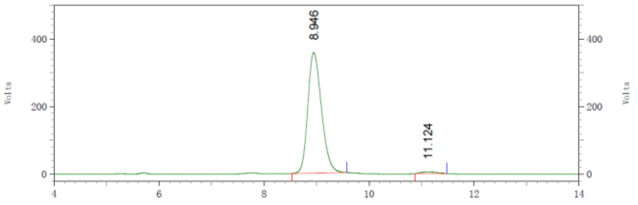

UV-WL1

RetTime (min)	Width (min)	Height (Volts)	Area	Area (%)
8.946	1.04	1429584	25771691	98.441
11.124	0.59	20681	408145	1.559

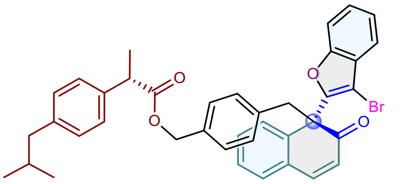

2z

4-(((R)-1-(3-bromobenzofuran-2-yl)-2-oxo-1,2-dihydronaphthalen-1-yl)methyl)benzyl (S)-2-(4-isobutylphenyl)propanoate (2z)

Compound **2z** is an unknown compound, and was synthesized in 74% yield (24.0 mg, 0.05 mmol scale) under condition [C].

Yellow solid. (R_f = 0.4, PE/EA = 5:1)

^{1}H NMR (400 MHz, $CDCl_3$) δ 7.57 (d, J = 8.1 Hz, 1H), 7.44 (d, J = 7.6 Hz, 1H), 7.36 (t, J = 7.3 Hz, 2H), 7.33 – 7.26 (m, 2H), 7.24 (d, J = 7.7 Hz, 1H), 7.16 (t, J = 9.2 Hz, 3H), 7.08 (d, J = 7.8 Hz, 2H), 7.03 (d, J = 9.9 Hz, 1H), 6.85 (d, J = 7.8 Hz, 2H), 6.51 (d, J = 7.8 Hz, 2H), 5.96 (d, J = 9.9 Hz, 1H), 5.07 – 4.89 (m, 2H), 4.01 (d, J = 12.5 Hz, 1H), 3.74 (d, J = 12.6 Hz, 1H), 3.71 (d, J = 7.3 Hz, 1H), 2.45 (d, J = 7.1 Hz, 2H), 1.90 – 1.77 (m, 1H), 1.48 (d, J = 7.1 Hz, 3H), 0.90 (d, J = 6.5 Hz, 6H).

^{13}C NMR (100 MHz, $CDCl_3$) δ 198.85, 174.41, 155.02, 153.14, 145.08, 142.47, 140.54, 137.49, 134.69, 133.58, 130.50, 130.16, 130.11, 129.25, 128.80, 128.67, 128.36, 127.68, 127.15, 126.81, 125.89, 125.02, 123.48, 119.56, 111.52, 95.40, 65.89, 58.01, 47.29, 45.04, 44.99, 30.17, 22.37, 18.40.

HRMS (ESI) *m/z* Calcd for $C_{39}H_{36}BrO_4^+$ $[M + H]^+$: 647.1791, Found: 647.1790.

Optical Rotation: $[\alpha]_D^{25}$ = -13.3° (*c* = 0.2, DCM).

HPLC analysis: Chiralcel IB-H (Hexane/*i*-PrOH = 85:15, flow rate = 1.0 mL/min, wave length = 254 nm), t_R = 6.640 min (major), t_R = 7.623 min (minor), 99:1 d. r.

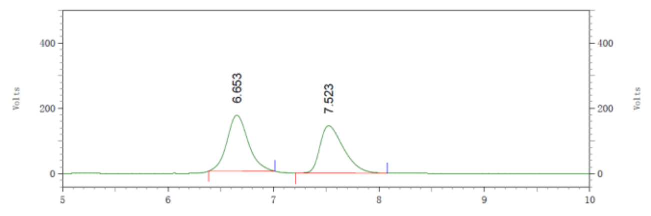

UV-WL1

RetTime (min)	Width (min)	Height (Volts)	Area	Area (%)
6.653	0.63	683984	9586460	51.574
7.523	0.87	580858	9001215	48.426

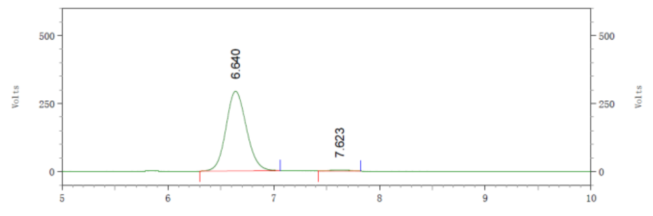

UV-WL1

RetTime (min)	Width (min)	Height (Volts)	Area	Area (%)
6.640	0.76	1173076	15733267	98.847
7.623	0.40	14600	183511	1.153

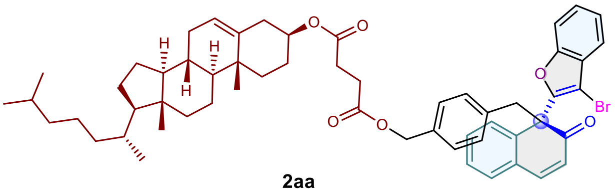

2aa

4-(((R)-1-(3-bromobenzofuran-2-yl)-2-oxo-1,2-dihydronaphthalen-1-yl)methyl)benzyl ((3S,8S,9S,10R,13R,14S,17R)-10,13-dimethyl-17-((R)-6-methylheptan-2-yl)-2,3,4,7,8,9,10,11,12,13,14,15,16,17-tetradecahydro-1H-cyclopenta[a]phenanthren-3-yl) succinate (2aa)

Compound **2aa** is an unknown compound, and was synthesized in 80% yield (37.1 mg, 0.05 mmol scale) under condition [C].

Yellow solid. (R_f = 0.2, PE/EA = 10:1)

^{1}H NMR (400 MHz, $CDCl_3$) δ 7.57 (d, *J* = 8.1 Hz, 1H), 7.45 (d, *J* = 7.6 Hz, 1H), 7.37 (q, *J* = 7.6 Hz, 2H), 7.31 (q, *J* = 7.4 Hz, 2H), 7.27 – 7.24 (m, 1H), 7.19 (d, *J* = 7.4 Hz, 1H), 7.10 (d, *J* = 9.9 Hz, 1H), 6.97 (d, *J* = 7.6 Hz, 2H), 6.57 (d, *J* = 7.7 Hz, 2H), 6.00 (d, *J* = 9.9 Hz, 1H), 5.35 (d, 1H), 5.00 (s, 2H), 4.66 – 4.55 (m, 1H), 4.03 (d, *J* = 12.5 Hz, 1H), 3.76 (d, *J* = 12.5 Hz, 1H), 2.61 (dd, *J* = 12.5, 5.0 Hz, 3H), 2.28 (d, *J* = 7.7 Hz, 2H), 1.99 (t, *J* = 15.5 Hz, 2H), 1.83 (t, *J* = 11.6 Hz, 3H), 1.66 – 1.39 (m, 9H), 1.39 – 1.23 (m, 6H), 1.23 – 1.05 (m, 7H), 1.01 (s, 3H), 0.92 (d, *J* = 6.2 Hz, 3H), 0.87 (d, *J* = 6.5 Hz, 6H), 0.68 (s, 3H).

^{13}C NMR (100 MHz, $CDCl_3$) δ 198.84, 172.09, 171.55, 155.00, 153.15, 145.18, 142.46, 139.50, 134.38, 133.87, 130.51, 130.30, 130.15, 128.87, 128.68, 128.38, 127.73, 127.21, 125.92, 125.03, 123.49, 122.69, 119.58, 111.52, 95.41, 74.36, 66.08, 58.00, 56.64, 56.08, 49.96, 47.28, 42.28, 39.69, 39.49, 37.99, 36.92, 36.54, 36.15, 35.77, 31.87, 31.82, 31.60, 29.40, 29.20, 28.21, 27.99, 27.67, 24.26, 23.80, 22.81, 22.55, 21.00, 19.29, 18.69, 11.84.

HRMS (ESI) *m/z* Calcd for $C_{57}H_{68}BrO_6^+$ [M + H]$^+$: 927.4194, Found: 927.4192.

Optical Rotation: $[\alpha]_D^{25}$= -5.6° (c = 0.4, DCM).

HPLC analysis: Chiralcel 2IB-H (Hexane/*i*-PrOH = 82:18, flow rate = 1.0 mL/min, wave length = 254 nm), t_R = 17.320 min (major), t_R = 18.539 min (minor), 99:1 d. r.

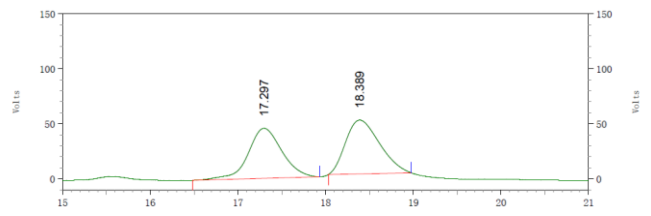

UV-WL1

RetTime (min)	Width (min)	Height (Volts)	Area	Area (%)
17.297	1.45	182061	4856128	47.901
18.389	0.95	195880	5281661	52.099

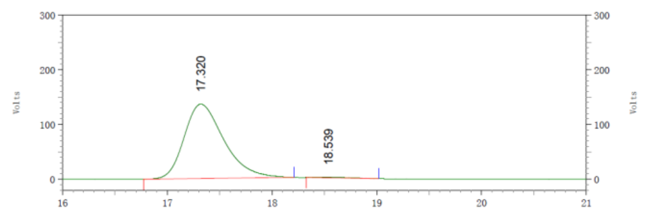

UV-WL1

RetTime (min)	Width (min)	Height (Volts)	Area	Area (%)
17.320	1.44	543770	14361095	99.179
18.539	0.69	5471	118873	0.821

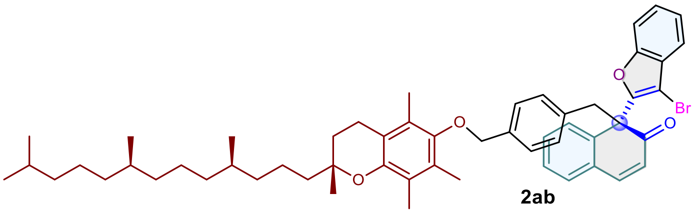

2ab

(R)-1-(3-bromobenzofuran-2-yl)-1-(4-((((R)-2,5,7,8-tetramethyl-2-((4R,8R)-4,8,12-trimethyltridecyl)chroman-6-yl)oxy)methyl)benzyl)naphthalen-2(1H)-one (2ab)

Compound **2ab** is an unknown compound, and was synthesized in 81% yield (35.3 mg, 0.05 mmol scale) under condition [C].

Yellow solid. (R_f = 0.4, PE/EA = 10:1)

^{1}H NMR (400 MHz, $CDCl_3$) δ 7.58 (d, J = 8.1 Hz, 1H), 7.45 (d, J = 7.5 Hz, 1H), 7.41 – 7.24 (m, 5H), 7.19 (d, J = 7.3 Hz, 1H), 7.15 – 7.08 (m, 2H), 6.61 (d, J = 7.7 Hz, 2H), 6.03 (d, J = 9.9 Hz, 1H), 4.57 (s, 2H), 4.07 (d, J = 12.6 Hz, 1H), 3.80 (d, J = 12.6 Hz, 1H), 2.57 (t, J = 6.0 Hz, 2H), 2.14 (s, 3H), 2.12 – 2.04 (m, 5H), 1.87 – 1.72 (m, 2H), 1.67 – 0.99 (m, 28H), 0.92 – 0.78 (m, 10H).

^{13}C NMR (100 MHz, $CDCl_3$) δ 198.93, 155.12, 153.18, 148.13, 147.85, 145.25, 142.59, 136.63, 133.41, 130.57, 130.23, 130.13, 128.89, 128.74, 128.45, 127.85, 127.68, 127.11, 125.99, 125.87, 125.02, 123.48, 122.85, 119.59, 117.54, 111.54, 95.40, 74.81, 74.60, 58.09, 47.33, 40.06, 39.36, 37.47, 37.45, 37.41, 37.28, 32.79, 32.70, 31.61, 31.30, 29.65, 27.97, 24.79, 24.43, 23.86, 22.71, 22.62, 21.02, 20.67, 19.75, 19.65, 12.92, 12.05, 11.81.

HRMS (ESI) *m/z* Calcd for $C_{55}H_{68}BrO_4^+$ [M + H]$^+$: 871.4295, Found: 871.4291.

Optical Rotation: $[\alpha]_D^{25}$= +30.8° (c = 0.4, DCM).

HPLC analysis: Chiralcel IB-H (Hexane/*i*-PrOH = 95:5, flow rate = 1.0 mL/min, wave length = 254 nm), t_R = 7.657 min (major), t_R = 9.101 min (minor), 97.5:2.5 d. r.

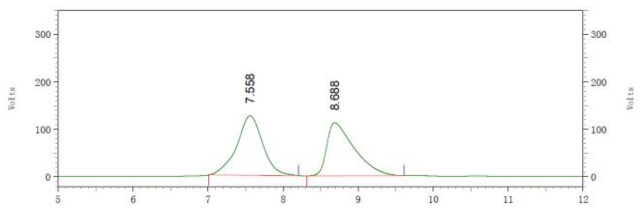

UV-WL1

RetTime (min)	Width (min)	Height (Volts)	Area	Area (%)
7.558	1.20	499619	11988530	51.435
8.688	1.30	446085	11319615	48.565

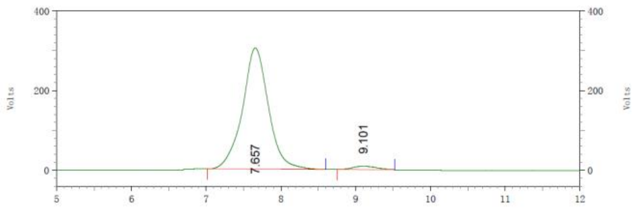

UV-WL1

RetTime (min)	Width (min)	Height (Volts)	Area	Area (%)
7.657	1.58	1218881	29515597	97.542
9.101	0.77	34175	743838	2.458

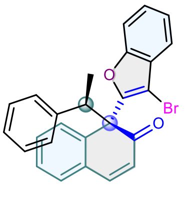

2ac

1-(3-bromobenzofuran-2-yl)-1-((R)-1-phenylethyl)naphthalen-2(1H)-one (2ac)

Compound **2ac** is an unknown compound, and was synthesized in 79% yield (17.5 mg, 0.05 mmol scale) under condition [C].

Yellow solid. (R_f = 0.5, PE/EA = 10:1)

^{1}H NMR (400 MHz, $CDCl_3$) δ 7.59 (d, J = 8.1 Hz, 1H), 7.44 (d, J = 7.5 Hz, 1H), 7.36 (t, J = 7.4 Hz, 1H), 7.29 (d, J = 7.5 Hz, 1H), 7.26 (t, J = 6.6 Hz, 1H), 7.18 – 7.13 (m, 2H), 7.04 (t, J = 7.2 Hz, 1H), 7.00 – 6.90 (m, 4H), 6.70 (d, J = 7.5 Hz, 2H), 6.16 (d, J = 9.9 Hz, 1H), 4.23 (q, J = 6.8 Hz, 1H), 1.64 (d, J = 6.9 Hz, 3H).

^{13}C NMR (100 MHz, $CDCl_3$) δ 196.87, 155.01, 152.90, 144.40, 143.21, 138.97, 129.78, 129.69, 129.31, 128.74, 128.50, 128.05, 127.29, 127.11, 126.77, 124.95, 123.48, 119.63, 111.31, 96.38, 61.10, 50.43, 16.11.

HRMS (ESI) *m/z* Calcd for $C_{26}H_{20}BrO_2^+$ $[M + H]^+$: 443.0641, Found: 443.0640.

Optical Rotation: $[\alpha]_D^{25}$= +193.2° (c = 0.2, DCM).

HPLC analysis: Chiralcel IB-H (Hexane/*i*-PrOH = 95:5, flow rate = 1.0 mL/min, wave length = 254 nm), t_R = 6.053 min (major), t_R = 7.445 min (minor), 98.5:1.5 d. r.

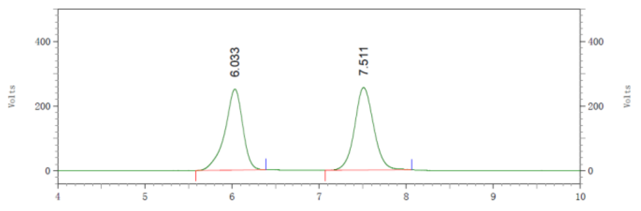

UV-WL1

RetTime (min)	Width (min)	Height (Volts)	Area	Area (%)
6.033	0.81	1001214	14176669	47.863
7.511	0.99	1020215	15442687	52.137

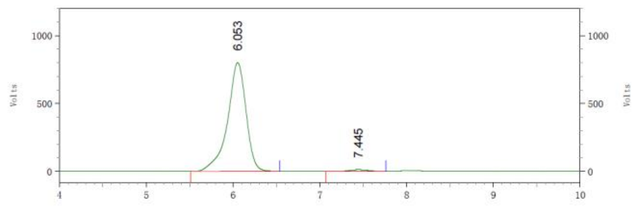

UV-WL1

RetTime (min)	Width (min)	Height (Volts)	Area	Area (%)
6.053	1.03	3207561	47523503	98.473
7.445	0.69	50342	736736	1.527

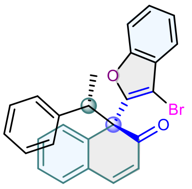

2ad

1-(3-bromobenzofuran-2-yl)-1-((S)-1-phenylethyl)naphthalen-2(1H)-one (2ad)

Compound **2ad** is an unknown compound, and was synthesized in 76% yield (16.8 mg, 0.05 mmol scale) under condition [C].

Yellow solid. (R_f = 0.5, PE/EA = 10:1)

^{1}H NMR (400 MHz, $CDCl_3$) δ 7.61 (d, *J* = 8.0 Hz, 1H), 7.47 – 7.33 (m, 4H), 7.33 – 7.20 (m, 3H), 7.12 (d, *J* = 7.0 Hz, 1H), 7.03 (t, *J* = 7.0 Hz, 2H), 6.90 (d, *J* = 9.8 Hz, 1H), 6.54 (d, *J* = 7.0 Hz, 2H), 5.62 (d, *J* = 9.8 Hz, 1H), 4.36 (q, *J* = 6.7 Hz, 1H), 1.60 (d, *J* = 6.8 Hz, 3H).

^{13}C NMR (100 MHz, $CDCl_3$) δ 199.79, 154.67, 153.23, 144.39, 139.60, 138.46, 131.76, 130.64, 129.17, 128.73, 127.93, 127.52, 127.14, 124.94, 123.39, 119.50, 111.59, 96.33, 62.24, 48.87, 18.71.

HRMS (ESI) *m/z* Calcd for $C_{26}H_{20}BrO_2^+$ $[M + H]^+$: 443.0641, Found: 443.0640.

Optical Rotation: $[\alpha]_D^{25}$= -214.7° (*c* = 0.3, DCM).

HPLC analysis: Chiralcel IB-H (Hexane/*i*-PrOH = 95:5, flow rate = 1.0 mL/min, wave length = 254 nm), t_R = 6.017 min (minor), t_R = 7.332 min (major), 99:1 d. r.

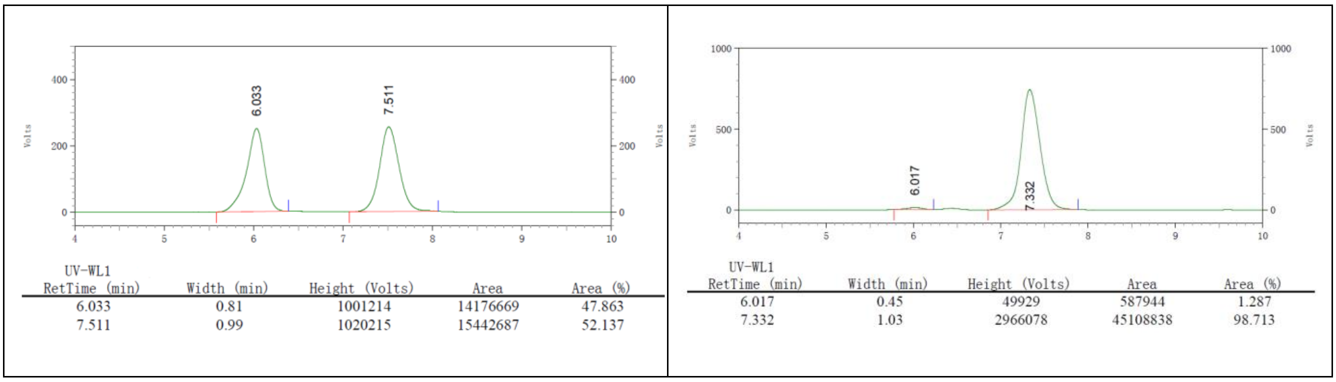

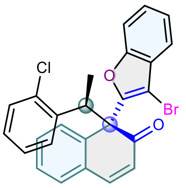

2ae

1-(3-bromobenzofuran-2-yl)-1-((S)-1-(2-chlorophenyl)ethyl)naphthalen-2(1H)-one (2ae)

Compound **2ae** is an unknown compound, and was synthesized in 57% yield (13.6 mg, 0.05 mmol scale) under condition [C].

Yellow solid. (R_f = 0.5, PE/EA = 10:1)

^{1}**H NMR** (400 MHz, CDCl3) δ 7.63 (d, J = 8.1 Hz, 1H), 7.43 (q, J = 8.9, 7.8 Hz, 2H), 7.40 – 7.34 (m, 2H), 7.32 – 7.26 (m, 3H), 7.22 (d, J = 7.6 Hz, 1H), 7.06 (t, J = 7.6 Hz, 1H), 6.99 (d, J = 9.9 Hz, 1H), 6.84 (t, J = 7.4 Hz, 1H), 6.11 (d, J = 7.2 Hz, 1H), 5.77 (d, J = 9.8 Hz, 1H), 5.10 (q, J = 6.8 Hz, 1H), 1.54 (d, 3H).

^{13}**C NMR** (100 MHz, CDCl3) δ 198.25, 154.76, 153.21, 144.27, 138.48, 138.02, 133.76, 132.05, 130.76, 129.44, 129.21, 129.12, 128.85, 128.69, 128.22, 128.15, 125.66, 124.93, 123.39, 119.46, 111.76, 96.45, 61.47, 42.05, 19.36.

HRMS (ESI) *m/z* Calcd for $C_{26}H_{19}BrClO_2^+$ $[M + H]^+$: 477.0251, Found: 477.0250.

Optical Rotation: $[\alpha]_D^{25}$= -138.8° (c = 0.4, DCM).

HPLC analysis: Chiralcel IA-H (Hexane/i-PrOH = 95:5, flow rate = 1.0 mL/min, wave length = 254 nm), t_R = 6.848 min (major), t_R = 7.898 min (minor), 97:3 d. r.

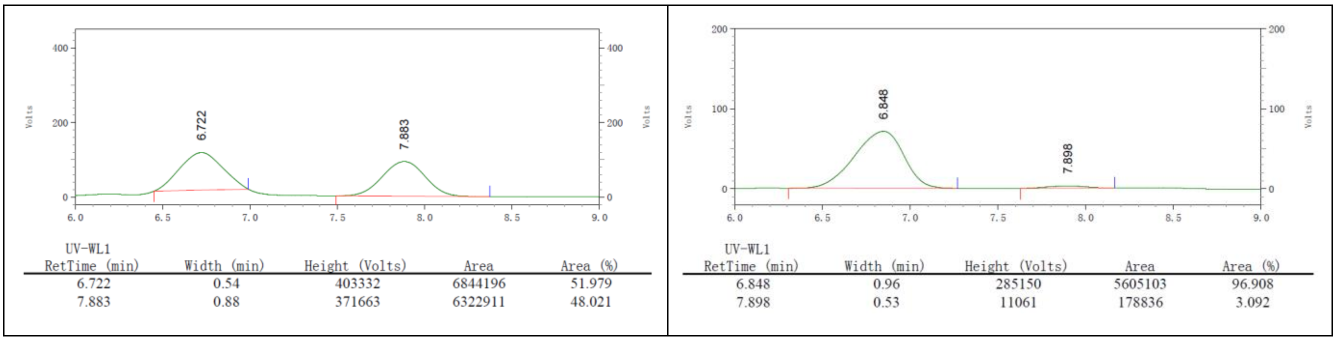

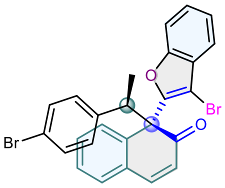

2af

1-(3-bromobenzofuran-2-yl)-1-((R)-1-(4-bromophenyl)ethyl)naphthalen-2(1H)-one (2af)

Compound **2af** is an unknown compound, and was synthesized in 59% yield (15.4 mg, 0.05 mmol scale) under condition [D].

White solid. (R_f = 0.5, PE/EA = 10:1)

^{1}**H NMR** (400 MHz, $CDCl_3$) δ 7.58 (d, J = 8.1 Hz, 1H), 7.44 (d, J = 7.6 Hz, 1H), 7.37 (t, J = 7.7 Hz, 1H), 7.29 (t, 2H), 7.19 (t, J = 7.4 Hz, 1H), 7.15 (d, J = 7.7 Hz, 1H), 7.10 – 7.01 (m, 4H), 6.57 (d, J = 8.3 Hz, 2H), 6.17 (d, J = 9.9 Hz, 1H), 4.19 (q, J = 6.8 Hz, 1H), 1.60 (d, J = 6.9 Hz, 3H).

^{13}**C NMR** (100 MHz, $CDCl_3$) δ 196.62, 154.65, 152.90, 144.70, 142.92, 138.17, 130.95, 129.90, 129.87, 129.75, 128.78, 128.69, 128.03, 127.54, 127.05, 125.05, 123.56, 120.79, 119.70, 111.30, 96.48, 60.79, 49.82, 16.04.

HRMS (ESI) *m/z* Calcd for $C_{26}H_{19}Br_2O_2^+$ $[M + H]^+$: 520.9746, Found: 520.9741.

Optical Rotation: $[\alpha]_D^{25}$= +178.0° (c = 0.4, DCM).

HPLC analysis: Chiralcel IB-H (Hexane/*i*-PrOH = 95:5, flow rate = 1.0 mL/min, wave length = 254 nm), t_R = 6.611 min (major), t_R = 7.279 min (minor), 99:1 d. r.

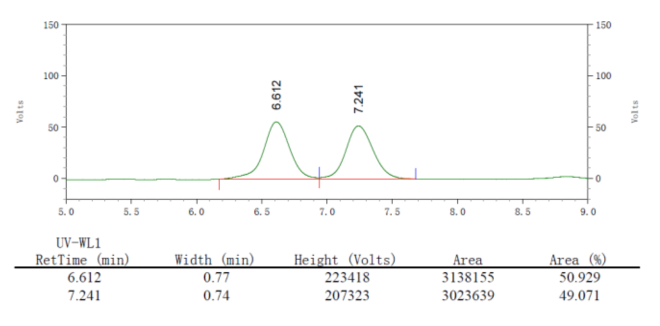

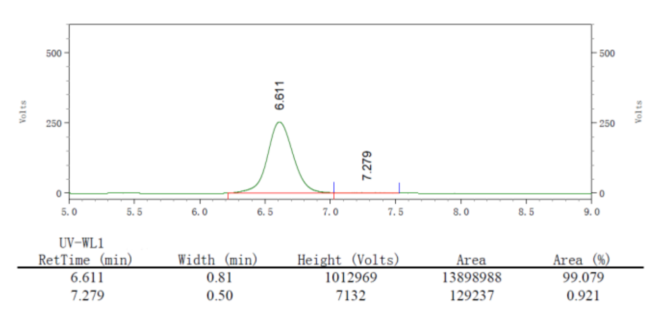

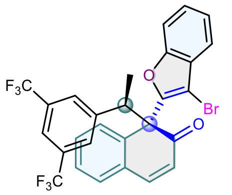

2ag

1-((R)-1-(3,5-bis(trifluoromethyl)phenyl)ethyl)-1-(3-bromobenzofuran-2-yl)naphthalen-2(1H)-one (2ag)

Compound **2ag** is an unknown compound, and was synthesized in 65% yield (18.8 mg, 0.05 mmol scale) under condition [D].

White solid. (R_f = 0.7, PE/EA = 10:1)

^{1}H NMR (400 MHz, $CDCl_3$) δ 7.60 (d, *J* = 8.2 Hz, 2H), 7.45 (d, *J* = 7.6 Hz, 1H), 7.39 (t, *J* = 7.6 Hz, 1H), 7.31 (t, *J* = 7.5 Hz, 2H), 7.24 (d, *J* = 6.8 Hz, 1H), 7.16 (s, 2H), 7.09 (d, *J* = 7.7 Hz, 1H), 7.02 (d, *J* = 9.3 Hz, 2H), 6.21 (d, *J* = 9.9 Hz, 1H), 4.36 (q, *J* = 6.8 Hz, 1H), 1.65 (d, *J* = 6.9 Hz, 3H).

^{13}C NMR (100 MHz, $CDCl_3$) δ 196.06, 154.05, 152.97, 144.90, 142.09, 141.76, 130.23, 129.87 (q, *J* = 33.2 Hz), 129.63, 129.57, 128.99, 128.59, 128.19, 128.08, 126.80, 125.28, 123.73, 123.08 (q, *J* = 273.0 Hz), 120.71, 119.77, 111.34, 96.77, 60.79, 49.73, 15.63.

HRMS (ESI) *m/z* Calcd for $C_{28}H_{18}BrF_6O_2^+$ $[M + H]^+$: 579.0389, Found: 579.0387.

Optical Rotation: $[\alpha]_D^{25}$ = +123.4° (*c* = 0.2, DCM).

HPLC analysis: Chiralcel IC-H (Hexane/*i*-PrOH = 98:2, flow rate = 1.0 mL/min, wave length = 254 nm), t_R = 9.332 min (minor), t_R = 12.308 min (major), 99:1 d. r.

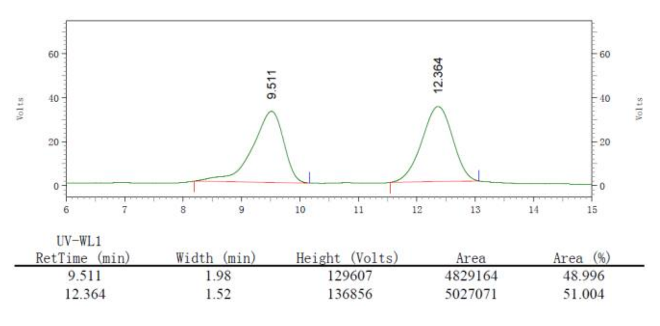

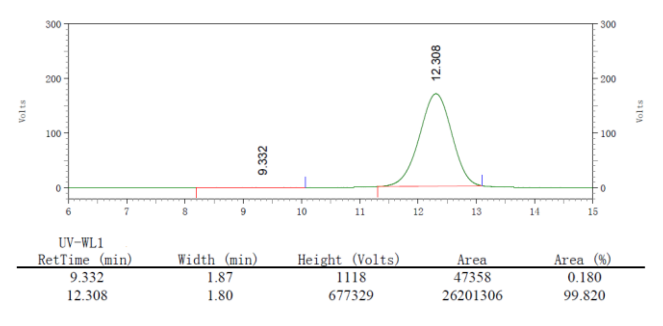

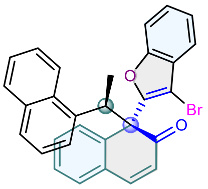

2ah

1-(3-bromobenzofuran-2-yl)-1-((R)-1-(naphthalen-1-yl)ethyl)naphthalen-2(1H)-one (2ah)

Compound **2ah** is an unknown compound, and was synthesized in 63% yield (15.5 mg, 0.05 mmol scale) under condition [A].

White solid. (R_f = 0.6, PE/EA = 10:1)

^{1}H NMR (400 MHz, $CDCl_3$) δ 7.92 (d, *J* = 8.0 Hz, 1H), 7.64 – 7.56 (m, 3H), 7.46 (d, *J* = 7.5 Hz, 1H), 7.38 (t, *J* = 7.6 Hz, 1H), 7.32 (d, *J* = 7.3 Hz, 2H), 7.23 (dd, *J* = 15.6, 7.4 Hz, 4H), 7.01 (t, *J* = 7.5 Hz, 1H), 6.91 (d, *J* = 9.8 Hz, 1H), 6.80 (t, *J* = 7.2 Hz, 1H), 6.64 (d, *J* = 7.3 Hz, 1H), 6.29 (d, *J* = 9.8 Hz, 1H), 5.33 (q, *J* = 6.6 Hz, 1H), 1.74 (d, *J* = 6.4 Hz, 3H).

^{13}C NMR (100 MHz, $CDCl_3$) δ 197.69, 155.73, 152.91, 144.46, 142.49, 136.04, 132.84, 131.49, 129.60, 129.29, 128.82, 128.77, 128.16, 128.10, 127.67, 127.42, 127.06, 126.80, 125.00, 124.83, 124.59, 123.95, 123.55, 123.26, 119.70, 111.43, 96.37, 61.20, 42.47, 18.03.

HRMS (ESI) *m/z* Calcd for $C_{30}H_{22}BrO_2^+$ $[M + H]^+$: 493.0798, Found: 493.0797

Optical Rotation: $[\alpha]_D^{25}$= +35.8° (*c* = 0.2, DCM).

HPLC analysis: Chiralcel IA-H (Hexane/*i*-PrOH = 95:5, flow rate = 1.0 mL/min, wave length = 254 nm), t_R = 7.629 min (minor), t_R = 8.244 min (major), >99:1 d. r.

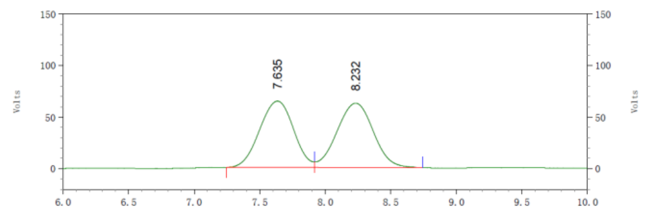

UV-WL1

RetTime (min)	Width (min)	Height (Volts)	Area	Area (%)
7.635	0.67	257266	4618068	49.063
8.232	0.82	249459	4794550	50.937

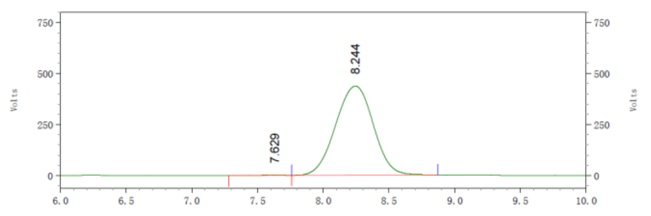

UV-WL1

RetTime (min)	Width (min)	Height (Volts)	Area	Area (%)
7.629	0.48	2202	30899	0.089
8.244	1.11	1746081	34845865	99.911

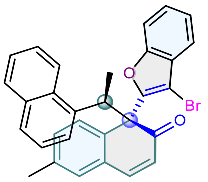

2ai

1-(3-bromobenzofuran-2-yl)-6-methyl-1-((R)-1-(naphthalen-1-yl)ethyl)naphthalen-2(1H)-one (2ai)

Compound **2ai** is an unknown compound, and was synthesized in 66% yield (16.7 mg, 0.05 mmol scale) under condition [A].

White solid. (R_f = 0.6, PE/EA = 10:1)

^{1}H NMR (400 MHz, $CDCl_3$) δ 7.92 (d, *J* = 8.3 Hz, 1H), 7.65 – 7.53 (m, 3H), 7.45 (d, *J* = 7.4 Hz, 1H), 7.37 (t, *J* = 7.5 Hz, 1H), 7.33 – 7.27 (m, 2H), 7.27 – 7.19 (m, 3H), 7.06 (d, *J* = 7.8 Hz, 1H), 6.84 (t, *J* = 9.6 Hz, 2H), 6.45 (s, 1H), 6.24 (d, *J* = 9.8 Hz, 1H), 5.29 (q, *J* = 6.6 Hz, 1H), 2.00 (s, 3H), 1.72 (d, *J* = 6.5 Hz, 3H).

^{13}C NMR (100 MHz, $CDCl_3$) δ 197.96, 155.85, 152.86, 144.59, 139.49, 136.78, 136.26, 132.83, 131.55, 130.41, 129.17, 128.83, 128.71, 128.64, 128.07, 127.56, 127.31, 126.78, 124.89, 124.72, 124.46, 123.95, 123.48, 123.44, 119.64, 111.38, 96.13, 60.97, 42.54, 20.47, 17.99.

HRMS (ESI) *m/z* Calcd for $C_{31}H_{24}BrO_2^+$ $[M + H]^+$: 507.0954, Found: 507.0952.

Optical Rotation: $[\alpha]_D^{25}$= -6.4° (*c* = 0.5, DCM).

HPLC analysis: Chiralcel IA-H (Hexane/*i*-PrOH = 95:5, flow rate = 1.0 mL/min, wave length = 254 nm), t_R = 6.588 min (minor), t_R = 7.927 min (major), 99:1 d. r.

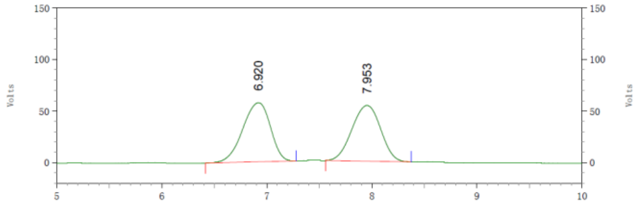

UV-WL1

RetTime (min)	Width (min)	Height (Volts)	Area	Area (%)
6.920	0.87	229560	4206418	50.617
7.953	0.81	216001	4103816	49.383

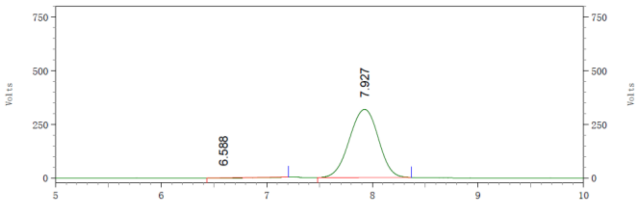

UV-WL1

RetTime (min)	Width (min)	Height (Volts)	Area	Area (%)
6.588	0.77	4260	273311	1.119
7.927	0.88	1268556	24161599	98.881

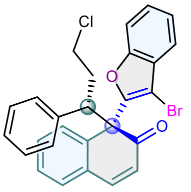

2aj

1-(3-bromobenzofuran-2-yl)-1-((S)-3-chloro-1-phenylpropyl)naphthalen-2(1H)-one (2aj)

Compound **2aj** is an unknown compound, and was synthesized in 76% yield (18.7 mg, 0.05 mmol scale) under condition [A].

Yellow solid. (R_f = 0.6, PE/EA = 10:1)

^{1}H NMR (400 MHz, $CDCl_3$) δ 7.64 (d, *J* = 8.2 Hz, 1H), 7.46 – 7.35 (m, 4H), 7.31 (d, *J* = 7.5 Hz, 1H), 7.29 – 7.24 (m, 2H), 7.17 (t, *J* = 7.3 Hz, 1H), 7.06 (t, *J* = 7.5 Hz, 2H), 6.92 (d, *J* = 9.9 Hz, 1H), 6.54 (d, *J* = 7.2 Hz, 2H), 5.62 (d, *J* = 9.9 Hz, 1H), 4.34 (d, *J* = 10.8 Hz, 1H), 3.38 – 3.26 (m, 2H), 2.89 – 2.79 (m, 1H), 2.25 – 2.11 (m, 1H).

^{13}C NMR (100 MHz, $CDCl_3$) δ 198.92, 153.88, 153.25, 144.58, 138.56, 136.05, 131.84, 130.24, 129.35, 129.20, 129.14, 128.59, 128.22, 127.93, 127.72, 125.12, 124.91, 123.50, 119.52, 111.75, 96.55, 61.71, 51.75, 42.66, 35.26.

HRMS (ESI) *m/z* Calcd for $C_{27}H_{21}BrClO_2^+$ $[M + H]^+$: 491.0408, Found: 491.0403.

Optical Rotation: $[\alpha]_D^{25}$= -178.5° (c = 0.5, DCM).

HPLC analysis: Chiralcel IB-H (Hexane/i-PrOH = 98.5:1.5, flow rate = 1.0 mL/min, wave length = 254 nm), t_R = 13.791 min (minor), t_R = 16.985 min (major), >99:1 d. r.

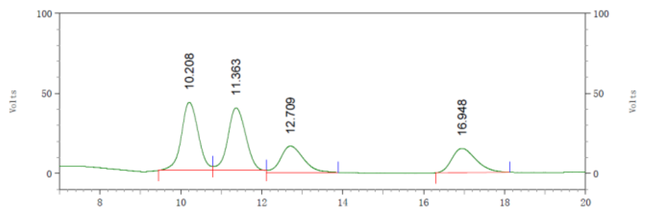

UV-WL1

RetTime (min)	Width (min)	Height (Volts)	Area	Area (%)
10.208	1.35	170354	4922412	33.278
11.363	1.31	156364	4789061	32.377
12.709	1.79	65938	2573107	17.396
16.948	1.83	60445	2507177	16.950

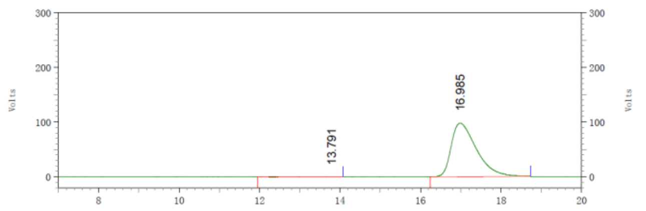

UV-WL1

RetTime (min)	Width (min)	Height (Volts)	Area	Area (%)
13.791	2.11	522	2996	0.018
16.985	2.48	391874	17022488	99.982

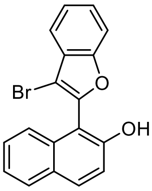

3a

1-((2-(benzylthio)phenyl)ethynyl)naphthalen-2-ol (3a)

White solid. (R_f = 0.4, PE/EA= 10:1)

^{1}H NMR (400 MHz, $CDCl_3$) δ 7.88 (d, J = 8.9 Hz, 1H), 7.80 (d, J = 8.0 Hz, 1H), 7.64 (d, J = 7.7 Hz, 1H), 7.54 (d, J = 8.0 Hz, 2H), 7.45 – 7.33 (m, 4H), 7.24 (d, J = 8.9 Hz, 1H), 5.70 (s, 1H).

^{13}C NMR (100 MHz, $CDCl_3$) δ 154.60, 153.18, 147.67, 132.82, 132.75, 128.75, 128.27, 128.25, 127.40, 125.99, 124.50, 123.97, 123.75, 119.97, 117.69, 111.77, 108.01, 100.29.

HRMS (ESI) m/z Calcd for $C_{18}H_{11}BrNaO_2^+$ $[M + Na]^+$: 360.9835, Found: 360.9833.

Control experiments

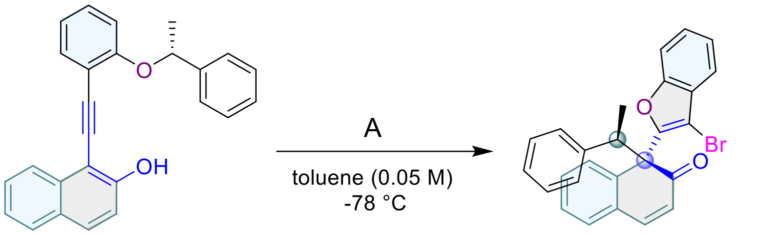

reaction conditions:	d.r.	conv. (%)
A1: NBS (1.05 eq.), catalyst-**C** (10 mol%)	99:1	100
A2: NBS (1.05 eq.), DABCO (10 mol%)	46:54	100

A solution of **1ac** (0.05 mmol, 1.0 equiv.) and catalyst **C** or DABCO (10 mol%) in toluene (1.0 mL) at -78 °C for 15 min, then NBS (1.05 eq.) at -78 °C, 12 h. The diastereomeric (d.r.) was determined by

high-performance liquid chromatography (HPLC) analysis with a chiral stationary phase. The conversion ratio was determined by ^{1}H NMR analysis of the crude mixtures.

HPLC analysis: Chiralcel IB-H (Hexane/*i*-PrOH = 95:5, flow rate = 1.0 mL/min, wave length = 254 nm), t_R = 5.981 min (major), t_R = 7.957 min (minor), 99:1 d.r.

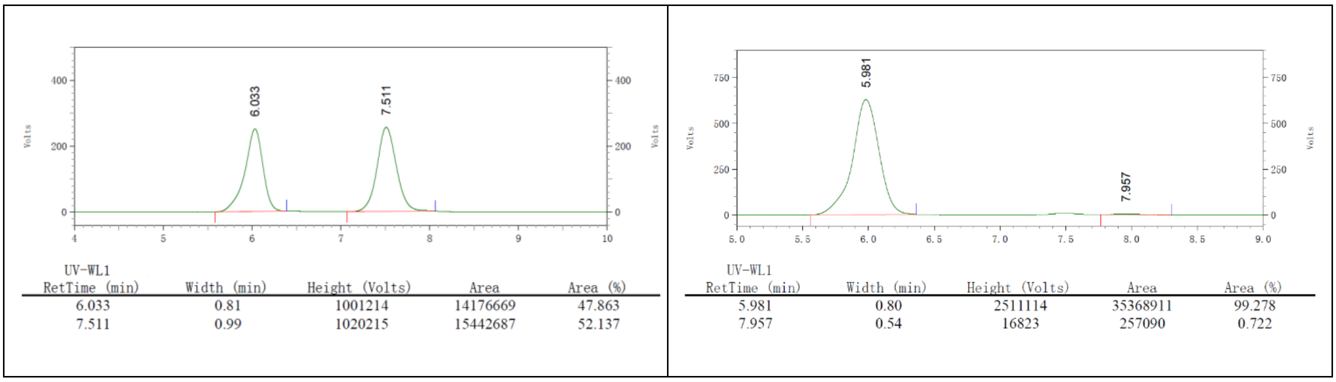

HPLC analysis: Chiralcel IB-H (Hexane/*i*-PrOH = 95:5, flow rate = 1.0 mL/min, wave length = 254 nm), t_R = 5.977 min (minor), t_R = 7.802 min (major), 46:54 d. r.

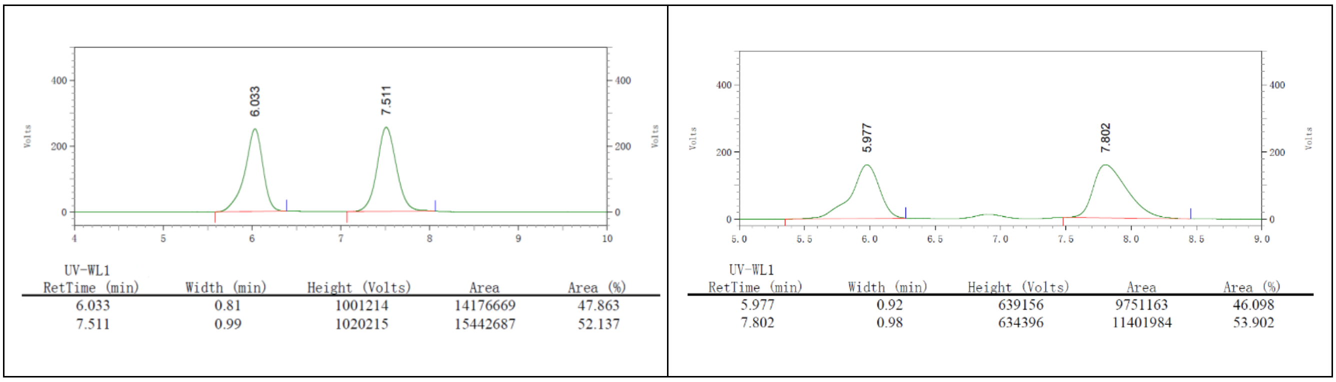

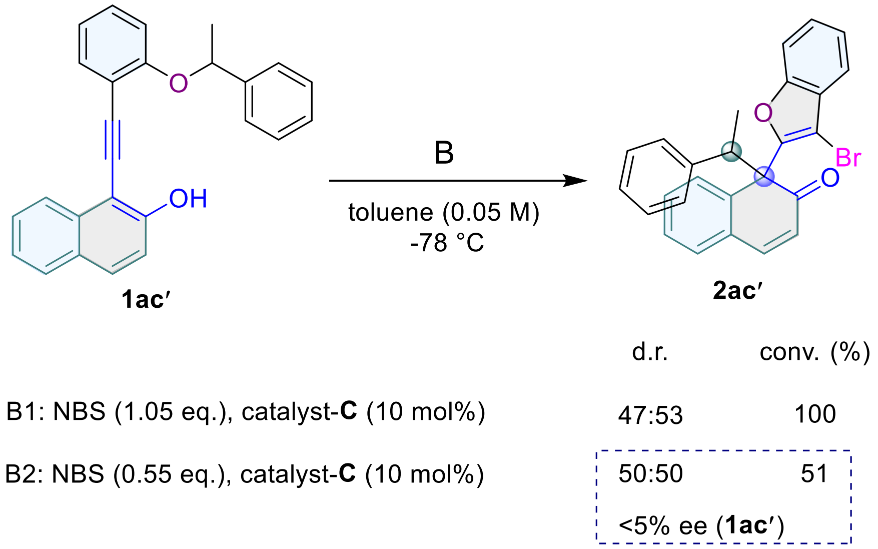

A solution of **1ac′** (0.05 mmol, 1.0 equiv.) and catalyst **C** (10 mol%) in toluene (1.0 mL) at -78 °C for 15 min, then NBS (1.05 or 0.55 eq.) at -78 °C, 12 h. The diastereomeric (d.r.) was determined by high-

performance liquid chromatography (HPLC) analysis with a chiral stationary phase. The conversion ratio was determined by ^1H NMR analysis of the crude mixtures.

Crossover experiments

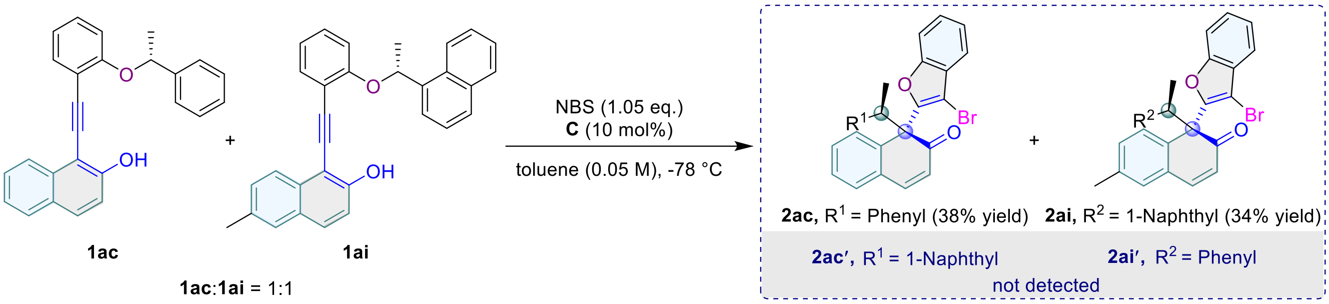

A solution of **1ac** (0.05 mmol, 1.0 equiv.), **1ai** (0.05 mmol, 1.0 equiv.) and catalyst **C** (10 mol%) in toluene (2.0 mL) at -78 °C for 15 min, then NBS (1.05 eq.) at -78 °C. After stirring at -78 °C for 12 h, the mixture was subjected to silica gel flash column chromatography using PE/EA eluent (40:1 to 25:1) to afford the product.

Electrophilic reagent screening

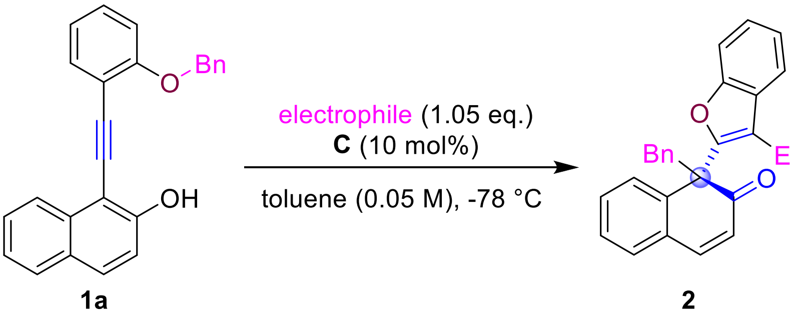

electrophile	yield of **2** [%]		ee of **2** [%]
NCS	--	n.d.	--
NIS	**2a′**	62	0
diphenyl sulfide	--	n.d.	--
diphenyl selenide	--	n.d.	--
selectfluor	--	n.d.	--
1-(phenylthio)pyrrolidine-2,5-dione	--	n.d.	--

A solution of **1a** (0.05 mmol, 1.0 equiv.) and catalyst **C** (10 mol%) in toluene (1.0 mL) at -78 °C for 15 min, then electrophile (1.05 eq.) at -78 °C. After stirring at -78 °C for 12 h, the mixture was subjected to silica gel flash column chromatography using PE/EA eluent (40:1 to 25:1) to afford the product.

^{1}H and ^{13}C NMR spectra

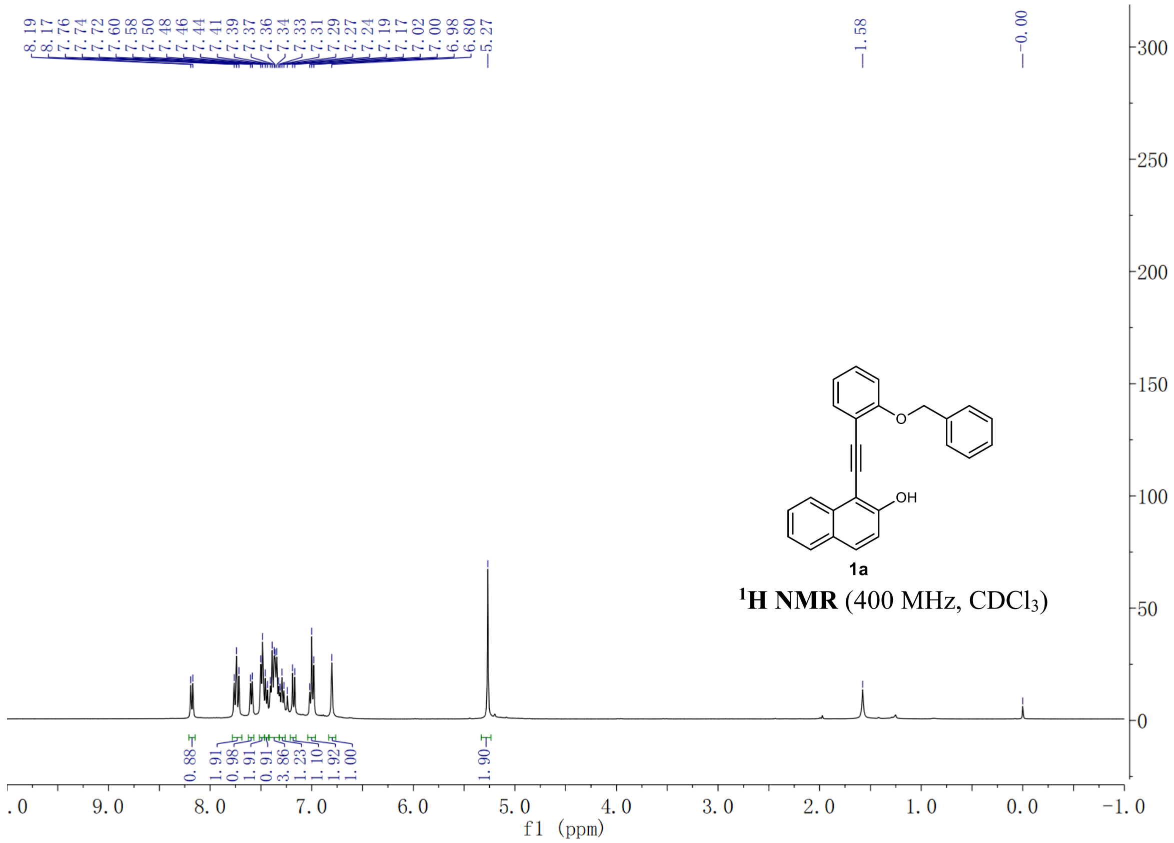

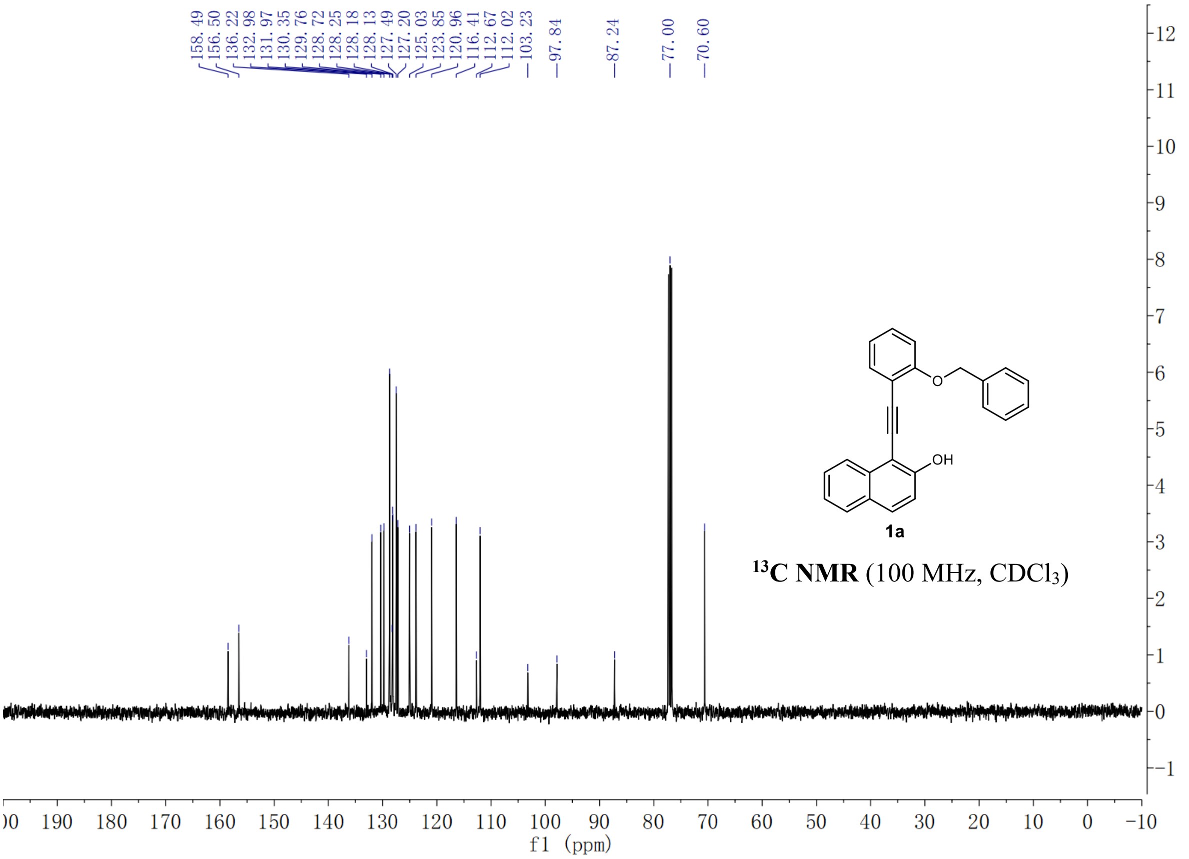

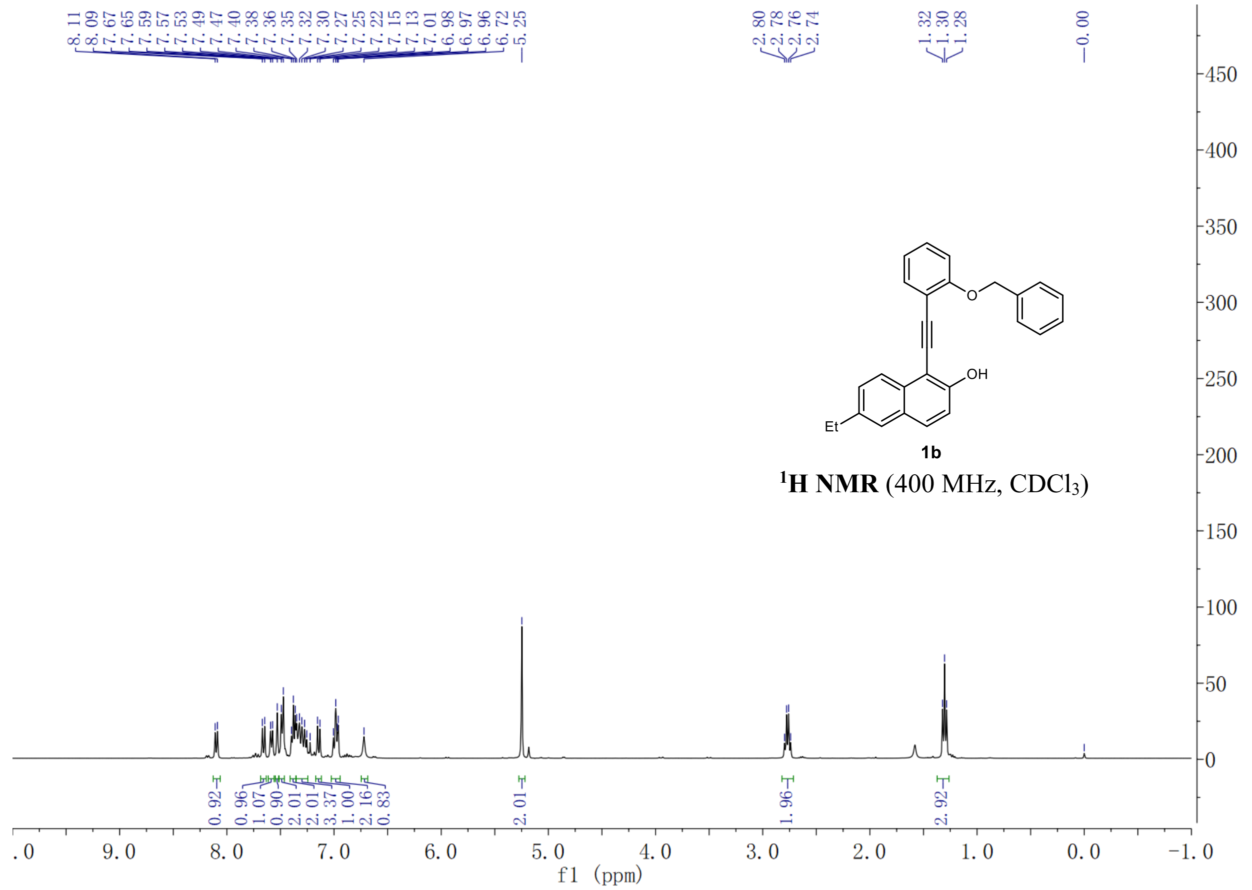

^{1}H NMR (400 MHz, $CDCl_3$)

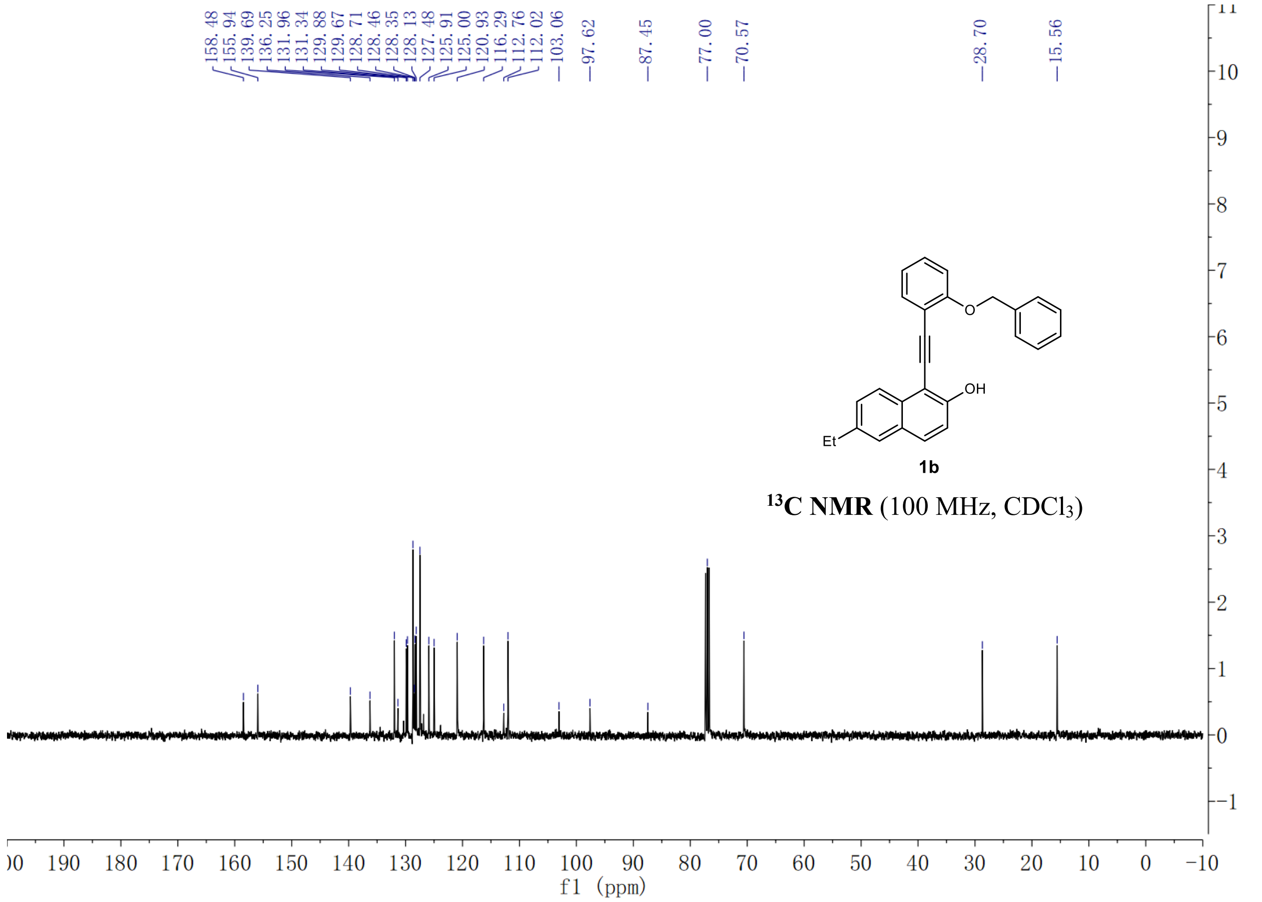

^{13}C NMR (100 MHz, $CDCl_3$)

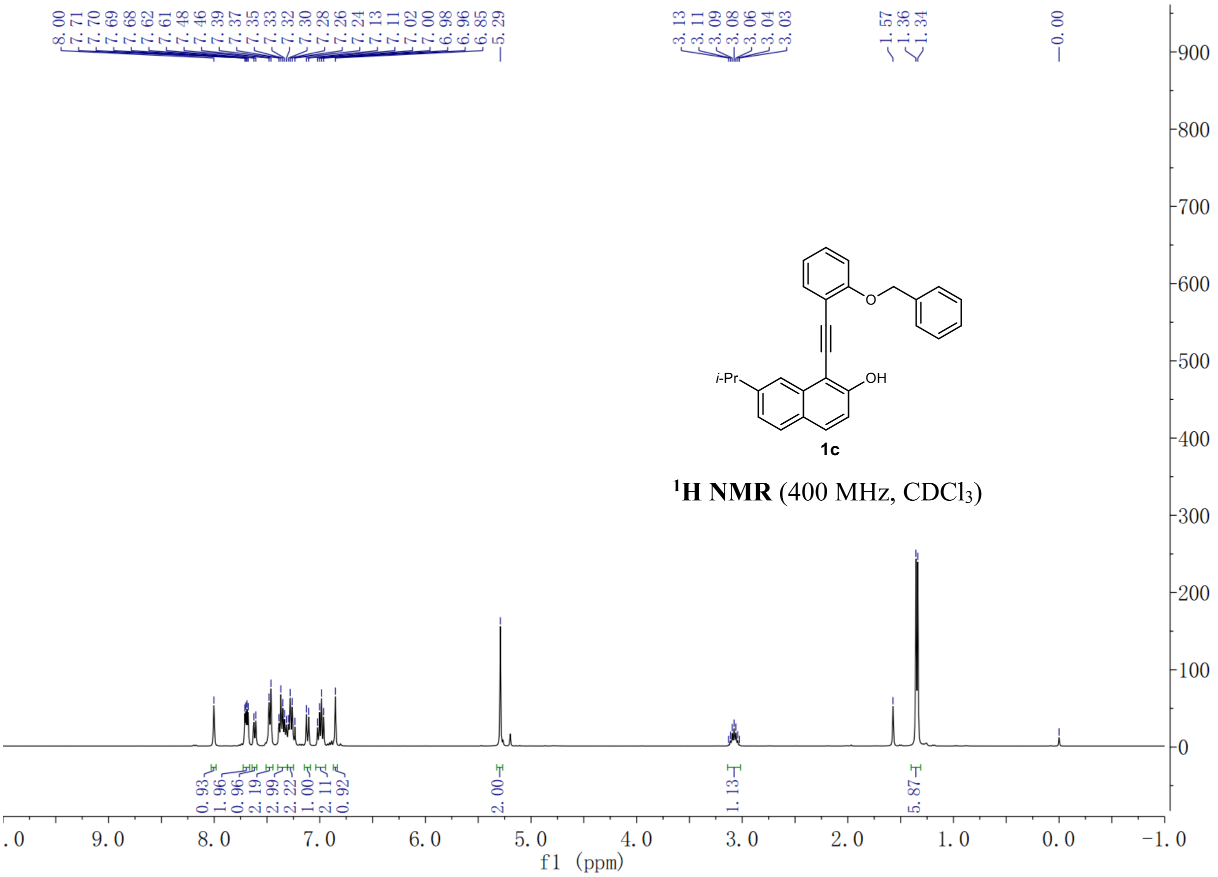
8.00 7.71 7.70 7.69 7.68 7.62 7.61 7.48 7.46 7.39 7.37 7.35 7.33 7.32 7.30 7.28 7.26 7.24 7.13 7.11 7.02 7.00 6.98 6.96 6.85 5.29
3.13 3.11 3.09 3.08 3.06 3.04 3.03
1.57 1.36 1.34
0.00
O
i-Pr
OH
1c
1H NMR (400 MHz, CDCl3)
0.93 1.96 0.96 2.19 2.99 2.22 1.00 2.11 0.92
2.00
1.13
5.87
f1 (ppm)

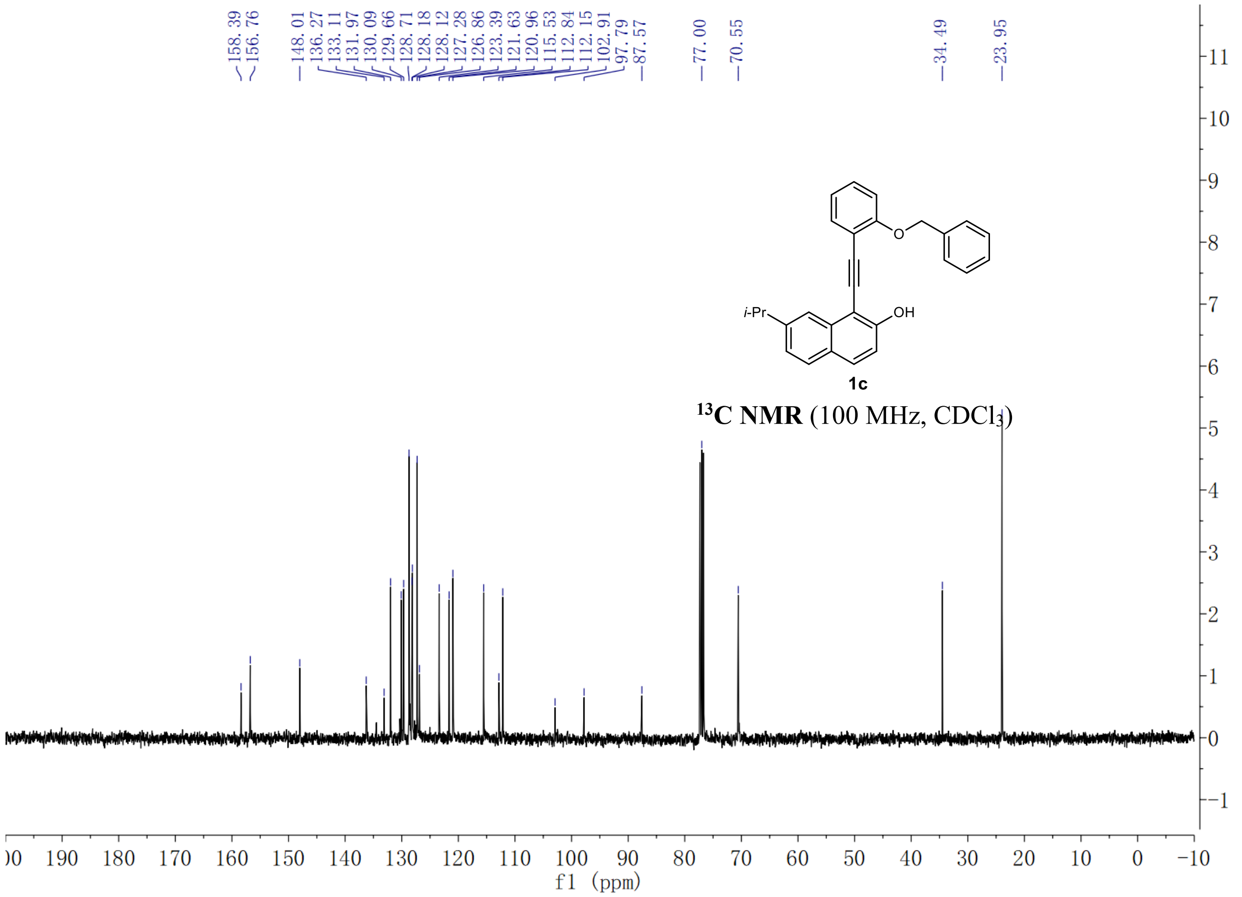
158.39 156.76 148.01 136.27 133.11 131.97 130.09 129.66 128.71 128.18 128.12 127.28 126.86 123.39 121.63 120.96 115.53 112.84 112.15 102.91 97.79 87.57 77.00 70.55 34.49 23.95
O
i-Pr
OH
1c
13C NMR (100 MHz, CDCl3)
f1 (ppm)

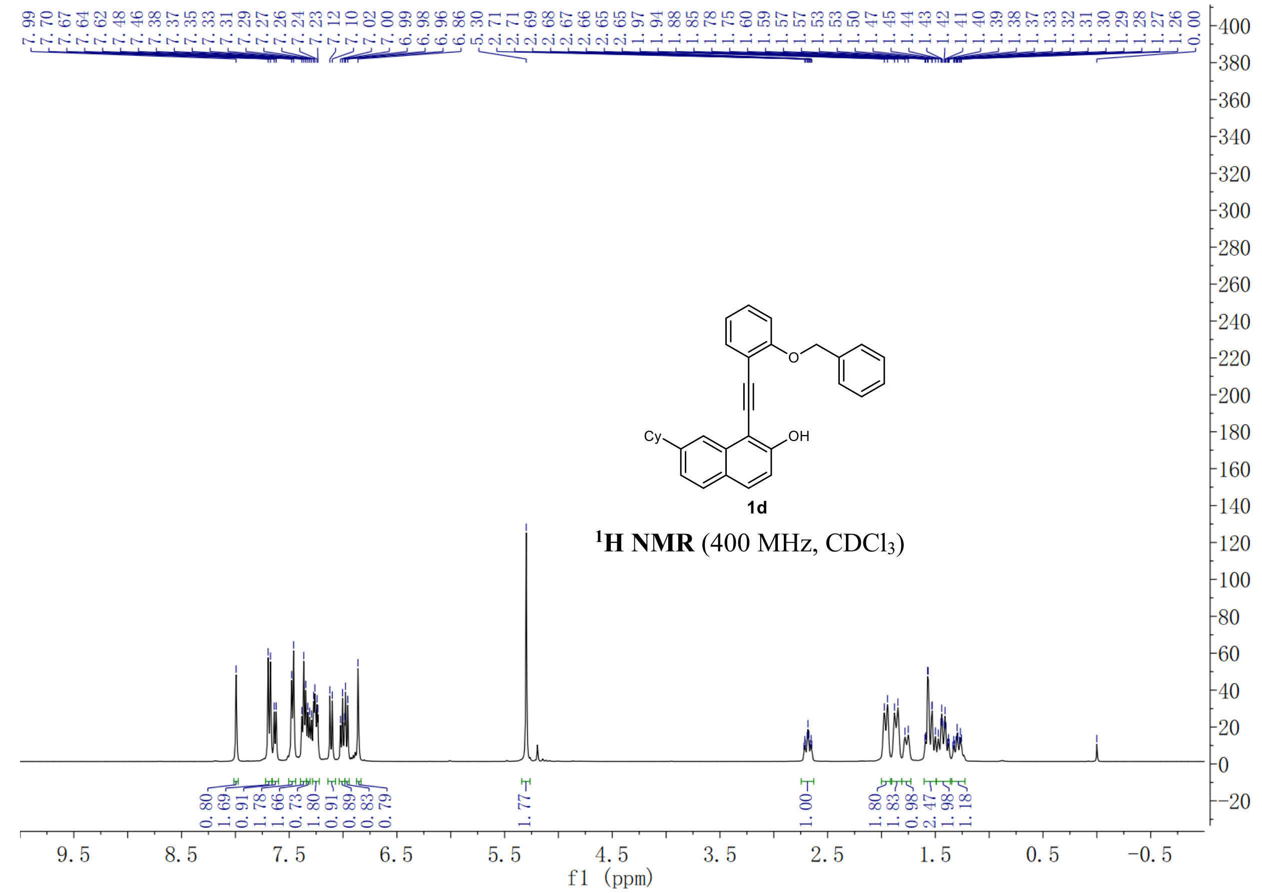

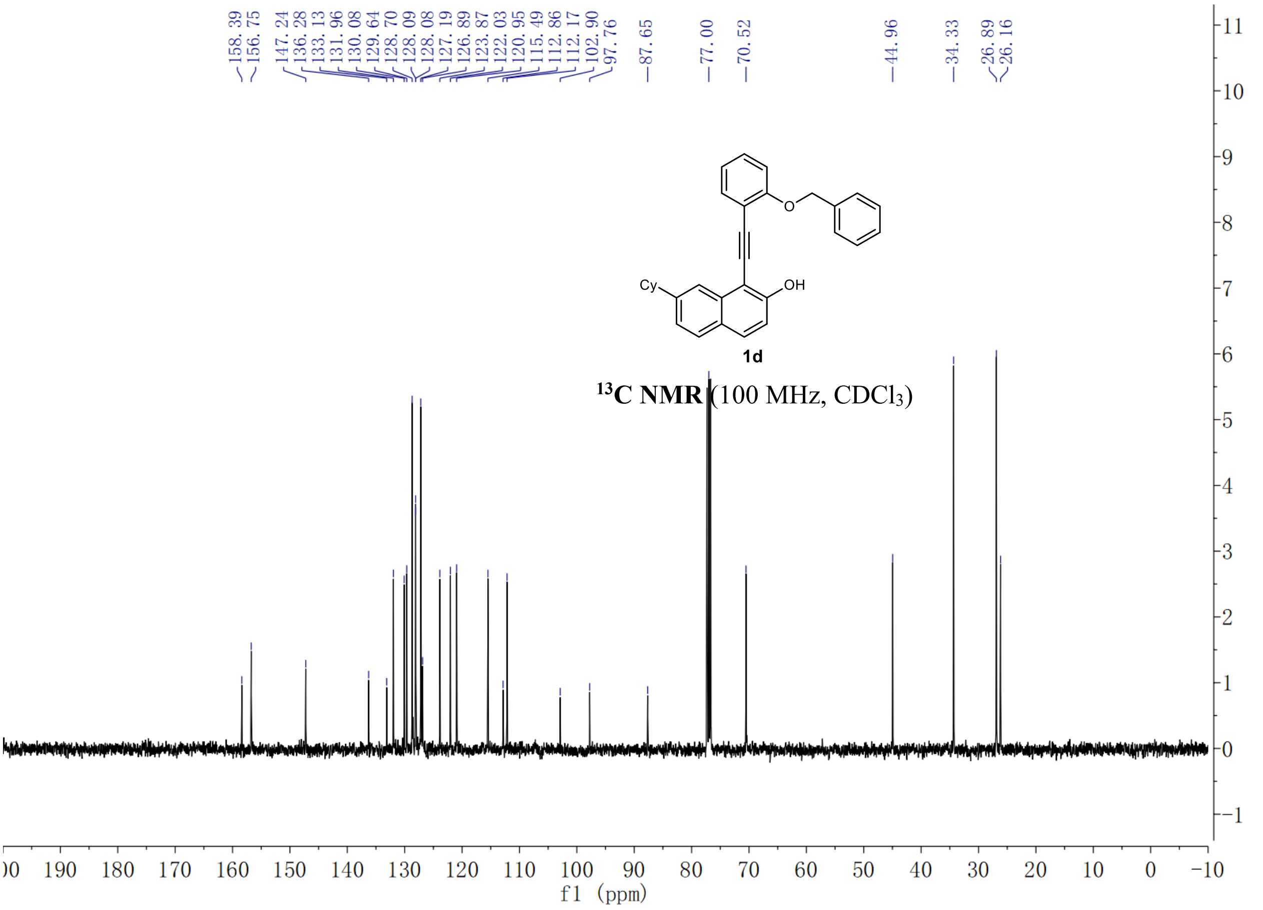

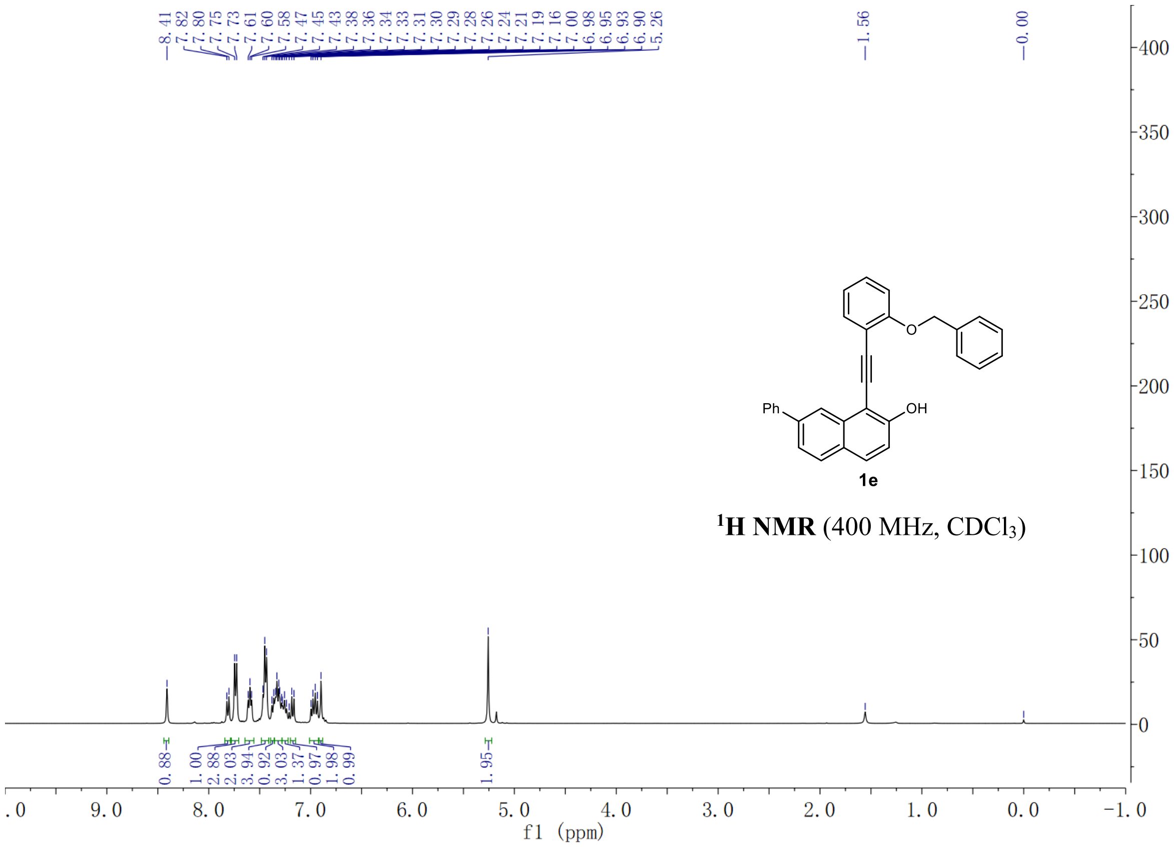

Ph
OH
O
1e
¹H NMR (400 MHz, CDCl₃)
f1 (ppm)

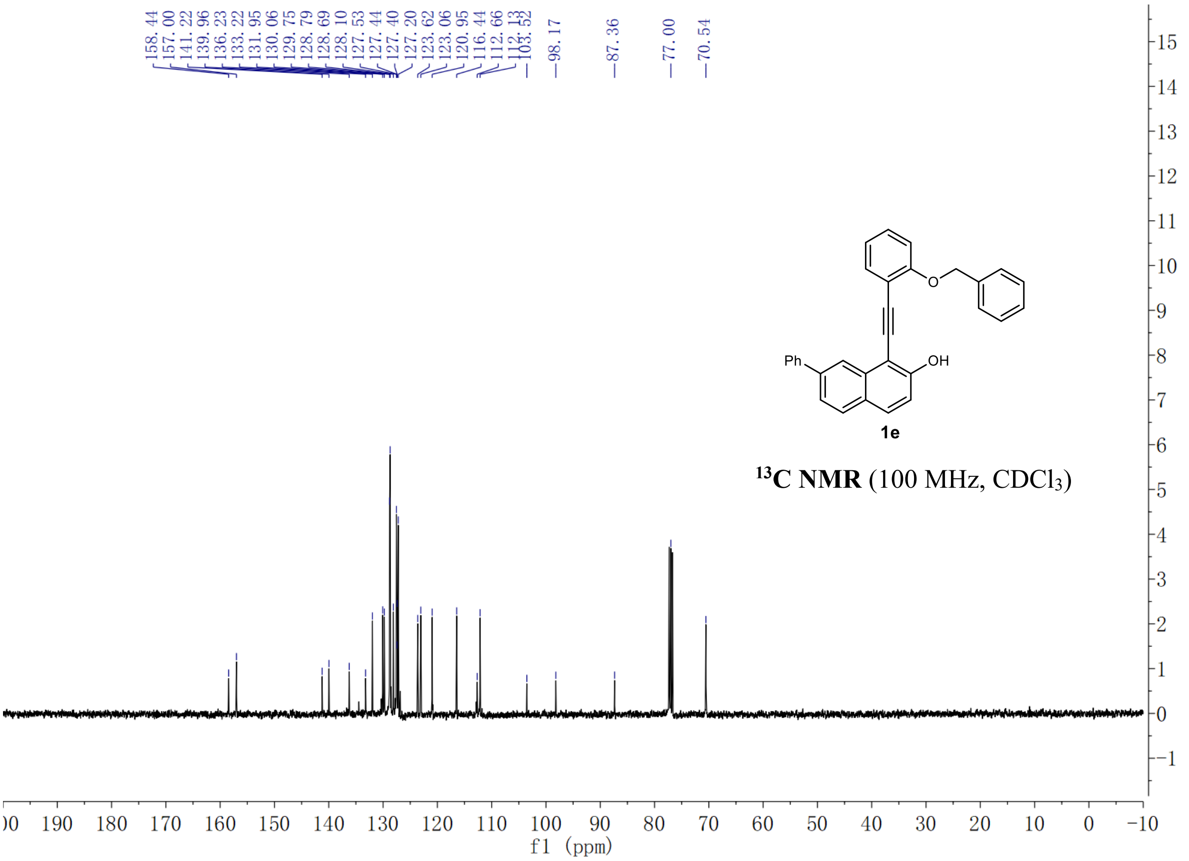

Ph
OH
O
1e
¹³C NMR (100 MHz, CDCl₃)
f1 (ppm)

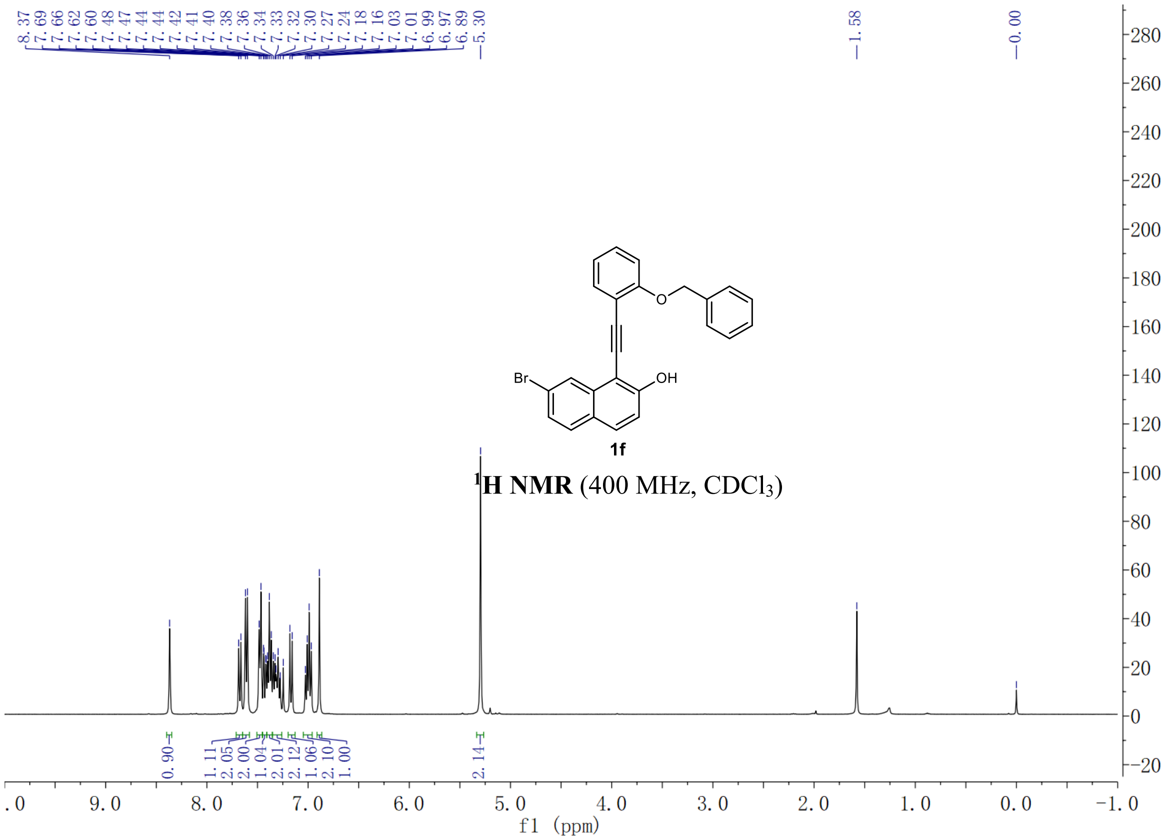

¹H NMR (400 MHz, $CDCl_3$)

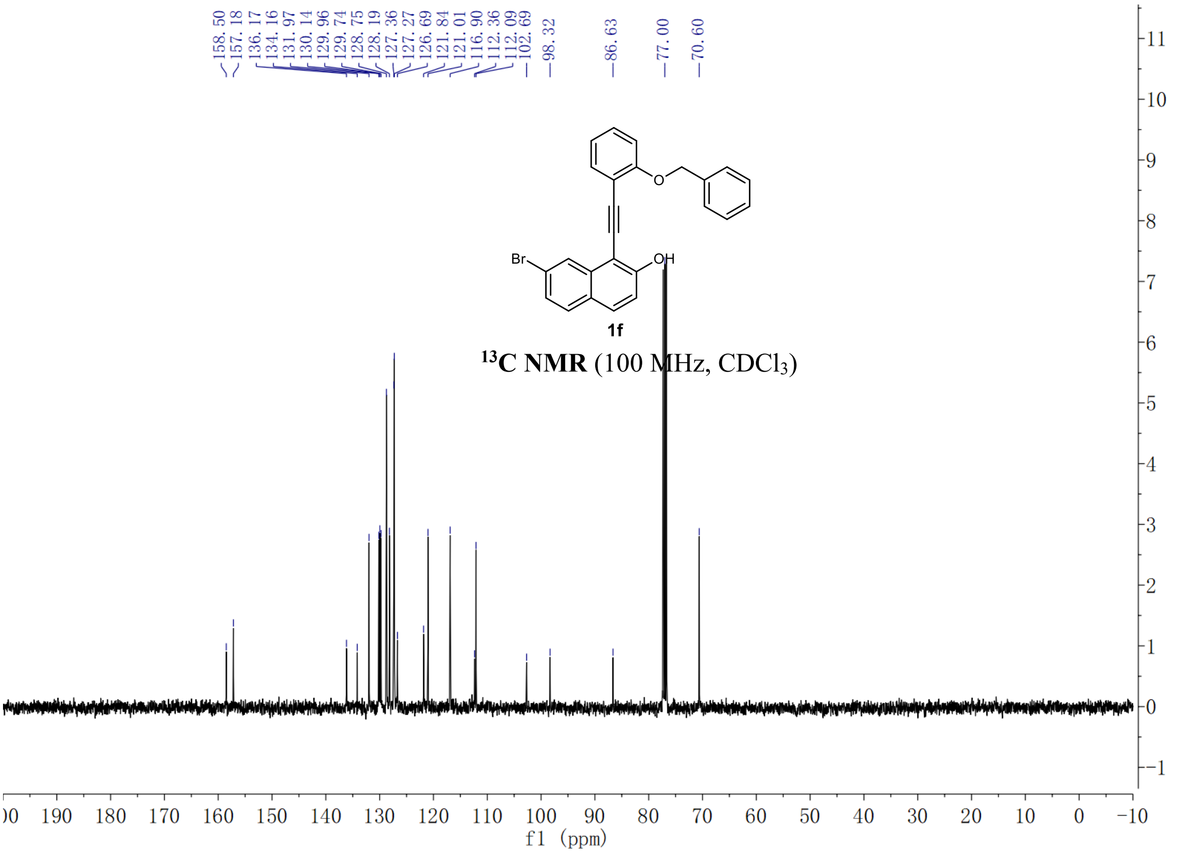

¹³C NMR (100 MHz, $CDCl_3$)

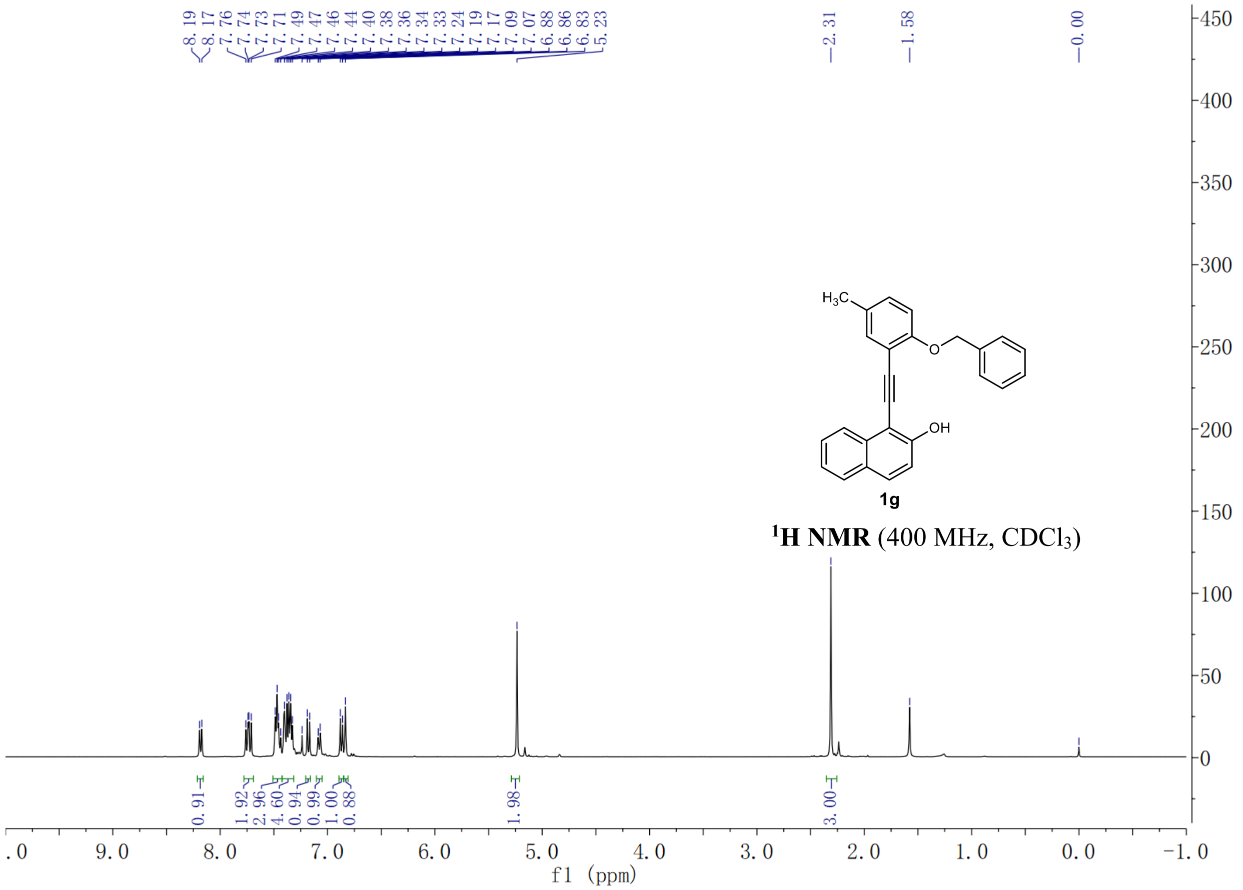

1g

^{1}H NMR (400 MHz, CDCl3)

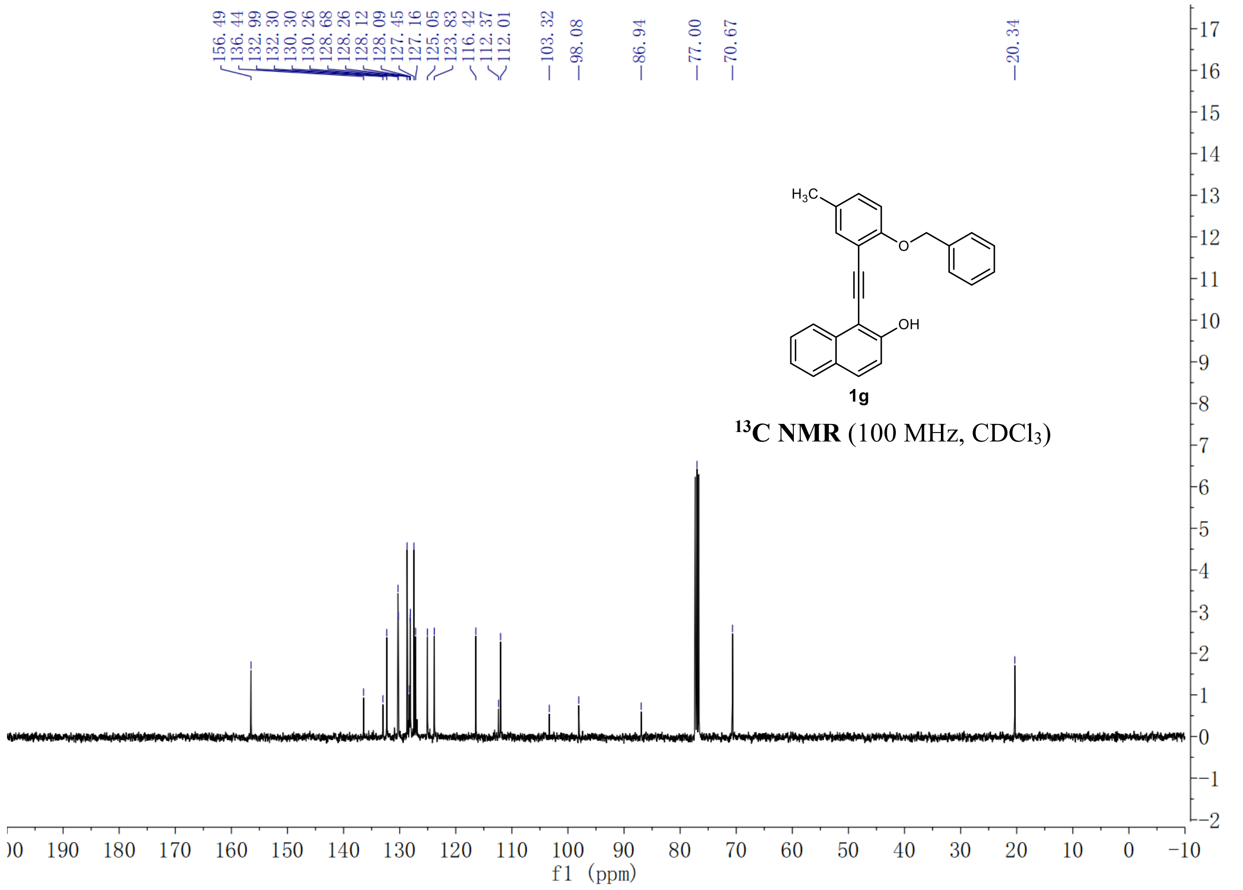

1g

^{13}C NMR (100 MHz, CDCl3)

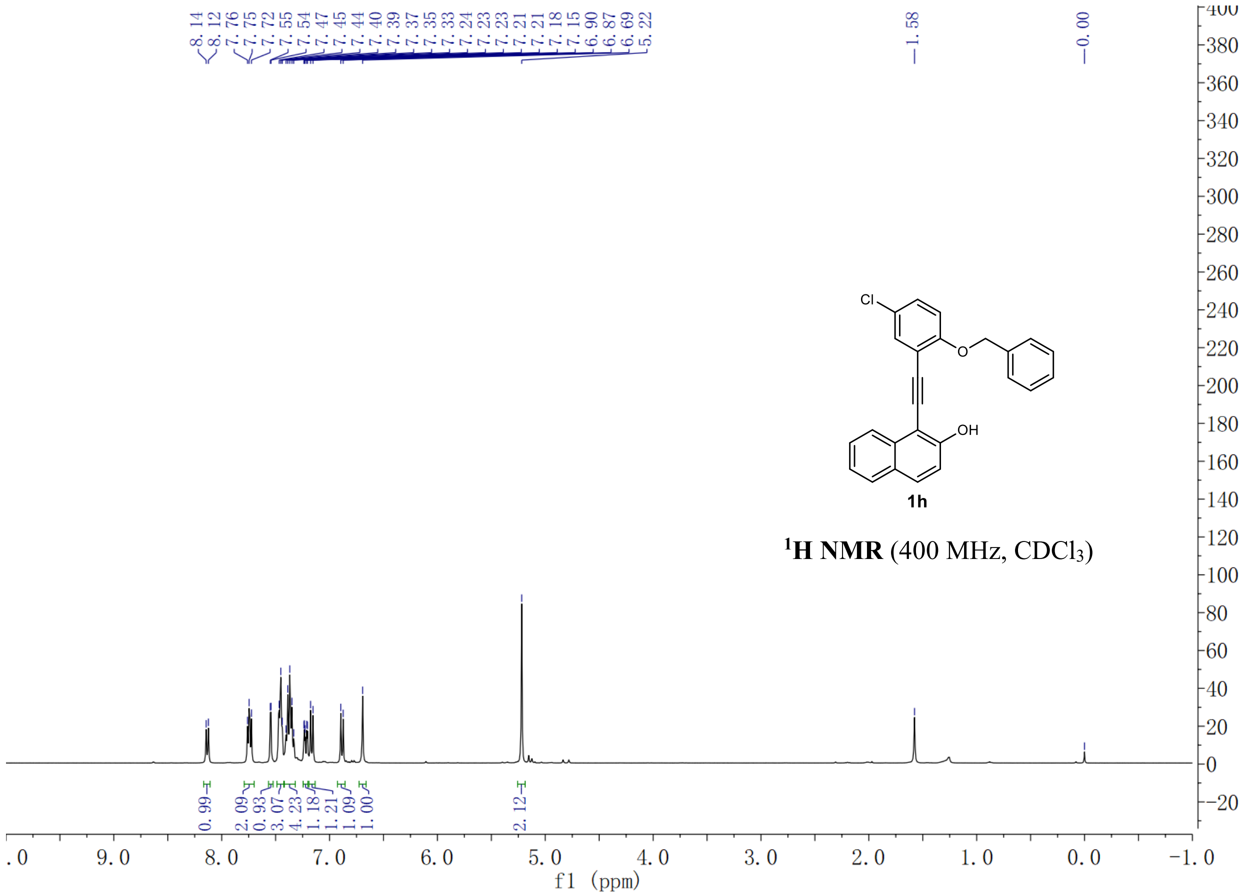
1h
1H NMR (400 MHz, CDCl3)

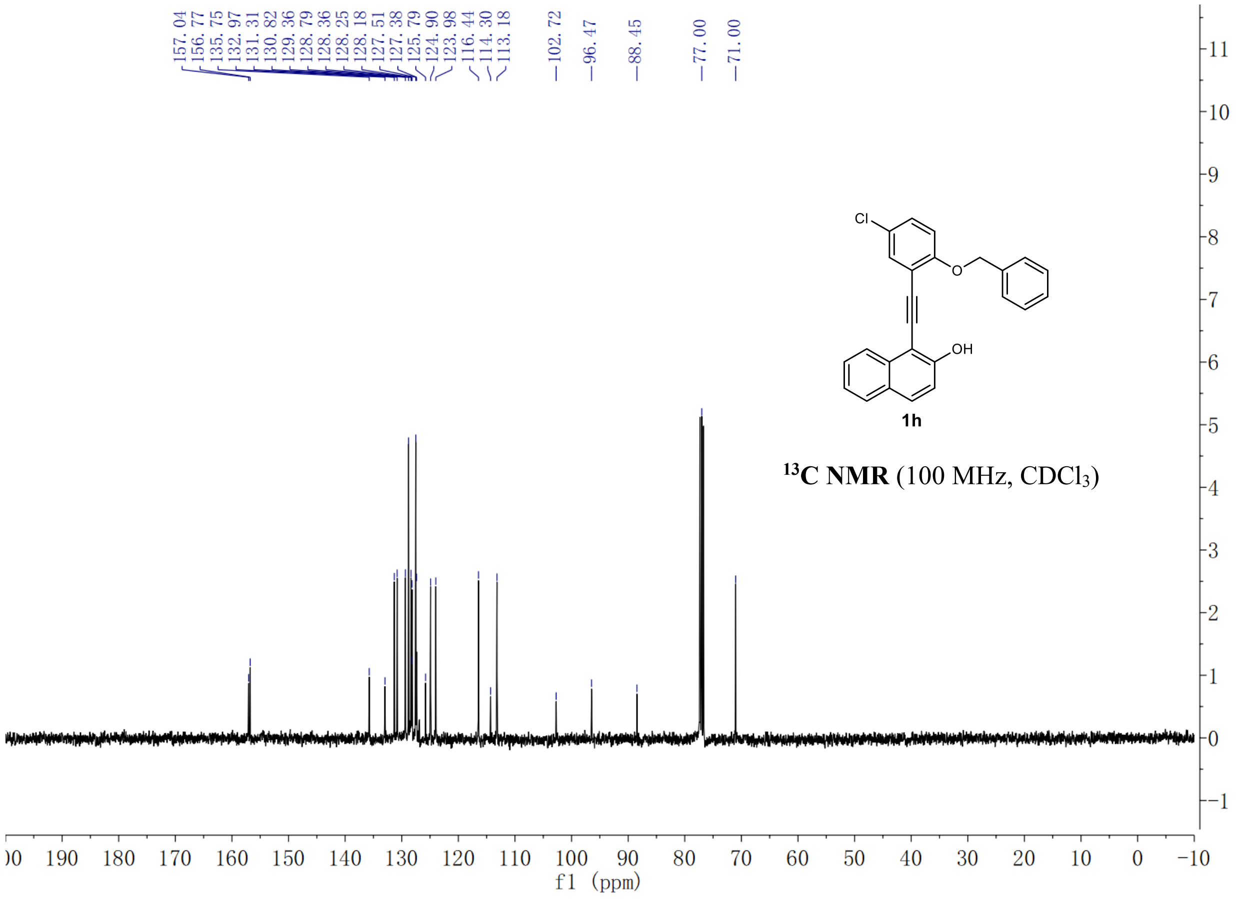
1h
13C NMR (100 MHz, CDCl3)

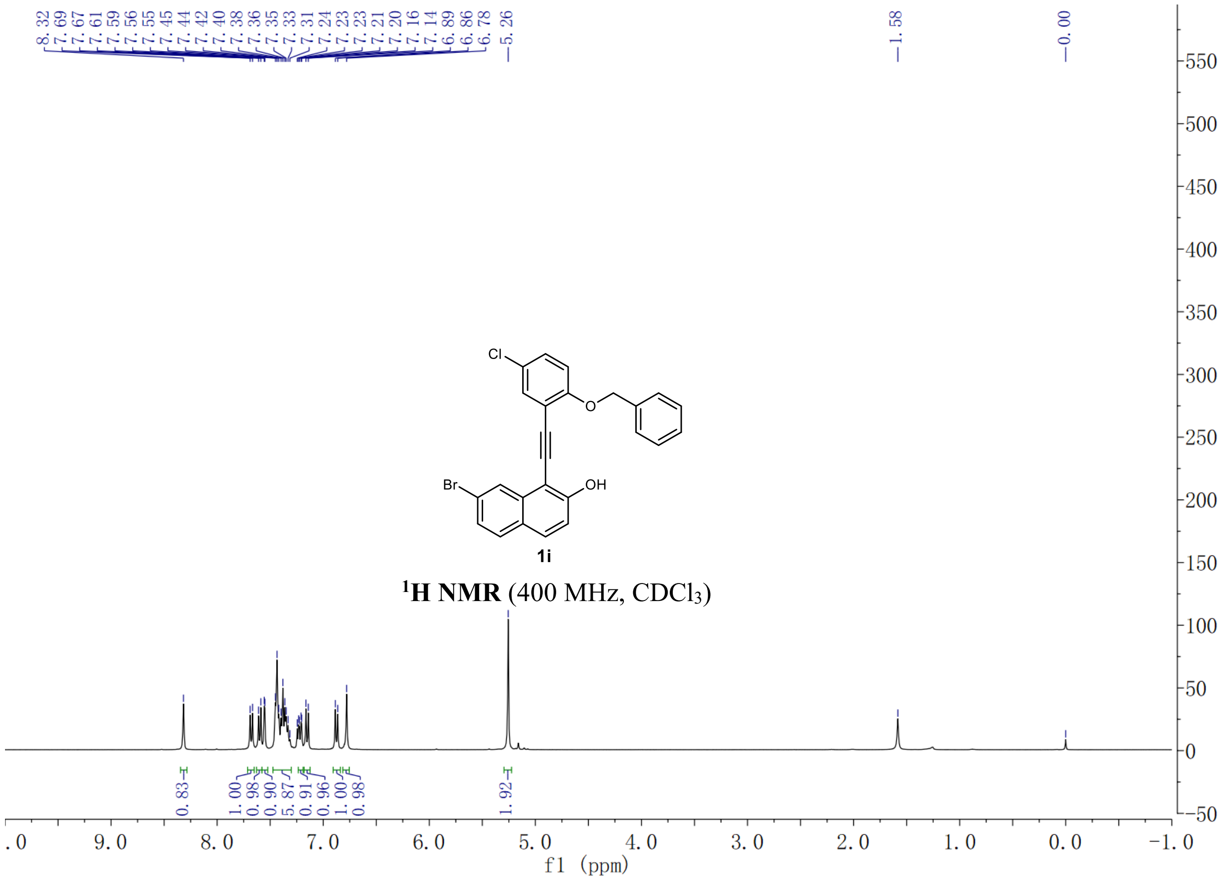

^{1}H NMR (400 MHz, $CDCl_3$)

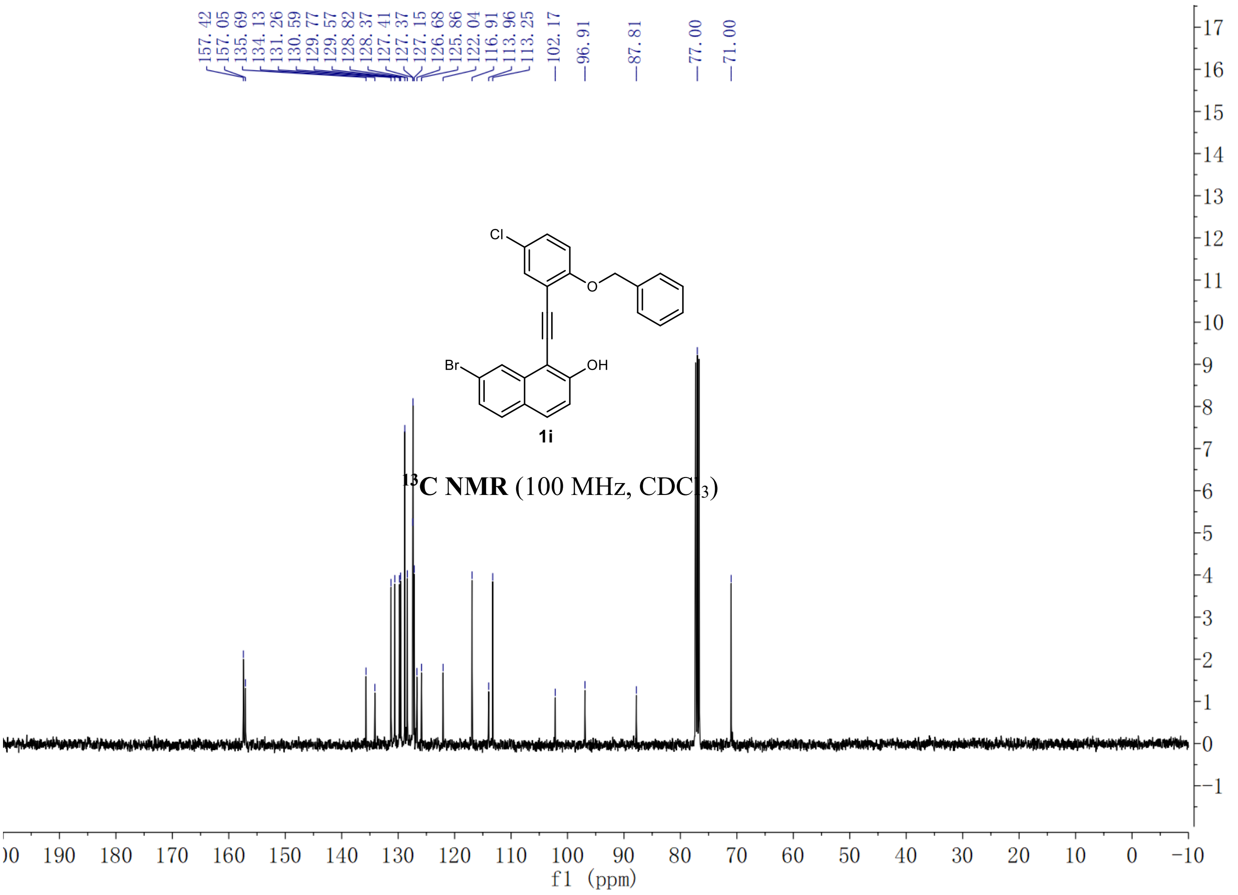

^{13}C NMR (100 MHz, $CDCl_3$)

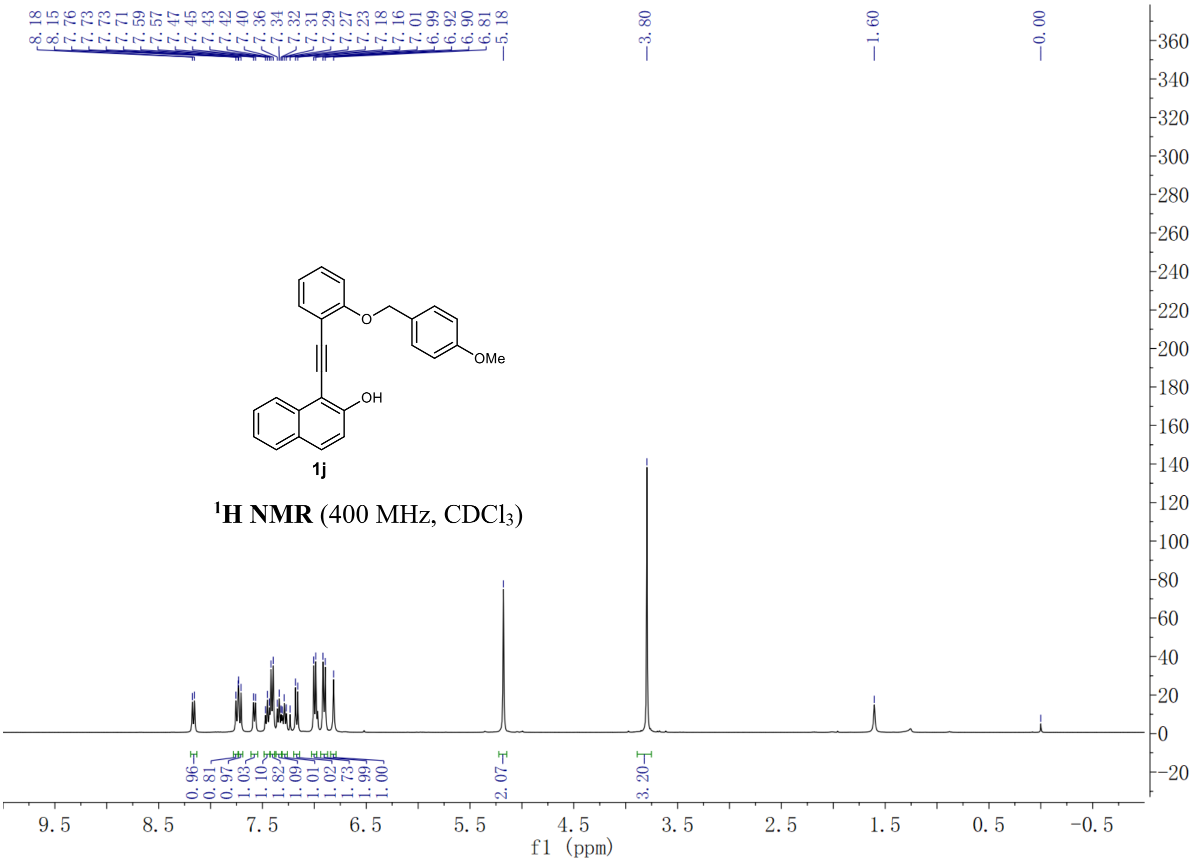

OMe
OH
O
1j
¹H NMR (400 MHz, CDCl3)
f1 (ppm)

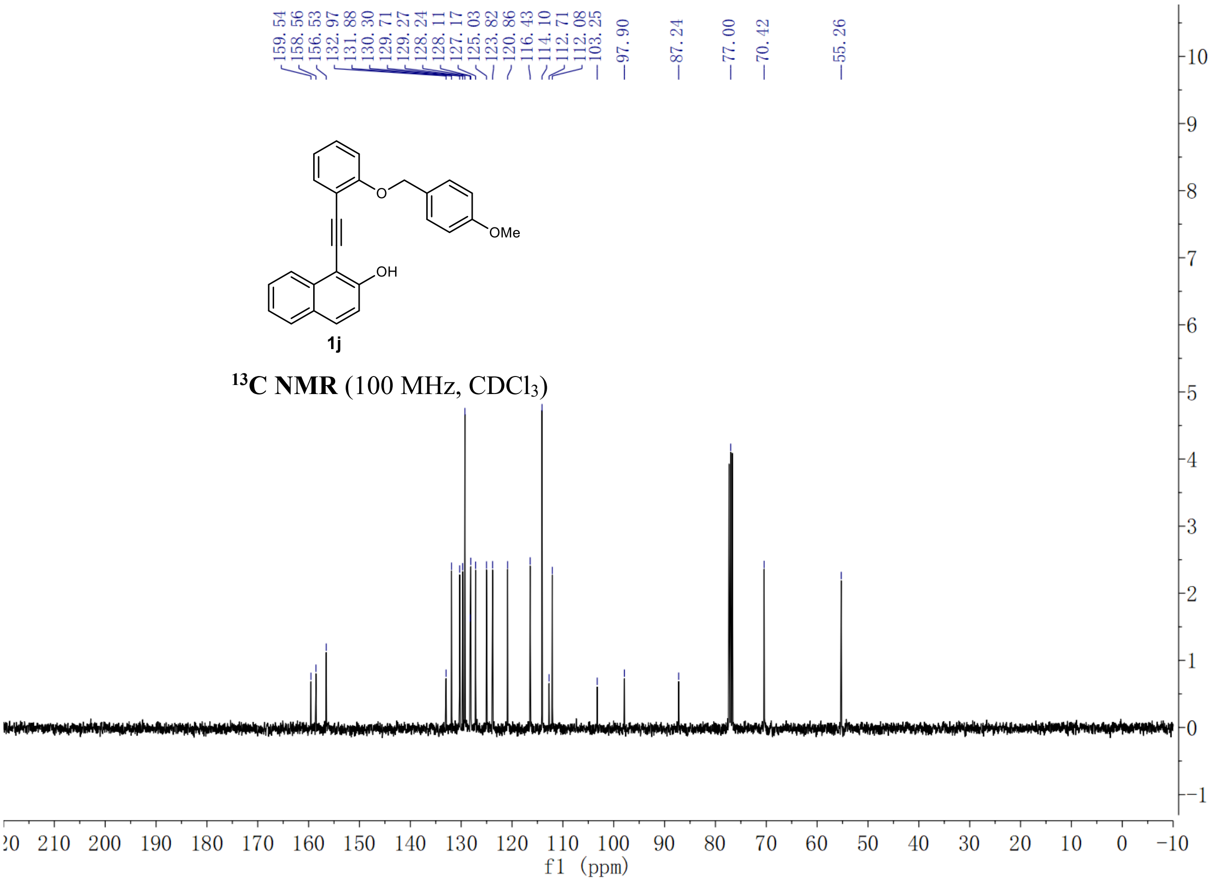

OMe
OH
O
1j
¹³C NMR (100 MHz, CDCl3)
f1 (ppm)

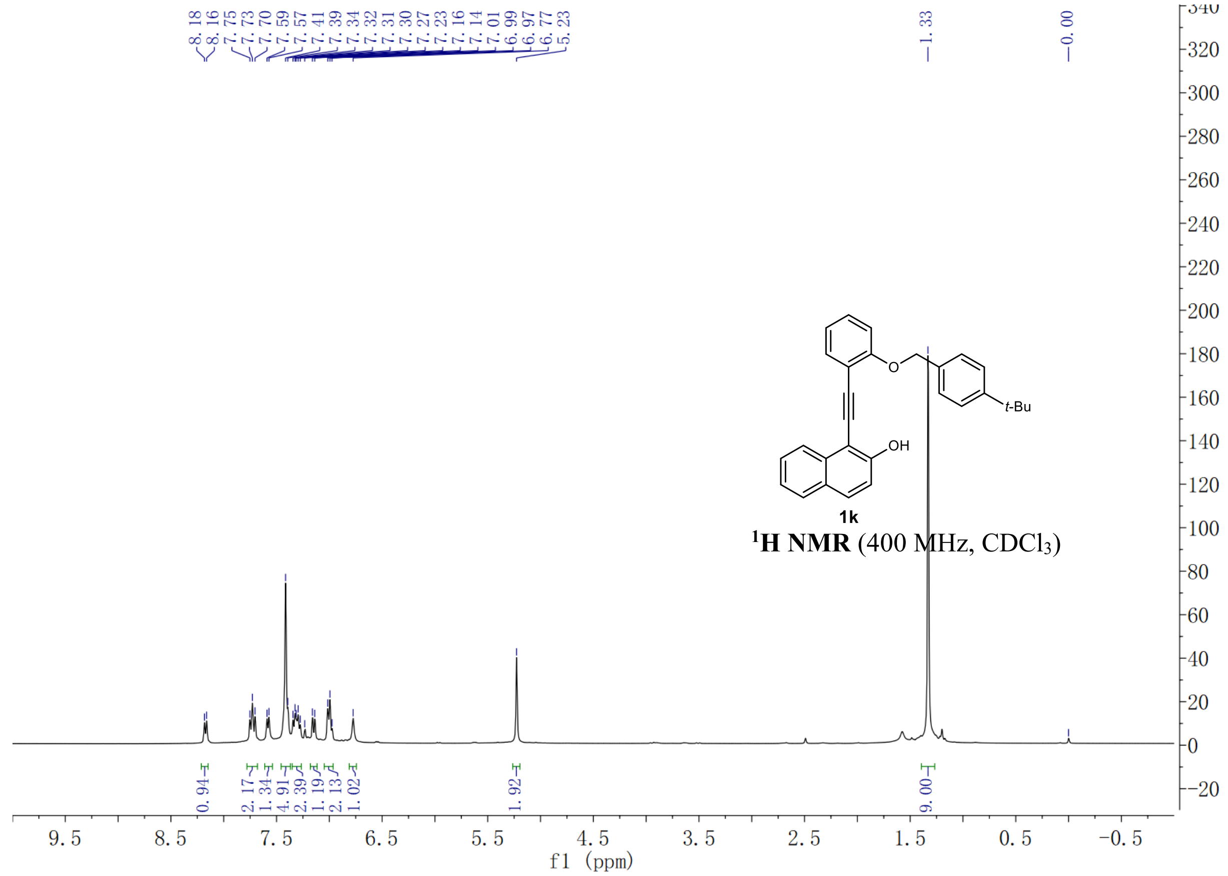

1k
^{1}H NMR (400 MHz, CDCl$_3$)

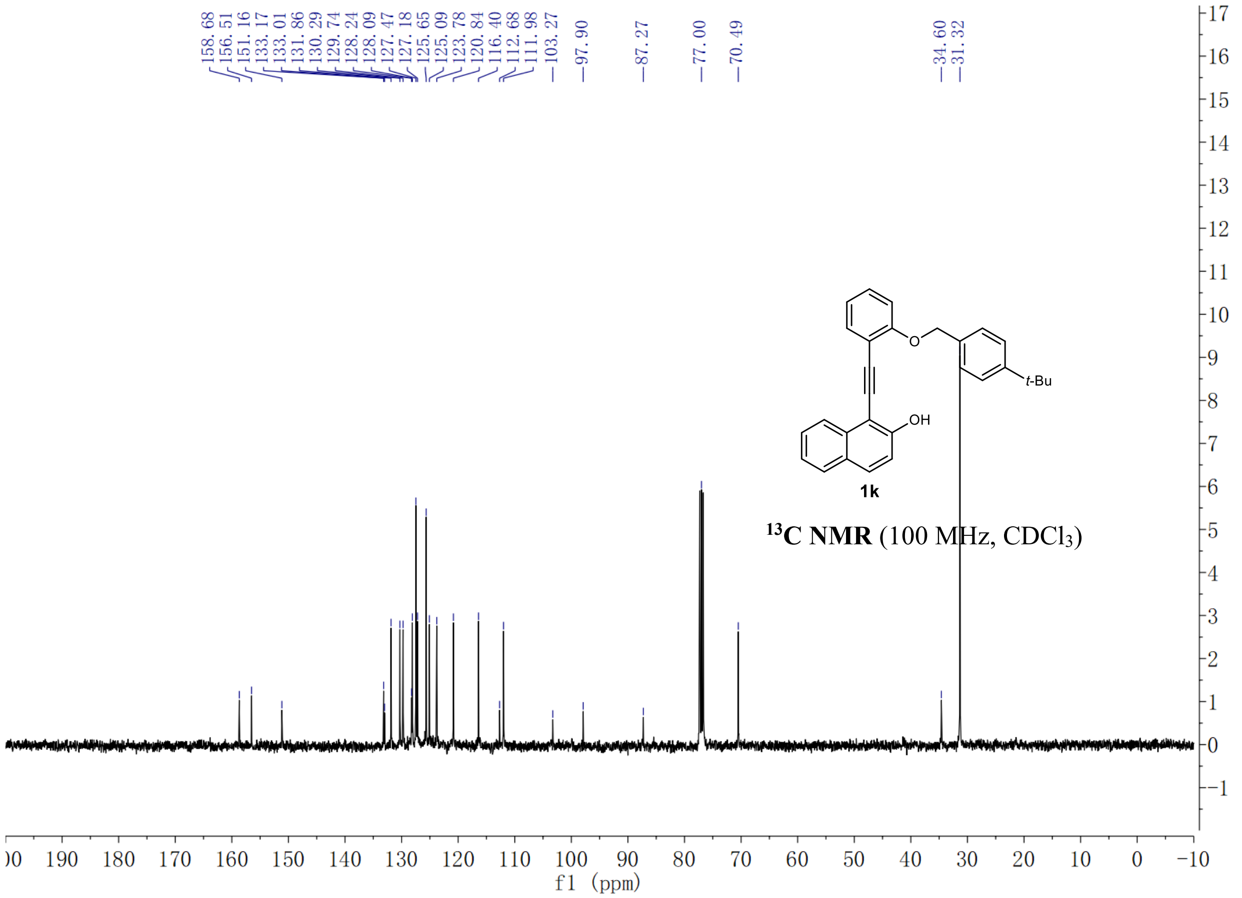

1k
^{13}C NMR (100 MHz, CDCl$_3$)

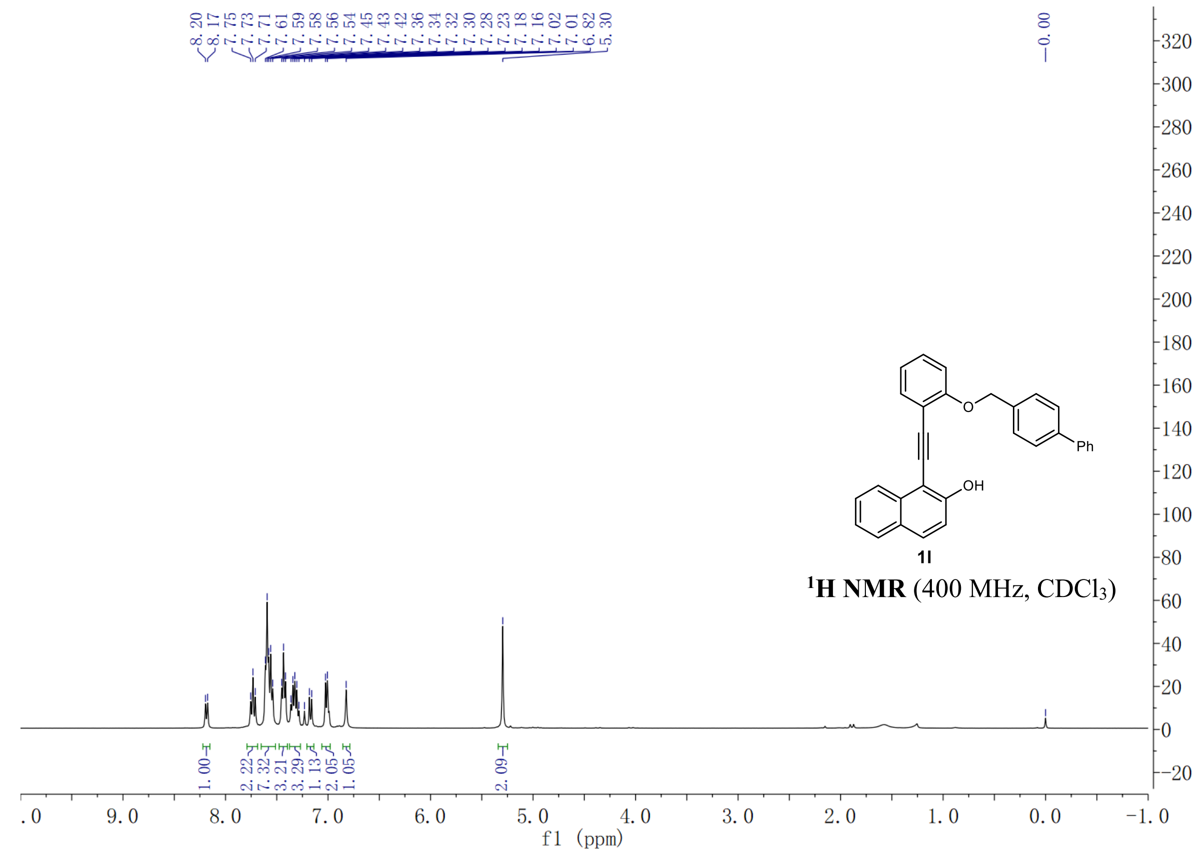

1l

^{1}H NMR (400 MHz, CDCl$_3$)

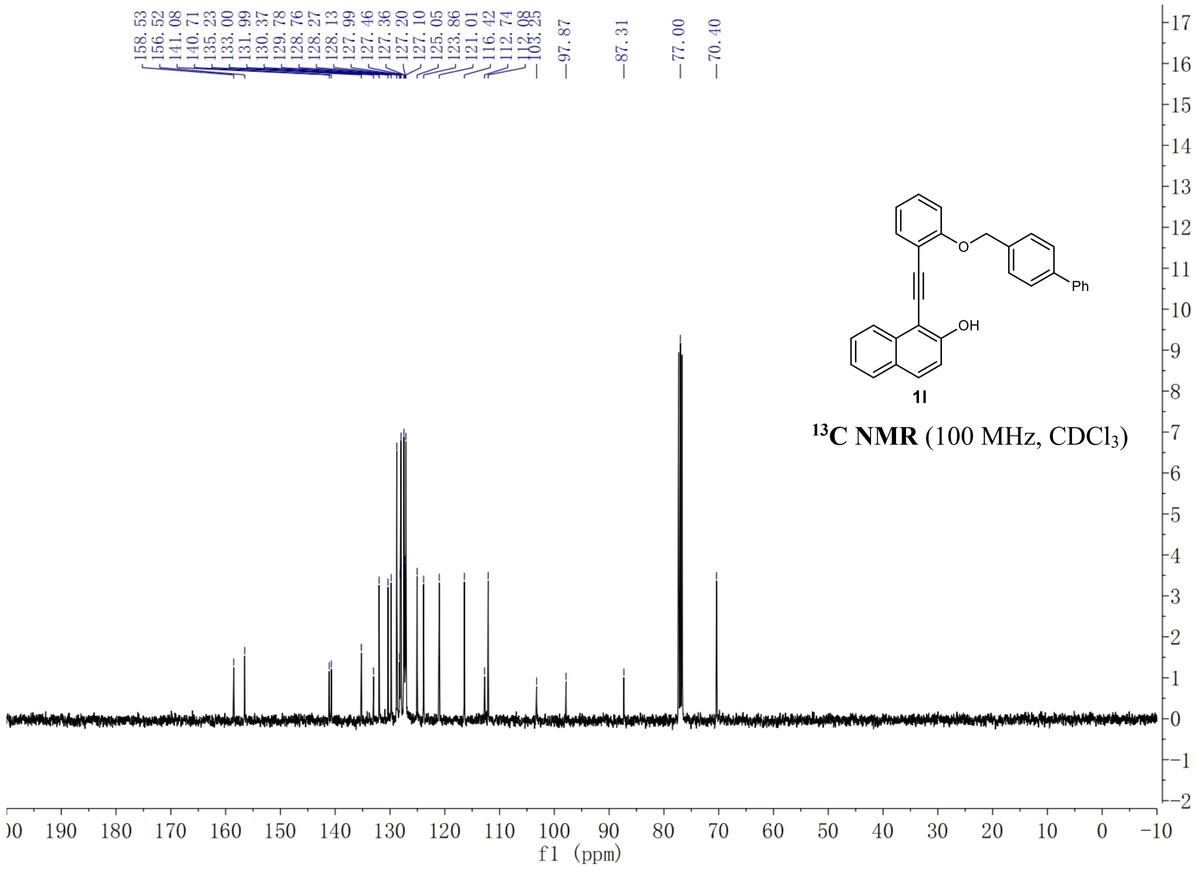

1l

^{13}C NMR (100 MHz, CDCl$_3$)

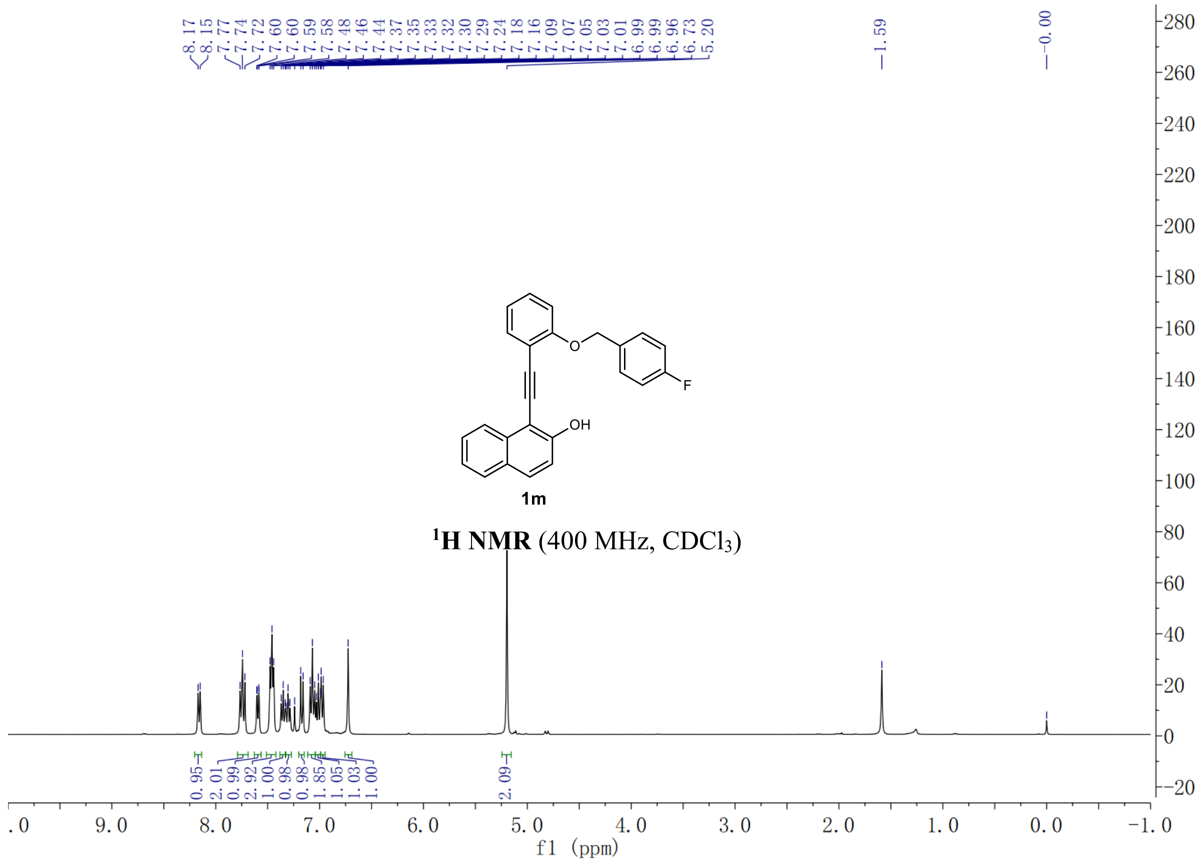

^{1}**H NMR** (400 MHz, $CDCl_3$)

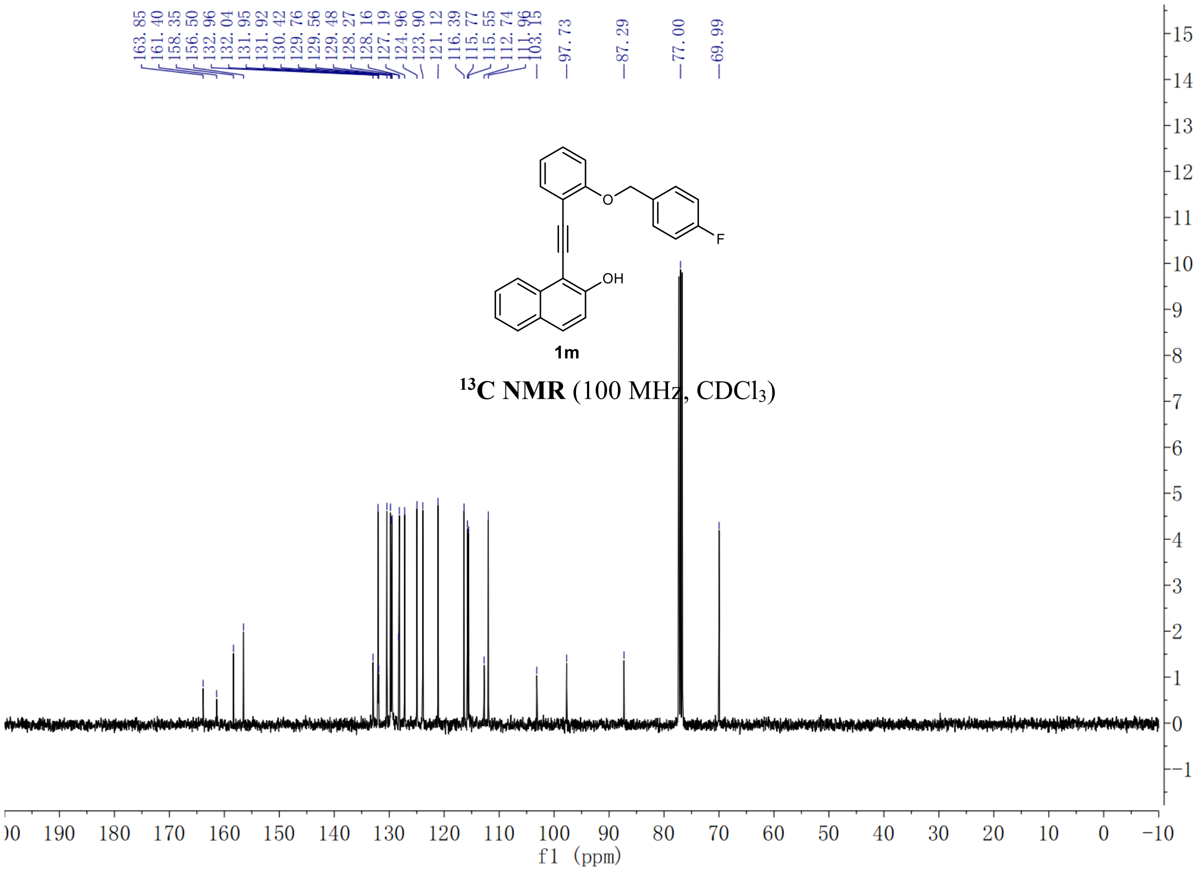

^{13}**C NMR** (100 MHz, $CDCl_3$)

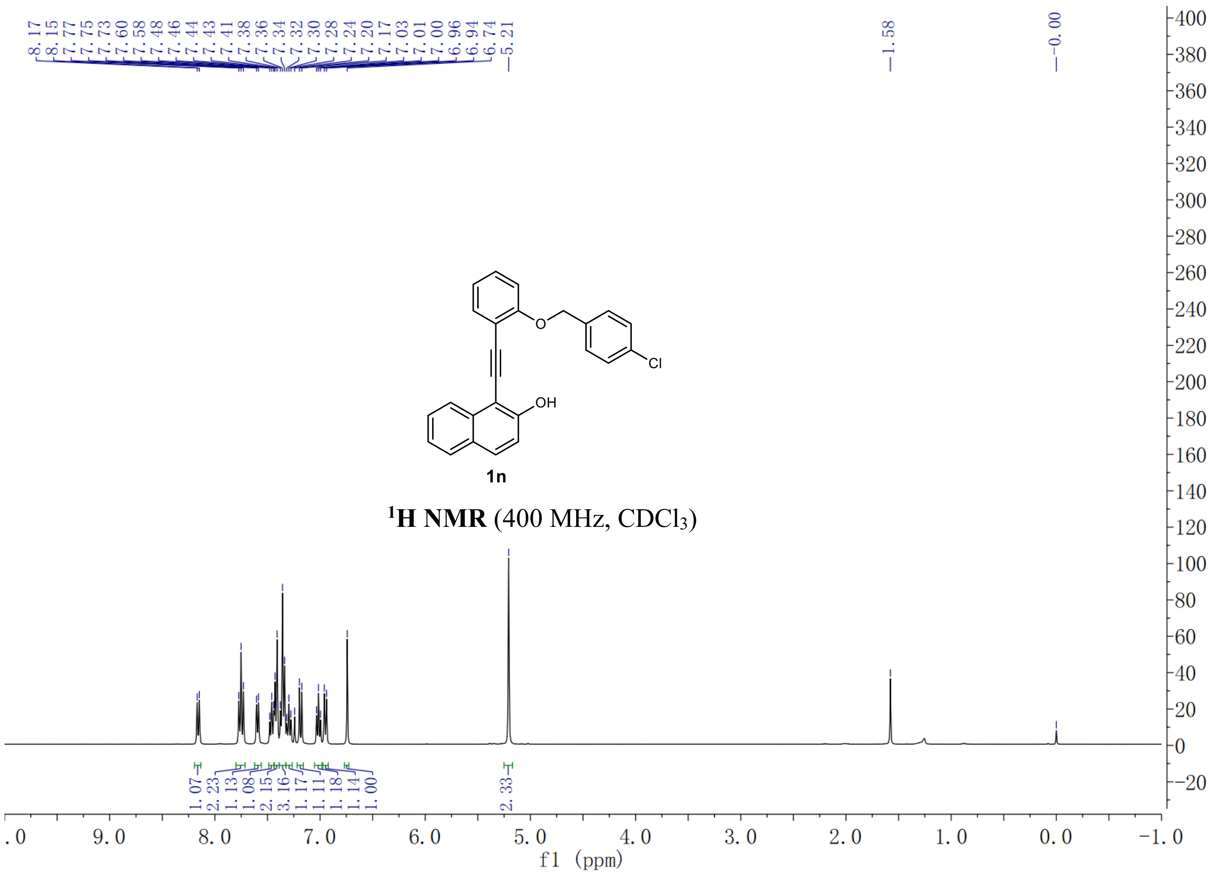
1n
^{1}H NMR (400 MHz, CDCl$_3$)
f1 (ppm)

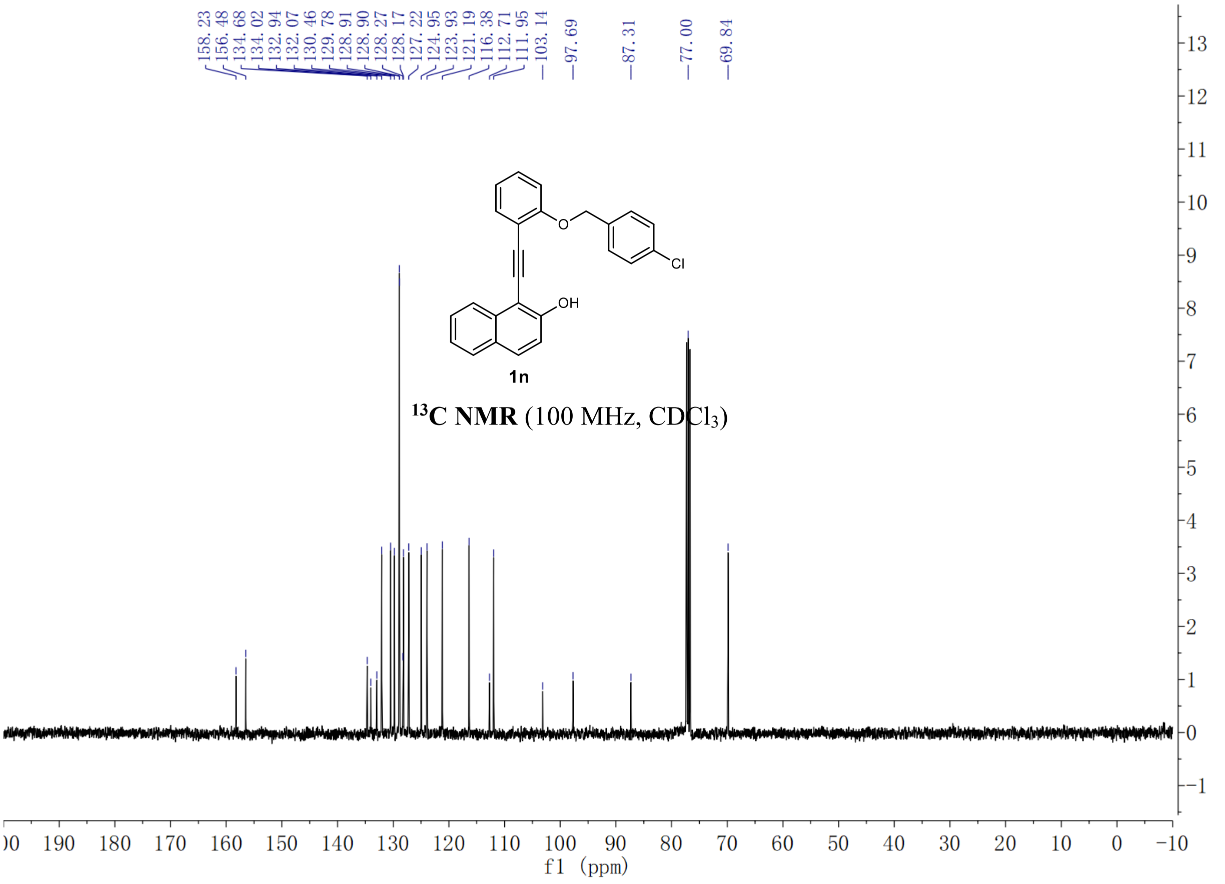
1n
^{13}C NMR (100 MHz, CDCl$_3$)
f1 (ppm)

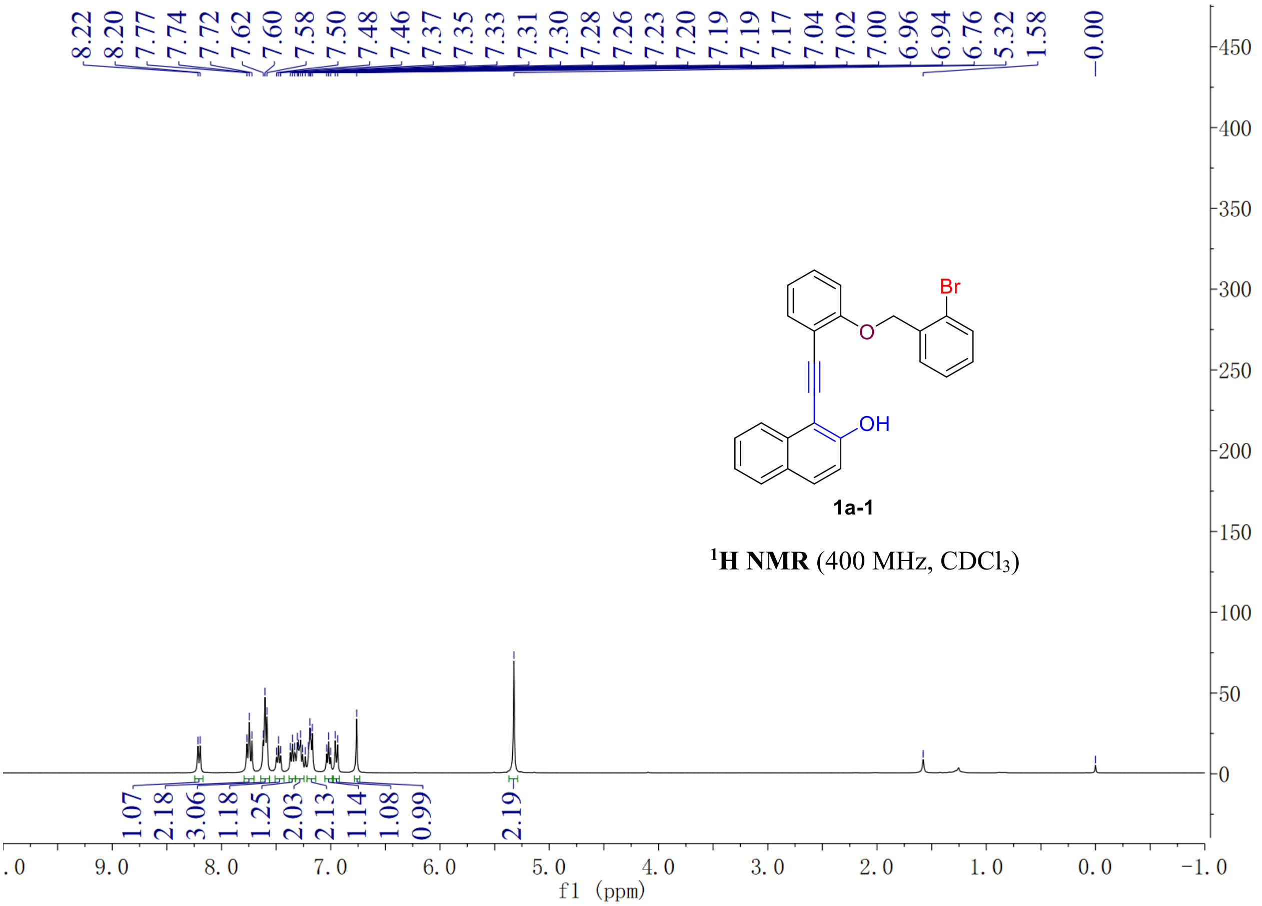

1a-1

^{1}H NMR (400 MHz, $CDCl_3$)

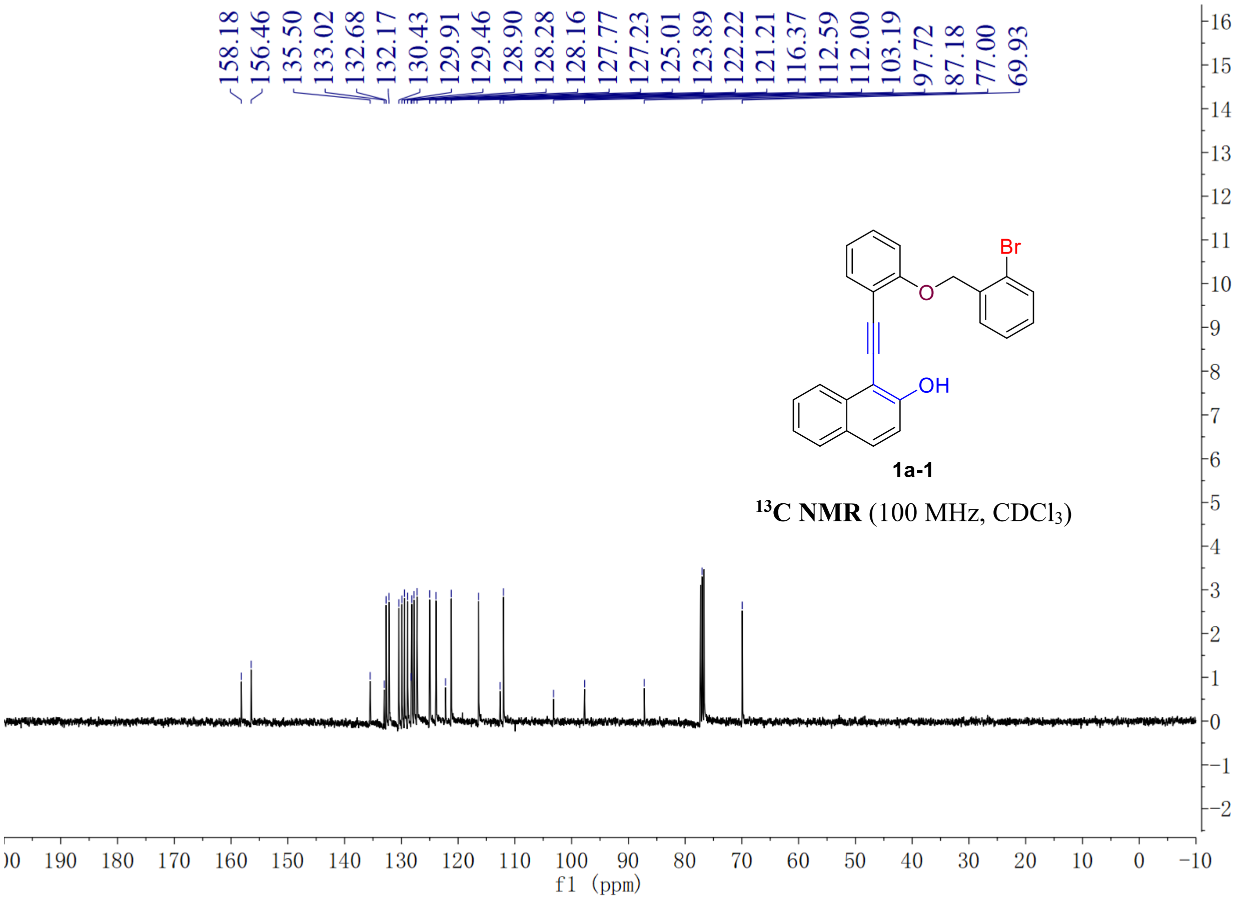

1a-1

^{13}C NMR (100 MHz, $CDCl_3$)

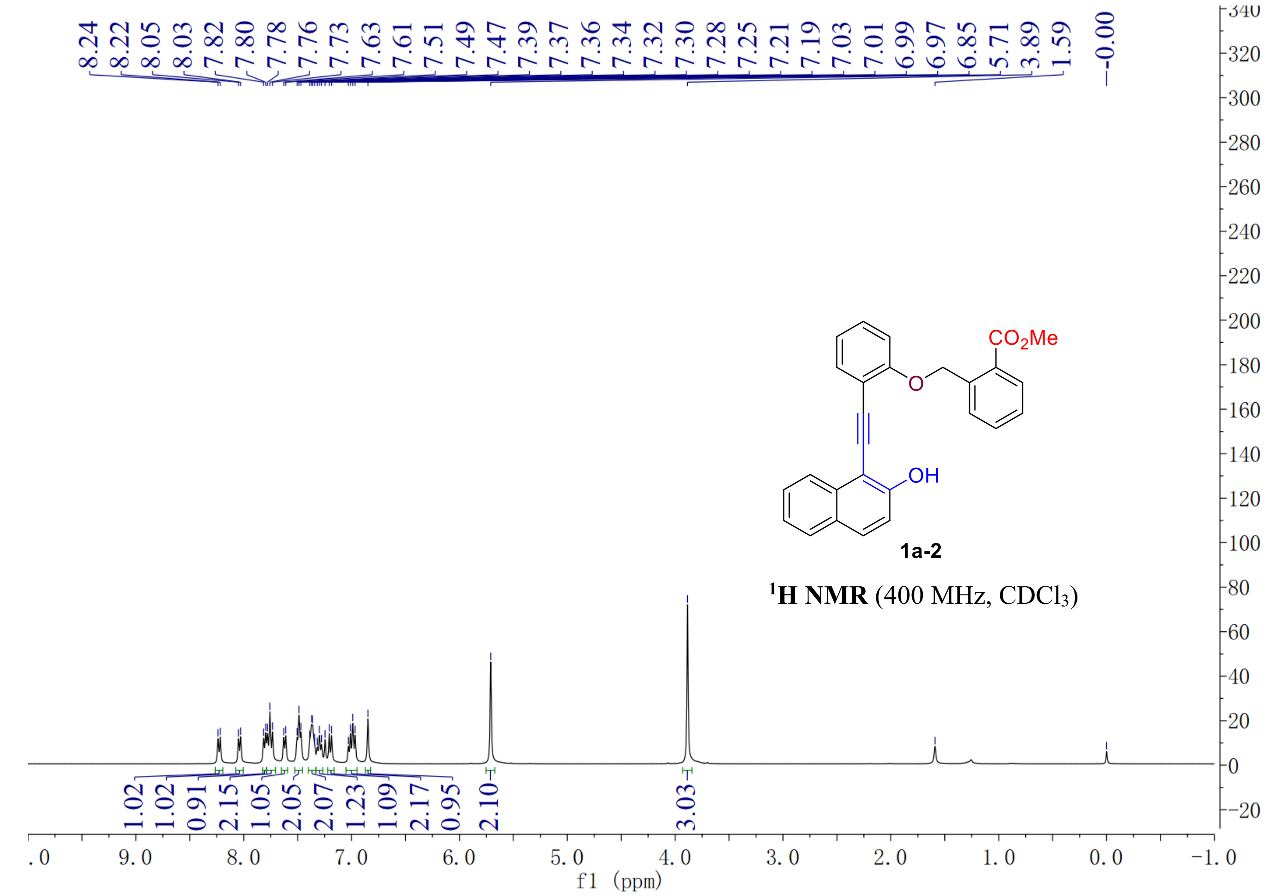
8.24 8.22 8.05 8.03 7.82 7.80 7.78 7.76 7.73 7.63 7.61 7.51 7.49 7.47 7.39 7.37 7.36 7.34 7.32 7.30 7.28 7.25 7.21 7.19 7.03 7.01 6.99 6.97 6.85 5.71 3.89 1.59 -0.00
CO2Me
O
OH
1a-2
1H NMR (400 MHz, CDCl3)
1.02 1.02 0.91 2.15 1.05 2.05 2.07 1.23 1.09 2.17 0.95 2.10 3.03
f1 (ppm)

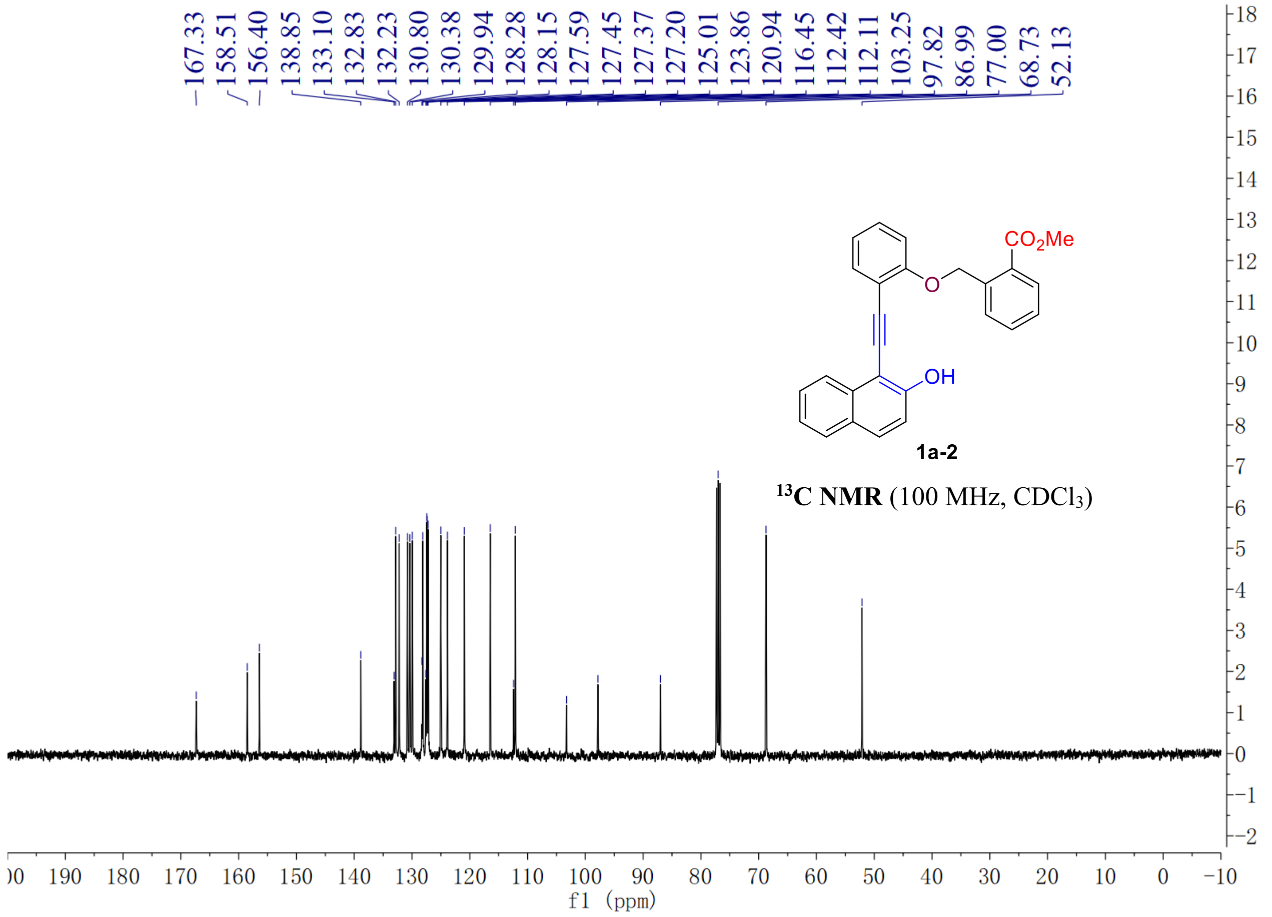
167.33 158.51 156.40 138.85 133.10 132.83 132.23 130.80 130.38 129.94 128.28 128.15 127.59 127.45 127.37 127.20 125.01 123.86 120.94 116.45 112.42 112.11 103.25 97.82 86.99 77.00 68.73 52.13
CO2Me
O
OH
1a-2
13C NMR (100 MHz, CDCl3)
f1 (ppm)

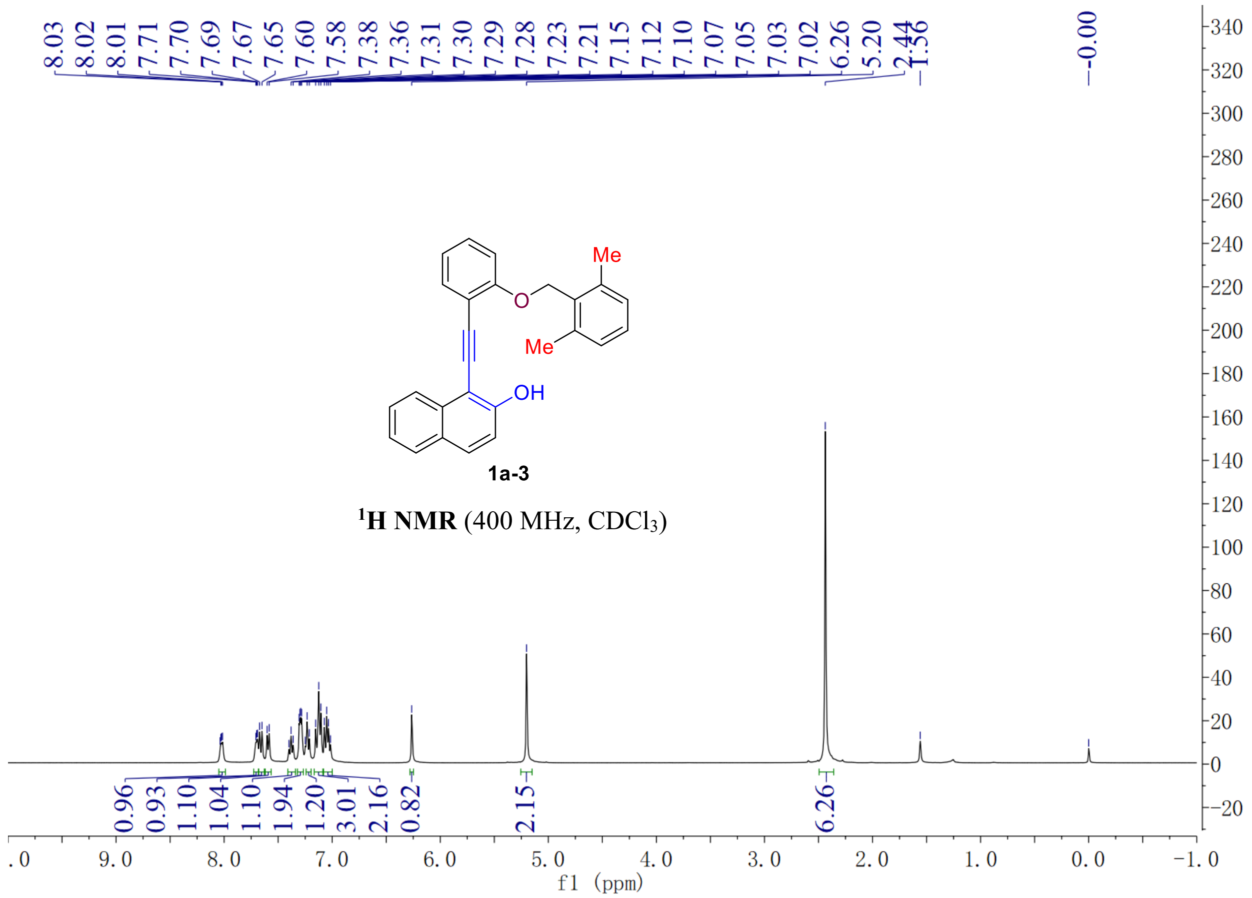
8.03
8.02
8.01
7.71
7.70
7.69
7.67
7.65
7.60
7.58
7.38
7.36
7.31
7.30
7.29
7.28
7.23
7.21
7.15
7.12
7.10
7.07
7.05
7.03
7.02
6.26
5.20
2.44
1.56
-0.00
Me
O
Me
OH
1a-3
¹H NMR (400 MHz, CDCl₃)
0.96
0.93
1.10
1.04
1.10
1.94
1.20
3.01
2.16
0.82
2.15
6.26
f1 (ppm)

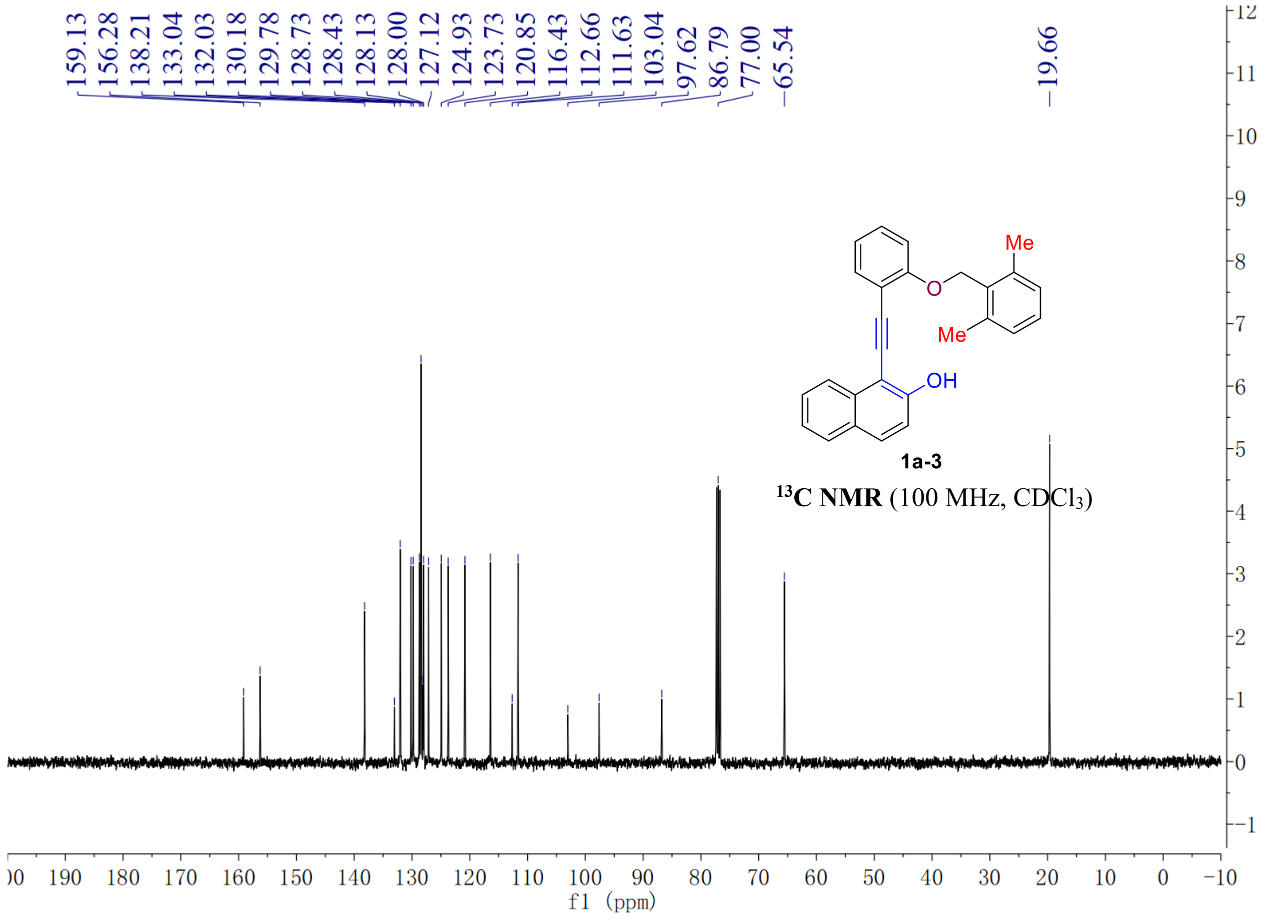
159.13
156.28
138.21
133.04
132.03
130.18
129.78
128.73
128.43
128.13
128.00
127.12
124.93
123.73
120.85
116.43
112.66
111.63
103.04
97.62
86.79
77.00
65.54
19.66
Me
O
Me
OH
1a-3
¹³C NMR (100 MHz, CDCl₃)
f1 (ppm)

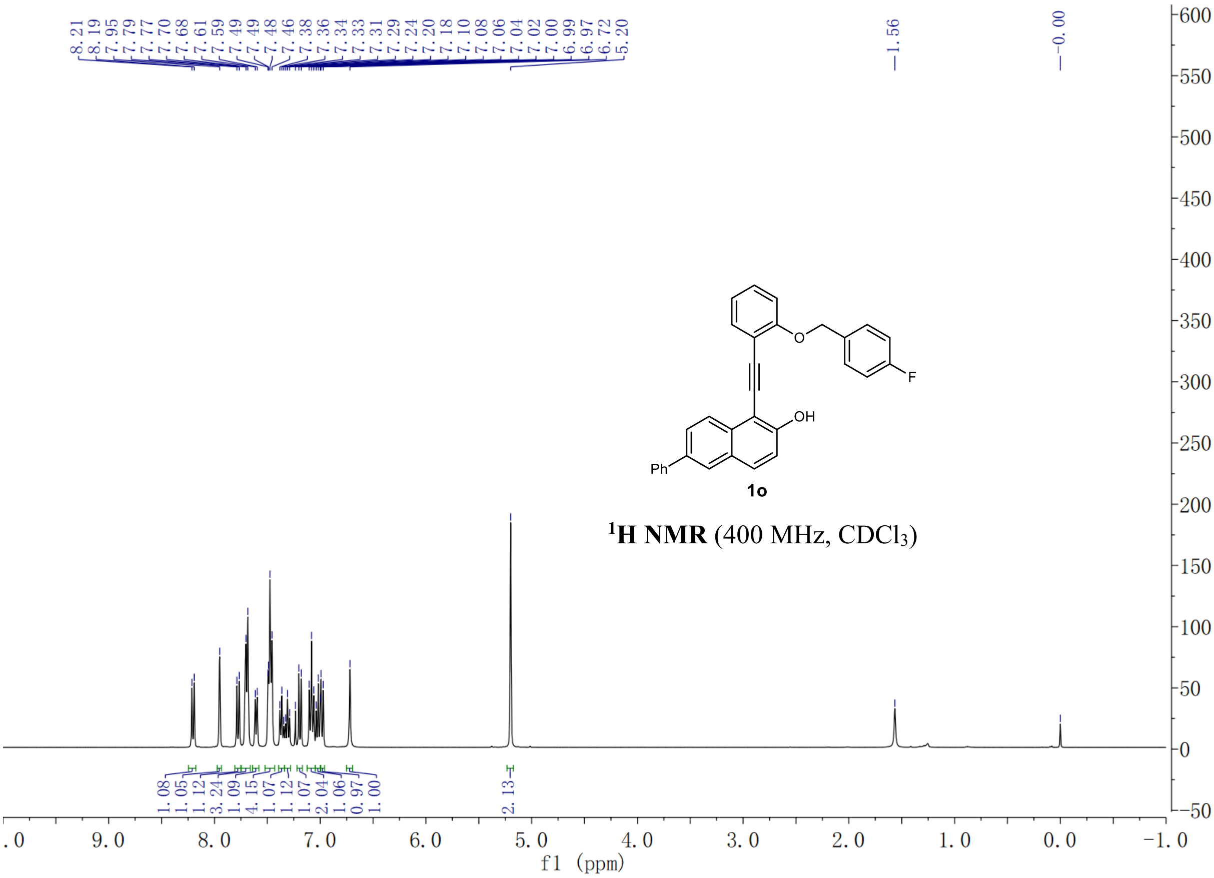
1o
^{1}H NMR (400 MHz, CDCl$_3$)

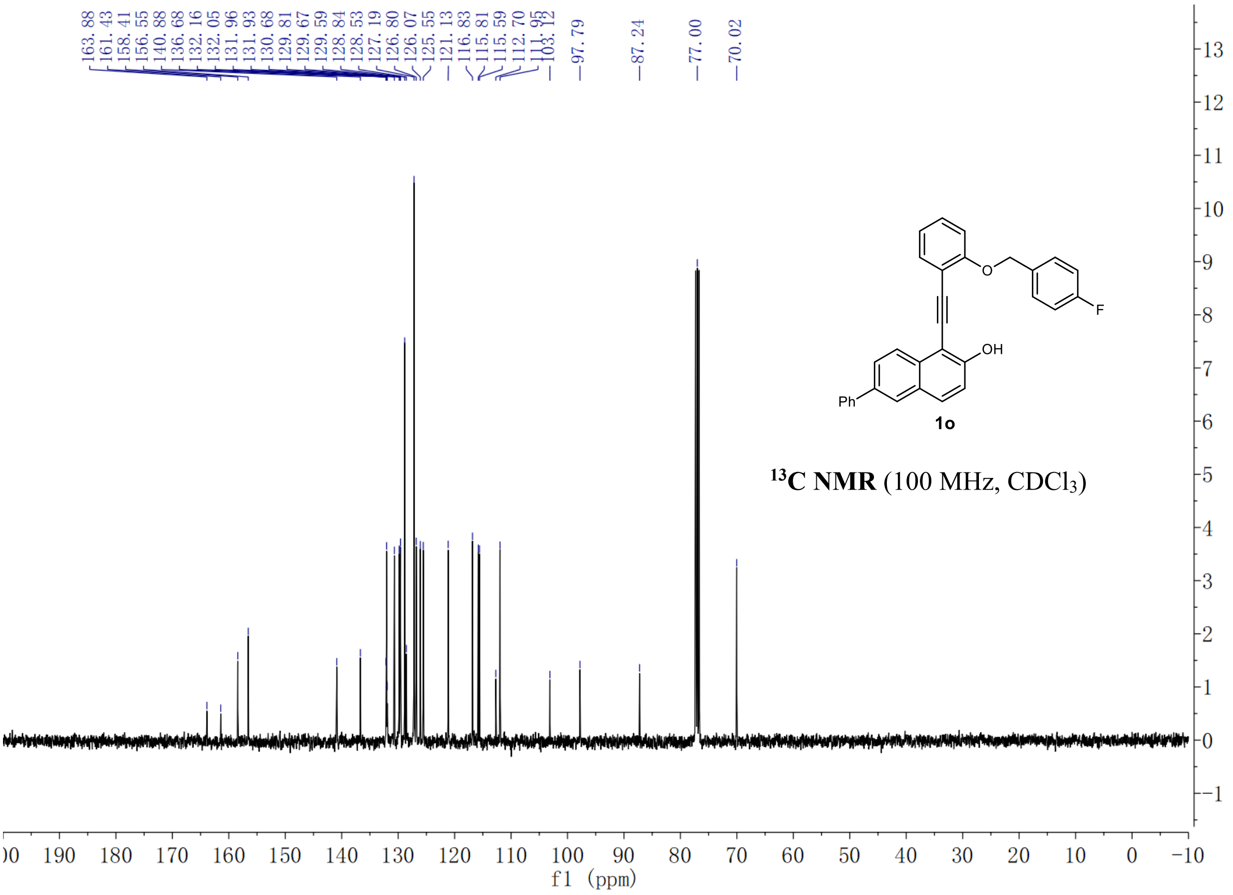
1o
^{13}C NMR (100 MHz, CDCl$_3$)

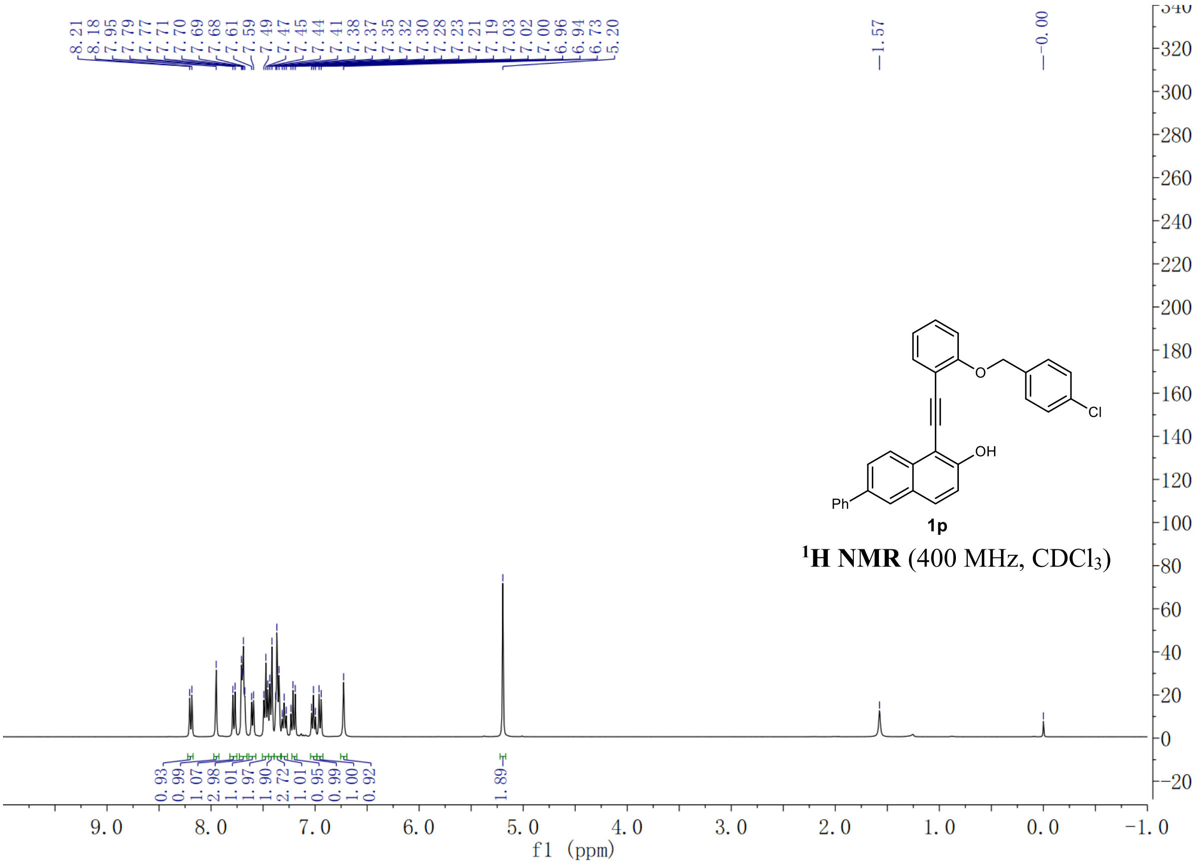

^{1}H NMR (400 MHz, $CDCl_3$)

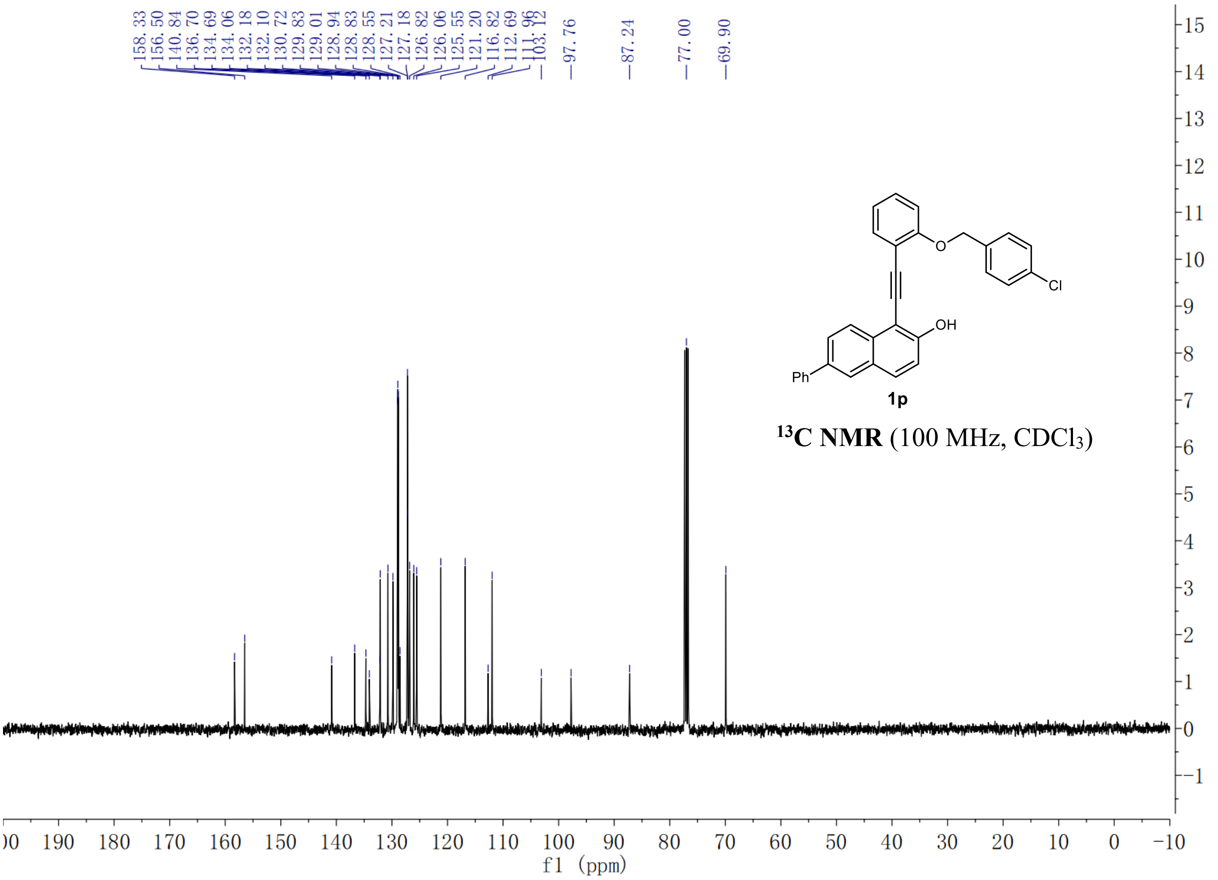

^{13}C NMR (100 MHz, $CDCl_3$)

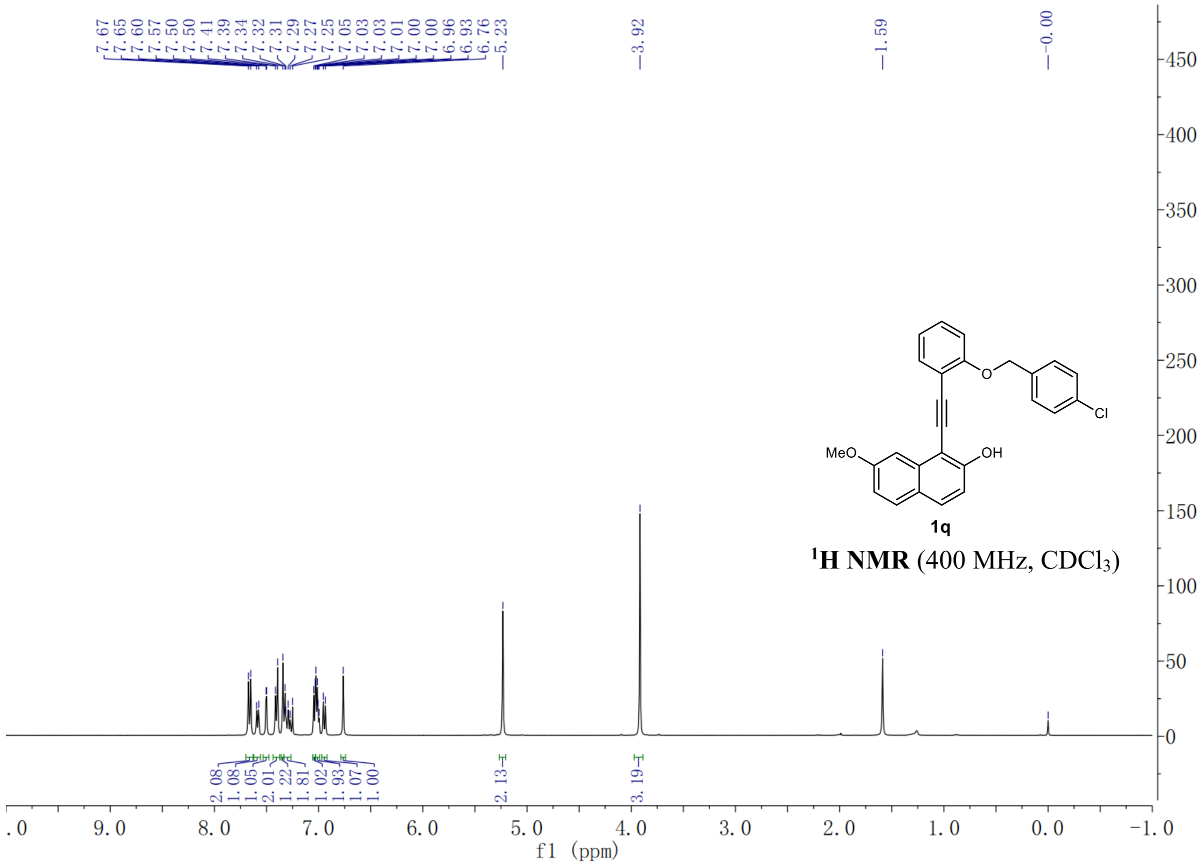
7.67
7.65
7.60
7.57
7.50
7.50
7.41
7.39
7.34
7.32
7.31
7.29
7.27
7.25
7.05
7.03
7.03
7.01
7.00
7.00
6.96
6.93
6.76
5.23
3.92
1.59
0.00
MeO
OH
O
Cl
1q
¹H NMR (400 MHz, CDCl3)
2.08
1.08
1.05
2.01
1.22
1.81
1.02
1.93
1.07
1.00
2.13
3.19
f1 (ppm)

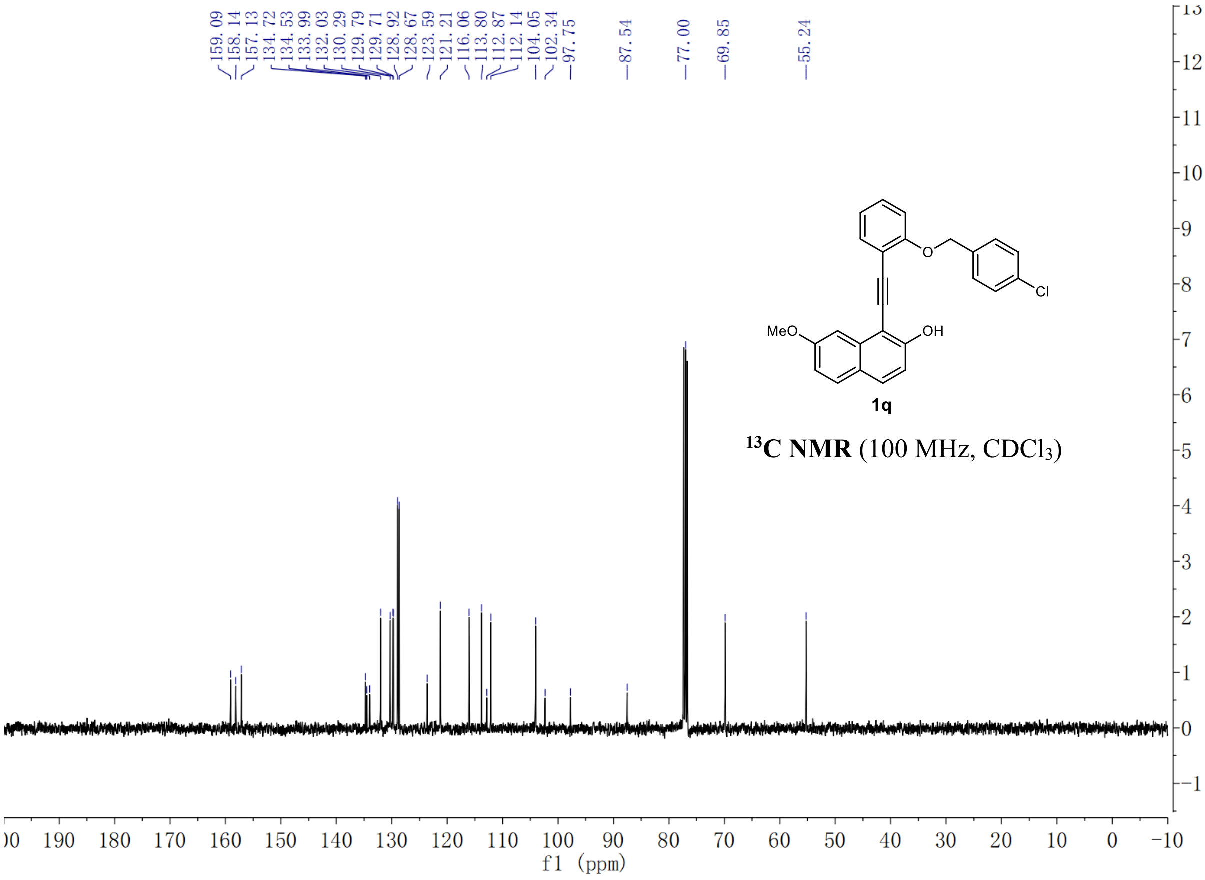
159.09
158.14
157.13
134.72
134.53
133.99
132.03
130.29
129.79
129.71
128.92
128.67
123.59
121.21
116.06
113.80
112.87
112.14
104.05
102.34
97.75
87.54
77.00
69.85
55.24
MeO
OH
O
Cl
1q
¹³C NMR (100 MHz, CDCl3)
f1 (ppm)

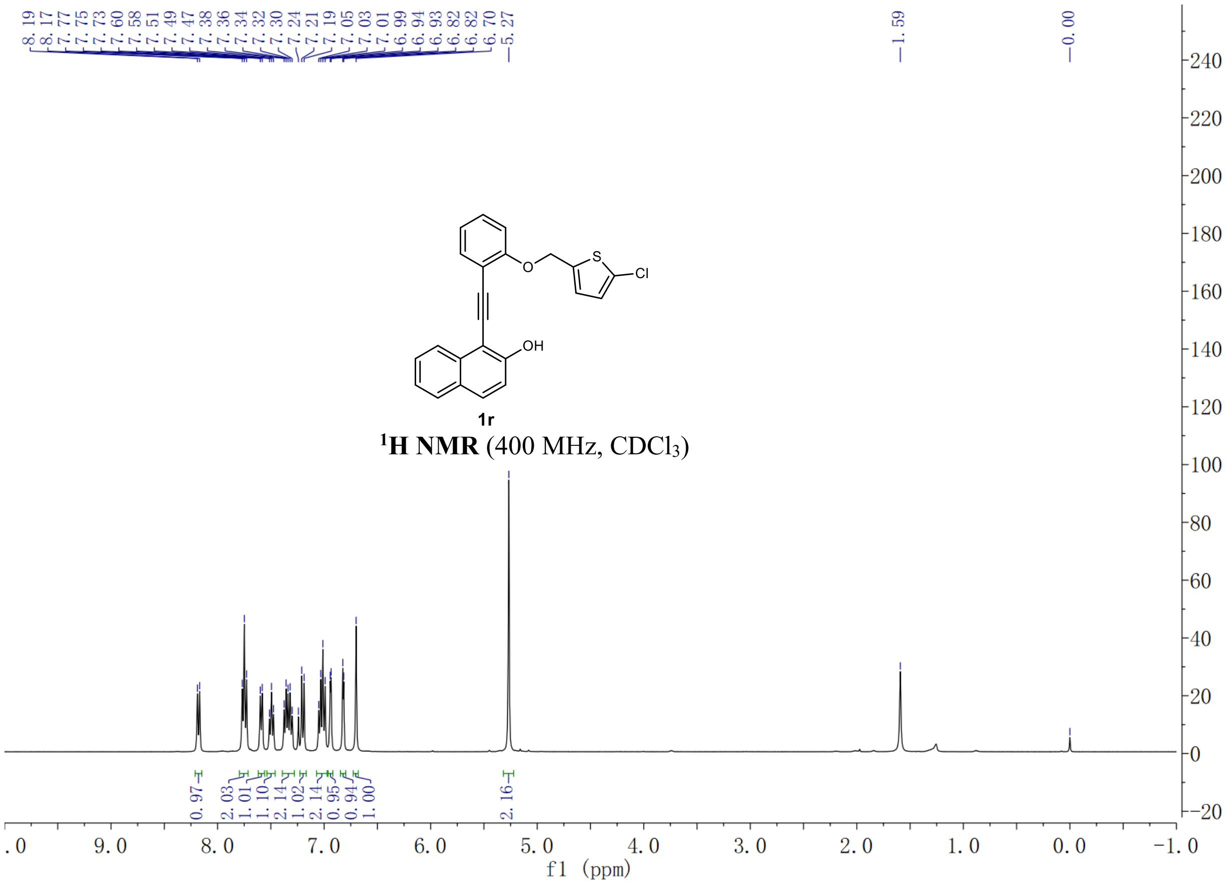

^{1}H NMR (400 MHz, $CDCl_3$)

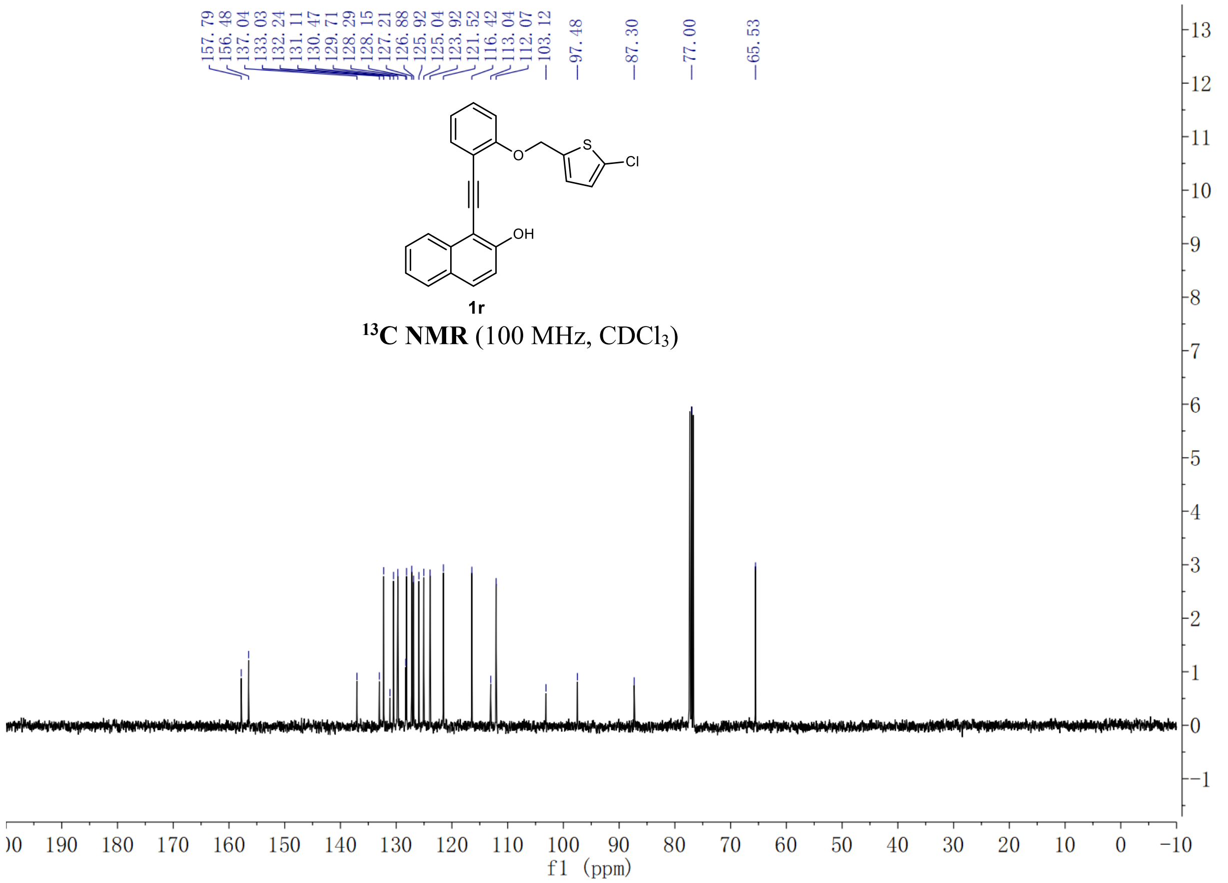

^{13}C NMR (100 MHz, $CDCl_3$)

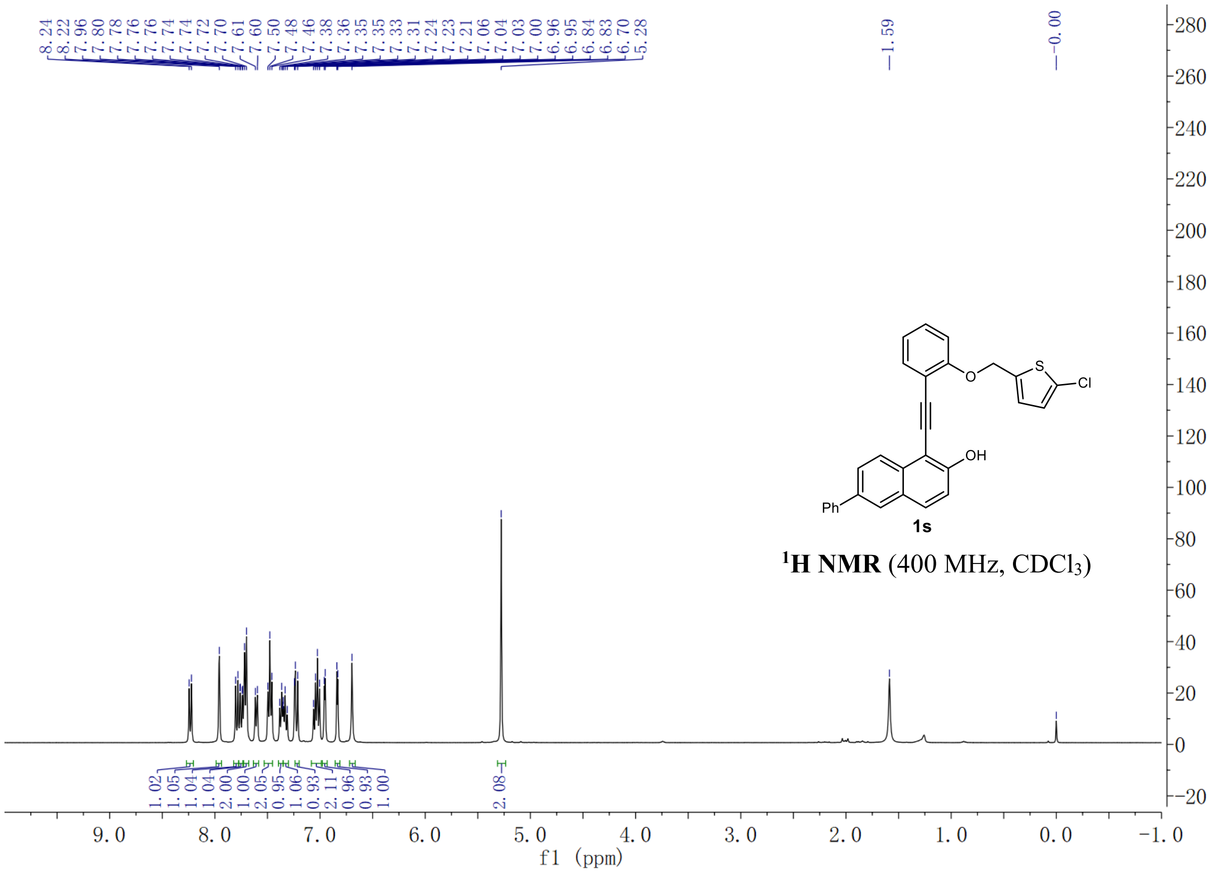

1s
^{1}H NMR (400 MHz, CDCl$_3$)
f1 (ppm)

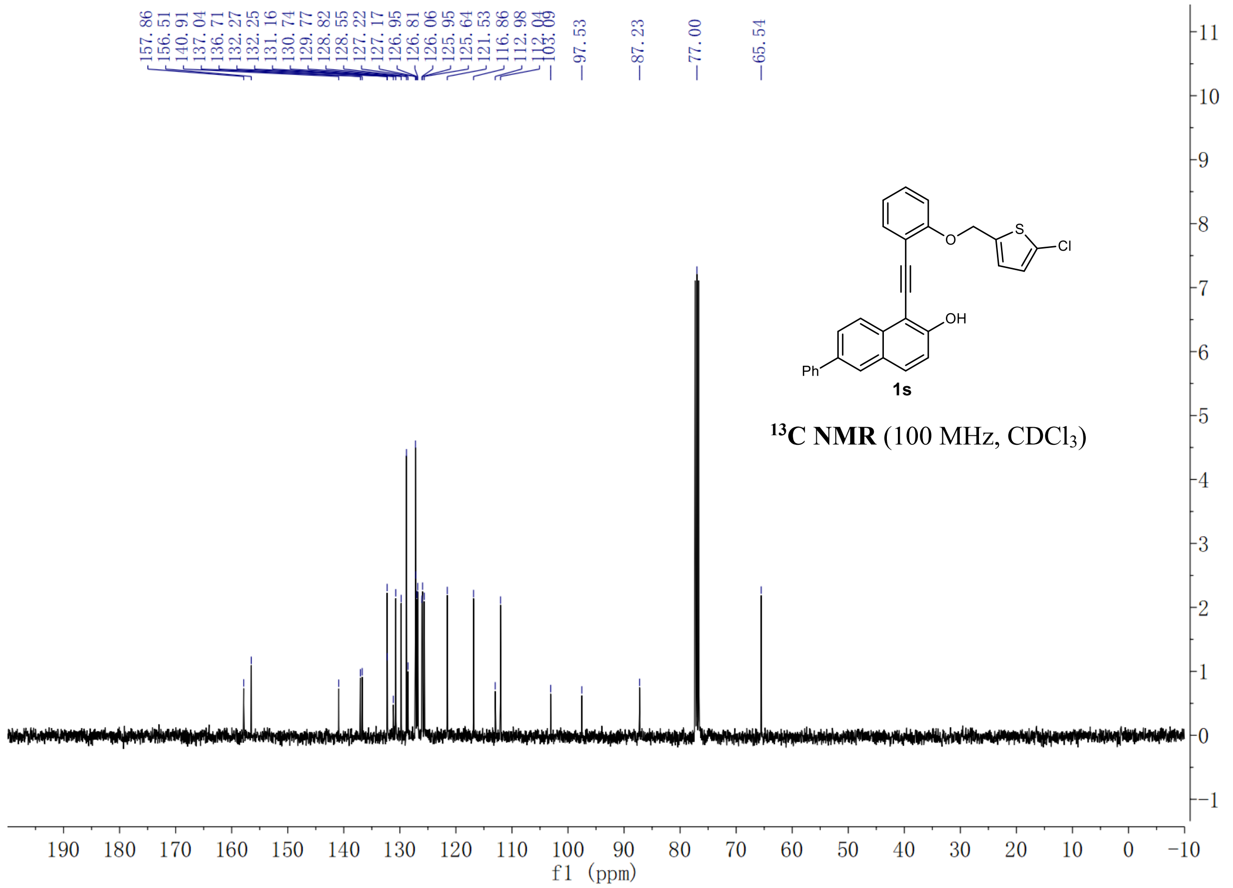

1s
^{13}C NMR (100 MHz, CDCl$_3$)
f1 (ppm)

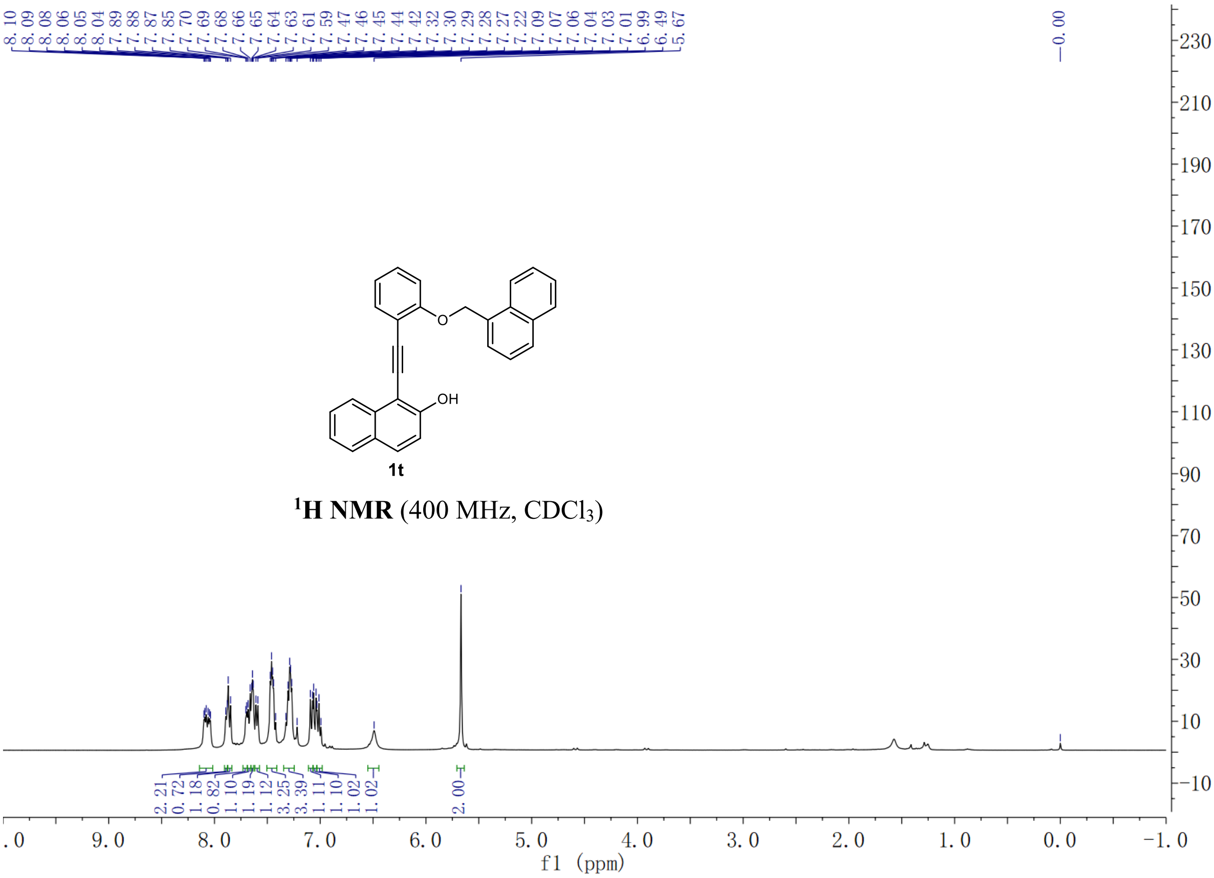
1t
¹H NMR (400 MHz, CDCl₃)
f1 (ppm)

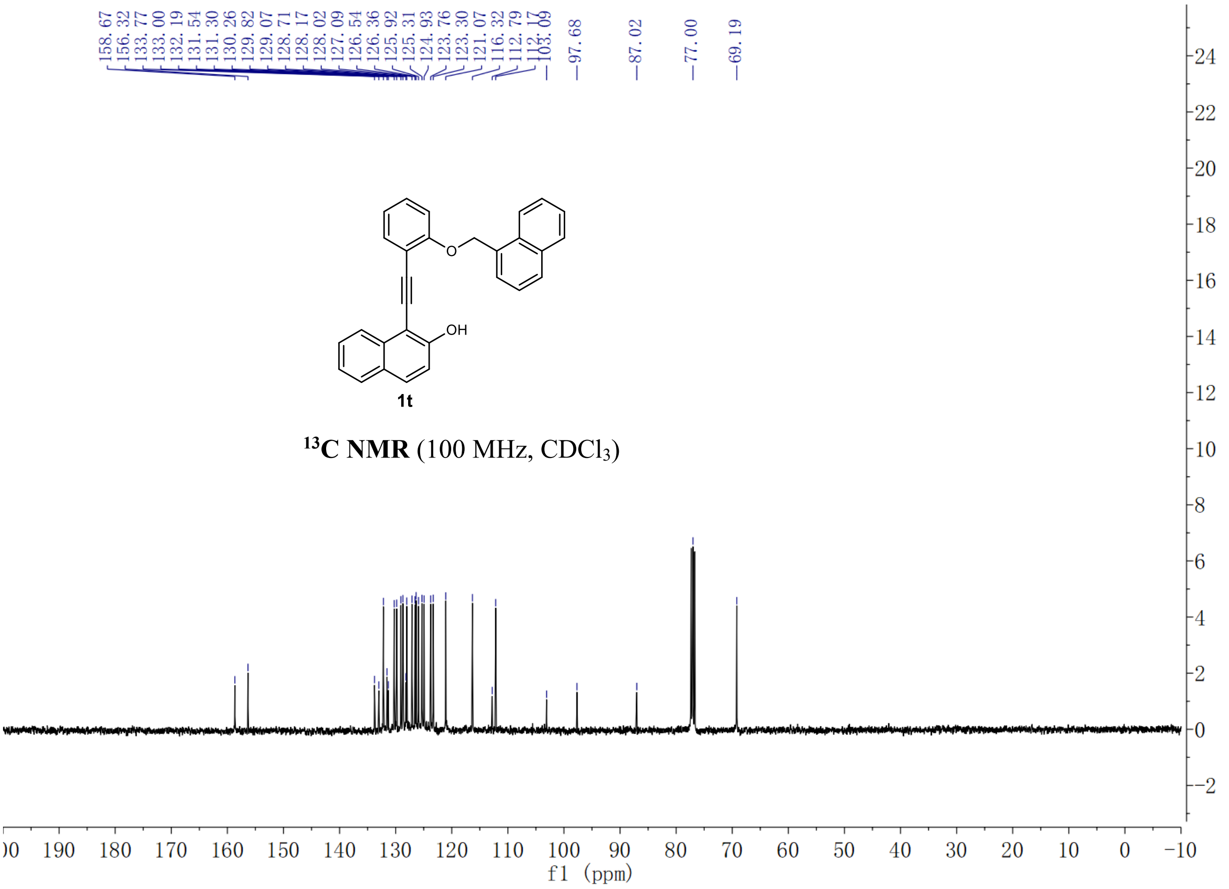
1t
¹³C NMR (100 MHz, CDCl₃)
f1 (ppm)

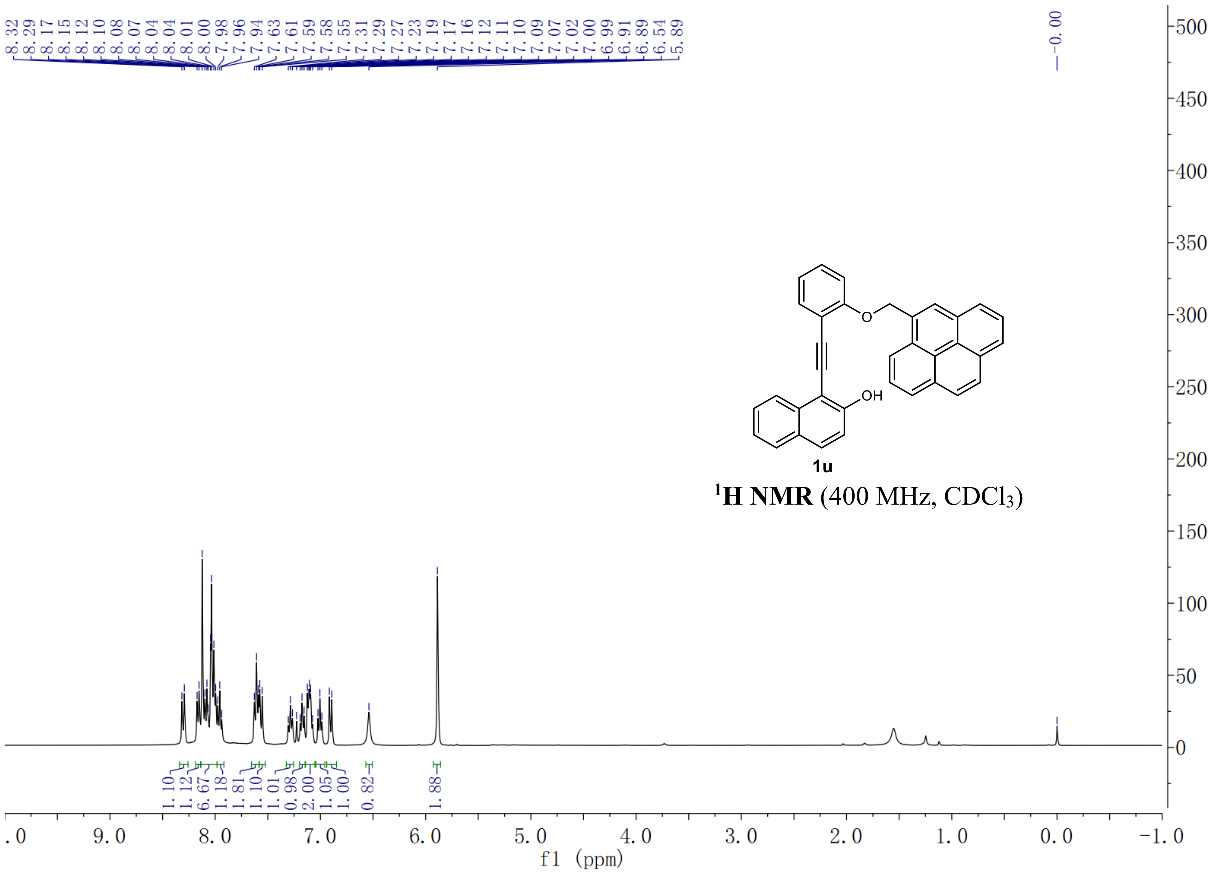

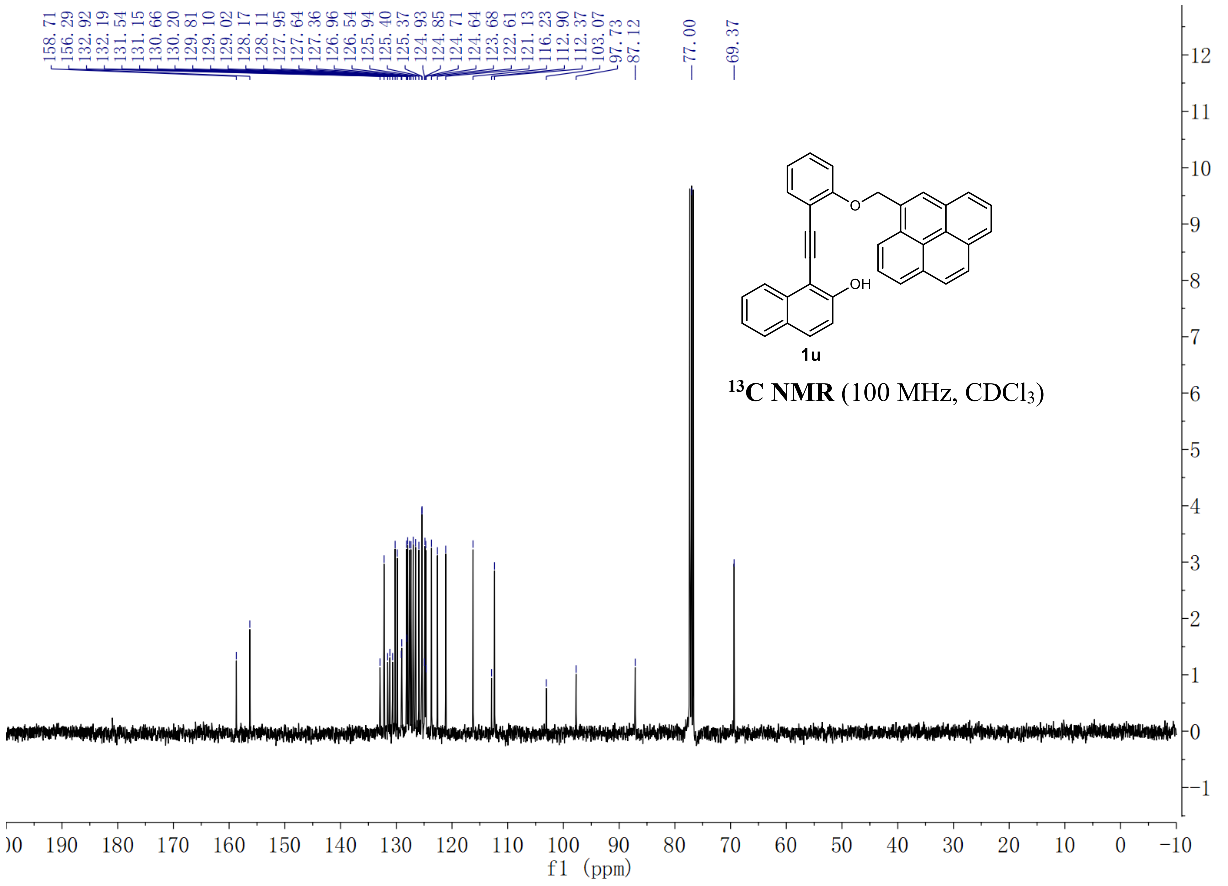

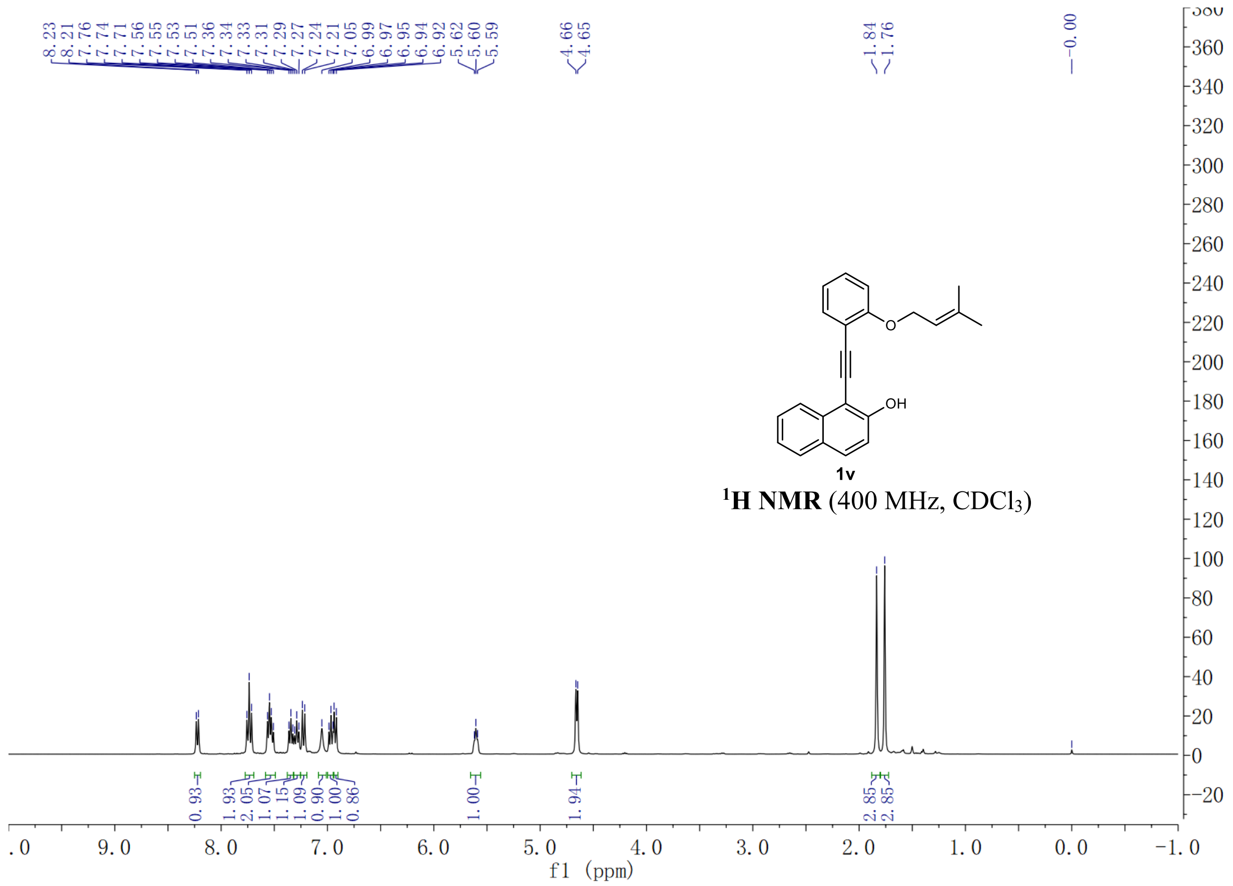

1v
^{1}H NMR (400 MHz, CDCl$_3$)
f1 (ppm)

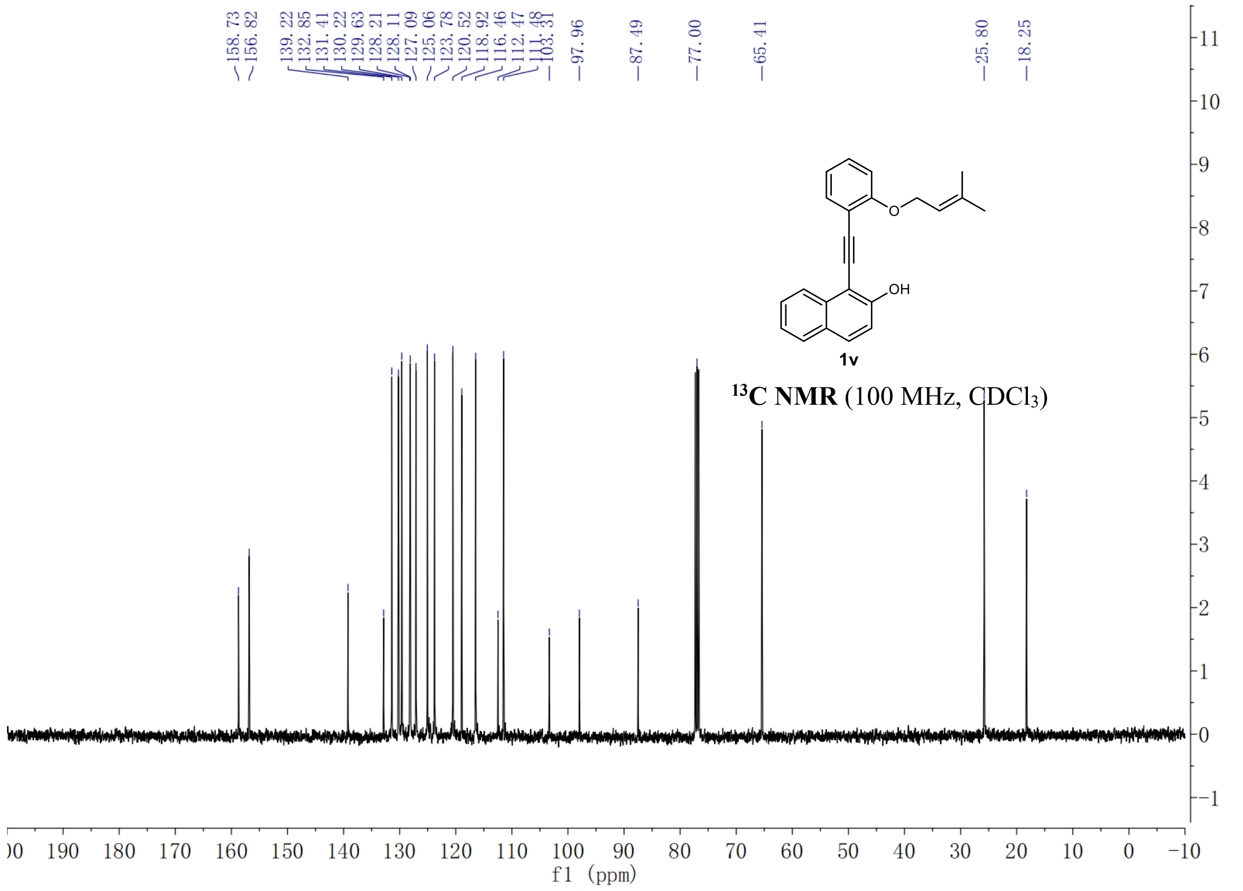

1v
^{13}C NMR (100 MHz, CDCl$_3$)
f1 (ppm)

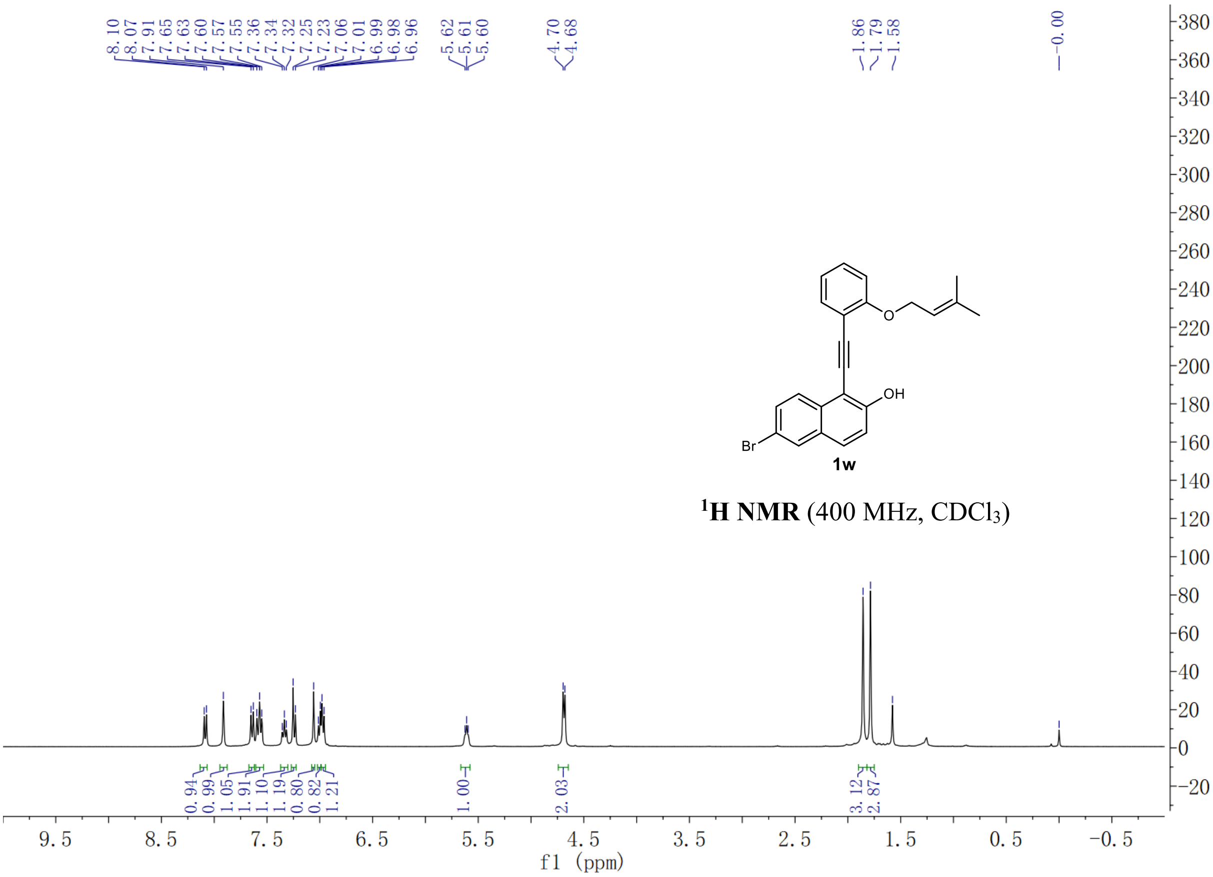

^{1}**H NMR** (400 MHz, $CDCl_3$)

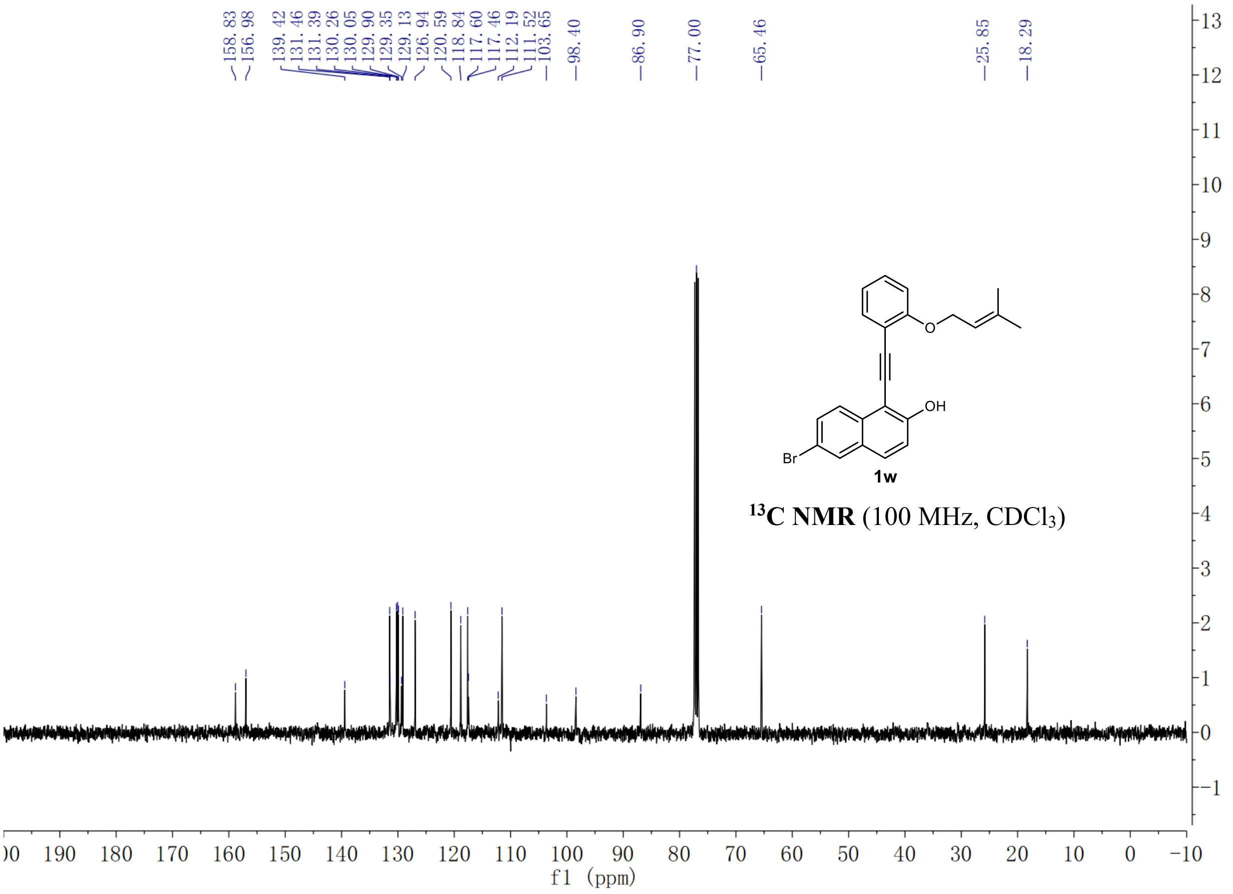

^{13}**C NMR** (100 MHz, $CDCl_3$)

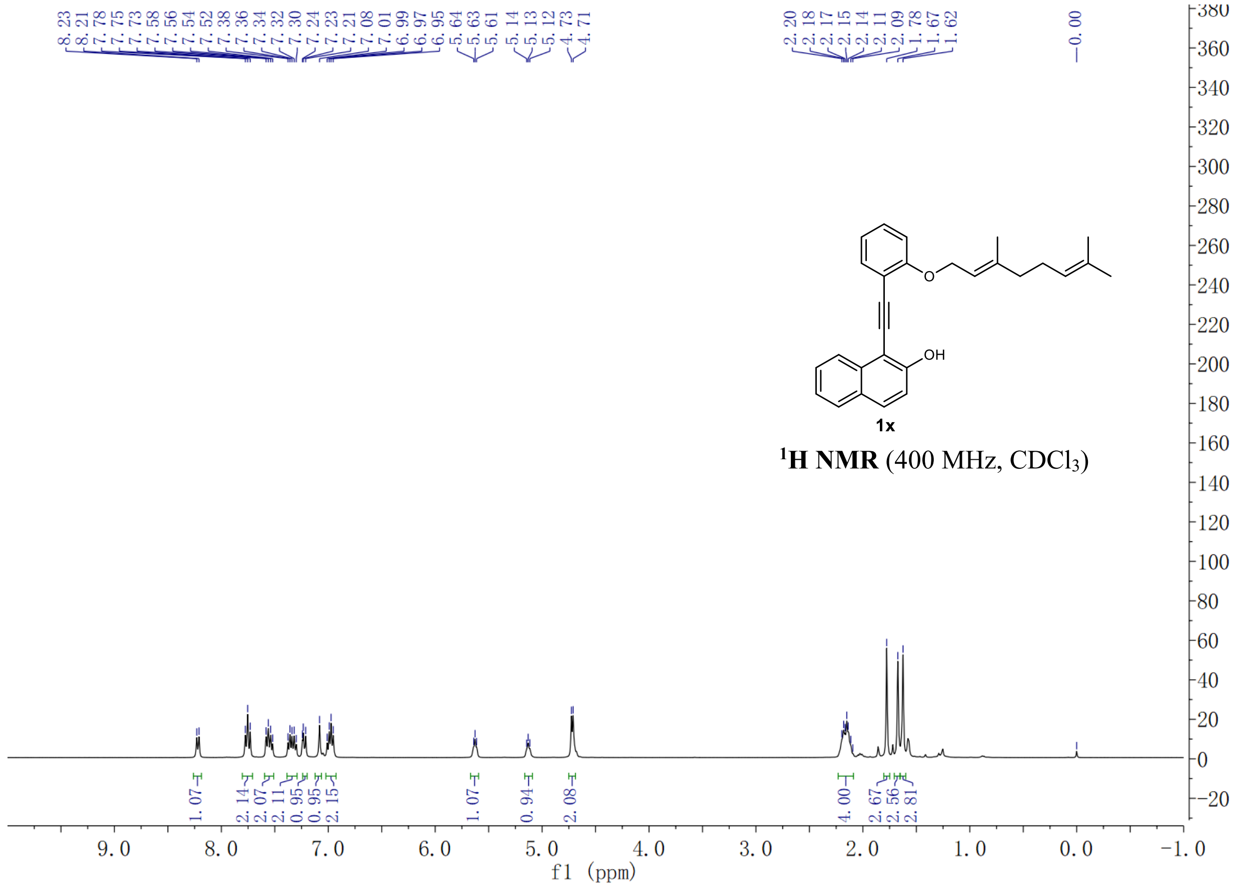

^{1}H NMR (400 MHz, $CDCl_3$)

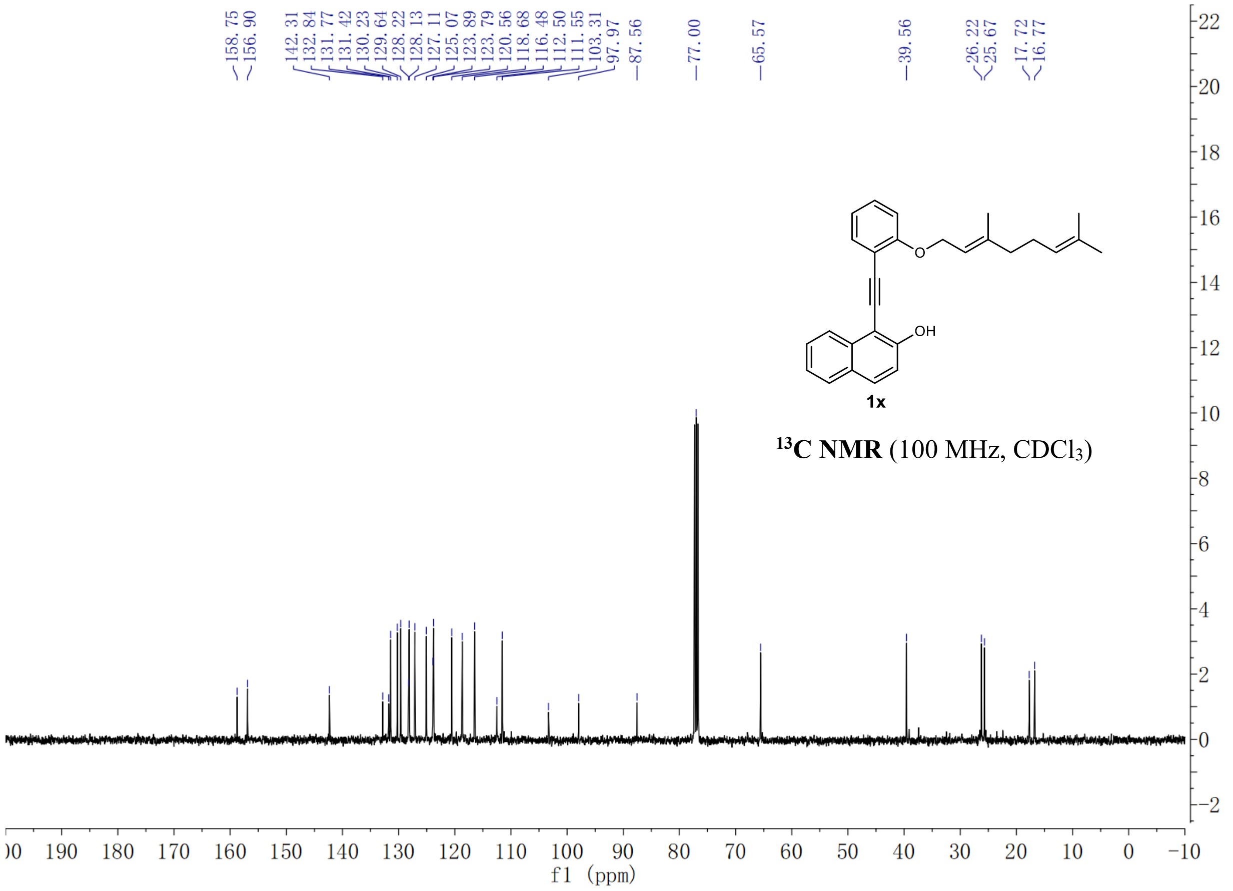

^{13}C NMR (100 MHz, $CDCl_3$)

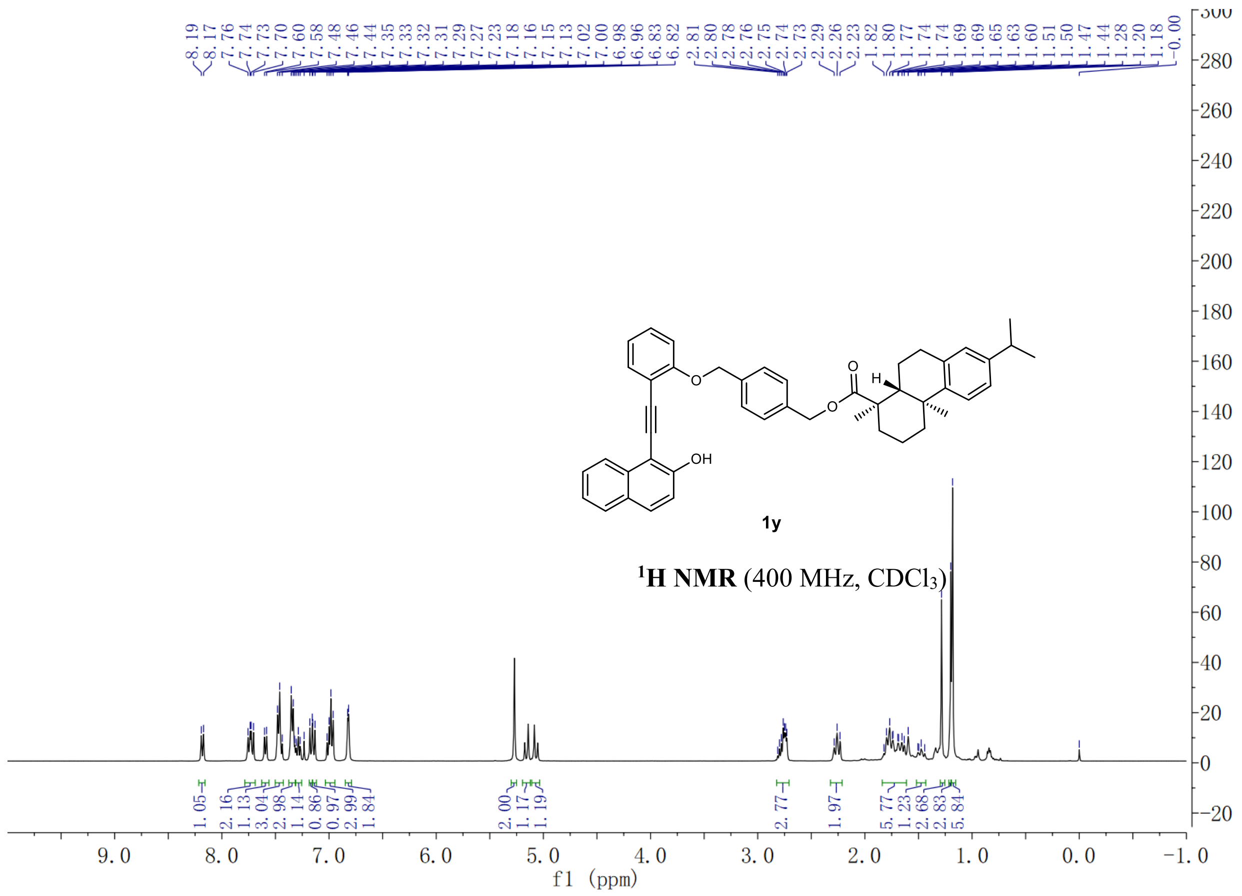
1y
^{1}H NMR (400 MHz, CDCl$_3$)
f1 (ppm)

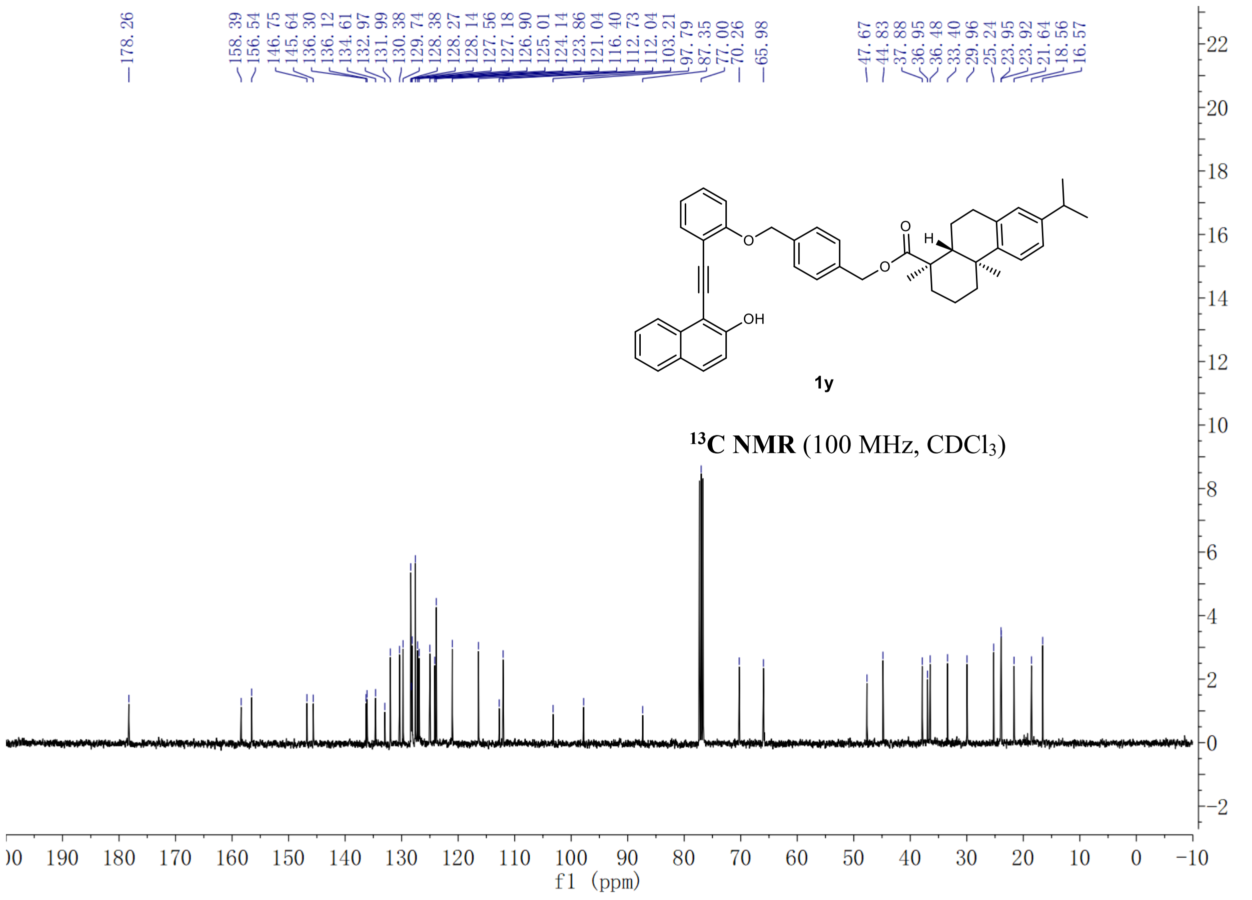
1y
^{13}C NMR (100 MHz, CDCl$_3$)
f1 (ppm)

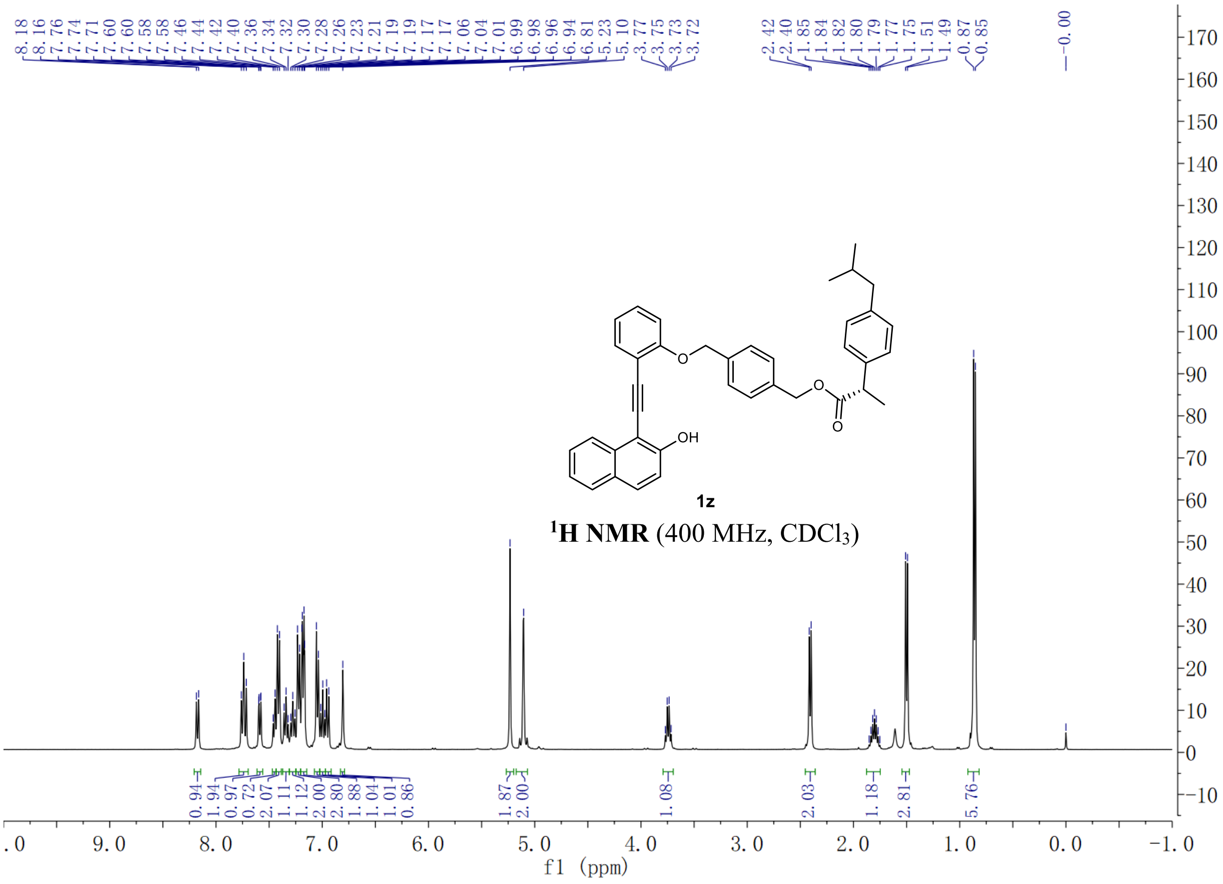
1z
^{1}H NMR (400 MHz, CDCl$_3$)
f1 (ppm)

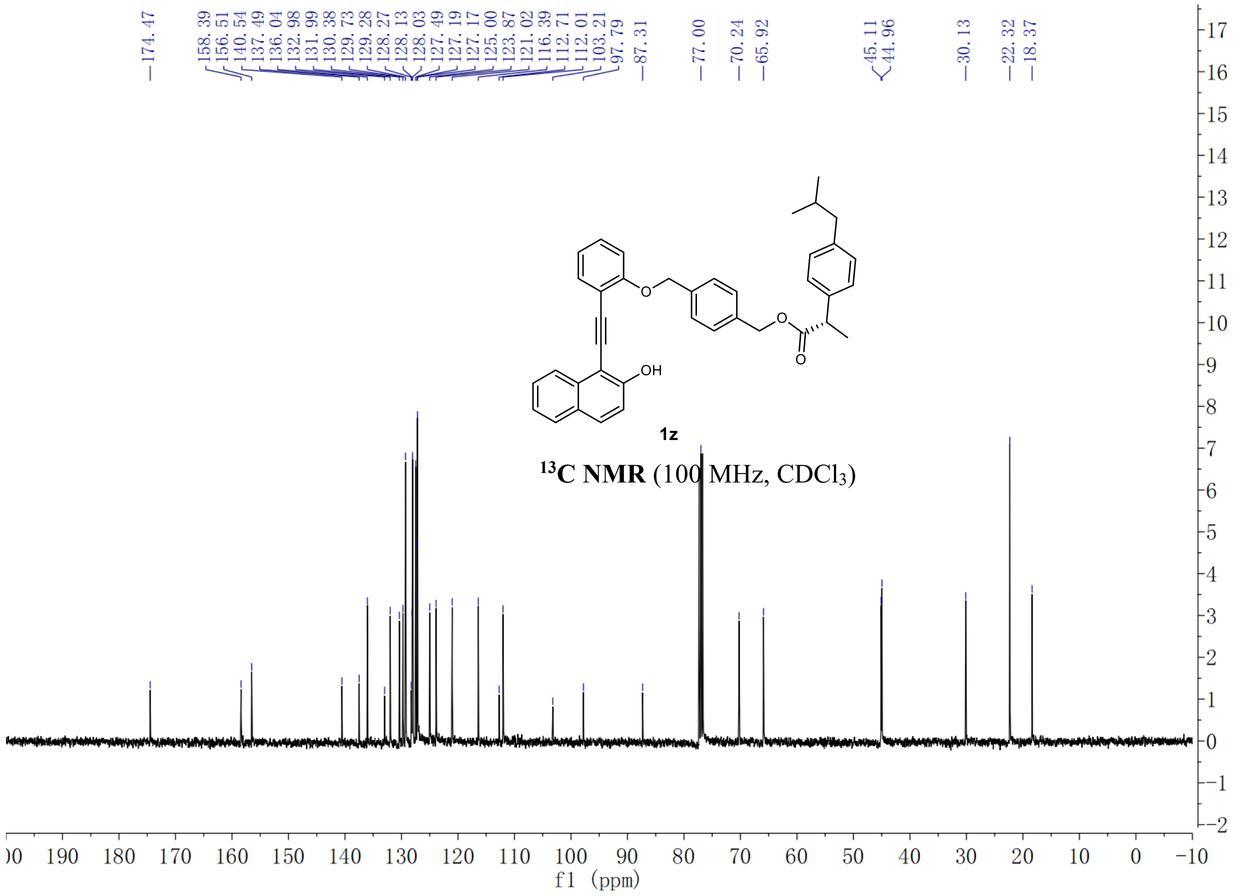
1z
^{13}C NMR (100 MHz, CDCl$_3$)
f1 (ppm)

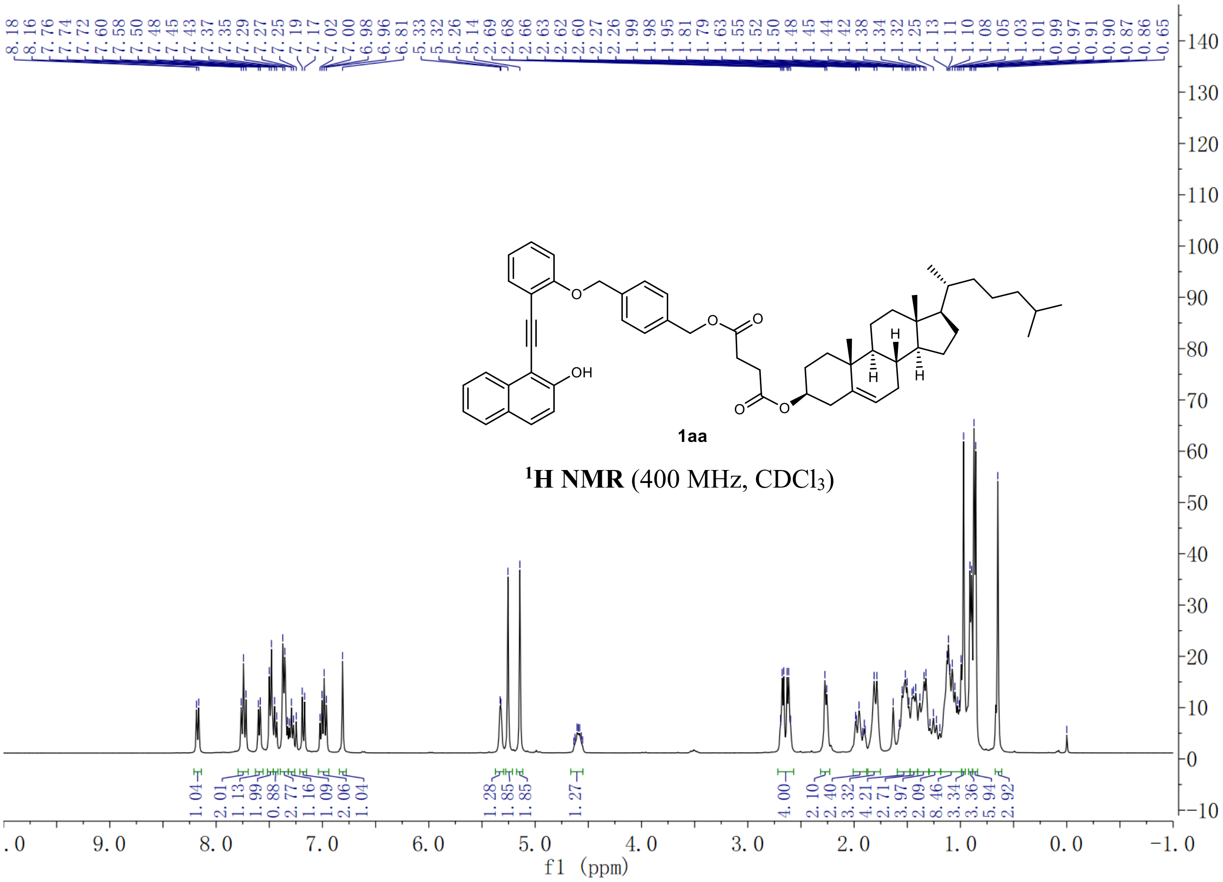
1aa
¹H NMR (400 MHz, CDCl₃)
f1 (ppm)

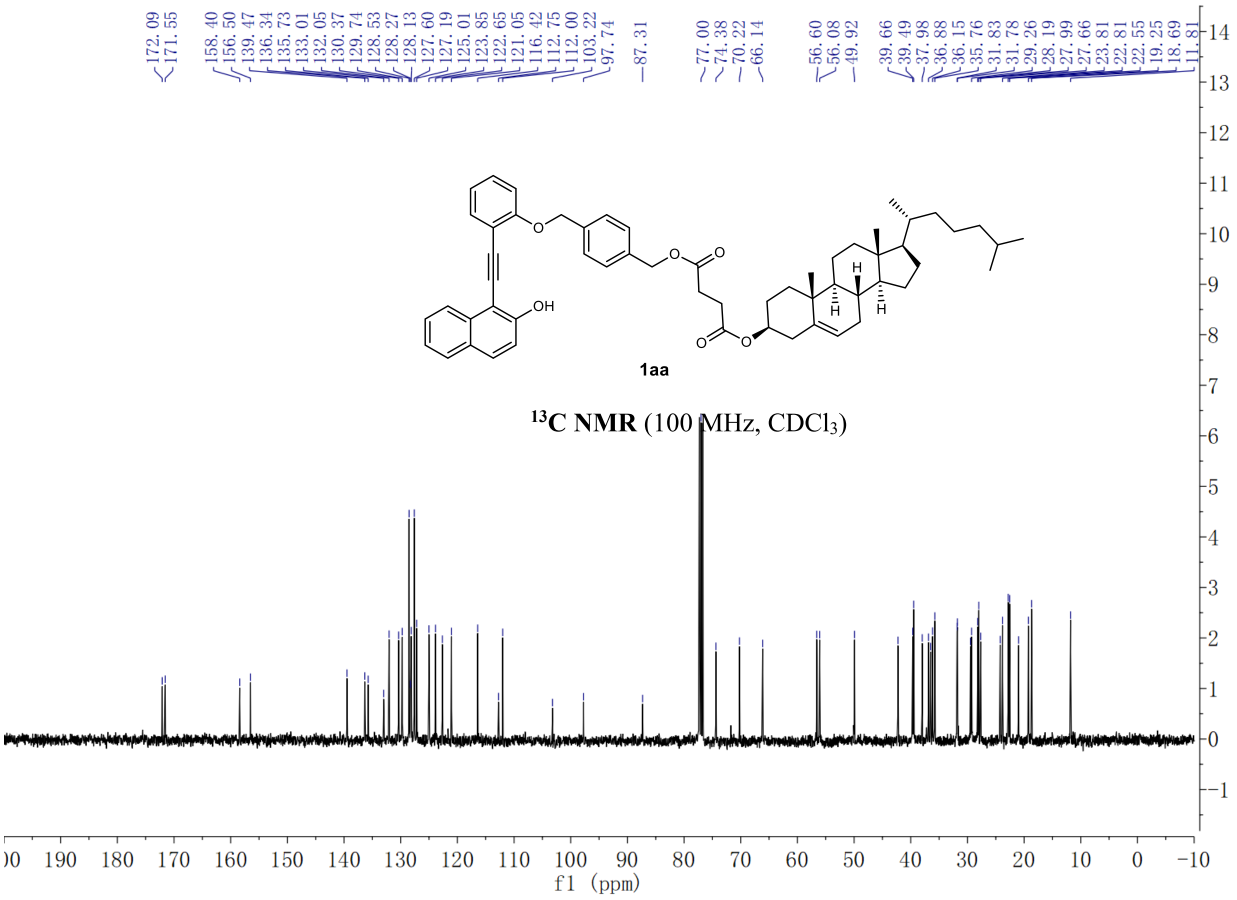
1aa
¹³C NMR (100 MHz, CDCl₃)
f1 (ppm)

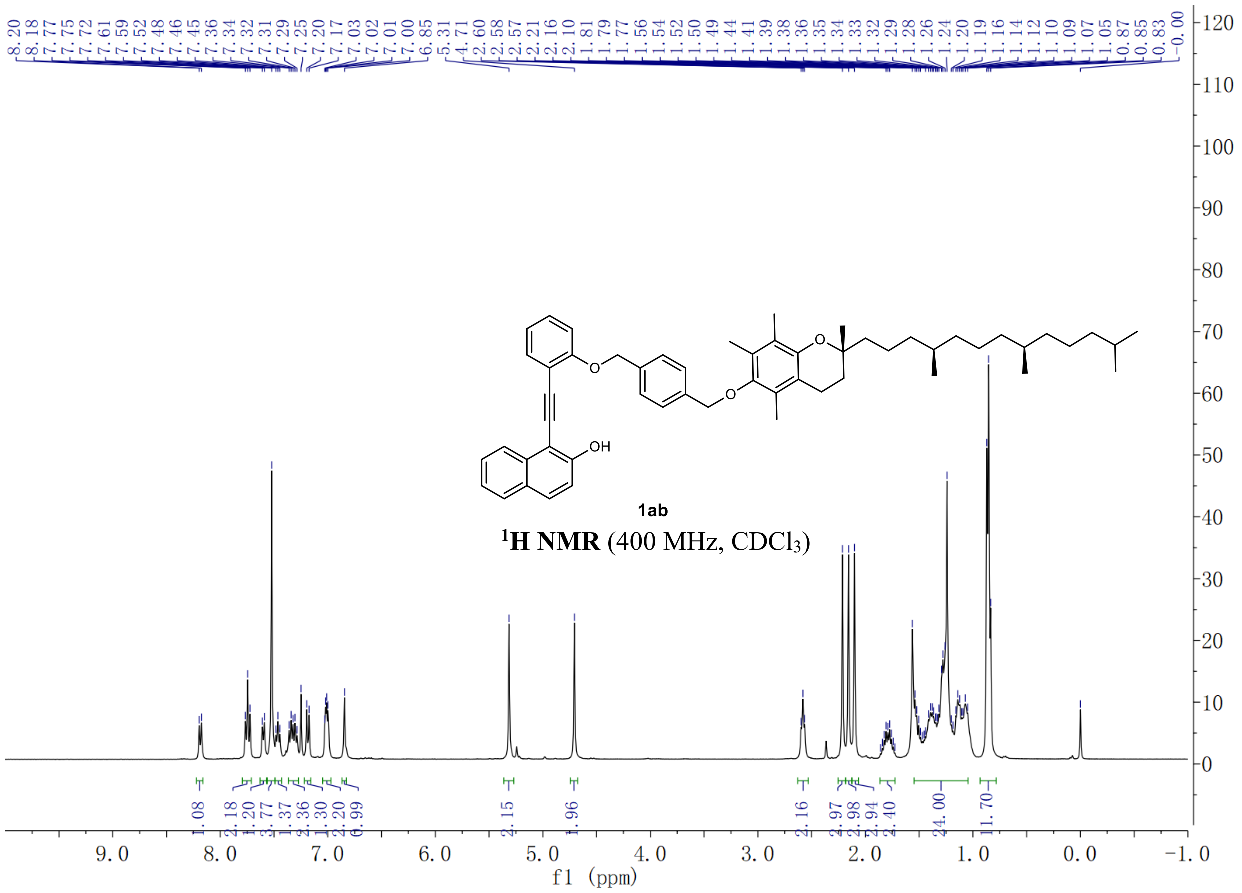

1ab

^{1}H NMR (400 MHz, $CDCl_3$)

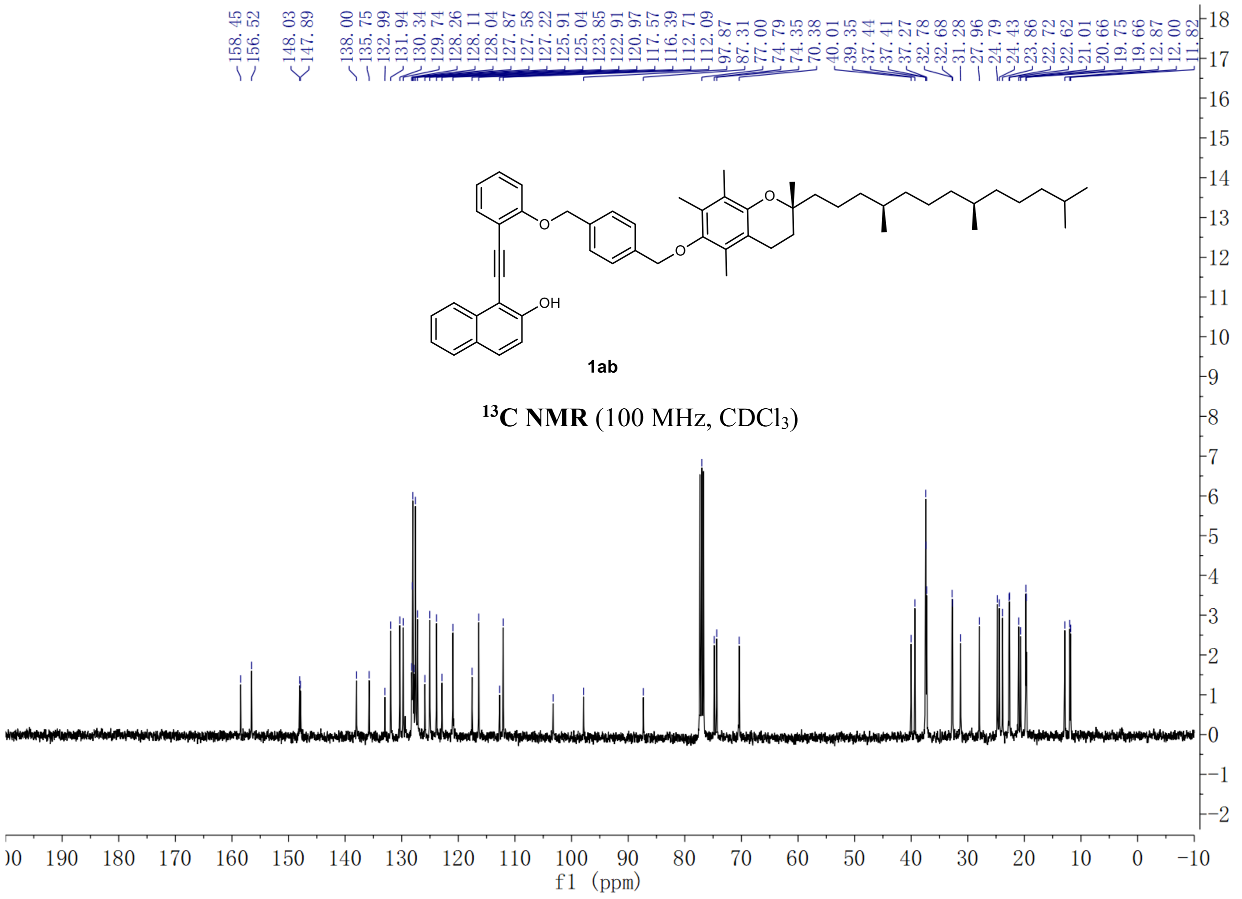

1ab

^{13}C NMR (100 MHz, $CDCl_3$)

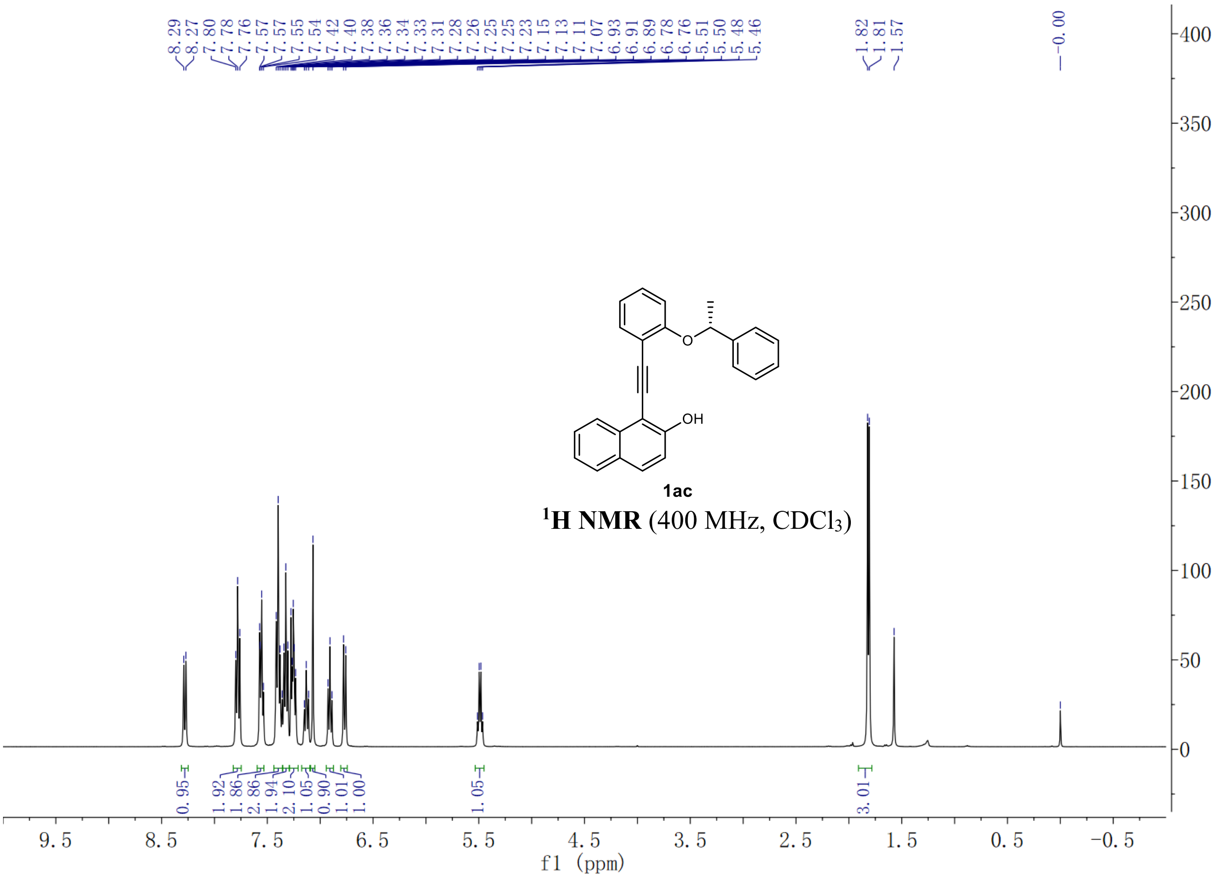

^{1}**H NMR** (400 MHz, $CDCl_3$)

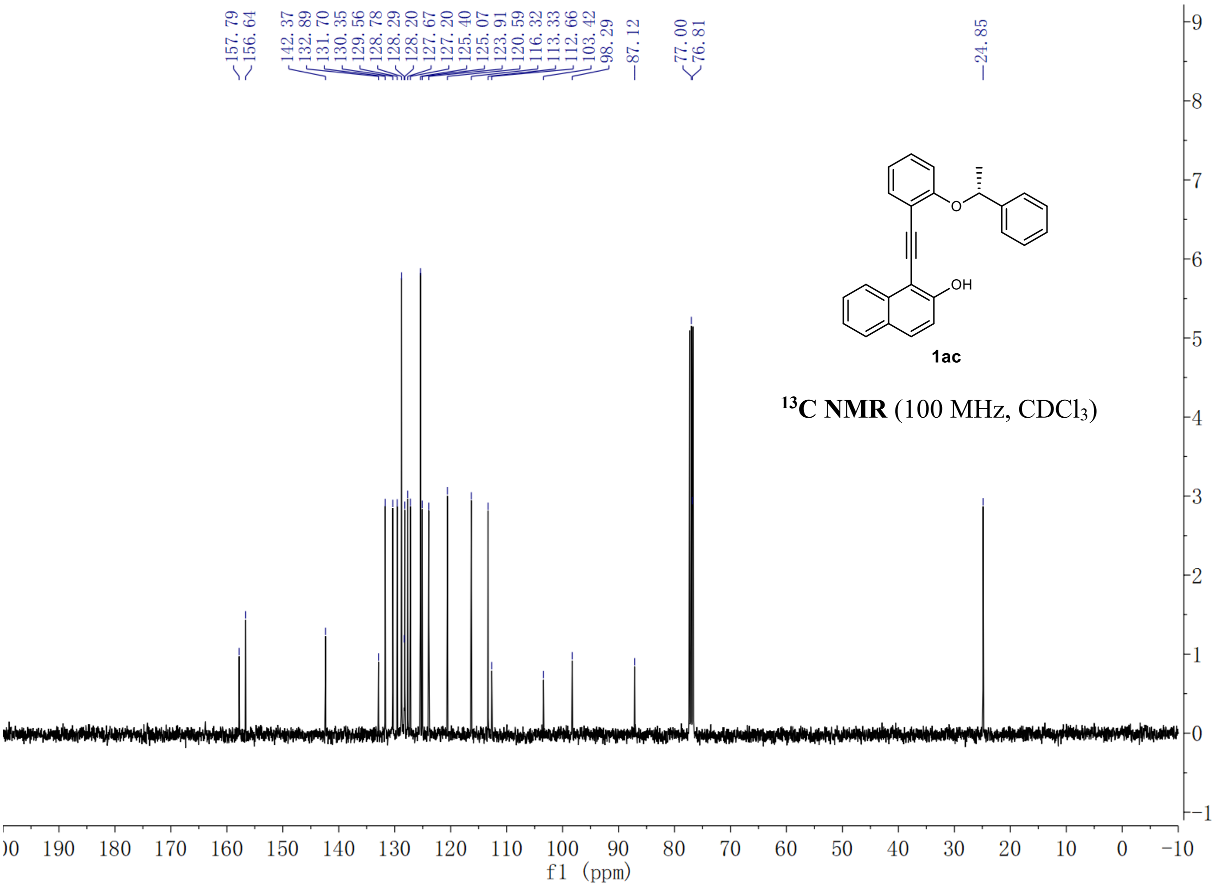

^{13}**C NMR** (100 MHz, $CDCl_3$)

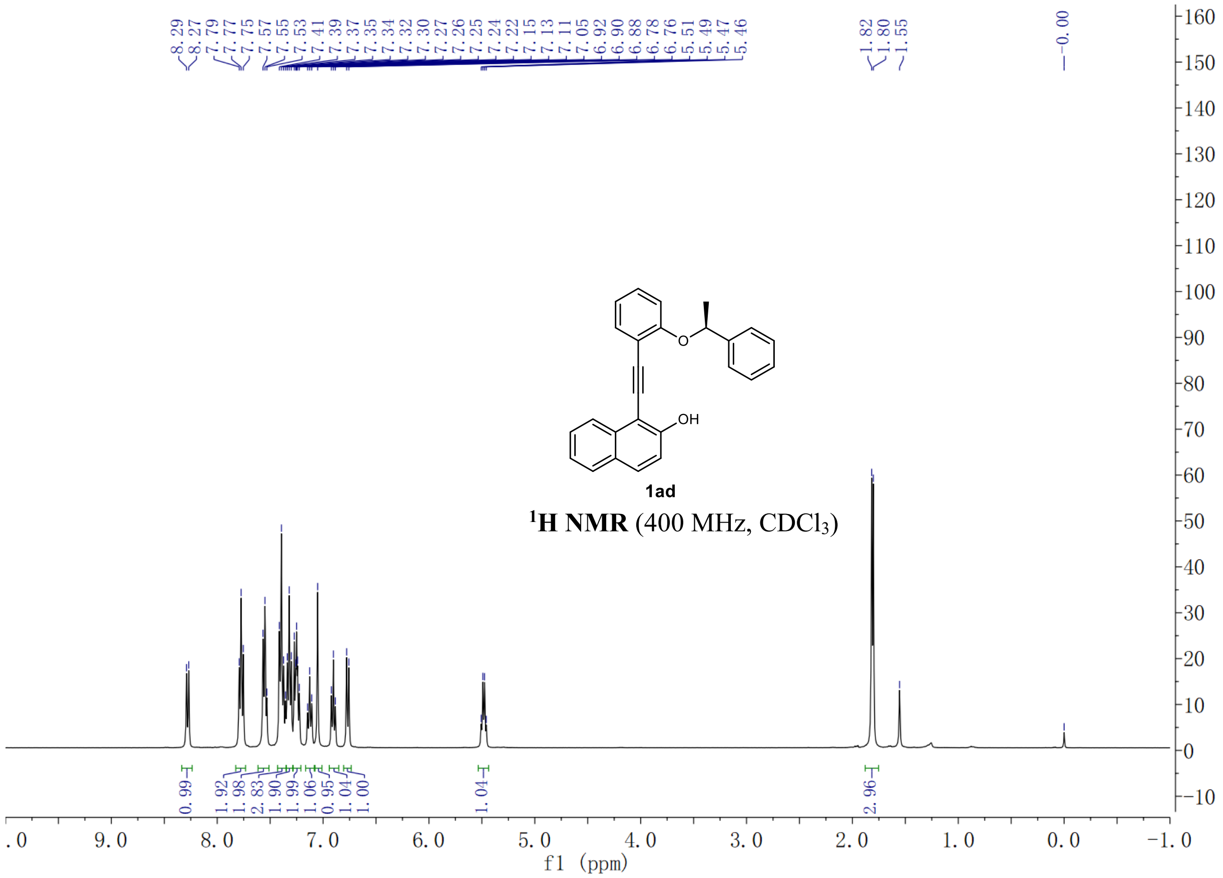
1ad
¹H NMR (400 MHz, CDCl₃)
f1 (ppm)

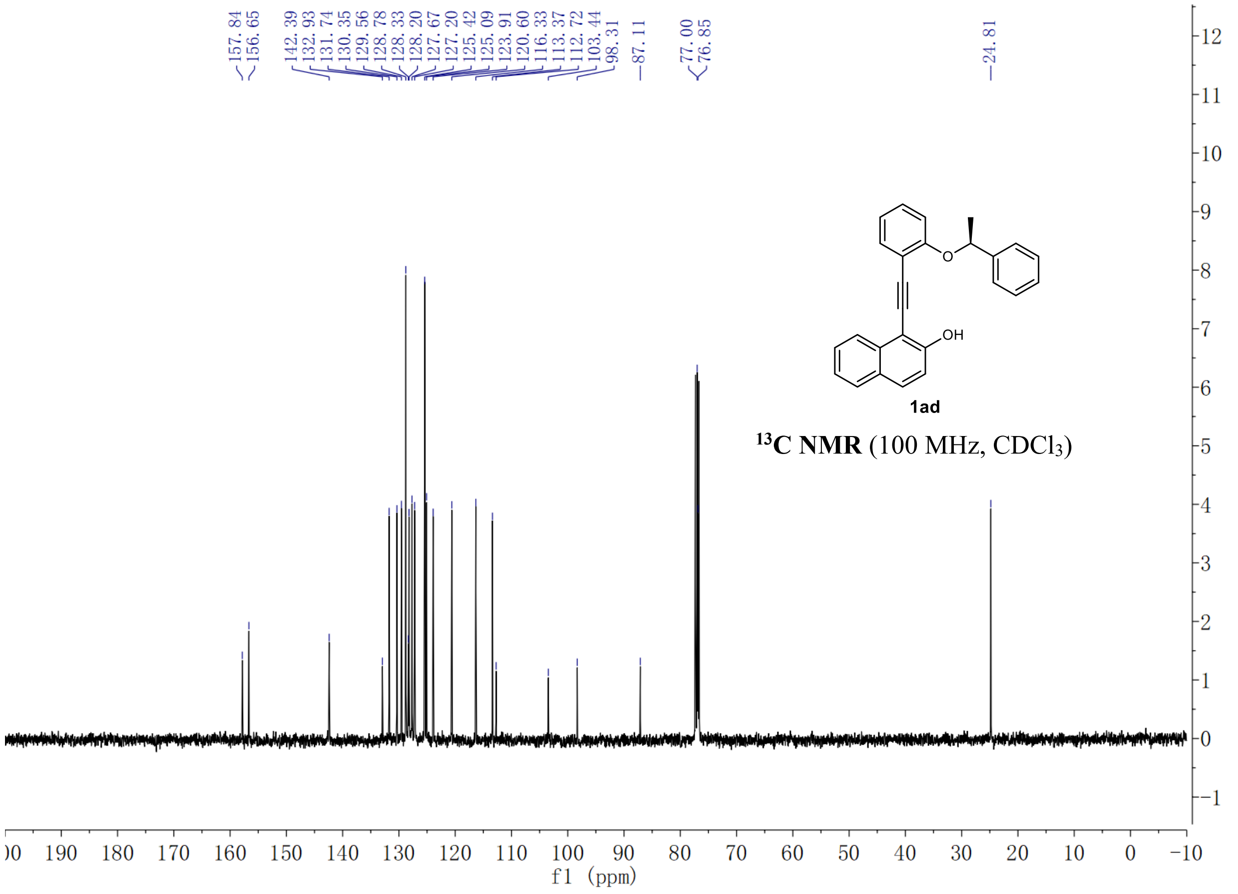
1ad
¹³C NMR (100 MHz, CDCl₃)
f1 (ppm)

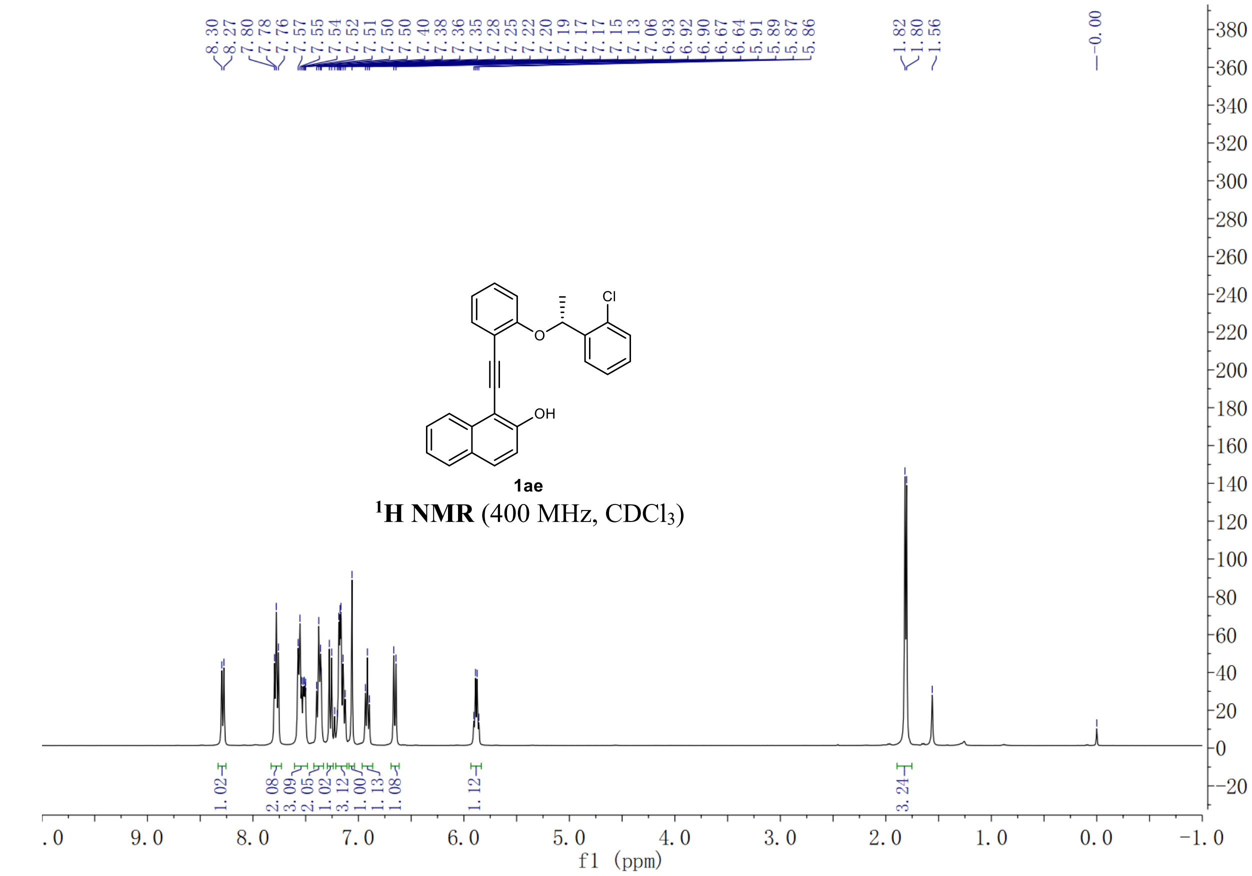

8.30
8.27
7.80
7.78
7.76
7.57
7.55
7.54
7.52
7.51
7.50
7.50
7.40
7.38
7.36
7.35
7.28
7.25
7.22
7.20
7.19
7.17
7.17
7.15
7.13
7.06
6.93
6.92
6.90
6.67
6.64
5.91
5.89
5.87
5.86
1.82
1.80
1.56
0.00
Cl
O
OH
1ae
¹H NMR (400 MHz, CDCl₃)
f1 (ppm)

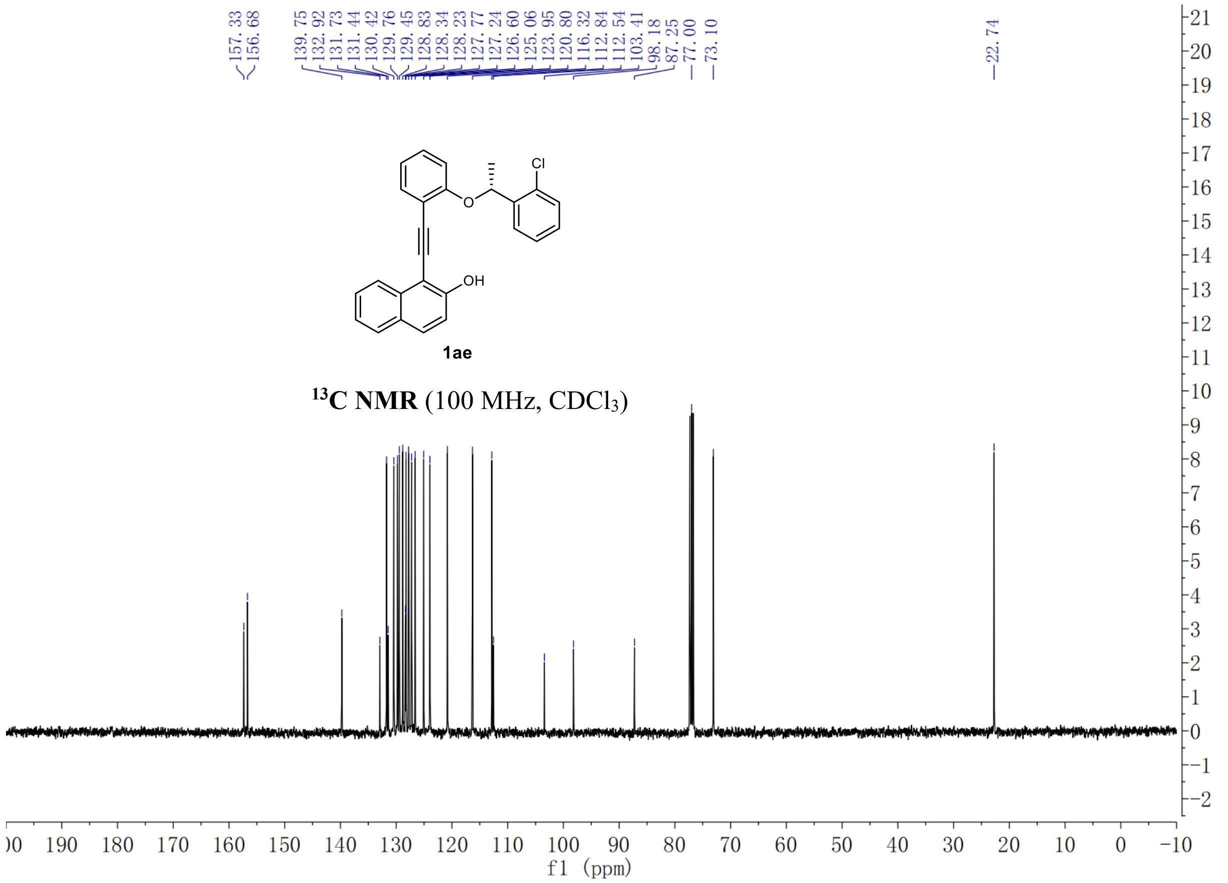

157.33
156.68
139.75
132.92
131.73
131.44
130.42
129.76
129.45
128.83
128.34
128.23
127.77
127.24
126.60
125.06
123.95
120.80
116.32
112.84
112.54
103.41
98.18
87.25
77.00
73.10
22.74
Cl
O
OH
1ae
¹³C NMR (100 MHz, CDCl₃)
f1 (ppm)

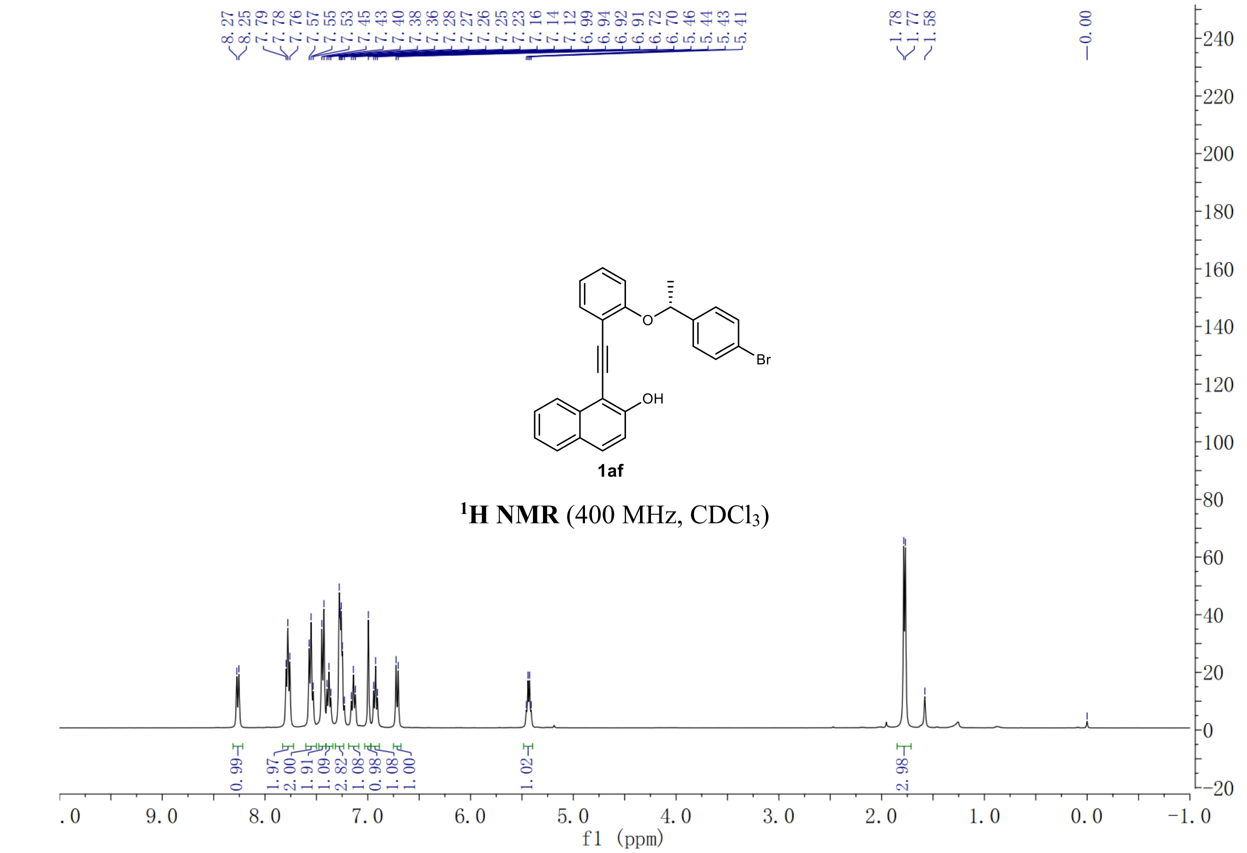
8.27
8.25
7.79
7.78
7.76
7.57
7.55
7.53
7.45
7.43
7.40
7.38
7.36
7.28
7.27
7.26
7.25
7.23
7.16
7.14
7.12
6.99
6.94
6.92
6.91
6.72
6.70
5.46
5.44
5.43
5.41
1.78
1.77
1.58
0.00
OH
O
Br
1af
¹H NMR (400 MHz, CDCl₃)
0.99
1.97
2.00
1.91
1.09
2.82
1.08
0.98
1.08
1.00
1.02
2.98
f1 (ppm)

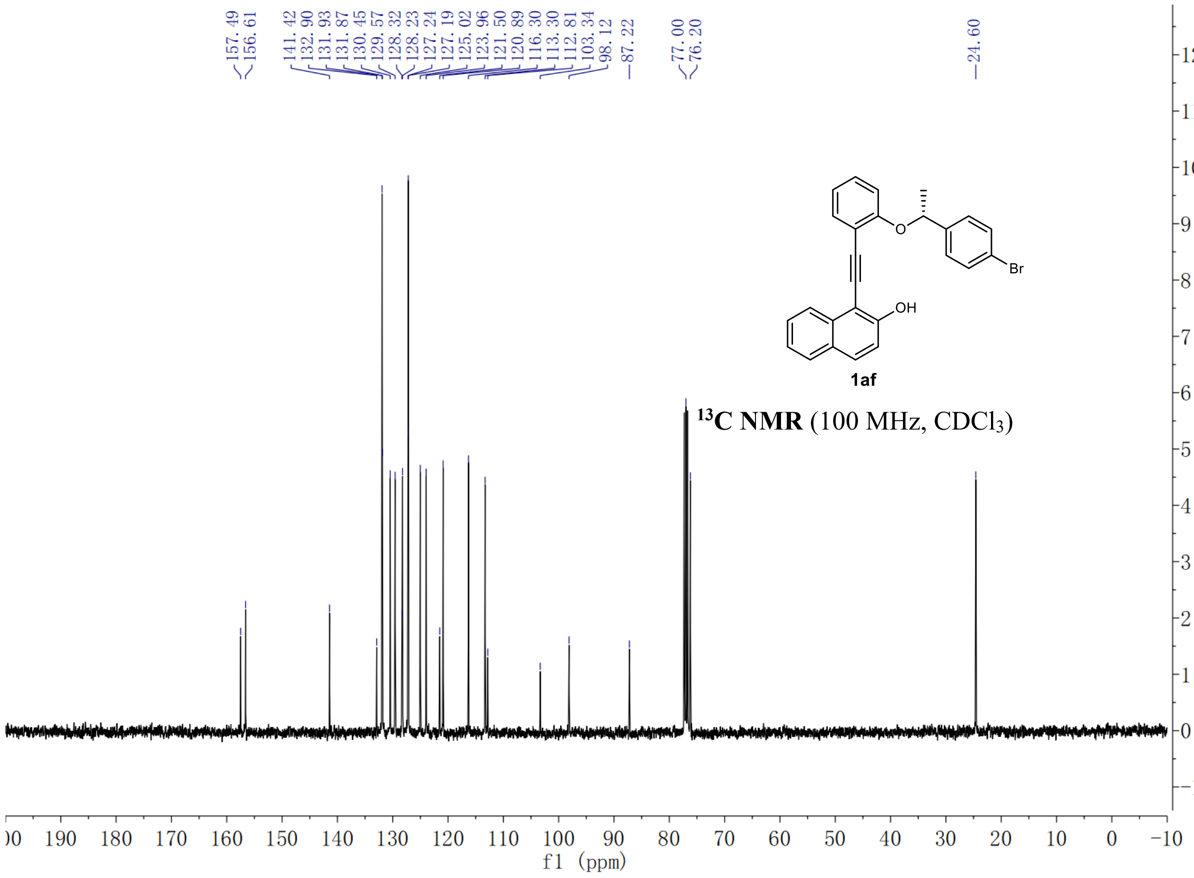
157.49
156.61
141.42
132.90
131.93
131.87
130.45
129.57
128.32
128.23
127.24
127.19
125.02
123.96
121.50
120.89
116.30
113.30
112.81
103.34
98.12
87.22
77.00
76.20
24.60
OH
O
Br
1af
¹³C NMR (100 MHz, CDCl₃)
f1 (ppm)

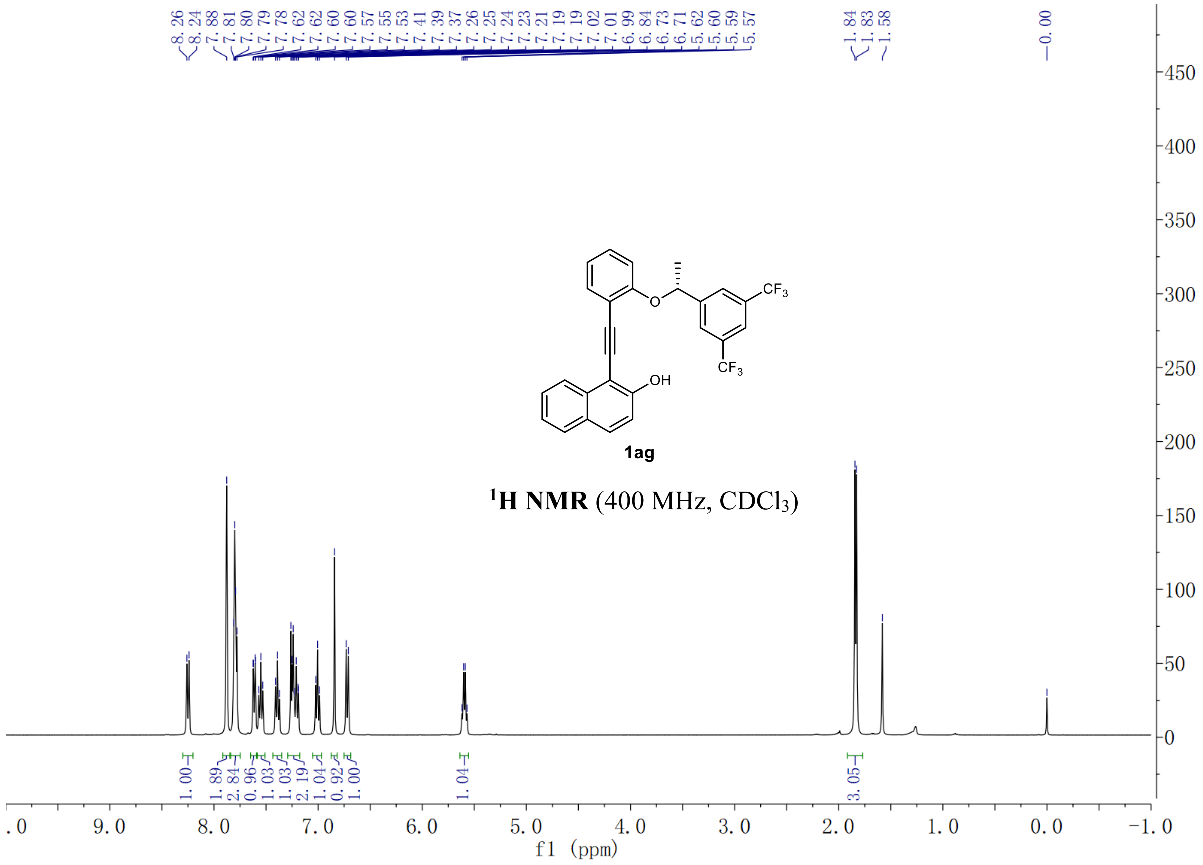

^{1}H NMR (400 MHz, $CDCl_3$)

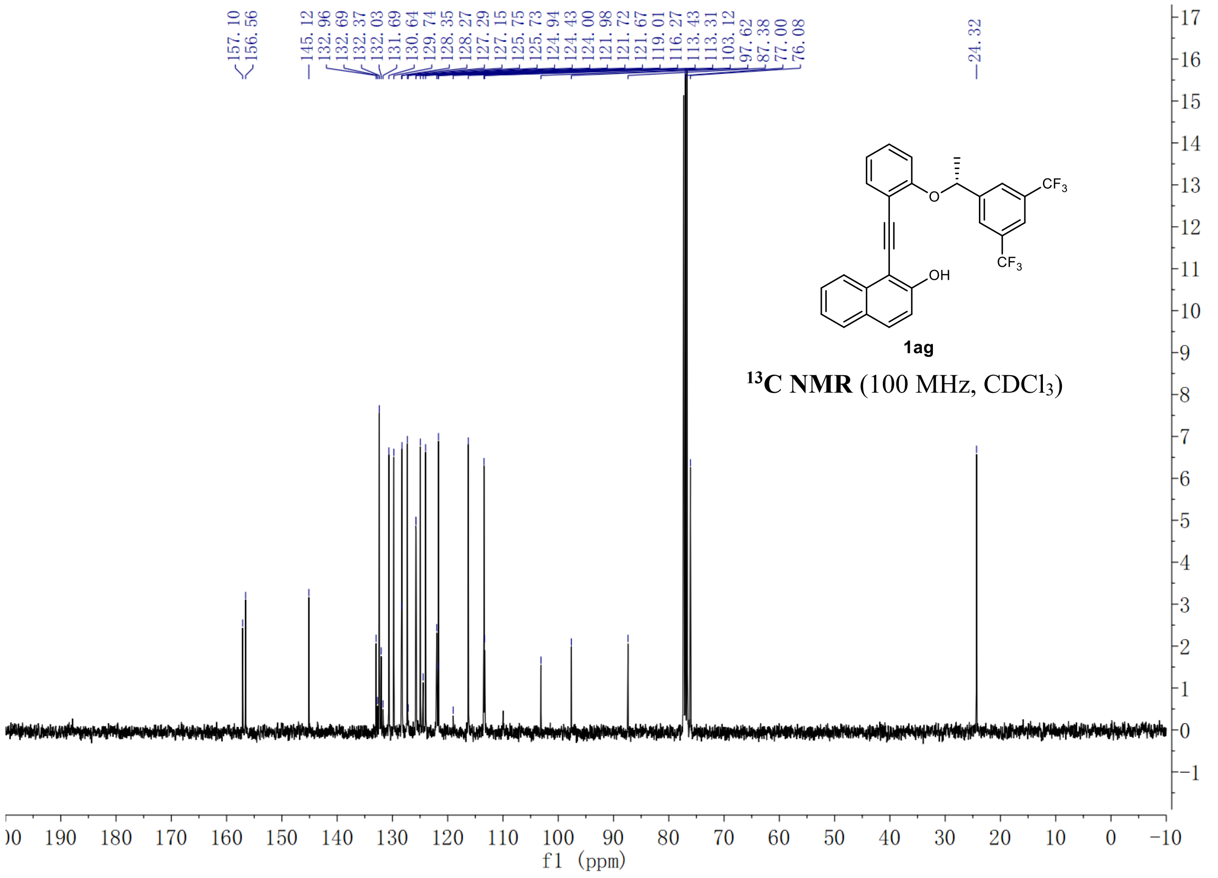

^{13}C NMR (100 MHz, $CDCl_3$)

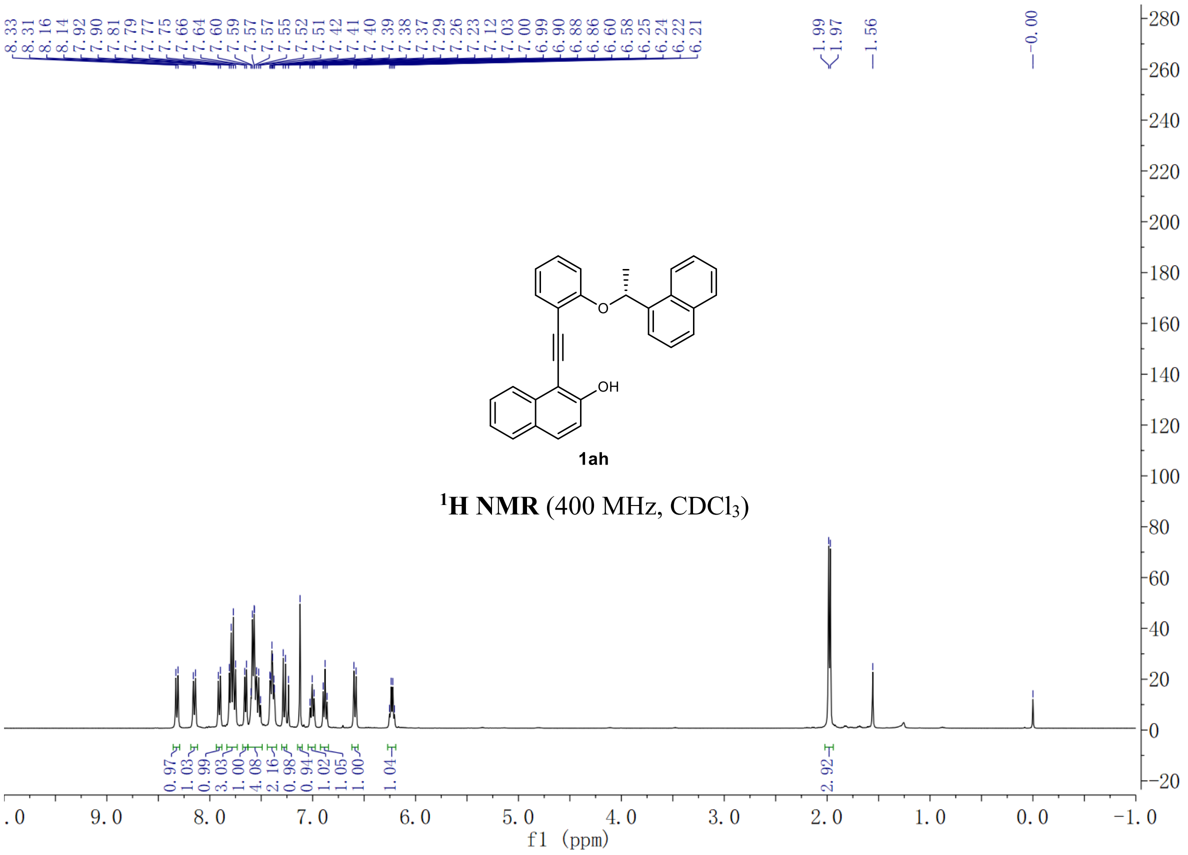
1ah
¹H NMR (400 MHz, CDCl₃)
f1 (ppm)

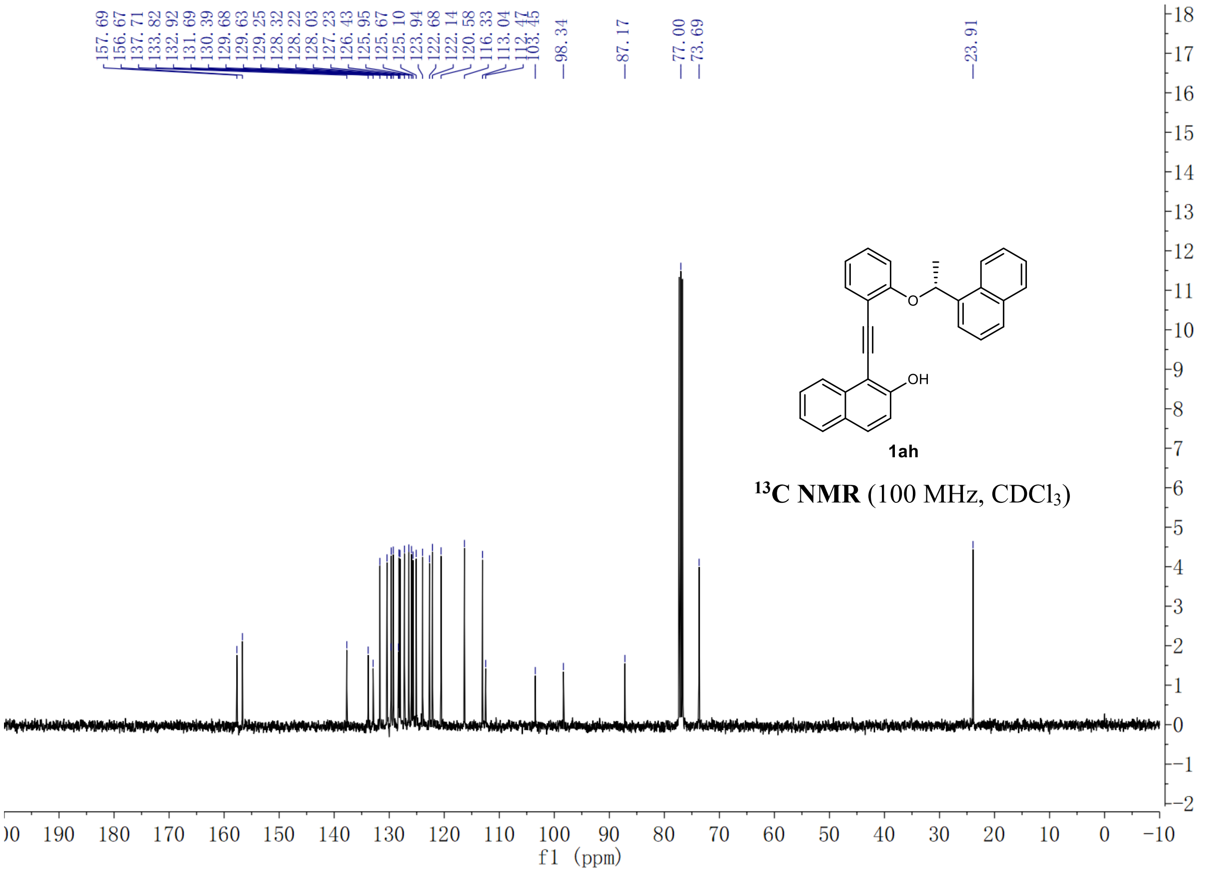
1ah
¹³C NMR (100 MHz, CDCl₃)
f1 (ppm)

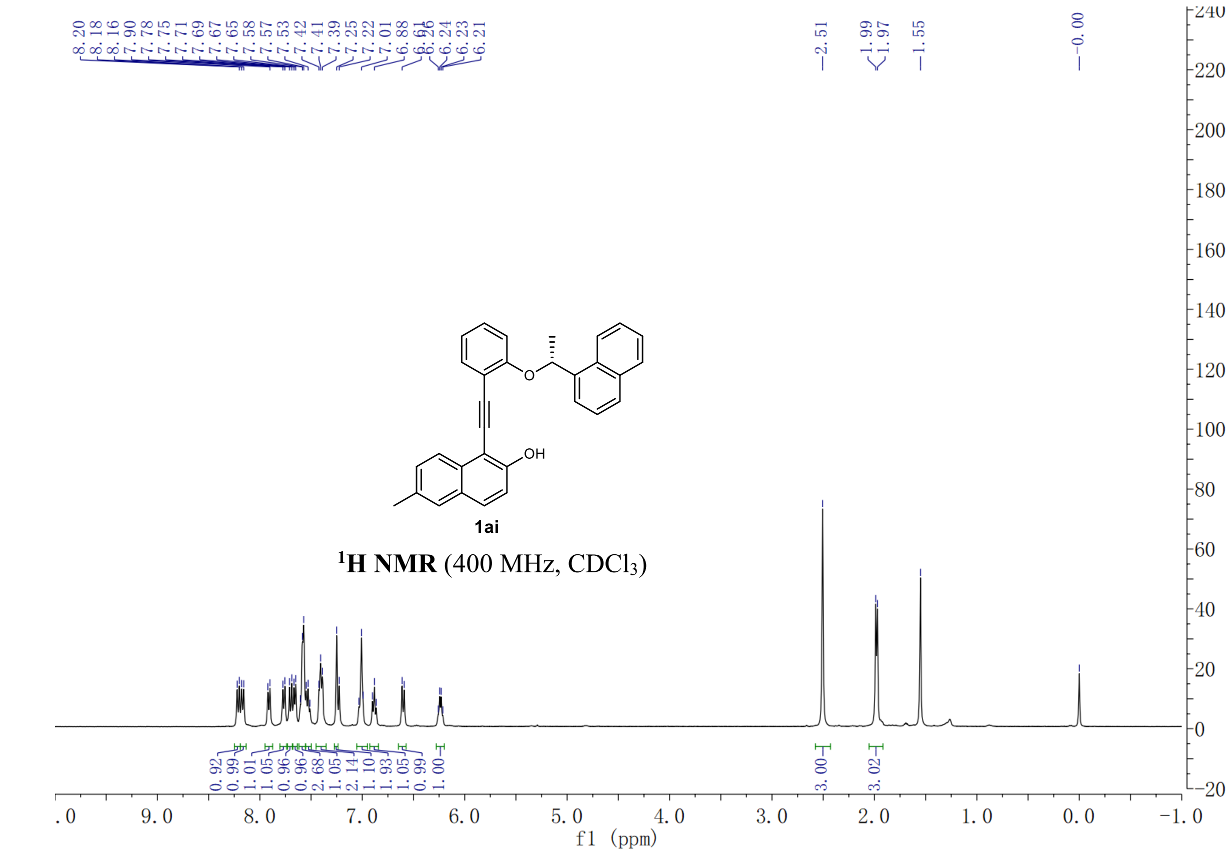
8.20
8.18
8.16
7.90
7.78
7.75
7.71
7.69
7.67
7.65
7.58
7.57
7.53
7.42
7.41
7.39
7.25
7.22
7.01
6.88
6.61
6.26
6.24
6.23
6.21
2.51
1.99
1.97
1.55
0.00
OH
O
1ai
^{1}H NMR (400 MHz, CDCl$_3$)
0.92
0.99
1.01
1.05
0.96
0.96
2.68
1.05
2.14
1.10
1.93
1.05
0.99
1.00
3.00
3.02
f1 (ppm)

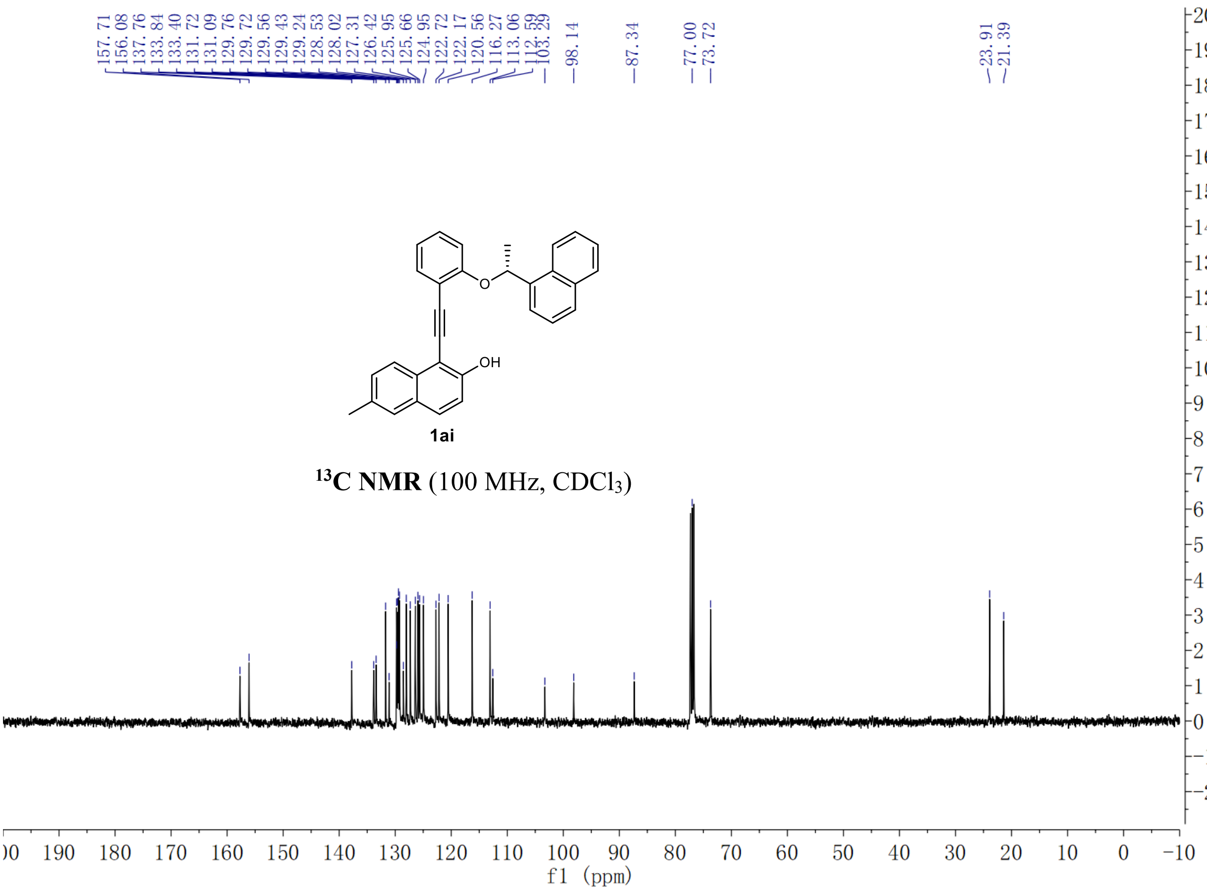
157.71
156.08
137.76
133.84
133.40
131.72
131.09
129.76
129.72
129.56
129.43
129.24
128.53
128.02
127.31
126.42
125.95
125.66
124.95
122.72
122.17
120.56
116.27
113.06
112.59
103.29
98.14
87.34
77.00
73.72
23.91
21.39
OH
O
1ai
^{13}C NMR (100 MHz, CDCl$_3$)
f1 (ppm)

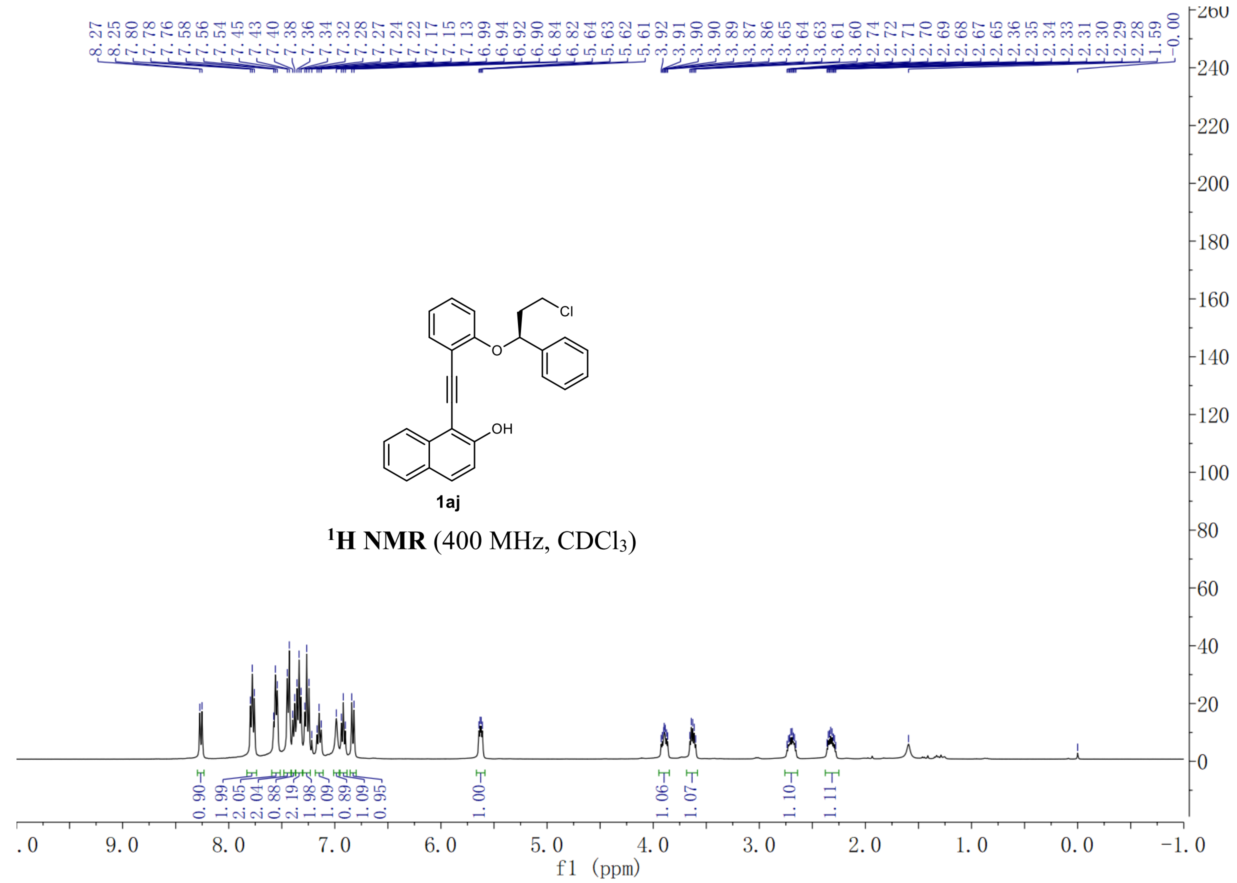

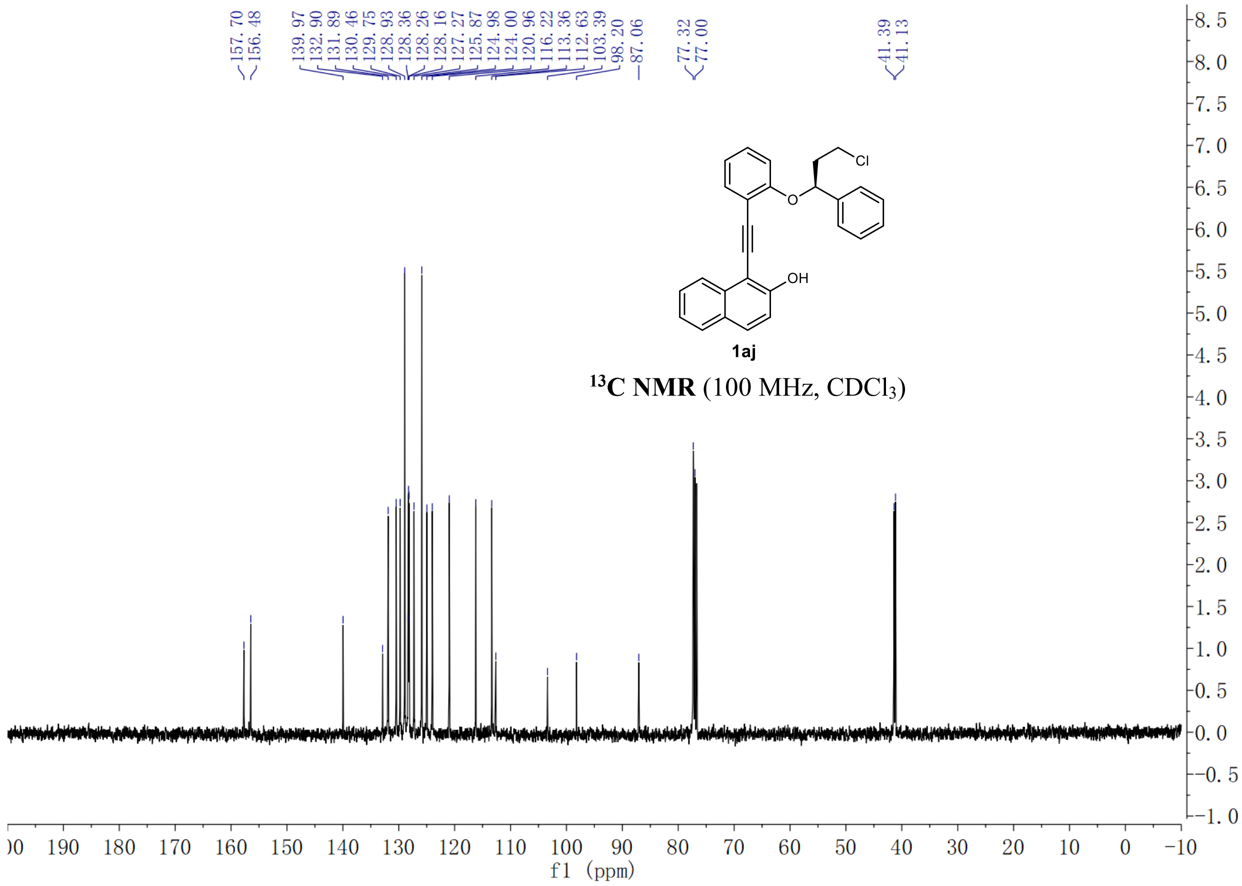

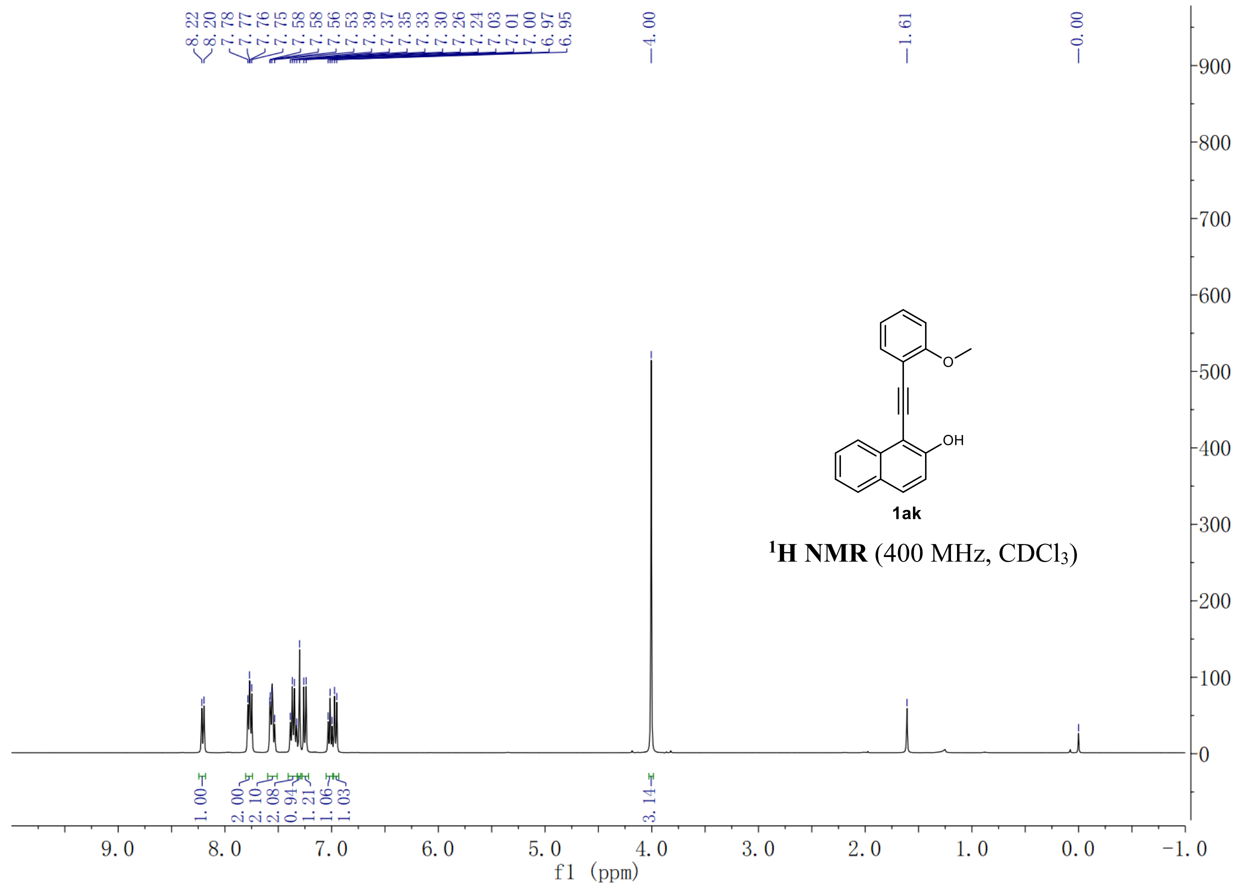

1ak
¹H NMR (400 MHz, CDCl₃)
f1 (ppm)

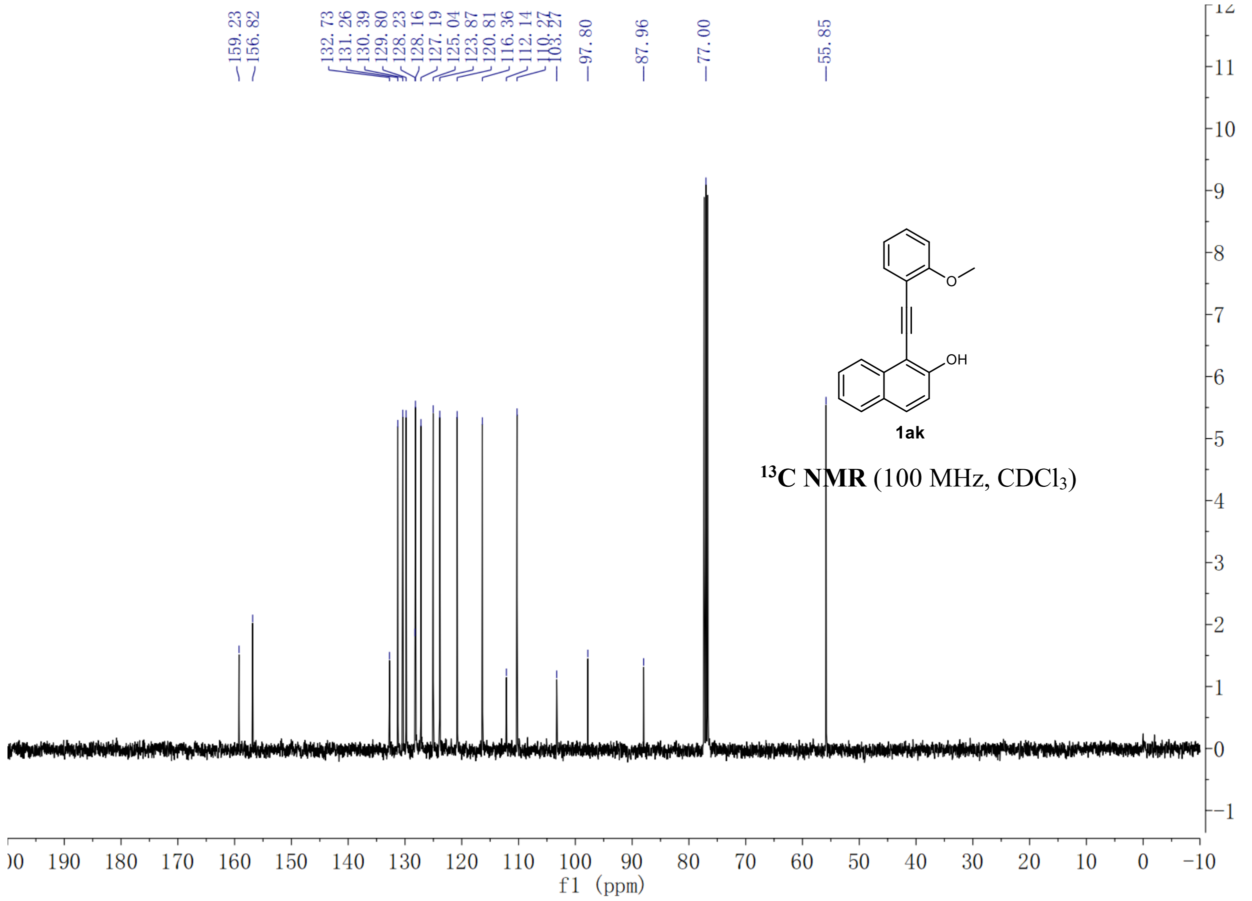

1ak
¹³C NMR (100 MHz, CDCl₃)
f1 (ppm)

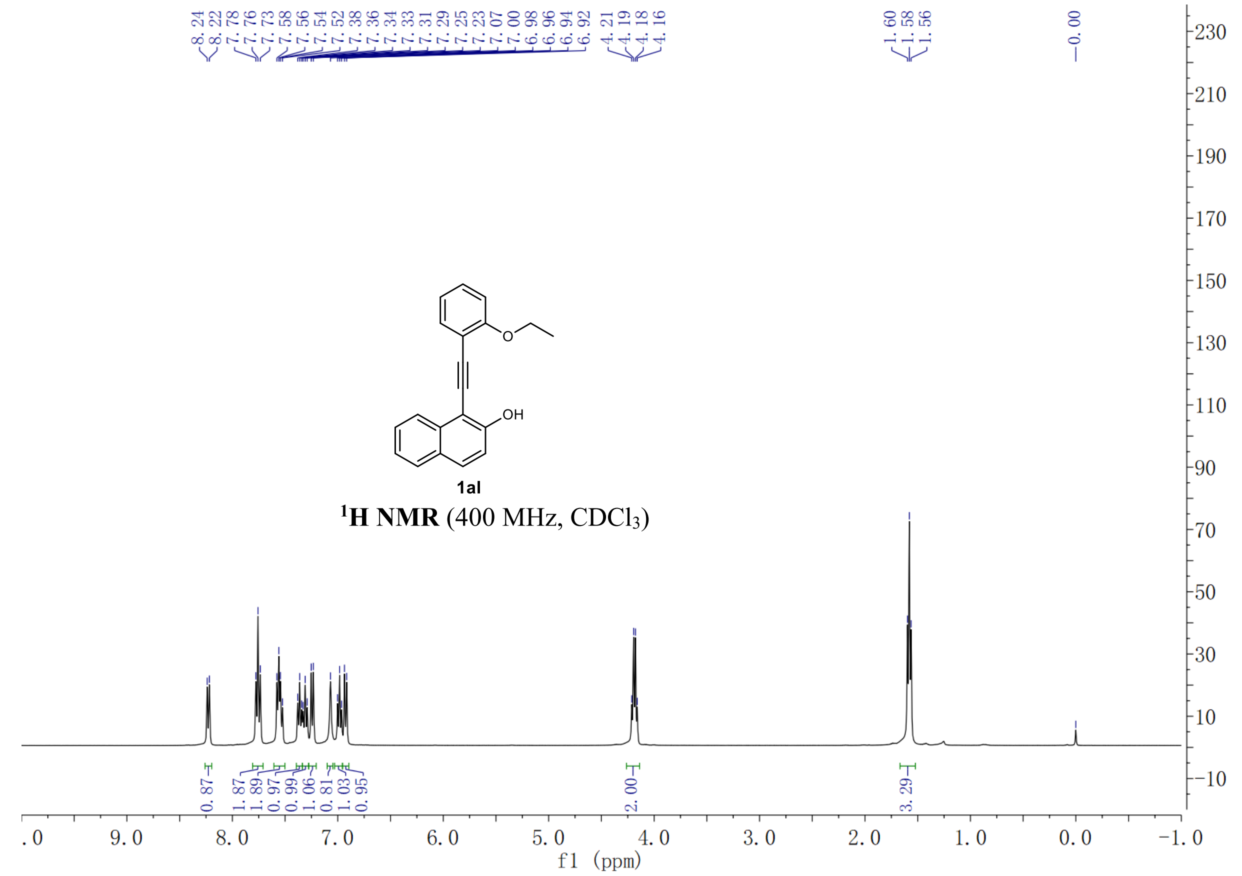

1al
^{1}H NMR (400 MHz, CDCl$_3$)

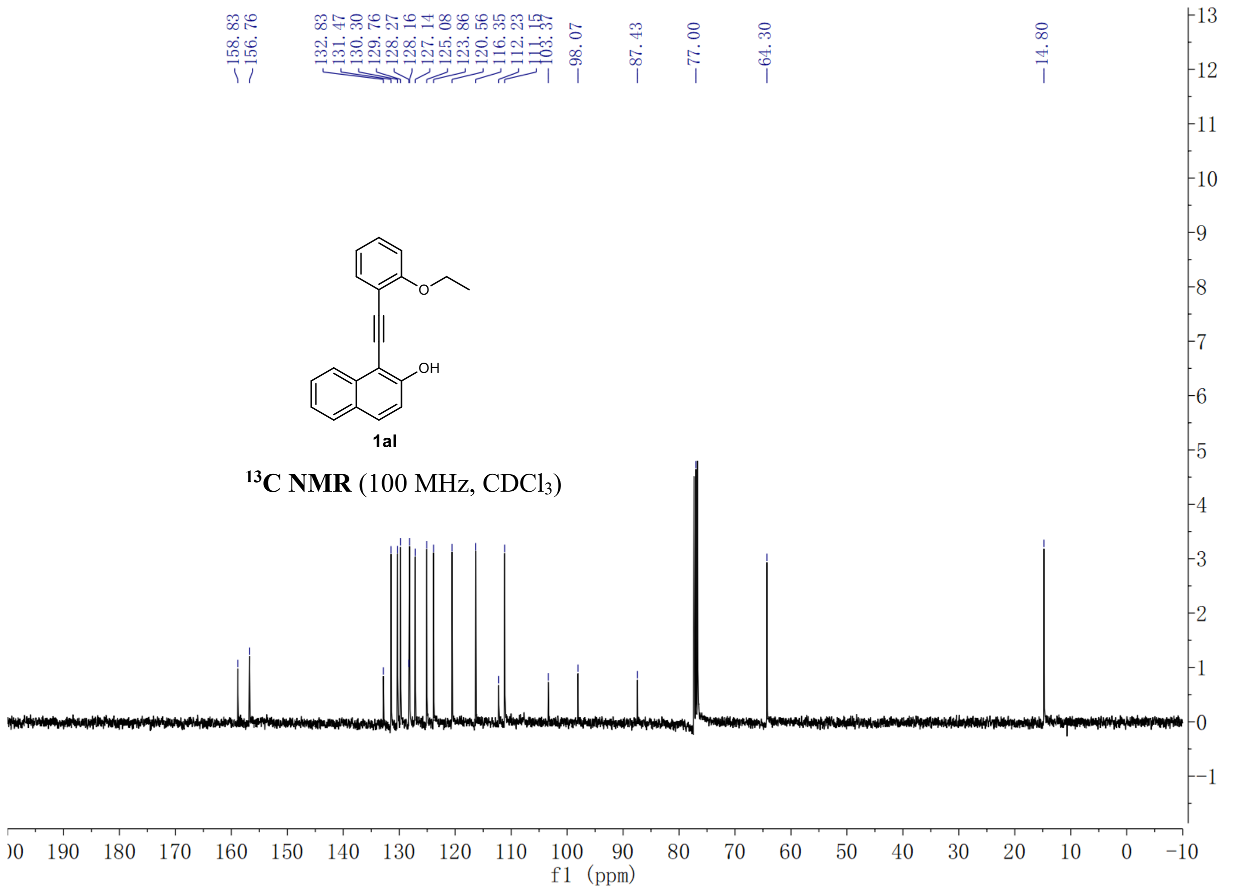

1al
^{13}C NMR (100 MHz, CDCl$_3$)

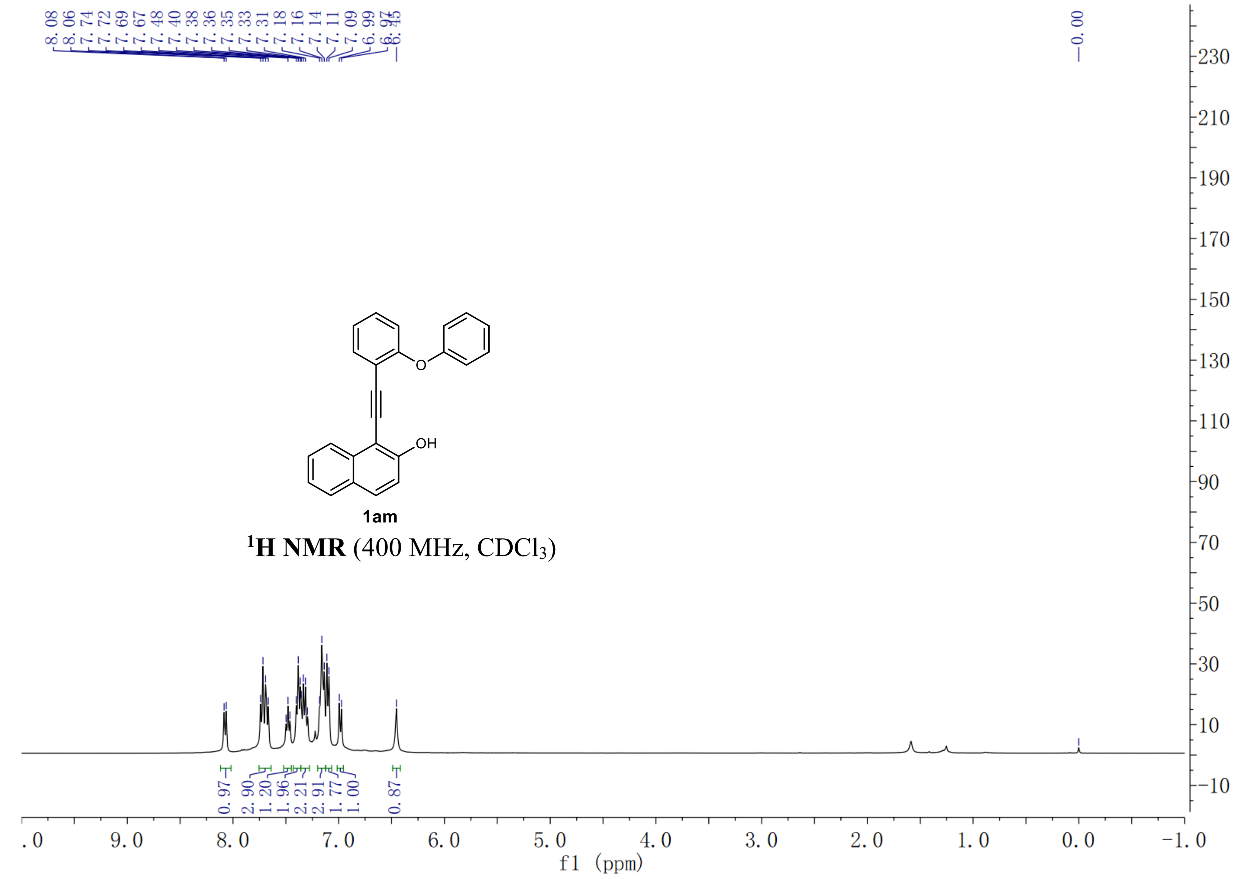

8.08 8.06 7.74 7.72 7.69 7.67 7.48 7.40 7.38 7.36 7.35 7.33 7.31 7.18 7.16 7.14 7.11 7.09 6.99 6.97 6.45
0.00
O
OH
1am
^{1}H NMR (400 MHz, CDCl$_3$)
0.97 2.90 1.20 1.96 2.21 2.91 1.77 1.00 0.87
f1 (ppm)

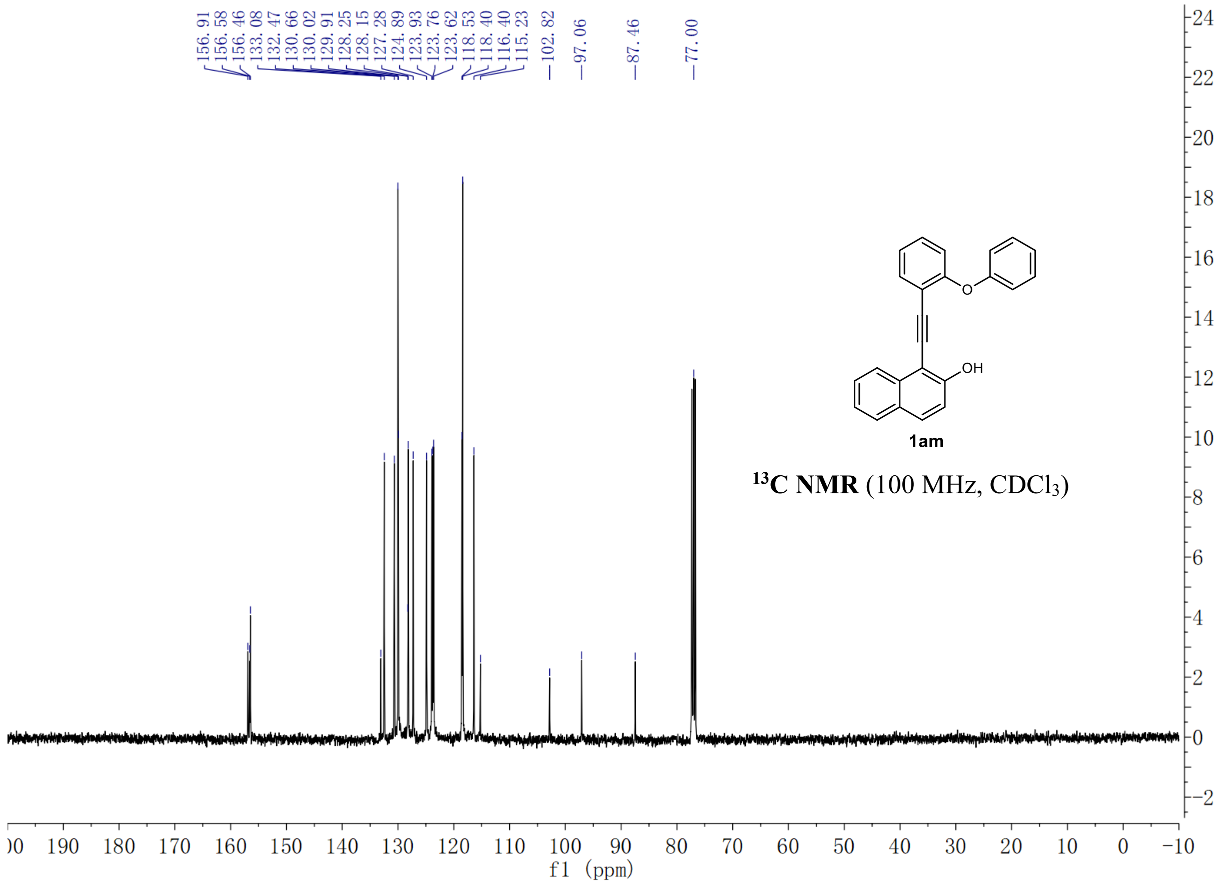

156.91 156.58 156.46 133.08 132.47 130.66 130.02 129.91 128.25 128.15 127.28 124.89 123.93 123.76 123.62 118.53 118.40 116.40 115.23
102.82
97.06
87.46
77.00
O
OH
1am
^{13}C NMR (100 MHz, CDCl$_3$)
f1 (ppm)

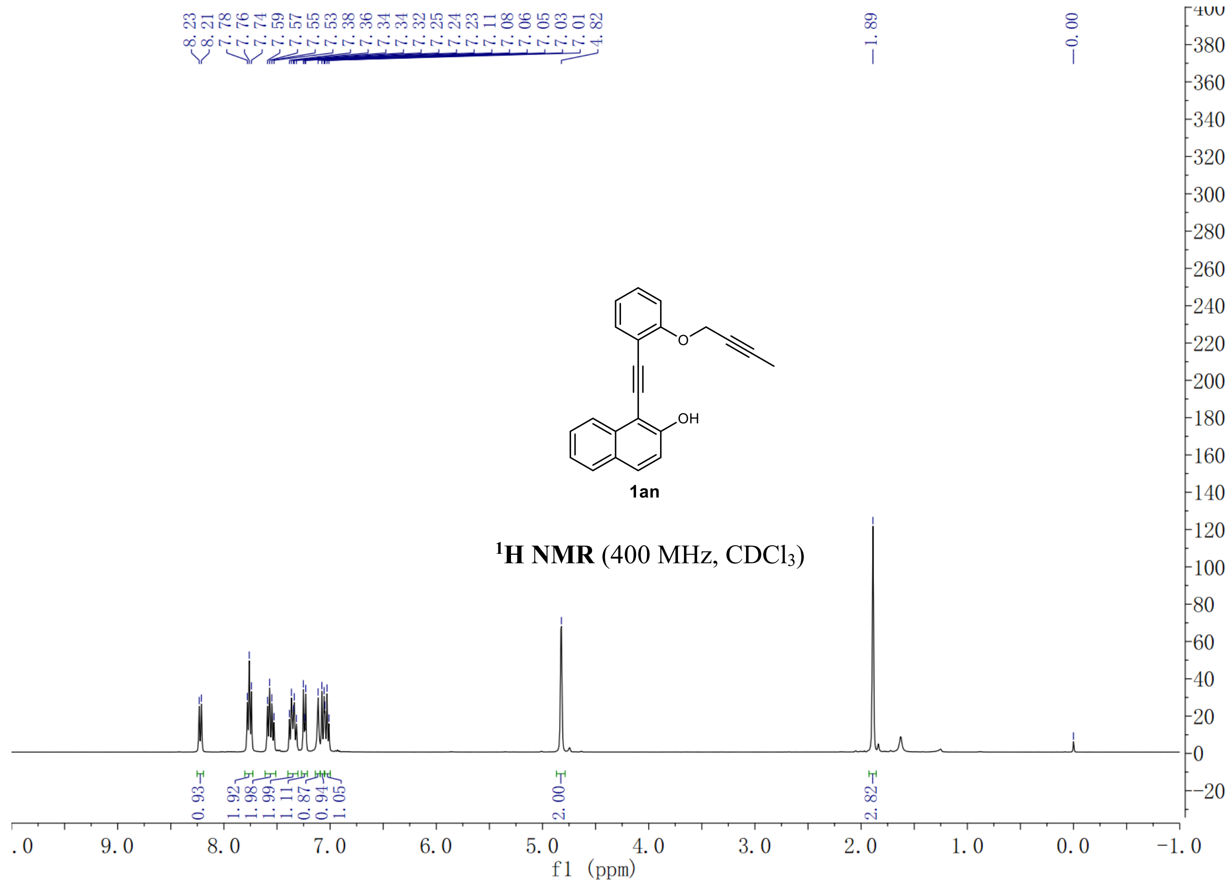

1an
¹H NMR (400 MHz, CDCl3)

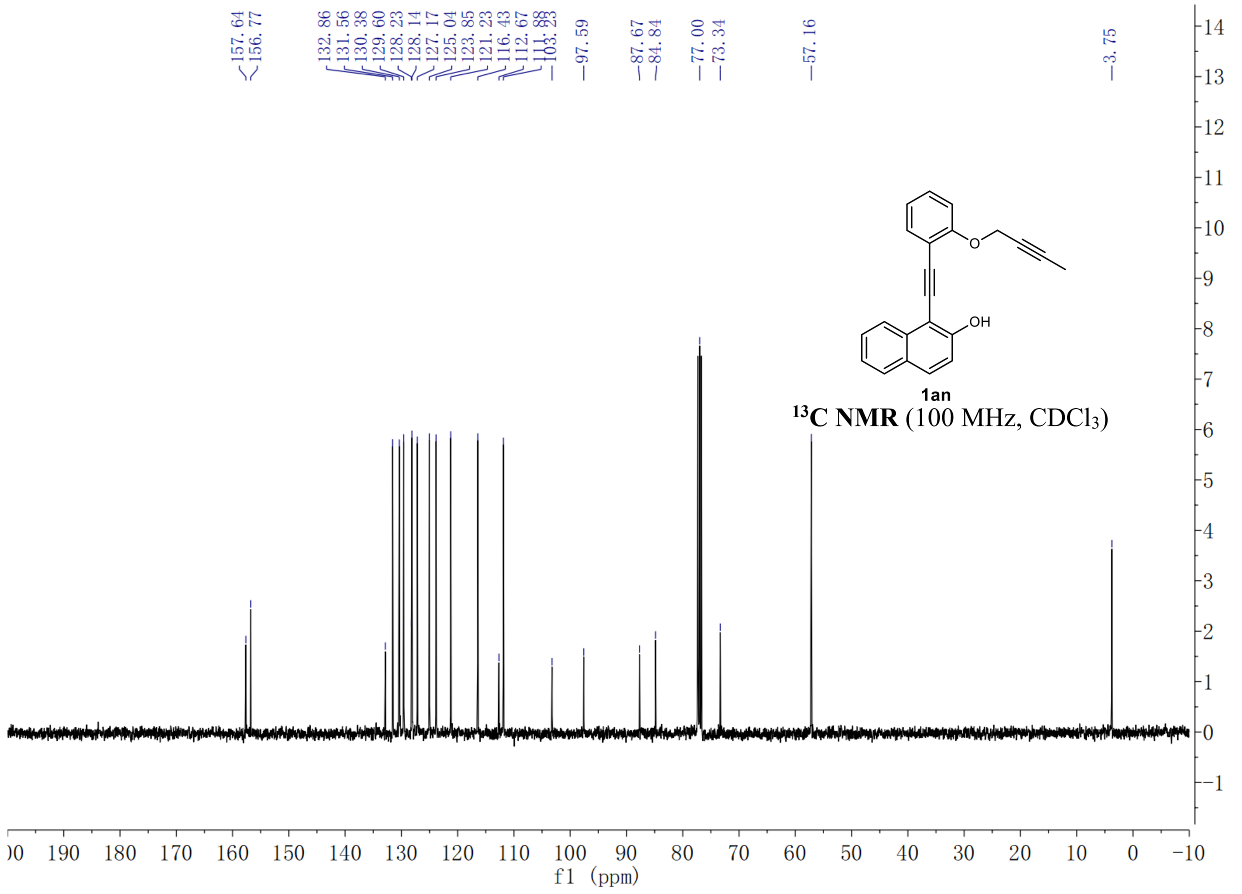

1an
¹³C NMR (100 MHz, CDCl3)

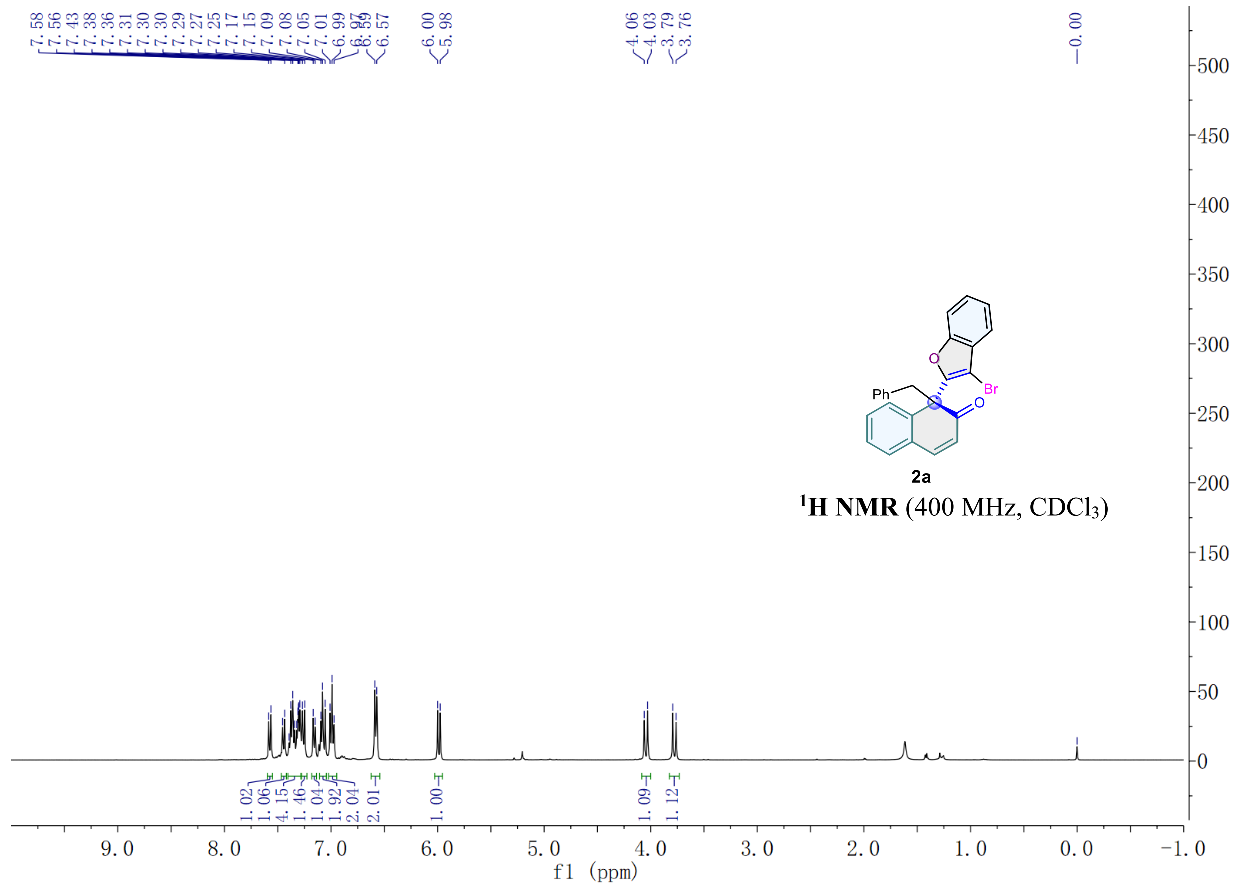

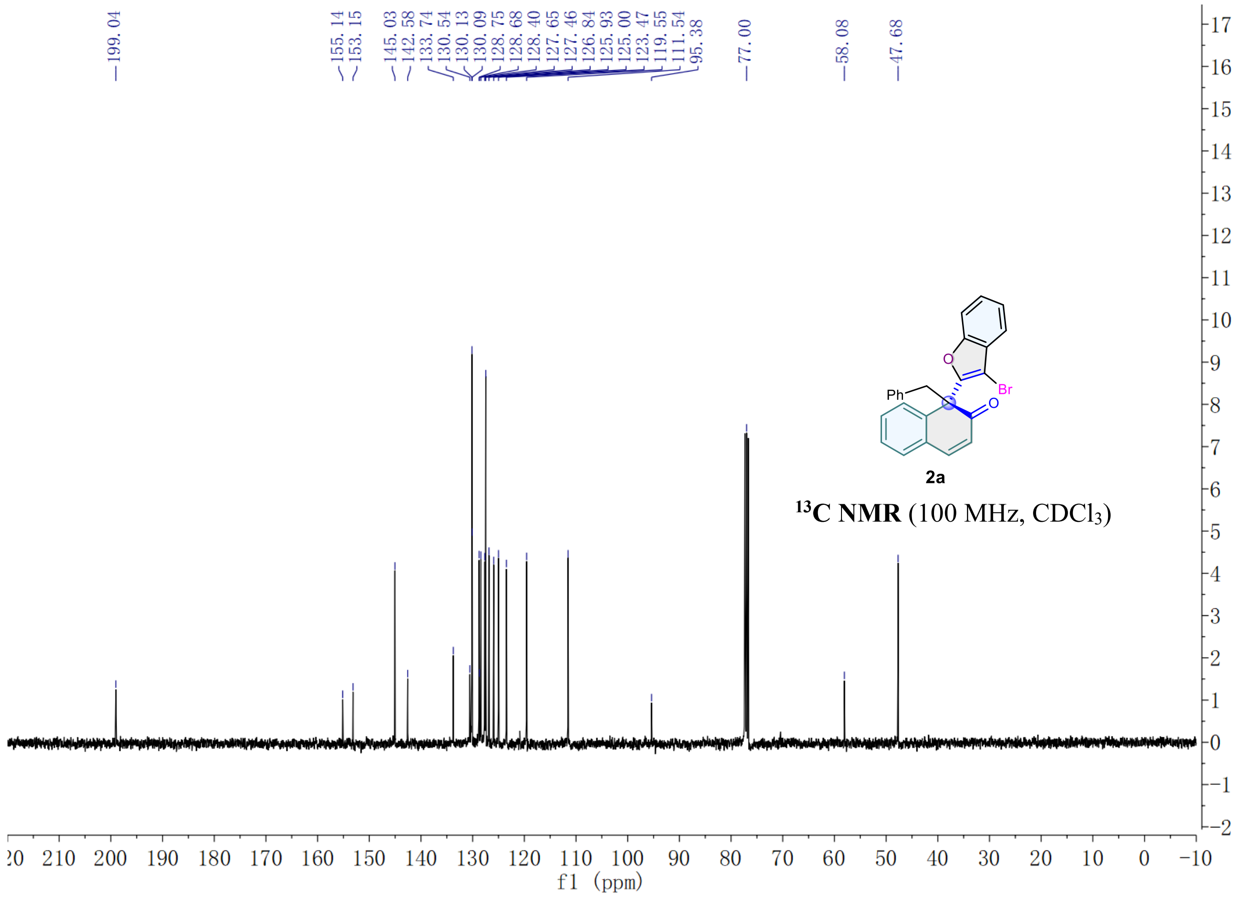

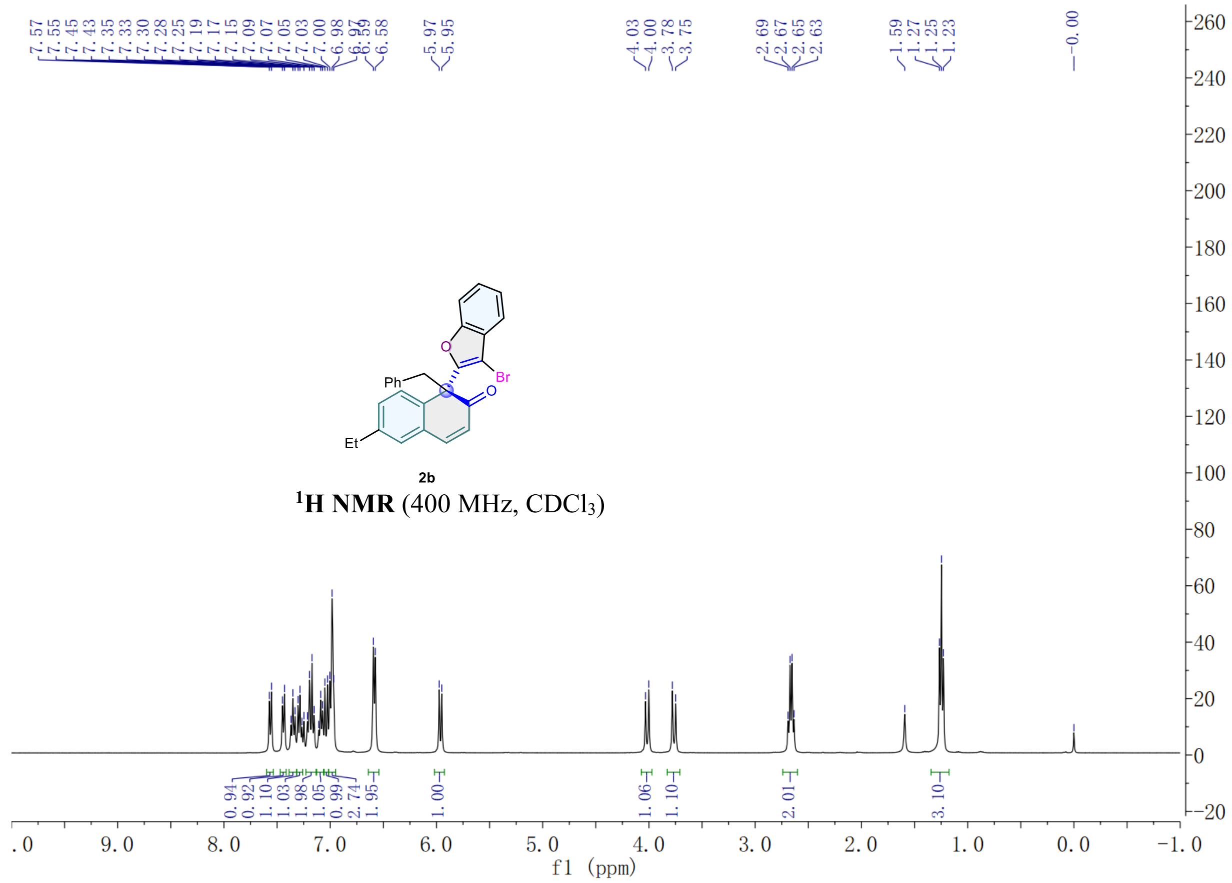

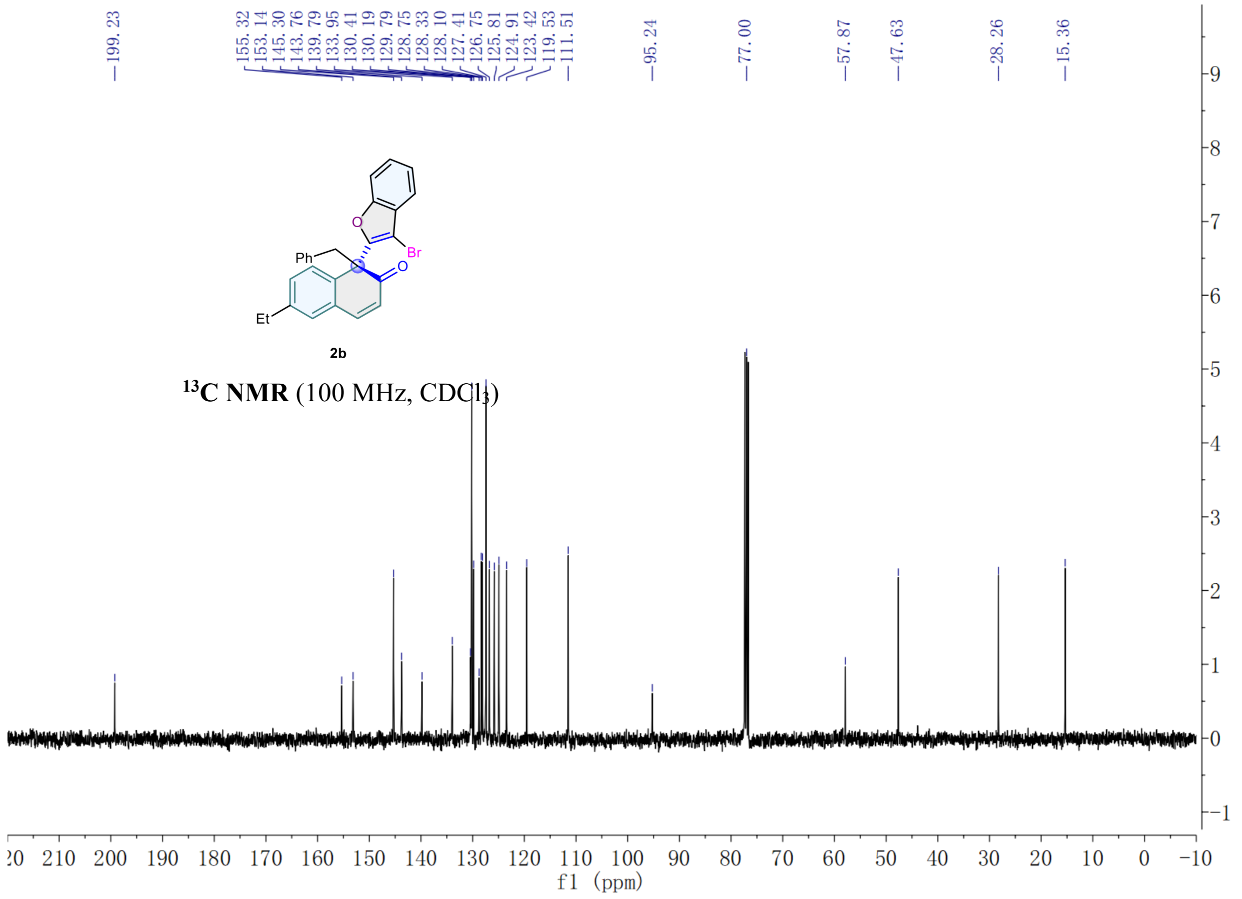

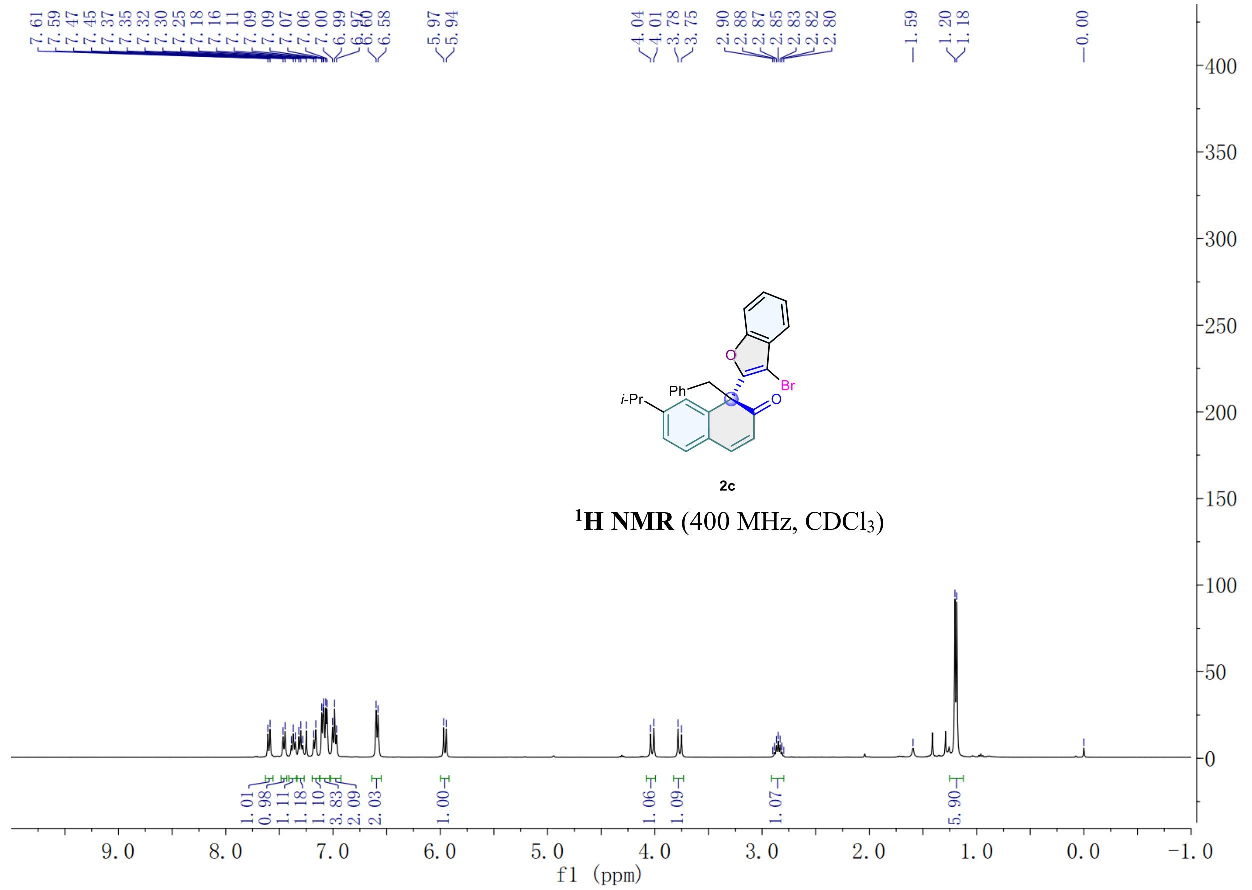

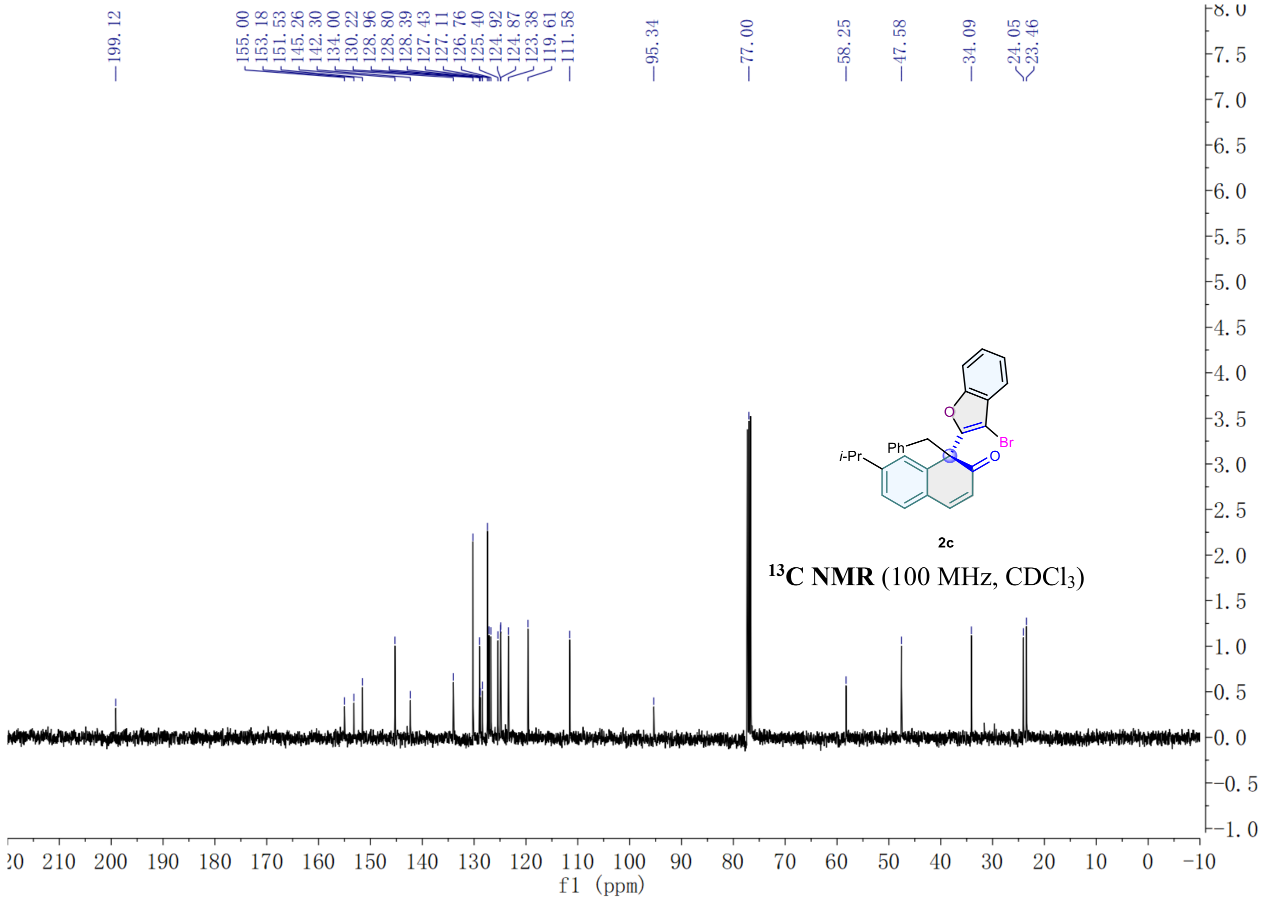

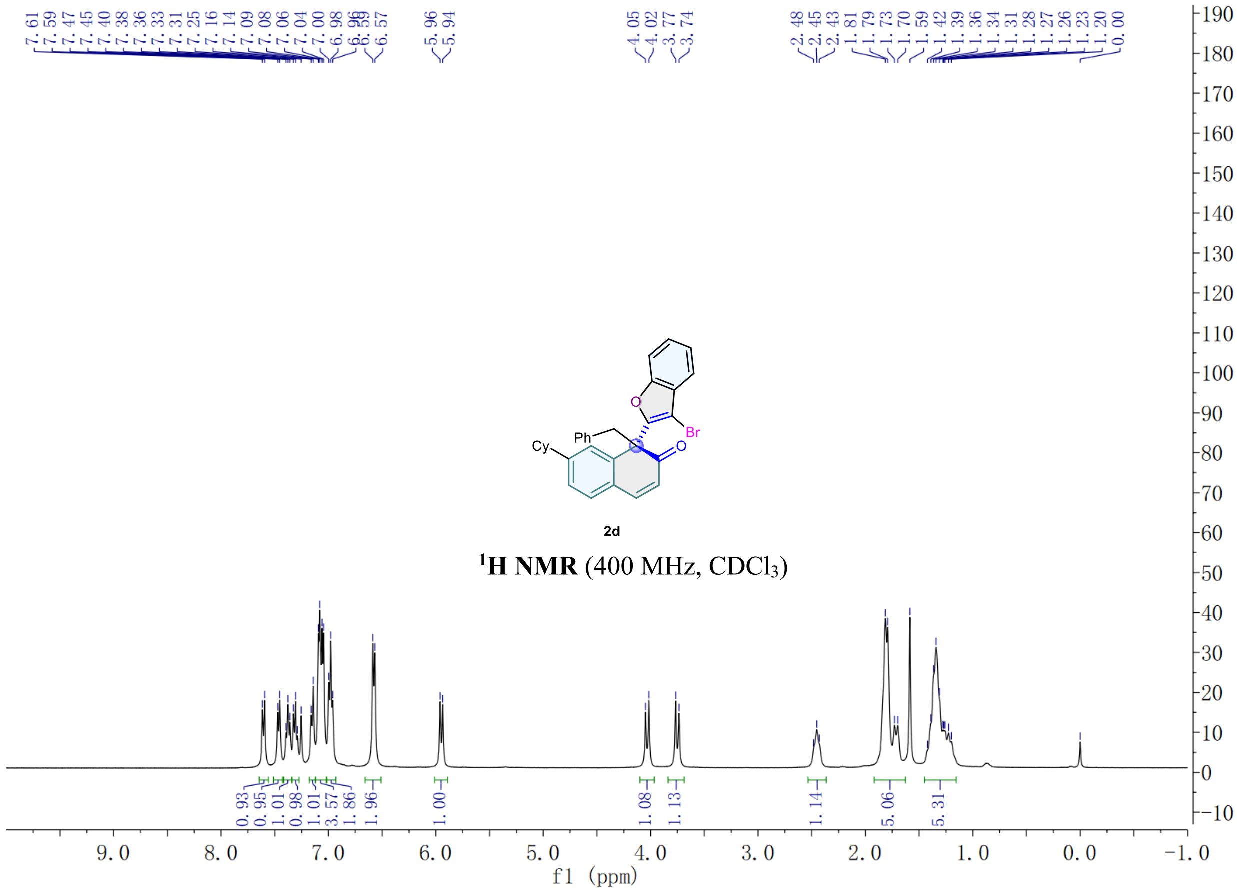
7.61 7.59 7.47 7.45 7.40 7.38 7.36 7.33 7.31 7.25 7.16 7.14 7.09 7.08 7.06 7.04 7.00 6.98 6.96 6.59 6.57 5.96 5.94 4.05 4.02 3.77 3.74 2.48 2.45 2.43 1.81 1.79 1.73 1.70 1.59 1.42 1.39 1.36 1.34 1.31 1.28 1.27 1.26 1.23 1.20 0.00
Cy
Ph
O
Br
2d
^{1}H NMR (400 MHz, CDCl$_3$)
0.93 0.95 1.01 0.98 1.01 3.57 1.86 1.96 1.00 1.08 1.13 1.14 5.06 5.31
f1 (ppm)

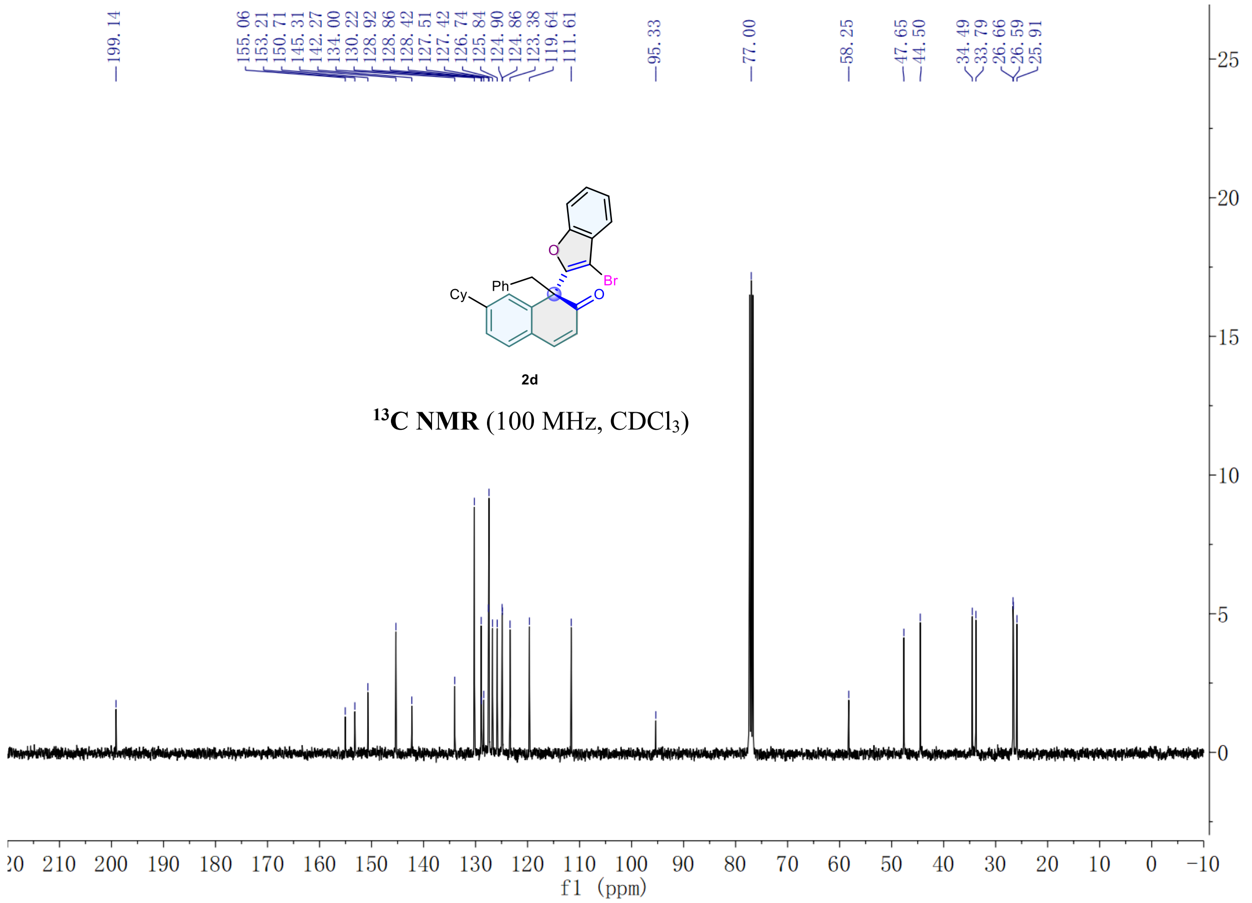
199.14 155.06 153.21 150.71 145.31 142.27 134.00 130.22 128.92 128.86 128.42 127.51 127.42 126.74 125.84 124.90 124.86 123.38 119.64 111.61 95.33 77.00 58.25 47.65 44.50 34.49 33.79 26.66 26.59 25.91
Cy
Ph
O
Br
2d
^{13}C NMR (100 MHz, CDCl$_3$)
f1 (ppm)

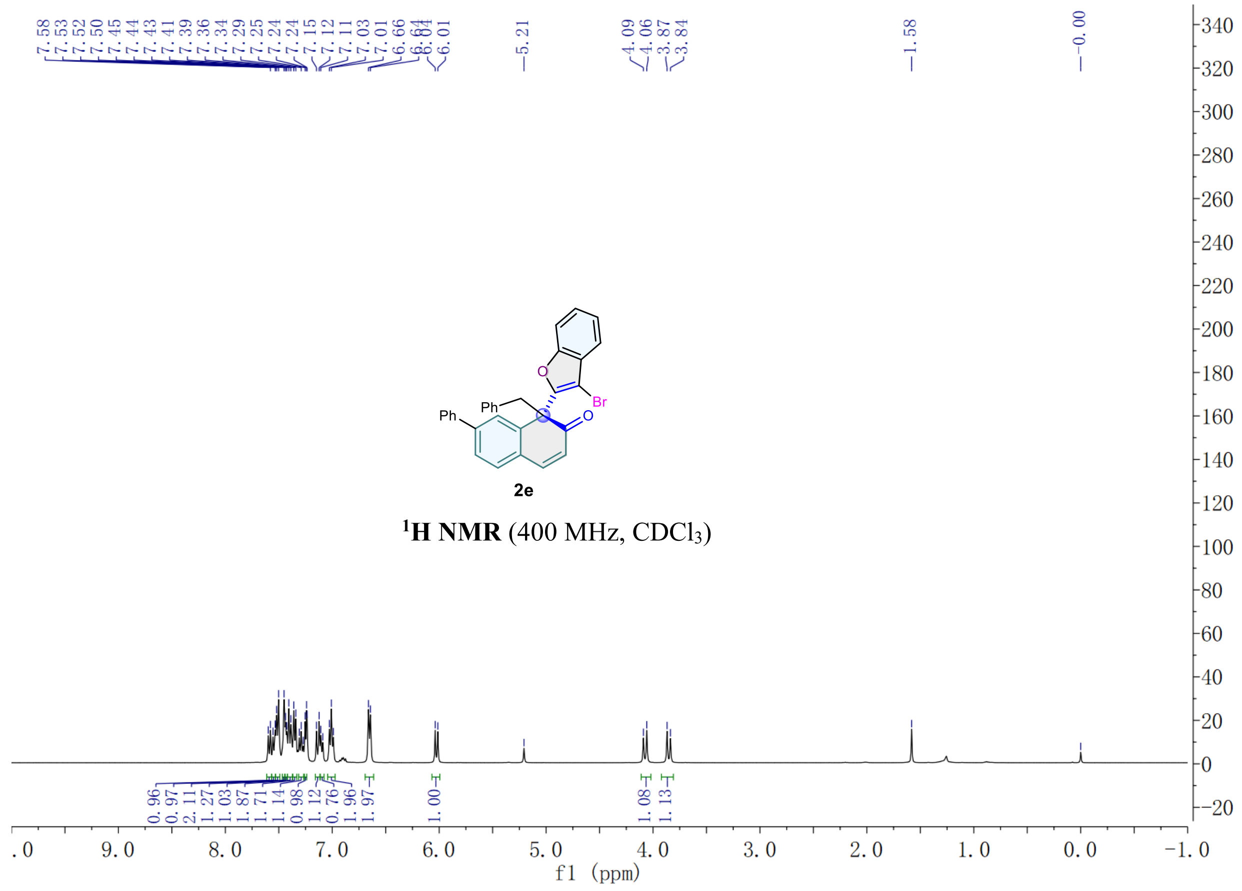

^{1}**H NMR** (400 MHz, $CDCl_3$)

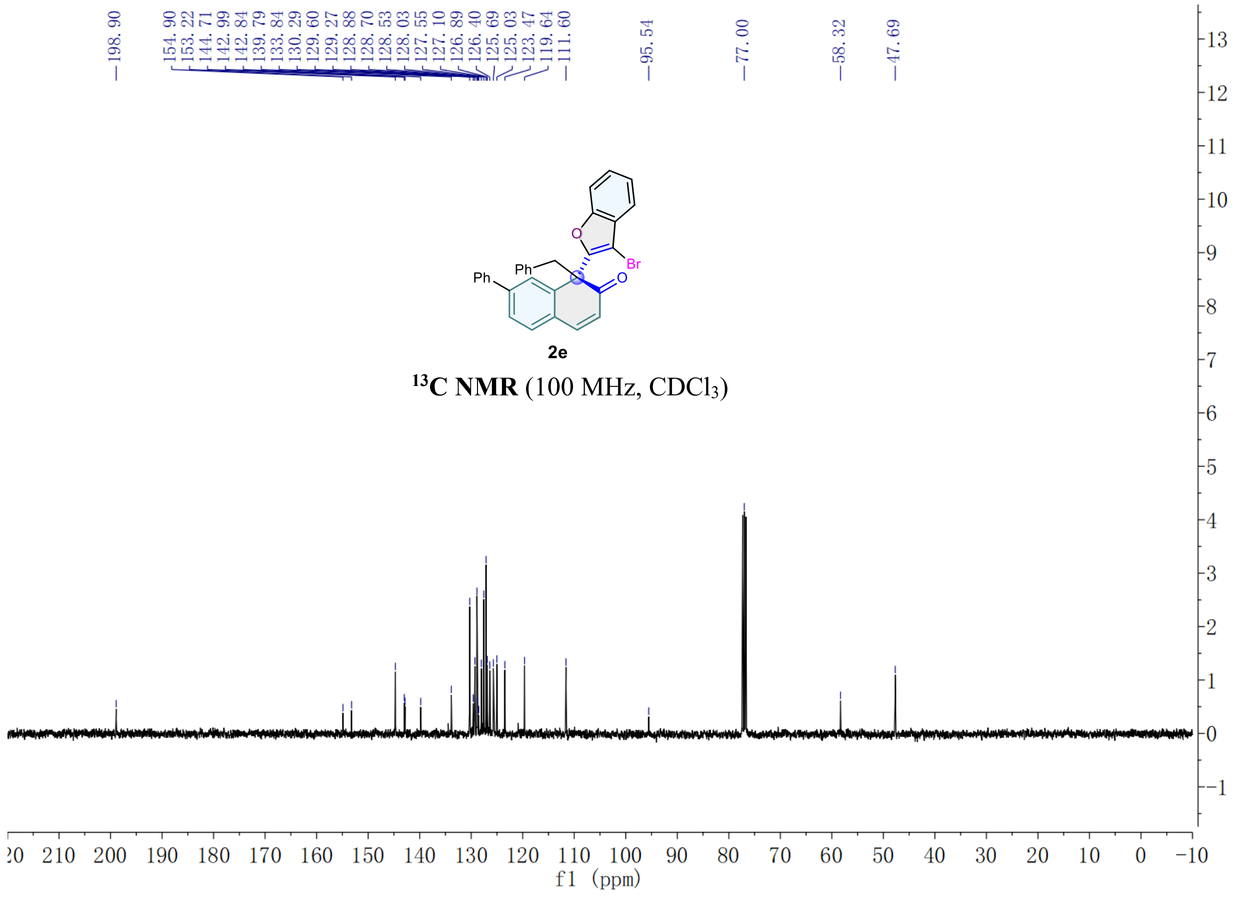

^{13}**C NMR** (100 MHz, $CDCl_3$)

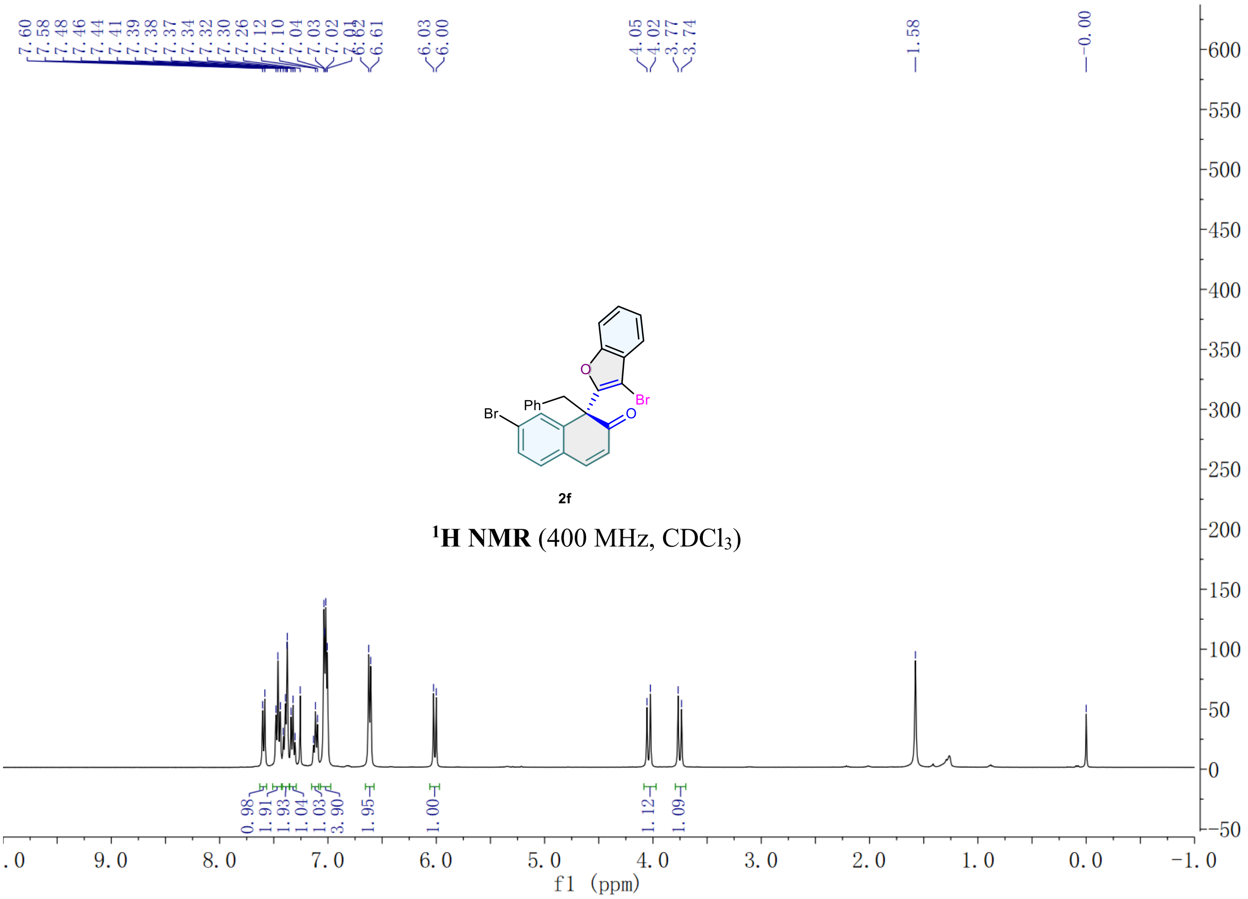

^{1}H NMR (400 MHz, $CDCl_3$)

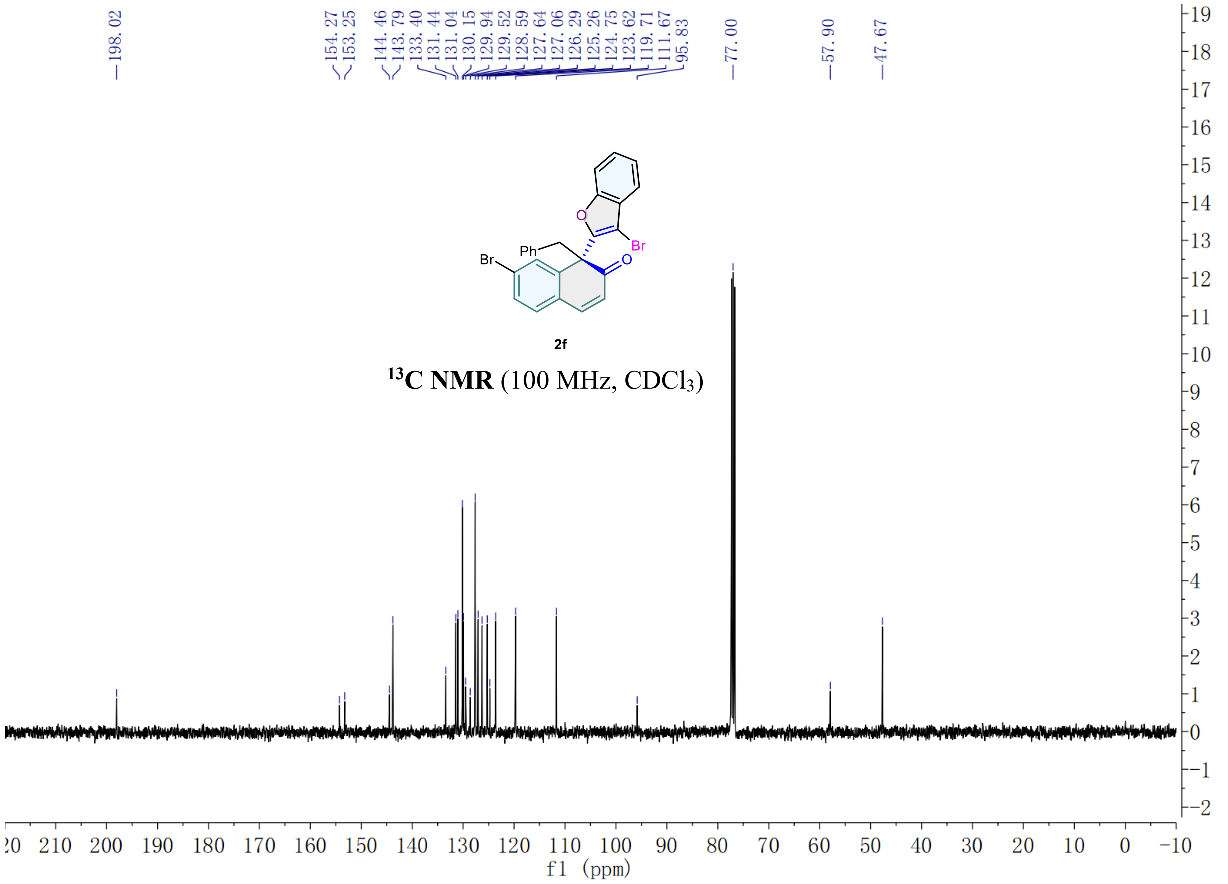

^{13}C NMR (100 MHz, $CDCl_3$)

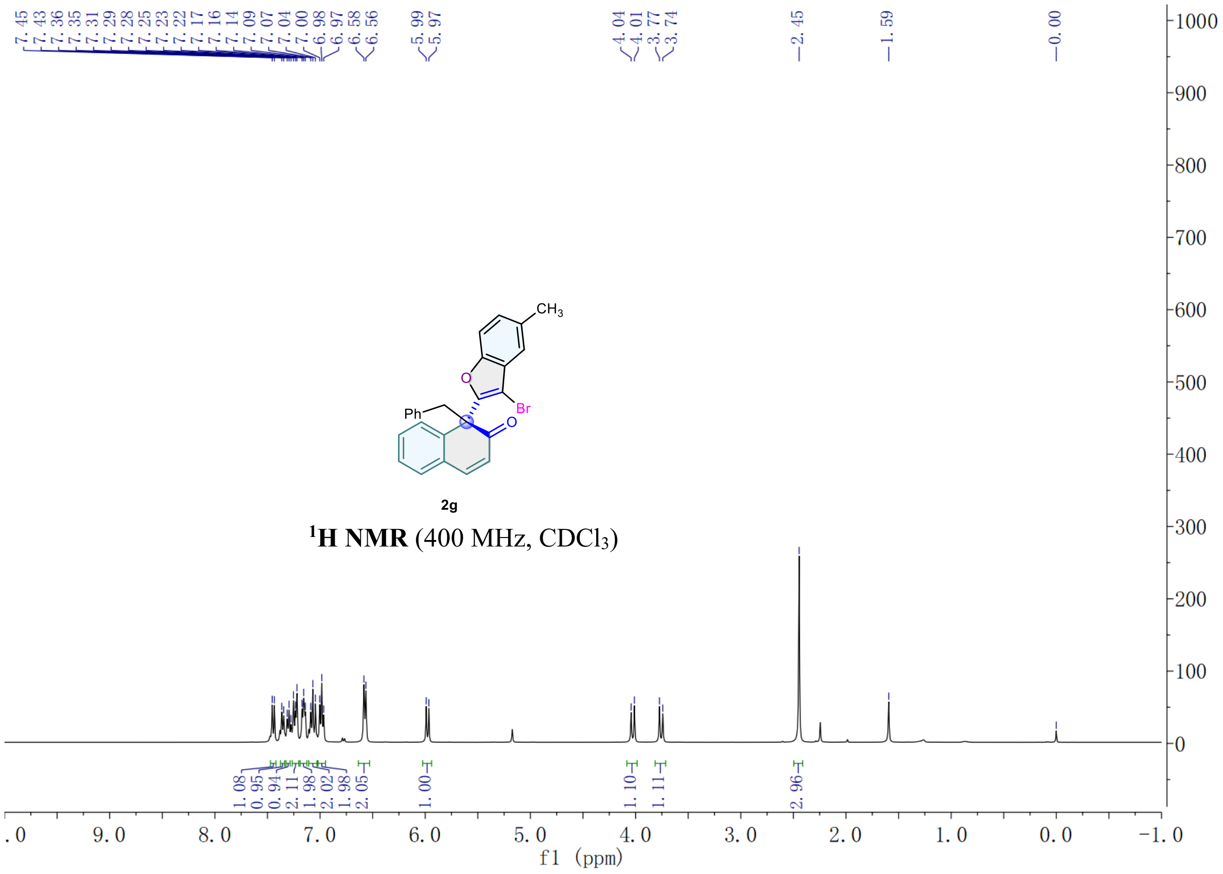

2g

^{1}H NMR (400 MHz, $CDCl_3$)

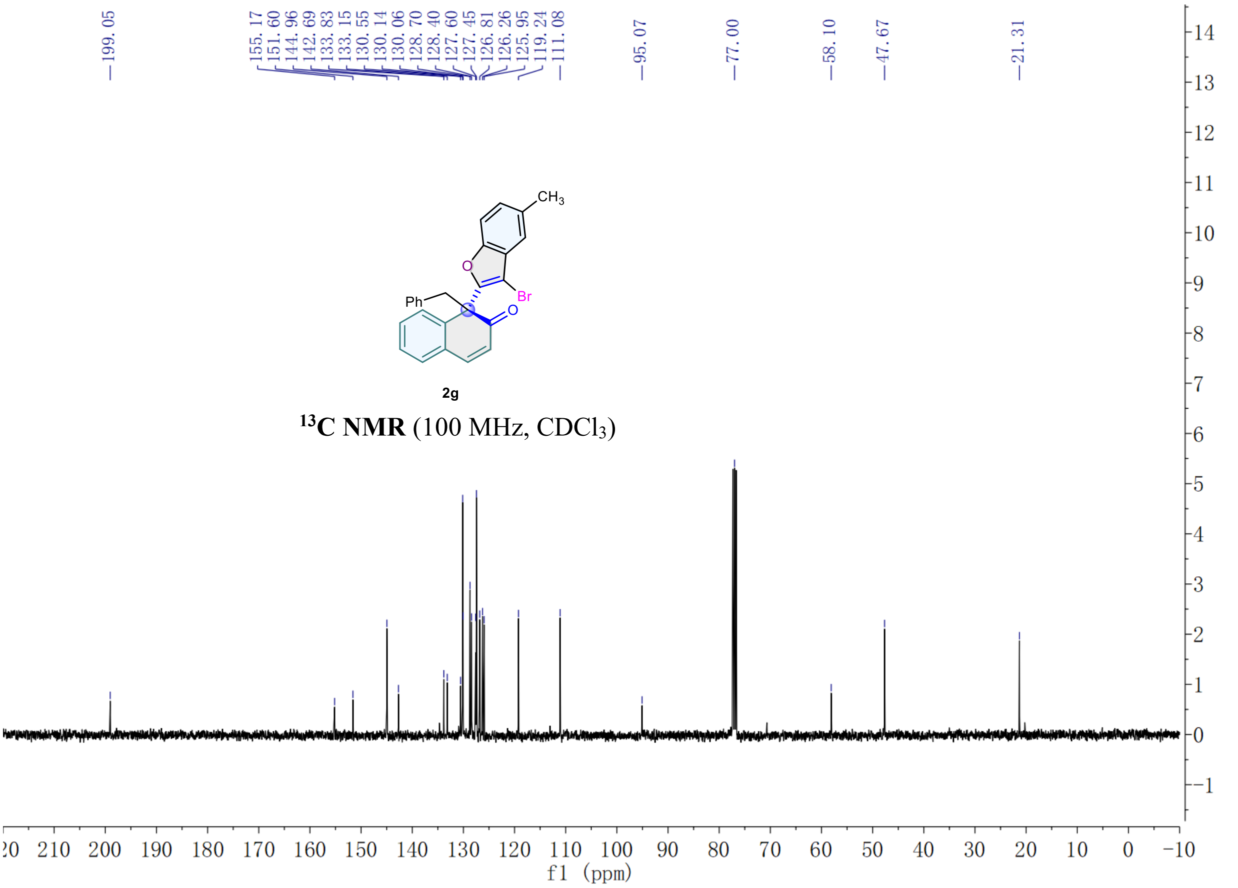

2g

^{13}C NMR (100 MHz, $CDCl_3$)

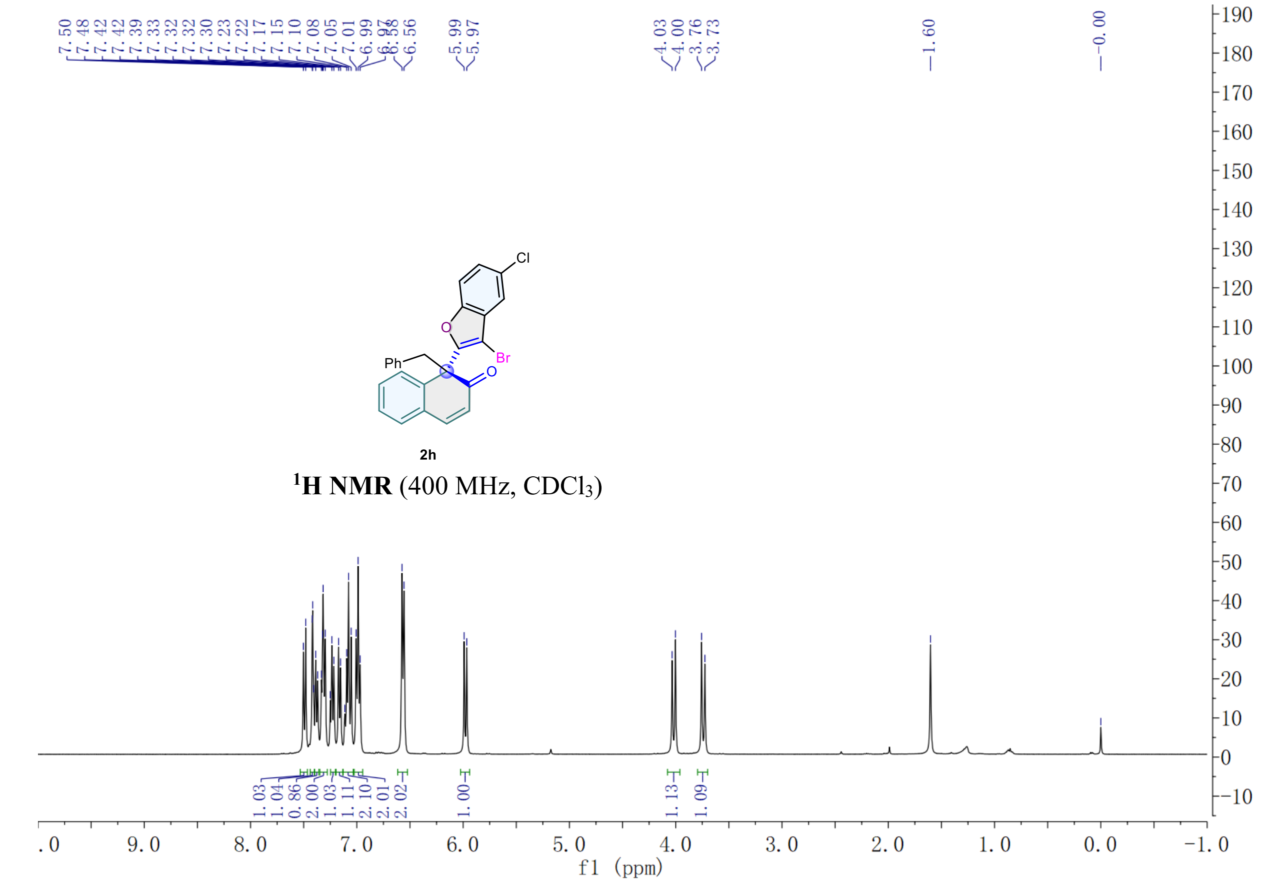
7.50 7.48 7.42 7.42 7.39 7.33 7.32 7.32 7.30 7.23 7.22 7.17 7.15 7.10 7.08 7.05 7.01 6.99 6.97 6.58 6.56
5.99 5.97
4.03 4.00 3.76 3.73
1.60
0.00
Cl
O
Br
Ph
O
2h
¹H NMR (400 MHz, CDCl3)
1.03 1.04 0.86 2.00 1.03 1.11 2.10 2.01 2.02
1.00
1.13
1.09
f1 (ppm)

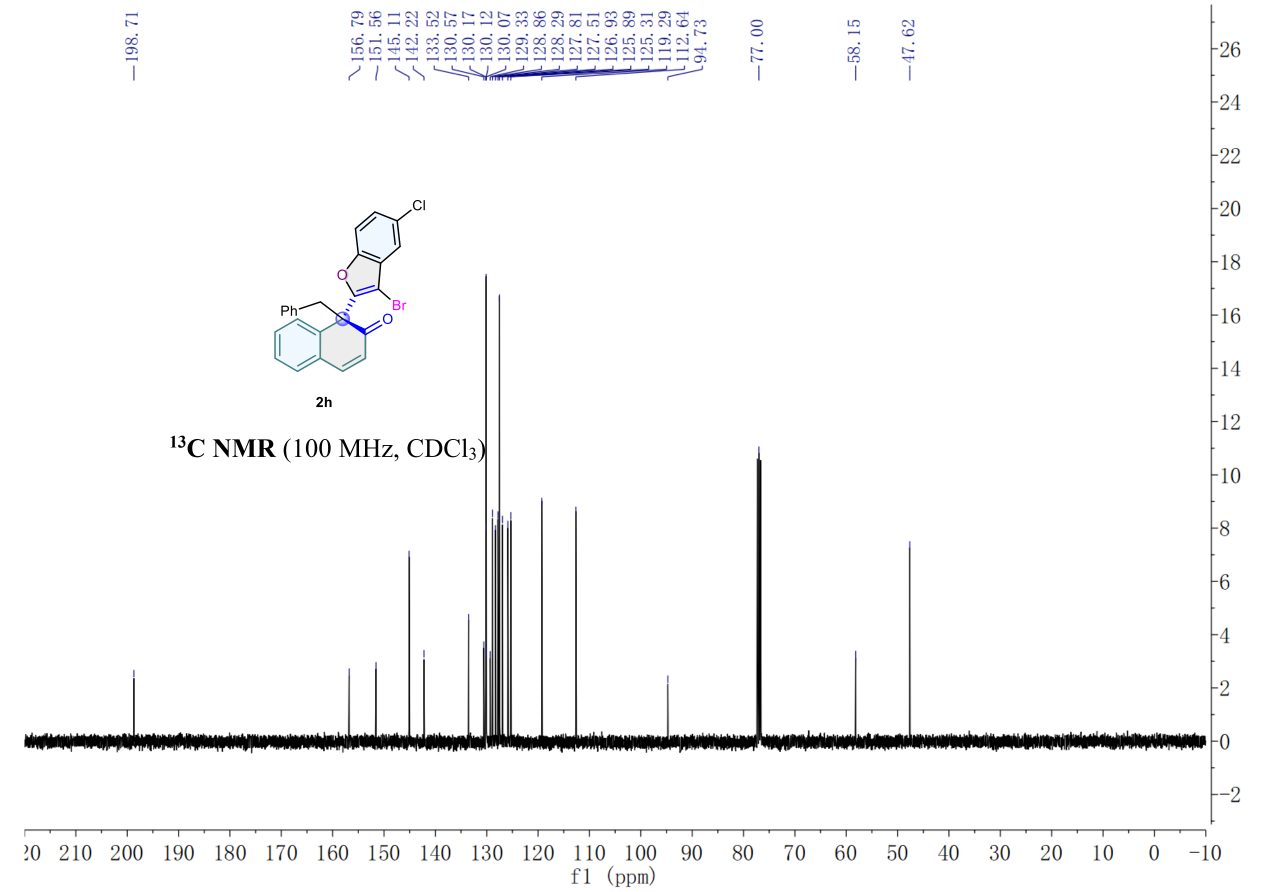
198.71
156.79 151.56 145.11 142.22 133.52 130.57 130.17 130.12 130.07 129.33 128.86 128.29 127.81 127.51 126.93 125.89 125.31 119.29 112.64 94.73
77.00
58.15
47.62
Cl
O
Br
Ph
O
2h
¹³C NMR (100 MHz, CDCl3)
f1 (ppm)

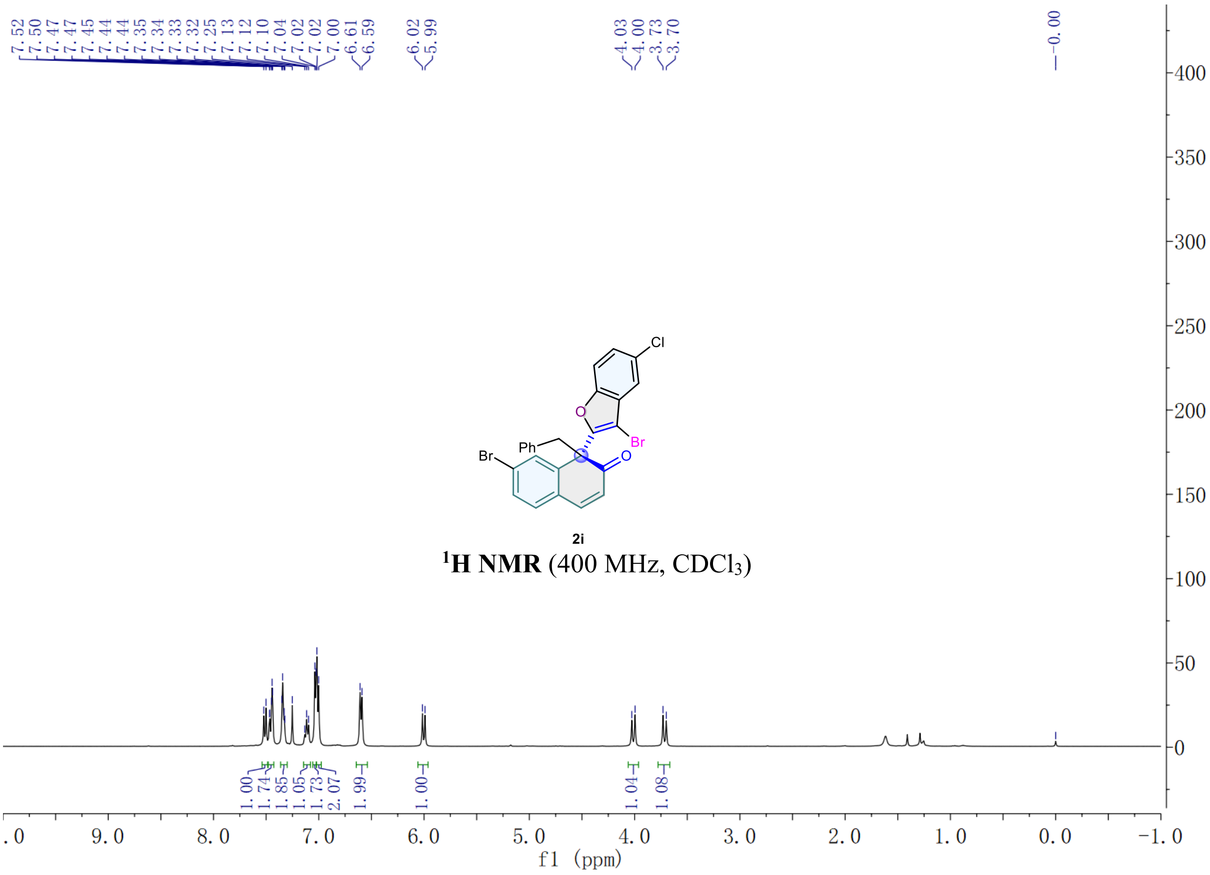

7.52 7.50 7.47 7.47 7.45 7.44 7.44 7.35 7.34 7.33 7.32 7.25 7.13 7.12 7.10 7.04 7.02 7.02 7.00 6.61 6.59 6.02 5.99 4.03 4.00 3.73 3.70 0.00
Cl
O
Br
Ph
Br
O
2i
^{1}H NMR (400 MHz, CDCl$_3$)
1.00 1.74 1.85 1.05 1.73 2.07 1.99 1.00 1.04 1.08
f1 (ppm)

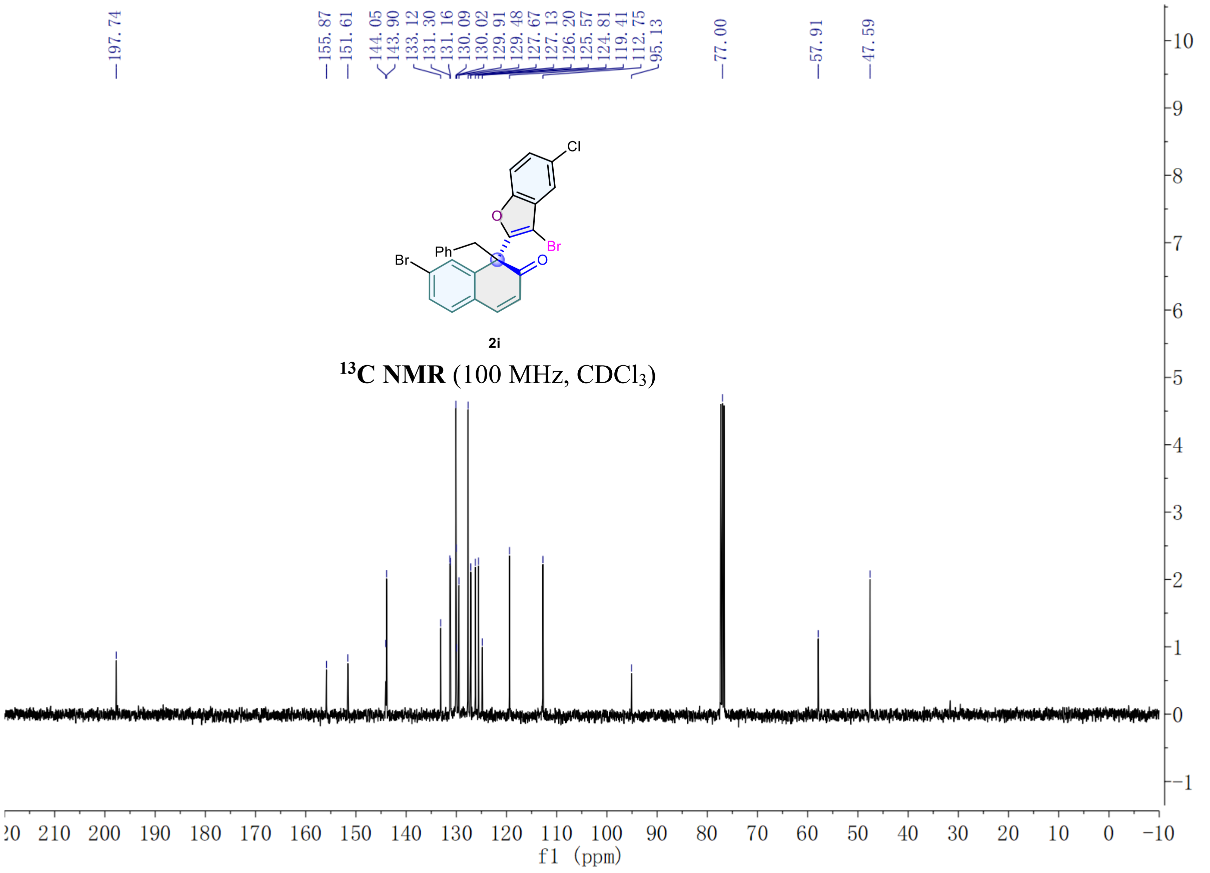

197.74 155.87 151.61 144.05 143.90 133.12 131.30 131.16 130.09 130.02 129.91 129.48 127.67 127.13 126.20 125.57 124.81 119.41 112.75 95.13 77.00 57.91 47.59
Cl
O
Br
Ph
Br
O
2i
^{13}C NMR (100 MHz, CDCl$_3$)
f1 (ppm)

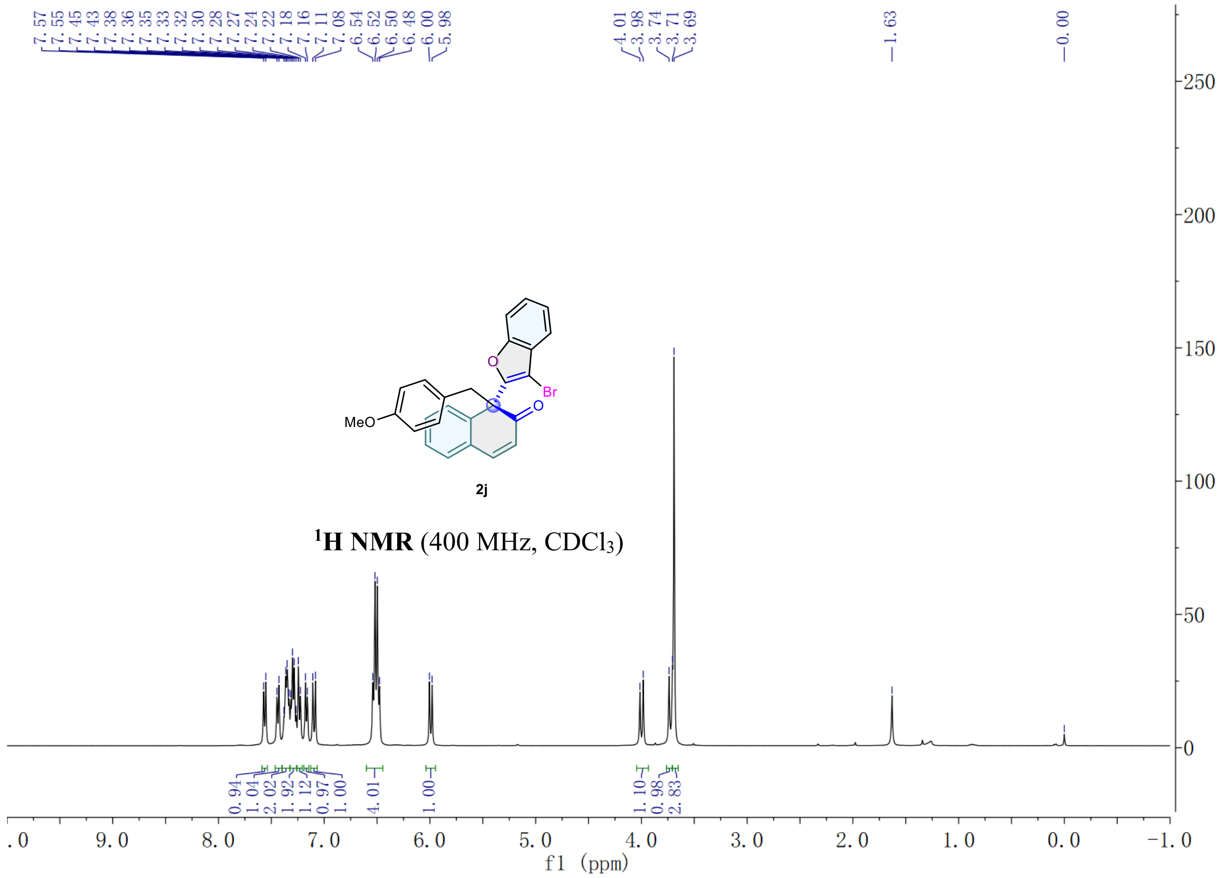

MeO
O
Br
O
2j
¹H NMR (400 MHz, CDCl₃)
f1 (ppm)

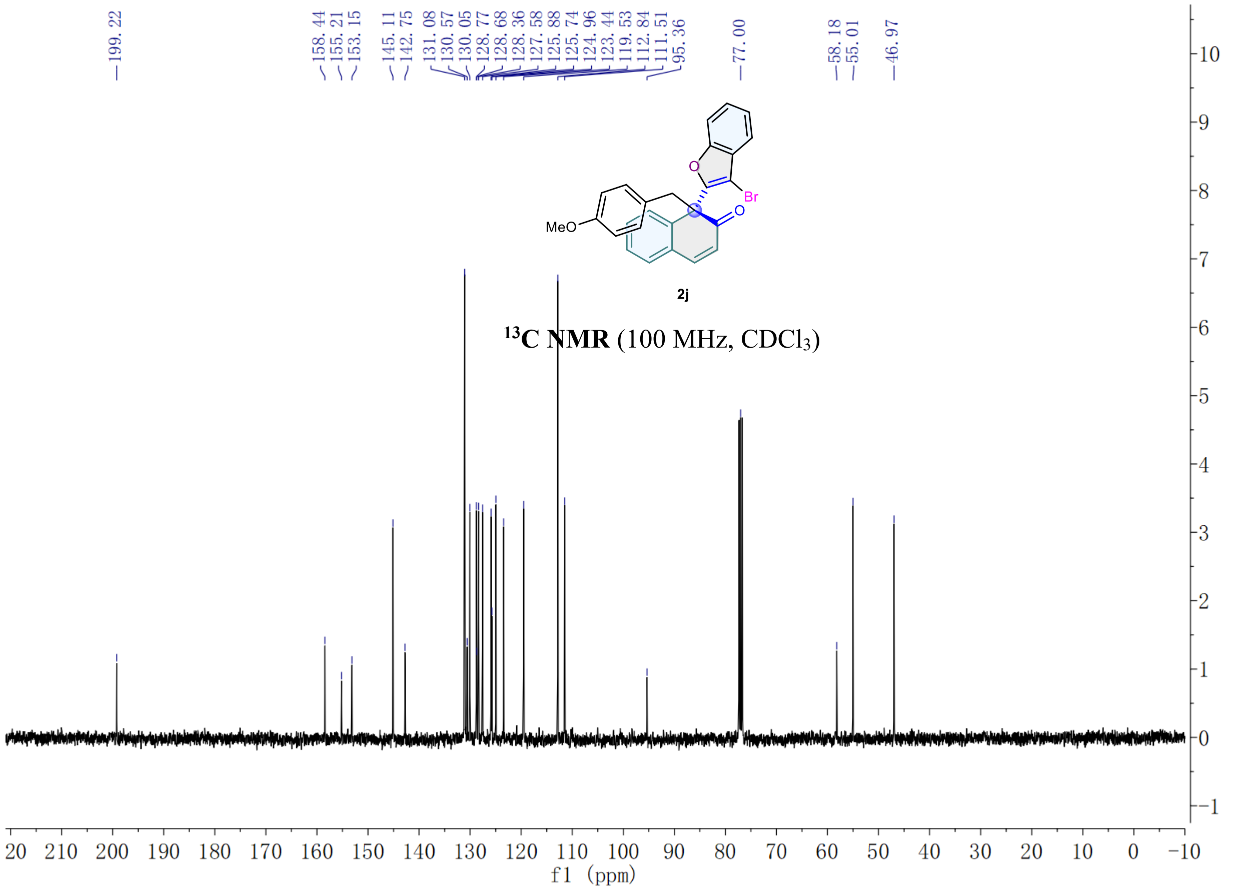

MeO
O
Br
O
2j
¹³C NMR (100 MHz, CDCl₃)
f1 (ppm)

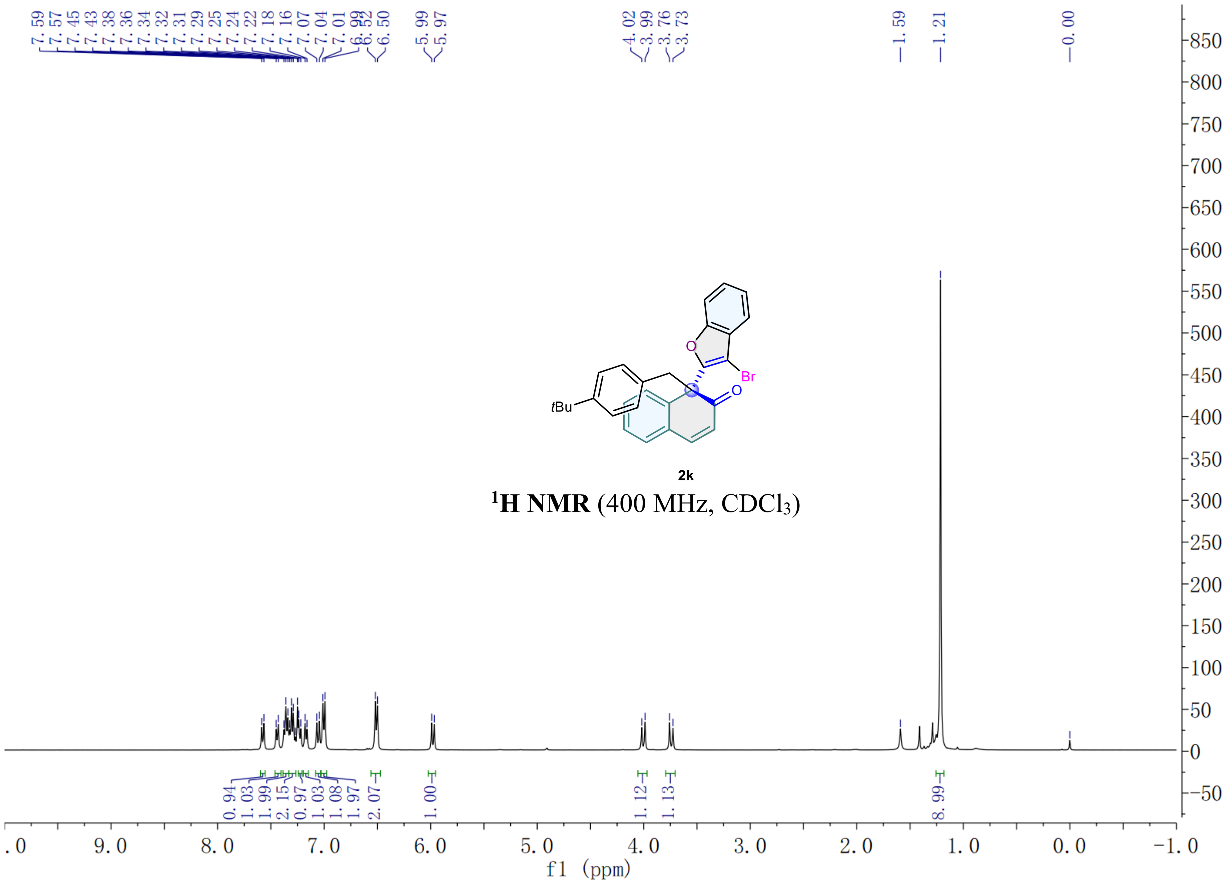

2k

^{1}H NMR (400 MHz, $CDCl_3$)

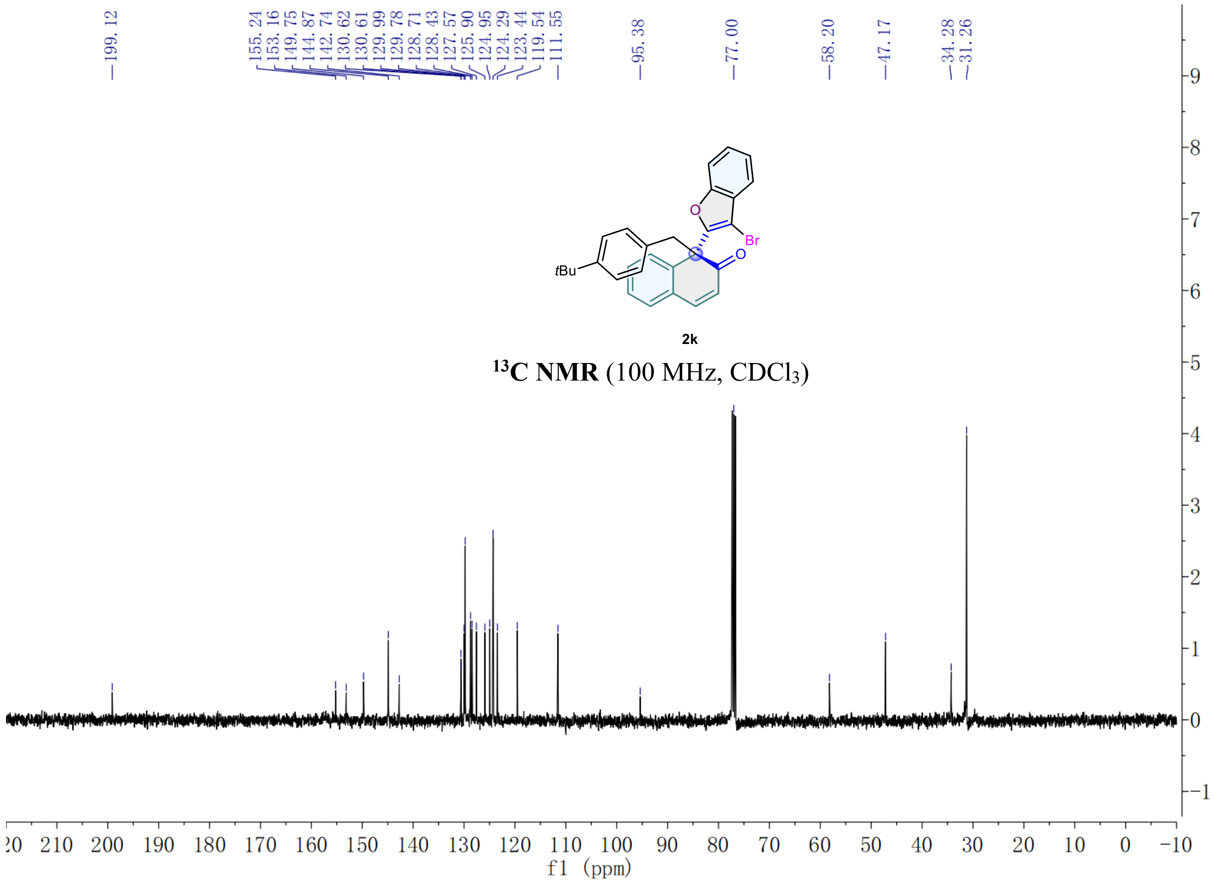

2k

^{13}C NMR (100 MHz, $CDCl_3$)

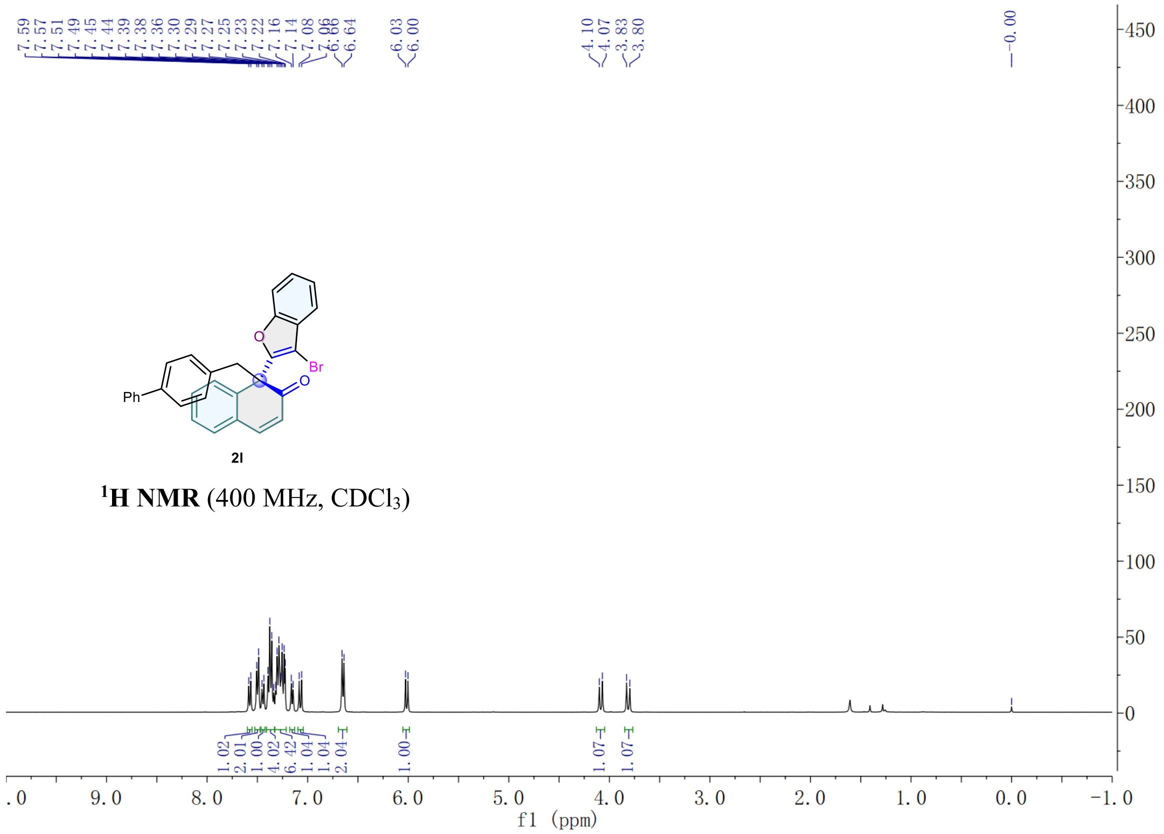

^{1}**H NMR** (400 MHz, $CDCl_3$)

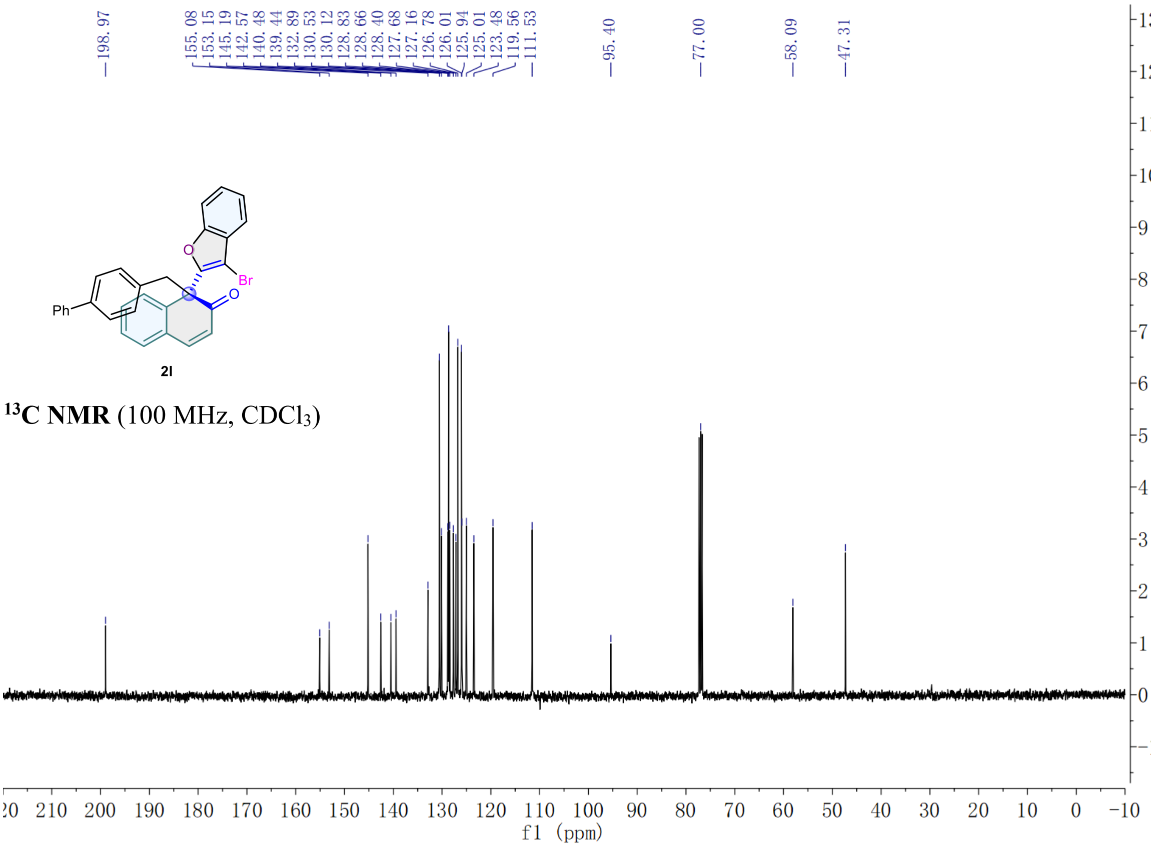

^{13}**C NMR** (100 MHz, $CDCl_3$)

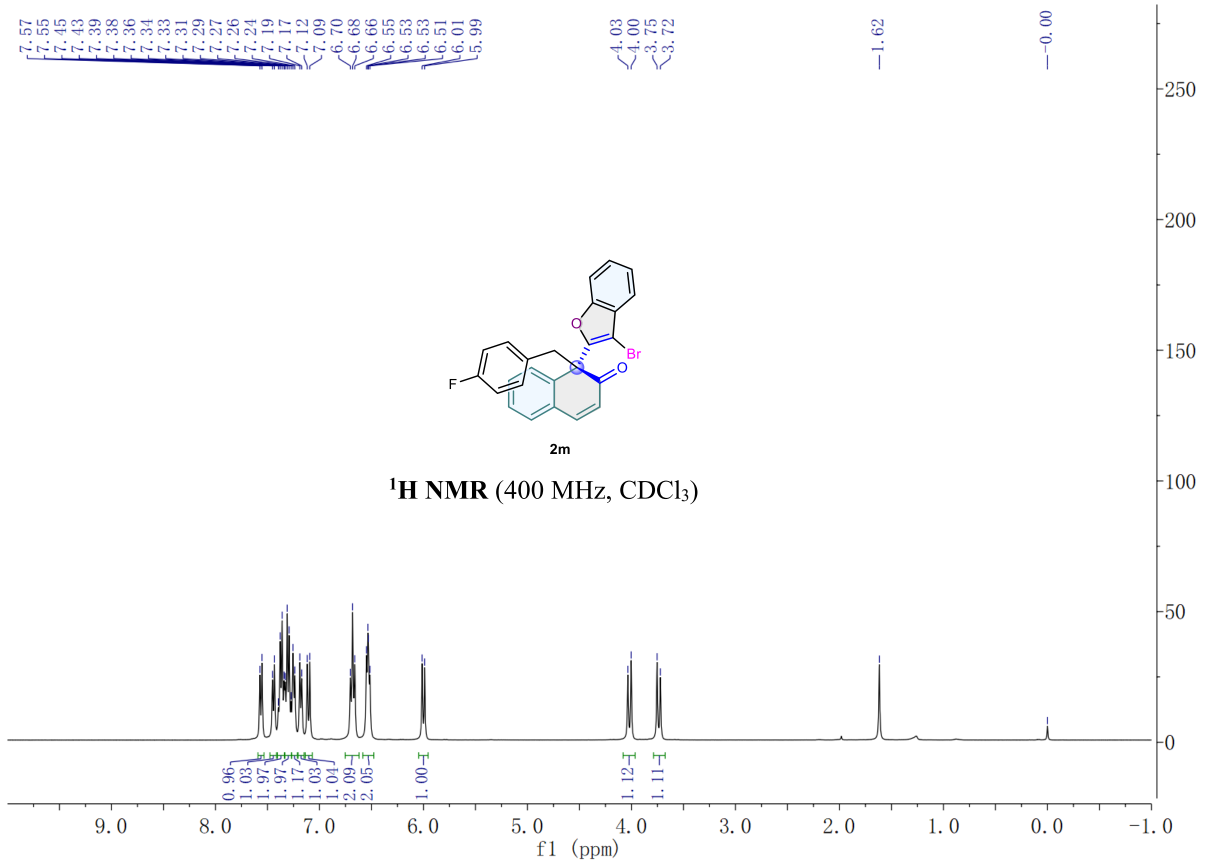

2m

^{1}H NMR (400 MHz, $CDCl_3$)

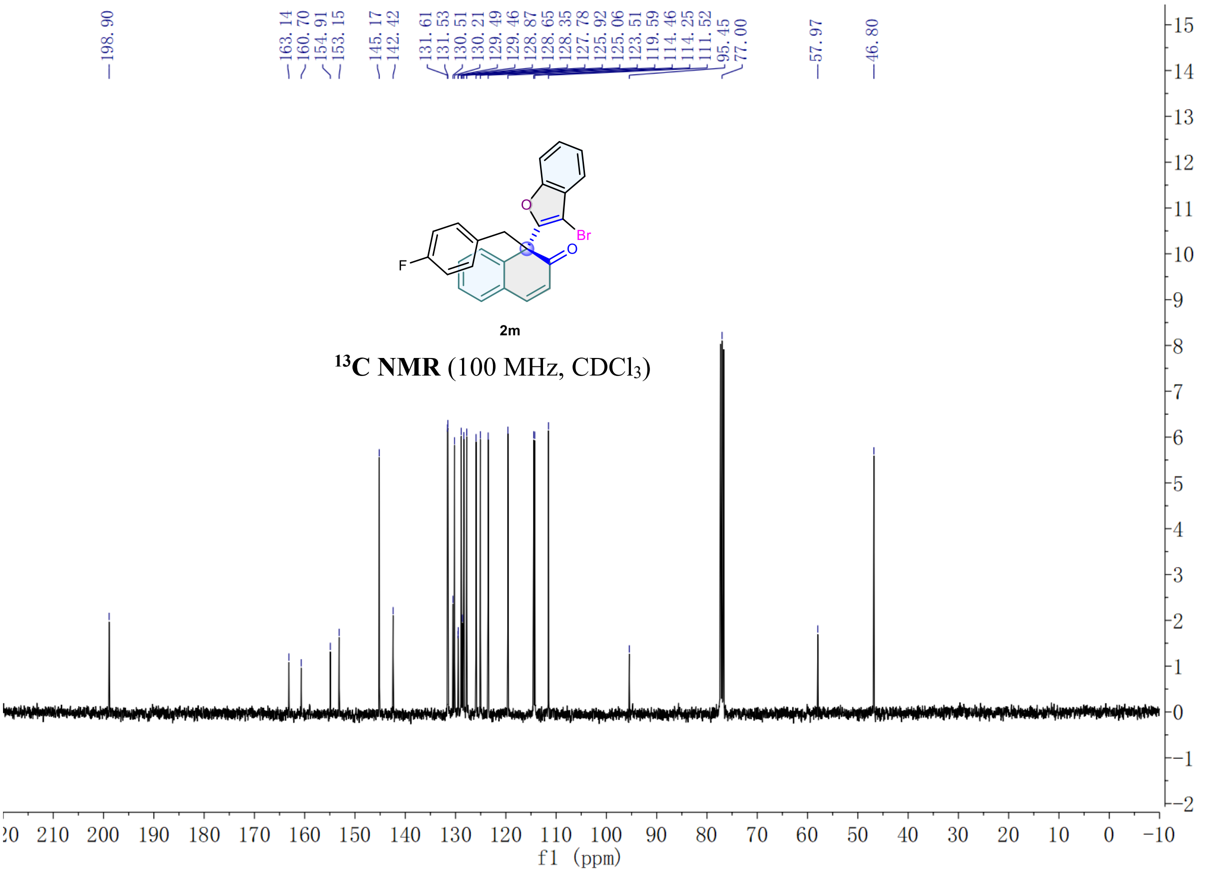

2m

^{13}C NMR (100 MHz, $CDCl_3$)

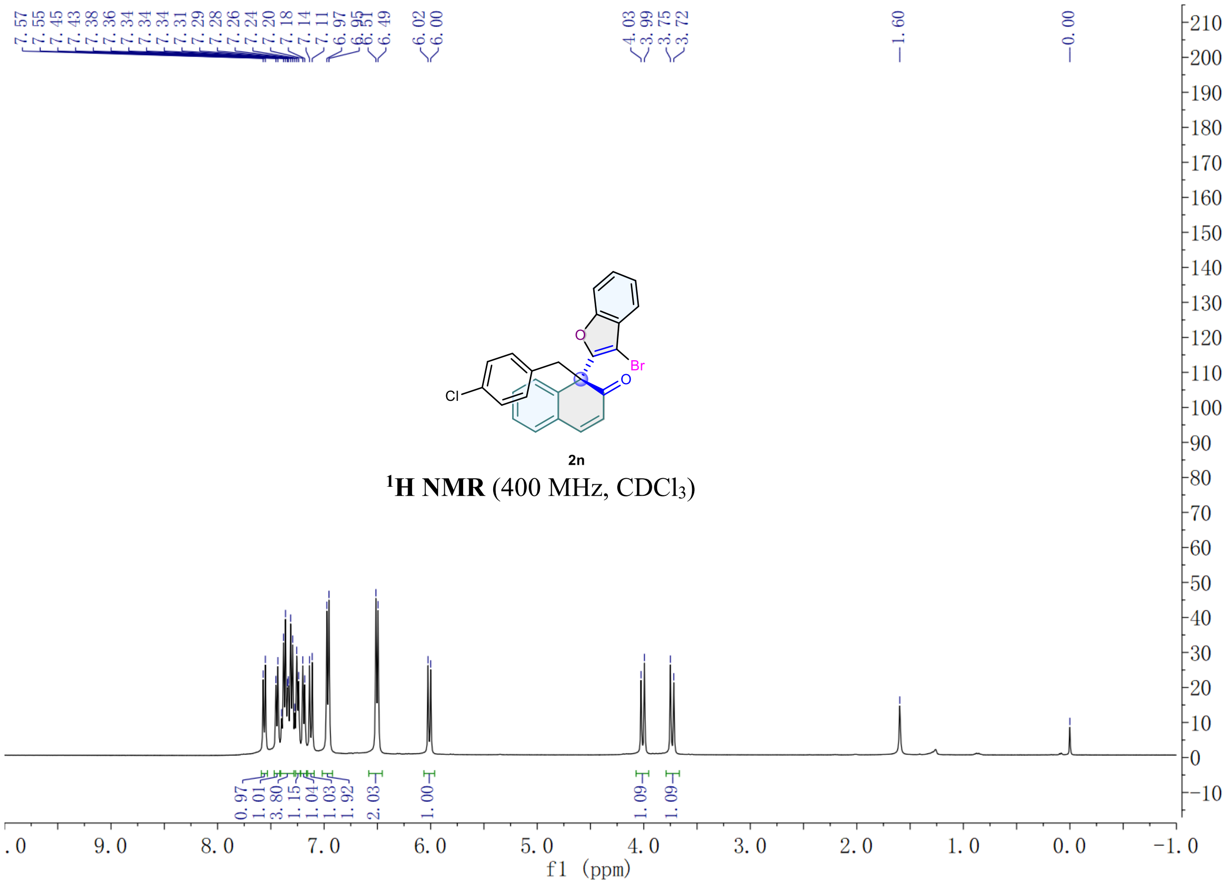

2n

^{1}H NMR (400 MHz, $CDCl_3$)

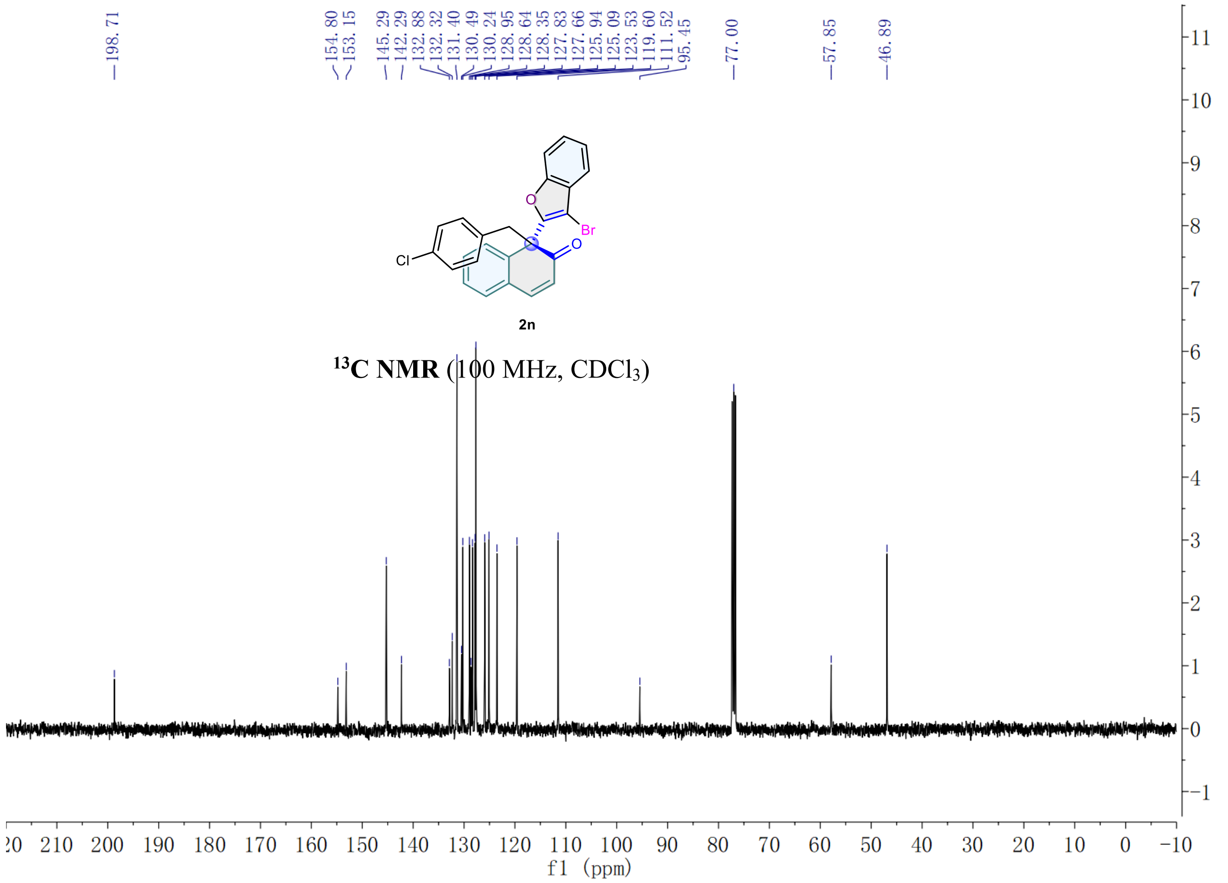

2n

^{13}C NMR (100 MHz, $CDCl_3$)

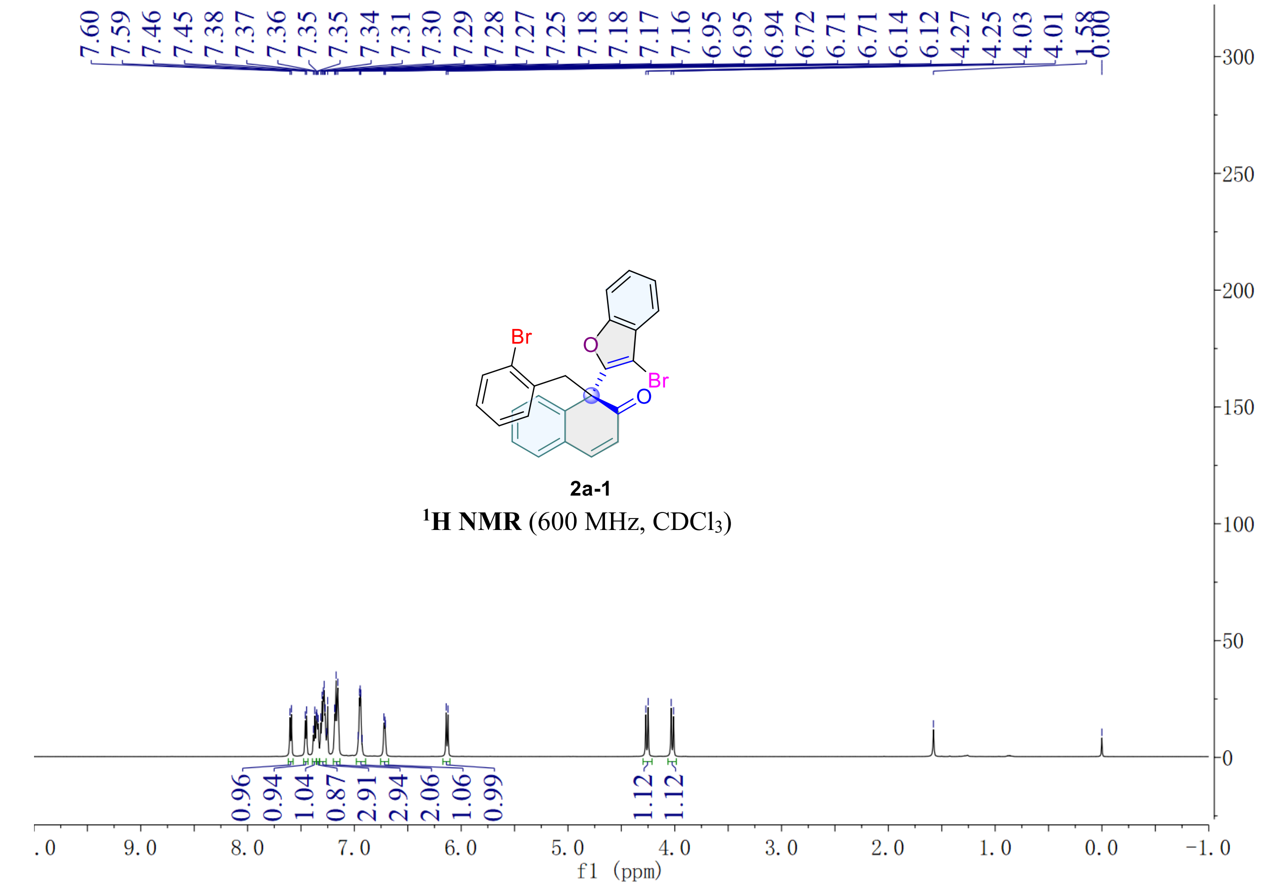

2a-1

^{1}H NMR (600 MHz, $CDCl_3$)

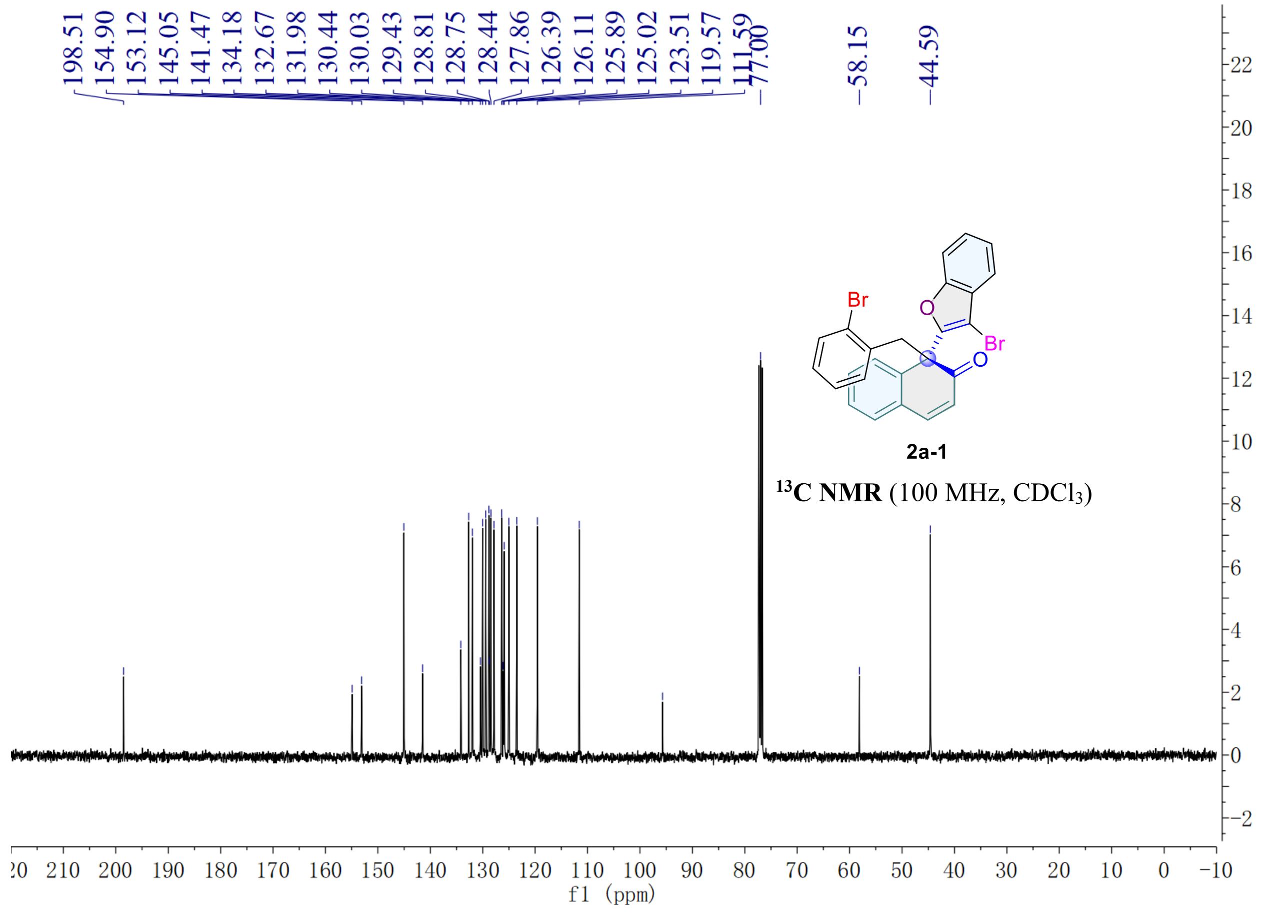

2a-1

^{13}C NMR (100 MHz, $CDCl_3$)

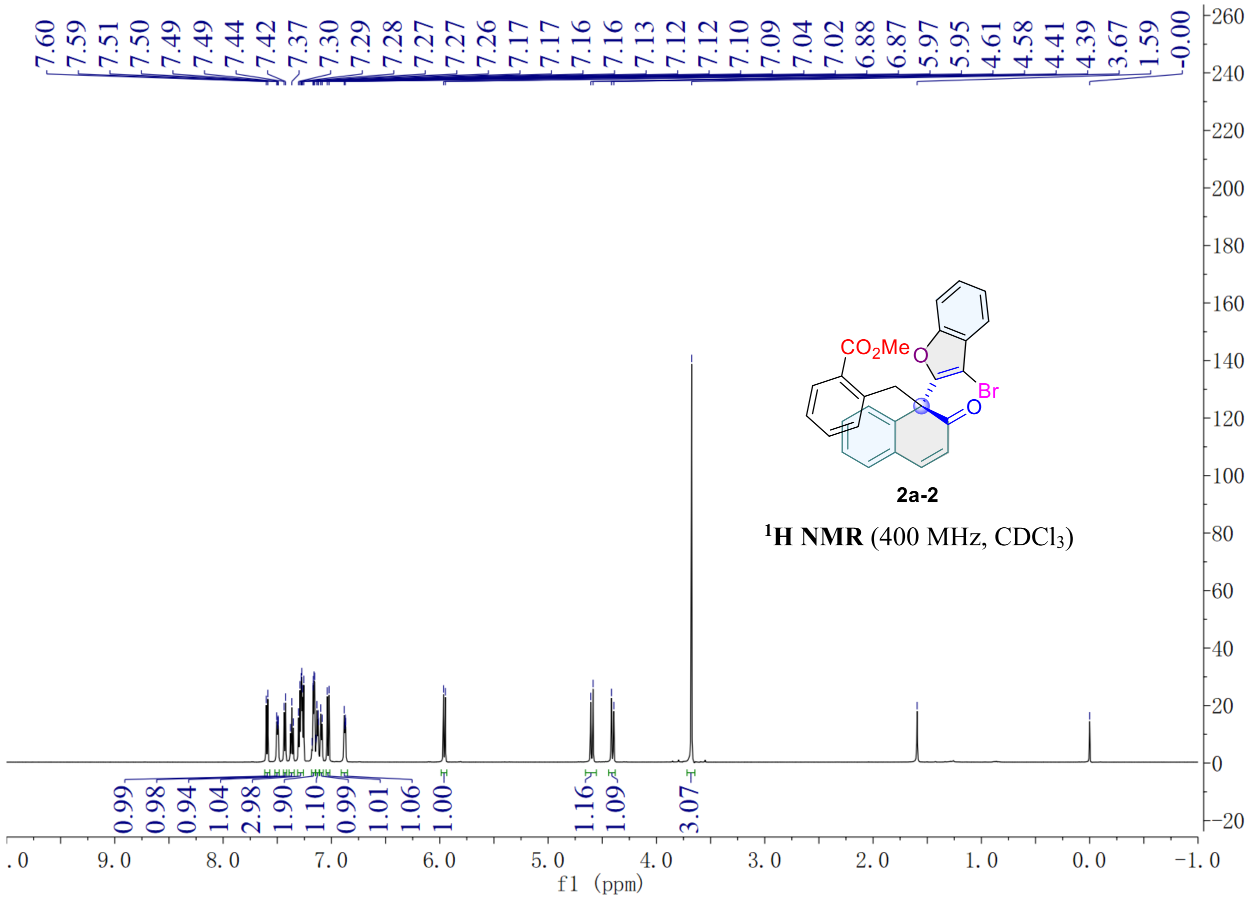
7.60 7.59 7.51 7.50 7.49 7.49 7.44 7.42 7.37 7.30 7.29 7.28 7.27 7.27 7.26 7.17 7.17 7.16 7.16 7.13 7.12 7.12 7.10 7.09 7.04 7.02 6.88 6.87 5.97 5.95 4.61 4.58 4.41 4.39 3.67 1.59 -0.00
CO2Me
O
Br
O
2a-2
1H NMR (400 MHz, CDCl3)
0.99 0.98 0.94 1.04 2.98 1.90 1.10 0.99 1.01 1.06 1.00 1.16 1.09 3.07
f1 (ppm)

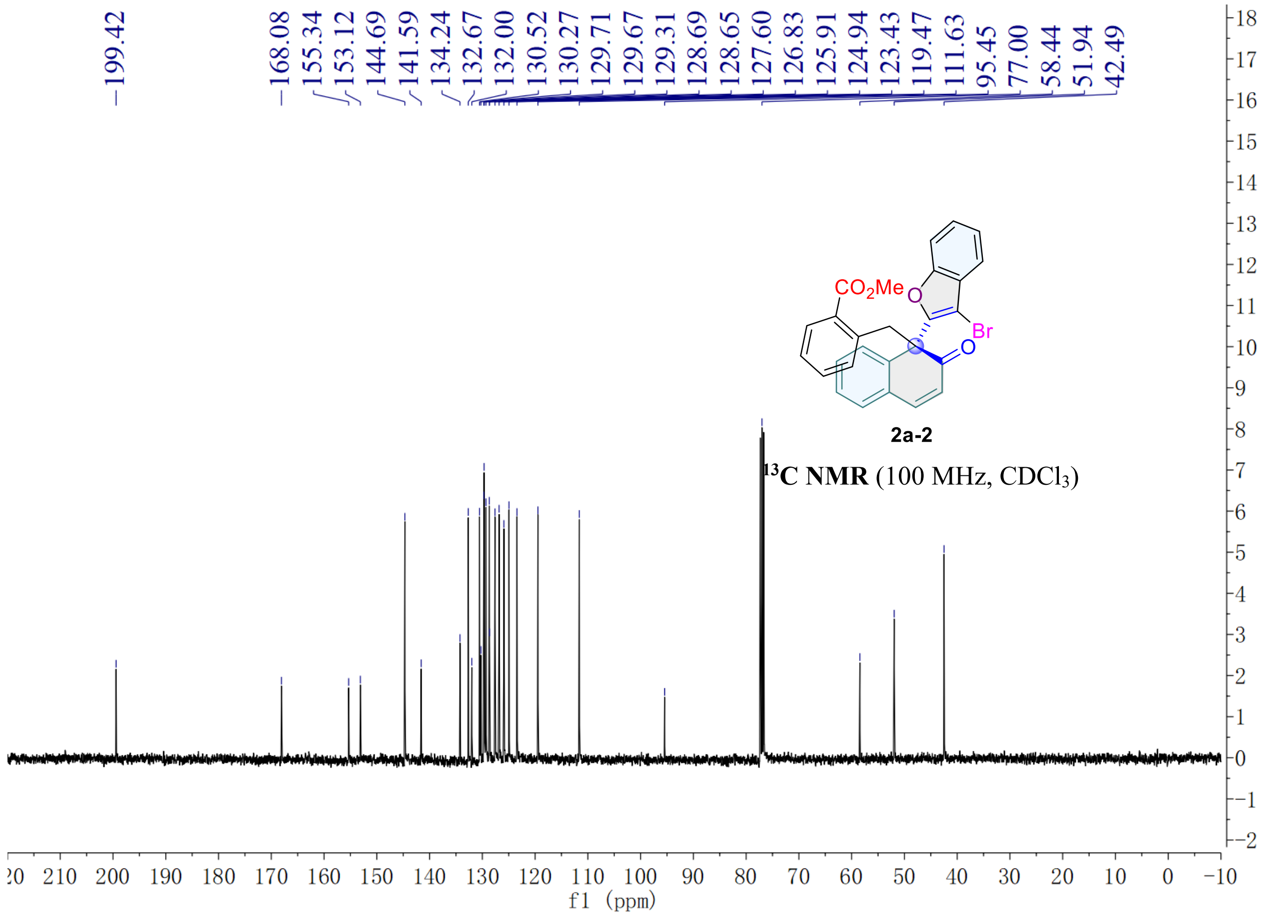
199.42
168.08 155.34 153.12 144.69 141.59 134.24 132.67 132.00 130.52 130.27 129.71 129.67 129.31 128.69 128.65 127.60 126.83 125.91 124.94 123.43 119.47 111.63 95.45 77.00 58.44 51.94 42.49
CO2Me
O
Br
O
2a-2
13C NMR (100 MHz, CDCl3)
f1 (ppm)

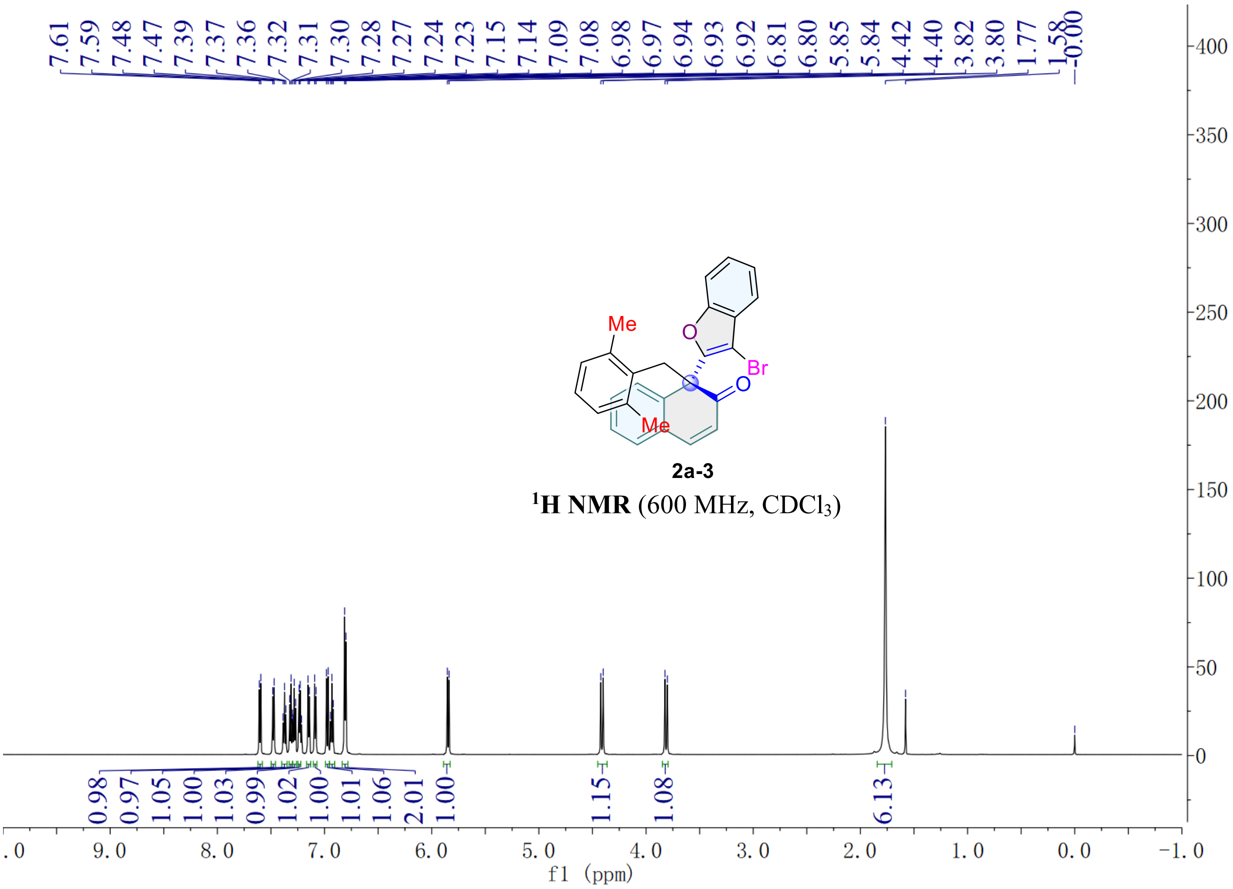

2a-3

^{1}H NMR (600 MHz, $CDCl_3$)

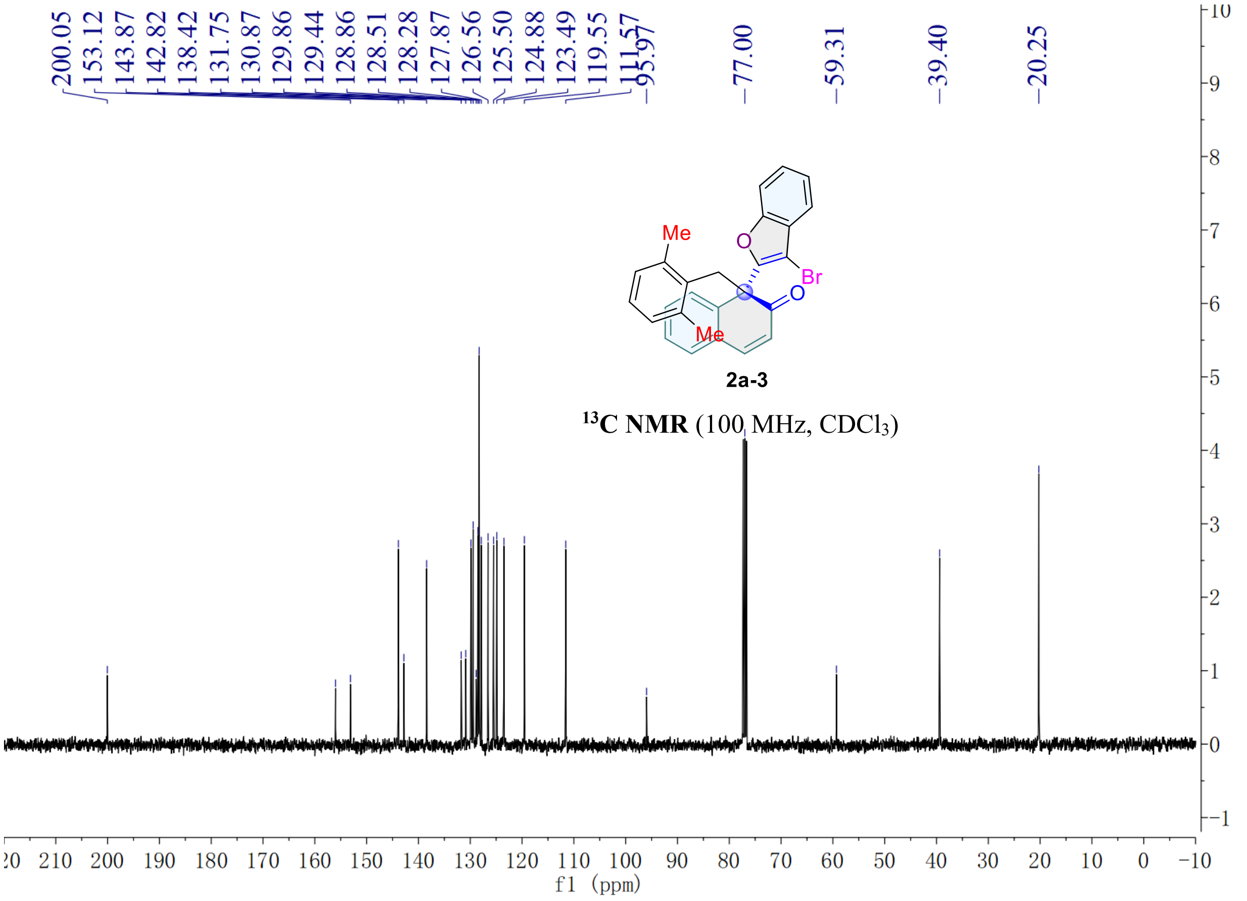

2a-3

^{13}C NMR (100 MHz, $CDCl_3$)

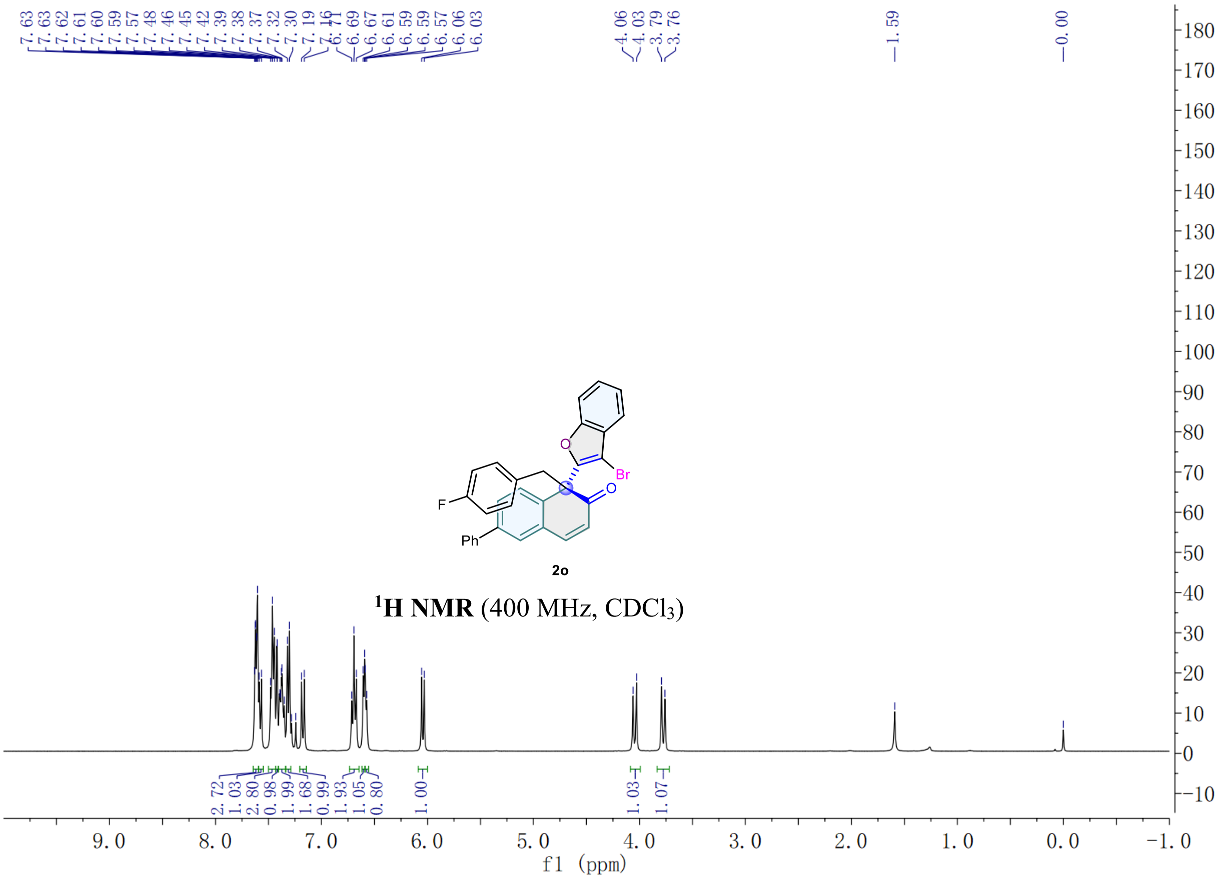

^{1}H NMR (400 MHz, $CDCl_3$)

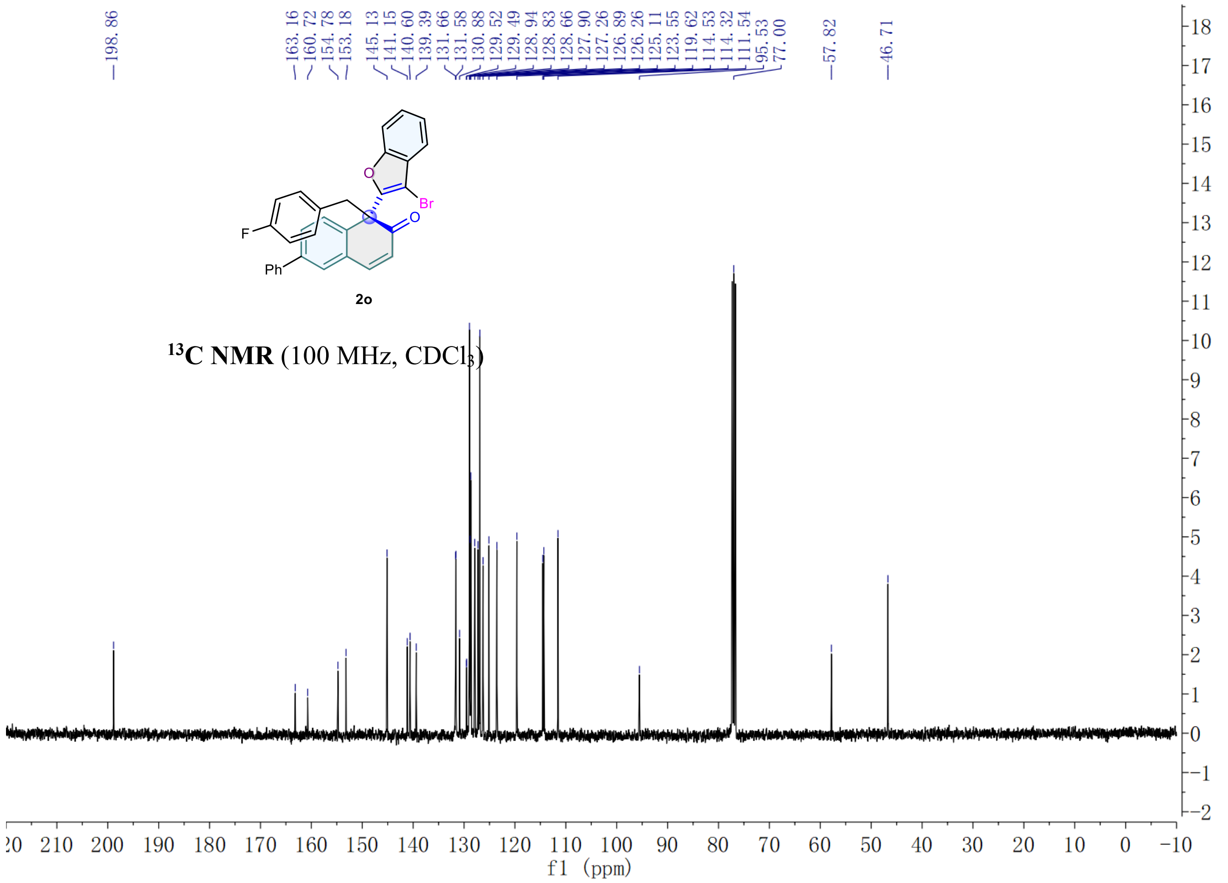

^{13}C NMR (100 MHz, $CDCl_3$)

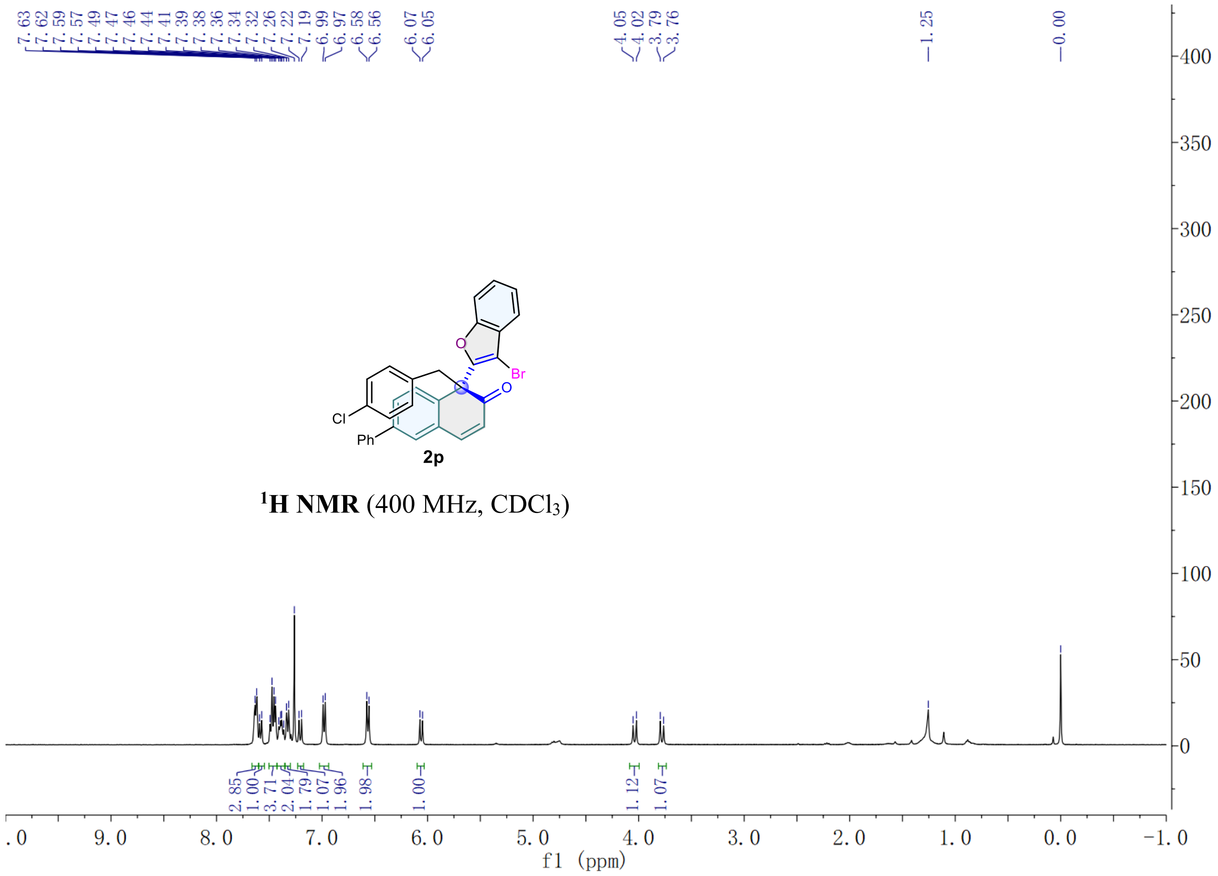

^{1}H NMR (400 MHz, $CDCl_3$)

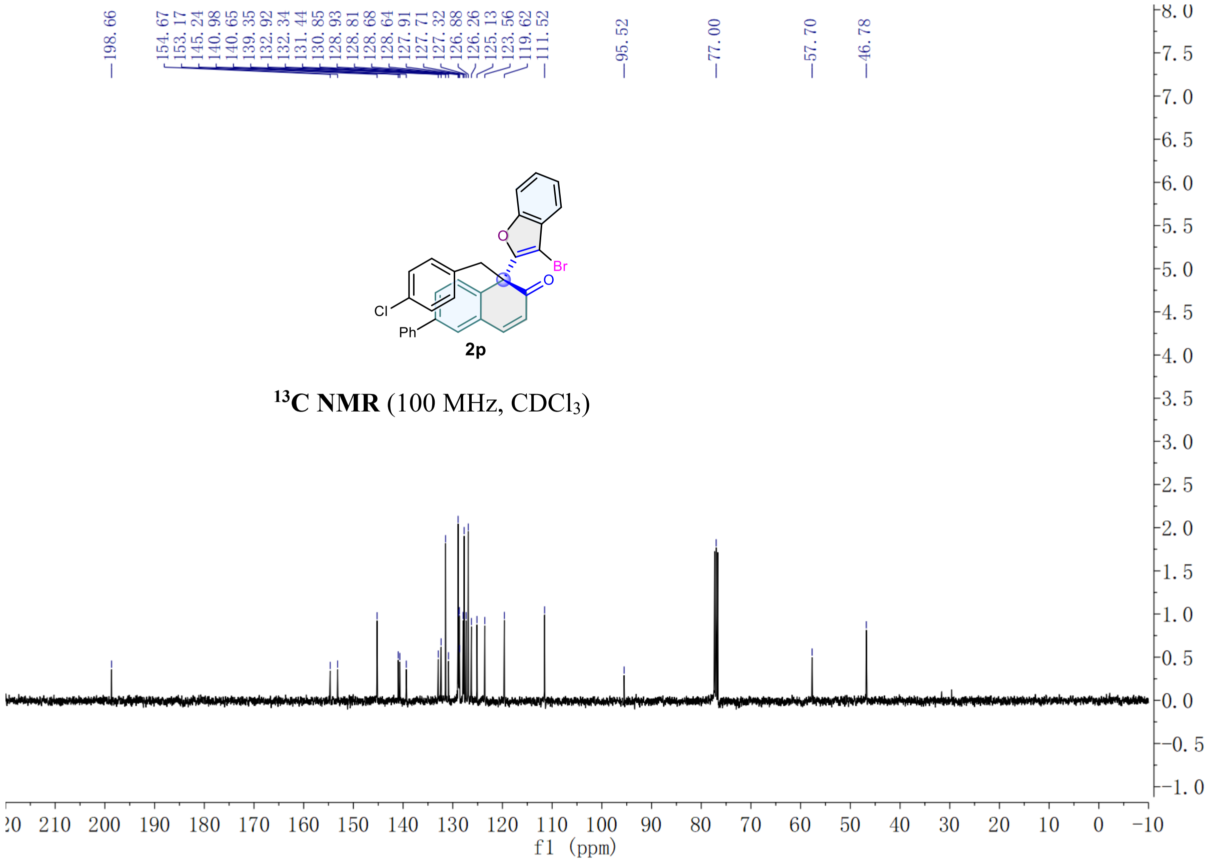

^{13}C NMR (100 MHz, $CDCl_3$)

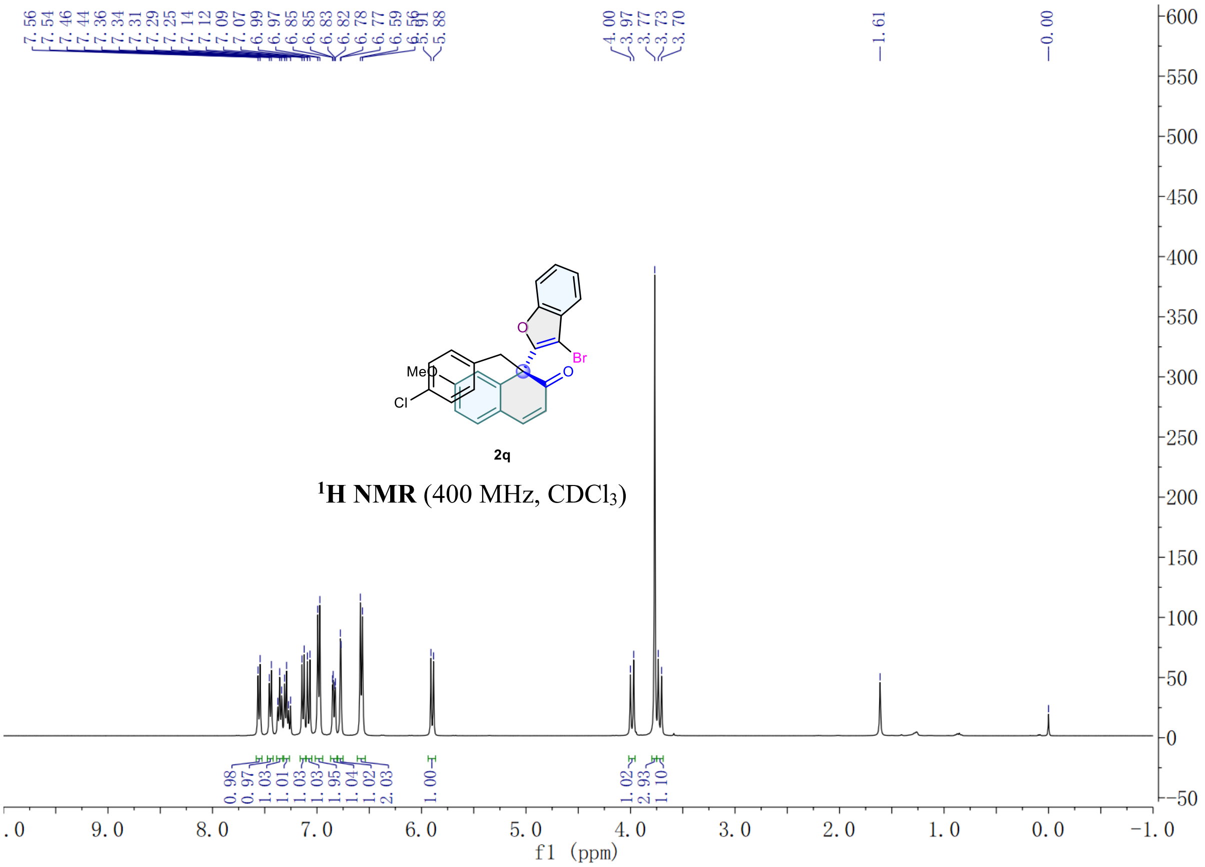
2q
¹H NMR (400 MHz, CDCl₃)

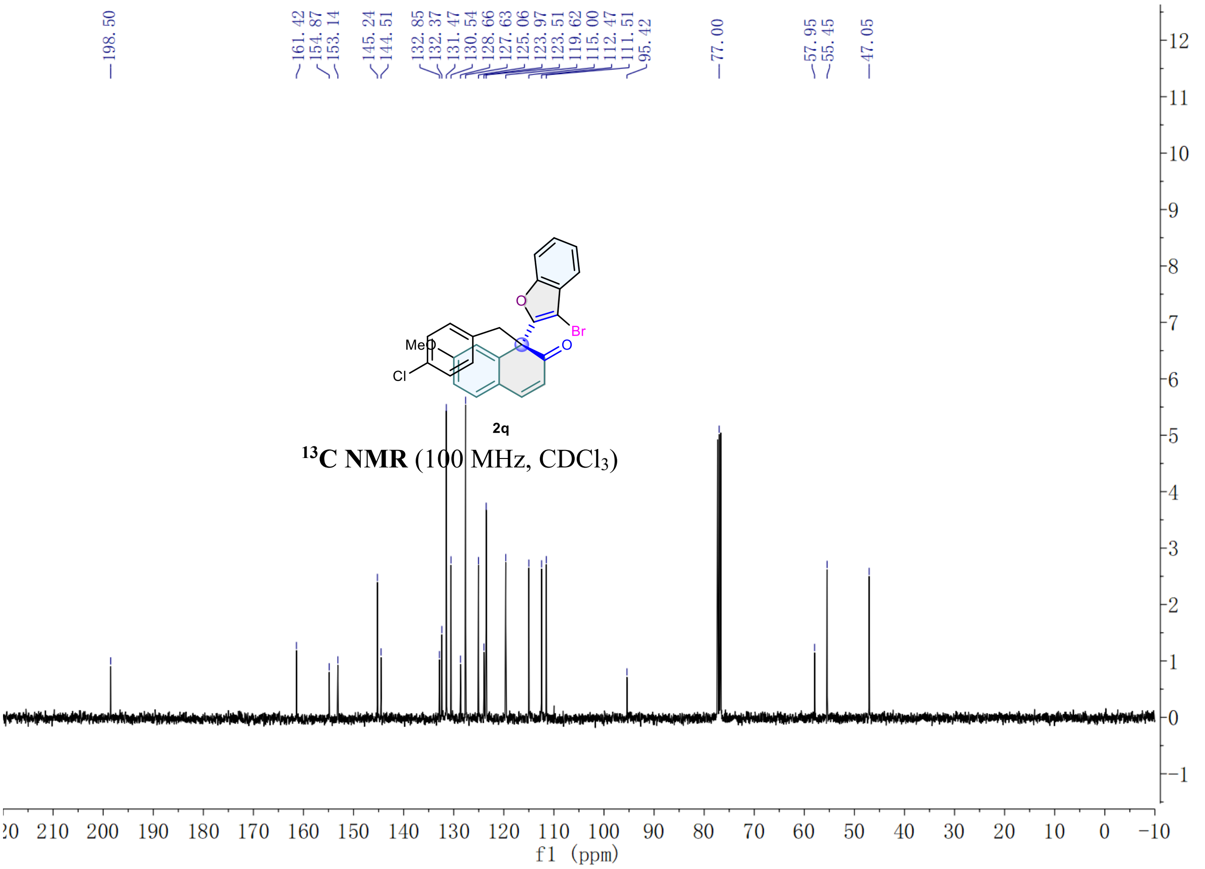
2q
¹³C NMR (100 MHz, CDCl₃)

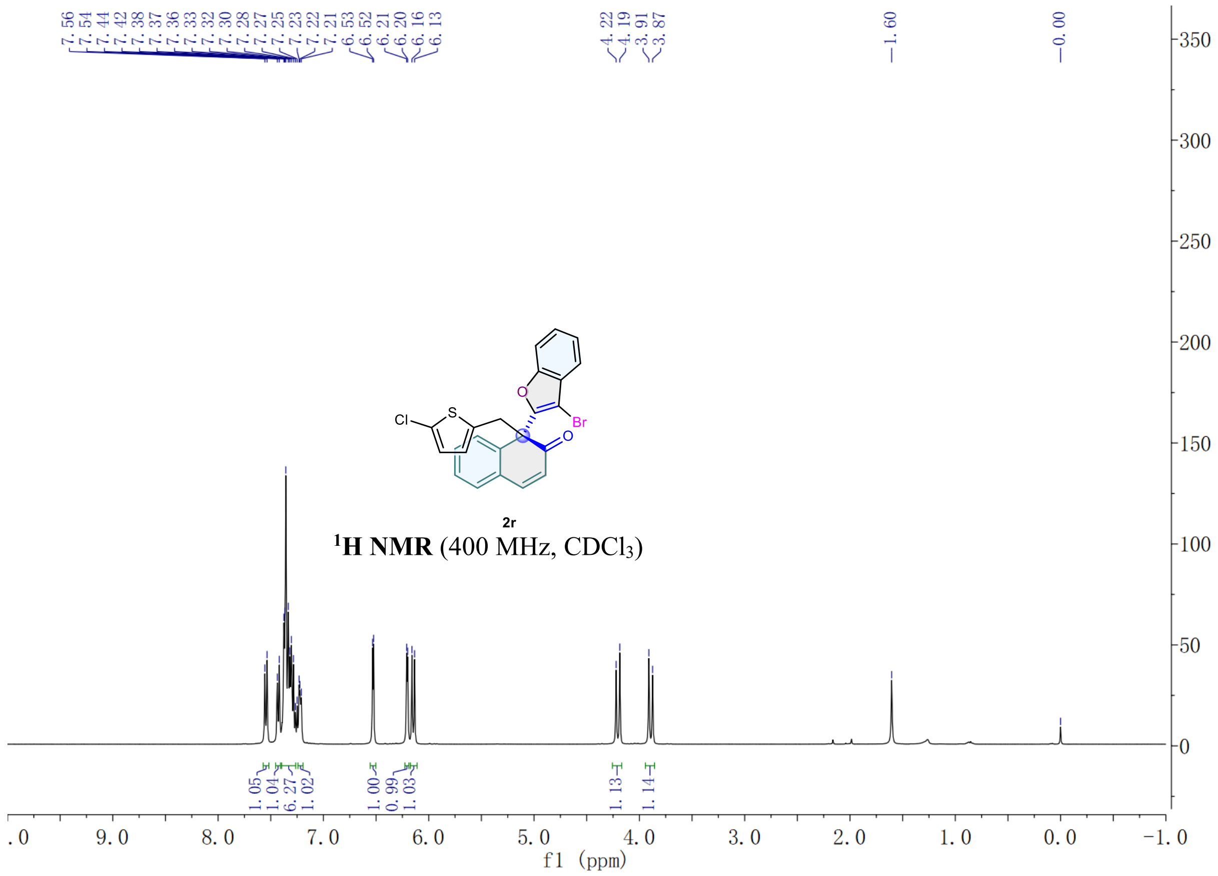

2r
^{1}H NMR (400 MHz, CDCl$_3$)

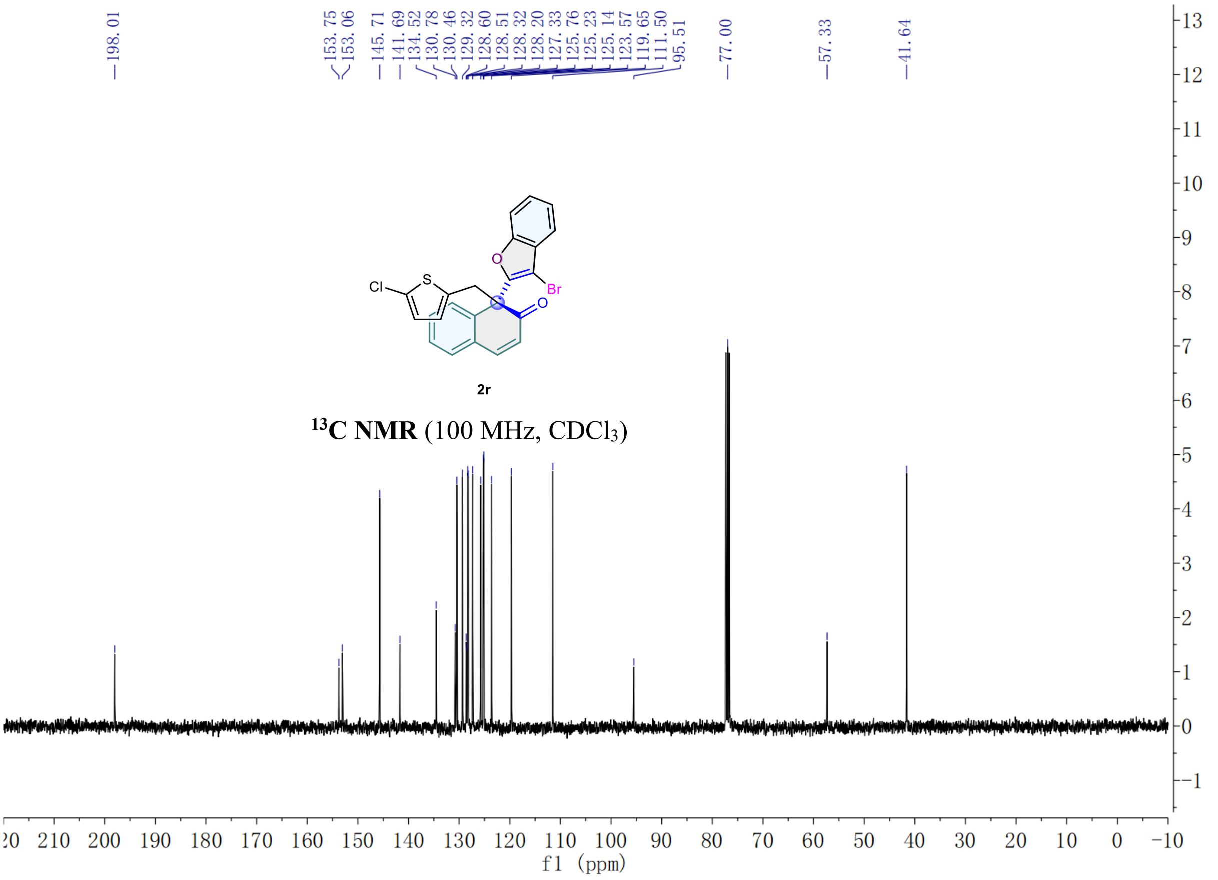

2r
^{13}C NMR (100 MHz, CDCl$_3$)

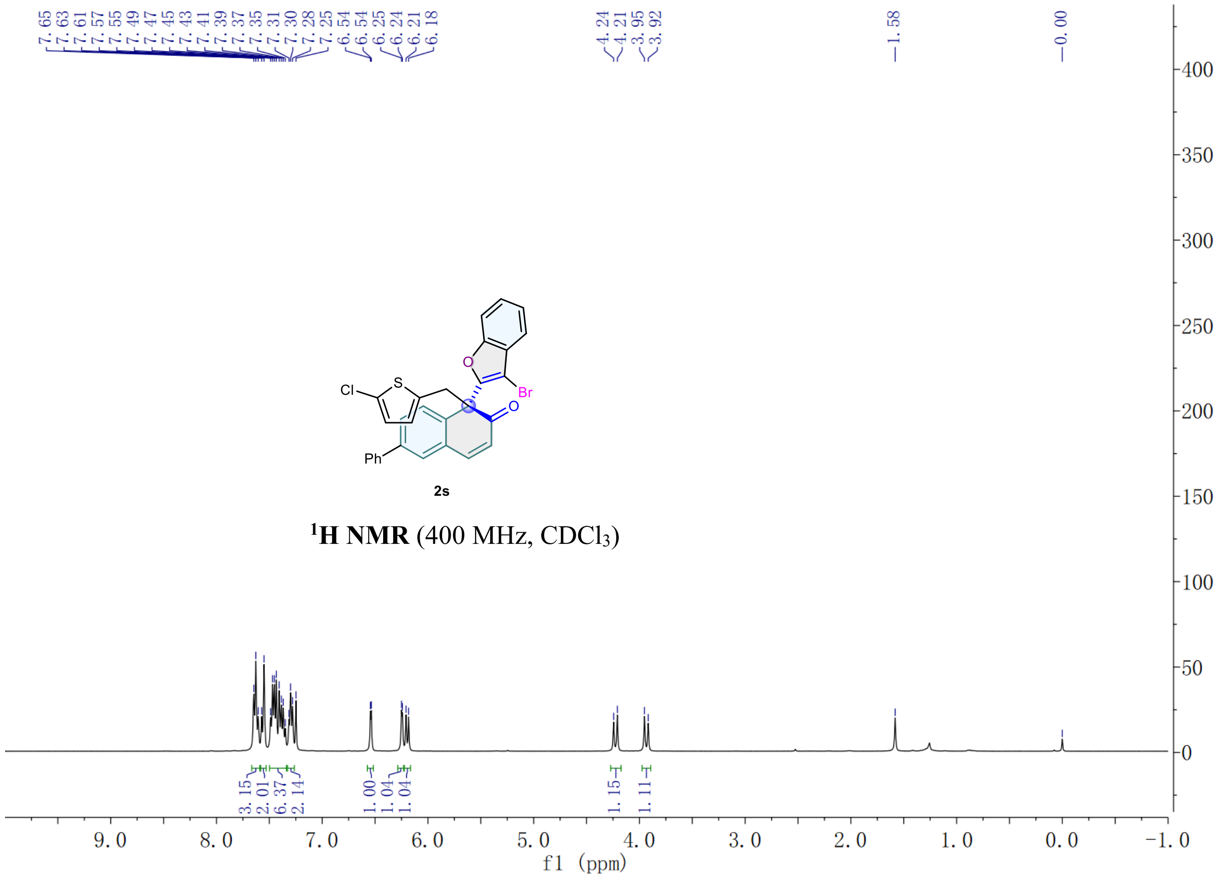
7.65 7.63 7.61 7.57 7.55 7.49 7.47 7.45 7.43 7.41 7.39 7.37 7.35 7.31 7.30 7.28 7.25 6.54 6.54 6.25 6.24 6.21 6.18
4.24 4.21 3.95 3.92
1.58
0.00
Cl
S
O
Br
O
Ph
2s
^{1}H NMR (400 MHz, CDCl$_3$)
3.15 2.01 6.37 2.14
1.00 1.04 1.04
1.15 1.11
f1 (ppm)

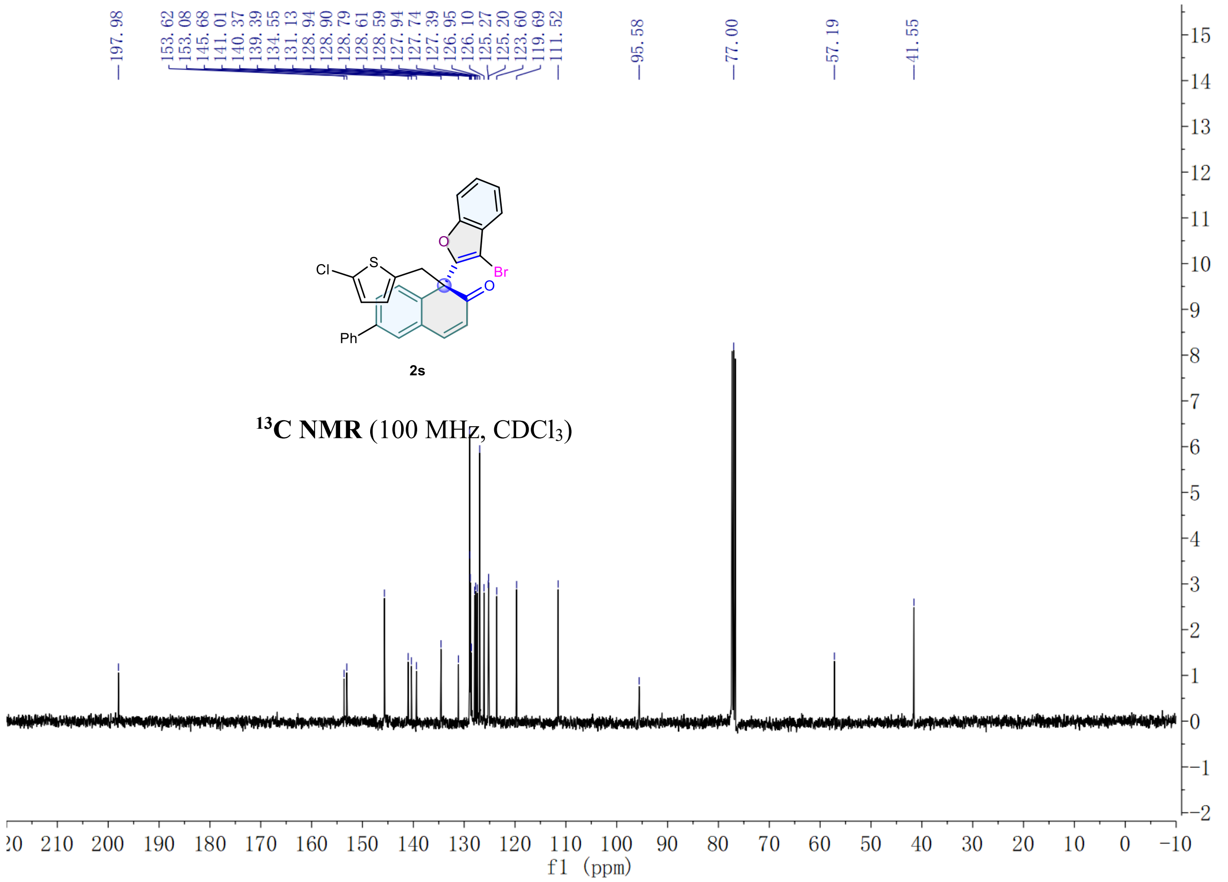
197.98
153.62 153.08 145.68 141.01 140.37 139.39 134.55 131.13 128.94 128.90 128.79 128.61 128.59 127.94 127.74 127.39 126.95 126.10 125.27 125.20 123.60 119.69 111.52
95.58
77.00
57.19
41.55
Cl
S
O
Br
O
Ph
2s
^{13}C NMR (100 MHz, CDCl$_3$)
f1 (ppm)

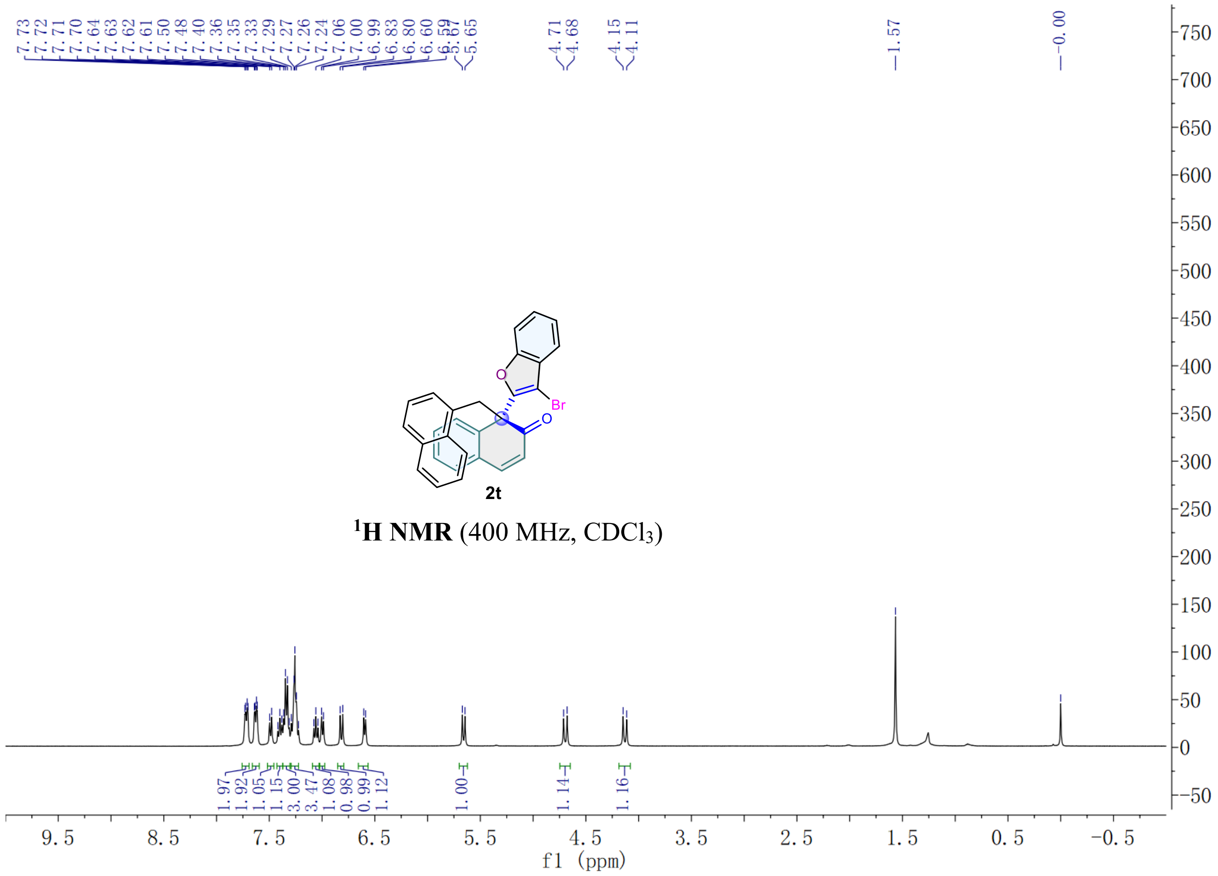
2t
¹H NMR (400 MHz, CDCl₃)
f1 (ppm)

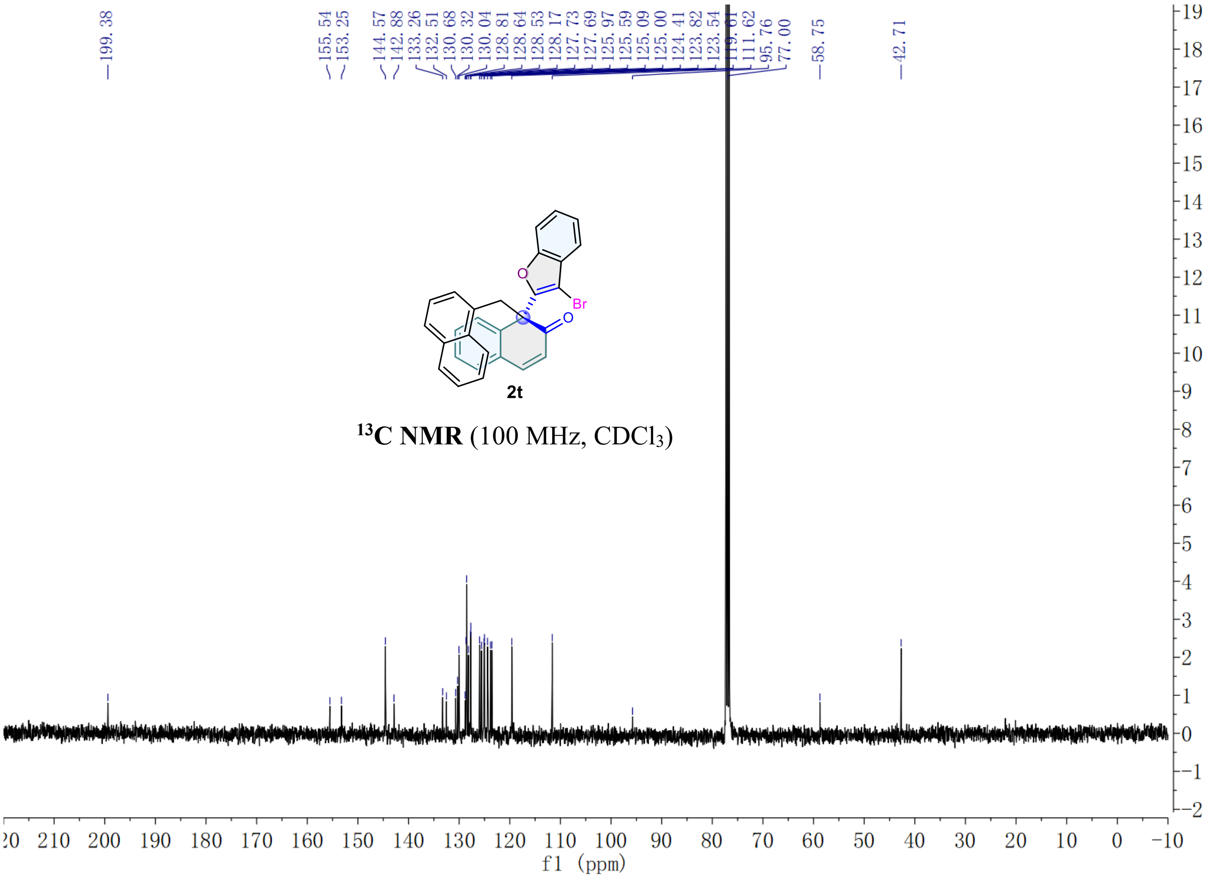
2t
¹³C NMR (100 MHz, CDCl₃)
f1 (ppm)

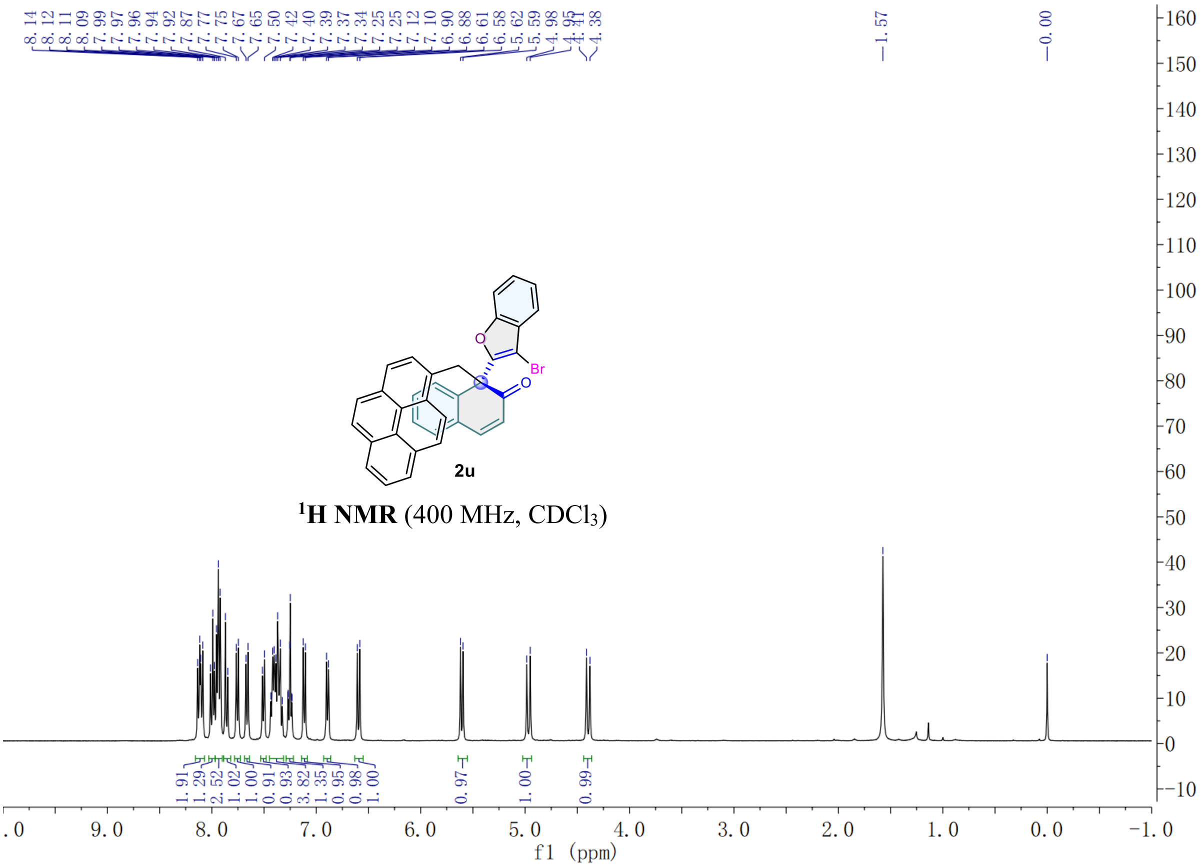
8.14 8.12 8.11 8.09 7.99 7.97 7.96 7.94 7.92 7.87 7.77 7.75 7.67 7.65 7.50 7.42 7.40 7.39 7.37 7.34 7.25 7.25 7.12 7.10 6.90 6.88 6.61 6.58 5.62 5.59 4.98 4.95 4.41 4.38
1.57
0.00
O
Br
O
2u
^{1}H NMR (400 MHz, $CDCl_3$)
1.91 1.29 2.52 1.02 1.00 0.91 0.93 3.82 1.35 0.95 0.98 1.00
0.97
1.00
0.99
f1 (ppm)

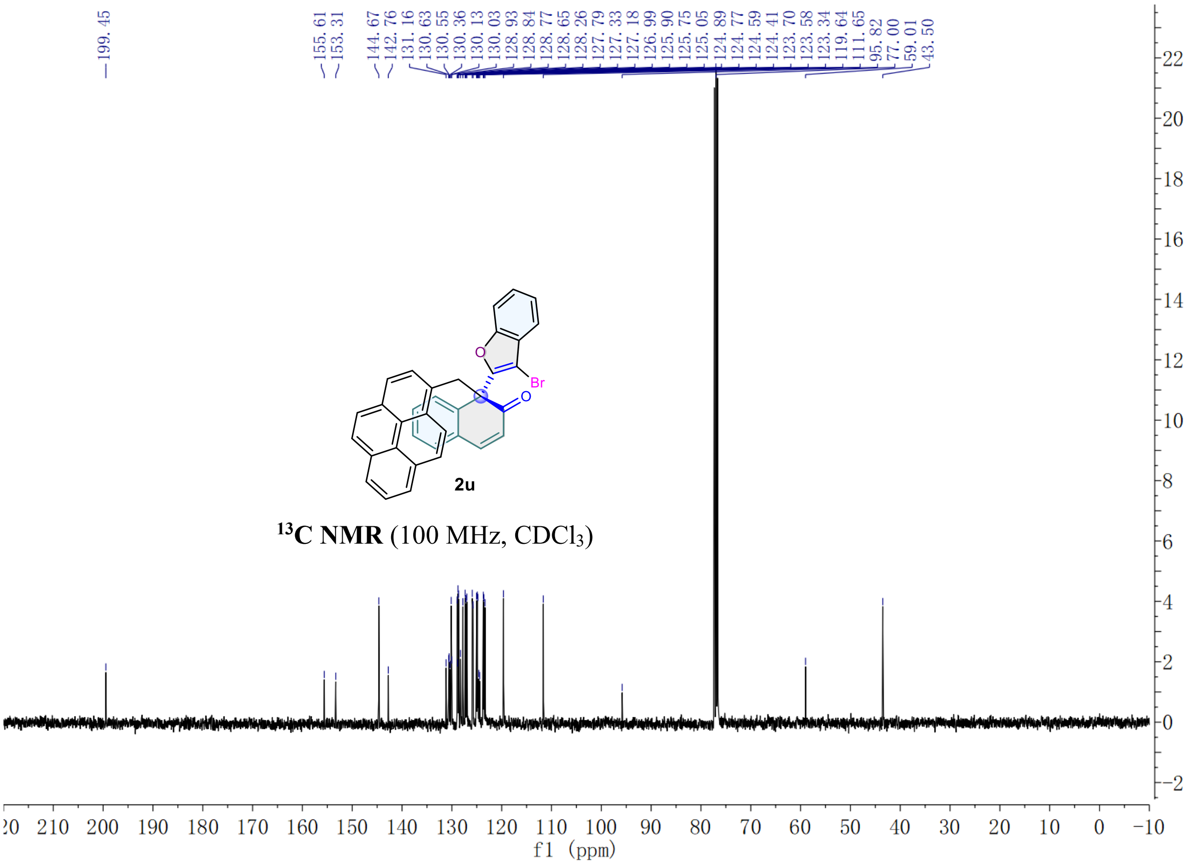
199.45
155.61 153.31
144.67 142.76 131.16 130.63 130.55 130.36 130.13 130.03 128.93 128.84 128.77 128.65 128.26 127.79 127.33 127.18 126.99 125.90 125.75 125.05 124.89 124.77 124.59 124.41 123.70 123.58 123.34 119.64 111.65 95.82 77.00 59.01 43.50
O
Br
O
2u
^{13}C NMR (100 MHz, $CDCl_3$)
f1 (ppm)

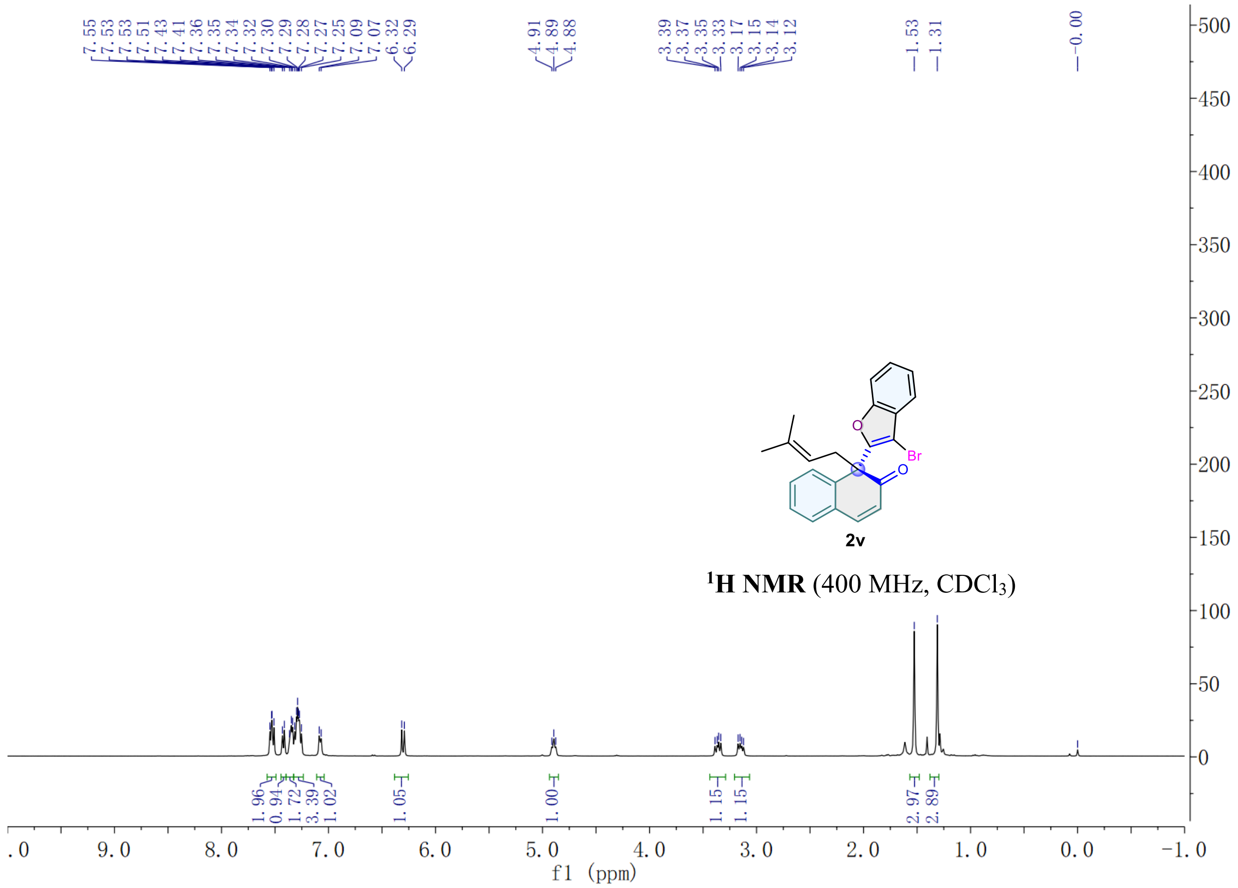

¹H NMR (400 MHz, $CDCl_3$)

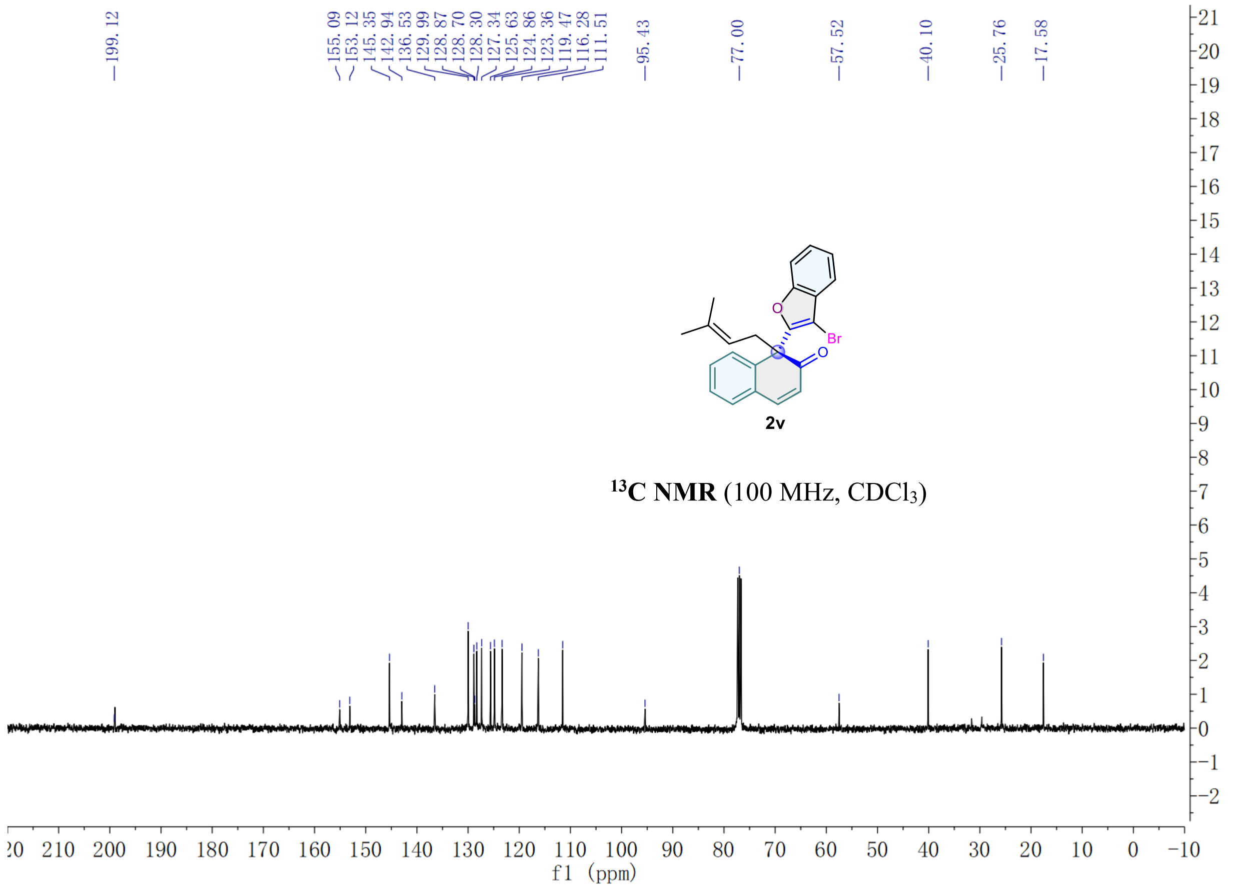

¹³C NMR (100 MHz, $CDCl_3$)

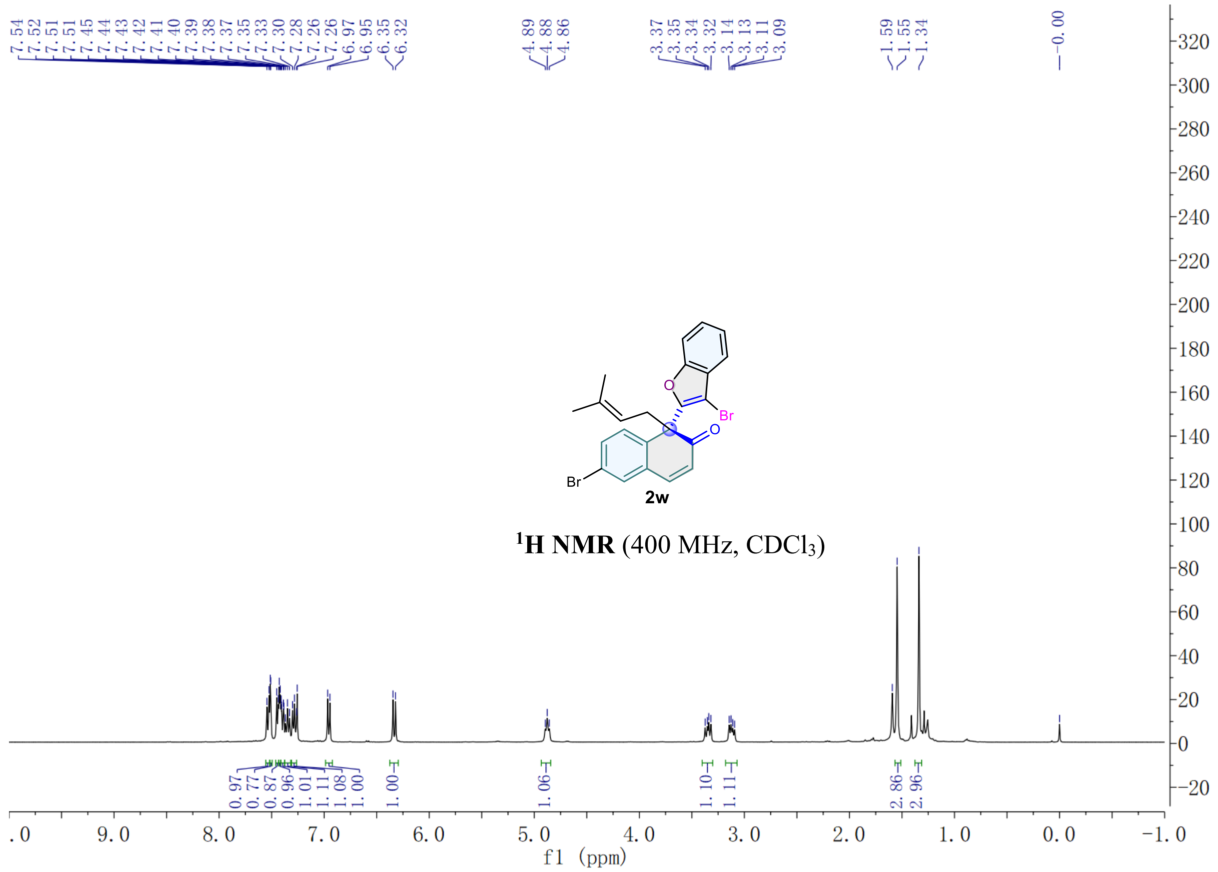
2w
¹H NMR (400 MHz, CDCl3)
f1 (ppm)

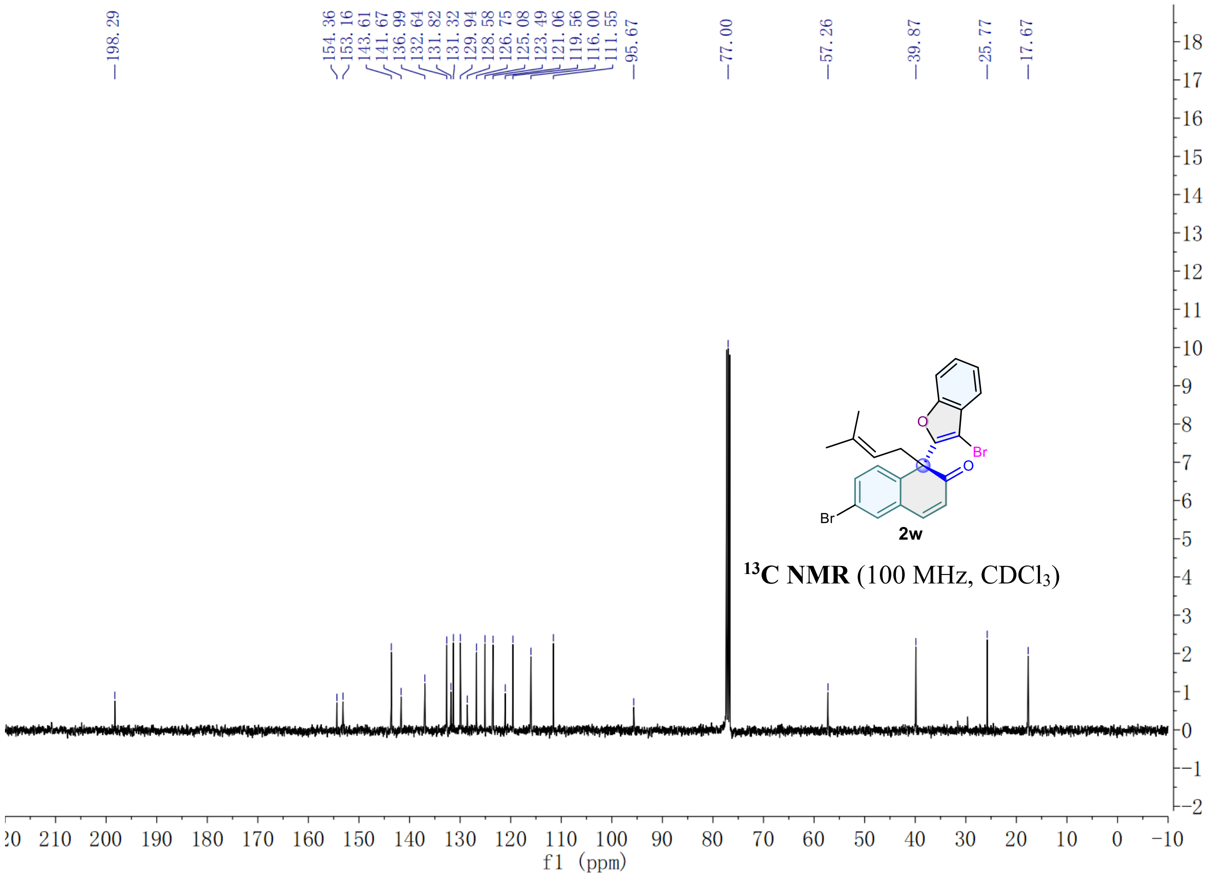
2w
¹³C NMR (100 MHz, CDCl3)
f1 (ppm)

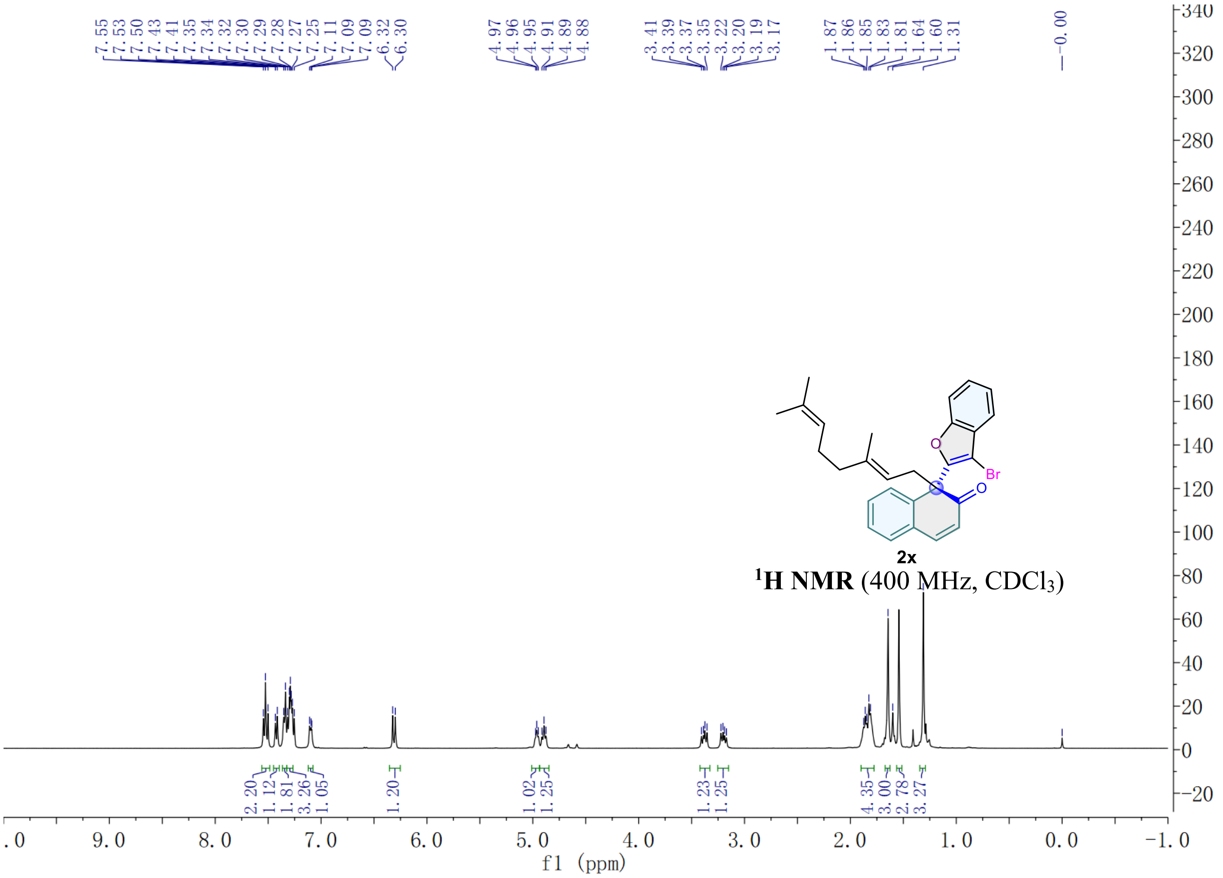
7.55
7.53
7.50
7.43
7.41
7.35
7.34
7.32
7.30
7.29
7.28
7.27
7.25
7.11
7.09
6.32
6.30
4.97
4.96
4.95
4.91
4.89
4.88
3.41
3.39
3.37
3.35
3.22
3.20
3.19
3.17
1.87
1.86
1.85
1.83
1.81
1.64
1.60
1.31
0.00
O
Br
O
2x
¹H NMR (400 MHz, CDCl₃)
2.20
1.12
1.81
3.26
1.05
1.20
1.02
1.25
1.23
1.25
4.35
3.00
2.78
3.27
f1 (ppm)

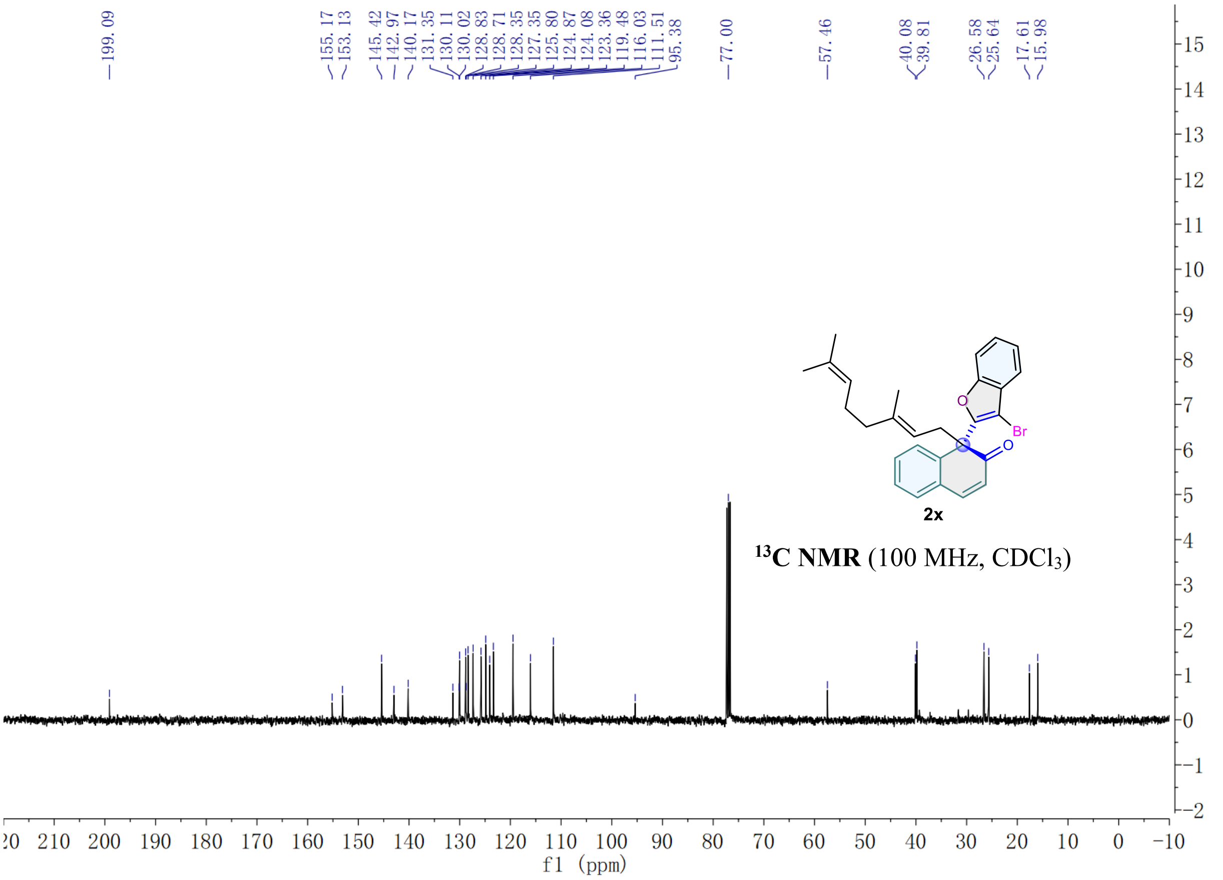
199.09
155.17
153.13
145.42
142.97
140.17
131.35
130.11
130.02
128.83
128.71
128.35
127.35
125.80
124.87
124.08
123.36
119.48
116.03
111.51
95.38
77.00
57.46
40.08
39.81
26.58
25.64
17.61
15.98
O
Br
O
2x
¹³C NMR (100 MHz, CDCl₃)
f1 (ppm)

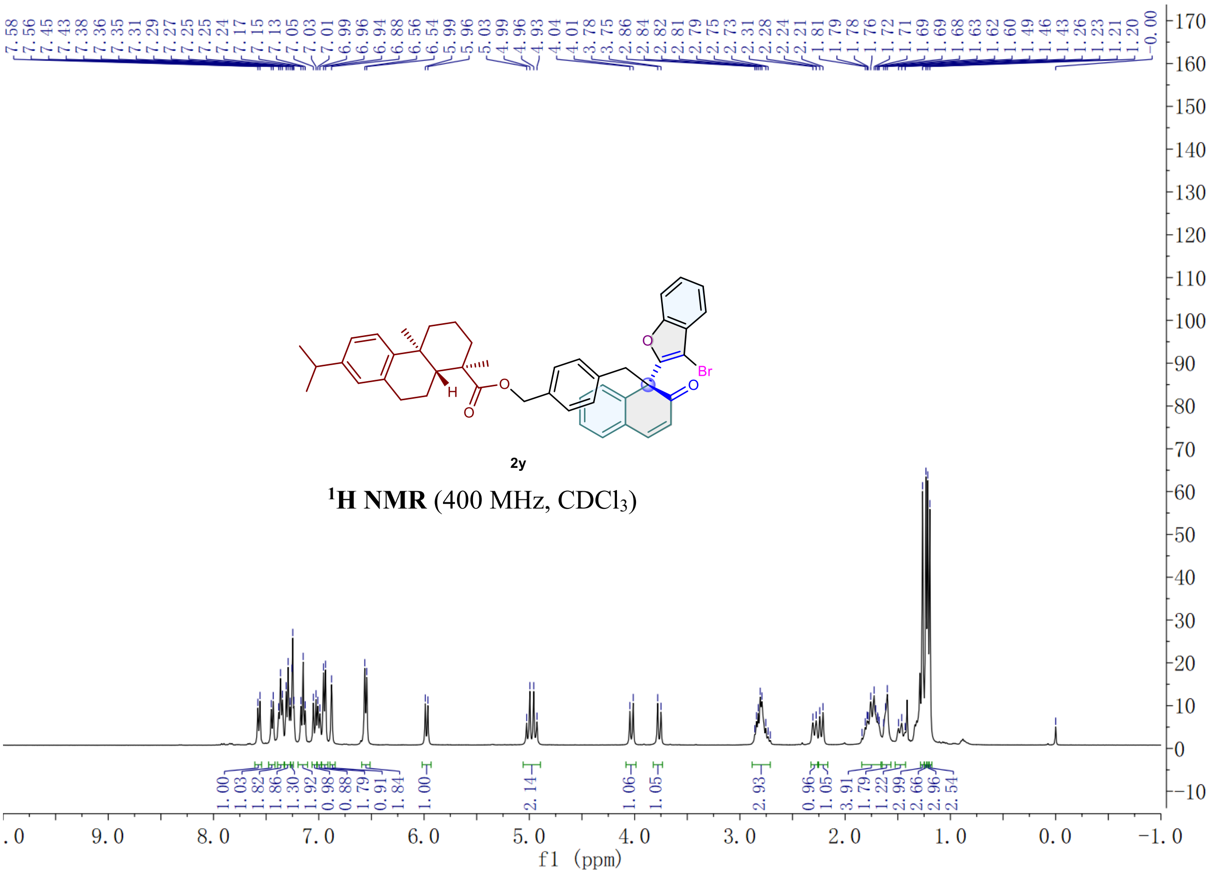

7.58 7.56 7.45 7.43 7.38 7.36 7.35 7.31 7.29 7.27 7.25 7.25 7.24 7.17 7.15 7.13 7.05 7.03 7.01 6.99 6.96 6.94 6.88 6.56 6.54 5.99 5.96 5.03 4.99 4.96 4.93 4.04 4.01 3.78 3.75 2.86 2.84 2.82 2.81 2.79 2.75 2.73 2.31 2.28 2.24 2.21 1.81 1.79 1.78 1.76 1.72 1.71 1.69 1.69 1.68 1.63 1.62 1.60 1.49 1.46 1.43 1.26 1.23 1.21 1.20 -0.00
O
Br
O
H
O
O
2y
1H NMR (400 MHz, CDCl3)
1.00 1.03 1.82 1.86 1.30 1.92 0.98 0.88 1.79 0.91 1.84 1.00 2.14 1.06 1.05 2.93 0.96 1.05 3.91 1.79 1.22 2.99 2.66 2.96 2.54
f1 (ppm)

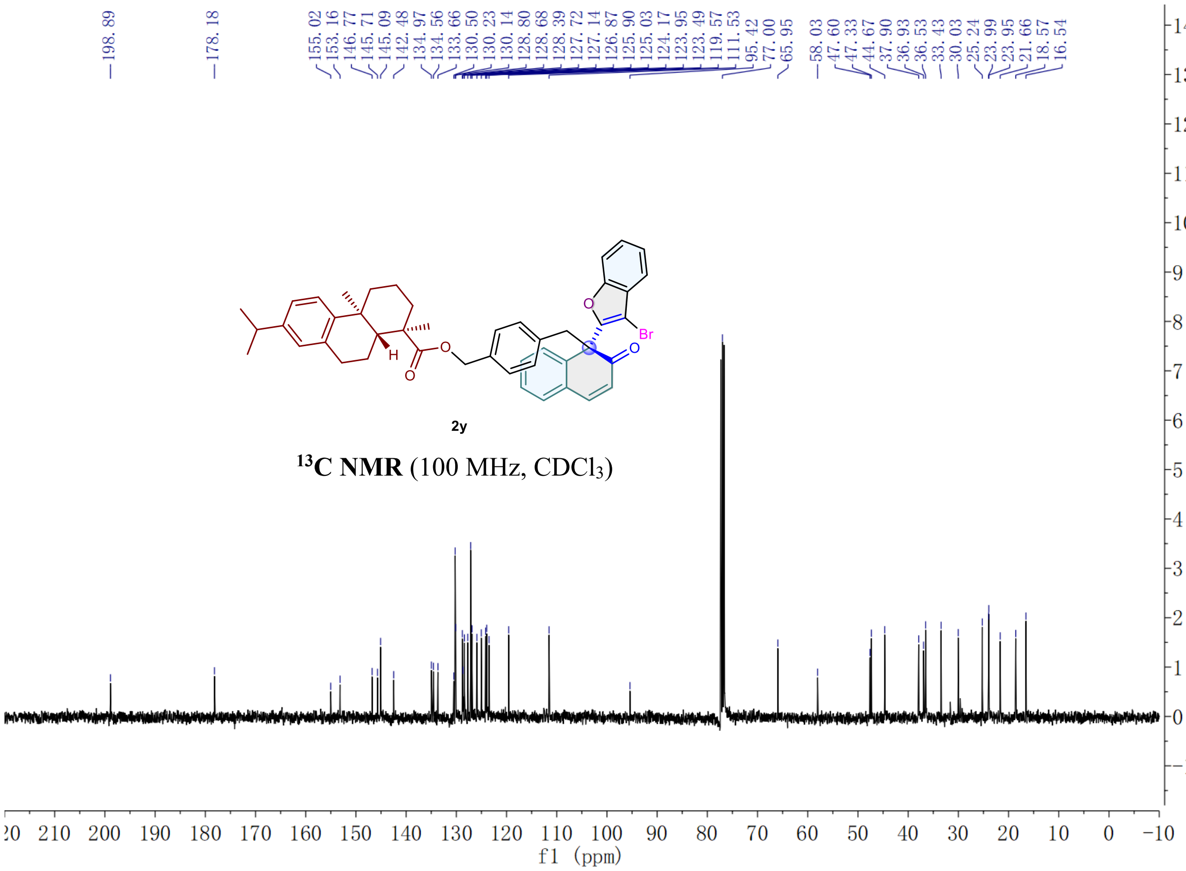

198.89 178.18 155.02 153.16 146.77 145.71 145.09 142.48 134.97 134.56 133.66 130.50 130.23 130.14 128.80 128.68 128.39 127.72 127.14 126.87 125.90 125.03 124.17 123.95 123.49 119.57 111.53 95.42 77.00 65.95 58.03 47.60 47.33 44.67 37.90 36.93 36.53 33.43 30.03 25.24 23.99 23.95 21.66 18.57 16.54
O
Br
O
H
O
O
2y
13C NMR (100 MHz, CDCl3)
f1 (ppm)

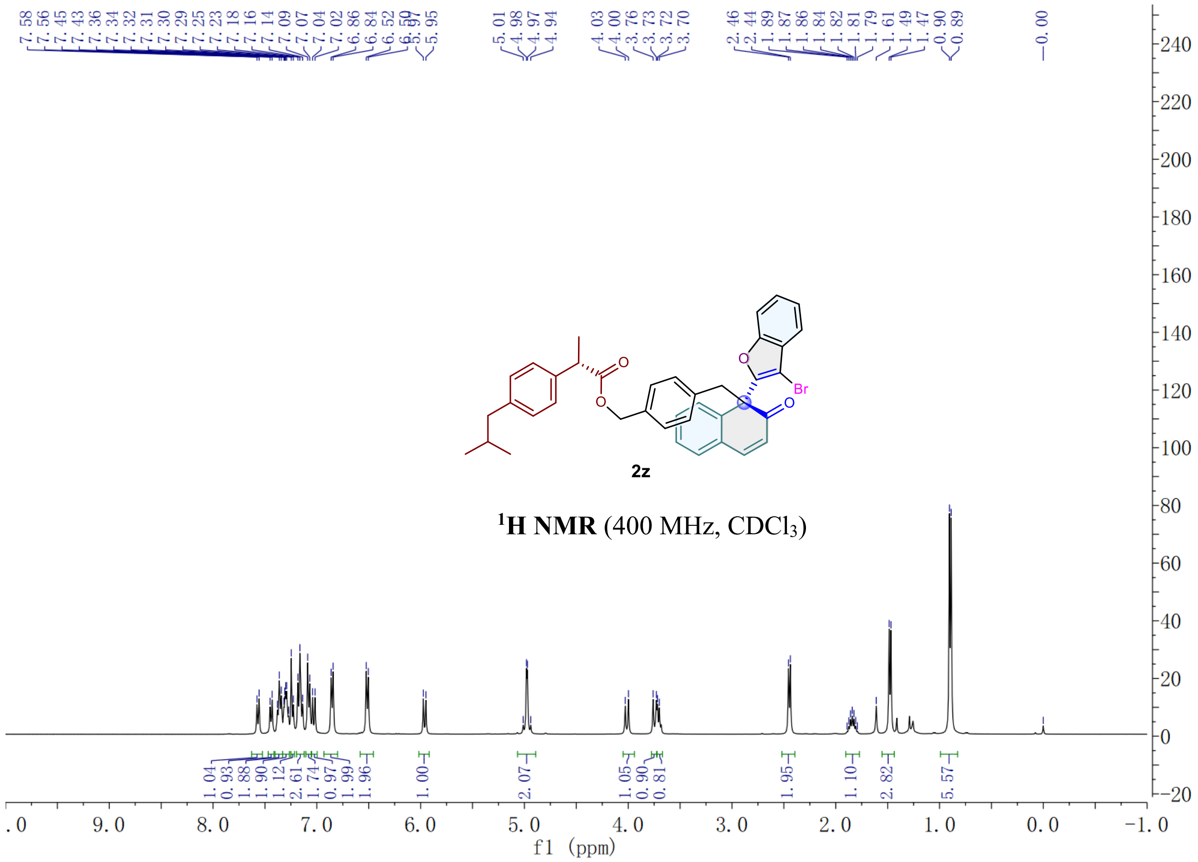
2z
¹H NMR (400 MHz, CDCl₃)
f1 (ppm)

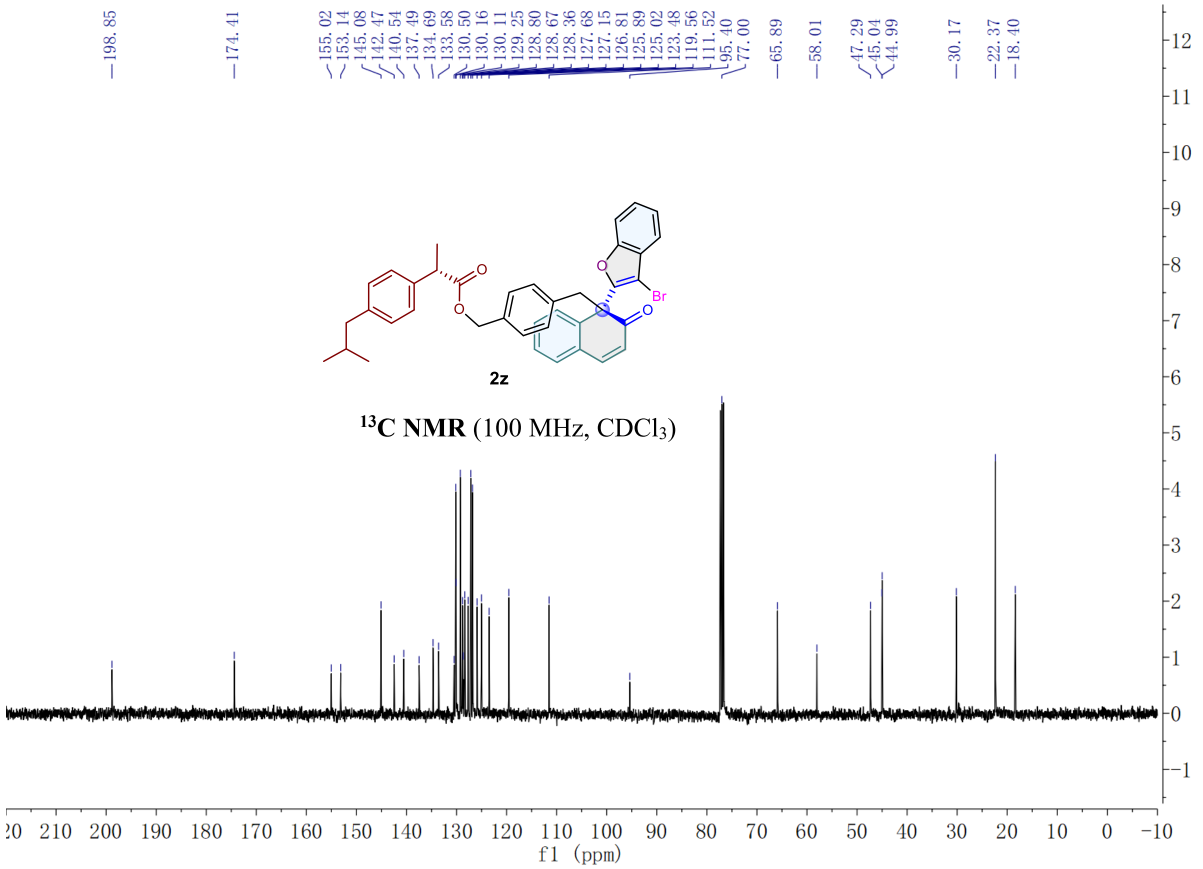
2z
¹³C NMR (100 MHz, CDCl₃)
f1 (ppm)

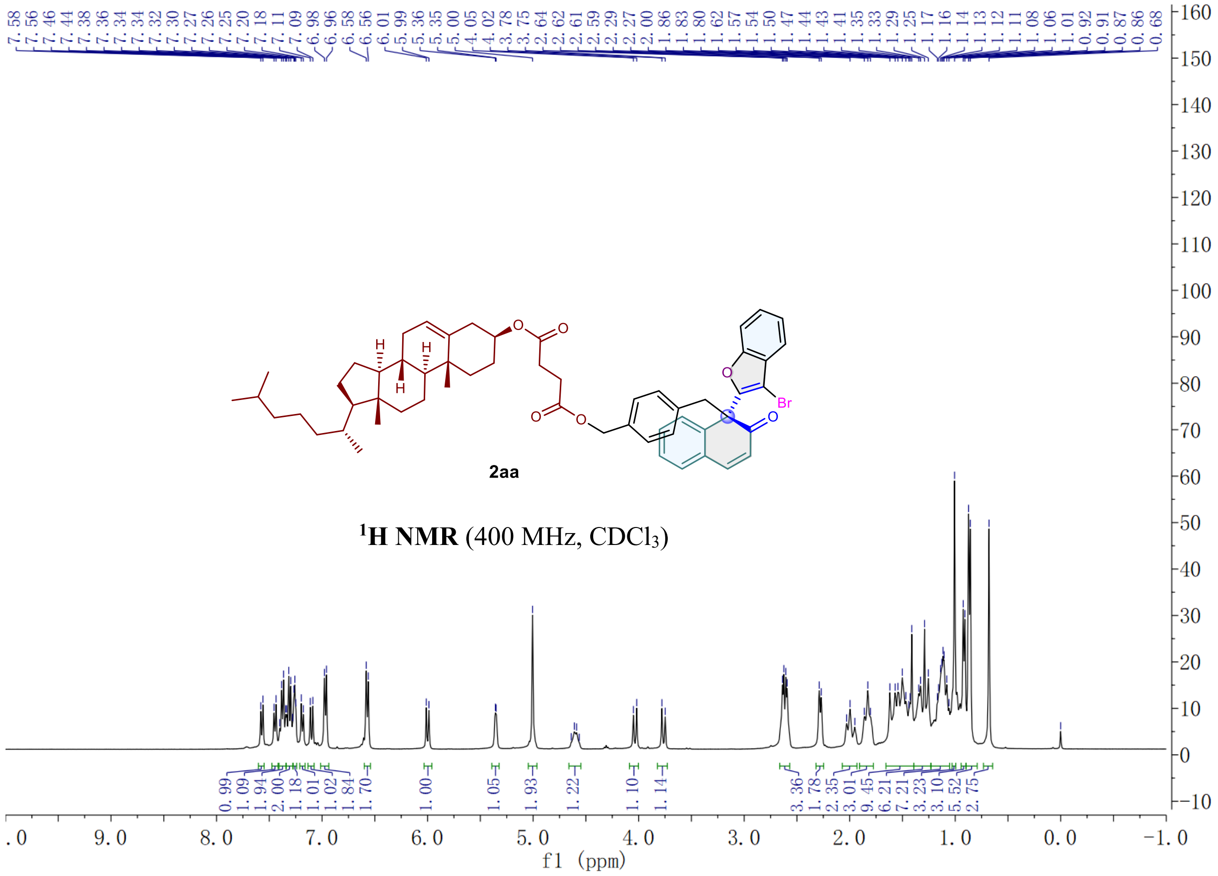

^{1}H NMR (400 MHz, $CDCl_3$)

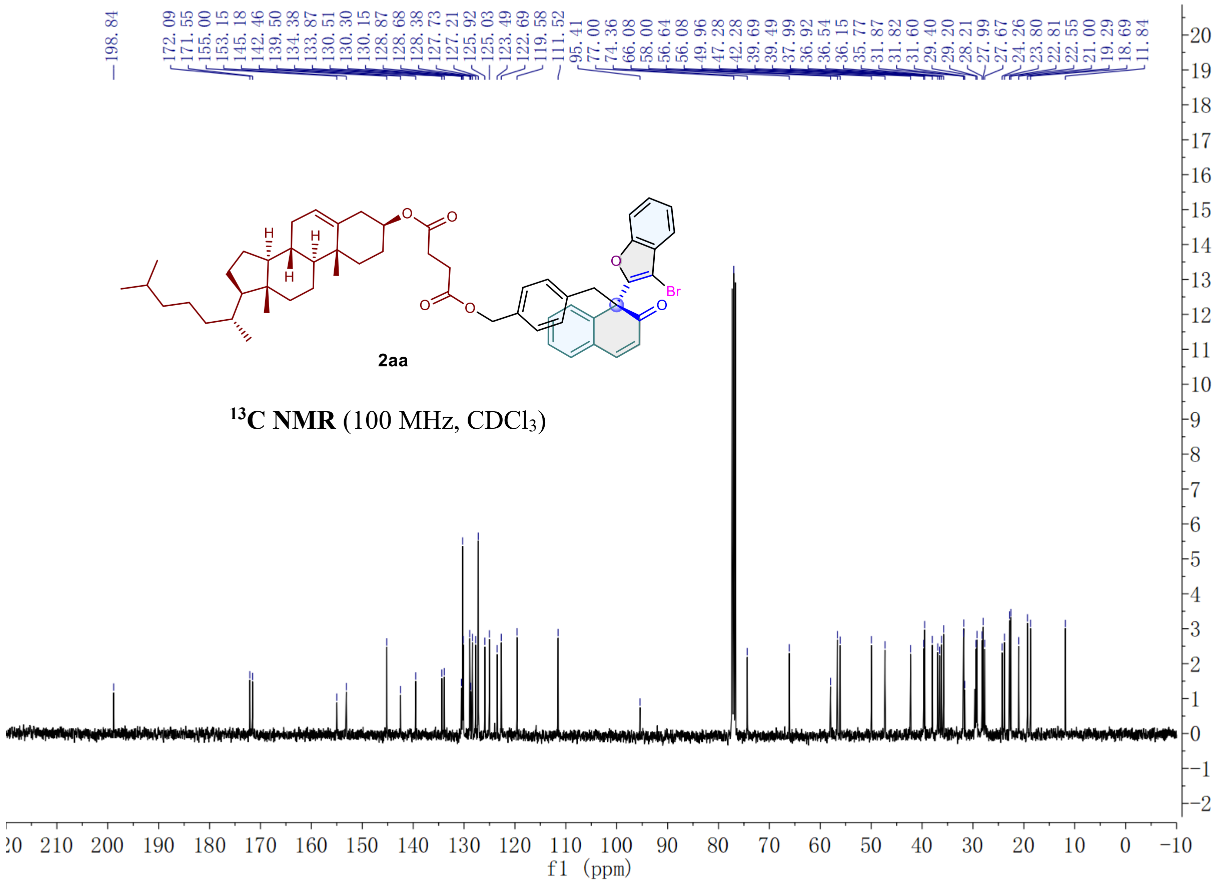

^{13}C NMR (100 MHz, $CDCl_3$)

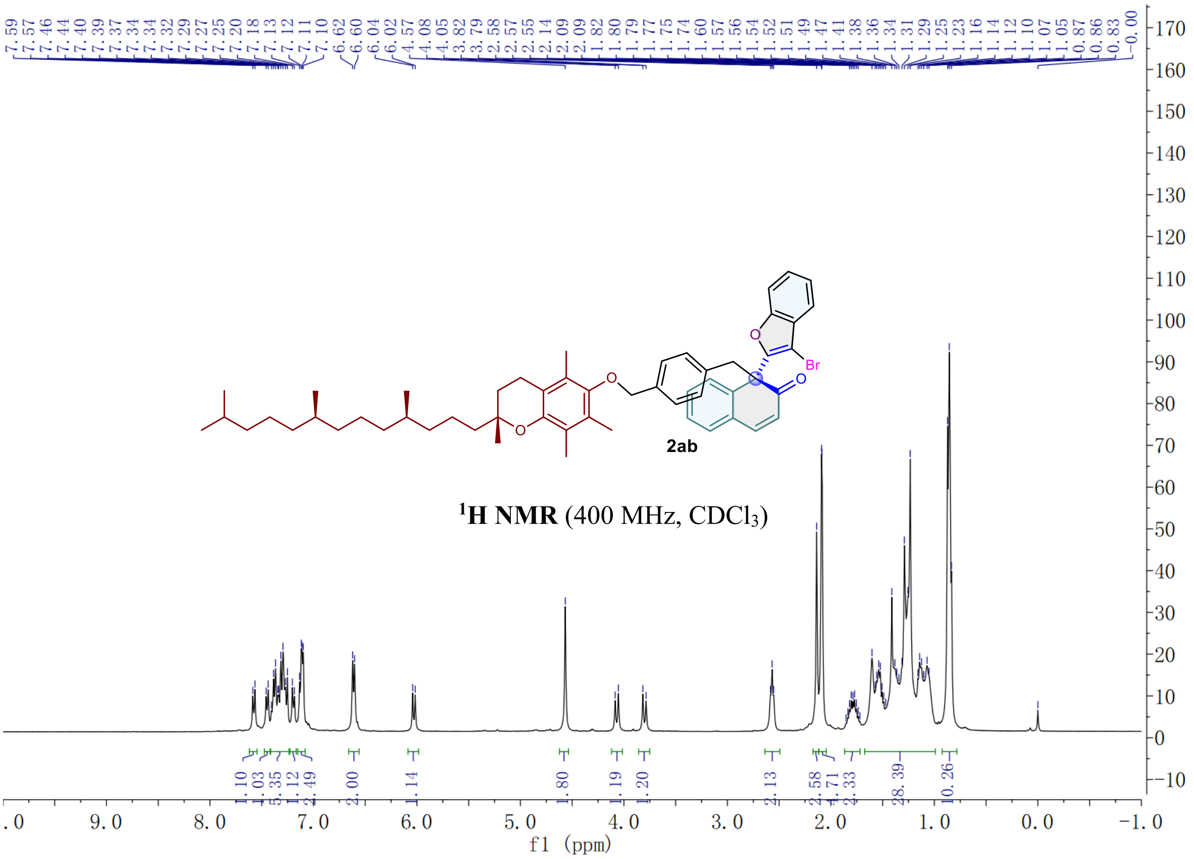
2ab
¹H NMR (400 MHz, CDCl₃)
f1 (ppm)

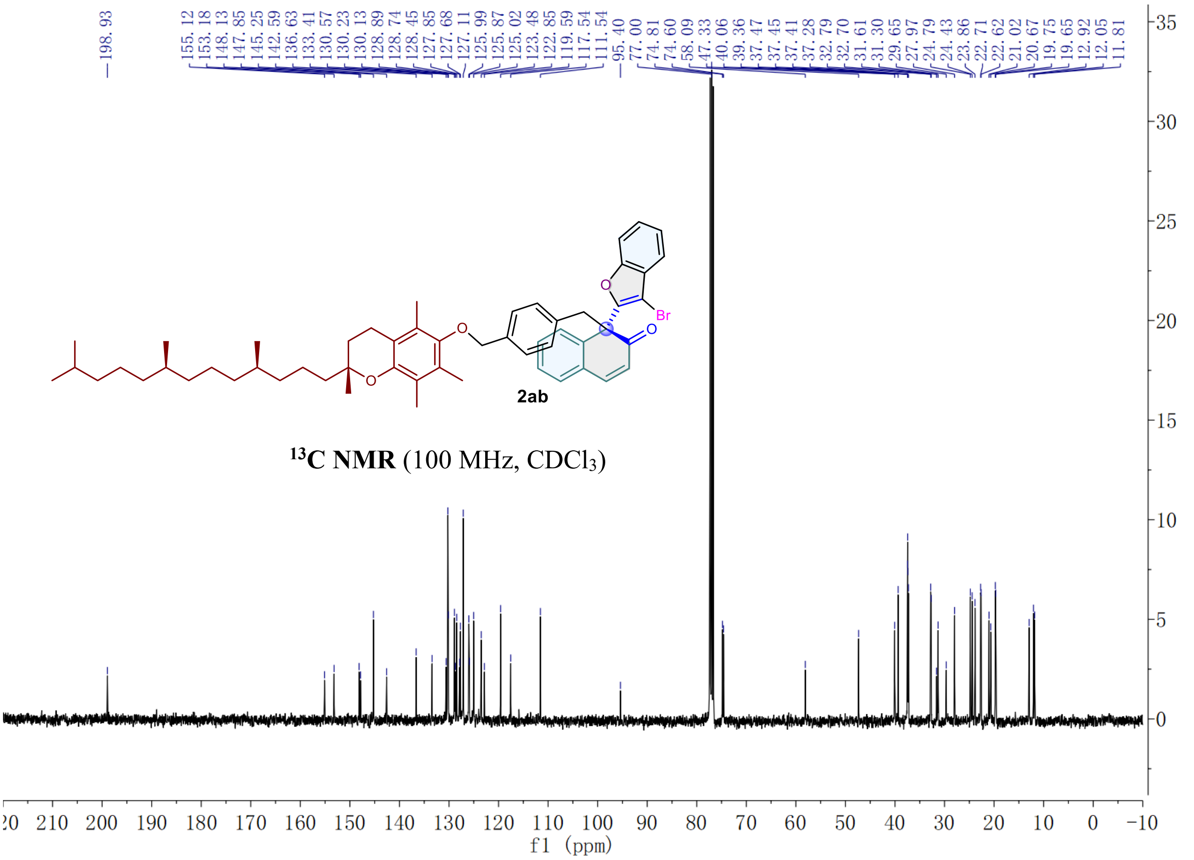
2ab
¹³C NMR (100 MHz, CDCl₃)
f1 (ppm)

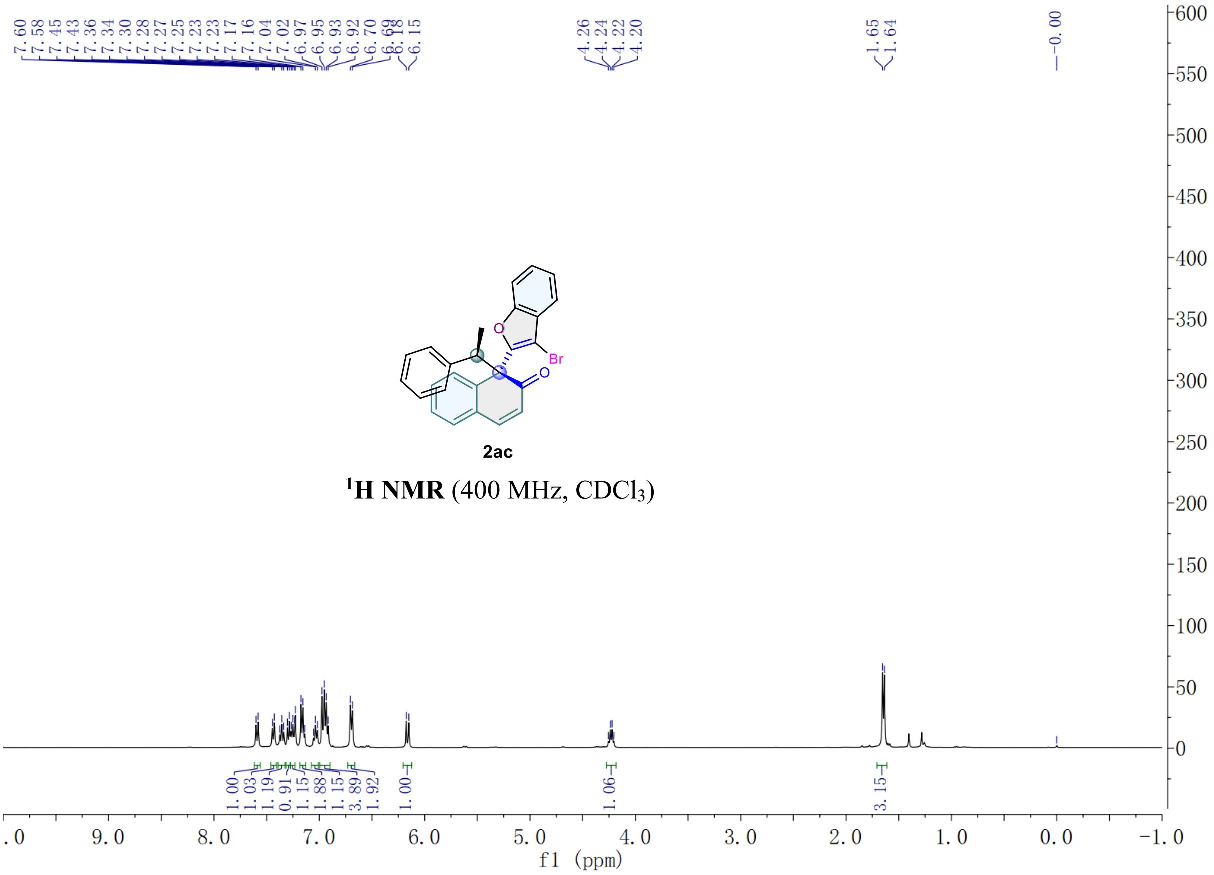

2ac

[1]**H NMR** (400 MHz, $CDCl_3$)

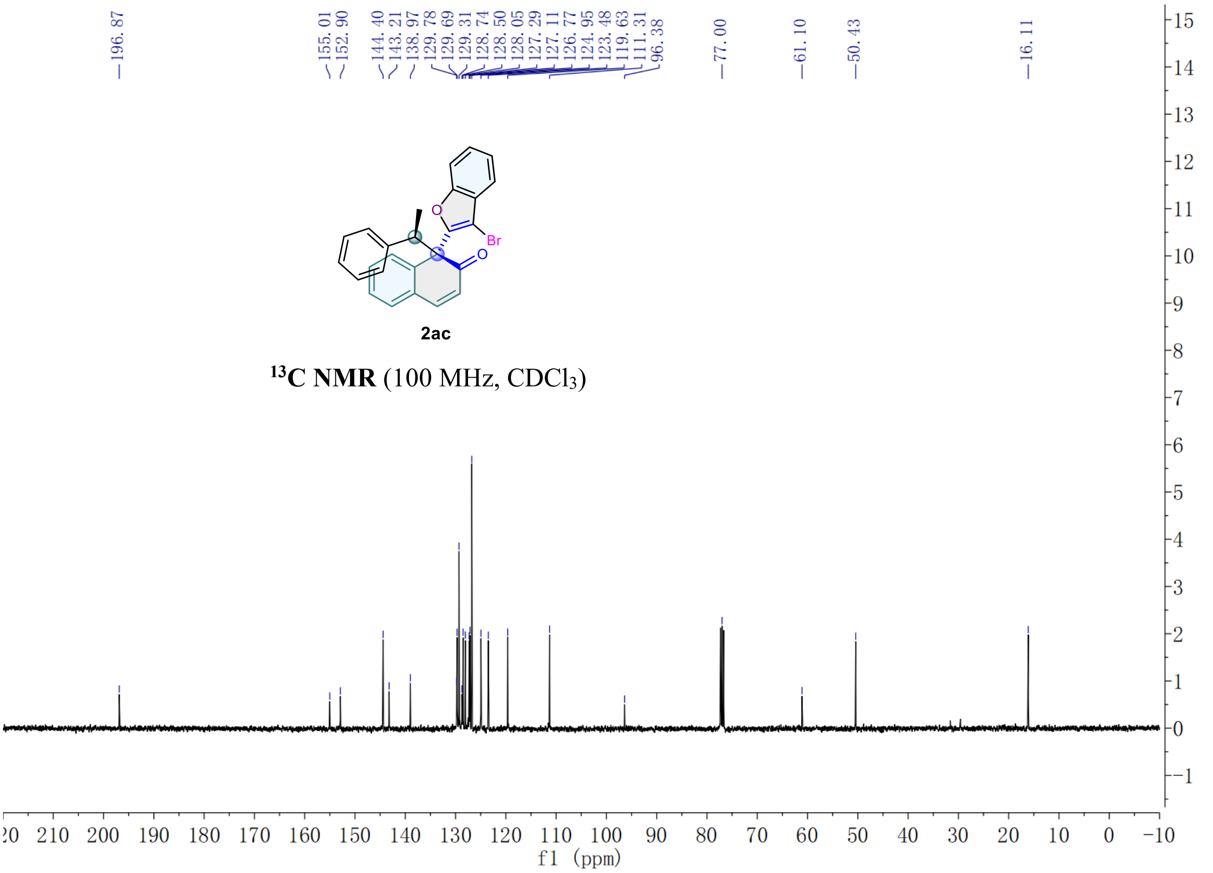

2ac

[13]**C NMR** (100 MHz, $CDCl_3$)

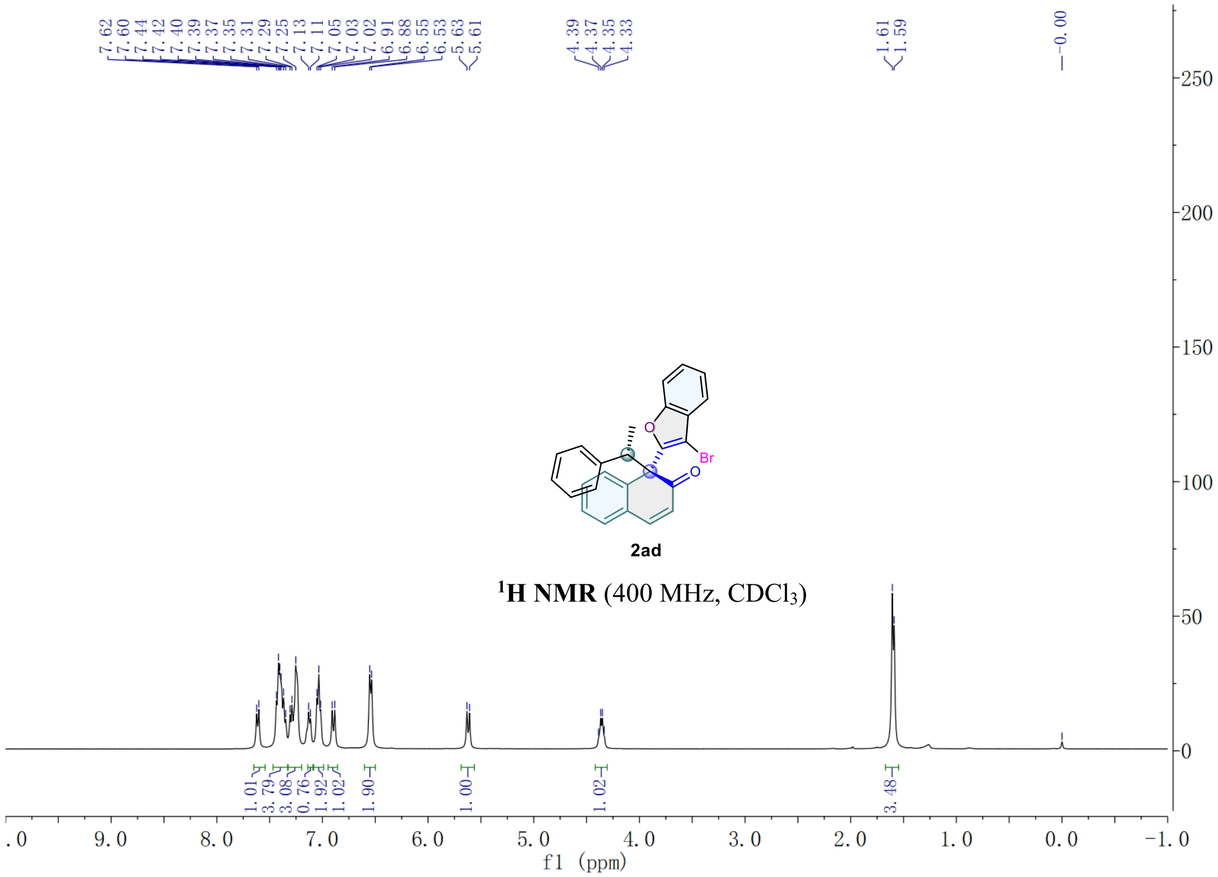

2ad

^{1}H NMR (400 MHz, $CDCl_3$)

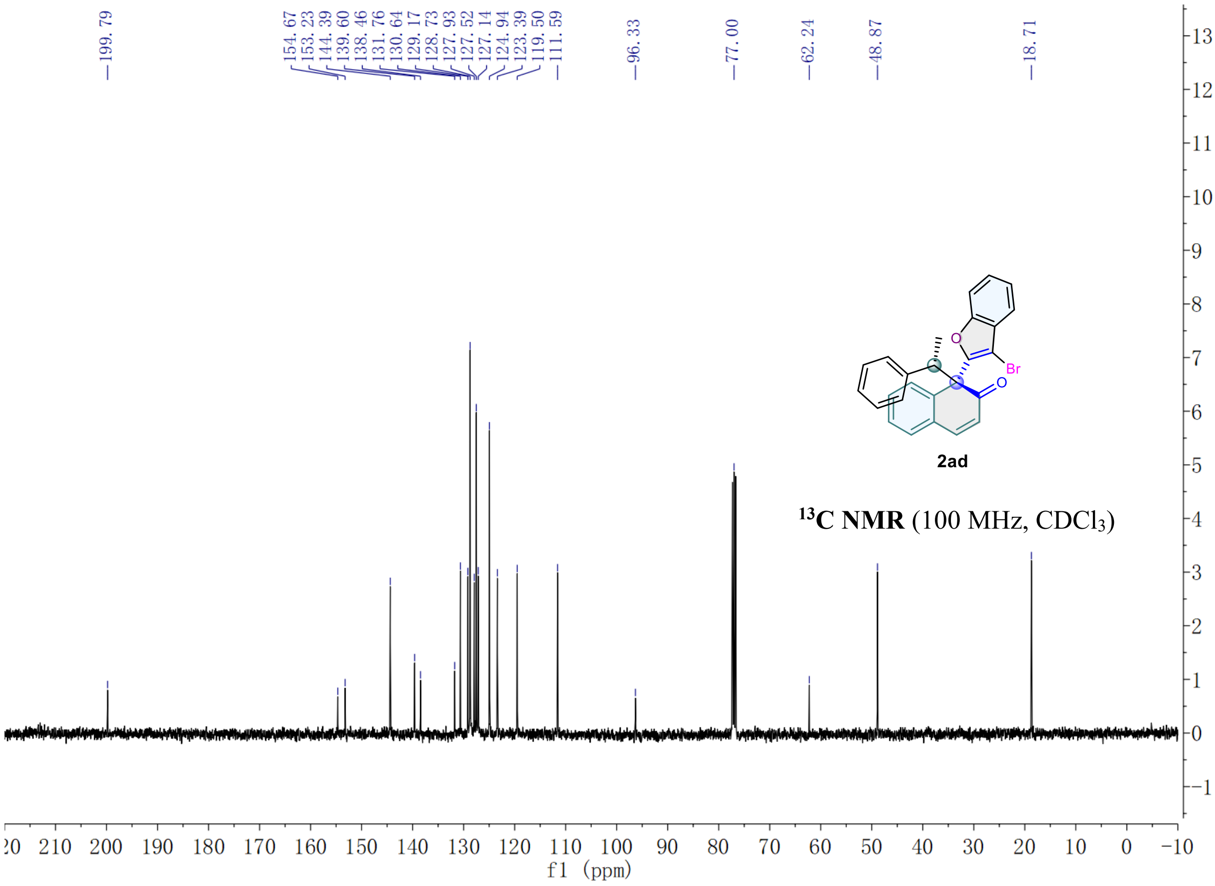

2ad

^{13}C NMR (100 MHz, $CDCl_3$)

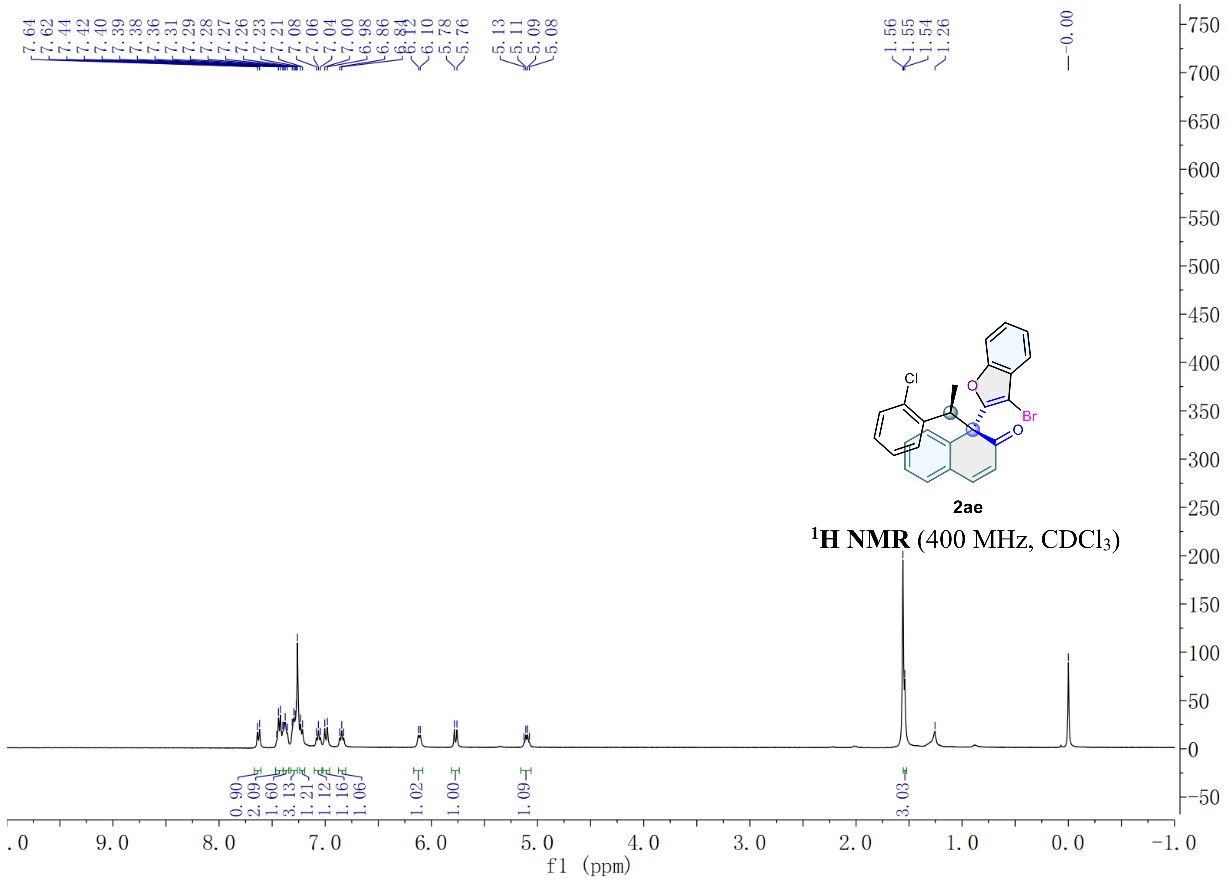
7.64 7.62 7.44 7.42 7.40 7.39 7.38 7.36 7.31 7.29 7.28 7.27 7.26 7.23 7.21 7.08 7.06 7.04 7.00 6.98 6.86 6.84 6.12 6.10 5.78 5.76 5.13 5.11 5.09 5.08
1.56 1.55 1.54 1.26
0.00
0.90 2.09 1.60 3.13 1.21 1.12 1.16 1.06 1.02 1.00 1.09 3.03
f1 (ppm)
2ae
^{1}H NMR (400 MHz, CDCl$_3$)

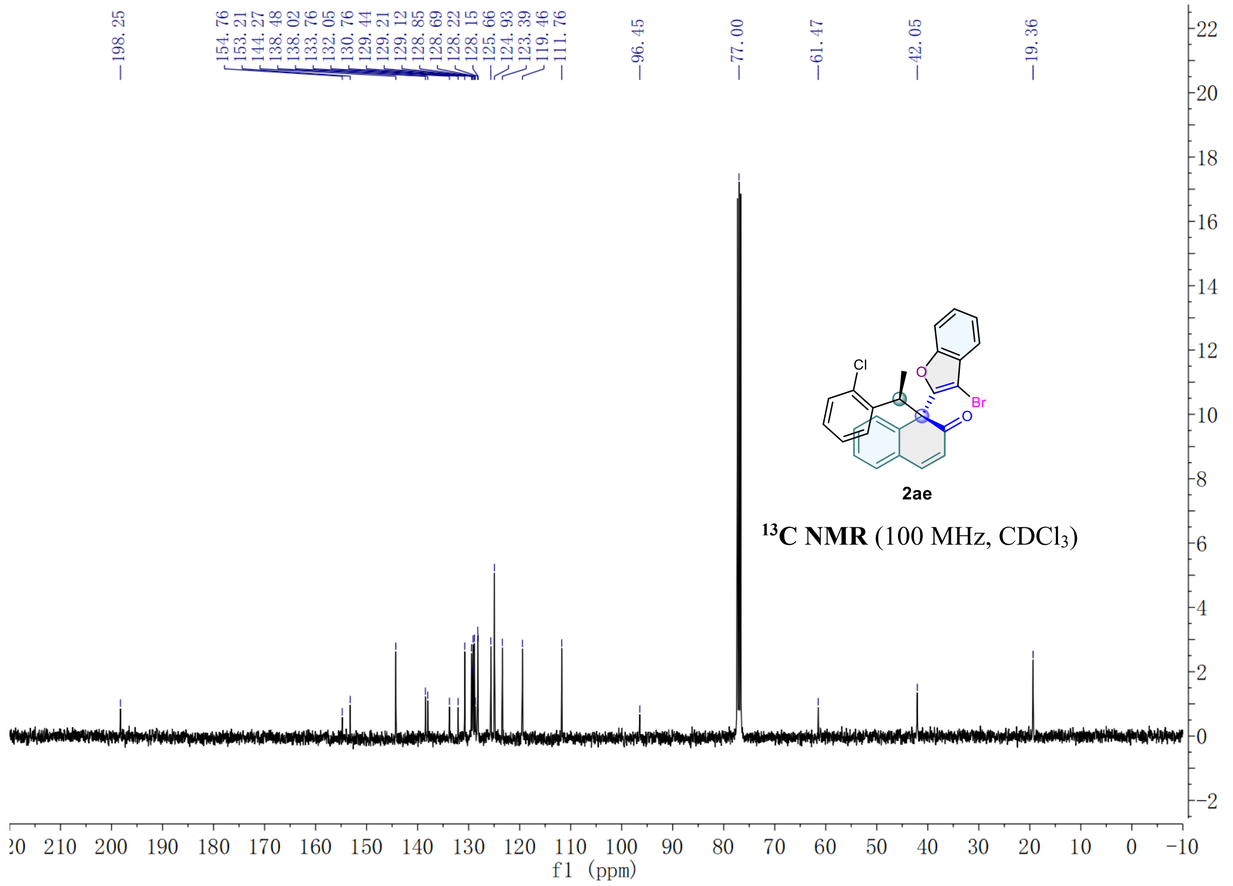
198.25
154.76 153.21 144.27 138.48 138.02 133.76 132.05 130.76 129.44 129.21 129.12 128.85 128.69 128.22 128.15 125.66 124.93 123.39 119.46 111.76
96.45
77.00
61.47
42.05
19.36
f1 (ppm)
2ae
^{13}C NMR (100 MHz, CDCl$_3$)

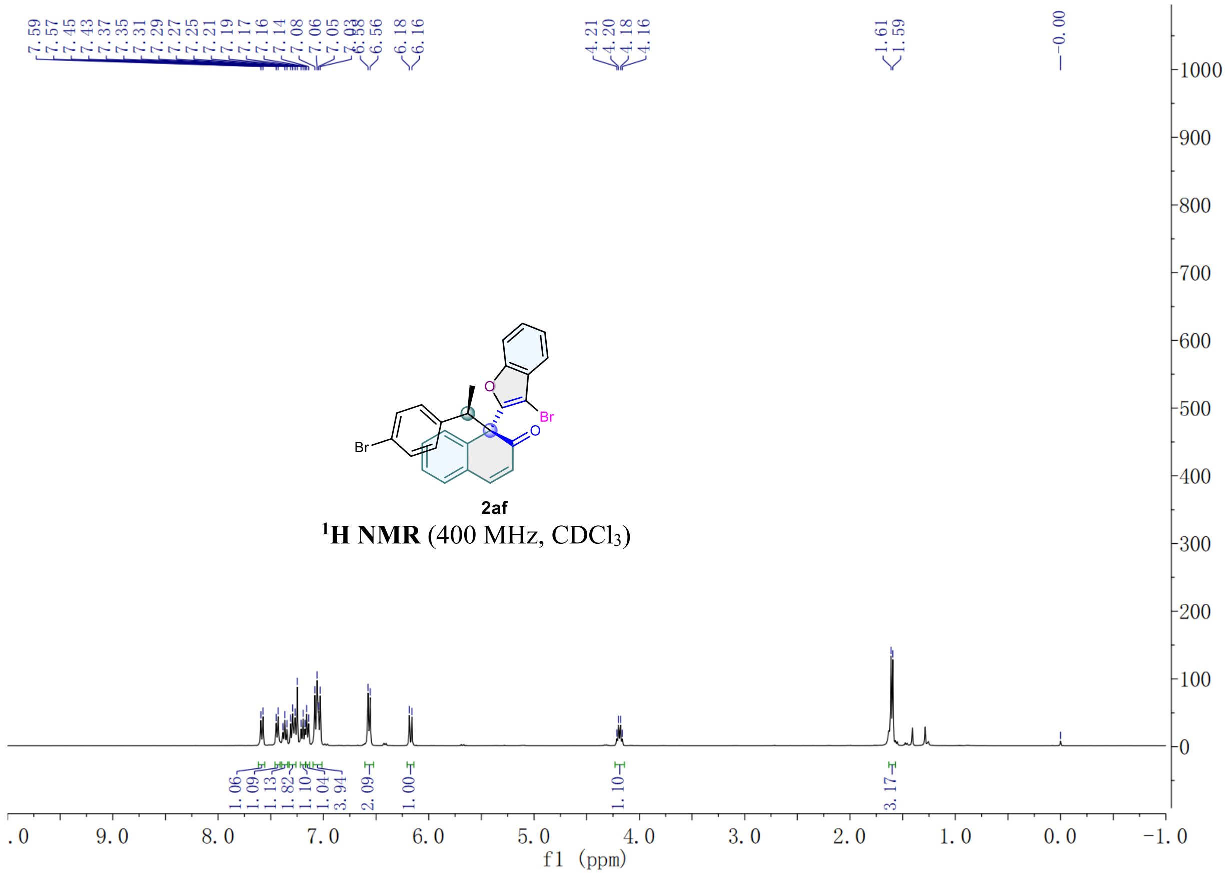
2af
1H NMR (400 MHz, CDCl3)
f1 (ppm)

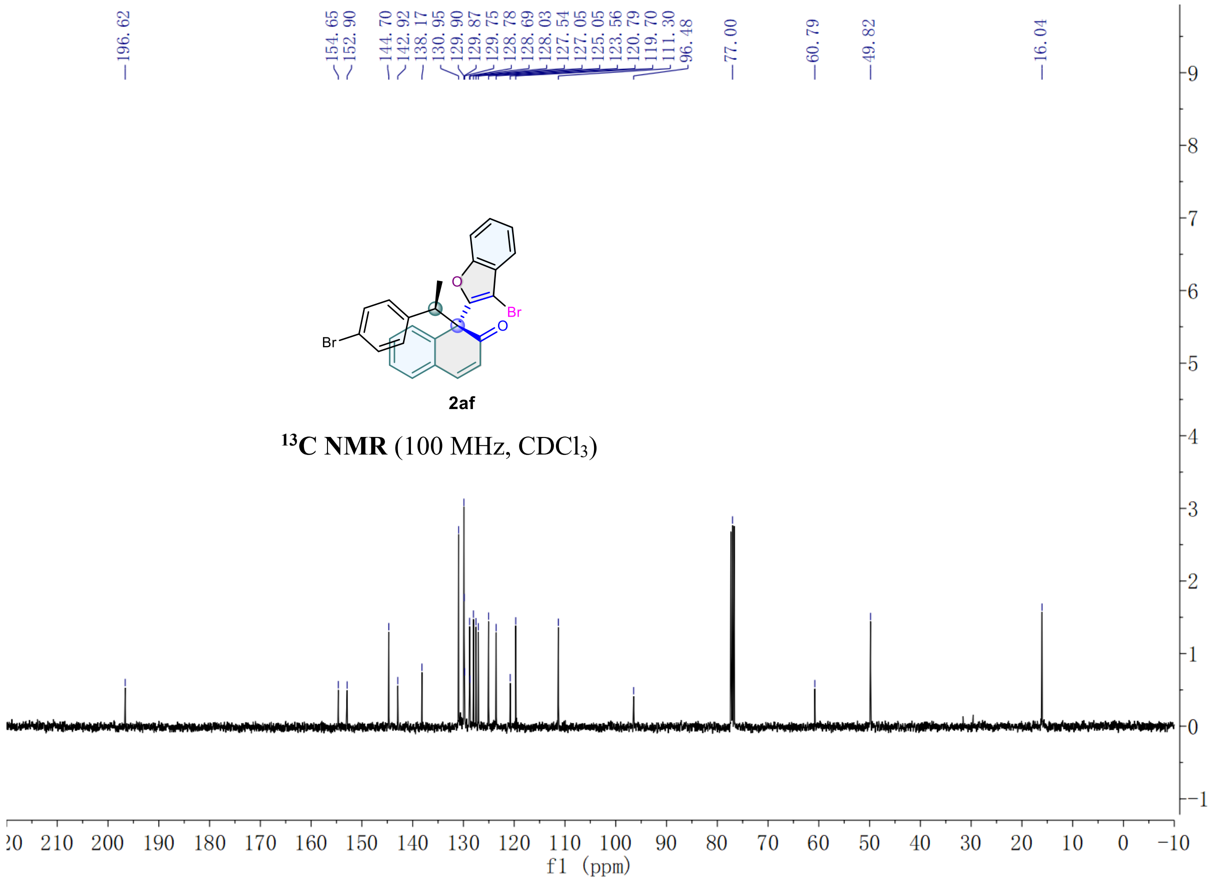
2af
13C NMR (100 MHz, CDCl3)
f1 (ppm)

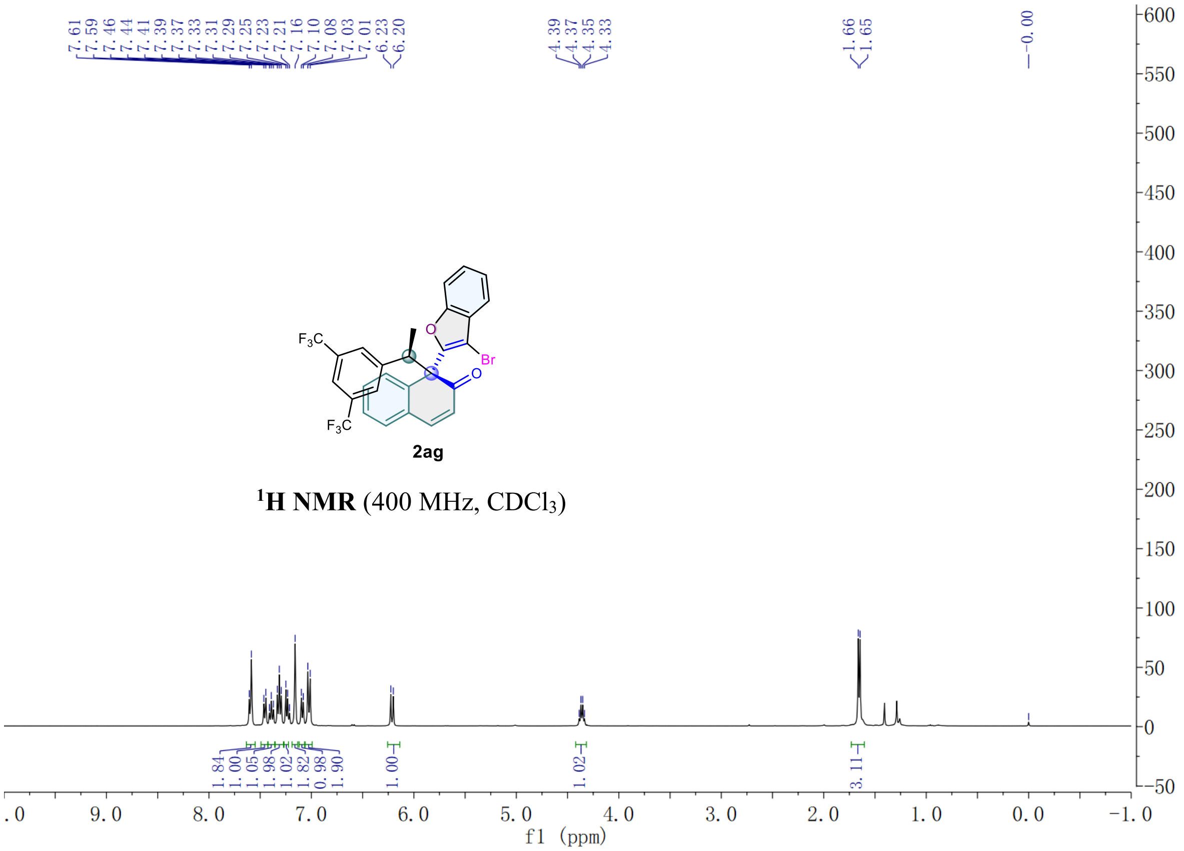

^{1}H NMR (400 MHz, $CDCl_3$)

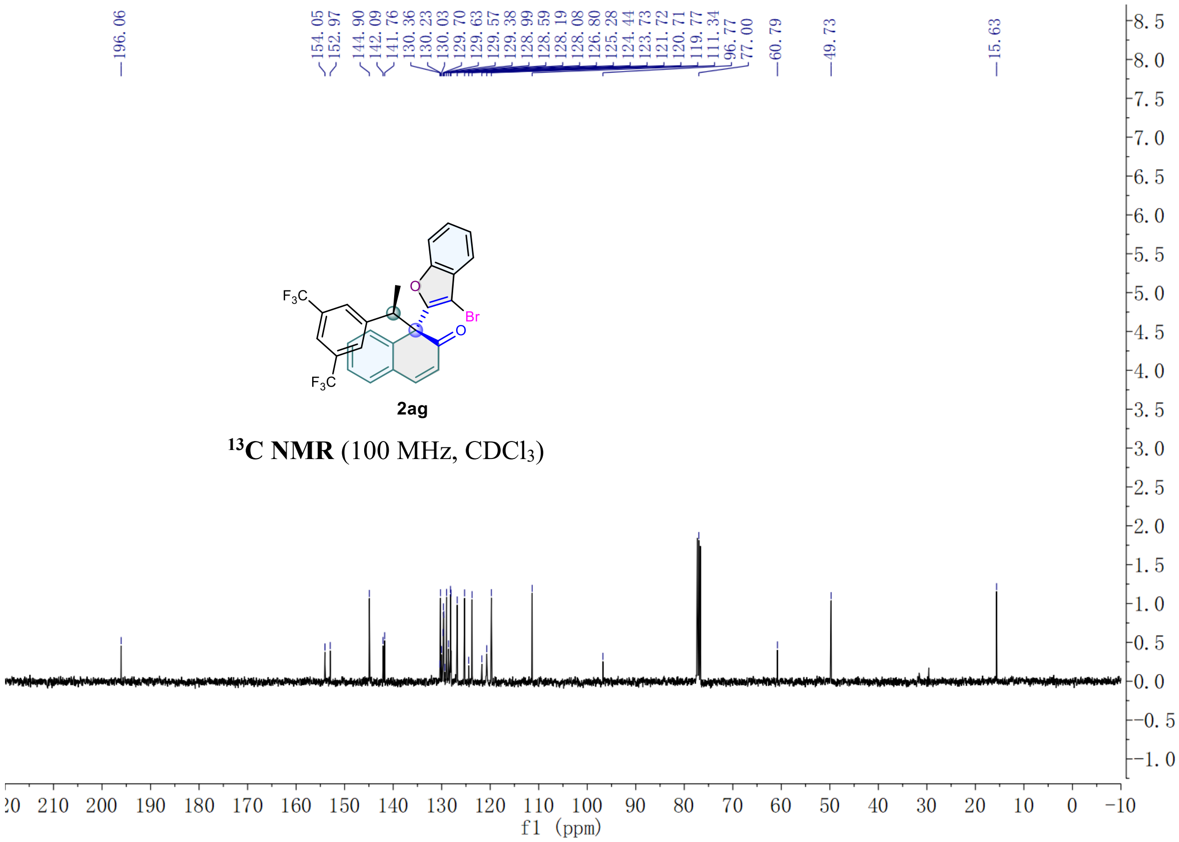

^{13}C NMR (100 MHz, $CDCl_3$)

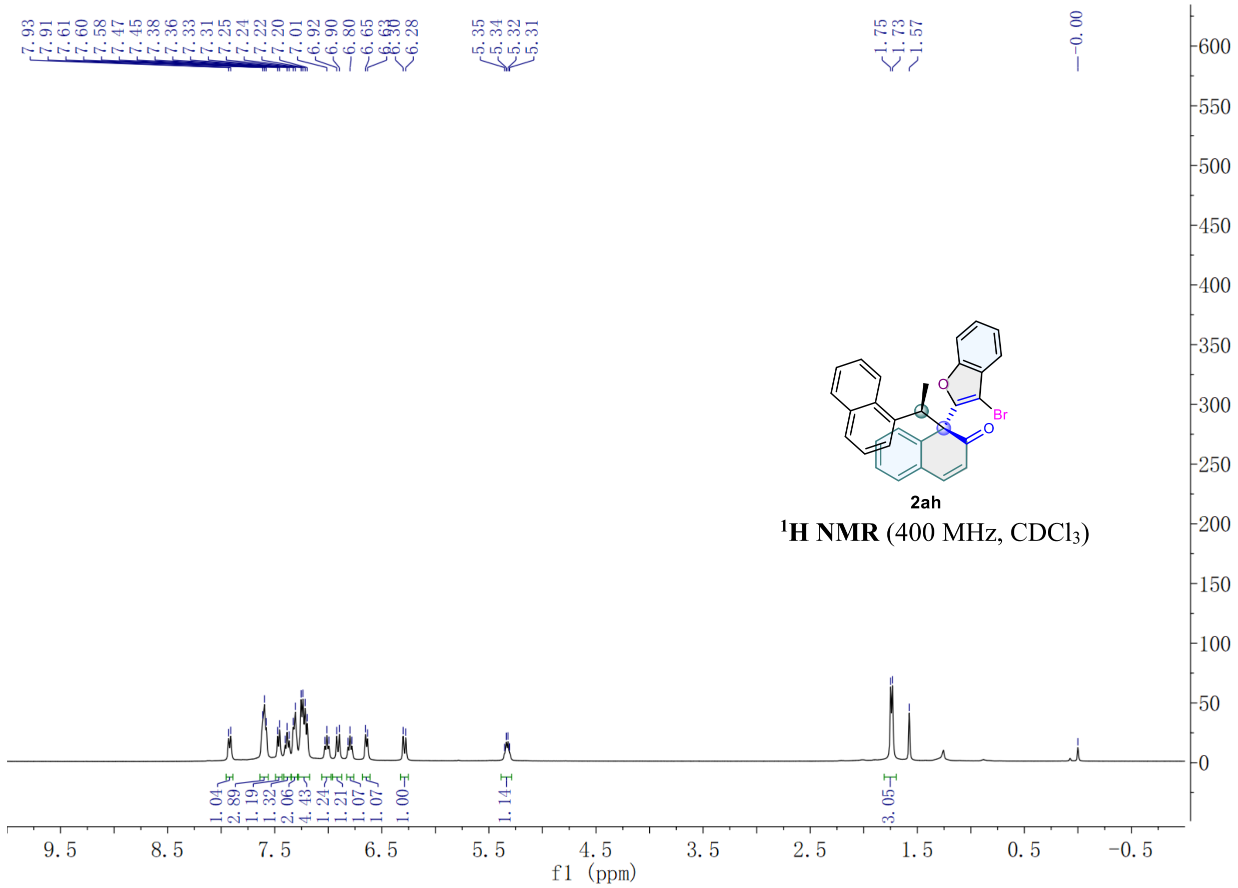
7.93 7.91 7.61 7.60 7.58 7.47 7.45 7.38 7.36 7.33 7.31 7.25 7.24 7.22 7.20 7.01 6.92 6.90 6.80 6.65 6.63 6.30 6.28
5.35 5.34 5.32 5.31
1.75 1.73 1.57
-0.00
O
Br
O
2ah
^{1}H NMR (400 MHz, $CDCl_3$)
1.04 2.89 1.19 1.32 2.06 4.43 1.21 1.21 1.07 1.07 1.00 1.14 3.05
f1 (ppm)

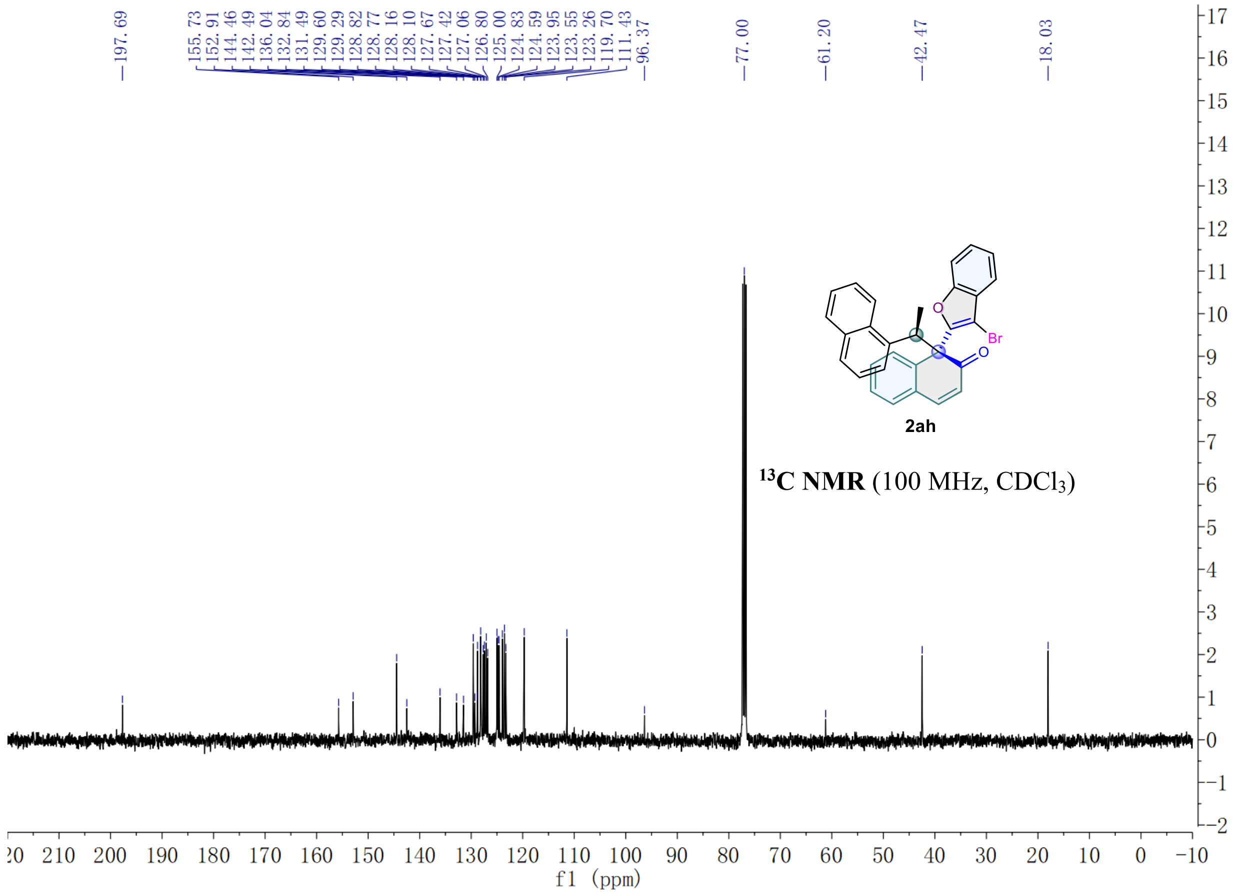
197.69
155.73 152.91 144.46 142.49 136.04 132.84 131.49 129.60 129.29 128.82 128.77 128.16 128.10 127.67 127.42 127.06 126.80 125.00 124.83 124.59 123.95 123.55 123.26 119.70 111.43 96.37
77.00
61.20
42.47
18.03
O
Br
O
2ah
^{13}C NMR (100 MHz, $CDCl_3$)
f1 (ppm)

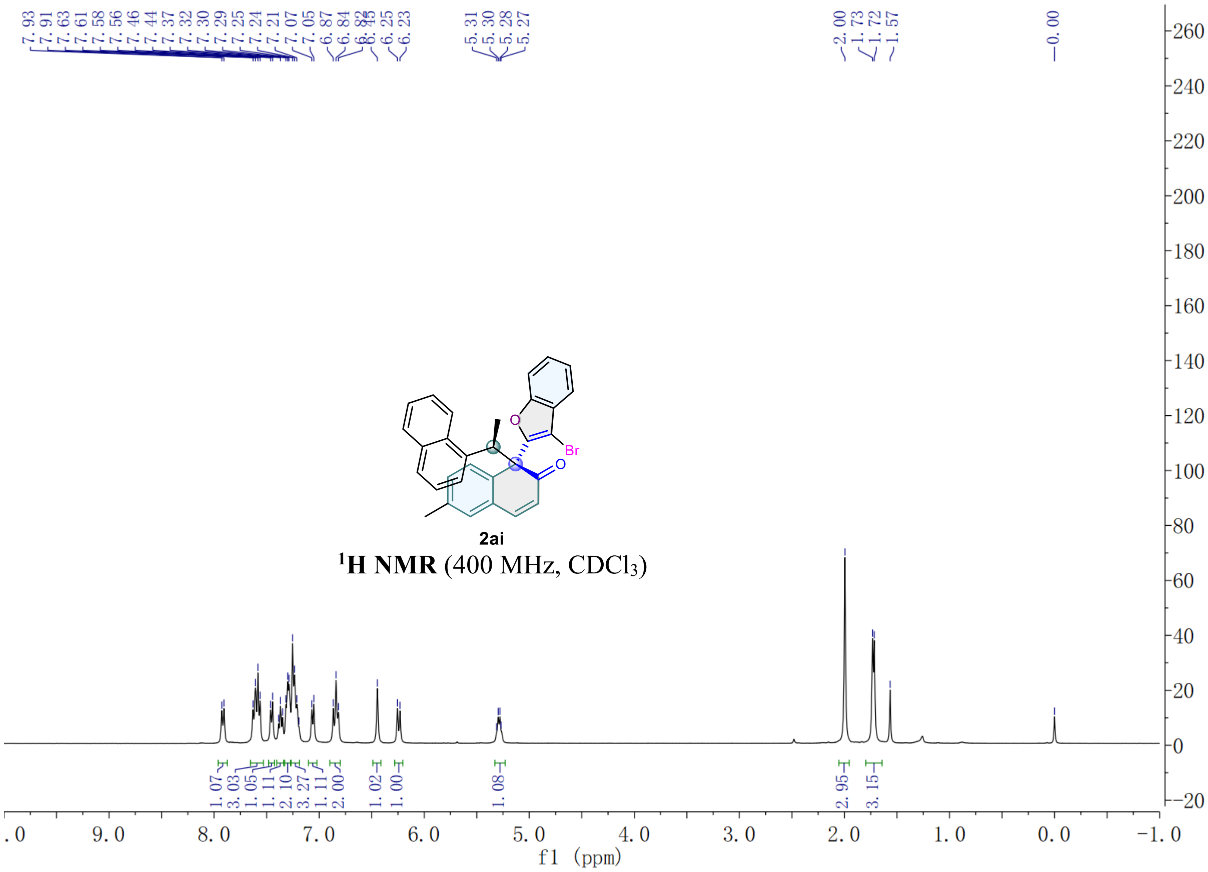

2ai

^{1}H NMR (400 MHz, $CDCl_3$)

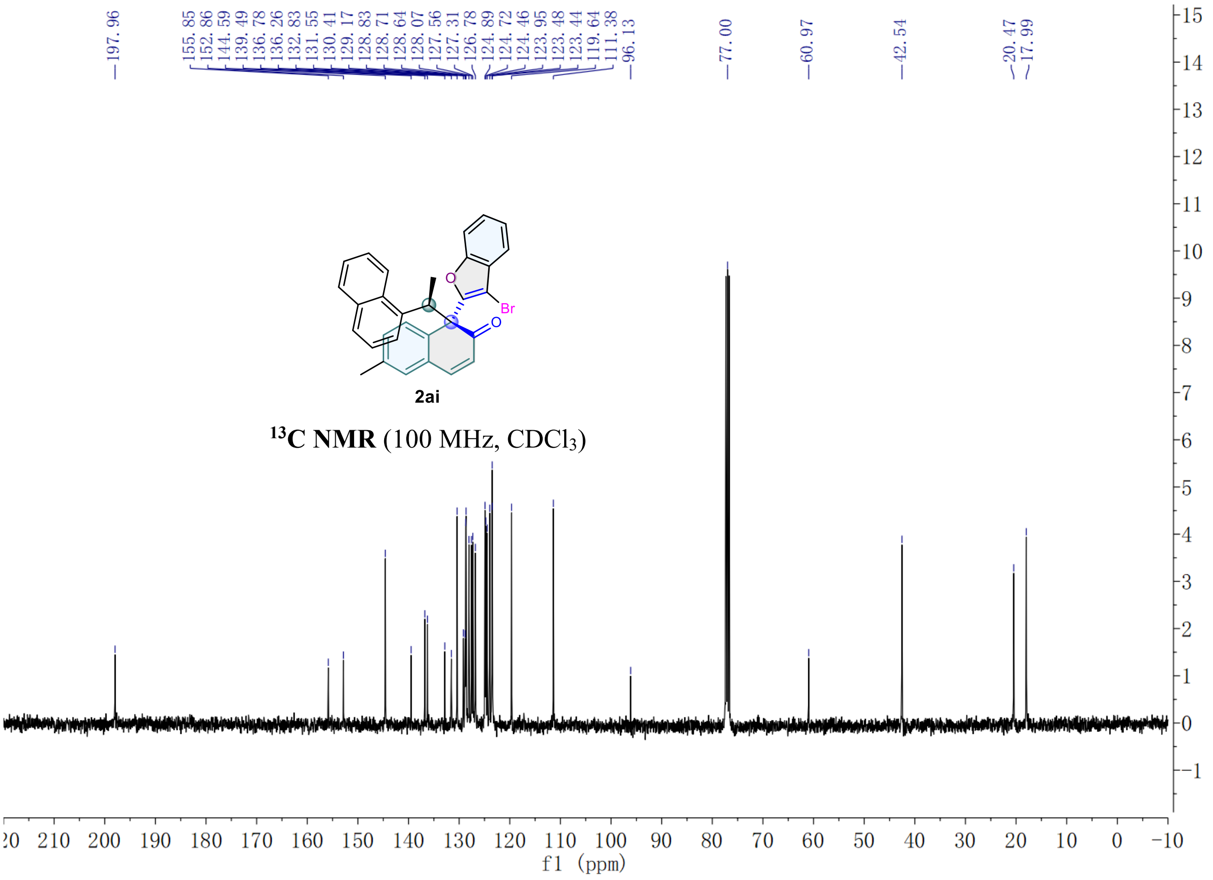

2ai

^{13}C NMR (100 MHz, $CDCl_3$)

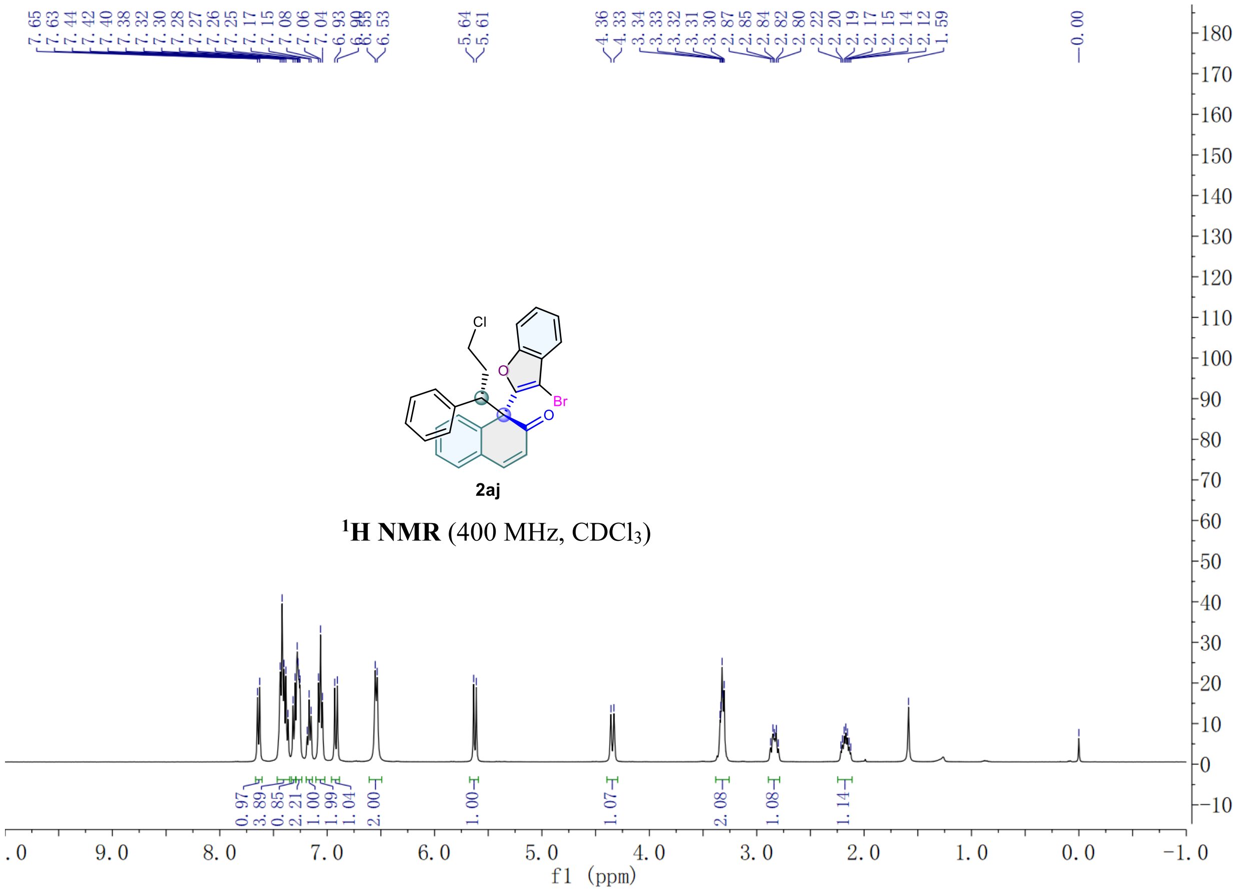

^{1}H NMR (400 MHz, $CDCl_3$)

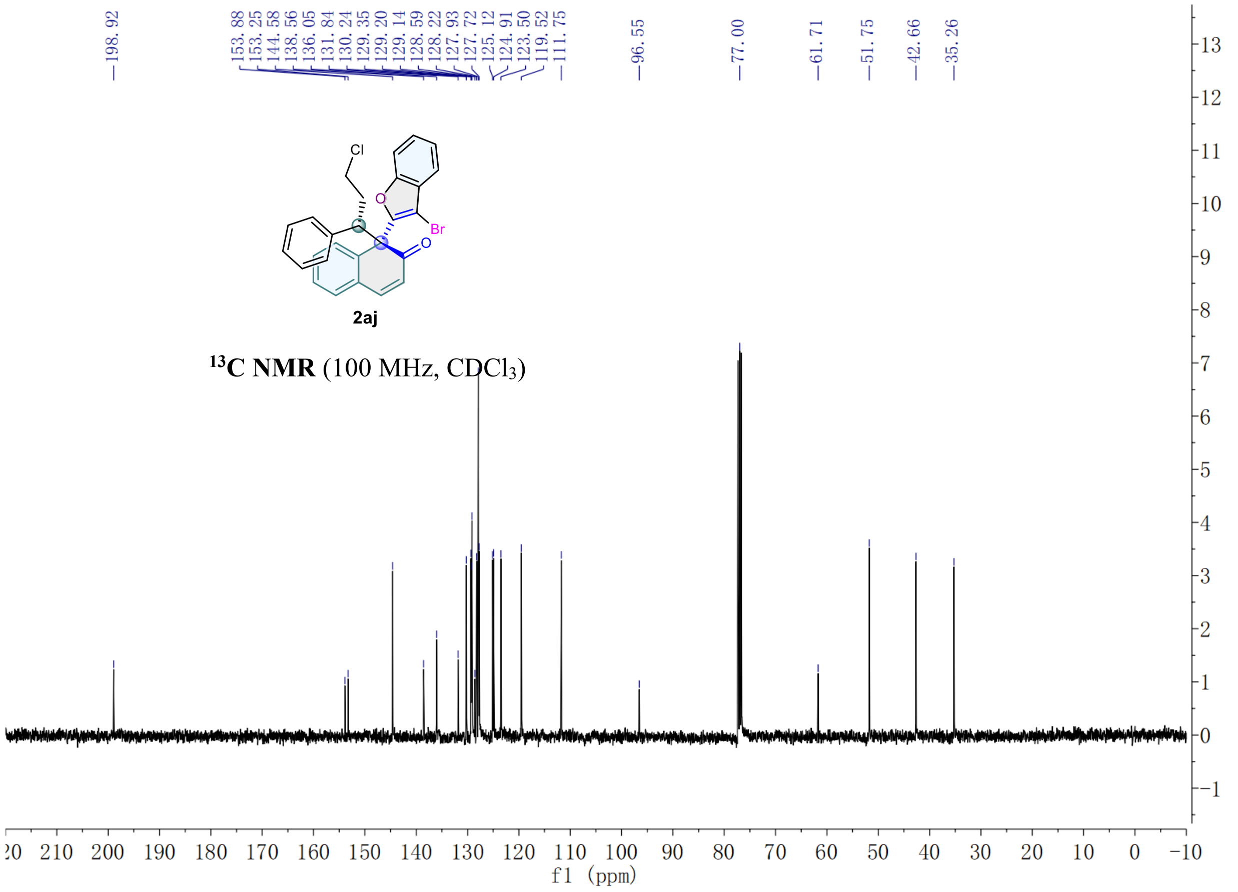

^{13}C NMR (100 MHz, $CDCl_3$)

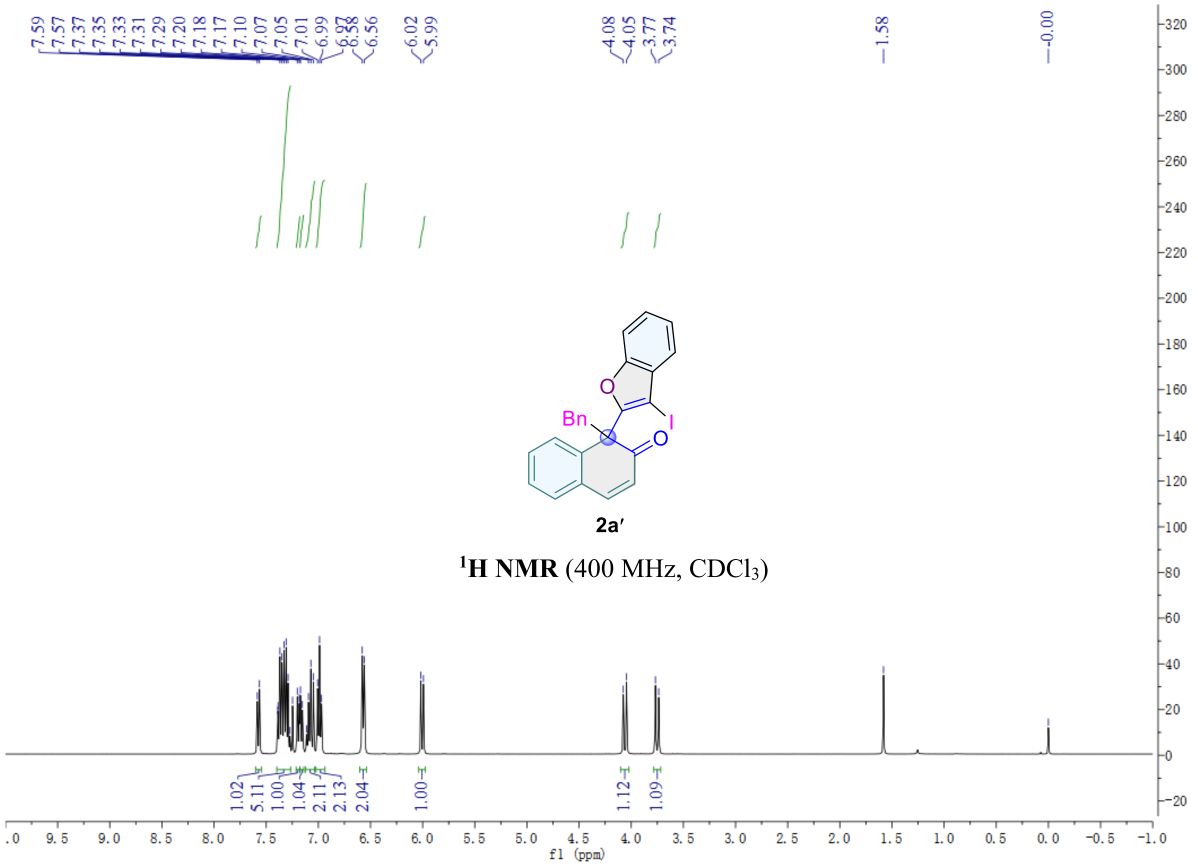

2a′

[1]**H NMR** (400 MHz, $CDCl_3$)

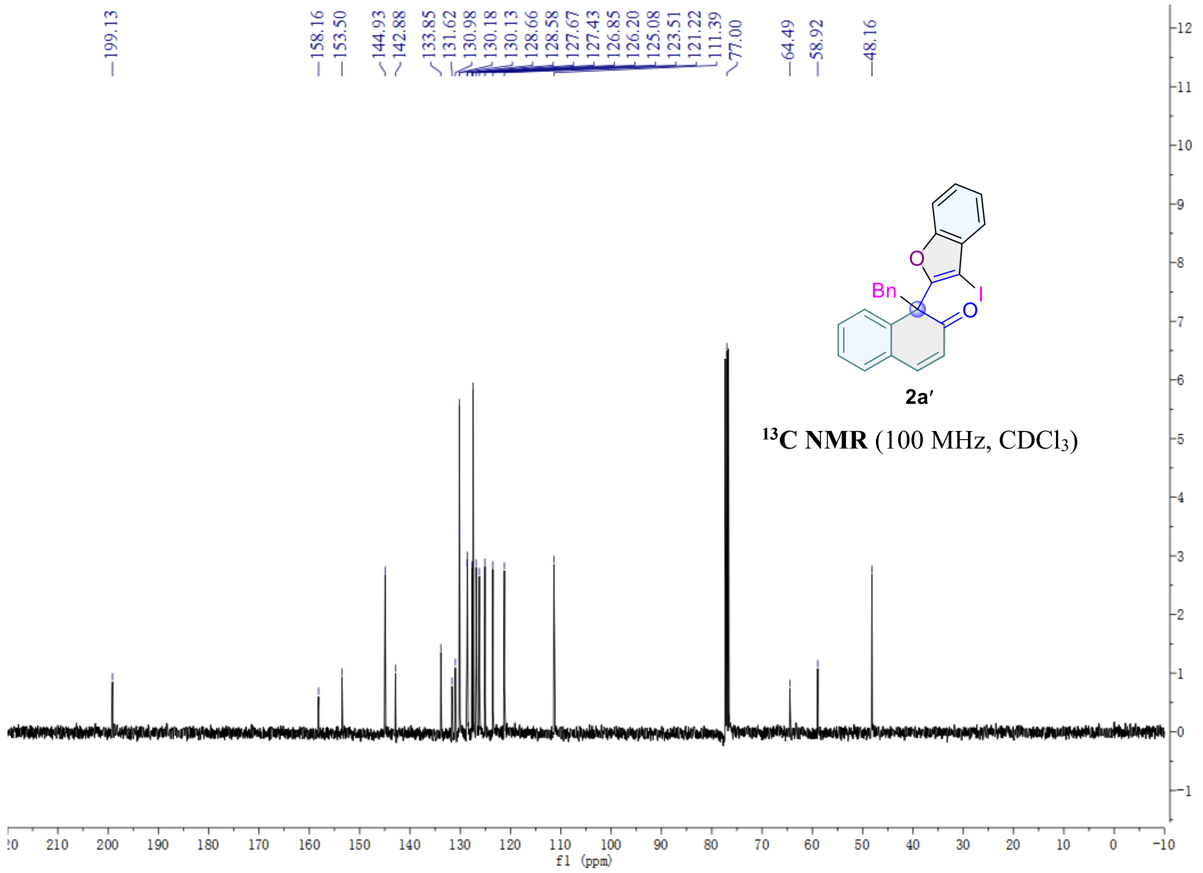

2a′

[13]**C NMR** (100 MHz, $CDCl_3$)

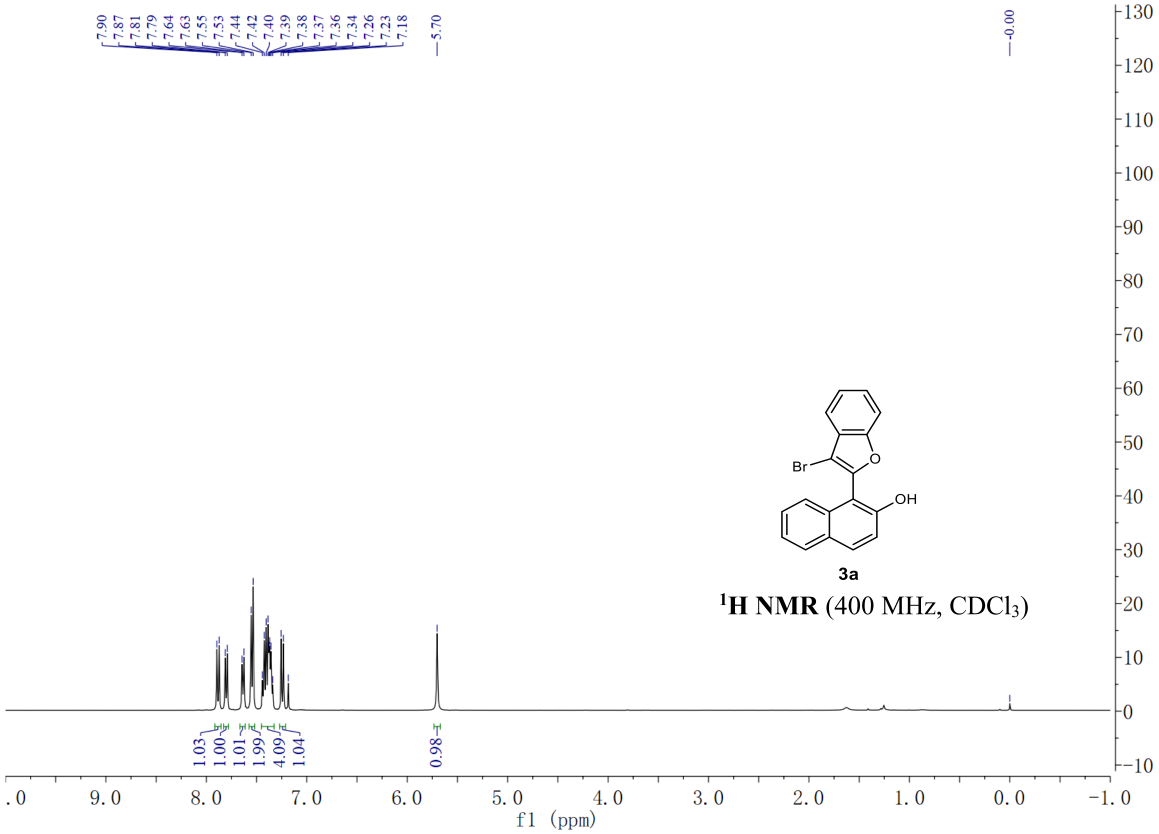

^{1}H NMR (400 MHz, $CDCl_3$)

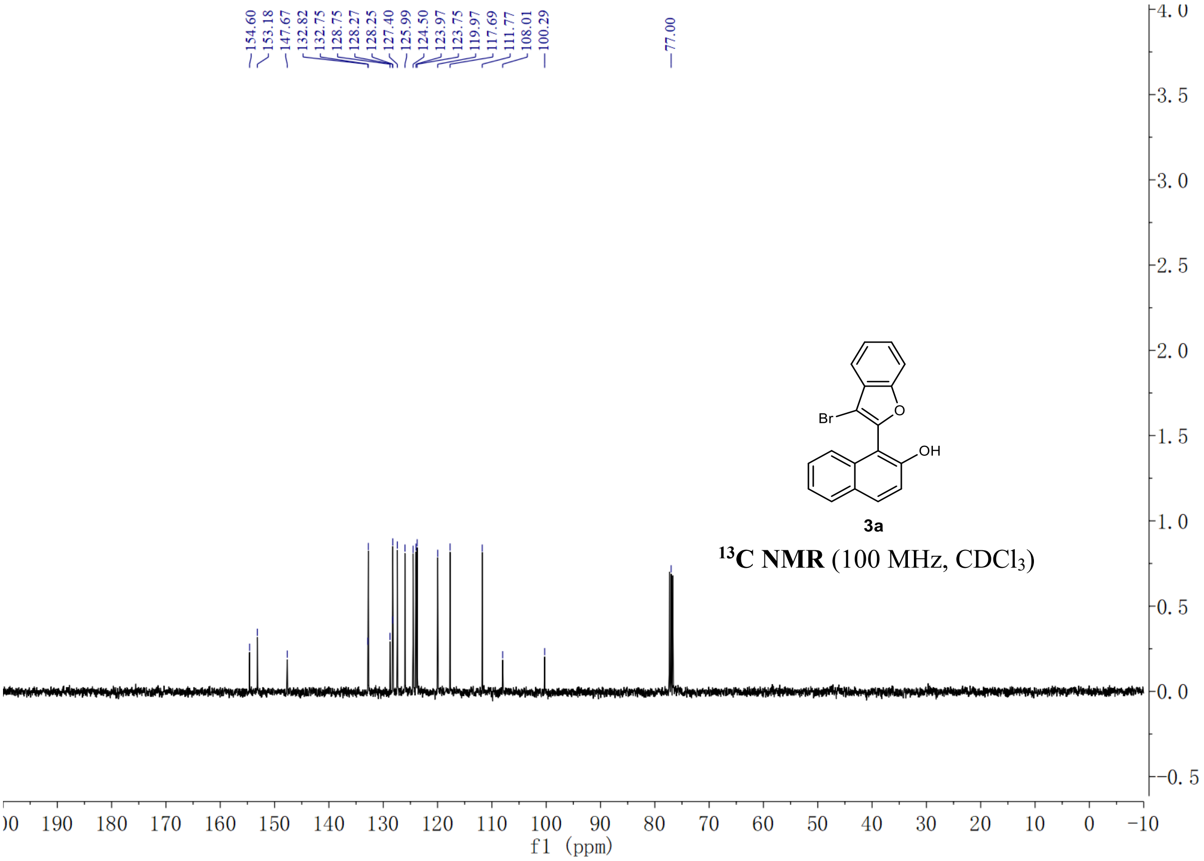

^{13}C NMR (100 MHz, $CDCl_3$)

X-ray crystallographic information

The authors thank Mr. Xiangnan Gong (Analytical and Testing Center of Chongqing University) for spectroscopic measurements.

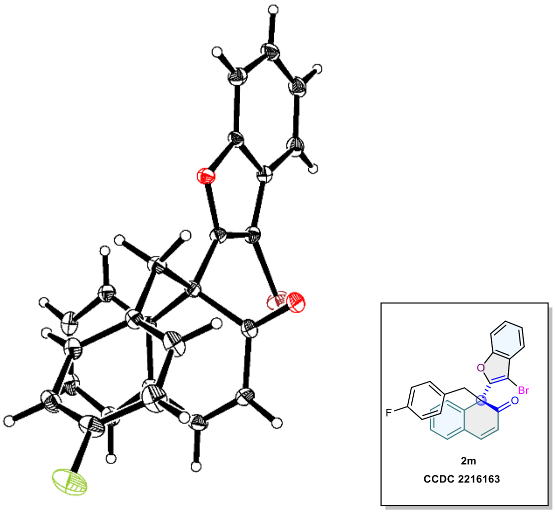

Figure S1. X-ray crystallographic information of **2m**.

Single crystals for X-ray studies were grown by slow evaporation of a solution of compound **2m** in a mixture of DCM/PE in a 3 mL tube at room temperature. The X-ray data of **2m** is deposited in the Cambridge Crystallographic Data Centre with a number of CCDC 2216163

Crystal Data for $C_{25}H_{16}BrFO_2$ (M =447.29 g/mol): orthorhombic, space group $P2_12_12_1$ (no. 19), a = 9.2974(8) Å, b = 14.0554(13) Å, c = 15.0863(13) Å, V = 1971.5(3) Å^3, Z = 4, T = 200.01(10) K, μ (Mo Kα) = 2.113 mm^{-1}, $Dcalc$ = 1.507 g/cm^3, 8010 reflections measured (6.956° ≤ 2Θ ≤ 57.97°), 4465 unique (R_{int} = 0.0346, R_{sigma} = 0.0704) which were used in all calculations. The final R_1 was 0.0482 (I > 2σ(I)) and wR_2 was 0.0959 (all data).

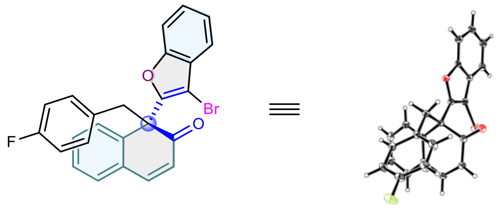

2m CCDC 2216163

Identification code	20220922-PL-4304-200K-2
Empirical formula	$C_{25}H_{16}BrFO_2$
Formula weight	447.29
Temperature/K	200.01(10)
Crystal system	orthorhombic
Space group	$P2_12_12_1$
a/Å	9.2974(8)
b/Å	14.0554(13)
c/Å	15.0863(13)
α/°	90
β/°	90
γ/°	90
Volume/Å^3	1971.5(3)
Z	4
ρ_{calc}g/cm^3	1.507
μ/mm^{-1}	2.113
F(000)	904.0
Crystal size/mm^3	0.45 × 0.42 × 0.39
Radiation	Mo Kα (λ = 0.71073)
2Θ range for data collection/°	6.956 to 57.97
Index ranges	$-11 \leqslant h \leqslant 12, -18 \leqslant k \leqslant 17, -20 \leqslant l \leqslant 18$
Reflections collected	8010
Independent reflections	4465 [R_{int} = 0.0346, R_{sigma} = 0.0704]
Data/restraints/parameters	4465/0/262
Goodness-of-fit on F^2	1.042
Final R indexes [I>=2σ (I)]	R_1 = 0.0482, wR_2 = 0.0832
Final R indexes [all data]	R_1 = 0.0718, wR_2 = 0.0959
Largest diff. peak/hole / e Å^{-3}	0.49/-0.69
Flack parameter	-0.013(8)

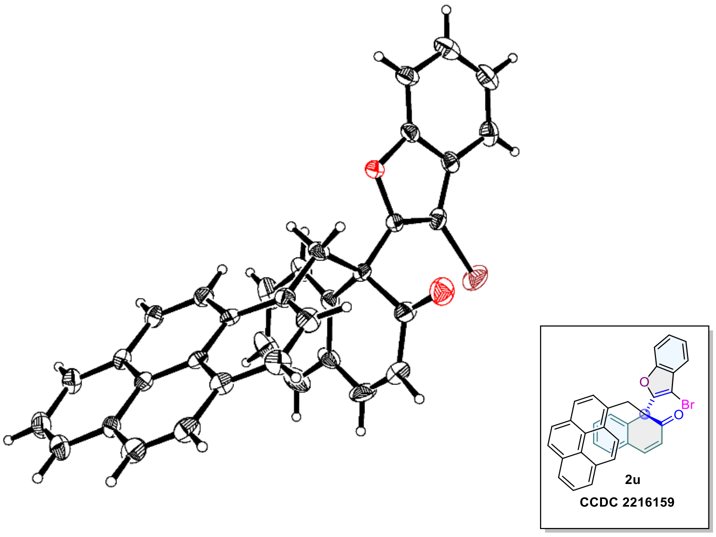

Figure S2. X-ray crystallographic information of **2u**.

Single crystals for X-ray studies were grown by slow evaporation of a solution of compound **2u** in a mixture of DCM/hexane in a 2 mL tube at room temperature. The X-ray data of **2u** is deposited in the Cambridge Crystallographic Data Centre with a number of CCDC 2216159

Crystal Data for $C_{35}H_{21}BrO_2$ (M = 553.43 g/mol): orthorhombic, space group $P2_12_12_1$ (no. 19), a = 7.7388(4) Å, b = 13.0068(8) Å, c = 25.1855(18) Å, V = 2535.1(3) Å^3, Z = 4, T = 298.15 K, μ (Mo K α) = 1.654 mm^{-1}, *Dcalc* = 1.450 g/cm^3, 10661 reflections measured (3.234° ⩽ 2Θ ⩽ 57.99°), 5766 unique (R_{int} = 0.0334, R_{sigma} = 0.0754) which were used in all calculations. The final R_1 was 0.0564 (I > 2 σ (I)) and wR_2 was 0.1128 (all data).

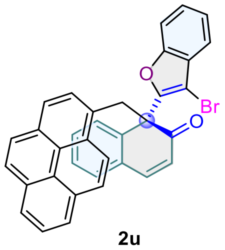

2u

≡

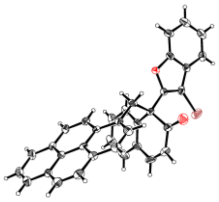

CCDC 2216159

Identification code	20220414-pl-3795
Empirical formula	$C_{35}H_{21}BrO_2$
Formula weight	553.43
Temperature/K	298.15
Crystal system	orthorhombic
Space group	$P2_12_12_1$
a/Å	7.7388(4)
b/Å	13.0068(8)
c/Å	25.1855(18)
α/°	90
β/°	90
γ/°	90
Volume/Å^3	2535.1(3)
Z	4
ρ_{calc}g/cm^3	1.450
μ/mm^{-1}	1.654
F(000)	1128.0
Crystal size/mm^3	0.26 × 0.19 × 0.18
Radiation	Mo Kα (λ = 0.71073)
2Θ range for data collection/°	3.234 to 57.99
Index ranges	$-10 \leqslant h \leqslant 6, -17 \leqslant k \leqslant 9, -33 \leqslant l \leqslant 25$
Reflections collected	10661
Independent reflections	5766 [R_{int} = 0.0334, R_{sigma} = 0.0754]
Data/restraints/parameters	5766/0/343
Goodness-of-fit on F^2	1.102
Final R indexes [I>=2σ (I)]	R_1 = 0.0564, wR_2 = 0.0928
Final R indexes [all data]	R_1 = 0.1249, wR_2 = 0.1128
Largest diff. peak/hole / e Å^{-3}	0.29/-0.41
Flack parameter	-0.005(6)

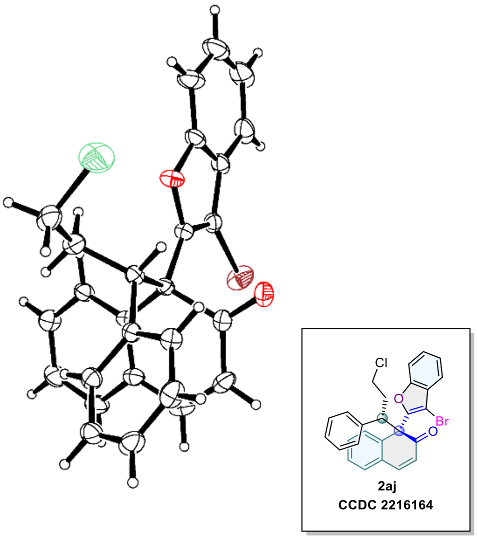

Figure S3. X-ray crystallographic information of **2aj**.

Single crystals for X-ray studies were grown by slow evaporation of a solution of compound **2aj** in a mixture of DCM/PE in a 2 mL tube at room temperature. The X-ray data of **2aj** is deposited in the Cambridge Crystallographic Data Centre with a number of CCDC 2216164

Crystal Data for $C_{27}H_{20}BrClO_2$ (M =491.79 g/mol): orthorhombic, space group $P2_12_12_1$ (no. 19), a = 10.1359(5) Å, b = 13.7691(7) Å, c = 16.5452(9) Å, V = 2309.1(2) Å^3, Z = 4, T = 293(2) K, μ (Mo K α) = 1.917 mm^{-1}, $Dcalc$ = 1.415 g/cm^3, 9196 reflections measured (7.012° ≤ 2Θ ≤ 58.118°), 5245 unique (R_{int} = 0.0277, R_{sigma} = 0.0636) which were used in all calculations. The final R_1 was 0.0469 (I > 2 σ (I)) and wR_2 was 0.0996 (all data).

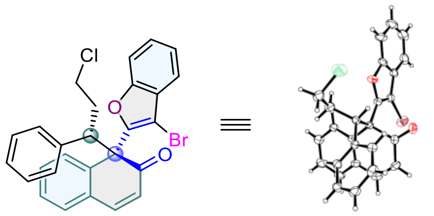

2aj **CCDC 2216164**

Identification code	20220926-PL-4314
Empirical formula	$C_{27}H_{20}BrClO_2$
Formula weight	491.79
Temperature/K	293(2)
Crystal system	orthorhombic
Space group	$P2_12_12_1$
a/Å	10.1359(5)
b/Å	13.7691(7)
c/Å	16.5452(9)
α/°	90
β/°	90
γ/°	90
Volume/Å^3	2309.1(2)
Z	4
ρ_{calc}g/cm^3	1.415
μ/mm^{-1}	1.917
F(000)	1000.0
Crystal size/mm^3	0.39 × 0.38 × 0.35
Radiation	Mo Kα (λ = 0.71073)
2Θ range for data collection/°	7.012 to 58.118
Index ranges	-11 ≤ h ≤ 13, -18 ≤ k ≤ 17, -11 ≤ l ≤ 22
Reflections collected	9196
Independent reflections	5245 [R_{int} = 0.0277, R_{sigma} = 0.0636]
Data/restraints/parameters	5245/0/280
Goodness-of-fit on F^2	1.010
Final R indexes [I>=2σ (I)]	R_1 = 0.0469, wR_2 = 0.0799
Final R indexes [all data]	R_1 = 0.1004, wR_2 = 0.0996
Largest diff. peak/hole / e Å^{-3}	0.23/-0.63
Flack parameter	-0.026(7)

Biological evaluation

Cell culture

A375, Hela, HepG-2, MCF-7, MIA PaCa-2, A549, CT-26, PANC-1 and HT-29 cells were cultured with dulbecco's modified eagle's medium (DMEM, gibco) containing 10% foetal bovine serum (FBS, gibco) and 1% penicillin-streptomycin (Sigma-Aldrich) under a 5% CO_2 humidified atmosphere at 37 °C. HCT-116 cells were cultured with Iscove's Modified Dulbecco's Medium (IMDM) supplemented with 10% FBS and 1% penicillin-streptomycin under a 5% CO_2 humidified atmosphere at 37 °C.

Anti-proliferation assay

The antiproliferative activities of the chiral benzofuran derivatives against the mentioned cancer cell lines were evaluated by MTT assay.[5] Cells in logarithmic growth phase were seeded in 96-well plates at a density of 2000 cells/100μL/ well and cultured for 12 h. After treated with the target compounds at a series concentration for 72 h under a 5% CO_2 humidified atmosphere at 37 °C. Then ,10 μL thiazolyl blue tetrazolium bromide solution (MTT， 5mg/mL) was added and incubated with cells for additional 4 h. The supernatant was discarded and the cells attached were dissolved with DMSO. Afterward， the absorbance at 490 nm was measured with an ELISA reader (Tecan, Austria). Each assay was performed three times at least.

Apoptosis assay

The cell apoptosis analysis assay was conducted using Annexin V-FITC apoptosis detection kit.[6] A375 cells were seeded in 12-well plates at a density of 1×10^5 cells/well and incubated for another 12 h. After the adherent cell growth, the culture medium was replaced with dulbecco's modified eagle's medium containing compound **2f** in different concentrations and cells were cultured for scheduled time. The treated cells were collected and washed with cold PBS. Then the cells were suspended with 195 μL Annexin V-FITC binding buffer.5 μL Annexin V-FITC and 10μL PI were added and incubated for 20 min in dark at room temperature. The cells were detected by Analytical Flow cytometry within 1 h and the results were analyzed using CytExpert software.

In Vitro ROS Generation

ROS generation was detected using DCFH-DA, an accurate ROS-sensitive fluorescence sensor.[7] A375 cells were seeded in 12-well plates at a density of 10^5 cells/well and incubated for another 12 h so as to adherent cell growth. After the incubation of A375 cells and **2f** with different concentrations (0,2, 4, and 8 μM) for 24 h, the culture medium was replaced with DCFH-DA-containing (2 μM) dulbecco's modified eagle's medium and the cells were incubated for another 20 min. Then the cells were trypsinized, washed, collected and finally detected by Analytical Flow cytometry.

Detection of Mitochondrial membrane potential (MMP)

Mitochondria membrane potential (MMP) was detected by using MMP assay kit with JC-1 (Beyotime, China) according to the manufacture's protocol.[8] A375 cells stimulated with gradient concentrations (0, 2, 4, 8 μM) of compound **2f** were collected and incubated with JC-1 working solution for 20 min at 37 °C. After being washed again and suspended with JC-1 buffer solution，A375 cells were analyzed by flow cytometry at emission wavelengths of 530 nm (green) and 575 nm (re).

Cell death manner assay

The manners of cells death were further analyzed by using cell apoptosis inhibitor Z-VAD-FMK and

cell necrosis inhibitor necrostatin-1. A375 cells were seeded in 96-well plates at a density of 2000 cells/100 μL/ well and cultured for 12 h. Subsequently, cells were incubated with DMEM containing **2f** (2 μM) and Z-VAD-FMK (10 μM) / necrostatin-1 (10 μM) for 72 h. Cell viability was assessed by MTT assay mentioned above. Each assay was performed three times at least.

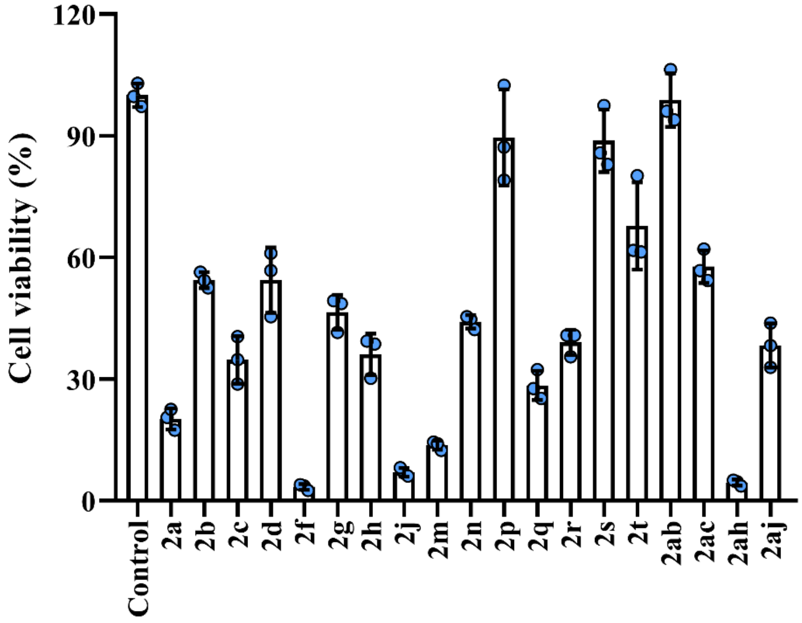

Figure S4. Viability of MCF-7 cells after the incubation with **2f** (20 μM) for 72h.

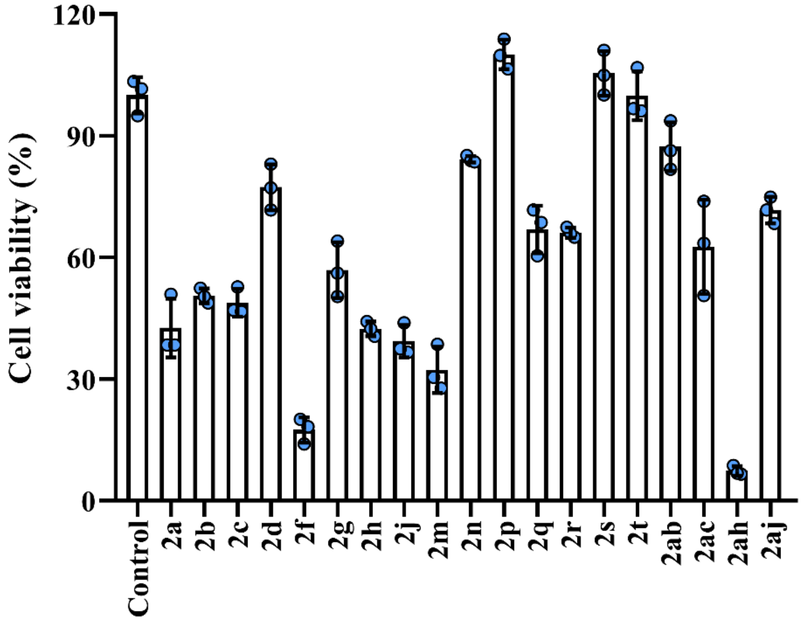

Figure S5. Viability of Mia PaCa-2 cells after the incubation with **2f** (20 μM) for 72h.

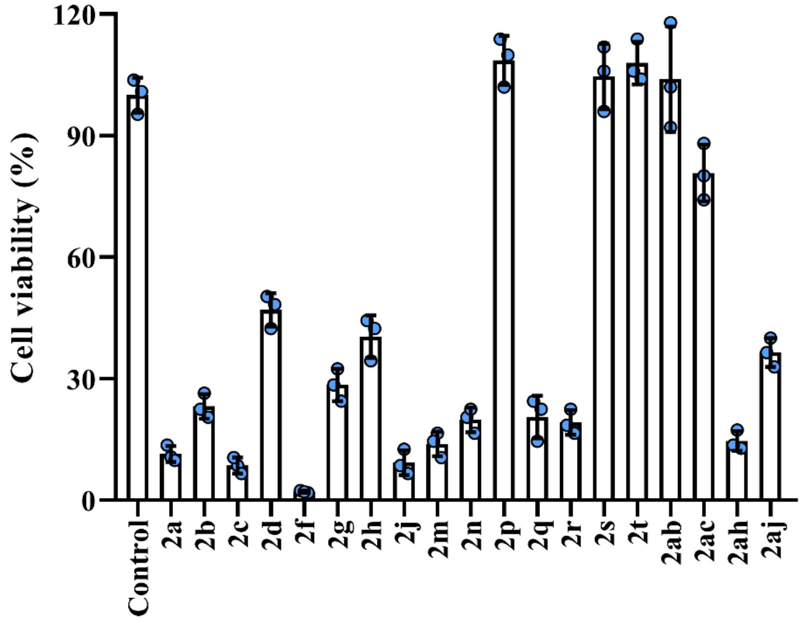

Figure S6. Viability of A549 cells after the incubation with **2f** (20 μM) for 72h.

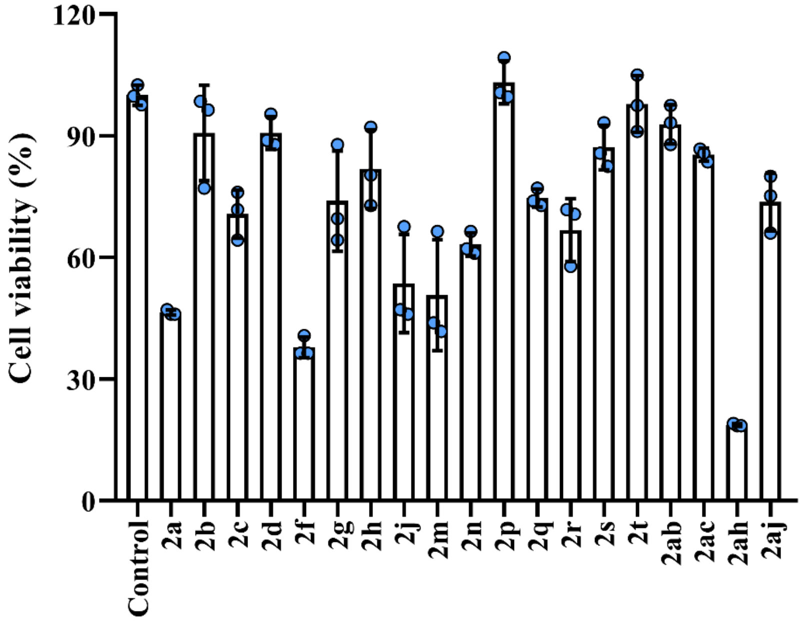

Figure S7. Viability of CT-26 cells after the incubation with **2f** (20 μM) for 72h.

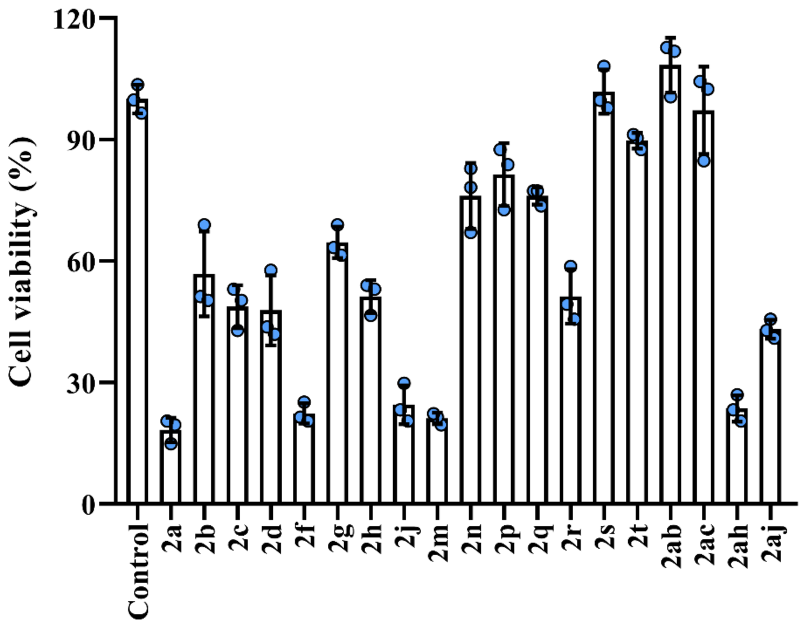

Figure S8. Viability of PANC-1 cells after the incubation with **2f** (20 μM) for 72h.

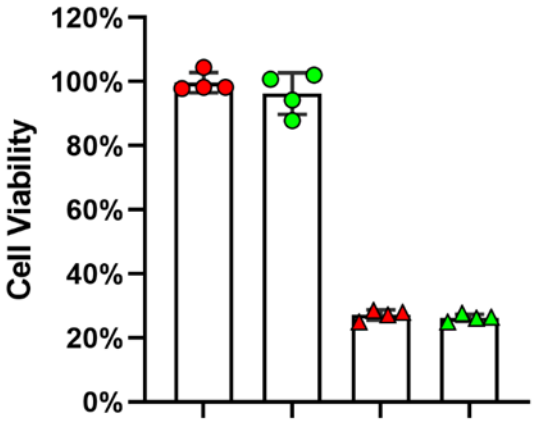

Figure S9. The percentage of cell viability after the incubation with **2f** (2 μM) and necrosis inhibitor necrostatin-1 (10 μM) for 72 h.

We had evaluated the anticancer activity of 29 synthesized compounds (with 7 compounds excluded from testing due to solubility issues in the assay solution; representative data are shown in Fig. S10) on several cancer cell lines. Depending on the cell line, the observed potency varied, which would lead to distinct SAR models. While most compound only showed moderate to poor potency toward A375 (IC50 > 20 μM), however, compound **2f** exhibited considerable inhibition activity with an IC50 value of approximately 1 μM.

Cell viability of different cancer cells after treated with tested compounds (%)			
	MCF-7	CT26	A549
Items	Average	Average	Average
Control	100.00	100.00	100.00
**2a**	20.21	46.43	11.40
**2b**	54.43	90.71	23.17
**2c**	34.71	70.72	8.60
**2d**	54.43	90.72	47.01
**2f**	3.42	37.83	2.11
**2g**	46.52	73.93	28.47
**2h**	36.12	81.79	40.39
**2i**	12.02	91.01	12.04
**2j**	7.04	53.60	9.27
**2k**	29.02	87.64	14.41
**2l**	2.03	81.44	10.81
**2m**	13.70	50.72	13.90
**2n**	44.16	63.21	19.86
**2o**	7.03	90.85	11.13
**2p**	89.61	103.21	108.60
**2q**	28.45	74.64	20.52
**2r**	39.08	66.79	19.20
**2s**	88.78	87.14	104.63
**2t**	67.77	97.86	107.94
**2v**	5.86	84.49	7.77
**2z**	1.85	78.79	6.82
**2ab**	98.82	92.86	103.97
**2ac**	57.73	85.36	80.79
**2ad**	19.12	85.04	13.47
**2ae**	2.65	87.59	5.50
**2af**	17.43	86.78	11.95
**2ag**	14.20	79.35	12.08
**2ah**	4.49	18.71	14.61
**2aj**	38.33	74.74	36.49

Figure S10. Cell viability assay with 10 μM of most synthesized compounds for 72h

Plausible catalytic cycle

In order to have a clearer understanding of the rearrangement mechanism, a plausible reaction mechanism has been proposed based on the control experiments and the investigated VQM-based reactions (Fig. S11). Initially, complex I was formed by coordination of NBS and catalyst with **1a**, followed by the enantioselective generation of chiral brominated VQMs intermediate via a proton shift. Subsequently, the electron-rich oxygen atom in substrate **1a** undergoes a nucleophilic reaction at the C4 position of VQM, leading to heterolytic cleavage of the C–O bond and forming an active tight ion pair intermediate, which generates TS-1. Finally, intramolecular [1,3] rearrangement produces the desired benzofuran derivative **2a** and releases the catalyst.

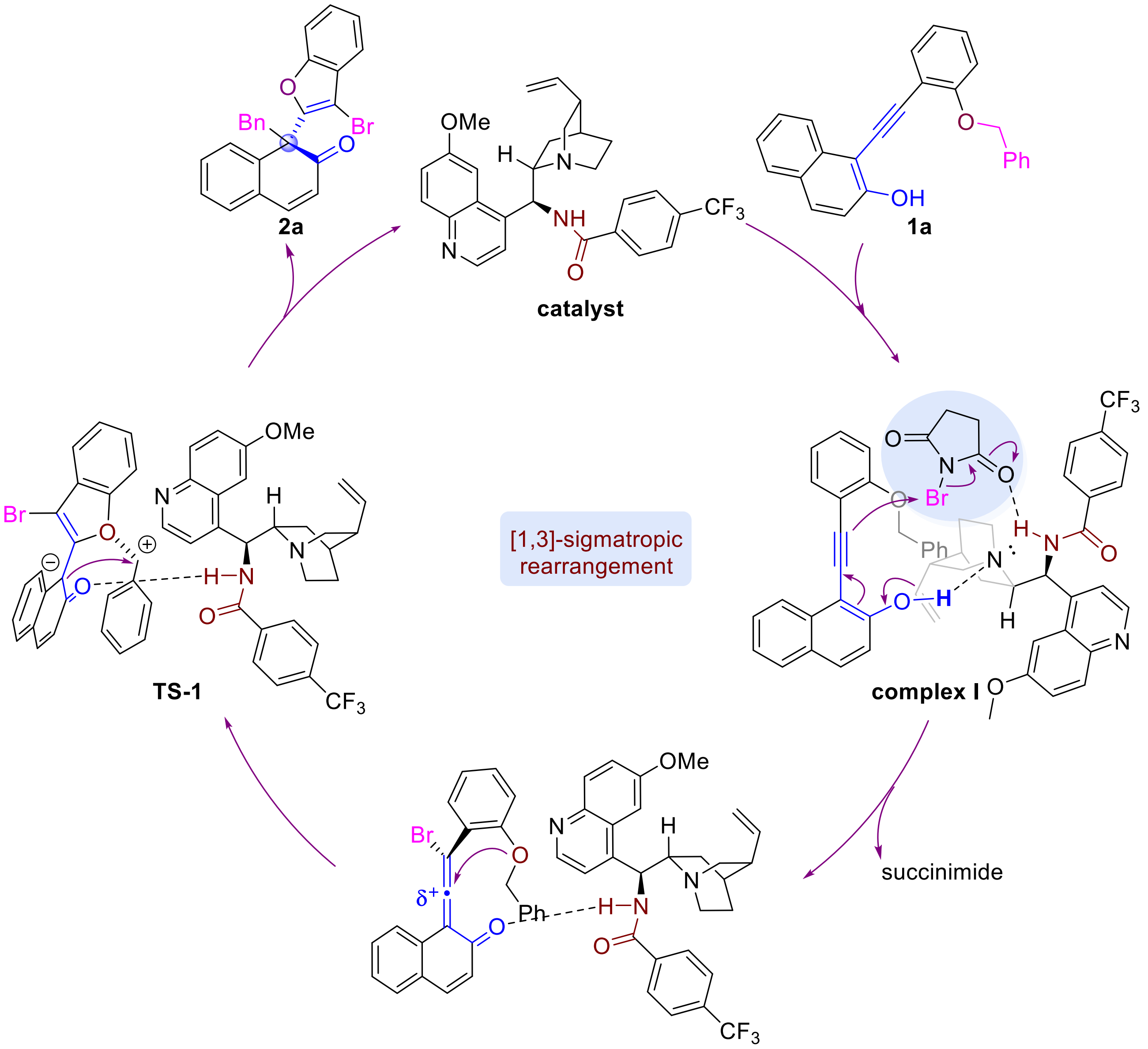

Figure S11. Plausible catalytic cycle

Theoretical calculations

Based on previous studies on VQMs and controlled experiments, we propose that the reaction first generates a chiral VQM intermediate, followed by a subsequent chiral transfer process that leads to the formation of the product. Consequently, we chose **1m** as the model reactant to conduct theoretical calculations in order to investigate the possible transition states involved in the chiral transfer process. We set the energy of VQM as the zero point, and the results indicate that the transition state barrier for the formation of the carbon-oxygen bond between the VQM and oxygen is 24.9 kcal/mol, while the barrier for the migration of the resulting carbocation is 31.7 kcal/mol (Fig. S12). Based on the control experiment results and theoretical calculations, the rearrangement should involve a tight ion pair intermediate.

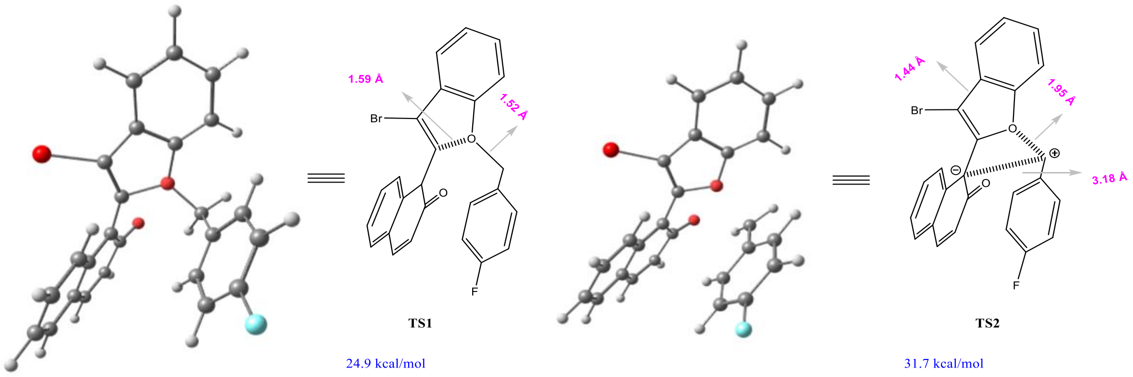

Figure S12. Theoretical calculations

B3-LYP-D3 and M06-2X calculated absolute energies, and free energies of all structure

Geometry	$E_{(elec\text{-}B3\text{-}LYP\text{-}D3)}$[1]	$G_{(corr\text{-}B3\text{-}LYP\text{-}D3)}$[2]	$H_{(corr\text{-}B3\text{-}LYP\text{-}D3)}$[3]	$E_{(solv\text{-}M06\text{-}2X)}$[4]	IF[5]
VQM	-3783.98	0.283468	0.363861	-3786.56	
TS1	-3783.95	0.286387	0.362604	-3786.53	-49.10
TS2	-3783.94	0.284828	0.361896	-3786.51	-127.82

TS1

F	-1.51845600	4.64956900	-1.95534900
O	1.37055900	0.16431700	1.40962800
O	-0.29097000	-1.19254400	3.28939300
C	2.60721300	-0.69202000	-0.29941600
C	0.15524700	2.30206800	0.98676400
C	0.44039800	-0.84091200	0.58846700
C	-1.96567000	-0.84690800	0.01360100
C	-1.17344300	-1.23019500	2.39794400
C	3.74635700	0.90517900	1.17671600
H	3.74120800	1.56782800	2.03454800
C	-3.30536600	-1.13192800	0.42098100
C	-1.76514300	-0.41605800	-1.32093200
H	-0.76821900	-0.14093400	-1.64717800
C	-0.90111300	-0.95595900	0.99258800

C	-0.97251200	3.88501600	-0.98771700
C	-2.56482200	-1.53955700	2.72052100
H	-2.76774900	-1.79524300	3.75705200
C	2.62862300	0.19783400	0.77663900
C	0.75421800	1.41454600	2.02393400
H	0.05209500	0.98683600	2.73383900
H	1.59231000	1.85661400	2.56120700
C	4.90177900	0.72586200	0.40314800
H	5.79955000	1.27800700	0.66290600
C	3.77545000	-0.88051800	-1.04180800
H	3.78471400	-1.58061700	-1.87069800
C	0.97054800	3.09282000	0.16101100
H	2.04851000	3.08227900	0.29246900
C	-3.55446900	-1.49671000	1.79080900
H	-4.58043200	-1.72569800	2.07600700
C	0.41123800	3.88672500	-0.83687900
H	1.02162400	4.50127700	-1.48992000
C	-1.81066200	3.12636500	-0.17842200
H	-2.88270600	3.14792100	-0.33975500
C	-4.36263100	-1.02152200	-0.50730500
H	-5.37234100	-1.25176500	-0.17302200
C	-2.82145200	-0.30640200	-2.21070400
H	-2.62942900	0.03330100	-3.22558600
C	-1.23600100	2.32992600	0.80876600
H	-1.87001400	1.71137600	1.43436100
C	4.91361800	-0.15445800	-0.68690300
H	5.82721400	-0.28064400	-1.26002500
C	-4.13392700	-0.61985900	-1.81292400
H	-4.95650000	-0.53788600	-2.51796300
C	1.30083300	-1.30506400	-0.34612500
Br	0.87884800	-2.71550200	-1.52053700
TS2			
F	-3.10447200	3.57825700	-2.26949200
O	1.38984200	0.60820800	1.24214700
O	0.00028900	-0.54900900	3.38581400
C	2.85061200	-0.16641900	-0.31520700
C	-0.63181000	2.45384500	0.80439800
C	0.78876700	-0.55735800	0.64441200
C	-1.55087500	-1.24431200	0.11135900
C	-0.79415300	-0.97968400	2.50335900
C	3.51893000	1.84927700	0.92359200
H	3.32944000	2.59472100	1.68857700
C	-2.82133400	-1.73115600	0.56499400
C	-1.42039300	-0.99450300	-1.28277200

H	-0.49969700	-0.56370100	-1.65940100
C	-0.52550900	-0.93898500	1.07868400
C	-2.30103000	3.21389300	-1.26334300
C	-2.08958700	-1.53421600	2.88588300
H	-2.26738700	-1.62768300	3.95419300
C	2.61814200	0.83857900	0.63371600
C	0.23339500	2.05259400	1.86009300
H	-0.10690800	1.40931500	2.67046900
H	1.09429000	2.67303100	2.08433400
C	4.70817100	1.85195900	0.18255400
H	5.44167400	2.63184900	0.36302700
C	4.05304200	-0.16311900	-1.02831300
H	4.26180300	-0.94460300	-1.75202500
C	-0.18876500	3.38777000	-0.16638600
H	0.81619100	3.79400800	-0.09653300
C	-3.03510100	-1.89010800	1.97442100
H	-3.99510700	-2.28394000	2.30618800
C	-1.01942000	3.76911900	-1.20563400
H	-0.70794700	4.47460700	-1.96771800
C	-2.78120500	2.29925000	-0.32486300
H	-3.77871900	1.88908300	-0.43270900
C	-3.85268000	-1.99803900	-0.36289600
H	-4.80135500	-2.37619300	0.01377200
C	-2.45032700	-1.25385900	-2.16892100
H	-2.30822700	-1.04300300	-3.22634000
C	-1.93949900	1.91295800	0.70379100
H	-2.27454800	1.18613900	1.43448700
C	4.96817500	0.86028600	-0.77604100
H	5.90506200	0.88467800	-1.32498700
C	-3.67950900	-1.77404100	-1.71722900
H	-4.48138800	-1.98248900	-2.42014900
C	1.69995900	-1.03042500	-0.26050300
Br	1.57268700	-2.65124400	-1.20652400

References

1. J. Grabowski, J. M. Granda, J. Jurczak, *Org. Biomol. Chem.* **2018**, *16*, 3114.

2. J. Carreras, M. Patil, W. Thiel, M. Alcarazo, *J. Am. Chem. Soc.* **2012**, *134*, 16753.

3. R. D. Riley, B. S. N. Huchenski, K. L. Bamford, A. W. H. Speed, *Angew. Chem. Int. Ed.* **2022**, *61*, e202204088.

4. H. Iwasaki, T. Eguchi, N. Tsutsui, H. Ohno, T. Tanaka, *J. Org. Chem.* **2008**, *73*, 7145.

5. Y. Chang, C. Xie, H. Liu, S. Huang, P. Wang, W. Qin, H. Yan, *Nat. Commun.* **2022**, *13*, 1933.

6. J. Nai, J. Zhang, J. Li, H. Li, Y. Yang, M. Yang, Y. Wang, W. Gong, Z. Li, L. Li, C. Gao, *Mol. Ther. Nucleic Acids* **2022**, *27*, 349.

7. Q. Bian, L. Huang, Y. Xu, R. Wang, Y. Gu, A. Yuan, X. Ma, J. Hu, Y. Rao, D. Xu, H. Wang, J. Gao, *ACS Nano* **2021**, *15*, 19468.

8. L. Chen, W. Li, D. Qi, L. Lu, Z. Zhang, D. Wang, *Life Sci.* **2018**, *210*, 86.
