# Supplementary material for: Organocatalytic C─O Bond Cleavage and Asymmetric Transformations via [1,3]‐Sigmatropic Rearrangement
Source: Adv Sci (Weinh). 2025 May 19;12(29):70006. doi: 10.1002/advs.202504718 (PMC12362732; doi:10.1002/advs.202504718)

checkCIF/PLATON report

Structure factors have been supplied for datablock(s) 20220414-pl-3795

THIS REPORT IS FOR GUIDANCE ONLY. IF USED AS PART OF A REVIEW PROCEDURE FOR PUBLICATION, IT SHOULD NOT REPLACE THE EXPERTISE OF AN EXPERIENCED CRYSTALLOGRAPHIC REFEREE.

No syntax errors found. CIF dictionary Interpreting this report

Datablock: 20220414-pl-3795

```
Bond precision:   C-C = 0.0099 A          Wavelength=0.71073

Cell:              a=7.7388(4)     b=13.0068(8)      c=25.1855(18)
                   alpha=90        beta=90           gamma=90
Temperature:       298 K

                  Calculated                   Reported
Volume            2535.1(3)                    2535.1(3)
Space group       P 21 21 21                   P 21 21 21
Hall group        P 2ac 2ab                    P 2ac 2ab
Moiety formula    C35 H21 Br O2                C35 H21 Br O2
Sum formula       C35 H21 Br O2                C35 H21 Br O2
Mr                553.42                       553.43
Dx,g cm-3         1.450                        1.450
Z                 4                            4
Mu (mm-1)         1.654                        1.654
F000              1128.0                       1128.0
F000'             1127.28
h,k,lmax          10,17,34                     10,17,33
Nref              6753[ 3816]                  5766
Tmin,Tmax         0.693,0.743                  0.509,1.000
Tmin'             0.644

Correction method= # Reported T Limits: Tmin=0.509 Tmax=1.000
AbsCorr = MULTI-SCAN

Data completeness= 1.51/0.85       Theta(max)= 28.995

                                                      wR2(reflections)=
R(reflections)= 0.0564( 3178)                         0.1128( 5766)

S = 1.102                  Npar= 343
```

The following ALERTS were generated. Each ALERT has the format
test-name_ALERT_alert-type_alert-level.
Click on the hyperlinks for more details of the test.

Alert level C

```
PLAT234_ALERT_4_C Large Hirshfeld Difference C16      --C17      .       0.18 Ang.
PLAT341_ALERT_3_C Low Bond Precision on  C-C Bonds ...............    0.00988 Ang.
PLAT906_ALERT_3_C Large K Value in the Analysis of Variance ......      3.925 Check
PLAT934_ALERT_3_C Number of (Iobs-Icalc)/Sigma(W) > 10 Outliers ..          1 Check
```

Alert level G

```
PLAT398_ALERT_2_G Deviating  C-O-C   Angle From 120 for O1       .      105.9 Degree
PLAT791_ALERT_4_G Model has Chirality at C9          (Sohnke SpGr)          R Verify
PLAT912_ALERT_4_G Missing # of FCF Reflections Above STh/L=  0.600        439 Note
PLAT941_ALERT_3_G Average HKL Measurement Multiplicity ...........        3.2 Low
PLAT978_ALERT_2_G Number C-C Bonds with Positive Residual Density.          0 Info
```

0 **ALERT level A** = Most likely a serious problem - resolve or explain
0 **ALERT level B** = A potentially serious problem, consider carefully
4 **ALERT level C** = Check. Ensure it is not caused by an omission or oversight
5 **ALERT level G** = General information/check it is not something unexpected

0 ALERT type 1 CIF construction/syntax error, inconsistent or missing data
2 ALERT type 2 Indicator that the structure model may be wrong or deficient
4 ALERT type 3 Indicator that the structure quality may be low
3 ALERT type 4 Improvement, methodology, query or suggestion
0 ALERT type 5 Informative message, check

It is advisable to attempt to resolve as many as possible of the alerts in all categories. Often the minor alerts point to easily fixed oversights, errors and omissions in your CIF or refinement strategy, so attention to these fine details can be worthwhile. In order to resolve some of the more serious problems it may be necessary to carry out additional measurements or structure refinements. However, the purpose of your study may justify the reported deviations and the more serious of these should normally be commented upon in the discussion or experimental section of a paper or in the "special_details" fields of the CIF. checkCIF was carefully designed to identify outliers and unusual parameters, but every test has its limitations and alerts that are not important in a particular case may appear. Conversely, the absence of alerts does not guarantee there are no aspects of the results needing attention. It is up to the individual to critically assess their own results and, if necessary, seek expert advice.

Publication of your CIF in IUCr journals

A basic structural check has been run on your CIF. These basic checks will be run on all CIFs submitted for publication in IUCr journals (*Acta Crystallographica*, *Journal of Applied Crystallography*, *Journal of Synchrotron Radiation*); however, if you intend to submit to *Acta Crystallographica Section C* or *E* or *IUCrData*, you should make sure that full publication checks are run on the final version of your CIF prior to submission.

Publication of your CIF in other journals

Please refer to the *Notes for Authors* of the relevant journal for any special instructions relating to CIF submission.

PLATON version of 19/02/2022; check.def file version of 19/02/2022

Datablock 20220414-pl-3795 - ellipsoid plot

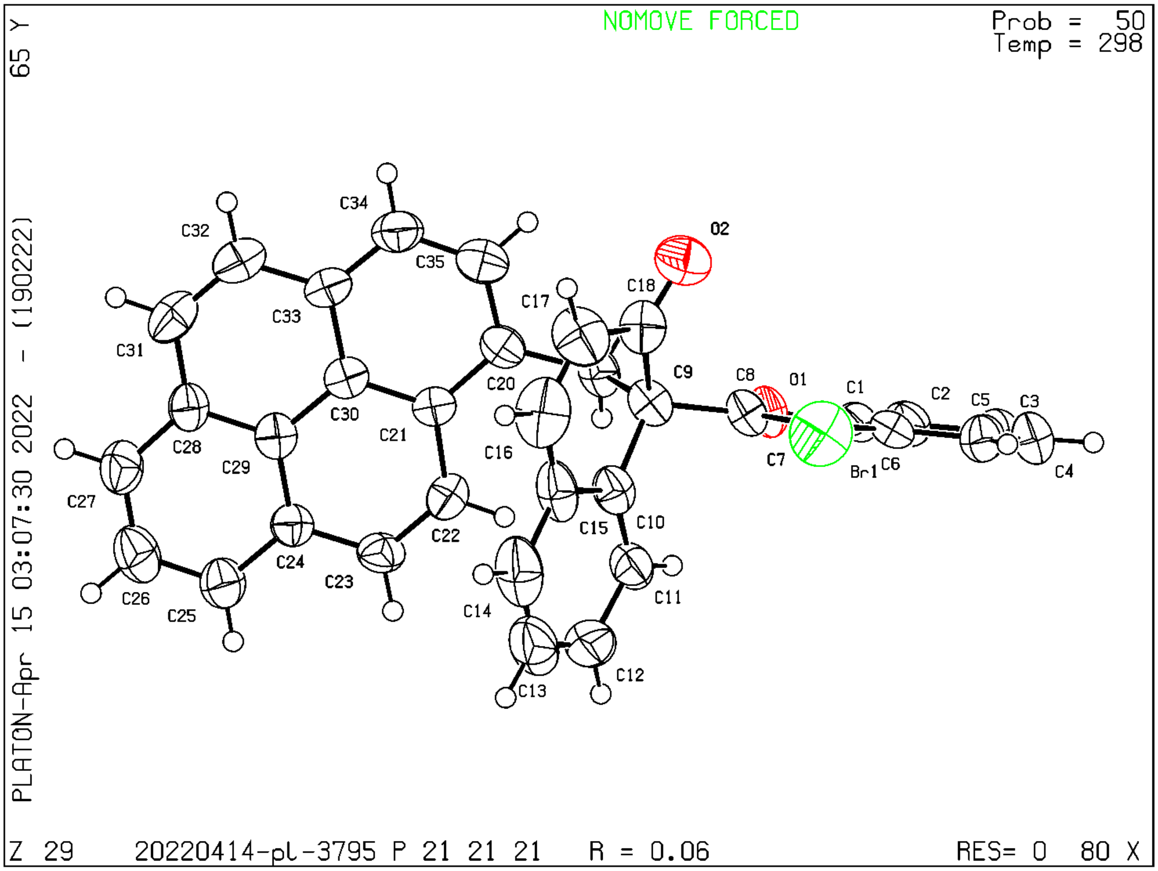

Supplement: Supplementary file 2 — Supporting Information [file ADVS-12-70006-s001.zip › advs70006-sup-0007-checkcif of 2u.pdf]
